# Deaminative chlorination of aminoheterocycles

**DOI:** 10.1038/s41557-021-00812-0

**Published:** 2021-12-16

**Authors:** Clément Ghiazza, Teresa Faber, Alejandro Gómez-Palomino, Josep Cornella

**Affiliations:** Max-Planck-Institut für Kohlenforschung, Mülheim an der Ruhr, Germany

**Keywords:** Synthetic chemistry methodology, Synthetic chemistry methodology

## Abstract

Selective modification of heteroatom-containing aromatic structures is in high demand as it permits rapid evaluation of molecular complexity in advanced intermediates. Inspired by the selectivity of deaminases in nature, herein we present a simple methodology that enables the NH_2_ groups in aminoheterocycles to be conceived as masked modification handles. With the aid of a simple pyrylium reagent and a cheap chloride source, C(*sp*^2^)‒NH_2_ can be converted into C(*sp*^2^)‒Cl bonds. The method is characterized by its wide functional group tolerance and substrate scope, allowing the modification of >20 different classes of heteroaromatic motifs (five- and six-membered heterocycles), bearing numerous sensitive motifs. The facile conversion of NH_2_ into Cl in a late-stage fashion enables practitioners to apply Sandmeyer- and Vilsmeier-type transforms without the burden of explosive and unsafe diazonium salts, stoichiometric transition metals or highly oxidizing and unselective chlorinating agents.

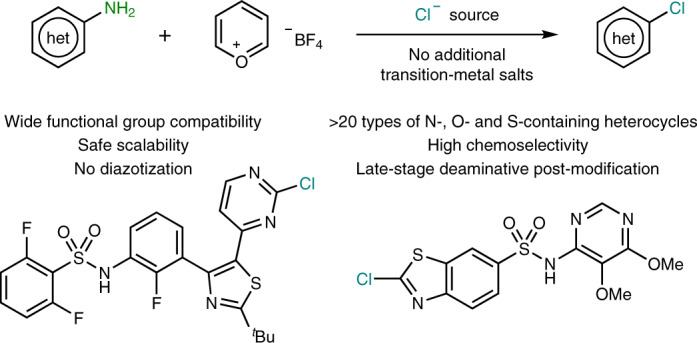

## Abstract (web summary)

Strategies for the selective modification of heteroatom-containing aromatic compounds are in high demand. Using a simple pyrylium reagent and a cheap chloride source, a method has now been developed that can selectively replace NH_2_ groups attached to heteroaromatic rings with a chlorine atom to give heteroaryl chlorides.

## Main

**Fig. 1:**
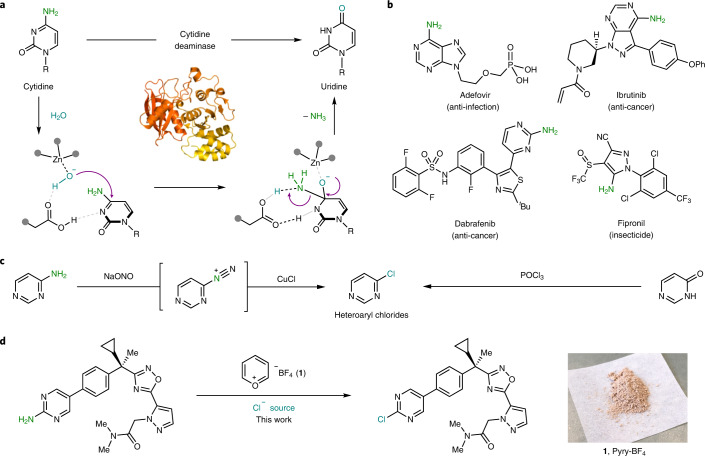
Selective modification of NH_2_ moieties, state of the art and challenges. **a**, Deaminases catalyse the conversion of cytidine to uridine using a mild and highly chemoselective exchange of NH_2_ for an OH. Enzyme picture from Protein Data Bank no. 6K63 (ref. ^32^). **b**, Compounds bearing the C(*sp*^2^)‒NH_2_ group are ubiquitous in nature and are part of many biological compounds. **c**, The widely exploited Sandmeyer- and Vilsmeier-type reactions. Sandmeyer’s limitations include explosive diazonium intermediates, limited functional group tolerance preventing its use for late-stage applications and being challenging for five-membered N-heterocycles. Vilsmeier-type reactions are relegated to early stages as they pose chemoselectivity issues, require high temperatures for five-membered rings and suffer from the low availability of the precursors. **d**, Chemoselective deaminative chlorination of aminoheterocycles using Pyry-BF_4_ 1 is applicable to molecular systems bearing various heterocyclic frameworks bearing sensitive functional groups enabling the application of this strategy in late stages without any scalability issues.

Installation and removal of chemical functionalities in complex molecular settings with a high degree of precision still remain a challenging and highly coveted milestone for organic chemists, as they would permit facile decoration of any molecular entity at will^1,2^. Nature offers a clear example of this strategy and has mastered an intricate system of enzymatic biochemical processes to enable the selective modification of specific groups in large chemical systems. A notable example is the conversion of cytidine to uridine via a deaminative process: aided by a Zn ion in the active site, these enzymes replace the NH_2_ with a molecule of H_2_O (Fig. 1a)^3,4^. From the chemical point of view, the cleavage of a C(*sp*^2^)‒N bond is energetically costly (bond dissociation energy, 102.6 ± 1.0 kcal mol^–1^) and amido groups are extremely poor leaving groups (NH_2_^–^), with virtually no examples in the synthetic world^5^. Yet, deaminases overcome these hurdles and enable the process to occur with high efficiency at room temperature. Inspired by this selective deamination process, we hypothesized that a synthetic tool that enables the precise removal of C(*sp*^2^)‒NH_2_ groups in various complex heterocyclic frameworks beyond nucleobases would be highly desirable. Aminoheterocycles are at the core of many biologically relevant compounds such as fungicides, herbicides, pharmaceutical compounds, natural products, vitamins, DNA, RNA and so on (Fig. 1b)^6–8^. Hence, the conversion of the NH_2_ group into a modular and versatile leaving group would be highly desirable. In this sense, we turned our attention to heteroaryl chlorides since they have occupied a preferential place in synthetic routes due to the myriad of robust methods available for their chemical modification^9^.

The classical Sandmeyer reaction remains the ‘textbook’ reaction when the conversion of C(*sp*^2^)‒NH_2_ into C(*sp*^2^)‒Cl is desired. Yet, it still relies on the diazotization of the amine followed by chlorination with CuCl (Fig. 1c, left)^10,11^. This protocol has been useful in simple anilines and certain aminoheterocycles. However, the strongly oxidizing and acidic conditions needed to reveal the diazonium salt present a certain incompatibility for structures containing more sensitive functionalities. More importantly, the formation of nitrogen-rich heteroaryl diazonium salts results in highly energetic compounds, which raise severe concerns about their safe handling^12,13^. Recently, awareness has also been raised by the use of aqueous nitrite solutions, which can lead to important levels of carcinogenic *N*-nitrosamines^14^. Altogether, these setbacks prohibited the translation of such a strategy in complex settings, and the perception of C(*sp*^2^)‒NH_2_ as late-stage functionalization handles was abandoned. With the aim of providing a robust protocol for C(*sp*^2^)‒Cl bond formation, we drew inspiration from the venerable Vilsmeier-type reaction that converts amides into imidoyl chlorides (Fig. 1c, right). This powerful transformation has found notorious applications in organic synthesis; yet, the use of highly reactive and unselective POCl_3_ relegates this disconnection to the early stages of the synthesis, making it incompatible with late-stage modifications^15,16^.

Herein, we present a method that merges the availability of starting materials offered by the Sandmeyer approach with the usefulness of the heteroaryl halides obtained when a Vilsmeier-type disconnection is desired. The method smoothly converts NH_2_ groups from heteroaromatic compounds into heteroaryl chlorides by means of a simple and commercially available pyrylium reagent (Pyry-BF_4_, 1)^17^ and various chloride sources (Fig. 1d). The method is demonstrated to be applicable to >20 distinct types of heterocyclic motifs, including both five- and six-membered rings containing N, O and S atoms. Importantly, the protocol is characterized by the broad functional group tolerance, thus permitting the formation of electrophilic C(*sp*^2^)‒Cl bonds onto complex pharmaceuticals, agrochemicals and natural products in a late-stage fashion. To contextualize the functional group tolerance of the reported methodology, we benchmarked our system with the state-of-the-art Sandmeyer conditions, demonstrating that our protocol is truly enabling in providing the heteroaryl chloride. Finally, we show that this reactivity is not limited to chloride anions, and we demonstrate that bromide as well as fluoride can also deliver the halogenated product.

## Results and discussion

**Fig. 2:**
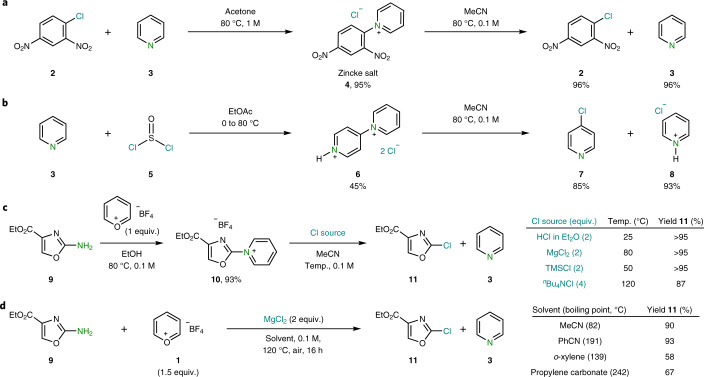
Deaminative chlorination: preliminary observations and optimized procedure. **a**, Interrupted Zincke reaction: the formation of pyridinium chloride is reversible depending on concentration and solvent. **b**, A similar behaviour to Zincke salts is observed for other heteroaromatic pyridinium chlorides. **c**, Development and implementation of a two-step deaminative chlorination of aminoheterocycles using various chloride sources and Pyry-BF_4_. Temp., temperature. **d**, Conditions for an open-flask one-pot deaminative chlorination.

Our investigations started with an interesting behaviour observed for the Zincke salt in solution. When 1-chloro-2,4-dinitrobenzene (2) is refluxed in the presence of pyridine (3), the Zincke salt precipitates in quantitative yields (4) and is easily separated and purified by filtration (Fig. 2a)^18^. Yet, when 4 is dissolved in MeCN, partial formation of 2 and 3 was observed in a 1:1 ratio, suggesting a reversible process. Increasing the temperature and diluting the solution led to the almost quantitative recovery of the 1-chloro-2,4-dinitrobenzene (2) as well as pyridine (3). Despite the wealth of literature for the reaction of nucleophiles with the Zincke salt, the use of the chloride counterion as a nucleophile to recover the parent aryl chloride 2 remained largely underexplored^19^. Although a plethora of examples of nucleophilic aromatic substitution exist with Cl regarded as the leaving group, reports on its role as nucleophile are comparatively much less exploited^20–23^. Inspired by these observations, we speculated that a similar behaviour could be translated to other arylpyridinium chloride systems, namely the product of oxidative dimerization of pyridine (6)^24^. Indeed, when compound 6 is heated in MeCN at 80 °C under diluted conditions (0.1 M), almost complete conversion to 4-chloropyridine (7) was observed with concomitant formation of pyridinium hydrochloride (8; Fig. 2b). This result immediately suggested that this phenomenon is not restricted to activated aryl moieties but also extends to heteroaromatic substrates. Our group has recently reported on the synthesis and properties of a simple pyrylium reagent (Pyry-BF_4_, 1) and its capacity to engage certain azines in Zincke-type reactivity^25–27^; although narrow in scope, the Pyry-BF_4_ displayed high chemoselectivity for amino groups. Then, we envisaged that a merger of the reactivity observed in Fig. 2b in combination with the selectivity offered by Pyry-BF_4_ would provide an opportunity for a broad and chemoselective deaminative chlorination strategy. To test this hypothesis, we subjected oxazole 9 to pyridinium formation with Pyry-BF_4_ (1), which smoothly afforded pyridinium tetrafluoroborate 10 (Fig. 2c). At this point, various chloride sources were examined to effect an anion exchange and trigger the conversion of the C(*sp*^2^)‒N bond to a C(*sp*^2^)‒Cl bond (Fig. 2c, inset table). When using an etherated HCl solution, complete chlorination was obtained at room temperature (Fig. 2c, entry 1, inset table). The use of a non-Brønsted acidic counterion such as MgCl_2_ boded well and smoothly delivered 11 at 80 °C (Fig. 2c, entry 2, inset table). Noteworthily, 2.0 equivalents of trimethylsilyl chloride (TMSCl) quantitatively furnished the desired oxazolyl chloride 11 under milder conditions (Fig. 2c, entry 3, inset table). Anticipating potential issues when translating this methodology to complex molecules bearing acid-sensitive functionalities, we tested a chloride source bearing a non-coordinating cation. The use of 4.0 equivalents of ^*n*^Bu_4_NCl displaced the pyridine and forged 11 in excellent yields (Fig. 2c, entry 4, inset table). To provide facile and practical set-ups, a single-flask operation was established, which enables the formation of the pyridinium salt and subsequent in situ chlorination in high yields with no special precautions required (Fig. 2d). In order to increase the translational potential of the methodology, we demonstrated that alternative solvents with higher boiling points than MeCN such as benzonitrile, *o*-xylenes or propylene carbonate were also amenable for this one-pot sequence.

Having established a protocol using various chloride sources, we turned our attention to explore the scope of the aminoheterocycle (Table 1). First, we engaged a panel of 4- and 2-aminopyridines (7, 12–20). Tuning the temperature turned out to be crucial to achieve satisfactory yields and accommodate functionalities such as halides (12, 14), aromatic rings (16, 17), an ester (15), a morpholine (18), a nitro (19) or a cyano (20). Diazines, including pyrimidine (21, 22) and pyridazine (23), were also smoothly converted to their chlorinated analogues in good yields. When our protocol was applied to herbicide chloridazone, high yields of the dichlorinated pyridazone 24 were obtained. Even the polyaromatic and crowded acrisorcin could be converted in 9-chloroacridine (25), albeit at 36%. Importantly, fused polycyclic substrates present at the core of biologically relevant molecules such as adenine or remdesivir, were smoothly converted to their chlorinated analogues in very good yields (26 and 27). Contrary to most strategies based on the Vilsmeier approach, our protocol boded well with five-membered heteroaromatic amines. For example, fused triazolopyridine, a motif present in certain sodium current inhibitors, smoothly afforded compound 28 in excellent yields^28^. Benzo-fused five-membered rings bearing other heteroatoms, such as benzothiazoles and isobenzothiazoles, which include drugs such as riluzole, boded well with the protocol obtaining excellent yields of the chlorinated analogues (29–31). Simple five-membered rings bearing sensitive functionalities such as ester or oximine were also well tolerated, as exemplified by 11 and 32. To further study the functional group tolerance, a variety of oxazole-based compounds bearing pendant functionalities were scrutinized. The presence of halogens (Cl, I, Br, F), pyridine, cinnamyl, cyano, methylsulfone or even aldehyde posed no difficulties for chlorination (33–40). Heterocycles bearing three heteroatoms prone to ring-opening such as thiadiazole and oxadiazole (41, 42) were smoothly chlorinated in high yields. The presence of a free secondary alcohol in 43 required the use of ^*n*^Bu_4_NCl to avoid side reactions through Mg-induced dehydration. Based on the functional group tolerance observed, we envisaged our protocol to be applicable to more complex and densely functionalized bioactive molecules. Gratifyingly, when the deaminative protocol was applied to hepatitis B pro-drug adefovir diethyl, analogue 44 was obtained in 57% yield. The anti-inflammatory amlexanox was successfully chlorinated at the two position of the pyridine motif (45). Thiazole derivatives from amoxapine, paroxetine or SF_5_-containing building blocks behaved well and were smoothly converted to the corresponding chloride in high yields (46–48). Remarkably, chlorination of the pyrimidine moiety in lipoxygenase-activating protein antagonist (BI-665915) was obtained in 38% yield (49), tolerating the presence of the rather weak N‒O bond of oxadiazole, among others. Insecticide fipronil bearing a cyano and a trifluoromethylsulfoxide embedded in a pyrazolyl ring posed no difficulties for chlorination (50). Despite the presence of a Michael acceptor and a piperidinyl moiety, chlorination of the pyrazolopyrimidine core of anti-cancer ibrutinib smoothly occurred in 87% yield (51). Benzothiazole derivative from sulfadoxine was smoothly converted, yielding 54% of 52 using HCl at room temperature. Interestingly, when MgCl_2_ at high temperature was used instead, chlorination was accompanied by a demethylation of one –OMe group from the pyrimidine (Supplementary Information, page 36 for details). Finally, another anti-cancer medication such as dabrafenib was also similarly subjected to chlorination, obtaining 40% of 53. To fully benchmark the usefulness of the protocol developed herein, some of the most critical examples were also tested under state-of-the-art Sandmeyer conditions. Whereas 24, 30 and 32 could be obtained under Sandmeyer conditions, the yields were comparably lower than when our protocol was applied. Although the Sandmeyer conditions were not optimized for each substrate and a general protocol was applied, no product was detected in any of the other ten challenging substrates tested. Decomposition of the compounds leading to intractable mixtures was the general trend.

**Table 1:**
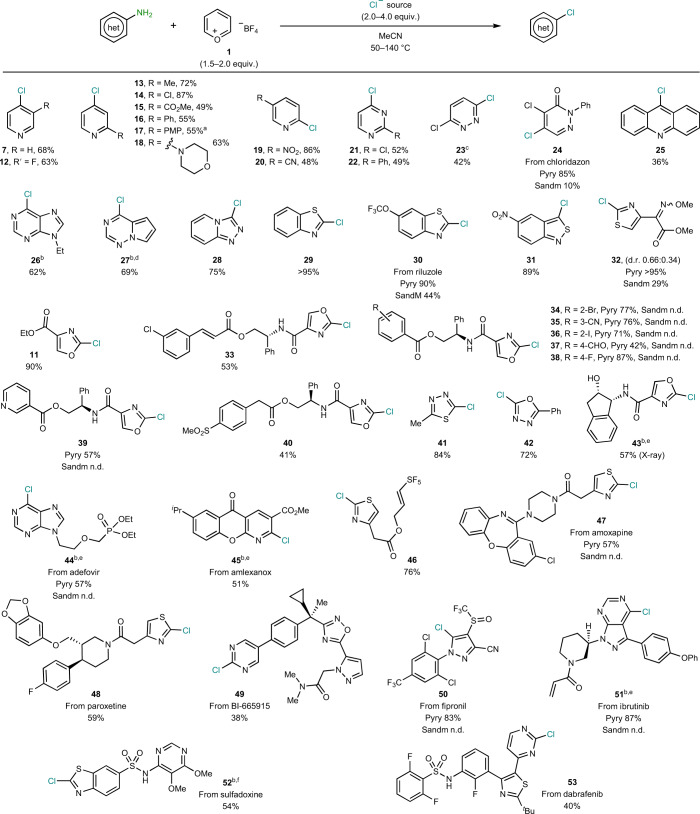
Substrate scope of the deaminative chlorination of aminoheterocycles

Unless otherwise stated, reactions were conducted in a one-step procedure as follows. Pyry, general conditions: aminoheterocycle (1 equiv.), 1 (1.5 equiv.), MgCl_2_ (2.0–5.0 equiv.), CH_3_CN (0.1 M), 50‒140 °C, 16 h. Sandm, Sandmeyer conditions: aminoheterocycle (1 equiv.), NaONO (1.1 equiv.); HCl (aq) 37% (0.2 M), 0 °C, 15 min; then, CuCl (1.3 equiv.), 25 °C, 3 h. For compounds 34–38, 2 = *ortho*, 3 = *meta*, 4 = *para.* ^a^PMP = 4-methoxylphenyl. ^b^Two-step procedure: pyridinium formation then chlorination. ^c^CsCl (4 equiv.) instead of MgCl_2_. ^d^Me_4_NCl (4 equiv.) instead of MgCl_2_. ^e^ ^*n*^Bu_4_NCl (4 equiv.) instead of MgCl_2_. ^f^HCl (1 M) in Et_2_O (4 equiv.) instead of MgCl_2_. n.d., not detected; het, heterocycle.

**Fig. 3:**
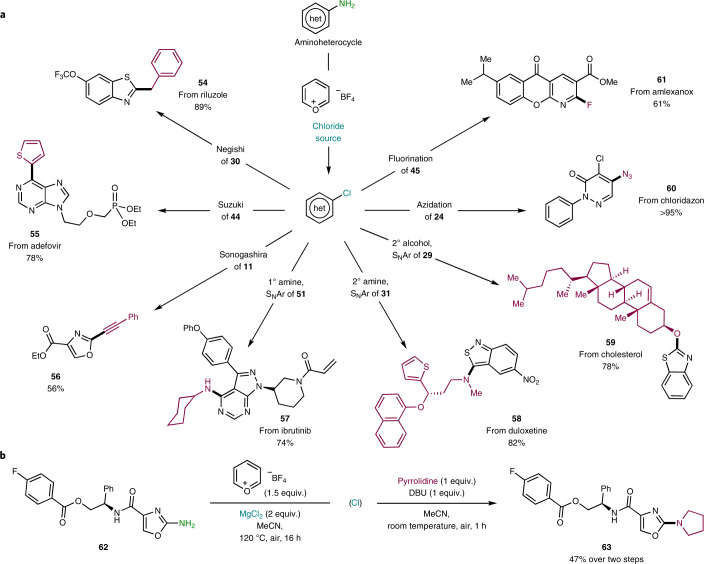
Deaminative chlorination bridges the wide availability of aminoheterocycles with the powerful chemistry of aryl chlorides. **a**, Examples of derivatization of aryl chlorides from the parent aminoheterocycle. Details for the procedures for each particular example can be found in the Supplementary Information, section VI, ‘Post-functionalization’. **b**, Telescoped deaminochlorination followed by nucleophilic aromatic amination. DBU, 1,8,-diazabicyclo[5.4.0]undec-7-ene; 1°, primary; 2°, secondary; S_N_Ar, nucleophilic aromatic substitution.

Unlocking a late-stage deaminative chlorination strategy permits the incorporation of all the well-known reactions for aryl halides to be applied in a late-stage functionalization context (Fig. 3a). For example, C(*sp*^2^)‒C(*sp*^2^), C(*sp*^2^)‒C(*sp*^3^) and C(*sp*^2^)‒C(*sp*) cross-couplings can now be carried out in substrates where such reactivity was limited (Negishi 54, Suzuki 55 and Sonogashira 56). Nucleophilic aromatic substitution, one of the most robust and utilized reactions with aryl halides, is also within reach; aliphatic amines, both primary and secondary, including duloxetine, can be incorporated in high yields through simple protocols (57, 58). Secondary alcohols such as cholesterol can be easily decorated with a benzothiazole group in good yield (59). Other nucleophiles, namely fluoride and azide, were also engaged and displaced the Cl atom, leading to valuable products (60, 61). With the aim of highlighting the practicality of the method, a telescoped three-step sequence to the pyrrolidine-functionalized analogue of 62 was attempted; 63 could be obtained in 47% yield without the need for purification of the pyridinium or chlorinated intermediate (Fig. 3b).

**Fig. 4:**
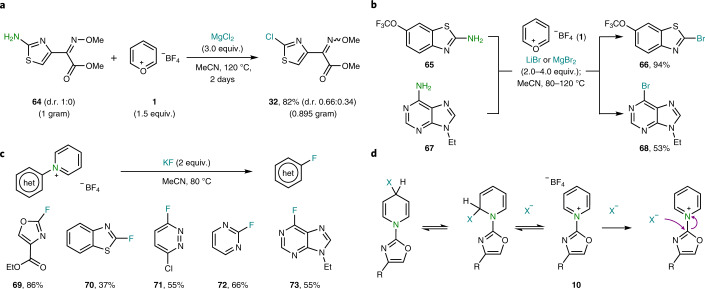
Beyond deaminative chlorination. **a**, Scalability of the process. **b**, Deaminative bromination. Reaction conditions: aminoheterocycle (1 equiv.), 1 (1.5 equiv.), EtOH (1 ml), 80 °C, 16 h; then the solvent is removed followed by the addition of the Br source (4 equiv.), CD_3_CN (1 ml), 80–120 °C, 16 h. **c**, Fluorination of pyridinium salts. Reaction conditions: pyridinium salt (1 equiv.), KF (2 equiv.), CH_3_CN (1 ml), 80 °C, 1–16 h. **d**, Tentative mechanism of the deaminative halogenation.

The scalability of the protocol was demonstrated by the gram-scale reaction performed on 64 without erosion of the yield (32; Fig. 4a). The deaminative halogenation was also extended to other halogens. For example, both five- and six-membered rings could be smoothly brominated using simple LiBr or MgBr_2_ (66 and 68; Fig. 4b). To test the limits of our protocol, fluorides were also considered as nucleophiles. In contrast to the chlorination or bromination, fluorination required preformation of the pyridinium salt, followed by the addition of rigorously dried KF (Fig. 4c). In this case, C(*sp*^2^)‒F bond formation occurs in moderate to good yields, as demonstrated by the five examples reported (69–73). When the exclusion of water is not possible, the pyridine ring-opened products are obtained (Supplementary Information, page 47 for details)^29,30^. In the case of milder nucleophiles such Cl or Br, the attack on the pyridinium might be reversible and occur in both C2 and C4 (ref. ^31^). It is not until the *ipso* position is attacked that the process becomes irreversible and ultimately leads to the formation of pyridine and the aryl chloride (Fig. 4d).

## Conclusions

Inspired by natural deaminases, we herein report a synthetic tool that enables the conversion of C(*sp*^2^)‒NH_2_ groups into C(*sp*^2^)‒Cl in high chemoselectivity under mild conditions. The use of the simple Pyry-BF_4_ selectively targets the NH_2_ attached to a heterocyclic motif and primes it for reactivity by converting it into a pyrydinium intermediate, which further reacts with a chloride source. This protocol merges the potential of the venerable Vilsmeier reaction to decorate aromatic heterocycles, with the ubiquity of aminoheterocycles, resulting in a deaminative chlorination protocol that avoids the use of explosive intermediates or strongly oxidizing reagents. As a result, the high chemoselectivity permits the chlorination of >40 compounds containing a myriad of functional groups, embedded in >20 different aminoheterocycles including five- and six-membered rings. The method is easily scalable, without the need for air-extrusion and without problematic runaway exotherms. Deaminative bromination of the amino group has also been demonstrated with a similar efficiency. More importantly, this deaminative chlorination protocol was applicable to the late-stage chlorination of various drugs and agrochemicals, thus permitting post-modification of complex structures beyond the realms of the Sandmeyer reaction. The method has been shown to extend to other halogenation processes, namely bromination and fluorination. We believe this methodology provides practitioners with an alternative tool that will permit the scrutiny of unexplored chemical space and ultimately accelerate the drug discovery process.

## Methods

### General procedure for the chlorination of amino heteroaromatic compounds

Unless otherwise specified, an 18 ml screw-capped tube under normal atmosphere is charged with pyrylium tetrafluoroborate 1 (1.5 equiv.) and MgCl_2_ (2.0 equiv.). The starting material (1.0 equiv.) is then added and directly followed by CH_3_CN (0.1 M). The resulting mixture is then stirred 5 minutes at 25 °C and then 16 hours at 120 °C. The reaction is allowed to cool to 25 °C. The crude mixture is partitioned between water and EtOAc. The aqueous layer is extracted with EtOAc (3 × 10 ml). The combined organic layers are dried over Na_2_SO_4_, concentrated to dryness and purified on silica gel to afford the desired product.

## Online content

Any methods, additional references, Nature Research reporting summaries, source data, extended data, supplementary information, acknowledgements, peer review information; details of author contributions and competing interests; and statements of data and code availability are available at 10.1038/s41557-021-00812-0.

### Supplementary information

Supplementary Information All experimental data; details of the procedures, synthesis and characterization of all new compounds; NMR spectra; high-resolution mass spectrometry data; X-ray crystallographic data; supplementary figures and tables; additional mechanistic experiments; optimization details; and troubleshooting.

Supplementary Data 1 Crystallographic data for compound 43; CCDC reference 2070324.

Supplementary Data 2 Crystallographic data for compound 52′; CCDC reference 2086010.

Supplementary Data 3 Structure factors file for compound 52′; CCDC reference 2086010.

## Data Availability

The Supplementary Information contains all experimental procedures and analytical data (^1^H, ^19^F, ^31^P, ^13^C, high-resolution mass spectrometry and crystallographic data) for all compounds, all reversibility experiments and the complete optimization study as well as a limitations section. Crystallographic data for the structures reported in this Article have been deposited at the Cambridge Crystallographic Data Centre, under deposition numbers CCDC 2070324 (43) and 2086010 (52′). Copies of the data can be obtained free of charge via https://www.ccdc.cam.ac.uk/structures/.

## pyrylium tetrafluoroborate

PubChemID: 443533822
InChIKey: SJNICXIZTGPXAU-UHFFFAOYSA-N
MDL Molfile
Chemdraw file

## 1-chloro-2,4-dinitrobenzene

PubChemID: 443533731
InChIKey: VYZAHLCBVHPDDF-UHFFFAOYSA-N
MDL Molfile
Chemdraw file

## pyridine

PubChemID: 443533732
InChIKey: JUJWROOIHBZHMG-UHFFFAOYSA-N
MDL Molfile
Chemdraw file

## 1-(2,4-dinitrophenyl)pyridin-1-ium chloride

PubChemID: 443533733
InChIKey: UYHMQTNGMUDVIY-UHFFFAOYSA-M
MDL Molfile
Chemdraw file

## thionyl chloride

PubChemID: 443533734
InChIKey: FYSNRJHAOHDILO-UHFFFAOYSA-N
MDL Molfile
Chemdraw file

## 4-(pyridyl)pyridinium chloride hydrochloride

PubChemID: 443533735
InChIKey: QZOFFMRDIRXGKJ-UHFFFAOYSA-M
MDL Molfile
Chemdraw file

## 4-chloropyridine

PubChemID: 443533736
InChIKey: PVMNPAUTCMBOMO-UHFFFAOYSA-N
MDL Molfile
Chemdraw file

## pyridine hydrochloride

PubChemID: 443533737
InChIKey: AOJFQRQNPXYVLM-UHFFFAOYSA-N
MDL Molfile
Chemdraw file

## ethyl 2-aminooxazole-4-carboxylate

PubChemID: 443533738
InChIKey: NBABLVASYFPOEV-UHFFFAOYSA-N
MDL Molfile
Chemdraw file

## ethoxycarbonyloxazole pyridinium tetrafluoroborate

PubChemID: 443533739
InChIKey: CJBMTFVGXUBBOK-UHFFFAOYSA-N
MDL Molfile
Chemdraw file

## ethyl 2-chlorooxazole-4-carboxylate

PubChemID: 443533740
InChIKey: SYWQOPRAPDMWMC-UHFFFAOYSA-N
MDL Molfile
Chemdraw file

## 4-chloro-3-fluoropyridine

PubChemID: 443533741
InChIKey: BEQUUSCRAKEKQM-UHFFFAOYSA-N
MDL Molfile
Chemdraw file

## 4-chloro-2-methylpyridine

PubChemID: 443533742
InChIKey: DAOZBJCTEPJGES-UHFFFAOYSA-N
MDL Molfile
Chemdraw file

## 2,4-dichloropyridine

PubChemID: 443533743
InChIKey: TYPVHTOETJVYIV-UHFFFAOYSA-N
MDL Molfile
Chemdraw file

## methyl 4-chloropicolinate

PubChemID: 443533744
InChIKey: VTENWIPSWAMPKI-UHFFFAOYSA-N
MDL Molfile
Chemdraw file

## 4-chloro-2-phenylpyridine

PubChemID: 443533745
InChIKey: BFRWDRFLYBYSFX-UHFFFAOYSA-N
MDL Molfile
Chemdraw file

## 4-chloro-2-(4-methoxyphenyl)pyridine

PubChemID: 443533746
InChIKey: VUYAGNFNGPJGTC-UHFFFAOYSA-N
MDL Molfile
Chemdraw file

## 4-(4-chloropyridin-2-yl)morpholine

PubChemID: 443533747
InChIKey: NEERRKPKVXOJPT-UHFFFAOYSA-N
MDL Molfile
Chemdraw file

## 2-chloro-5-nitropyridine

PubChemID: 443533748
InChIKey: BAZVFQBTJPBRTJ-UHFFFAOYSA-N
MDL Molfile
Chemdraw file

## 6-chloronicotinonitrile

PubChemID: 443533749
InChIKey: ORIQLMBUPMABDV-UHFFFAOYSA-N
MDL Molfile
Chemdraw file

## 2,4-dichloropyrimidine

PubChemID: 443533750
InChIKey: BTTNYQZNBZNDOR-UHFFFAOYSA-N
MDL Molfile
Chemdraw file

## 4-chloro-2-phenylpyrimidine

PubChemID: 443533751
InChIKey: RDLQLVAVVVLVEW-UHFFFAOYSA-N
MDL Molfile
Chemdraw file

## 3,6-dichloropyridazine

PubChemID: 443533752
InChIKey: GUSWJGOYDXFJSI-UHFFFAOYSA-N
MDL Molfile
Chemdraw file

## 4,5-dichloro-2-phenylpyridazin-3(2*H*)-one

PubChemID: 443533753
InChIKey: VKWCOHVAHQOJGU-UHFFFAOYSA-N
MDL Molfile
Chemdraw file

## 9-chloroacridine

PubChemID: 443533754
InChIKey: BPXINCHFOLVVSG-UHFFFAOYSA-N
MDL Molfile
Chemdraw file

## 6-chloro-9-ethyl-9*H*-purine

PubChemID: 443533755
InChIKey: YSKHRCGNQFWRFM-UHFFFAOYSA-N
MDL Molfile
Chemdraw file

## 4-chloropyrrolo[2,1-*f*][1,2,4]triazine

PubChemID: 443533756
InChIKey: ONTLBVKRDUFQFP-UHFFFAOYSA-N
MDL Molfile
Chemdraw file

## 3-chloro-[1,2,4]triazolo[4,3-a]pyridine

PubChemID: 443533757
InChIKey: NTMZRYSIBSLHLD-UHFFFAOYSA-N
MDL Molfile
Chemdraw file

## 2-chlorobenzo[*d*]thiazole

PubChemID: 443533758
InChIKey: BSQLQMLFTHJVKS-UHFFFAOYSA-N
MDL Molfile
Chemdraw file

## 2-chloro-6-(trifluoromethoxy)benzo[*d*]thiazole

PubChemID: 443533759
InChIKey: PPGSYGPSOLZOOU-UHFFFAOYSA-N
MDL Molfile
Chemdraw file

## 3-chloro-5-nitrobenzo[*d*]isothiazole

PubChemID: 443533760
InChIKey: JLKBGSBCVBKCBM-UHFFFAOYSA-N
MDL Molfile
Chemdraw file

## methyl 2-(2-chlorothiazol-4-yl)-2-(methoxyimino)acetate

PubChemID: 443533761
InChIKey: IPFWMKGLHCSQQP-UHFFFAOYSA-N
MDL Molfile
Chemdraw file

## (*R*)-2-(2-chlorooxazole-4-carboxamido)-2-phenylethyl (*E*)-3-(3-chlorophenyl)acrylate

PubChemID: 443533762
InChIKey: PPSGOYVESRVCKU-FVNWOWOISA-N
MDL Molfile
Chemdraw file

## (*R*)-2-(2-chlorooxazole-4-carboxamido)-2-phenylethyl 2-bromobenzoate

PubChemID: 443533763
InChIKey: LJGSENNYZDMBGB-HNNXBMFYSA-N
MDL Molfile
Chemdraw file

## (*R*)-2-(2-chlorooxazole-4-carboxamido)-2-phenylethyl 3-cyanobenzoate

PubChemID: 443533764
InChIKey: RAMLBPFQXOFANN-INIZCTEOSA-N
MDL Molfile
Chemdraw file

## (*R*)-2-(2-chlorooxazole-4-carboxamido)-2-phenylethyl 2-iodobenzoate

PubChemID: 443533765
InChIKey: QPEFKWVLOWOGLO-HNNXBMFYSA-N
MDL Molfile
Chemdraw file

## (*R*)-2-(2-chlorooxazole-4-carboxamido)-2-phenylethyl 4-formylbenzoate

PubChemID: 443533766
InChIKey: WXKJUUNXQPORBP-INIZCTEOSA-N
MDL Molfile
Chemdraw file

## (*R*)-2-(2-chlorooxazole-4-carboxamido)-2-phenylethyl 4-fluorobenzoate

PubChemID: 443533767
InChIKey: RVAINGCBQUNJMP-HNNXBMFYSA-N
MDL Molfile
Chemdraw file

## (*R*)-2-(2-chlorooxazole-4-carboxamido)-2-phenylethyl nicotinate

PubChemID: 443533768
InChIKey: QOLKJHYNPZTFQL-AWEZNQCLSA-N
MDL Molfile
Chemdraw file

## (*R*)-2-(2-chlorooxazole-4-carboxamido)-2-phenylethyl 2-(4-(methylsulfonyl)phenyl)acetate

PubChemID: 443533769
InChIKey: NJVPKSAWVFHBPB-KRWDZBQOSA-N
MDL Molfile
Chemdraw file

## 2-chloro-5-methyl-1,3,4-thiadiazole

PubChemID: 443533770
InChIKey: IPLGMBJDQYRJLJ-UHFFFAOYSA-N
MDL Molfile
Chemdraw file

## 2-chloro-5-phenyl-1,3,4-oxadiazole

PubChemID: 443533771
InChIKey: QSGNNXKJPIMRPO-UHFFFAOYSA-N
MDL Molfile
Chemdraw file

## 2-chloro-*N*-((1*R*,2*S*)-2-hydroxy-2,3-dihydro-1*H*-inden-1-yl)oxazole-4-carboxamide

PubChemID: 443533772
InChIKey: QFKZYSYYFQUGIQ-WDEREUQCSA-N
Crystallographic data
MDL Molfile
Chemdraw file

## diethyl ((2-(6-chloro-9*H*-purin-9-yl)ethoxy)methyl)phosphonate

PubChemID: 443533773
InChIKey: MSUISBLFCIYDAB-UHFFFAOYSA-N
MDL Molfile
Chemdraw file

## methyl 2-chloro-7-isopropyl-5-oxo-5*H*-chromeno[2,3-*b*]pyridine-3-carboxylate

PubChemID: 443533774
InChIKey: CYCCNOCVFFOCTE-UHFFFAOYSA-N
MDL Molfile
Chemdraw file

## (*E*)-3-(pentafluoro-λ^6^-sulfanyl)allyl 2-(2-chlorothiazol-4-yl)acetate

PubChemID: 443533775
InChIKey: ZEJRHWKXLHSCEV-HNQUOIGGSA-N
MDL Molfile
Chemdraw file

## 1-(4-(2-chlorodibenzo[*b*,*f*][1,4]oxazepin-11-yl)piperazin-1-yl)-2-(2-chlorothiazol-4-yl)ethan-1-one

PubChemID: 443533776
InChIKey: MTLRTRXCRJHFCW-UHFFFAOYSA-N
MDL Molfile
Chemdraw file

## 1-((3*S*,4*R*)-3-((benzo[*d*][1,3]dioxol-5-yloxy)methyl)-4-(4-fluorophenyl)piperidin-1-yl)-2-(2-chlorothiazol-4-yl)ethan-1-one

PubChemID: 443533777
InChIKey: DABKOAIXYJNFDT-JXFKEZNVSA-N
MDL Molfile
Chemdraw file

## (*R*)-2-(5-(3-(1-(4-(2-chloropyrimidin-5-yl)phenyl)-1-cyclopropylethyl)-1,2,4-oxadiazol-5-yl)-1*H*-pyrazol-1-yl)-*N*,*N*-dimethylacetamide

PubChemID: 443533778
InChIKey: DQZQQFUOCOKUID-DEOSSOPVSA-N
MDL Molfile
Chemdraw file

## 5-chloro-1-(2,6-dichloro-4-(trifluoromethyl)phenyl)-4-((trifluoromethyl)sulfinyl)-1*H*-pyrazole-3-carbonitrile

PubChemID: 443533779
InChIKey: HKVPVKFXPQTYCT-UHFFFAOYSA-N
MDL Molfile
Chemdraw file

## (*R*)-1-(3-(4-chloro-3-(4-phenoxyphenyl)-1*H*-pyrazolo[3,4-*d*]pyrimidin-1-yl)piperidin-1-yl)prop-2-en-1-one

PubChemID: 443533780
InChIKey: BPGOFBSYKBFRBC-GOSISDBHSA-N
MDL Molfile
Chemdraw file

## 2-chloro-*N*-(5,6-dimethoxypyrimidin-4-yl)benzo[*d*]thiazole-6-sulfonamide

PubChemID: 443533781
InChIKey: AZKLABBWNKETOR-UHFFFAOYSA-N
MDL Molfile
Chemdraw file

## 2-chloro-*N*-(5-methoxy-6-oxo-1,6-dihydropyrimidin-4-yl)benzo[*d*]thiazole-6-sulfonamide

PubChemID: 443533782
InChIKey: KDJXUIINDLFZHL-UHFFFAOYSA-N
Crystallographic data
Structure factors file
MDL Molfile
Chemdraw file

## *N*-(3-(2-(*tert*-butyl)-5-(2-chloropyrimidin-4-yl)thiazol-4-yl)-2-fluorophenyl)-2,6-difluorobenzenesulfonamide

PubChemID: 443533783
InChIKey: IOJHPWJJWDACRN-UHFFFAOYSA-N
MDL Molfile
Chemdraw file

## 2-benzyl-6-(trifluoromethoxy)benzo[*d*]thiazole

PubChemID: 443533784
InChIKey: NYQOTWXOLPEFPC-UHFFFAOYSA-N
MDL Molfile
Chemdraw file

## diethyl ((2-(6-(thiophen-2-yl)-9*H*-purin-9-yl)ethoxy)methyl)phosphonate

PubChemID: 443533785
InChIKey: LKFKEXIPYUNPMA-UHFFFAOYSA-N
MDL Molfile
Chemdraw file

## ethyl 2-(phenylethynyl)oxazole-4-carboxylate

PubChemID: 443533786
InChIKey: UHFMPKLLSCXGNO-UHFFFAOYSA-N
MDL Molfile
Chemdraw file

## (*S*)-1-(3-(4-(cyclohexylamino)-3-(4-phenoxyphenyl)-1*H*-pyrazolo[3,4-*d*]pyrimidin-1-yl)piperidin-1-yl)prop-2-en-1-one

PubChemID: 443533787
InChIKey: OPJWJJAGYUMWNM-UHFFFAOYSA-N
MDL Molfile
Chemdraw file

## (*S*)-*N*-methyl-*N*-(3-(naphthalen-1-yloxy)-3-(thiophen-2-yl)propyl)-5-nitrobenzo[*d*]isothiazol-3-amine

PubChemID: 443533788
InChIKey: XDSJQPLTSVUPJC-QFIPXVFZSA-N
MDL Molfile
Chemdraw file

## cholesteroxy-benzo[*d*]thiazole

PubChemID: 443533789
InChIKey: QTWMZVVRNJUMML-NVYQQUOZSA-N
MDL Molfile
Chemdraw file

## 5-azido-4-chloro-2-phenylpyridazin-3(2*H*)-one

PubChemID: 443533790
InChIKey: PXDKCEJJDZPWLF-UHFFFAOYSA-N
MDL Molfile
Chemdraw file

## methyl 2-fluoro-7-isopropyl-5-oxo-5*H*-chromeno[2,3-*b*]pyridine-3-carboxylate

PubChemID: 443533791
InChIKey: NLKMLUAYIZUCHB-UHFFFAOYSA-N
MDL Molfile
Chemdraw file

## (*R*)-2-(2-aminooxazole-4-carboxamido)-2-phenylethyl 4-fluorobenzoate

PubChemID: 443533792
InChIKey: UWEUPBJNHKQNLN-HNNXBMFYSA-N
MDL Molfile
Chemdraw file

## (*R*)-2-phenyl-2-(2-(pyrrolidin-1-yl)oxazole-4-carboxamido)ethyl 4-fluorobenzoate

PubChemID: 443533793
InChIKey: SCGSQMRZBULJNO-IBGZPJMESA-N
MDL Molfile
Chemdraw file

## methyl -2-(2-aminothiazol-4-yl)-2-(methoxyimino)acetate

PubChemID: 443533794
InChIKey: MJAIDGPTIFCPGD-YHYXMXQVSA-N
MDL Molfile
Chemdraw file

## 6-(trifluoromethoxy)benzo[*d*]thiazol-2-amine

PubChemID: 443533795
InChIKey: FTALBRSUTCGOEG-UHFFFAOYSA-N
MDL Molfile
Chemdraw file

## 2-bromo-6-(trifluoromethoxy)benzo[*d*]thiazole

PubChemID: 443533796
InChIKey: KDMNQLMJVHMDCQ-UHFFFAOYSA-N
MDL Molfile
Chemdraw file

## 9-ethyl-9*H*-purin-6-amine

PubChemID: 443533797
InChIKey: MUIPLRMGAXZWSQ-UHFFFAOYSA-N
MDL Molfile
Chemdraw file

## 6-bromo-9-ethyl-9*H*-purine

PubChemID: 443533798
InChIKey: PHZAXCZSIJYOQA-UHFFFAOYSA-N
MDL Molfile
Chemdraw file

## ethyl 2-fluorooxazole-4-carboxylate

PubChemID: 443533799
InChIKey: SMFBBQOOWDPUQN-UHFFFAOYSA-N
MDL Molfile
Chemdraw file

## 2-fluorobenzo[*d*]thiazole

PubChemID: 443533800
InChIKey: QVWCHVAUHZEAAT-UHFFFAOYSA-N
MDL Molfile
Chemdraw file

## 3-chloro-6-fluoropyridazine

PubChemID: 443533801
InChIKey: VKLXDCKTJUUITE-UHFFFAOYSA-N
MDL Molfile
Chemdraw file

## 2-fluoropyrimidine

PubChemID: 443533802
InChIKey: WAVYAFBQOXCGSZ-UHFFFAOYSA-N
MDL Molfile
Chemdraw file

## 9-ethyl-6-fluoro-9*H*-purine

PubChemID: 443533803
InChIKey: NJTRXDPBANKBMH-UHFFFAOYSA-N
MDL Molfile
Chemdraw file

## ethyl 2-(((1*E*,2*E*,4*E*)-5-hydroxypenta-2,4-dien-1-ylidene)amino)oxazole-4-carboxylate

PubChemID: 443533804
InChIKey: UPCBTCILHGIBEJ-CEDMUFSGSA-N
MDL Molfile
Chemdraw file

## 2-phenylpyridin-4-amine

PubChemID: 443533805
InChIKey: CHVKPWIABFICLK-UHFFFAOYSA-N
MDL Molfile
Chemdraw file

## 2-(4-methoxyphenyl)pyridin-4-amine

PubChemID: 443533806
InChIKey: QJMIYFPLCWCXQB-UHFFFAOYSA-N
MDL Molfile
Chemdraw file

## 2-morpholinopyridin-4-amine

PubChemID: 443533807
InChIKey: ZSXQDGMZXKOADT-UHFFFAOYSA-N
MDL Molfile
Chemdraw file

## methyl 2-amino-7-isopropyl-5-oxo-5*H*-chromeno[2,3-*b*]pyridine-3-carboxylate

PubChemID: 443533808
InChIKey: JEMBXPKPPPPJKL-UHFFFAOYSA-N
MDL Molfile
Chemdraw file

## 2-amino-*N*-(5,6-dimethoxypyrimidin-4-yl)benzo[*d*]thiazole-6-sulfonamide

PubChemID: 443533809
InChIKey: MTAOFZFIFWWQGF-UHFFFAOYSA-N
MDL Molfile
Chemdraw file

## 2-(2-aminothiazol-4-yl)-1-(4-(2-chlorodibenzo[*b*,*f*][1,4]oxazepin-11-yl)piperazin-1-yl)ethan-1-one

PubChemID: 443533810
InChIKey: DBPXALDAMSHPBJ-UHFFFAOYSA-N
MDL Molfile
Chemdraw file

## 2-(2-aminothiazol-4-yl)-1-((3*S*,4*R*)-3-((benzo[*d*][1,3]dioxol-5-yloxy)methyl)-4-(4-fluorophenyl)piperidin-1-yl)ethan-1-one

PubChemID: 443533811
InChIKey: IQLONOYTUONRMW-JXFKEZNVSA-N
MDL Molfile
Chemdraw file

## (*E*)-3-(pentafluoro-λ^6^-sulfanyl)allyl 2-(2-aminothiazol-4-yl)acetate

PubChemID: 443533812
InChIKey: QWVOAUXZSCRVMK-HNQUOIGGSA-N
MDL Molfile
Chemdraw file

## (*R*)-2-amino-*N*-(2-hydroxy-1-phenylethyl)oxazole-4-carboxamide

PubChemID: 443533813
InChIKey: VDUTVORPVYHNMR-VIFPVBQESA-N
MDL Molfile
Chemdraw file

## 2-amino-*N*-((1*R*,2*S*)-2-hydroxy-2,3-dihydro-1*H*-inden-1-yl)oxazole-4-carboxamide

PubChemID: 443533814
InChIKey: KBNNPBOURXVXDK-WDEREUQCSA-N
MDL Molfile
Chemdraw file

## (*R*)-2-(2-aminooxazole-4-carboxamido)-2-phenylethyl (*E*)-3-(3-chlorophenyl)acrylate

PubChemID: 443533815
InChIKey: HLJQXJBBFNGLCW-FVNWOWOISA-N
MDL Molfile
Chemdraw file

## (*R*)-2-(2-aminooxazole-4-carboxamido)-2-phenylethyl 2-bromobenzoate

PubChemID: 443533816
InChIKey: RWULYHYNZYTGRS-HNNXBMFYSA-N
MDL Molfile
Chemdraw file

## (*R*)-2-(2-aminooxazole-4-carboxamido)-2-phenylethyl 3-cyanobenzoate

PubChemID: 443533817
InChIKey: INPJAWRJHJGZPB-INIZCTEOSA-N
MDL Molfile
Chemdraw file

## (*R*)-2-(2-aminooxazole-4-carboxamido)-2-phenylethyl 2-iodobenzoate

PubChemID: 443533818
InChIKey: JXVHHUSONIZYTE-HNNXBMFYSA-N
MDL Molfile
Chemdraw file

## (*R*)-2-(2-aminooxazole-4-carboxamido)-2-phenylethyl 4-formylbenzoate

PubChemID: 443533819
InChIKey: NYBBPQHQXOUOTJ-INIZCTEOSA-N
MDL Molfile
Chemdraw file

## (*R*)-2-(2-aminooxazole-4-carboxamido)-2-phenylethyl nicotinate

PubChemID: 443533820
InChIKey: CJKRIJOALDGOSW-AWEZNQCLSA-N
MDL Molfile
Chemdraw file

## (*R*)-2-(2-aminooxazole-4-carboxamido)-2-phenylethyl 2-(4-(methylsulfonyl)phenyl)acetate

PubChemID: 443533821
InChIKey: ASAJBGHZGQMJMC-KRWDZBQOSA-N
MDL Molfile
Chemdraw file

## References

[1] Blakemore DC (2018). Organic synthesis provides opportunities to transform drug discovery. Nat. Chem..

[2] Bode JW (2017). Chemical protein synthesis with the α-ketoacid–hydroxylamine ligation. Acc. Chem. Res..

[3] Ko T-P (2003). Crystal structure of yeast cytosine deaminase. J. Biol. Chem..

[4] Sklenak S, Yao L, Cukier RI, Yan H (2004). Catalytic mechanism of yeast cytosine deaminase: an ONIOM computational study. J. Am. Chem. Soc..

[5] Luo, Y. R. *Comprehensive Handbook of Chemical Bond Energies* (CRC Press, 2007).

[6] Baumann M, Baxendale IR (2013). An overview of the synthetic routes to the best selling drugs containing 6-membered heterocycles. Beilstein J. Org. Chem..

[7] Saini MS, Kumar A, Dwivedi J, Singh R (2013). A review: biological significances of heterocyclic compounds. Int. J. Pharm. Sci. Res..

[8] Vitaku E, Smith DT, Njardarson JT (2014). Analysis of the structural diversity, substitution patterns, and frequency of nitrogen heterocycles among U.S. FDA approved pharmaceuticals. J. Med. Chem..

[9] Levy JN (2020). Selective halogenation of pyridines using designed phosphine reagents. J. Am. Chem. Soc..

[10] He L, Qiu G, Gao Y, Wu J (2014). Removal of amino groups from anilines through diazonium salt-based reactions. Org. Biomol. Chem..

[11] Mo F, Dong G, Zhang Y, Wang J (2013). Recent applications of arene diazonium salts in organic synthesis. Org. Biomol. Chem..

[12] Firth JD, Fairlamb IJS (2020). A need for caution in the preparation and application of synthetically versatile aryl diazonium tetrafluoroborate salts. Org. Lett..

[13] Sheng M, Frurip D, Gorman D (2015). Reactive chemical hazards of diazonium salts. J. Loss Prev. Process Ind..

[14] Ashworth IW, Dirat O, Teasdale A, Whiting M (2020). Potential for the formation of *N*-nitrosamines during the manufacture of active pharmaceutical ingredients: an assessment of the risk posed by trace nitrite in water. Org. Progress Res. Dev..

[15] Su W (2010). Recent progress in the use of Vilsmeier-type reagents. Org. Prep. Proced. Int..

[16] Joule, J. A. & Mills, K. *Heterocyclic Chemistry* (John Wiley & Sons, 2008).

[17] Pang Y, Moser D, Cornella J (2020). Pyrylium salts: selective reagents for the activation of primary amino groups in organic synthesis. Synthesis.

[18] Michels TD, Rhee JU, Vanderwal CD (2008). Synthesis of δ-tributylstannyl-α,β,γ,δ-unsaturated aldehydes from pyridines. Org. Lett..

[19] Fier PS (2017). A bifunctional reagent designed for the mild, nucleophilic functionalization of pyridines. J. Am. Chem. Soc..

[20] Ullmann F, Nádai G (1908). Über die herstellung von o-nitrierten aminen aus den entsprechenden phenolderivaten. Ber. Dtsch. Chem. Ges..

[21] Bunnett JF, Zahler RE (1951). Aromatic nucleophilic substitution reactions. Chem. Rev..

[22] Attia M (1984). Linear free energy relationships in the thiophene series. Part 3. A kinetic study of chlorine-isotopic exchange between lithium chloride and some 2-chloro-3-nitro-5-X-thiophenes. J. Chem. Soc.Perkin Trans..

[23] Sekiguchi S, Ishikura H, Hirosawa Y, Ono N (1990). Aromatic nucleophilic substitution reactions of 1-dialkylamino-substituted activated benzenes with various amines in dimethyl sulfoxide. Tetrahedron.

[24] Gurinov AA, Lesnichin SB, Limbach H-H, Shenderovich IG (2014). How short is the strongest hydrogen bond in the proton-bound homodimers of pyridine derivatives?. J. Phys. Chem. A.

[25] Gómez‐Palomino A, Cornella J (2019). Selective late‐stage sulfonyl chloride formation from sulfonamides enabled by Pyry‐BF_4_. Angew. Chem. Int. Ed..

[26] Moser D (2018). Selective functionalization of aminoheterocycles by a pyrylium salt. Angew. Chem. Int. Ed..

[27] Pérez-Palau M, Cornella J (2020). Synthesis of sulfonyl fluorides from sulfonamides. Eur. J. Org. Chem..

[28] Koltun DO (2016). Discovery of triazolopyridine GS-458967, a late sodium current inhibitor (Late I_Na_i) of the cardiac Na_V_ 1.5 channel with improved efficacy and potency relative to ranolazine. Bioorg. Med. Chem. Lett..

[29] Becher J (1980). Synthesis and reactions of glutaconaldehyde and 5-amino-2,4-pentadienals. Synthesis.

[30] Kearney AM, Vanderwal CD (2006). Synthesis of nitrogen heterocycles by the ring opening of pyridinium salts. Angew. Chem. Int. Ed..

[31] Sowmiah S, Esperança JMSS, Rebelo LPN, Afonso CAM (2018). Pyridinium salts: from synthesis to reactivity and applications. Org. Chem. Front..

[32] Liu W (2019). Biochemical and structural analysis of the *Klebsiella pneumoniae* cytidine deaminase CDA. Biochem. Biophys. Res. Commun..

