# Author Correction: Deaminative chlorination of aminoheterocycles

**DOI:** 10.1038/s41557-022-00932-1

**Published:** 2022-03-30

**Authors:** Clément Ghiazza, Teresa Faber, Alejandro Gómez-Palomino, Josep Cornella

**Affiliations:** Max-Planck-Institut für Kohlenforschung, Mülheim an der Ruhr, Germany

**Keywords:** Synthetic chemistry methodology, Synthetic chemistry methodology

Correction to: *Nature Chemistry* 10.1038/s41557-021-00812-0, published online 16 December 2021.

In the version of this Article originally published, there was an error in the structure of compound **31** in Table 1 (and pages 25, 158, 159 of the Supplementary Information) and in the structure of compound **58** in Fig. 3 (and pages 41, 252, 253 of the Supplementary Information). In the original version, the nitrogen and sulfur atoms in the benzoisothiazole ring present in each structure were the wrong way around. This has now been corrected.

