# Supplementary material for: Deaminative chlorination of aminoheterocycles
Source: Nat Chem. 2021 Dec 16;14(1):78–84. doi: 10.1038/s41557-021-00812-0 (PMC8755540; doi:10.1038/s41557-021-00812-0)
Supplement: Supplementary file 1 — All experimental data; details of the procedures, synthesis and characterization of all new compounds; NMR spectra; high-resolution mass spectrometry data; X-ray crystallographic data; supplementary figures and tables; additional mechanistic experiments; optimization details; and troubleshooting. [file 41557_2021_812_MOESM188_ESM.pdf]

nature portfolio

https://doi.org/10.1038/s41557-021-00812-0

Supplementary information

Deaminative chlorination of aminoheterocycles

In the format provided by the authors and unedited

Deaminative chlorination of aminoheterocycles

Clément Ghiazza,[1] Teresa Faber,[1] Alejandro Gómez-Palomino,[1] Josep Cornella[1]*

[1]Max-Planck-Institut für Kohlenforschung, Kaiser-Wilhelm-Platz 1, 45470, Mülheim an der Ruhr, Germany.

cornella@kofo.mpg.de

Supporting Information

I. General considerations 2

II. Synthesis of starting materials 2

III. Reactivity of pyridinium salts 14

IV. Chlorination reactions and characterization of products 17

V. Limitations 37

VI. Post-functionalization 39

VII. Deaminative brominations 44

VIII. Fluorination of pyridinium salts 45

IX. Ring-opening at the 2-position with fluorides 47

X. Crystallographic data for compounds 43 and 52’ 48

XI. References 54

I. General considerations

Unless otherwise stated, all manipulations were performed using standard experimental techniques without any specific precautions. Anhydrous solvents were distilled from appropriate drying agents and were transferred under Argon: CH_3CN (MS), EtOH (Mg, I_2), DMSO (MS).

All reagents were used as supplied. Pyrylium tetrafluoroborate **1** (CAS: 80279-50-1) was purchased from SigmaAldrich© or prepared according to the reported procedure.[1] Chemical *BI665915* was supplied by Boehringer Ingelheim (OpnMe).

Flash chromatography: Merck silica gel 60 (40-63 µm). MS (EI): Finnigan MAT 8200 (70 eV), ESI-MS: ESQ 3000 (Bruker). Accurate mass determinations: Bruker APEX III FT-MS (7 T magnet) or MAT 95 (Finnigan).

NMR spectra were recorded using a Bruker Avance VIII-300, Bruker Avance III HD 400 MHz spectrometer, Bruker Avance III 500MHz spectrometer equipped with a 5mm BBFO probe. ^{1}H NMR spectra were referenced to the residual protons of the deuterated solvent used. ^{13}C {^{1}H} NMR spectra were referenced internally to the D-coupled ^{13}C resonances of the NMR solvent. ^{19}F NMR spectra were referenced externally to the ^{19}F resonances of $CFCl_3$. ^{31}P NMR spectra were referenced externally to the ^{31}P resonances of H_3PO_4. Chemical shifts (δ) are given in ppm, relative to deuterated solvent residual peak, and coupling constants (*J*) provided in Hz.

Melting points were recorded on a Büchi© melting point apparatus, Model B-540 (Büchi, Switzerland) and are uncorrected.

II. Synthesis of starting materials

Procedure for cross-coupling reactions with heteroaryl chlorides

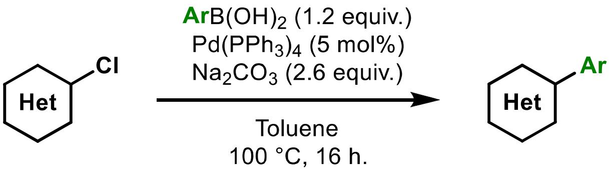

To a flam-dried schlenk flask under Ar are added $Pd(PPh_3)_4$ (225 mg, 0.195 mmol), arylboronic acid (4,675 mmol), 4-amino-2-chloropyridine (500 mg, 3.889 mmol), Na_2CO_3 2 M in water (5 mL, 10 mmol) and toluene (6.3 mL). The mixture is stirred 16 hours at 100 °C. The reaction is allowed to cool down to 25 °C and is partitioned between water and EtOAc. The aqueous layer is extracted with EtOAc (3 × 30 mL). The combined organic layers are dried over Na_2SO_4, concentrated to dryness and purified on silica gel to afford the desired products **SM1** and **SM2**.

Synthesis of 2-phenylpyridin-4-amine (SM1)

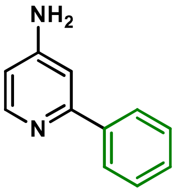

White solid, 55 %

^{1}H NMR (300 MHz, $CDCl_3$) δ 8.32 (d, *J* = 5.6 Hz, 1H), 7.91 (m, 2H), 7.44 (m, 2H), 7.39 (m, 1H), 6.96 (dd, *J* = 2.3, 0.6 Hz, 1H), 6.50 (dd, *J* = 5.6, 2.3 Hz, 1H), 4.21 (bs, 2H).

Characterization data matched with a previously reported example.[1]

Synthesis of 2-(4-methoxyphenyl)pyridin-4-amine (SM2)

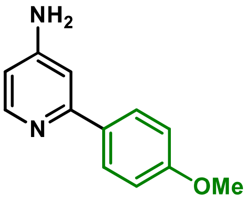

Yellow solid, 62 %

^{1}H NMR (300 MHz, DMSO-d_6) δ 8.04 (d, *J* = 5.5 Hz, 1H), 7.86 (d, *J* = 8.3 Hz, 2H), 6.99 (d, *J* = 8.3 Hz, 2H), 6.92 (d, *J* = 2.2 Hz, 1H), 6.39 (dd, *J* = 5.5, 2.2 H

z, 1H), 5.97 (bs, 2H), 3.79 (s, 3H).

Characterization data matched with a previously reported example.[1]

Procedure for the SNAr of 2-chloropyridin-4-amine (SM3)

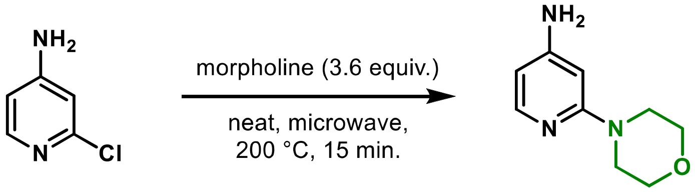

To a microwave reactor are added 2-chloropyridin-4-amine (500 mg, 3.89 mmol) and morpholine (1.22 mL, 14 mmol). The mixture is heated at 200 °C for 15 min. The reaction is allowed to cool down to 25 °C and is evaporated to dryness. The residue is then dissolved in saturated K_2CO_3 (40 mL). The aqueous phase is extracted 3 times with CH_2Cl_2 (3 × 40 mL). The combined organic layers are dried over Na_2SO_4 and concentrated to dryness afford **SM3** as a slightly yellow solid (635 mg, 91 %).

^{1}H NMR (400 MHz, $CDCl_3$) δ 7.88 (d, *J* = 5.6 Hz, 1H), 6.03 (dd, *J* = 5.6, 1.9 Hz, 1H), 5.84 (d, *J* = 1.9 Hz, 1H), 4.01 (bs, 2H), 3.81–3.78 (m, 4H), 3.43–3.41 (m, 4H).

Characterization data matched with a previously reported example.[1]

Procedure for the methylation of *amlexanox* (SM4)

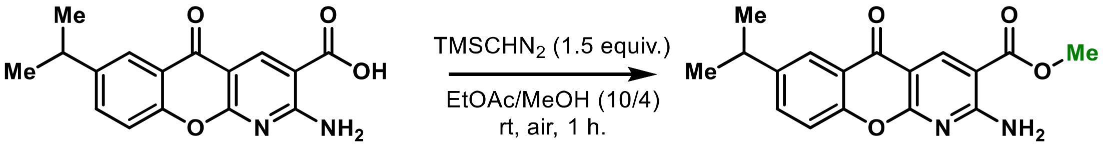

To a 50 mL schlenk flask under normal atmosphere is added *amlexanox* (201 mg, 0.67 mmol) in EtOAc and MeOH (10 and 4 mL respectively). The mixture is stirred at 25 °C and (trimethylsilyl)-diazomethane (2 M in Et_2O, 0.5 mL, 1 mmol) is added via syringe. The mixture is stirred 1 h at 25 °C. The suspension is then filtered and washed with EtOH and Et_2O to afford **SM4** as a white solid (135 mg, 64 %).

^{1}H NMR (300 MHz, DMSO-d_6) δ 8.82 (s, 1H), 8.34 (bs, 1H), 8.06 (bs, 1H), 7.93 (d, *J* = 2.3 Hz, 1H), 7.74 (dd, *J* = 8.6, 2.4 Hz, 1H), 7.53 (d, *J* = 8.6 Hz, 1H), 3.89 (s, 3H), 3.06 (hept, *J* = 6.9 Hz, 1H), 1.26 (d, *J* = 6.9 Hz, 6H).

Characterization data matched with a previously reported example.[2]

Procedure for the synthesis of 2-amino-N-(5,6-dimethoxypyrimidin-4-yl)benzo[d]thiazole-6-sulfonamide (SM5)

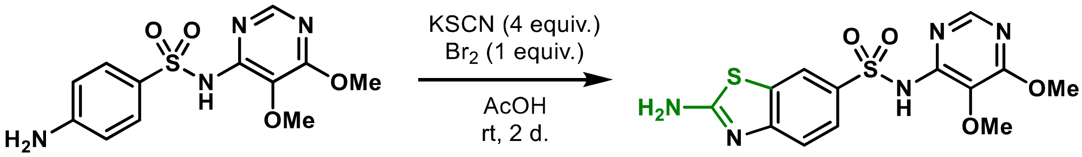

To a round bottom flask are added *sulfadoxine* (508.0 mg, 1.64 mmol), KSCN (636 mg, 6.55 mmol) and acetic acid (2 mL). A solution of dibrome (84 µL, 1.64 mmol) in acetic acid (2 mL) is slowly added to the stirred mixture at 25 °C. The reaction is stirred at 25 °C for 2 days. The reaction is diluted in EtOAC (5 mL) and carefully quenched with saturated $NaHCO_3$.* The reaction is partitioned between water and EtOAc. The aqueous layer is extracted with EtOAc (3 × 20 mL). The combined organic layers are dried over Na_2SO_4, concentrated to dryness and purified on silica gel (CH_2Cl_2:MeOH, 95:5) to afford the desired product **SM5** as a yellow solid (517 mg, 86%).

**Note: a small spatula of sodium thiosulfate was added to quench the last traces of Br_2.*

^{1}H NMR (300 MHz, MeOD) δ 8.32 (d, *J* = 2.0 Hz, 1H), 8.05 (s, 1H), 7.92 (dd, *J* = 8.6, 2.0 Hz, 1H), 7.42 (d, *J* = 8.6 Hz, 1H), 3.95 (s, 3H), 3.78 (s, 3H).

Note: The signals for –NH_2 and –NH are not detected due to rapid exchange with MeOD.

^{13}C NMR (75 MHz, MeOD) δ 163.2, 157.2, 151.8, 151.8, 140.6, 134.1, 132.1, 127.1, 122.8, 118.1, 60.8, 54.6.

Note: 1 C is missing.

HRMS (ESI) calculated $[M+H]^+$ 368.048174 , measured 368.048120.

Procedure for the amidation of 2-(2-aminothiazol-4-yl)acetic acid with *amoxapine* (SM6)

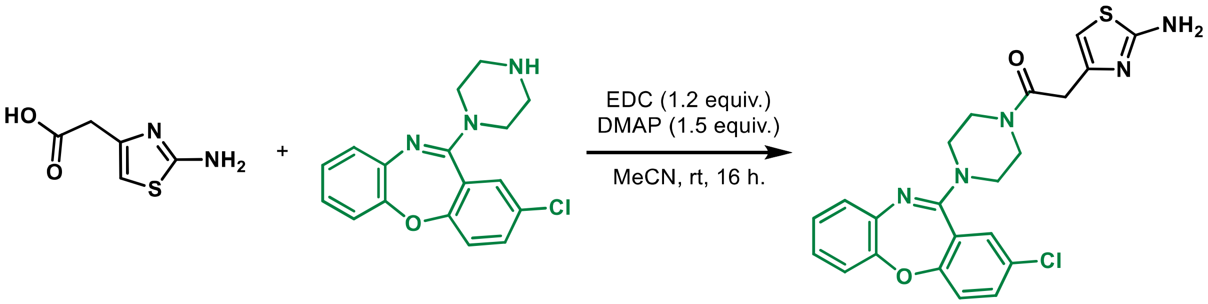

To a round bottom flask are added 2-(2-aminothiazol-4-yl)acetic acid (69.6 mg, 0.44 mmol), *amoxapine* (138.0 mg, 0.44 mmol), *N*-ethyl-*N*'-(dimethylaminopropyl)-carbodiimide hydrochloride (101.2 mg, 0.53 mmol), 4-(dimethylamino)-pyridine (80.6 mg, 0.66 mmol) and acetonitrile (2 mL). The reaction is stirred at 25 °C for 16 hours. The reaction is partitioned between water and EtOAc. The aqueous layer is extracted with EtOAc (3 × 10 mL). The combined organic layers are dried over Na_2SO_4, concentrated to dryness and purified on silica gel (gradient CH_2Cl_2:MeOH, 95:5) to afford the desired product **SM6** as a white solid (200 mg, quant.).

^{1}H NMR (300 MHz, $CDCl_3$) δ 7.40 (dd, *J* = 8.6, 2.6 Hz, 1H), 7.30 (d, *J* = 2.6 Hz, 1H), 7.18 (d, *J* = 8.7 Hz, 1H), 7.15–7.05 (m, 3H), 7.00 (m, 1H), 6.30 (s, 1H), 5.20 (bs, 2H), 3.83–3.60 (m, 6H), 3.55–3.41 (m, 4H).

^{13}C NMR (75 MHz, $CDCl_3$) δ 168.7, 167.9, 159.5, 158.8, 151.9, 145.6, 139.9, 132.9, 130.6, 129.0, 127.2, 126.0, 124.9, 124.8, 123.0, 120.3, 104.9, 46.0, 41.7, 37.5.

HRMS (ESI) calculated $[M+H]^+$ 454.109899, measured 454.110070.

Procedure for the amidation of 2-(2-aminothiazol-4-yl)acetic acid with *paroxetine* (SM7)

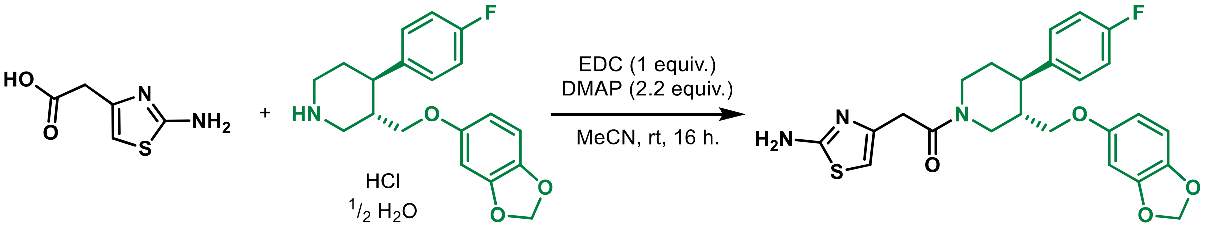

To a round bottom flask are added 2-(2-aminothiazol-4-yl)acetic acid (52.7 mg, 0.33 mmol), *paroxetine hydrochloride hemihydrate* (125.0 mg, 0.33 mmol), *N*-ethyl-*N*'-(dimethylaminopropyl)-carbodiimide hydrochloride (63.9 mg, 0.33 mmol), 4-(dimethylamino)-pyridine (89.6 mg, 0.73 mmol) and acetonitrile (2.5 mL). The reaction is stirred at 25 °C for 16 hours. The reaction is partitioned between water and EtOAc. The aqueous layer is extracted with EtOAc (3 × 10 mL). The combined organic layers are dried over Na_2SO_4, concentrated to dryness and purified on silica gel (gradient CH_2Cl_2:MeOH, 95:5) to afford the desired product **SM7** as a white solid (147 mg, 94%).

^{1}H NMR (300 MHz, $CDCl_3$) δ 7.08 (dd, *J* = 8.4, 5.3 Hz, 2H), 6.96 (m, 2H), 6.61 (dd, *J* = 8.4, 2.7 Hz, 1H), 6.34 (m, 1H), 6.26 (s, 1H), 6.12 (ddd, *J* = 8.3, 5.4, 2.5 Hz, 1H), 5.87 (d, *J* = 2.9 Hz, 2H), 5.68 (bs, 2H), 4.88 (m, 1H), 4.21 (m, 1H), 3.77–3.51 (m, 3H), 3.42 (td, *J* = 9.9, 6.1 Hz, 1H), 3.08 (m, 1H), 2.83–2.60 (m, 2H), 2.03–1.47 (m, 3H).

^{13}**C NMR** (75 MHz, $CDCl_3$) δ 168.5–168.4 (m, 2C), 161.7 (dd, ^{1}J(C,F) = 245 Hz, J = 2 Hz), 154.3 (d, J = 18 Hz), 148.3 (d, J = 7 Hz), 145.8 (d, J = 2 Hz), 141.8 (d, J = 9 Hz), 138.8 (t, J = 3 Hz), 128.9 (t, ^{3}J(C,F) = 7 Hz), 115.7 (dd, ^{2}J(C,F) = 21 Hz, J = 5 Hz), 108.0 (d, J = 5 Hz), 105.7 (d, J = 9 Hz), 104.3 (d, J = 4 Hz), 101.2 (d, J = 6 Hz), 98.1 (d, J = 11 Hz), 68.7 (d, J = 1 Hz), 48.4 (d, J = 217 Hz), 45.5–41.9 (m, 3C), 37.8 (d, J = 6 Hz), 33.9 (d, J = 51 Hz).

^{19}**F NMR** (282 MHz, $CDCl_3$) δ -115.94 (d, J = 27.0 Hz, 1F).

Note: The 2 conformations of piperidine led to a signal splitting in ^{1}H, ^{13}C as well as ^{19}F NMR.

HRMS (ESI) calculated $[M+H]^+$470.154432, measured 470.154860.

Procedure for the amidation of 2-(2-aminothiazol-4-yl)acetic acid with (*E*)-3-(pentafluoro-λ6-sulfanyl)prop-2-en-1-ol (SM8)

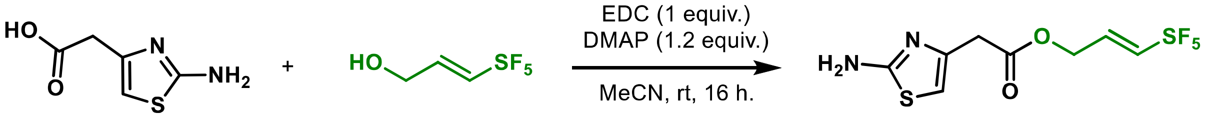

To a round bottom flask are added 2-(2-aminothiazol-4-yl)acetic acid (262.0 mg, 1.66 mmol), (*E*)-3-(pentafluoro-λ6-sulfanyl)prop-2-en-1-ol (305.0 mg, 1.66 mmol), *N*-ethyl-*N*'-(dimethylaminopropyl)-carbodiimide hydrochloride (317.5 mg, 1.66 mmol), 4-(dimethylamino)-pyridine (242.8 mg, 1.99 mmol) and acetonitrile (3 mL). The reaction is stirred at 25 °C for 16 hours. The reaction is partitioned between water and EtOAc. The aqueous layer is extracted with EtOAc (3 × 30 mL). The combined organic layers are dried over Na_2SO_4, concentrated to dryness and purified on silica gel (gradient hexanes:EtOAc, 50:50 to 30:70) to afford the desired product **SM8** as a white solid (320 mg, 60%).

^{1}**H NMR** (300 MHz, $CDCl_3$) δ 6.73 (m, 1H), 6.54 (m, 1H), 6.37 (t, J = 0.9 Hz, 1H), 5.09 (bs, 2H), 4.80 (dq, J = 4.4, 2.2 Hz, 2H), 3.64 (d, J = 0.9 Hz, 2H).

^{13}**C NMR** (75 MHz, $CDCl_3$) δ 169.4, 156.8, 144.0, 141.8 (m), 132.6 (m), 106.3, 61.0, 37.2.

^{19}**F NMR** (282 MHz, $CDCl_3$) δ 82.56 (m, 1F), 63.11 (d, ^{2}J(F,F) = 150.7 Hz, 4F).

HRMS (ESI) calculated $[M+H]^+$ 325.009840, measured 325.009620.

General procedure for the amidation of ethyl 2-aminooxazole-4-carboxylate

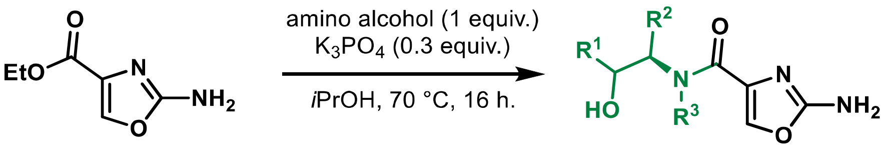

To a flam-dried schlenk flask under Ar are added K_3PO_4 (0.3 equiv.), ethyl 2-aminooxazole-4-carboxylate (1 equiv.), amino alcohol (1 equiv.) and *i*PrOH (1 mL/mmol). The mixture is stirred at 70 °C for 16 hours. The reaction is allowed to cool down to 25 °C and is partitioned between water and EtOAc. The aqueous layer is extracted with EtOAc. The combined organic layers are dried over Na_2SO_4, concentrated to dryness and purified on silica gel (gradient CH_2Cl_2:MeOH) to afford the desired products **SM9** and **SM10**.[3]

Synthesis of (*R*)-2-amino-*N*-(2-hydroxy-1-phenylethyl)oxazole-4-carboxamide (SM9)

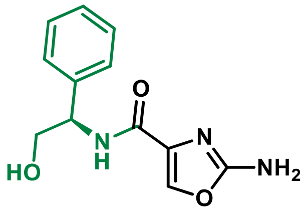

Off white solid (mp: 169.1 °C)

16.0 mmol scale, 2.2 g, 56 %

^{1}**H NMR** (300 MHz, MeOD) δ 7.72 (s, 1H), 7.39–7.22 (m, 6H), 5.11 (t, *J* = 6.0 Hz, 1H), 3.82 (dd, *J* = 6.1, 2.1 Hz, 2H).

Note: The signals for –NH$_2$ and –OH are not detected due to rapid exchange with MeOD.

^{13}**C NMR** (75 MHz, MeOD) δ 163.5, 163.2, 141.0, 136.3, 135.8, 129.5, 128.5, 127.9, 66.1, 56.6.

HRMS (ESI) calculated [M+Na]$^+$ 270.084910, measured 270.084710.

Synthesis of 2-amino-*N*-((1*R*,2*S*)-2-hydroxy-2,3-dihydro-1*H*-inden-1-yl)oxazole-4-carboxamide (SM10)

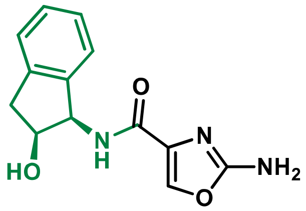

Beige solid (mp: 148.6 °C)

1.6 mmol scale, 191 mg, 46 %

^{1}**H NMR** (300 MHz, MeOD) δ 7.80 (s, 1H), 7.28–7.16 (m, 4H), 5.43 (d, *J* = 5.1 Hz, 1H), 4.60 (td, *J* = 5.1, 1.7 Hz, 1H), 3.18 (dd, *J* = 16.5, 5.1 Hz, 1H), 2.95 (dd, *J* = 16.5, 1.6 Hz, 1H).

Note: The signals for –NH$_2$, –NH, and –OH are not detected due to rapid exchange with MeOD.

^{13}**C NMR** (75 MHz, MeOD) δ 163.9, 163.1, 142.2, 141.7, 136.3, 135.8, 129.0, 127.9, 126.2, 125.3, 74.0, 58.2, 40.8.

HRMS (ESI) calculated [M+H]$^+$ 260.102966, measured 260.103220.

General procedure for the esterification of 2-amino-N-(2-hydroxyethyl)oxazole-4-carboxamide

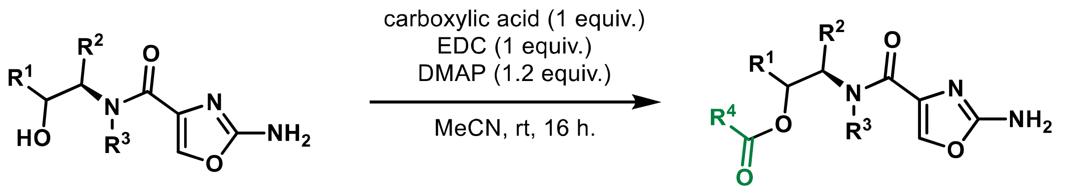

To a round bottom flask are added the starting material (50.0 mg, 0.2 mmol), carboxylic acid (0.2 mmol), *N*-ethyl-*N*'-(dimethylaminopropyl)-carbodiimide hydrochloride (38.8 mg, 0.2 mmol), 4-(dimethylamino)-pyridine (29.6 mg, 0.24 mmol) and acetonitrile (1.5 mL). The reaction is stirred at 25 °C for 16 hours. The reaction is partitioned between water and EtOAc. The aqueous layer is extracted with EtOAc (3 × 10 mL). The combined organic layers are dried over Na_2SO_4, concentrated to dryness and purified on silica gel (gradient CH_2Cl_2:MeOH) to afford the desired products **SM11** to **SM17** and **62**.

Synthesis of (*R*)-2-(2-aminooxazole-4-carboxamido)-2-phenylethyl (*E*)-3-(3-chlorophenyl)acrylate (SM11)

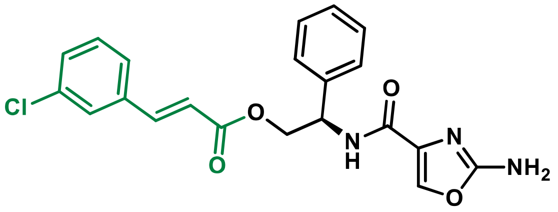

White solid (mp: 152.5 °C)

85 %

^{1}H NMR (300 MHz, $CDCl_3$) δ 7.70 (s, 1H), 7.58 (d, *J* = 16.0 Hz, 1H), 7.47 (m, 1H), 7.41–7.26 (m, 9H), 6.40 (d, *J* = 16.0 Hz, 1H), 5.51 (td, *J* = 7.6, 4.8 Hz, 1H), 4.84 (s, 2H), 4.59 (dd, *J* = 11.5, 7.1 Hz, 1H), 4.50 (dd, *J* = 11.5, 4.8 Hz, 1H).

^{13}C NMR (75 MHz, $CDCl_3$) δ 166.6, 160.9, 159.5, 144.1, 138.3, 136.2, 135.5, 135.4, 135.1, 130.4, 130.3, 129.0, 128.2, 128.0, 126.9, 126.5, 119.0, 66.5, 52.1.

HRMS (ESI) calculated $[M+Na]^+$ 434.087803, measured 434.087680.

Synthesis of (*R*)-2-(2-aminooxazole-4-carboxamido)-2-phenylethyl 2-bromobenzoate (SM12)

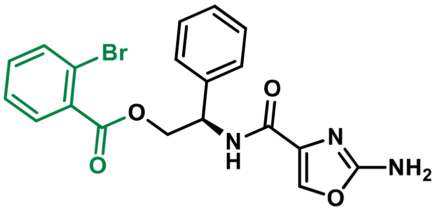

White solid (mp: 120.4 °C)

77 %

^{1}H NMR (300 MHz, $CDCl_3$) δ 7.72–7.67 (m, 2H), 7.62 (m, 1H), 7.48 (d, *J* = 8.7 Hz, 1H), 7.43–7.26 (m, 7H), 5.59 (ddd, *J* = 8.7, 6.7, 4.6 Hz, 1H), 4.87 (s, 2H), 4.72 (dd, *J* = 11.4, 6.7 Hz, 1H), 4.62 (dd, *J* = 11.5, 4.7 Hz, 1H).

^{13}C NMR (75 MHz, $CDCl_3$) δ 166.2, 161.0, 159.9, 138.0, 135.3, 135.1, 134.3, 132.9, 131.7, 131.6, 128.9, 128.1, 127.2, 126.8, 121.7, 67.4, 51.8.

HRMS (ESI) calculated $[M+Na]^+$ 452.021650, measured 452.021330.

Synthesis of (*R*)-2-(2-aminooxazole-4-carboxamido)-2-phenylethyl 3-cyanobenzoate (SM13)

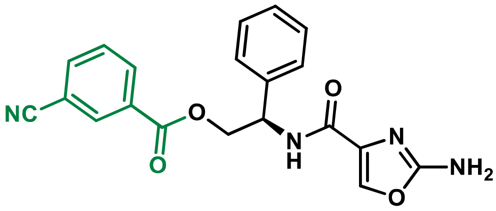

White sticky solid (mp: 179.5 °C)

82 %

^{1}H NMR (300 MHz, DMSO-d_6) δ 8.43 (d, *J* = 9.0 Hz, 1H), 8.23 (t, *J* = 1.6 Hz, 1H), 8.14 (ddt, *J* = 14.8, 7.8, 1.4 Hz, 2H), 7.85 (s, 1H), 7.74 (t, *J* = 7.8 Hz, 1H), 7.50 (m, 2H), 7.37 (m, 2H), 7.30 (m, 1H), 6.83 (bs, 2H), 5.49 (td, *J* = 8.5, 5.4 Hz, 1H), 4.63 (m, 2H).

^{13}C NMR (75 MHz, DMSO-d_6) δ 163.9, 160.9, 160.7, 139.1, 136.7, 135.2, 134.2, 133.4, 132.6, 130.7, 130.2, 128.4, 127.6, 127.1, 117.8, 112.1, 67.0, 51.0.

HRMS (ESI) calculated $[M+Na]^+$ 399.106374, measured 399.106750.

Synthesis of (*R*)-2-(2-aminooxazole-4-carboxamido)-2-phenylethyl 2-iodobenzoate (SM14)

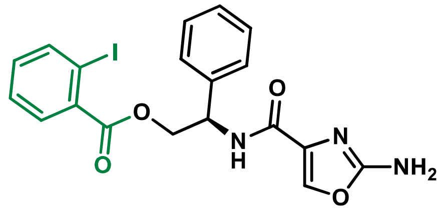

White solid (mp: 136.8 °C)

80 %

^{1}H NMR (300 MHz, MeOD) δ 7.96 (dd, *J* = 8.0, 1.1 Hz, 1H), 7.73 (s, 1H), 7.66 (dd, *J* = 7.8, 1.7 Hz, 1H), 7.47 (m, 2H), 7.42 – 7.35 (m, 3H), 7.32 (m, 1H), 7.17 (td, *J* = 7.7, 1.7 Hz, 1H), 5.55 (t, *J* = 6.5 Hz, 1H), 4.63 (d, *J* = 6.6 Hz, 2H).

Note: The signals for –NH_2 and –NH are not detected due to rapid exchange with MeOD.

^{13}C NMR (75 MHz, MeOD) δ 168.0, 163.4, 163.2, 142.3, 139.6, 136.8, 136.2, 136.1, 133.9, 131.9, 129.8, 129.1, 129.1, 128.1, 94.3, 68.3, 53.4.

HRMS (ESI) calculated $[M+Na]^+$ 500.007772, measured 500.007860.

Synthesis of (*R*)-2-(2-aminooxazole-4-carboxamido)-2-phenylethyl 4-formylbenzoate (SM15)

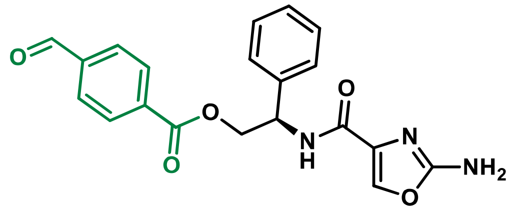

White solid (mp: 96.1 °C)

60 %

^{1}H NMR (300 MHz, DMSO-*d*$_6$) δ 10.10 (s, 1H), 8.41 (d, *J* = 9.0 Hz, 1H), 8.08 (d, *J* = 8.3 Hz, 2H), 8.03 (d, *J* = 8.5 Hz, 2H), 7.86 (s, 1H), 7.50 (m, 2H), 7.38 (m, 2H), 7.30 (m, 1H), 6.82 (bs, 2H), 5.48 (td, *J* = 8.6, 5.3 Hz, 1H), 4.70–4.57 (m, 2H).

^{13}C NMR (75 MHz, DMSO-*d*$_6$) δ 192.8, 164.8, 160.9, 160.6, 139.2, 139.2, 135.1, 134.2, 134.2, 129.7, 129.6, 128.4, 127.5, 127.0, 66.9, 51.0.

HRMS (ESI) calculated $[M+Na]^+$ 402.106040, measured 402.106620.

Synthesis of (*R*)-2-(2-aminooxazole-4-carboxamido)-2-phenylethyl nicotinate (SM16)

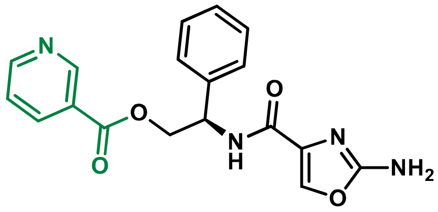

Beige solid (mp: 100.6 °C)

68 %

^{1}H NMR (300 MHz, $CDCl_3$) δ 9.19 (d, *J* = 1.8 Hz, 1H), 8.76 (dd, *J* = 5.0, 1.7 Hz, 1H), 8.27 (m, 1H), 7.70 (m, 1H), 7.44–7.26 (m, 7H), 5.60 (m, 1H), 4.74 – 4.67 (m, 4H).

^{13}C NMR (75 MHz, $CDCl_3$) δ 165.22 161.1, 159.9, 153.5, 151.0, 137.9, 137.3, 135.4, 135.1, 129.0, 128.3, 126.9, 125.8, 123.5, 67.2, 51.8.

HRMS (ESI) calculated $[M+Na]^+$ 375.106374, measured 375.106510.

Synthesis of (*R*)-2-(2-aminooxazole-4-carboxamido)-2-phenylethyl 2-(4-(methylsulfonyl)phenyl)acetate (SM17)

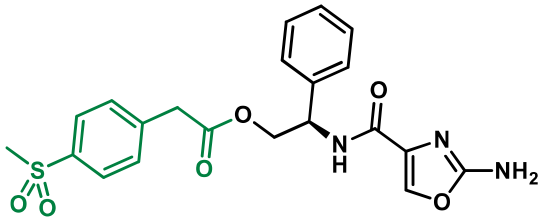

White solid (mp: 186.0 °C)

64 %

^{1}H NMR (300 MHz, DMSO-d_6) δ 8.22 (d, *J* = 9.0 Hz, 1H), 7.86 (s, 1H), 7.81 (d, *J* = 8.1 Hz, 2H), 7.47 (d, *J* = 8.1 Hz, 2H), 7.39–7.25 (m, 6H), 6.84 (s, 2H), 5.28 (td, *J* = 8.5, 5.3 Hz, 1H), 4.45–4.32 (m, 2H), 3.81 (s, 2H), 3.18 (s, 3H).

^{13}C NMR (75 MHz, DMSO-d_6) δ 170.3, 161.0, 160.5, 140.1, 139.3, 139.2, 135.2, 134.3, 130.4, 128.4, 127.5, 127.0, 126.9, 66.2, 51.0, 43.5, 39.9.

HRMS (ESI) calculated $[M+Na]^+$ 466.104328, measured 466.103980.

Synthesis of (*R*)-2-(2-aminooxazole-4-carboxamido)-2-phenylethyl 4-fluorobenzoate (62)

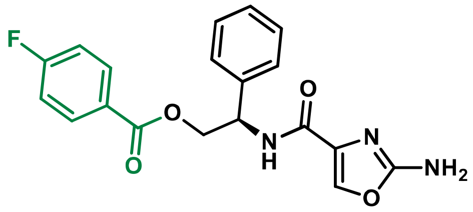

White solid (mp: 159.4 °C)

86 %

^{1}**H NMR** (300 MHz, MeOD) δ 8.00 (m, 2H), 7.73 (s, 1H), 7.47 (m, 2H), 7.37 (m, 2H), 7.31 (m, 1H), 7.15 (m, 2H), 5.54 (dd, J = 7.8, 5.4 Hz, 1H), 4.61 (m, 2H).

Note: The signals for –NH_2 and –NH are not detected due to rapid exchange with MeOD.

^{13}**C NMR** (75 MHz, MeOD) δ 168.9, 165.6, 165.1 (d, ^{1}J(C,F) = 245 Hz), 163.2, 139.6, 136.1, 136.1, 133.4 (d, ^{3}J(C,F) = 10 Hz), 129.8, 129.0, 128.0, 127.5 (d, ^{4}J(C,F) = 3 Hz), 116.5 (d, ^{2}J(C,F) = 22 Hz), 67.9, 53.5.

^{19}**F NMR** (282 MHz, $CDCl_3$) δ -105.29 (s, 3F).

HRMS (ESI) calculated $[M+Na]^+$ 392.101704, measured 392.102070.

Synthesis of methyl 2-(2-aminothiazol-4-yl)-2-(methoxyimino)acetate (64)

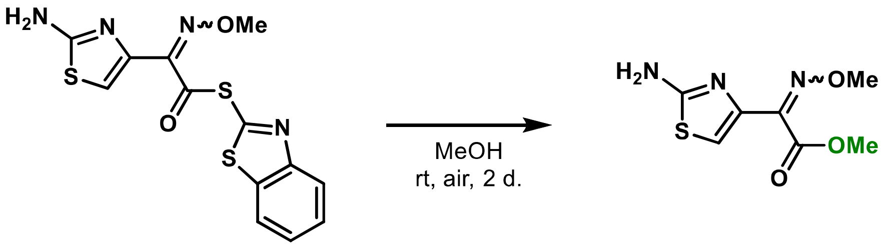

To a 18 mL screw-capped tube under normal atmosphere are added S-(benzo[d]thiazol-2-yl) 2-(2-aminothiazol-5-yl)-2-(methoxyimino)ethanethioate (144 mg, 0.41 mmol) and MeOH (5 mL). The resulting mixture is stirred 2 days at 25 °C. The solvent was then removed. The crude solid is redissolved in EtOAc (5 mL) and extracted with 2 M HCl (3 × 5 mL). The combined aqueous phases are neutralized with saturated $NaHCO_3$ and extracted with EtOAc (3 x 5 mL). The combined organic layers are dried over Na_2SO_4 and concentrated to dryness to afford **64** as a pale yellow solid (72 mg, 81 %).

^{1}**H NMR** (300 MHz, DMSO-d_6) δ 7.25 (bs, 2H), 6.93 (s, 1H), 3.87 (s, 3H), 3.80 (s, 3H).

Characterization data matched with a previously reported example.[4]

Procedure for the ethylation of adenine (67)

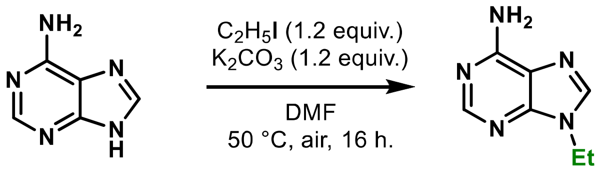

In a 250 mL round bottom flask are added adenine (2.26 g, 16.7 mmol), potassium carbonate (2.77 g, 20.0 mmol) and DMF (15 mL). The mixture is stirred at 25 °C and iodoethane is added by syringe (1.61 mL, 20.0 mmol). The reaction is then heated at 50 °C overnight. The mixture is then cooled down to 25 °C and diluted with water/brine (1:1, 50 mL). The aqueous layer is then extracted as many times as needed with EtOAc (20 mL each, extraction of the product monitored by TLC) and the combined organic layers are dried over Na_2SO_4. The crude is then concentrated to the minimum amount of solvent and Et_2O is then added. The precipitate is recovered by filtration and washed with the minimum amount of Et_2O to afford **67** as a pale yellow solid (1.23 g, 45 %). The filtrate can be concentrated again to repeat this operation.

^{1}H NMR (300 MHz, DMSO-*d*$_6$) δ 8.14 (s, 1H), 8.13 (s, 1H), 7.14 (bs, 2H), 4.16 (q, *J* = 7.2 Hz, 2H), 1.40 (t, *J* = 7.3 Hz, 3H).

Characterization data matched with a previously reported example.[5]

III. Reactivity of pyridinium salts

Synthesis and reversibility experiments of the Zincke salt (4)

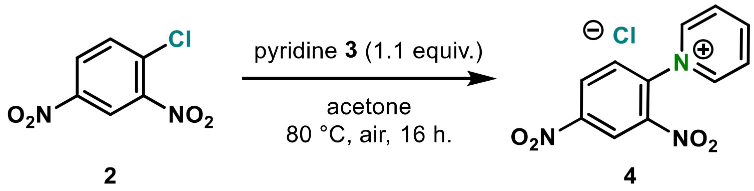

To a 18 mL screw-capped tube under normal atmosphere are added 1-chloro-2,4-dinitrobenzene **2** (413 mg, 2.04 mmol), pyridine **3** (177 mg, 2.24 mmol) and acetone (2 mL). The colorless mixture is gently stirred at 80 °C for 16 hours. After cooling down the reaction to 25 °C, acetone (2 mL) is added to the mixture and the suspension is filtered and washed with acetone to afford **4** as a white solid (532 mg, 95 %).

^{1}H NMR (300 MHz, DMSO-d_6) δ 9.50 (d, J = 5.2 Hz, 2H), 9.12 (d, J = 2.5 Hz, 1H), 8.98 (m, 2H), 8.51 (d, J = 8.7 Hz, 1H), 8.46 (m, 2H).

Characterization data matched with a previously reported example.[6]

Reversibility experiments. All experiments are conducted in a 18 mL screw-capped tube under normal atmosphere filled with 0.5 mL of solvent. The corresponding starting materials **2+3** or **4** (either 0.05 or 0.5 mmol respectively for 0.1 or 1 M) are dissolved at 25 °C before heating the reaction 16 hours at 80 °C. Yields are determined by ^{1}H NMR with mesitylene (around 1 equiv., amount determined by mass) as internal standard.

1/

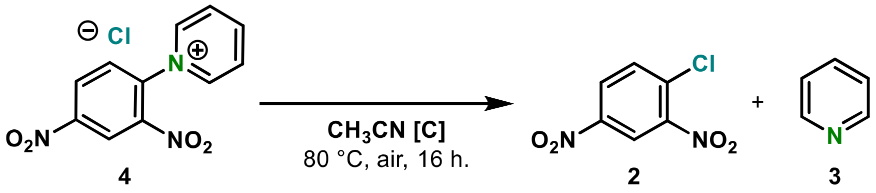

	2	3
[0.1 M] :	quant.	quant.
[1 M] :	20 %	20 %

2/

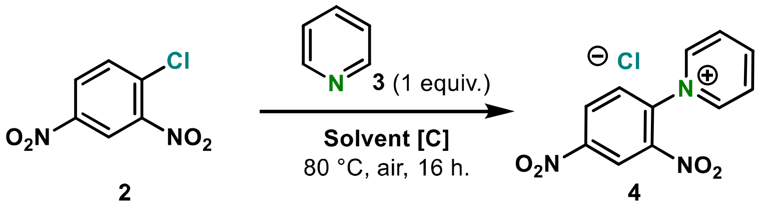

	4
Acetone [1 M] :	quant.
Acetone [0.1 M] :	0 %
CH_3CN [1 M] :	80 %
CH_3CN [0.1 M] :	0 %

Synthesis and reversibility experiments of 4-[pyridyl]pyridinium chloride hydrochloride (6)

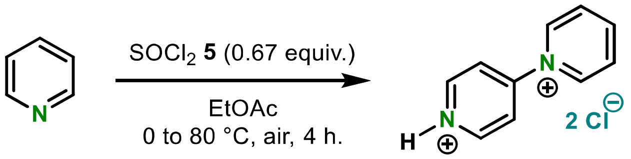

3 **6**

To a 18 mL screw-capped tube under normal atmosphere are added pyridine **3** (500 mg, 6.32 mmol) and EtOAc (300 µL). The mixture is cooled down to 0 °C and $SOCl_2$ **5** (310 µL, 4.27 mmol) is added dropwise. The reaction is then allowed to reach 25 °C and finally 80 °C for 4 hours. The reaction is cooled down to 25 °C and EtOAc as well as remaining $SOCl_2$ as distilled off under high vacuum. The black crude oil is then dissolved in EtOH (2 mL) and vigorously stirred at 60 °C for 15 minutes. The mixture is cooled down to 25 °C, filtered and washed with the minimum amount of EtOH and Et_2O (2 × 5 mL) to afford **6** as a yellow powder (230 mg, 32 %).

^{1}**H NMR** (300 MHz, DMSO-d_6) δ 9.46 (d, J = 6.1 Hz, 2H), 9.01 (d, J = 5.9 Hz, 2H), 8.87 (t, J = 7.8 Hz, 1H), 8.38 (t, J = 7.1 Hz, 2H), 8.06 (m, 2H).

Characterization data matched with a previously reported example.[7,8]

Reversibility experiment. In a 18 mL screw-capped tube under normal atmosphere are added starting material (11.5 mg, 0.05 mmol) and CD_3CN (0.5 mL). The reaction is stirred 16 hours at 80 °C. Yields are determined by ^{1}H NMR as the ratio between all components (everything was soluble).

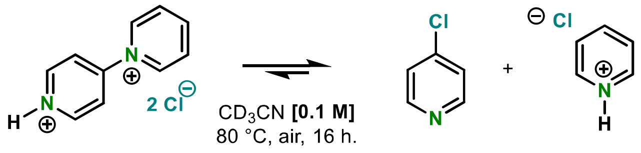

6, 15% **7**, 85% **8**, 93%

Synthesis of ethoxycarbonyloxazole pyridinium tetrafluoroborate (10)

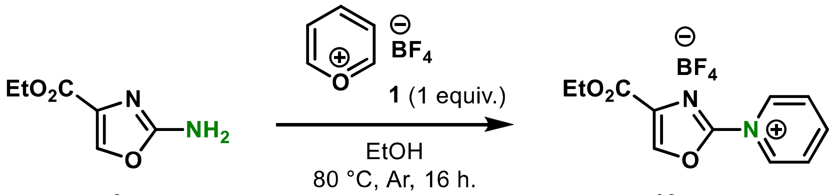

9 **10**, 93%

To a flam-dried schlenk under Ar are added **9** (78.1 mg, 0.5 mmol), pyrylium tetrafluoroborate **1** (84 mg, 0.5 mmol) in EtOH (5 mL). The flask is wrapped in aluminum foil is stirred 16 hours at 80 °C. The reaction is cooled down to 25 °C and the precipitate is filtered and quickly washed with EtOAc (minimum amount to cover the solid) and Et_2O (2 × 5 mL) to afford **10** as an orange solid (143 mg, 93 %).

^{1}**H NMR** (300 MHz, DMSO-d_6) δ 9.72 (d, J = 6.2 Hz, 2H), 9.36 (s, 1H), 8.95 (t, J = 7.8 Hz, 1H), 8.36 (t, J = 7.1 Hz, 2H), 4.39 (q, J = 7.1 Hz, 2H), 1.34 (t, J = 7.1 Hz, 3H).

^{13}**C NMR** (75 MHz, DMSO-d_6) δ 159.4, 151.8, 151.1, 146.9, 141.6, 133.8, 128.3, 61.5, 14.1.

^{19}**F NMR** (282 MHz, DMSO-d_6) δ -148.40 (s, 4F).

HRMS (ESI) calculated $[M-BF_4]^+$ 219.076417, measured 219.076560.

Optimization experiments. All experiments are conducted in a 18 mL screw-capped tube under normal atmosphere with **10** (15.3 mg, 0.05 mmol), chloride source and CH_3CN (0.5 mL). The reaction is stirred 16 hours at the corresponding temperature. Yields (product **11**) are determined by ^{1}H NMR with mesitylene (around 1 equiv., amount determined by mass) as internal standard.

Chloride source (equiv.)	Temperature (°C)	Yield of 11 (%)
$MgCl_2$ (2)	120	>95
$MgCl_2$ (2)	80	>95
$MgCl_2$ (2)	25	50
$MgCl_2$ (1)	80	>95
$MgCl_2$ (0.5)	120	60
^{n}Bu_4NCl (1)	80	27
^{n}Bu_4NCl (2)	80	41
^{n}Bu_4NCl (4)	80	47
^{n}Bu_4NCl (4)	120	87
HCl in Et_2O (1)	25	90
HCl in Et_2O (2)	25	>95
TMSCl (1)	50	70
TMSCl (2)	50	>95

As the use of CH_3CN (bp: 82 °C) at elevated temperatures raises several concerns about safety, solvents with high boiling points were also interrogated.
Conditions: **10** (15.3 mg, 0.05 mmol), $MgCl_2$ (9.5 mg, 0.01 mmol) and solvent (0.5 mL). The reaction is stirred 5 hours at 120 °C. Yields (product **11**) are determined by ^{1}H NMR with mesitylene (around 1 equiv., amount determined by mass) as internal standard.

Solvent (bp °C)	Yield of 11 (%)
NMP (202)	66
DMA (165)	78
o-xylene (139)	94
PhCN (191)	>95
^{n}BuOH (118)	0 (88 oxazolol)
DMSO (189)	decompositions
Propylene carbonate (242)	48

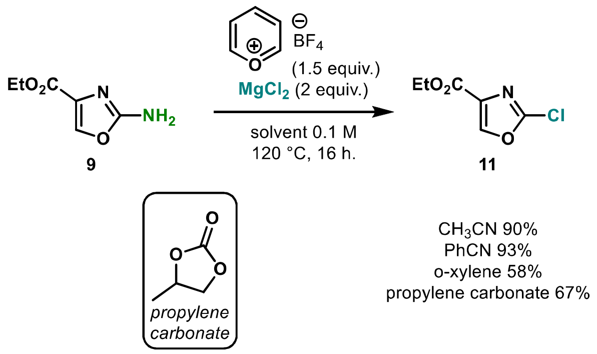

A single-flask procedure was also undertaken as follow: **1** (12.6 mg, 0.075 mmol), $MgCl_2$ (9.5 mg, 0.01 mmol), **9** (7.8 mg, 0.05 mmol) and solvent (0.5 mL). The reaction is stirred 5 minutes at 25 °C then 16 hours at 120 °C. Yields (product **11**) are determined by ^{1}H NMR with mesitylene (around 1 equiv., amount determined by mass) as internal standard.

IV. Chlorination reactions and characterization of products

General procedure for the chlorination of amino heteroaromatic compounds

Unless otherwise specified: a 18 mL screw-capped tube under normal atmosphere is charged with pyrylium tetrafluoroborate **1** (1.5 equiv.) and $MgCl_2$ (2.0 equiv.). The starting material (1.0 equiv.) is then added and directly followed by CH_3CN (0.1 M). The resulting mixture is then stirred 5 minutes at 25 °C and then 16 hours at 120 °C. The reaction is allowed to cool down to 25 °C.

NMR yields: Mesitylene (around 1 equiv., amount determined by mass) is added to the reaction. A sample of the reaction is transferred into a NMR tube and DMSO-d_6 (300 mL) is added.

Purification: The crude mixture is partitioned between water and EtOAc. The aqueous layer is extracted with EtOAc (3 × 10 mL). The combined organic layers are dried over Na_2SO_4, concentrated to dryness and purified on silica gel to afford the desired product.

Troubleshooting on deviations from the general procedure

For some substrates, the above mentioned procedure might not be adapted for different reasons listed below:

- Functional group tolerance: when the substrate is bearing sensitive functionalities such as protic groups, some undesirable side-reactions are sometimes observed (*e.g.* deoxychlorination of the secondary alcohol in **43** with $MgCl_2$). Therefore, a sequential procedure in which the pyridinium salt is firstly formed before the subsequent addition of the chloride source was developed. If applied, the corresponding procedure is detailed for each substrate.
- Temperatures: if the formation of the pyridinium salt requires usually moderate temperatures (between 50 – 80 °C), the S_NAr step necessitates sometimes elevated ones (up to 140 °C). For many substrates prepared in a one-pot fashion, we observed a significantly better yields at 120 °C and above. This can be correlated to the boiling point of pyridine (115 °C). On the other hand, some activated pyridinium salts can be converted to their chlorinated analogs at low temperatures (*e.g.* purine containing elaborated molecules as **44** or **51**). It should be mentioned that the choice of chloride source also governs the temperature as seen in the optimization table (HCl can be used at 25 °C whereas ^{n}Bu$_4$NCl requires higher temperature for a same substrate).
- Tuning the conditions: during or investigations on the scope, we noticed several trends to finely tune the reaction conditions. First of all, if the pyridinium is still detected in the crude mixture, it would mean that it is rather stable. To displace this equilibrium toward the formation of the desired product, increasing the amount of introduced chlorides showed very good results. Then, if the product is detected in a complex mixture, increasing the temperature helps. Finally, if the general procedure lead to decompositions, a sequential procedure in which the pyridinium is formed in the first place followed by the addition of chlorides often prevents side-reactions.

General procedure for the Sandmeyer reaction of amino heteroaromatic compounds

The starting heteroaromatic amine (0.1 mmol) is dissolved in concentrated HCl 37 % (0.5 mL) at 0 °C. A solution of NaONO 0.11 M (1 mL, 0.11 mmol) is added dropwise to the solution. The mixture is stirred 15 minutes at 0 °C. Then, CuCl (12.9 mg, 0.13 mmol) is added and the mixture is stirred 3 hours at 25 °C. The mixture is warmed up at 60 °C until the end of the bubbling (N_2). The reaction is carefully quenched with saturated $NaHCO_3$ and extracted with CH_2Cl_2 (3 × 5 mL). The combined organic layers are dried over Na_2SO_4 and concentrated to dryness. Mesitylene (around 1 equiv., amount determined by mass) is added to the crude. A sample of the reaction is transferred into a NMR tube with $CDCl_3$ to determine the dosed yield.

Synthesis of 4-chloropyridine (7)

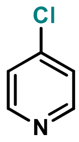

The reaction was performed at 100 °C instead of 120 °C.

^{1}H NMR – in situ (300 MHz, DMSO-d_6) δ 8.86 (d, J = 6.9 Hz, 2H), 8.13 – 8.02 (m, 2H)*.

**Note: Overlap with pyridine*

Confirmed with the commercially available product [7379-35-3].

Synthesis of ethyl 2-chlorooxazole-4-carboxylate (11)

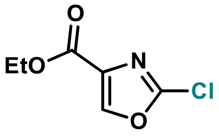

No column was required for this compound; characterized after the work up. Slightly yellow liquid

^{1}H NMR (300 MHz, $CDCl_3$) δ 8.17 (s, 1H), 4.37 (q, J = 7.2 Hz, 2H), 1.36 (t, J = 7.1 Hz, 3H).

^{13}C NMR (75 MHz, $CDCl_3$) δ 160.1, 148.3, 145.5, 135.2, 61.8, 14.3.

HRMS (EI) calculated $[M]^+$ 175.003072, measured 175.003070.

Characterization data matched with supplier data [460081-18-9].

Synthesis of 4-chloro-3-fluoropyridine (12)

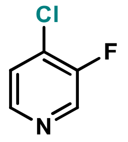

The reaction was performed at 100 °C instead of 120 °C.

^{1}H NMR – in situ (300 MHz, DMSO-d_6) δ 8.71 (d, *J* = 1.6 Hz, 1H), 8.46 (t, *J* = 6.4 Hz, 1H)*, 7.73 (t, *J* = 5.9 Hz, 1H).

**Note: Overlap with impurities*

Confirmed with the commercially available product [2546-56-7].

Synthesis of 4-chloro-2-methylpyridine (13)

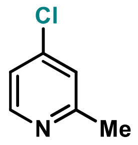

The reaction was performed at 100 °C instead of 120 °C.

^{1}H NMR – in situ (300 MHz, DMSO-d_6) δ 8.70 (d, *J* = 6.4 Hz, 1H), 8.16 – 8.03 (m, 1H)*, 7.95 (d, *J* = 6.5 Hz, 1H), 2.77 (s, 3H).

**Note: Overlap with pyridine*

HRMS (EI) calculated $[M]^+$ 128.026152, measured 128.026220.

Synthesis of 2,4-dichloropyridine (14)

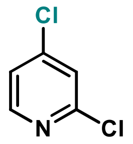

The reaction was performed at 100 °C instead of 120 °C.

^{1}H NMR – in situ (300 MHz, DMSO-d_6) δ 8.39 (d, *J* = 5.4 Hz, 1H), 7.69 (d, *J* = 1.8 Hz, 1H), 7.52 (dd, *J* = 5.4, 1.8 Hz, 1H).

HRMS (ESI) calculated $[M+H]^+$ 147.971530, measured 147.971520.

Synthesis of methyl 4-chloropicolinate (15)

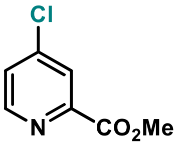

The reaction was performed at 80 °C instead of 120 °C.

^{1}H NMR – in situ (300 MHz, DMSO-d_6) δ 8.70 (d, *J* = 5.2 Hz, 1H), 8.11 (d, *J* = 2.1 Hz, 1H), 7.77 (dd, *J* = 5.3, 2.1 Hz, 1H), 3.94 (s, 3H).

Characterization data matched with a previously reported example.[9]

Synthesis of 4-chloro-2-phenylpyridine (16)

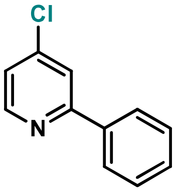

Colorless oil (volatile)

^{1}H NMR (300 MHz, $CDCl_3$) δ 8.59 (dd, *J* = 5.3, 0.6 Hz, 1H), 7.99–7.95 (m, 2H), 7.74 (dd, *J* = 1.9, 0.6 Hz, 1H), 7.52–7.341 (m, 2H), 7.25 (d, *J* = 4.9 Hz, 1H).

Characterization data matched with a previously reported example.[10]

Synthesis of 4-chloro-2-(4-methoxyphenyl)pyridine (17)

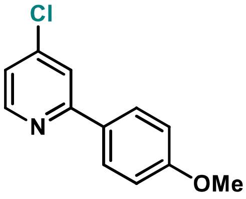

Colorless oil

^{1}H NMR (300 MHz, $CDCl_3$) δ 8.54 (dd, *J* = 5.3, 0.6 Hz, 1H), 7.96–7.91 (m, 2H), 7.67 (dd, *J* = 1.9, 0.6 Hz, 1H), 7.18 (dd, *J* = 5.3, 1.9 Hz, 1H), 7.02–6.97 (m, 2H), 3.87 (s, 3H).

Characterization data matched with a previously reported example.[11]

Synthesis of 4-(4-chloropyridin-2-yl)morpholine (18)

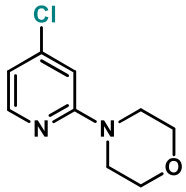

The reaction was performed at 140 °C instead of 120 °C.

^{1}H NMR – in situ (300 MHz, DMSO-d_6) δ 8.06 (d, *J* = 6.4 Hz, 1H), 7.42 (s, 1H), 6.98 (d, *J* = 6.4 Hz, 1H), 3.80-3.70 (m, 8H).

Characterization data matched with a previously reported example.[12]

Synthesis of 2-chloro-5-nitropyridine (19)

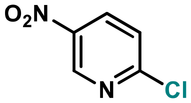

^{1}H NMR – in situ (300 MHz, DMSO-d_6) δ 9.23 (d, *J* = 2.9 Hz, 1H), 8.60 (m, 1H)*, 7.77 (d, *J* = 8.8 Hz, 1H).

**Note: Overlap with pyridine*

Characterization data matched with a previously reported example.[13]

Synthesis of 6-chloronicotinonitrile (20)

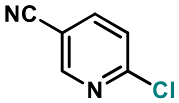

The reaction was performed at 140 °C instead of 120 °C.

^{1}H NMR – in situ (300 MHz, DMSO-d_6) δ δ 8.86 (m, 1H)*, 8.30 (dd, *J* = 8.4, 2.4 Hz, 1H), 7.73 (d, *J* = 8.4 Hz, 1H).

**Note: Overlap with pyridine*

HRMS (ESI) calculated $[M+H]^+$ 139.005770, measured 139.005750.

Synthesis of 2,4-dichloropyrimidine (21)

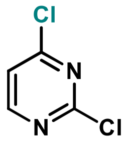

5 equivalents of $MgCl_2$ were used.

^{1}H NMR – in situ (300 MHz, DMSO-d_6) δ 8.75 (d, J = 5.3 Hz, 1H), 7.77 (d, J = 5.3 Hz, 1H).

HRMS (EI) calculated $[M]^+$ 147.958954, measured 147.959150.

Synthesis of 4-chloro-2-phenylpyrimidine (22)

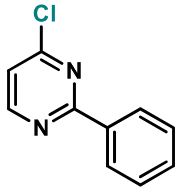

Colorless liquid

^{1}H NMR (300 MHz, $CDCl_3$) δ 8.67 (d, J = 5.2 Hz, 1H), 8.46–8.43 (m, 2H), 7.53–4.41 (m, 3H), 7.23 (d, J = 5.3 Hz, 1H).

^{13}C NMR (75 MHz, $CDCl_3$) δ 165.8, 161.7, 158.4, 136.3, 131.7, 128.8, 128.7, 119.5.

HRMS (EI) calculated $[M]^+$ 190.029225, measured 190.029520.

Synthesis of 3,6-dichloropyridazine (23)

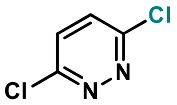

4 equivalents of CsCl were used instead of $MgCl_2$.

^{1}H NMR – in situ (300 MHz, DMSO-d_6) δ 8.01 (s, 2H).

HRMS (ESI) calculated $[M+H]^+$ 148.966778, measured 148.966910.

Synthesis of 4,5-dichloro-2-phenylpyridazin-3(2H)-one (24)

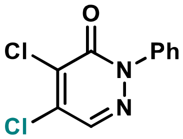

Yellow solid

^{1}H NMR (300 MHz, $CDCl_3$) δ 7.92 (s, 1H), 7.57 (d, *J* = 7.2 Hz, 2H), 7.52 – 7.40 (m, 3H).

^{13}C NMR (75 MHz, $CDCl_3$) δ 156.3, 141.0, 136.5, 136.2, 135.4, 129.0, 129.0, 125.3.

HRMS (ESI) calculated $[M+Na]^+$ 262.974938, measured 262.975020.

Synthesis of 9-chloroacridine (25)

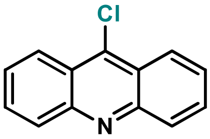

^{1}H NMR – in situ (300 MHz, DMSO-d_6) δ 8.85 (d, *J* = 8.7 Hz, 2H), 8.09 (d, *J* = 8.0 Hz, 2H)*, 7.99 (t, *J* = 7.7 Hz, 2H), 7.57 (t, *J* = 7.7 Hz, 2H).

**Note: Overlap with pyridine*

Confirmed with the commercially available product [1207-69-8].

Synthesis of 6-chloro-9-ethyl-9H-purine (26)

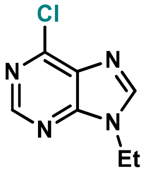

A 18 mL screw-capped tube under normal atmosphere is charged with pyrylium tetrafluoroborate **1** (25.2 mg, 0.15 mmol), **67** (16.3 mg, 0.1 mmol) and EtOH (2 mL). The resulting mixture is then stirred 5 minutes at 25 °C and then 16 hours at 80 °C (covered with aluminum foil). The reaction is allowed to cool down to 25 °C and the solvent is removed under high vacuum. The crude mixture is then dissolved in CH_3CN (2 mL) and $MgCl_2$ (19.0 mg, 0.2 mmol) is added. The resulting mixture is then stirred 5 minutes at 25 °C and then 16 hours at 120 °C. The reaction is allowed to cool down to 25 °C and concentrated to dryness. The crude mixture can be purified on a silica pad and washed several times with CH_2Cl_2 to afford the desired product **26** as a colorless liquid (11 mg, 60 %).

^{1}H NMR (300 MHz, $CDCl_3$) δ 8.74 (s, 1H), 8.14 (s, 1H), 4.36 (q, *J* = 7.3 Hz, 2H), 1.58 (t, *J* = 7.3 Hz, 3H).

^{13}C NMR (75 MHz, $CDCl_3$) δ 152.0, 151.2, 144.8, 134.7, 131.9, 39.7, 15.4.

HRMS (ESI) calculated $[M+H]^+$ 183.043198, measured 183.043180.

Synthesis of 4-chloropyrrolo[2,1-*f*][1,2,4]triazine (27)

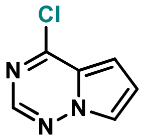

A 18 mL screw-capped tube under normal atmosphere is charged with pyrylium tetrafluoroborate **1** (25.2 mg, 0.15 mmol), aminoheterocycle (13.4 mg, 0.1 mmol) and EtOH (2 mL). The resulting mixture is then stirred 5 minutes at 25 °C and then 16 hours at 80 °C (covered with aluminum foil). The reaction is allowed to cool down to 25 °C and Me_4NCl (43.8 mg, 0.4 mmol) is added. The resulting mixture is then stirred 5 minutes at 25 °C and then 16 hours at 80 °C. After cooling down the reaction to 25 °C, the yield is determined by NMR. Attempts to purify the compound were unsuccessful and led to decompositions.

^{1}H NMR – in situ (300 MHz, DMSO-d_6) δ 7.76 (d, *J* = 2.0 Hz, 1H), 7.52 (d, *J* = 2.6 Hz, 1H), 6.86 (dd, *J* = 4.4, 1.6 Hz, 1H), 6.50 (t, *J* = 3.5 Hz, 1H).

HRMS (EI) calculated $[M]^+$ 153.008824, measured 153.008950.

Synthesis of 3-chloro-[1,2,4]triazolo[4,3-a]pyridine (28)

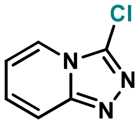

Colorless liquid

^{1}H NMR (300 MHz, $CDCl_3$) δ 8.00 (dt, *J* = 7.0, 1.2 Hz, 1H), 7.75 (dt, *J* = 9.3, 1.1 Hz, 1H), 7.33 (ddd, *J* = 9.4, 6.6, 1.2 Hz, 1H), 6.97 (td, *J* = 6.8, 1.0 Hz, 1H).

^{13}C NMR (75 MHz, $CDCl_3$) δ 150.7, 132.9, 127.8, 121.9, 116.8, 114.8.

HRMS (EI) calculated $[M]^+$ 153.008824, measured 153.009070.

Synthesis of 2-chlorobenzo[d]thiazole (29)

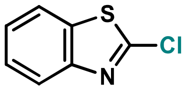

Colorless liquid (volatile)

^{1}H NMR (300 MHz, $CDCl_3$) δ 7.95 (ddd, *J* = 8.1, 1.4, 0.6 Hz, 1H), 7.78 (ddd, *J* = 7.8, 1.4, 0.6 Hz, 1H), 7.49 (td, *J* = 7.7, 1.4 Hz, 1H), 7.41 (ddd, *J* = 7.8, 7.3, 1.3 Hz, 1H).

Confirmed with the commercially available product [615-20-3].

Synthesis of 2-chloro-6-(trifluoromethoxy)benzo[d]thiazole (30)

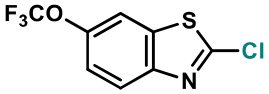

Colorless liquid (volatile)

^{1}H NMR (300 MHz, $CDCl_3$) δ 7.96 (dd, *J* = 8.9, 0.5 Hz, 1H), 7.66 (ddd, *J* = 2.5, 1.0, 0.5 Hz, 1H), 7.37 (ddq, *J* = 8.8, 2.5, 0.9 Hz, 1H).

^{13}C NMR (75 MHz, $CDCl_3$) δ 154.3, 149.7, 147.0 (q, ^3J(C,F) = 1.7 Hz), 137.0, 124.0, 120.8 (q, ^4J(C,F) = 0.67 Hz), 120.6 (q, ^1J(C,F) = 258.0 Hz), 113.9 (q, ^4J(C,F) = 1.3 Hz).

^{19}F NMR (282 MHz, $CDCl_3$) δ -58.06 (s, 3F).

HRMS (EI) calculated $[M]^+$ 252.957050, measured 252.957470.

Synthesis of 3-chloro-5-nitrobenzo[d]isothiazole (31)

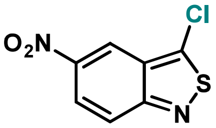

Yellow solid

^{1}H NMR (300 MHz, $CDCl_3$) δ 8.71 (d, *J* = 2.3 Hz, 1H), 8.24 (dd, *J* = 9.7, 2.3 Hz, 1H), 7.86 (d, *J* = 9.7 Hz, 1H).

^{13}C NMR (75 MHz, $CDCl_3$) δ 161.5, 155.9, 145.5, 130.9, 123.9, 123.0, 118.0.

HRMS (ESI) calculated $[M+H]^+$ 214.967653, measured 214.967750.

Synthesis of methyl (*E*)-2-(2-chlorothiazol-4-yl)-2-(methoxyimino)acetate and methyl (*Z*)-2-(2-chlorothiazol-4-yl)-2-(methoxyimino)acetate (32a and 32b)

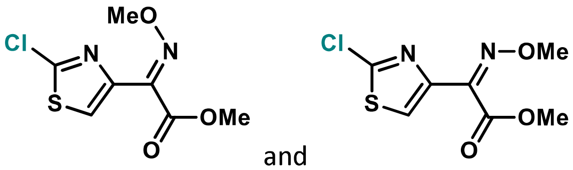

dr: 0.66:0.34

Colorless liquids

Gram scale experiment: A similar procedure was applied: To a 100 mL pressure schlenk under normal atmosphere were added pyrylium tetrafluoroborate **1** (1.170 g, 6.97 mmol), $MgCl_2$ (0.884 g, 9.29 mmol) and **64** (1.000 g, 4.65 mmol) in CH_3CN (50 mL). The resulting mixture is then stirred 5 minutes at 25 °C and then 2 days at 120 °C (conversion monitored by crude ^{1}H NMR). The reaction is allowed to cool down to 25 °C. The crude mixture is partitioned between water and EtOAc. The aqueous layer is extracted with EtOAc (3 × 100 mL). The combined organic layers are dried over Na_2SO_4, concentrated to dryness and purified on silica gel (hexanes:EtOAc, 70:30) to afford the desired products **32a** and **32b** as a pale yellow oils (0.895 mg, 82%).

A) ^{1}**H NMR** (300 MHz, $CDCl_3$) δ 7.50 (s, 1H), 4.04 (s, 3H), 3.94 (s, 3H).

A) ^{13}**C NMR** (75 MHz, $CDCl_3$) δ 162.7, 153.2, 145.2, 145.2, 120.3, 63.6, 52.9.

B) ^{1}**H NMR** (300 MHz, $CDCl_3$) δ 8.03 (s, 1H), 4.13 (s, 3H), 3.93 (s, 3H).

B) ^{13}**C NMR** (75 MHz, $CDCl_3$) δ 163.3, 151.2, 143.1, 141.4, 127.8, 64.2, 53.3.

HRMS (ESI) calculated $[M+Na]^+$ 256.975812 measured 256.975680.

Synthesis of (*R*)-2-(2-chlorooxazole-4-carboxamido)-2-phenylethyl (*E*)-3-(3-chlorophenyl)acrylate (33)

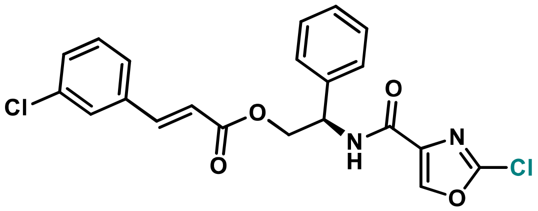

Beige solid (mp: 152.4 °C)

^{1}**H NMR** (300 MHz, $CDCl_3$) δ 8.15 (s, 1H), 7.61 (d, *J* = 16.0 Hz, 1H), 7.49 (d, *J* = 2.0 Hz, 1H), 7.45-7.28 (m, 9H), 6.42 (d, *J* = 16.0 Hz, 1H), 5.52 (ddd, *J* = 8.5, 6.9, 4.9 Hz, 1H), 4.62-4.50 (m, 2H).

^{13}**C NMR** (75 MHz, $CDCl_3$) δ 166.4, 158.9, 147.2, 144.2, 143.3, 137.8, 137.6, 136.1, 135.1, 130.4, 130.2, 129.1, 128.3, 128.0, 126.9, 126.5, 118.9, 66.3, 52.3.

HRMS (ESI) calculated $[M+Na]^+$ 453.037932, measured 453.038660.

Synthesis of (*R*)-2-(2-chlorooxazole-4-carboxamido)-2-phenylethyl 2-bromobenzoate (34)

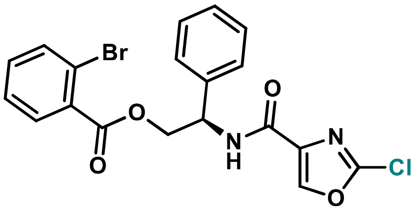

Off-white foam (mp: 151.2 °C)

^{1}**H NMR** (300 MHz, $CDCl_3$) δ 8.15 (s, 1H), 7.72 (m, 1H), 7.65 (m, 1H), 7.56 (d, *J* = 8.6 Hz, 1H), 7.44-7.28 (m, 7H), 5.60 (m, 1H), 4.754.63 (m, 2H).

^{13}**C NMR** (75 MHz, $CDCl_3$) δ 166.2, 158.9, 147.2, 143.3, 137.9, 137.6, 134.5, 133.0, 131.9, 131.8, 129.1, 128.3, 127.4, 127.0, 121.9, 67.4, 52.1.

HRMS (ESI) calculated $[M+Na]^+$ 470.971780, measured 470.972260.

Synthesis of (*R*)-2-(2-chlorooxazole-4-carboxamido)-2-phenylethyl 3-cyanobenzoate (35)

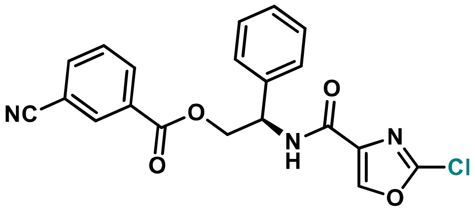

Beige solid (mp: 101.2 °C)

^{1}H NMR (300 MHz, $CDCl_3$) δ 8.25 (s, 1H), 8.20 (d, *J* = 7.9 Hz, 1H), 8.15 (s, 1H), 7.83 (d, *J* = 7.8 Hz, 1H), 7.56 (t, *J* = 7.8 Hz, 1H), 7.44-7.31 (m, 6H), 5.61 (dt, *J* = 8.5, 6.3 Hz, 1H), 4.72-4.64 (m, 2H).

^{13}C NMR (75 MHz, $CDCl_3$) δ 164.5, 159.0, 147.3, 143.3, 137.5, 137.3, 136.3, 133.9, 133.5, 131.1, 129.7, 129.3, 128.7, 126.9, 117.9, 113.2, 67.3, 52.2.

HRMS (ESI) calculated $[M+Na]^+$ 418.056503, measured 418.056640.

Synthesis of (*R*)-2-(2-chlorooxazole-4-carboxamido)-2-phenylethyl 2-iodobenzoate (36)

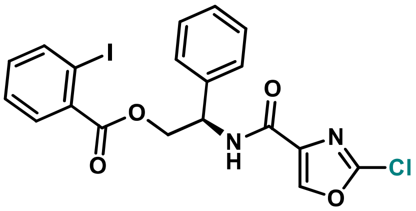

Off-white foam (mp: 118.3 °C)

^{1}H NMR (300 MHz, $CDCl_3$) 8.14 (s, 1H), 7.97 (dd, *J* = 7.9, 1.2 Hz, 1H), 7.71 (dd, *J* = 7.8, 1.7 Hz, 1H), 7.54 (d, *J* = 8.6 Hz, 1H), 7.44-7.29 (m, 6H), 7.14 (td, *J* = 7.7, 1.7 Hz, 1H), 5.61 (ddd, *J* = 8.6, 6.7, 4.7 Hz, 1H), 4.76-4.62 (m, 2H).

^{13}C NMR (75 MHz, $CDCl_3$) δ 166.4, 158.9, 147.2, 143.3, 141.4, 137.8, 137.6, 134.9, 133.0, 131.5, 129.1, 128.4, 128.1, 127.0, 94.2, 67.3, 52.2.

HRMS (ESI) calculated $[M+Na]^+$ 518.957902, measured 518.957980.

Synthesis of (*R*)-2-(2-chlorooxazole-4-carboxamido)-2-phenylethyl 4-formylbenzoate (37)

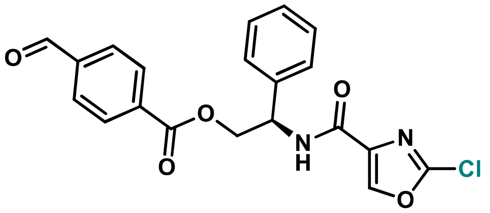

Slightly yellow solid (mp: 143.4 °C)

^{1}H NMR (300 MHz, $CDCl_3$) δ 10.09 (s, 1H), 8.15-8.12 (m, 3H), 7.93 (d, *J* = 8.1 Hz, 2H), 7.45-7.32 (m, 6H), 5.62 (dt, *J* = 8.6, 6.2 Hz, 1H), 4.75-4.64 (m, 2H).

^{13}C NMR (75 MHz, $CDCl_3$) δ 191.6, 165.5, 159.0, 147.3, 143.3, 139.5, 137.5, 137.5, 134.7, 130.5, 129.7, 129.2, 128.6, 126.9, 67.1, 52.2.

HRMS (ESI) calculated $[M+Na]^+$ 421.056169, measured 421.056580.

Synthesis of (*R*)-2-(2-chlorooxazole-4-carboxamido)-2-phenylethyl 4-fluorobenzoate (38)

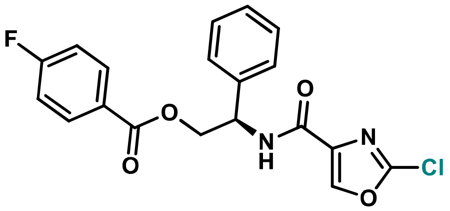

White solid (mp: 176.8 °C)

^{1}H NMR (300 MHz, $CDCl_3$) δ 8.14 (s, 1H), 8.00 (m, 2H), 7.44-7.30 (tt, J = 13.5, 6.4 Hz, 6H), 7.09 (t, J = 8.6 Hz, 2H), 5.59 (ddd, J = 8.6, 7.1, 5.3 Hz, 1H), 4.70-4.59 (m, 2H).

^{13}C NMR (75 MHz, $CDCl_3$) δ 166.1 (d, ^1J(C,F) = 255 Hz), 165.5, 159.0, 147.2, 143.2, 137.8, 137.6, 132.4 (d, ^3J(C,F) = 9 Hz), 129.2, 128.5, 126.9, 126.0 (d, ^4J(C,F) = 3 Hz), 115.8 (d, ^2J(C,F) = 22 Hz), 66.8, 52.3.

^{19}F NMR (282 MHz, $CDCl_3$) δ -105.10 (s, 3F).

HRMS (ESI) calculated $[M+Na]^+$ 411.051833, measured 411.052650.

Synthesis of (*R*)-2-(2-chlorooxazole-4-carboxamido)-2-phenylethyl nicotinate (39)

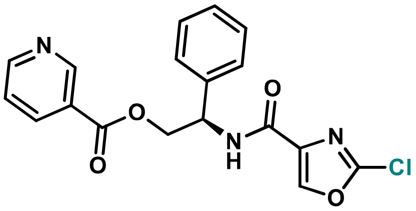

Brown solid (mp: 152.4 °C)

^{1}H NMR (300 MHz, $CDCl_3$) δ 9.16 (s, 1H), 8.76 (d, *J* = 4.6 Hz, 1H), 8.24 (d, *J* = 8.0 Hz, 1H), 8.14 (s, 1H), 7.44-7.30 (m, 7H), 5.61 (q, *J* = 6.8 Hz, 1H), 4.69 (d, *J* = 6.2 Hz, 2H).

^{13}C NMR (75 MHz, $CDCl_3$) δ 165.2, 159.0, 153.8, 151.1, 147.3, 143.3, 137.5, 137.5, 137.3, 129.2, 128.6, 126.9, 125.8, 123.5, 67.0, 52.2.

HRMS (ESI) calculated $[M+Na]^+$ 394.056503, measured 394.056660.

Synthesis of (*R*)-2-(2-chlorooxazole-4-carboxamido)-2-phenylethyl 2-(4-(methylsulfonyl)phenyl)acetate (40)

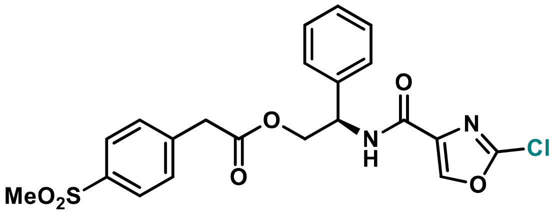

White solid (mp: 116.9 °C)

^{1}H NMR (300 MHz, $CDCl_3$) δ 8.16 (s, 1H), 7.85 (d, *J* = 8.3 Hz, 2H), 7.40 (d, *J* = 8.1 Hz, 2H), 7.37-7.20 (m, 6H), 5.45 (dt, *J* = 8.7, 6.1 Hz, 1H), 4.46 (d, *J* = 6.1 Hz, 2H), 3.71 (s, 2H), 3.03 (s, 3H).

^{13}C NMR (75 MHz, $CDCl_3$) δ 170.2, 158.9, 147.3, 143.3, 139.9, 139.6, 137.4, 137.4, 130.5, 129.1, 128.5, 127.8, 126.8, 66.7, 52.0, 44.7, 41.1.

HRMS (ESI) calculated $[M+Na]^+$ 485.054457, measured 485.055210.

Synthesis of 2-chloro-5-methyl-1,3,4-thiadiazole (41)

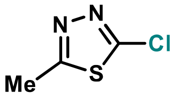

^{1}H NMR – in situ (300 MHz, DMSO-*d_6*) δ 2.72 (s, 3H).

HRMS (EI) calculated $[M]^+$ 133.969998, measured 133.970270.

Synthesis of 2-chloro-5-phenyl-1,3,4-oxadiazole (42)

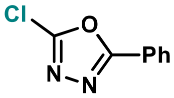

Attempts to purify the compound were unsuccessful and led to decompositions.

[1]**H NMR – in situ** (300 MHz, DMSO-*d*$_6$) δ 8.13-7.82 (m, 2H), 7.78-7.45 (m, 3H).

Note: The signals from the product are overlapping with the pyridine. However, the dosed yield remains consistent with both components.

HRMS (EI) calculated $[M]^+$ 180.008490, measured 180.008840.

Synthesis of 2-chloro-*N*-((1*R*,2*S*)-2-hydroxy-2,3-dihydro-1*H*-inden-1-yl)oxazole-4-carboxamide (43)

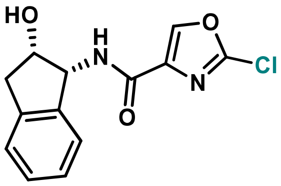

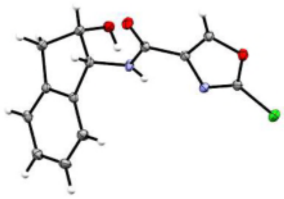

A 18 mL screw-capped tube under normal atmosphere is charged with pyrylium tetrafluoroborate **1** (25.2 mg, 0.15 mmol), **SM10** (25.9 mg, 0.01 mmol) and EtOH (2 mL). The resulting mixture is then stirred 5 minutes at 25 °C and then 16 hours at 50 °C (covered with aluminum foil). The reaction is allowed to cool down to 25 °C and the solvent is removed under high vacuum. The crude mixture is then dissolved in CH_3CN (2 mL) and $^nBu_4NCl$ (111.2 mg, 0.4 mmol) is added. The resulting mixture is then stirred 5 minutes at 25 °C and then 16 hours at 50 °C. The reaction is allowed to cool down to 25 °C and directly purified on silica gel (hexanes:EtOAc, 50:50) to afford the desired product **43** as a colorless solid (16 mg, 57 %).

[1]**H NMR** (300 MHz, $CDCl_3$) δ 8.21 (s, 1H), 7.42 (d, *J* = 7.4 Hz, 1H), 7.36-7.20 (m, 4H), 5.52 (dd, *J* = 8.6, 5.1 Hz, 1H), 4.73 (s, 1H), 3.23 (dd, *J* = 16.4, 5.3 Hz, 1H), 3.00 (d, *J* = 16.5 Hz, 1H), 2.21 (s, 1H).

[13]**C NMR** (75 MHz, $CDCl_3$) δ 160.1, 160.0, 143.3, 140.1, 140.1, 137.7, 128.7, 127.5, 125.6, 124.9, 73.9, 57.5, 39.9.

HRMS (ESI) calculated $[M+Na]^+$ 301.035039, measured 301.034780.

Synthesis of diethyl ((2-(6-chloro-9H-purin-9-yl)ethoxy)methyl)phosphonate (44)

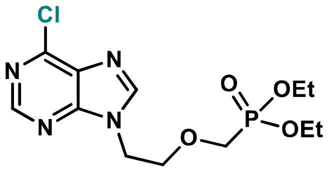

A schlenk under Ar is charged with pyrylium tetrafluoroborate **1** (25.2 mg, 0.15 mmol), *diethyl-adefovir* (32.9 mg, 0.01 mmol) and EtOH (2 mL). The resulting mixture is then stirred 5 minutes at 25 °C and then 16 hours at 80 °C (covered with aluminum foil). The reaction is allowed to cool down to 25 °C and the solvent is removed under high vacuum. The crude mixture is then dissolved in CH_3CN (2 mL) and $^{n}Bu_4NCl$ (111.2 mg, 0.4 mmol) is added. The resulting mixture is then stirred 5 minutes at 25 °C and then 16 hours at 80 °C. The reaction is allowed to cool down to 25 °C and directly purified on silica gel (CH_2Cl_2:MeOH, 100:0 to 90:10) to afford the desired product **44** as an orange oil (20 mg, 57 %).

^{1}H NMR (300 MHz, $CDCl_3$) δ 8.71 (s, 1H), 8.27 (s, 1H), 4.49 (t, J = 4.9 Hz, 2H), 4.11–4.02 (m, 4H), 3.96 (t, J = 5.0 Hz, 2H), 3.76 (d, J = 8.1 Hz, 2H), 1.29–1.23 (m, 6H).

^{13}C NMR (75 MHz, $CDCl_3$) δ 151.9, 152.0, 151.1, 146.3, 131.6, 70.9 (d, ^3J(C,F) = 10 Hz), 65.5 (d, ^1J(C,F) = 167 Hz), 62.6 (d, ^2J(C,F) = 7 Hz), 44.1, 16.6 (d, ^3J(C,F) = 6 Hz).

^{31}P NMR (243 MHz, $CDCl_3$) δ 20.21 (s, 1P).

HRMS (ESI) calculated $[M+Na]^+$ 371.064641, measured 371.064630.

Synthesis of methyl 2-chloro-7-isopropyl-5-oxo-5H-chromeno[2,3-b]pyridine-3-carboxylate (45)

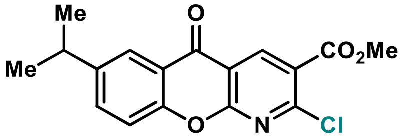

A schlenk under Ar is charged with pyrylium tetrafluoroborate **1** (25.2 mg, 0.15 mmol), **SM4** (31.2 mg, 0.01 mmol) and EtOH (2 mL). The resulting mixture is then stirred 5 minutes at 25 °C and then 16 hours at 80 °C (covered with aluminum foil). The reaction is allowed to cool down to 25 °C and the solvent is removed under high vacuum. The crude mixture is then dissolved in CH_3CN (2 mL) and $^{n}Bu_4NCl$ (111.2 mg, 0.4 mmol) is added. The resulting mixture is then stirred 5 minutes at 25 °C and then 16 hours at 80 °C. The reaction is allowed to cool down to 25 °C and directly purified on silica gel (hexanes:EtOAc, 80:20) to afford the desired product **45** as a white solid (17 mg, 51 %).

^{1}H NMR (300 MHz, $CDCl_3$) δ 9.19 (s, 1H), 8.14 (d, J = 2.3 Hz, 1H), 7.70 (dd, J = 8.7, 2.3 Hz, 1H), 7.55 (d, J = 8.6 Hz, 1H), 4.01 (s, 3H), 3.07 (hept, J = 6.9 Hz, 1H), 1.33 (d, J = 7.0 Hz, 6H).

^{13}C NMR (75 MHz, $CDCl_3$) δ 176.4, 163.6, 160.0, 154.5, 153.9, 146.9, 142.5, 135.4, 123.8, 121.5, 118.7, 115.0, 53.2, 33.9, 24.0.

HRMS (EI) calculated $[M]^+$ 331.060587, measured 331.060640.

Synthesis of (*E*)-3-(pentafluoro-λ6-sulfanyl)allyl 2-(2-chlorothiazol-4-yl)acetate (46)

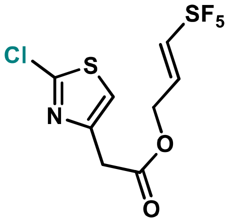

Yellow oil

^{1}H NMR (300 MHz, $CDCl_3$) δ 7.11 (s, 1H), 6.64 (m, 1H), 6.54 (m, 1H), 4.79 (dq, *J* = 4.1, 2.1 Hz, 2H), 3.84 (d, *J* = 0.8 Hz, 2H).

^{13}C NMR (75 MHz, $CDCl_3$) δ 168.8, 152.2, 147.1, 142.1 (t, ^{2}J(C,F) = 21 Hz), 132.3 (m), 118.8, 61.4, 36.9.

^{19}F NMR (282 MHz, $CDCl_3$) δ 82.09 (m, 1F), 63.02 (dd, *J* = 150.8, 2.1 Hz, 4F).

HRMS (ESI) calculated $[M+H]^+$ 343.959969, measured 343.959710.

Synthesis of 1-(4-(2-chlorodibenzo[*b,f*][1,4]oxazepin-11-yl)piperazin-1-yl)-2-(2-chlorothiazol-4-yl)ethan-1-one (47)

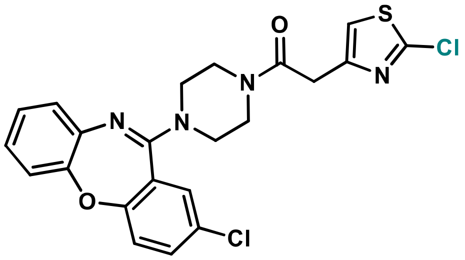

The reaction was performed at 80 °C instead of 120 °C. Yellow sticky oil

^{1}H NMR (300 MHz, $CDCl_3$) δ 7.40 (dd, *J* = 8.6, 2.6 Hz, 1H), 7.31 (d, *J* = 2.5 Hz, 1H), 7.19 (d, *J* = 8.6 Hz, 1H), 7.17-7.06 (m, 4H), 7.01 (m, 1H), 3.86 (s, 2H), 3.82-3.68 (m, 4H), 3.65-3.34 (m, 4H).

^{13}C NMR (75 MHz, $CDCl_3$) δ 167.7, 159.3, 158.5, 151.7, 151.4, 148.7, 139.7, 132.8, 130.4, 128.8, 127.1, 125.8, 125.0, 124.7, 122.8, 120.1, 118.0, 47.4, 47.4, 45.9, 41.6, 36.7.

HRMS (ESI) calculated $[M+H]^+$ 473.060028, measured 473.059970.

Synthesis of 1-((*3S,4R*)-3-((benzo[*d*][1,3]dioxol-5-yloxy)methyl)-4-(4-fluorophenyl)piperidin-1-yl)-2-(2-chlorothiazol-4-yl)ethan-1-one (48)

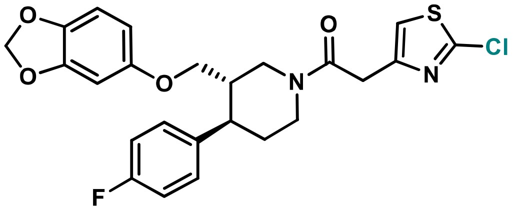

The reaction was performed at 80 °C instead of 120 °C. Yellow sticky oil

^{1}H NMR (600 MHz, DMSO- d_6) δ 7.48 (dd, *J* = 3.4, 0.9 Hz, 1H), 7.27 (ddd, *J* = 10.2, 8.7, 6.2 Hz, 2H), 7.12 (td, *J* = 8.8, 2.1 Hz, 2H), 6.72 (dd, *J* = 8.5, 1.7 Hz, 1H), 6.46 (dd, *J* = 10.1, 2.5 Hz, 1H), 6.17 (dd, *J* = 8.5, 2.5 Hz, 1H), 5.92 (bs, 2H), 4.68 (m, 1H), 4.13 (m, 1H), 3.89 (m, 2H), 3.51 (m, 2H), 3.11 (m, 1H), 2.78 (dtd, *J* = 16.0, 11.7, 3.9 Hz, 1H), 2.62 (m, 1H), 1.98 (dtt, *J* = 11.1, 7.5, 3.5 Hz, 1H), 1.74 (dq, *J* = 13.3, 2.9 Hz, 1H), 1.61 (dqd, *J* = 45.7, 12.6, 4.3 Hz, 1H).

^{13}C NMR (151 MHz, DMSO- d_6) δ 166.9 (d, *J* = 13 Hz), 160.9 (d, ^{1}J(C,F) = 242 Hz), 153.9, 153.8, 149.5 (m), 147.8, 141.2, 139.6, 129.1 (dd, ^{3}J(C,F) = 8 Hz, *J* = 3 Hz), 119.7 (d, *J* = 3 Hz), 115.2 (d, ^{2}J(C,F) = 21 Hz), 107.9 (d, *J* = 7 Hz), 105.6 (d, *J* = 13 Hz), 101.0, 97.8 (d, *J* = 10 Hz), 68.7 (d, *J* = 75 Hz), 47.4 (d, *J* = 414 Hz), 43.9 (d, *J* = 232 Hz), 42.5 (d, *J* = 186 Hz), 41.4 (d, *J* = 106 Hz), 36.0 (d, *J* = 26 Hz), 33.6 (d, *J* = 109 Hz).

^{19}F NMR (282 MHz, DMSO- d_6) δ -116.45 (d, *J* = 3.8 Hz, 1F).

Note: The 2 conformations of piperidine led to a signal splitting in ^{1}H, ^{13}C as well as ^{19}F NMR.

HRMS (ESI) calculated $[M+Na]^+$ 511.086506, measured 511.086620.

Synthesis of (*R*)-2-(5-(3-(1-(4-(2-chloropyrimidin-5-yl)phenyl)-1-cyclopropylethyl)-1,2,4-oxadiazol-5-yl)-1H-pyrazol-1-yl)-N,N-dimethylacetamide (49)

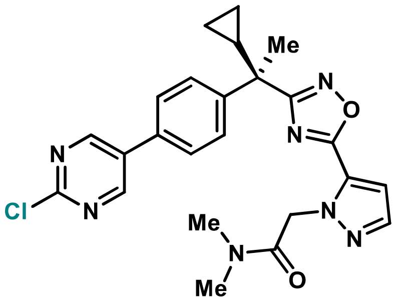

A schlenk under Ar is charged with pyrylium tetrafluoroborate **1** (3.4 mg, 0.02 mmol) and $MgCl_2$ (4.9 mg, 0.05 mmol). *BI665915* (4.7 mg, 0.01 mmol) is then added and directly followed by CH_3CN (1 mL). The resulting mixture is then stirred 5 minutes at 25 °C and then 16 hours at 120 °C. The reaction is allowed to cool down to 25 °C and is concentrated to dryness. The resulting solids were dissolved in the minimum amount of CH_2Cl_2 and purified by preparative TLC (CH_2Cl_2:MeOH, 90:10) to afford the desired product **49** (1.8 mg, 37 %).

^{1}H NMR (600 MHz, DMSO- d_6) δ 8.55 (s, 2H), 8.49 (d, *J* = 0.8 Hz, 1H), 8.06 (d, *J* = 0.7 Hz, 1H), 7.56 (d, *J* = 8.5 Hz, 2H), 7.38 (d, *J* = 8.6 Hz, 2H), 5.21 (s, 2H), 3.03 (s, 3H), 2.85 (s, 3H), 1.66 (m, 1H), 1.48 (s, 3H), 0.64 (m, 1H), 0.49 (m, 1H), 0.43 (m, 1H), 0.34 (m, 1H).

^{13}C NMR (151 MHz, DMSO- d_6) δ 175.6, 172.3, 170.3, 165.8, 155.8, 143.8, 138.5, 134.0, 133.5, 127.3, 125.2, 121.7, 106.6, 53.6, 53.25, 2.0, 1.0.

Note: Due to the amount of material recovered as well as the presence of several peaks in the aliphatic area, 4 carbons cannot be precisely attributed.

HRMS (ESI) calculated $[M+H]^+$ 478.175274, measured 478.174910.

Synthesis of 5-chloro-1-(2,6-dichloro-4-(trifluoromethyl)phenyl)-4-((trifluoromethyl)sulfinyl)-1H-pyrazole-3-carbonitrile (50)

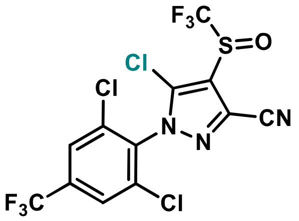

5 equivalents of $MgCl_2$ were used. Yellow solid

^{1}H NMR (300 MHz, $CDCl_3$) δ 7.84 (bs, 2H).

^{13}C NMR (75 MHz, $CDCl_3$) δ 136.1 (d, *J* = 3 Hz)*, 135.9 (q, ^2J(C,F) = 35 Hz), 134.7, 134.2 (bs), 128.0, 126.47 (qd, ^3J(C,F) = 4 Hz, *J* = 1 Hz)*, 125.2 (q, ^1J(C,F) = 336 Hz), 121.9 (q, ^1J(C,F) = 274 Hz), 118.5, 109.3.

**Note: A potential steric hindrance impedes a free rotation between the 2 aromatic rings. Therefore, a signal splitting is detected for these signals.*

^{19}F NMR (282 MHz, $CDCl_3$) δ -63.38 (s, 3F), -72.54 (s, 3F).

HRMS (ESI) calculated $[M+Na]^+$ 477.878058, measured 477.877890.

Synthesis of (*R*)-1-(3-(4-chloro-3-(4-phenoxyphenyl)-1H-pyrazolo[3,4-d]pyrimidin-1-yl)piperidin-1-yl)prop-2-en-1-one (51)

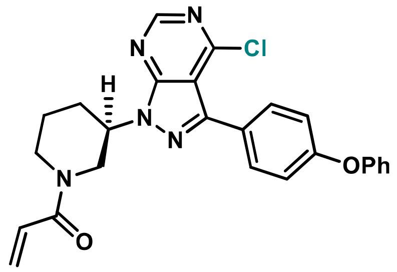

A schlenk under Ar is charged with pyrylium tetrafluoroborate (25.2 mg, 0.15 mmol), *ibrutinib* (44.0 mg, 0.01 mmol) and EtOH (2 mL). The resulting mixture is then stirred 5 minutes at 25 °C and then 16 hours at 50 °C (covered with aluminum foil). The reaction is allowed to cool down to 25 °C and the solvent is removed under high vacuum. The crude mixture is then dissolved in CH_3CN (4 mL) and ^{n}Bu$_4$NCl (111.2 mg, 0.4 mmol) is added. The resulting mixture is then stirred 5 minutes at 25 °C and then 16 hours at 50 °C. The reaction is allowed to cool down to 25 °C and directly purified on silica gel (hexanes:EtOAc, 20:80) to afford the desired product **51** as a yellow oil (40 mg, 87 %).

^{1}H NMR (300 MHz, $CDCl_3$) δ 8.75 (s, 1H), 7.74 (d, *J* = 8.7 Hz, 2H), 7.38 (t, *J* = 7.7 Hz, 2H), 7.20 – 6.98 (m, 5H), 6.58 (dt, *J* = 25.2, 13.6 Hz, 1H), 6.30 (d, *J* = 16.1 Hz, 1H), 5.69 (t, *J* = 14.4 Hz, 1H), 4.98 (bs, 1H), 4.74 (dd, *J* = 80.5, 10.4 Hz, 1H), 4.13 (dd, *J* = 52.9, 13.3 Hz, 1H), 3.61 (dt, *J* = 111.9, 11.9 Hz, 1H), 3.08 (dt, *J* = 92.2, 14.0 Hz, 1H), 2.41 (m, 1H), 2.28 (m, 1H), 2.03 (m, 1H), 1.77 (m, 1H).

^{13}**C NMR** (75 MHz, $CDCl_3$) δ 165.8, 158.8, 156.6, 154.4, 154.1, 144.9, 131.7, 130.0, 128.4, 127.6, 125.9, 125.7, 124.0, 119.7, 118.2, 111.4, 54.3 (0.5 C), 53.2(0.5 C) , 50.1 (0.5 C), 46.1 (0.5 C), 46.0 (0.5 C), 42.3 (0.5 C), 30.3 (0.5 C), 30.1 (0.5 C), 25.3 (0.5 C), 24.0 (0.5 C).

Note: The 2 conformations of piperidine led to a signal splitting in both ^{1}H and ^{13}C NMR.

HRMS (ESI) calculated $[M+H]^+$ 460.153476, measured 460.153380.

Synthesis of 2-chloro-N-(5,6-dimethoxypyrimidin-4-yl)benzo[d]thiazole-6-sulfonamide (52) and 2-chloro-N-(5-methoxy-6-oxo-1,6-dihydropyrimidin-4-yl)benzo[d]thiazole-6-sulfonamide (52’)

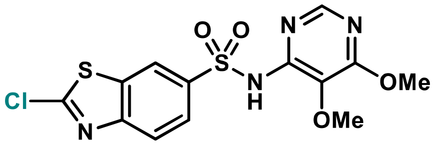

<u>Preparation of **52**</u>

A 18 mL screw-capped tube under normal atmosphere is charged with pyrylium tetrafluoroborate **1** (25.2 mg, 0.15 mmol), **SM5** (36.7 mg, 0.01 mmol) and EtOH (2 mL). The resulting mixture is then stirred 5 minutes at 25 °C and then 16 hours at 80 °C (covered with aluminum foil). The reaction is allowed to cool down to 25 °C and the solvent is removed under high vacuum. The crude mixture is then dissolved in CH_3CN (2 mL) and HCl 1 M in THF (400 µL, 0.4 mmol) is added. The resulting mixture is then stirred 2 days at 25 °C. The crude mixture is partitioned between water and EtOAc. The aqueous layer is extracted with EtOAc (3 × 10 mL). The combined organic layers are dried over Na_2SO_4, concentrated to dryness and purified on silica gel (hexanes:EtOAc, 50:50) to afford the desired product **52’** as a yellow solid (21 mg, 54 %).

^{1}**H NMR** (300 MHz, $CDCl_3$) δ 8.69 (dd, *J* = 1.9, 0.6 Hz, 1H), 8.22 (dd, *J* = 8.7, 1.9 Hz, 1H), 8.14 (s, 1H), 8.04 (dd, *J* = 8.7, 0.6 Hz, 1H), 3.97 (s, 3H), 3.88 (s, 3H).

Note: The signal for –NH is not detected.

^{13}**C NMR** (75 MHz, $CDCl_3$) δ 161.0, 158.0, 154.0, 151.0, 149.5, 137.0, 136.2, 126.7, 126.5, 123.3, 123.0, 60.8, 54.4.

HRMS (ESI) calculated $[M+H]^+$ 386.998303, measured 386.998240.

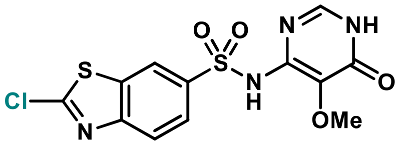

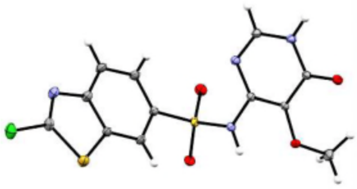

<u>Preparation of **52'**</u>

Prepared according to the general procedure. Note that the reaction was also performed at 80 °C affording the same product in 54% yield.

Yellow solid

^{1}H NMR (300 MHz, DMSO-d_6) δ 12.5 (bs, 1H), 10.91 (bs, 1H), 8.80(d, J = 1.5 Hz, 1H), 8.15–8.06 (m, 2H), 7.75 (s, 1H), 3.69 (s, 3H).

^{13}C NMR (75 MHz, DMSO-d_6) δ 157.9, 157.4, 152.7, 146.3, 143.9, 138.7, 135.8, 130.3, 125.6, 122.8, 122.7, 59.0.

HRMS (ESI) calculated [M-H]$^-$ 370.968103, measured 370.968360.

Synthesis of *N*-(3-(2-(tert-butyl)-5-(2-chloropyrimidin-4-yl)thiazol-4-yl)-2-fluorophenyl)-2,6-difluorobenzenesulfonamide (53)

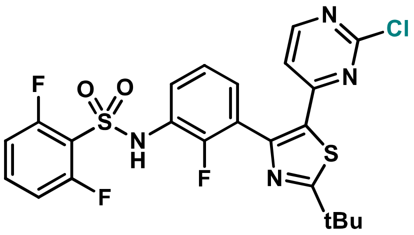

A schlenk under Ar is charged with pyrylium tetrafluoroborate **1** (6.5 mg, 0.04 mmol), $MgCl_2$ (9.2 mg, 0.10 mmol), *dabrafenib* (10.0 mg, 0.02 mmol) and directly followed by CH_3CN (0.5 mL). The resulting mixture is then stirred 5 minutes at 25 °C and then 16 hours at 120 °C. The reaction is allowed to cool down to 25 °C and filtered through celite with CH_2Cl_2. The resulting mixture is concentrated to the minimum and purified by preparative TLC (hexanes:EtOAc, 50:50) to afford the desired product **53** (4.2 mg, 40 %).

^{1}H NMR (300 MHz, $CDCl_3$) δ 8.29 (d, J = 5.4 Hz, 1H), 7.74 (t, J = 7.7 Hz, 1H), 7.50 (m, 1H), 7.34 (t, J = 7.1 Hz, 1H), 7.30–7.18 (m, 2H),* 6.99 (t, J = 8.9 Hz, 2H), 6.74 (d, J = 5.7 Hz, 1H), 1.48 (s, 9H).

**Note: Overlap with $CHCl_3$.*

^{13}C NMR (151 MHz, $CDCl_3$) δ 185.1, 161.5, 161.0, 160.0 (dd, ^{1}J(C,F) = 260, 4 Hz), 159.6, 150.7 (d, ^{1}J(C,F) = 248 Hz), 147.6, 135.5 (t, ^{3}J(C,F) = 11 Hz), 132.1, 128.0 (d, ^{3}J(C,F) = 2.1 Hz), 125.7 (d, ^{3}J(C,F) = 5 Hz), 125.0 (d, ^{2}J(C,F) = 12 Hz), 123.8 (d, ^{2}J(C,F) = 14 Hz), 123.4, 117.1 (t, ^{2}J(C,F) = 15 Hz), 114.9 (b), 113.4 (dd, ^{2}J(C,F) = 23, 4 Hz), 38.4, 30.8.

^{19}F NMR (282 MHz, $CDCl_3$) δ -106.85 (d, J = 3.7 Hz, 2F), -129.95 (bs, 1F).

HRMS (ESI) calculated [M+H]$^+$ 539.058459, measured 539.058050.

V. Limitations

During our investigations, some heterocycles and functional groups were not well tolerated or incompatible with the developed methodology. The main identified limitations are listed below with the associated problem.

Unsuccessful substrates. In this first category, the substrates are too electron rich and therefore the S_NAr step remained limited or was unfeasible. Even though the 2-amino benzimidazole **L1** and 2-aminopyridine (*crizotinib* intermediate) **L2** gave the corresponding products in low isolated yields, most of the rich heterocycles investigated gave the pyridinium salts (**L3-L5**). In the case of the dihydro imidazole *epinastin* **L6**, the starting material was always recovered after hydrolysis.

Decompositions. For **L7**, we could not detect anything in the crude mixture. We assume that the rather weak N–O bond leads to decompositions under the general conditions. Moreover, having two $-NH_2$ moieties appeared to be detrimental to the reaction since no product nor pyridinium intermediate were detected when **L8** was engaged under several conditions. When very activated 2-amino-5-nitropyrimidine was engaged, we first notice the formation of the product **L9** in 30% yield. However, the amount of pyridine detected was about twice higher. Attempts to improve or reproduce this result failed. Thus we postulate a possible decomposition of the formed product during the reaction. Finally, unprotected *mirabegron* **L10** under different conditions failed to give the expected product. If the left part of the molecule was successfully converted for several substrates (see Figure 3 in main text), it is however plausible to explain this setback based on the presence of either or both the secondary amine and the benzylic alcohol.

Fragmentations. Several functionalities are too fragile toward the chlorination step. Indeed, thiobenzoate in *thiamine* derivative **L11** was deprotected resulting in a cyclization with the formamide at room temperature. Even though the corresponding product was detected by mass and crude NMR, the carbamate in **L12** is mostly removed during the reaction yielding **43** as major product. Others fragmentations were also observed for *adenosine* **L13**, *prazosin* **L14** and *celecoxib* derivative **L15**.

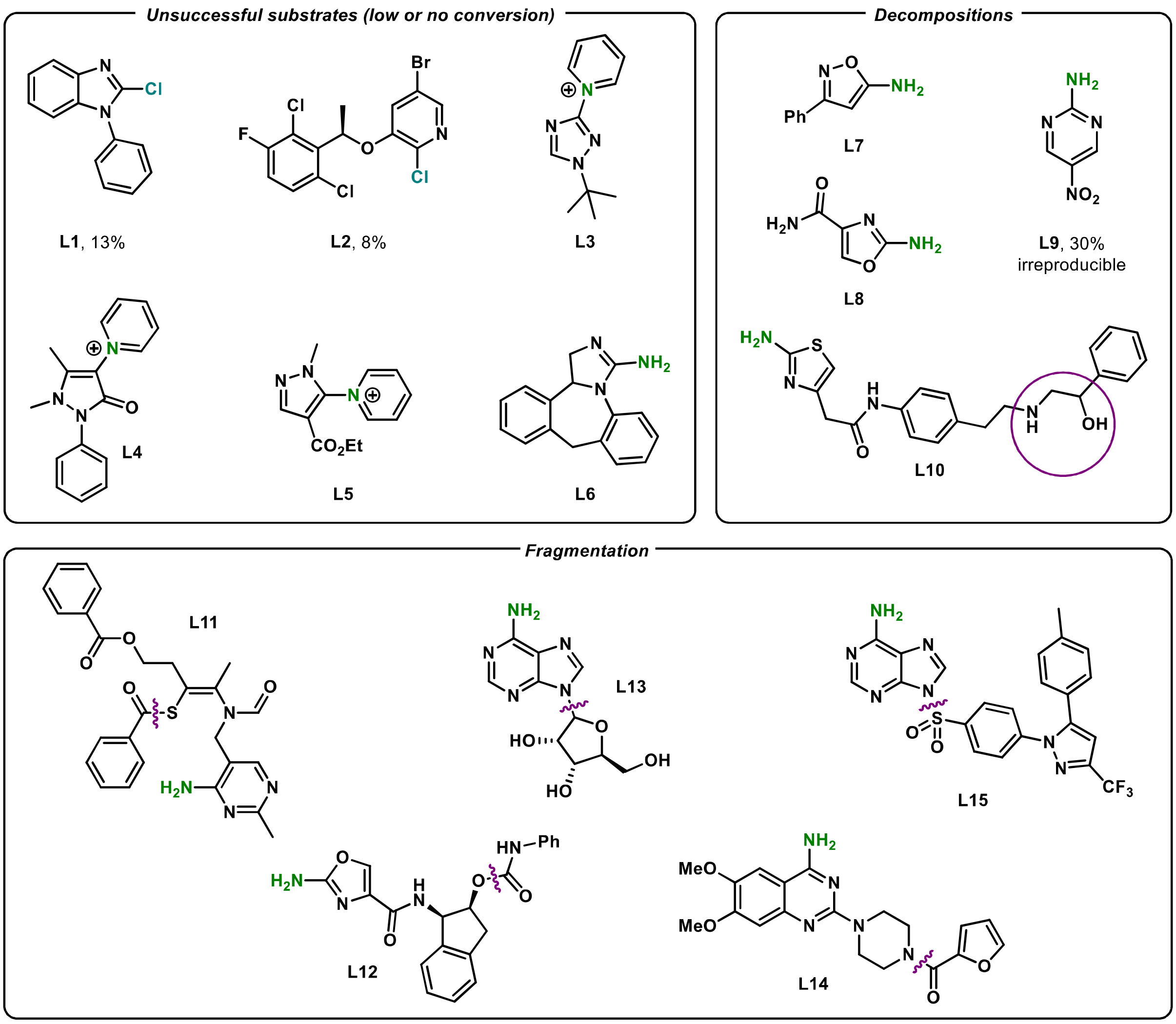

Unsuccessful substrates (low or no conversion)
L1, 13%
L2, 8%
L3
L4
L5
L6
Decompositions
L7
L8
L9, 30%
irreproducible
L10
Fragmentation
L11
L12
L13
L14
L15

VI. Post-functionalization

Negishi coupling of 30

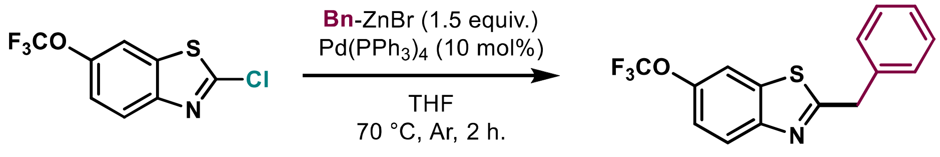

30 **54**, 89%

To a flam-dried schlenk under Ar are added **30** (11 mg, 0.043 mmol), $Pd(PPh_3)_4$ (5 mg, 0.004 mmol) and THF (1 mL). The mixture is stirred at 25 °C is Bn-ZnBr 0.5 M in THF (130 µL, 0.065 mmol) is added by syringe. The reaction is then stirred 2 hours at 70 °C. After cooling down, water is added to the reaction (5 mL) and the aqueous phase is extracted with Et_2O (3 × 5 mL), dried over Na_2SO_4 and purified on silica gel (*n*-pentane:Et_2O, 100:0 to 90:10) to afford **54** as a colorless oil (12 mg, 89 %).

^{1}H NMR (300 MHz, $CDCl_3$) δ 7.99 (d, *J* = 8.9 Hz, 1H), 7.65 (m, 1H), 7.37 – 7.27 (m, 6H), 4.44 (s, 2H).

^{13}C NMR (75 MHz, $CDCl_3$) δ 172.7, 151.9, 146.4 (q, ^3J(C,F) = 2 Hz), 136.9, 136.6, 129.3, 129.1, 127.7, 123.7, 120.7 (q, ^1J(C,F) = 258 Hz), 120.1, 114.3, 40.8.

^{19}F NMR (282 MHz, $CDCl_3$) δ -58.06 (s, 3F).

HRMS (EI) calculated $[M]^+$ 309.042972, measured 309.043220.

Suzuki coupling of 44

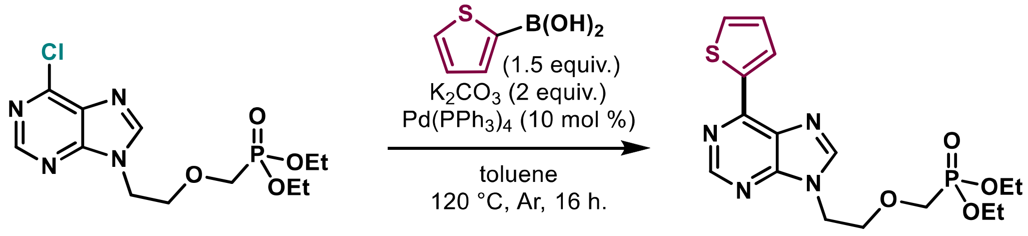

44 **55**, 78%

To a flam-dried schlenk under Ar are added **44** (9 mg, 0.026 mmol), K_2CO_3 (7 mg, 0.052 mmol), $Pd(PPh_3)_4$ (5 mg, 0.004 mmol), 2-thiopheneboronic acid (5 mg, 0.039 mmol) and toluene (1.5 mL). The mixture is stirred at 120 °C for 16 hours. After cooling down, water is added to the reaction (5 mL) and the aqueous phase is extracted with EtOAc (3 × 5 mL), dried over Na_2SO_4 and purified on silica gel (CH_2Cl_2:MeOH, 100:0 to 95:5) to afford **55** as an orange oil (8 mg, 78 %).

^{1}H NMR (600 MHz, $CDCl_3$) δ 8.87 (s, 1H), 8.68 (dd, *J* = 3.8, 1.2 Hz, 1H), 8.24 (s, 1H), 7.61 (dd, *J* = 5.0, 1.2 Hz, 1H), 7.26 (m, 1H), 4.50 (dd, J = 5.4, 4.6 Hz, 2H), 4.11 – 4.06 (m, 4H), 3.98 (dd, J = 5.3, 4.6 Hz, 2H), 3.78 (d, J = 8.2 Hz, 2H), 1.28 (2t *overlap*, J = 7.1, 0.4 Hz, 6H).

^{13}C NMR (151 MHz, $CDCl_3$) δ 152.5, 152.0, 150.2, 145.5, 140.1, 132.8, 130.9, 128.9, 128.9, 71.1 (d, ^3J(C,P) = 10 Hz), 65.5 (d, ^1J(C,P) = 167 Hz), 62.6 (d, ^2J(C,P) = 7 Hz), 43.6, 16.6 (d, ^3J(C,P) = 6 Hz).

^{31}P NMR (243 MHz, $CDCl_3$) δ 20.31 (s, 1P).

HRMS (ESI) calculated $[M+Na]^+$ 419.091336, measured 419.091270.

Sonogashira coupling of 11

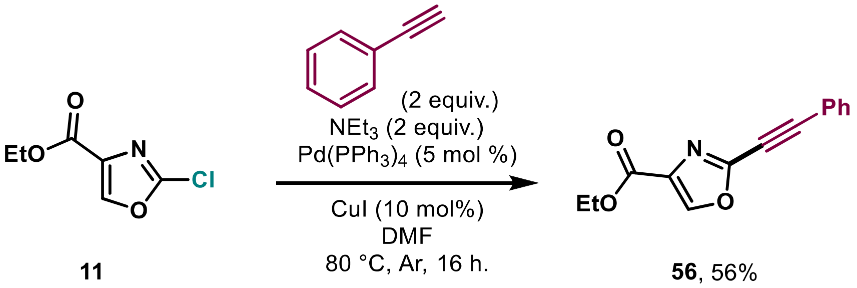

11 **56**, 56%

To a flam-dried schlenk under Ar are added **11** (35.1 mg, 0.2 mmol), $Pd(PPh_3)_4$ (11.6 mg, 0.01 mmol), CuI (3.8 mg, 0.02 mmol), phenylacetylene (44 µL, 0.4 mmol), trimethylamine (56 µL, 0.4 mmol) and DMF (1.5 mL). The mixture is stirred at 80 °C for 16 hours. After cooling down, aqueous solution of saturated $NaHCO_3$ is added to the reaction (5 mL) and the aqueous phase is extracted with EtOAc (3 × 5 mL), dried over Na_2SO_4 and purified on silica gel (*n*-pentane:Et_2O, 70:30) to afford **56** as a brown solid (27 mg, 56 %).

^{1}H NMR (300 MHz, $CDCl_3$) δ 8.24 (s, 1H), 7.60 (m, 2H), 7.49–7.36 (m, 3H), 4.42 (q, *J* = 7.1 Hz, 2H), 1.40 (t, *J* = 7.1 Hz, 3H).

^{13}C NMR (75 MHz, $CDCl_3$) δ 160.7, 147.4, 144.3, 134.7, 132.4, 130.5, 128.8, 120.2, 92.7, 76.3, 61.6, 14.4.

HRMS (EI) calculated $[M]^+$ 241.073344, measured 241.073490.

SNAr of 51 with cyclohexylamine

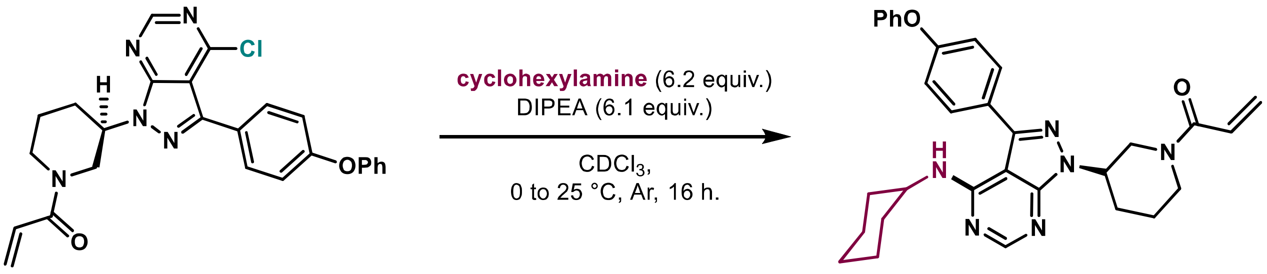

51 **57**, 74%

To a flam-dried schlenk under Ar are added **51** (13 mg, 0.028 mmol) and cyclohexylamine (20 µL, 0.175 mmol) in $CDCl_3$ (1 mL). The mixture is cooled down to 0 °C and DIPEA (30 µL, 0.172 mmol) is slowly added. The mixture is stirred from 0 °C to 25 °C during 16 hours (conversion checked by NMR). The product is then directly purified on silica gel (CH_2Cl_2:MeOH, 100:0 to 95:5) to afford **57** as a white film (11 mg, 74 %).

^{1}H NMR (600 MHz, $CDCl_3$) δ 8.44 (s, 1H), 7.60 (d, *J* = 8.1 Hz, 2H), 7.40 (m, 2H), 7.20–7.16 (m, 3H), 7.08 (m, 2H), 6.58 (m, 1H), 6.28 (t, *J* = 17.8 Hz, 1H), 5.68 (dd, *J* = 38.3, 10.7 Hz, 1H), 5.52 (bs, 1H), 4.86–4.57 (m, 2H), 4.27–4.01 (m, 2H), 3.58 (dt, *J* = 209.9, 11.5 Hz, 1H), 3.04 (m, 1H), 2.35 (m, 1H), 2.25 (m, 1H), 2.01–1.98 (m, 3H), 1.73 (m, 1H), 1.64–1.60 (m, 3H), 1.51–1.45 (m, 2H), 1.23–1.19 (m, 3H).

^{13}C NMR (151 MHz, $CDCl_3$) δ 165.9, 158.8, 156.5, 153.5, 153.2, 144.0, 130.1 (3C), 128.2, 127.8 (2C), 124.3, 119.6, 119.4, 98.5, 53.7 (0.5C), 52.9 (0.5C), 50.2 (0.5C), 46.2 (0.5C), 46.0 (0.5C), 42.3 (0.5C), 32.8, 30.6 (0.5C), 30.2 (0.5C), 29.9, 25.5, 25.4 (0.5C), 24.3, 24.0 (0.5C).

Note: The 2 conformations of piperidine led to a signal splitting in both ^{1}H and ^{13}C NMR.

HRMS (ESI) calculated $[M+H]^+$ 523.281597, measured 523.281660.

SNAr of 31 with *duloxetine*

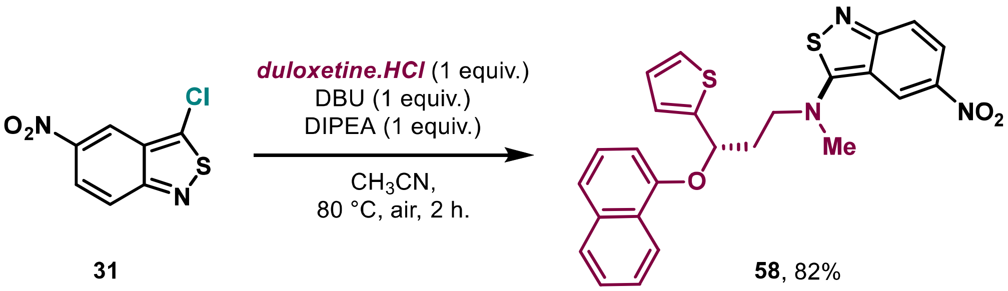

31 **58**, 82%

To a first 18 mL screw-capped tube under normal atmosphere are added *duloxetine.HCl* (24.1 mg, 0.072 mmol) and CH_3CN (0.5 mL). DIPEA (12.6 µL, 0.072 mmol) is then added and the mixture is stirred at 25 °C until it is homogeneous. To a second 18 mL screw-capped tube under normal atmosphere are added **31** (15.5 mg, 0.072 mmol) and CH_3CN (0.5 mL). DBU (10.8 µL, 0.072 mmol) is then added and a red complex is immediately formed. The first flask is finally added to the second one by syringe and the mixture is stirred at 80 °C during 2 hours (conversion checked by TLC). The reaction is then allowed to cooled down to 25 °C and water (5 mL) is added to the mixture. The aqueous phase is then extracted with EtOAc (3 × 10 mL), dried over Na_2SO_4 and purified on silica gel (hexanes:EtOAc, 50:50) to afford **58** as a red foam (28 mg, 82 %).

^{1}H NMR (300 MHz, $CDCl_3$) δ 8.74 (d, *J* = 2.2 Hz, 1H), 8.31 (m, 1H), 7.94 (dd, *J* = 9.8, 2.2 Hz, 1H), 7.76 (m, 1H), 7.51-7.45 (m, 2H), 7.41-7.32 (m, 2H), 7.26-7.21 (m, 2H), 7.08 (d, *J* = 3.5 Hz, 1H), 6.93 (dd, *J* = 5.1, 3.5 Hz, 1H), 6.79 (d, *J* = 7.7 Hz, 1H), 5.79 (dd, *J* = 8.1, 4.4 Hz, 1H), 3.96 (td, *J* = 6.9, 6.3, 1.7 Hz, 2H), 3.39 (s, 3H), 2.76-2.53 (m, 2H).

^{13}C NMR (75 MHz, $CDCl_3$) δ 178.2, 163.0, 152.6, 143.5, 139.9, 134.7, 127.8, 127.1, 126.7, 125.9, 125.7, 125.7, 125.5, 125.2, 122.3, 122.0, 122.0, 121.7, 121.4, 106.9, 73.7, 54.8, 43.2, 35.6.

Note: One C signal cannot be found probably due to an overlap.

HRMS (ESI) calculated $[M+H]^+$ 476.109711, measured 476.109480.

SNAr of 29 with cholesterol

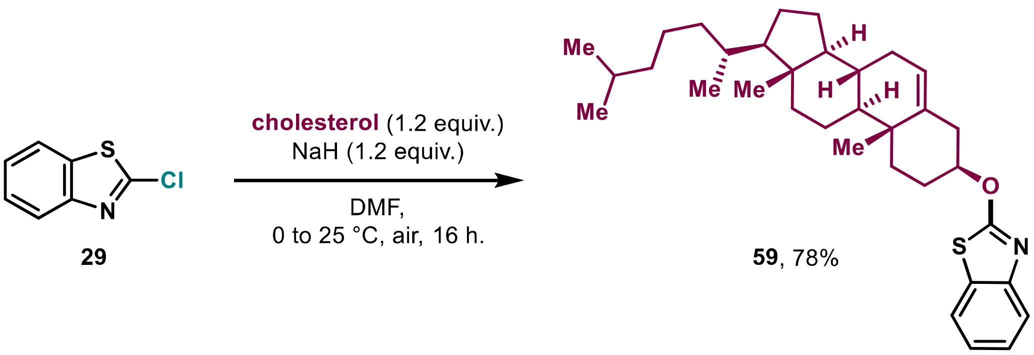

29 **59**, 78%

To a 18 mL screw-capped tube under normal atmosphere are added **29** (33.9 mg, 0.2 mmol), cholesterol 92.5 % (100.3 mg, 0.24 mmol) and DMF (2 mL). The mixture is cooled down to 0 °C and

NaH (5.8 mg, 0.24 mmol) is added. The mixture is stirred 5 minutes at 0 °C and is then allowed to reach 25 °C for 16 hours. The reaction is then carefully quenched with water. The aqueous phase is then extracted with EtOAc (3 × 10 mL), dried over Na_2SO_4 and purified on silica gel (hexanes:EtOAc, 95:5) to afford **59** as a colorless sticky oil (81 mg, 78 %).

^{1}H NMR (300 MHz, $CDCl_3$) δ 7.67-7.61 (m, 2H), 7.34 (m, 1H), 7.20 (td, *J* = 7.7, 1.2 Hz, 1H), 5.45 (dt, *J* = 5.6, 1.9 Hz, 1H), 5.02 (tt, J = 11.4, 4.8 Hz, 1H), 2.67 (ddd, J = 13.0, 5.1, 2.3 Hz, 1H), 2.50 (td, J = 12.7, 12.2, 2.7 Hz, 1H), 2.18 (m, 1H), 2.06-1.89 (m, 3H), 1.88-1.69 (m, 2H), 1.65 – 0.96 (m, 23H)*, 0.93 (d, *J* = 6.5 Hz, 3H), 0.89 (d, *J* = 1.3 Hz, 3H), 0.86 (d, *J* = 1.3 Hz, 3H), 0.70 (s, 3H).

**Note: This integral should represent 23 H but actually represents 26 H due to complex overlaps between the signals as well as residual grease and water.*

^{13}C NMR (75 MHz, $CDCl_3$) δ 172.4, 149.8, 139.6, 131.9, 126.0, 123.4, 123.3, 121.3, 120.8, 82.0, 56.9, 56.3, 50.3, 42.5, 39.9, 39.7, 38.3, 37.1, 36.8, 36.4, 36.0, 32.1, 32.0, 28.4, 28.2, 28.0, 24.5, 24.0, 23.0, 22.7, 21.3, 19.5, 18.9, 12.0.

HRMS (ESI) calculated $[M+Na]^+$ 542.342705, measured 542.342660.

Azidation of 24

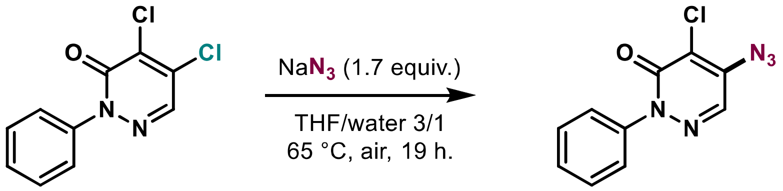

24 **60**, >95%

To a 18 mL screw-capped tube under normal atmosphere are added **24** (12 mg, 0.05 mmol) and NaN_3 (5.5 mg, 0.085 mmol) in THF/water (0.4 mL, 3/1 v/v). The reaction is stirred 19 hours at 65 °C. The mixture is then cooled down to 25 °C and quenched with water (5 mL). The aqueous layer is then extracted with EtOAc (3 × 5 mL) and the combined organic layers are dried over Na_2SO_4 and concentrated to dryness to obtained **60** as a white solid (12 mg, quant.).

^{1}H NMR (300 MHz, $CDCl_3$) δ 7.74 (s, 1H), 7.56 (m, 2H), 7.51 – 7.38 (m, 3H).

HRMS (ESI) calculated $[M+Na]^+$ 270.015306, measured 270.015370.

Characterization data matched with a previously reported example.[14]

Fluorination of 45

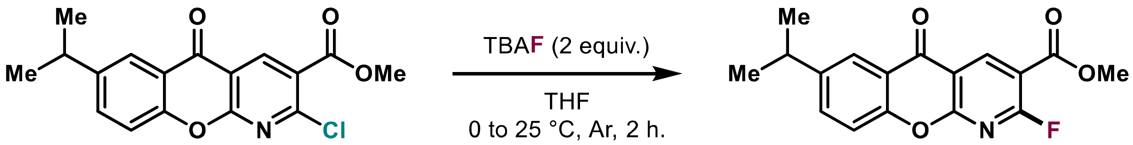

45 **61**, 61%

To a flam-dried schlenk under Ar are added **45** (13 mg, 0.039 mmol) and THF (1 mL). The mixture is cooled down to 0 °C and TBAF 1 M in THF (80 µL, 0.080 mmol) is slowly added. The mixture is stirred 1.5 hours at 0 °C and 30 minutes at 25 °C. The reaction is then quenched with water (1 mL) and

extracted with EtOAc (3 × 5 mL) and the combined organic layers are dried over Na_2SO_4 and purified on silica gel (hexanes:EtOAc, 8:2) to afford **61** as a white solid (7.5 mg, 61 %).

^{1}H NMR (300 MHz, $CDCl_3$) δ 9.38 (d, ^4J(H,F) = 9.0 Hz, 1H), 8.16 (d, J = 2.3 Hz, 1H), 7.70 (dd, J = 8.6, 2.3 Hz, 1H), 7.55 (d, J = 8.7 Hz, 1H), 4.01 (s, 3H), 3.08 (hept, J = 6.9 Hz, 1H), 1.33 (d, J = 6.9 Hz, 7H).

^{13}C NMR (151 MHz, $CDCl_3$) δ 176.0, 162.9 (d, ^1J(C,F) = 263 Hz), 162.3 (d, ^3J(C,F) = 8 Hz), 161.0 (d, ^3J(C,F) = 18 Hz), 153.9, 147.0, 146.0 (d, ^3J(C,F) = 4 Hz), 135.2, 123.9, 121.4, 118.6, 114.9 (d, ^4J(C,F) = 4 Hz), 111.7 (d, ^2J(C,F) = 24 Hz), 53.1, 33.9, 24.0.

^{19}F NMR (282 MHz, $CDCl_3$) δ -51.44 (s, 3F).

HRMS (EI) calculated $[M]^+$ 315.090137, measured 315.090100.

Sequential chlorination/S$_N$Ar with pyrrolidine from 62

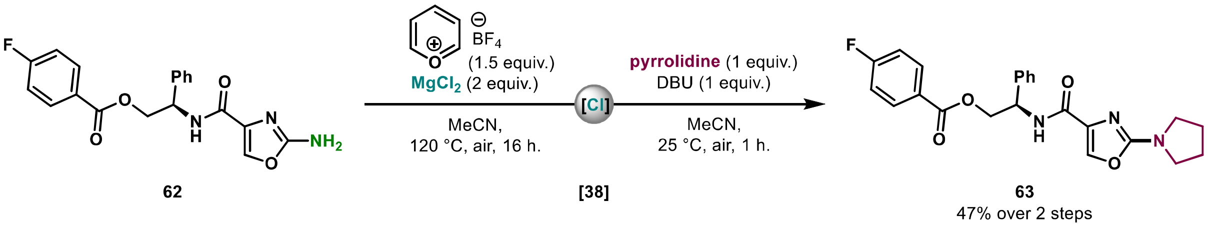

*From **38**:* To a 18 mL screw-capped tube under normal atmosphere are added **38** (19 mg, 0.049 mmol) and CH_3CN (0.5 mL). DBU (8.8 µL, 0.059 mmol) is then added at 25 °C and a red complex is immediately formed. The stirring is maintained for 5 minutes and pyrrolidine (5 µL, 0.06 mmol) is added. The mixture is stirred at 25 °C during 1 hour. Water (5 mL) is added to the mixture. The aqueous phase is then extracted with EtOAc (3 × 10 mL), dried over Na_2SO_4 and purified on silica gel (hexanes:EtOAc, 80:20) to afford **63** as a red oil (17 mg, 82 %).

*From **62**:* A 18 mL screw-capped tube under normal atmosphere is charged with pyrylium tetrafluoroborate **1** (1.5 equiv.) and $MgCl_2$ (19.5 mg, 0.2 mmol). **62** (36.9 mg, 0.1 mmol) is then added and directly followed by CH_3CN (1 mL). The resulting mixture is then stirred 5 minutes at 25 °C and then 16 hours at 120 °C. The reaction is allowed to cool down to 25 °C and is partitioned between water (10 mL) and EtOAc (10 mL). The aqueous layer is extracted with EtOAc (3 × 10 mL). The combined organic layers are dried over Na_2SO_4 and concentrated to dryness yielding a red sticky oil. The crude mixture is redissolved in CH_3CN (1 mL) followed by the addition of DBU (15 µL, 0.1 mmol). The red reaction became darker. The stirring is maintained for 5 minutes at 25 °C and pyrrolidine (8 µL, 0.1 mmol) is added. The mixture is stirred at 25 °C during 1 hour. Water (5 mL) is added to the mixture. The aqueous phase is then extracted with EtOAc (3 × 10 mL), dried over Na_2SO_4 and purified on silica gel (hexanes:EtOAc, 80:20) to afford **63** as a red oil (20 mg, 47 %).

^{1}H NMR (300 MHz, $CDCl_3$) δ 8.01 (m, 2H), 7.72 (s, 1H), 7.49–7.42 (m, 3H), 7.37 (m, 2H), 7.31 (m, 1H), 7.08 (m, 2H), 5.61 (dt, J = 8.7, 6.2 Hz, 1H), 4.70–4.61 (m, 2H), 3.48 (m, 4H), 1.98 (m, 4H).

^{13}C NMR (75 MHz, $CDCl_3$) δ 166.0 (d, ^1J(C,F) = 254 Hz), 165.5, 161.5, 159.9, 138.5, 135.8, 134.9, 132.4 (d, ^3J(C,F) = 9 Hz), 129.0, 128.2, 127.0, 126.2 (d, ^4J(C,F) = 3 Hz), 115.7 (d, ^2J(C,F) = 22 Hz), 67.0, 51.7, 47.5, 25.7.

^{19}F NMR (282 MHz, $CDCl_3$) δ -105.44 (s, 1F).

HRMS (ESI) calculated $[M+H]^+$ 424.166709, measured 424.166450.

VII. Deaminative brominations

Synthesis of 2-bromo-6-(trifluoromethoxy)benzo[d]thiazole (66)

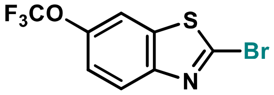

To a 18 mL screw-capped tube under normal atmosphere are added *riluzole* **65** (23.4 mg, 0.1 mmol) and pyrylium tetrafluoroborate **1** (25.2 mg, 0.15 mmol) in EtOH (1 mL). The resulting mixture is then stirred 5 minutes at 25 °C and then 16 hours at 80 °C (covered with aluminum foil). The reaction is allowed to cool down to 25 °C and the solvent is removed under high vacuum. The crude mixture is then dissolved in CD_3CN (1 mL) and LiBr (34.7 mg, 0.4 mmol) is added. The resulting mixture is then stirred 5 minutes at 25 °C and then 16 hours at 80 °C. The reaction is allowed to cool down to 25 °C. The crude mixture is partitioned between water and Et_2O. The aqueous layer is extracted with Et_2O (3 × 10 mL). The combined organic layers are dried over Na_2SO_4, concentrated to dryness and purified on silica gel (*n*-pentane:Et_2O, 100:0 to 80:20) to afford the desired product **66** as an off white solid (28 mg, 94%).

^{1}H NMR (300 MHz, $CDCl_3$) δ 7.99 (d, J = 8.9 Hz, 1H), 7.68 (dt, J = 2.1, 1.1 Hz, 1H), 7.35 (m, 1H).

^{13}C NMR (75 MHz, $CDCl_3$) δ 151.0, 147.0 (q, ^3J(C,F) = 2 Hz), 139.9, 138.3, 123.9, 120.7, 120.6 (q, ^1J(C,F) = 258 Hz), 113.6.

^{19}F NMR (282 MHz, $CDCl_3$) δ -58.05 (s, 3F).

HRMS (EI) calculated $[M]^+$ 296.906547, measured 296.906890.

Synthesis of 6-bromo-9-ethyl-9H-purine (68)

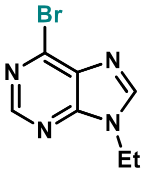

A 18 mL screw-capped tube under normal atmosphere is charged with pyrylium tetrafluoroborate **1** (12.6 mg, 0.075 mmol), **67** (8.2 mg, 0.05 mmol) and EtOH (1 mL). The resulting mixture is then stirred 5 minutes at 25 °C and then 16 hours at 80 °C (covered with aluminum foil). The reaction is allowed to cool down to 25 °C and the solvent is removed under high vacuum. The crude mixture is then dissolved in CH_3CN (1 mL) and $MgBr_2$ (18.4 mg, 0.1 mmol) is added. The resulting mixture is then stirred 5 minutes at 25 °C and then 16 hours at 120 °C. The reaction is allowed to cool down to 25 °C and concentrated to dryness. The crude mixture can be purified on a silica pad and washed several times with CH_2Cl_2 to afford the desired product **68** as a colorless liquid (12 mg, 53 %).

^{1}H NMR (300 MHz, $CDCl_3$) δ 8.71 (s, 1H), 8.16 (s, 1H), 4.36 (q, J = 7.3 Hz, 2H), 1.59 (t, J = 7.3 Hz, 3H).

^{13}C NMR (75 MHz, $CDCl_3$) δ 152.0, 150.6, 144.7, 143.3, 134.4, 39.8, 15.5.

HRMS (ESI) calculated $[M+H]^+$, measured.

VIII. Fluorination of pyridinium salts

General procedure for the fluorination of pyridinium tetrafluoroborate salts

A flam-dried 18 mL screw-capped tube under Ar is charged with dried KF (11.6 mg, 0.2 mmol) and pyridinium salt (0.1 mmol). Freshly distilled CD_3CN (1 mL) is added to the mixture under positive Ar pressure. The resulting mixture is then stirred at 80 °C. Completion of the reaction is monitored by ^1H NMR. Mesitylene (around 1 equiv., amount determined by mass) is added to the reaction. A sample (400 μL) of the reaction is transferred into a NMR tube.

Synthesis of ethyl 2-fluorooxazole-4-carboxylate (69)

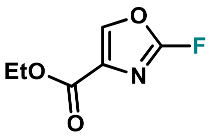

The reaction was performed in 1 h.

^{1}H NMR – in situ (300 MHz, CD_3CN) δ 8.07 (s, 1H), 4.31 (q, *J* = 7.1 Hz, 2H), 1.32 (t, *J* = 7.1 Hz, 3H).

^{19}F NMR – in situ (282 MHz, CD_3CN) δ -97.62 (s, 1F).

HRMS (ESI) calculated $[M+Na]^+$ 182.022391, measured 182.022450.

Synthesis of 2-fluorobenzo[*d*]thiazole (70)

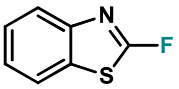

The reaction was performed in 16 h.

^{1}H NMR – in situ (300 MHz, CD_3CN) δ 7.93 (dt, *J* = 7.9, 1.7 Hz, 1H), 7.85 (m, 1H), 7.56 (td, *J* = 7.8, 1.4 Hz, 1H), 7.47 (m, 1H).

^{19}F NMR – in situ (282 MHz, CD_3CN) δ -75.19 (s, 1F).

HRMS (EI) calculated $[M]^+$ 153.004300, measured 153.004340.

Synthesis of 3-chloro-6-fluoropyridazine (71)

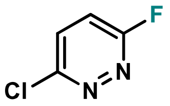

The reaction was performed in 16 h.

^{1}H NMR – in situ (300 MHz, CD_3CN) δ 7.85 (dd, *J* = 9.2, 6.5 Hz, 1H), 7.47 (dd, *J* = 9.2, 1.9 Hz, 1H).

^{19}F NMR – in situ (282 MHz, CD_3CN) δ -84.03 (s, 1F).

HRMS (EI) calculated $[M]^+$ 131.988504, measured 131.988720.

Synthesis of 2-fluoropyrimidine (72)

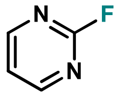

The reaction was performed in 16 h.

^{1}**H NMR – in situ** (300 MHz, CD_3CN) δ 8.72 (dd, *J* = 4.8, 2.0 Hz, 2H), 7.43 (q, *J* = 4.7 Hz, 1H).

^{19}**F NMR – in situ** (282 MHz, CD_3CN) δ -46.95 (s, 1F).

HRMS (EI) calculated $[M]^+$ 98.027476, measured 98.027610.

Synthesis of 9-ethyl-6-fluoro-9*H*-purine (73)

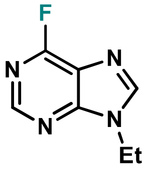

The reaction was performed in 1 h.

^{1}**H NMR – in situ** (300 MHz, CD_3CN) δ 8.62 (s, 1H),* 8.30 (s, 1H), 4.37 (q, *J* = 7.3 Hz, 2H), 1.53 (t, *J* = 7.3 Hz, 3H).

**Note: Overlap with pyridine*

^{19}**F NMR – in situ** (282 MHz, CD_3CN) δ -74.75 (s, 1F).

HRMS (EI) calculated $[M]^+$ 166.064923, measured 166.065090.

IX. Ring-opening at the 2-position with fluorides

Synthesis of ethyl 2-(((1*E*,3*E*)-5-oxopenta-1,3-dien-1-yl)amino)oxazole-4-carboxylate (74)

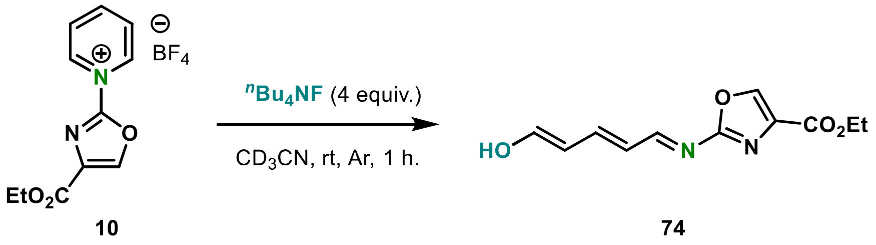

To a 18 mL screw-capped tube under Ar atmosphere are added ethoxycarbonyloxazole pyridinium tetrafluoroborate **10** (10 mg, 0.033 mmol) in CD_3CN (0.5 mL). TBAF 1 M in THF (0.131 mL, 0.131 mmol) is added at 25 °C. The reaction turned immediately deep green. After 1 hour, the reaction is carefully quenched with water (2 mL) and extracted with EtOAc (3 × 5 mL). The combined organic layers are dried over Na_2SO_4 and concentrated to dryness. The redish crude oil is dissolved in the minimum amount of CH_2Cl_2 and purified on preparative TLC (hexanes:EtOAc, 50:50) to afford **74** as a dark red oil (3 mg, 39 %).

^{1}H NMR (500 MHz, CD_3CN) δ 9.44 (d, J = 8.1 Hz, 1H), 8.67 (bs, 1H), 7.99 (s, 1H), 7.35–7.28 (m, 2H), 6.24 (ddd, J = 13.6, 11.4, 0.6 Hz, 1H), 6.01 (ddt, J = 15.0, 8.1, 0.7 Hz, 1H), 4.28 (q, J = 7.1 Hz, 2H), 1.31 (t, J = 7.1 Hz, 3H).*

**Note: This integral should represent 3 H but actually represents 4 H due to the small amount of product as well as the presence of grease.*

^{13}C NMR (126 MHz, CD_3CN) δ 193.9, 162.0, 157.0, 153.7, 140.5, 137.8, 134.0, 128.0, 109.2, 61.7, 14.5.

HRMS (ESI) calculated $[M+Na]^+$ 259.068926, measured 259.068840.

X. Crystallographic data for compounds 43 and 52’

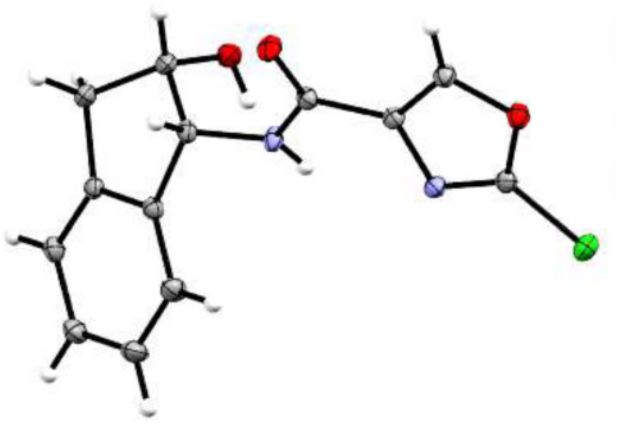

43 CCDC: 2070324

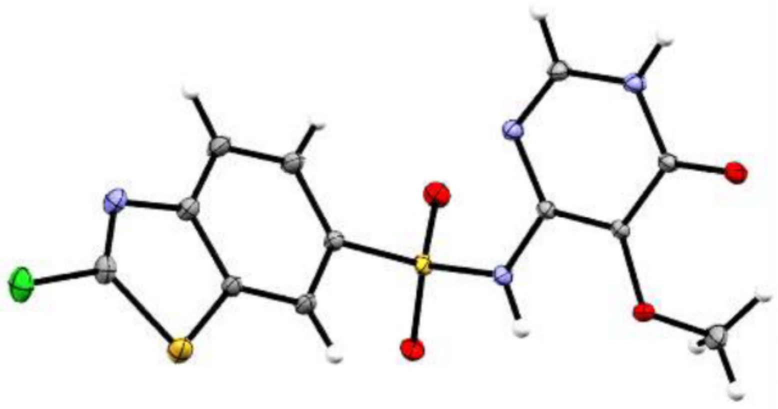

52’ CCDC: 2086010

Crystallographic data for compound 43, CCDC-2070324

Table 1. Crystal data and structure refinement.

Identification code	13594	
Empirical formula	$C_{13} H_{11} Cl N_2 O_3$	
Color	colourless	
Formula weight	278.69 g.mol^{-1}	
Temperature	100(2) K	
Wavelength	0.71073 Å	
Crystal system	ORTHORHOMBIC	
Space group	**$P2_12_12$, (no. 18)**	
Unit cell dimensions	a = 13.361(2) Å	α = 90°.
	b = 18.177(3) Å	β = 90°.
	c = 5.0728(3) Å	γ = 90°.
Volume	1232.0(3) Å^3	
Z	4	
Density (calculated)	1.503 mg.m^{-3}	
Absorption coefficient	0.315 mm^{-1}	
F(000)	576 e	
Crystal size	0.30 x 0.06 x 0.04 mm^3	
θ range for data collection	2.711 to 33.097°.	
Index ranges	$-20 \le h \le 20, -27 \le k \le 27, -7 \le l \le 7$	
Reflections collected	24317	
Independent reflections	4671 [R_{int} = 0.0464]	
Reflections with $I>2\sigma(I)$	4069	
Completeness to θ = 25.242°	99.8 %	
Absorption correction	Gaussian	
Max. and min. transmission	0.99 and 0.95	
Refinement method	Full-matrix least-squares on F^2	
Data / restraints / parameters	4671 / 0 / 188	
Goodness-of-fit on F^2	1.069	
Final R indices $[I>2\sigma(I)]$	R_1 = 0.0364	wR^2 = 0.0772
R indices (all data)	R_1 = 0.0478	wR^2 = 0.0818
Absolute structure parameter	-0.02(3)	
Largest diff. peak and hole	0.3 and -0.4 e · Å^{-3}	

Crystallographic data for compound 43, CCDC-2070324

Table 2. Bond lengths [Å] and angles [°].

Cl(1)-C(4)	1.6882(18)	O(1)-C(1)	1.239(2)
O(2)-C(3)	1.371(2)	O(2)-C(4)	1.352(2)
O(3)-H(3)	0.79(3)	O(3)-C(6)	1.420(2)
N(1)-H(1)	0.84(3)	N(1)-C(1)	1.336(2)
N(1)-C(5)	1.451(2)	N(2)-C(2)	1.402(2)
N(2)-C(4)	1.286(2)	C(1)-C(2)	1.479(2)
C(2)-C(3)	1.350(2)	C(5)-H(5)	0.95(2)
C(5)-C(6)	1.563(2)	C(5)-C(13)	1.517(2)
C(6)-H(6)	0.95(3)	C(6)-C(7)	1.546(2)
C(7)-C(8)	1.509(2)	C(8)-C(9)	1.393(3)
C(8)-C(13)	1.396(2)	C(9)-C(10)	1.394(3)
C(10)-C(11)	1.389(3)	C(11)-C(12)	1.396(3)
C(12)-C(13)	1.382(2)		
C(4)-O(2)-C(3)	103.46(14)	C(6)-O(3)-H(3)	107(2)
C(1)-N(1)-H(1)	117.0(17)	C(1)-N(1)-C(5)	122.24(16)
C(5)-N(1)-H(1)	120.5(17)	C(4)-N(2)-C(2)	102.73(15)
O(1)-C(1)-N(1)	124.37(16)	O(1)-C(1)-C(2)	119.72(16)
N(1)-C(1)-C(2)	115.89(15)	N(2)-C(2)-C(1)	123.75(15)
C(3)-C(2)-N(2)	109.56(15)	C(3)-C(2)-C(1)	126.65(16)
C(2)-C(3)-O(2)	107.93(15)	O(2)-C(4)-Cl(1)	115.76(13)
N(2)-C(4)-Cl(1)	127.89(15)	N(2)-C(4)-O(2)	116.32(16)
N(1)-C(5)-H(5)	109.8(13)	N(1)-C(5)-C(6)	115.44(14)
N(1)-C(5)-C(13)	112.54(15)	C(6)-C(5)-H(5)	109.0(13)
C(13)-C(5)-H(5)	107.0(13)	C(13)-C(5)-C(6)	102.56(14)
O(3)-C(6)-C(5)	112.06(14)	O(3)-C(6)-H(6)	106.0(15)
O(3)-C(6)-C(7)	111.65(16)	C(5)-C(6)-H(6)	112.8(15)
C(7)-C(6)-C(5)	102.85(14)	C(7)-C(6)-H(6)	111.6(15)
C(8)-C(7)-C(6)	103.25(14)	C(9)-C(8)-C(7)	129.96(16)
C(9)-C(8)-C(13)	119.85(16)	C(13)-C(8)-C(7)	110.19(15)
C(8)-C(9)-C(10)	118.51(16)	C(11)-C(10)-C(9)	121.36(17)
C(10)-C(11)-C(12)	120.00(17)	C(13)-C(12)-C(11)	118.71(17)
C(8)-C(13)-C(5)	109.81(15)	C(12)-C(13)-C(5)	128.63(16)
C(12)-C(13)-C(8)	121.56(16)		

Crystallographic data for compound 52', CCDC-2086010

Table 1. Crystal data and structure refinement.

Identification code	13805	
Empirical formula	$C_{12} H_9 Cl N_4 O_4 S_2$	
Color	colorless	
Formula weight	372.80 g·mol^{-1}	
Temperature	100(2) K	
Wavelength	0.71073 Å	
Crystal system	Triclinic	
Space group	*P*-1, (No. 2)	
Unit cell dimensions	a = 7.2216(8) Å	α= 103.284(4)°.
	b = 8.4524(9) Å	β= 90.410(4)°.
	c = 12.6676(14) Å	γ= 103.972(4)°.
Volume	728.67(14) Å^3	
Z	2	
Density (calculated)	1.699 Mg·m^{-3}	
Absorption coefficient	0.575 mm^{-1}	
F(000)	380 e	
Crystal size	0.085 x 0.043 x 0.041 mm^3	
θ range for data collection	1.655 to 35.728°.	
Index ranges	$-11 \le h \le 11$, $-13 \le k \le 13$, $-20 \le l \le 20$	
Reflections collected	30052	
Independent reflections	6677 [R_{int} = 0.0246]	
Reflections with I>2σ(I)	5560	
Completeness to θ = 25.242°	100.0 %	
Absorption correction	Gaussian	
Max. and min. transmission	0.98464 and 0.96485	
Refinement method	Full-matrix least-squares on F^2	
Data / restraints / parameters	6677 / 0 / 247	
Goodness-of-fit on F^2	1.046	
Final R indices [I>2σ(I)]	R_1 = 0.0270	wR^2 = 0.0572
R indices (all data)	R_1 = 0.0388	wR^2 = 0.0610
Extinction coefficient	n/a	
Largest diff. peak and hole	0.423 and -0.369 e·Å^{-3}	

Crystallographic data for compound 52', CCDC-2086010

Table 2. Bond lengths [Å] and angles [°].

C(1)-N(1)	1.2828(12)	C(1)-Cl(1)	1.7108(9)
C(1)-S(1)	1.7388(9)	C(2)-N(1)	1.3888(11)
C(2)-C(7)	1.3950(12)	C(2)-C(3)	1.4046(11)
C(3)-C(4)	1.3935(11)	C(3)-S(1)	1.7324(8)
C(4)-C(5)	1.3905(11)	C(4)-H(4A)	0.919(12)
C(5)-C(6)	1.3969(11)	C(5)-S(2)	1.7591(8)
C(6)-C(7)	1.3896(12)	C(6)-H(6)	0.965(13)
C(7)-H(7)	0.996(14)	C(8)-N(3)	1.3619(10)
C(8)-C(9)	1.3769(10)	C(8)-N(2)	1.3858(10)
C(9)-O(3)	1.3601(9)	C(9)-C(10)	1.4253(10)
C(10)-O(4)	1.2475(9)	C(10)-N(4)	1.3774(10)
C(11)-N(3)	1.3042(10)	C(11)-N(4)	1.3452(10)
C(11)-H(11)	0.986(12)	C(12)-O(3)	1.4333(10)
C(12)-H(12A)	1.023(13)	C(12)-H(12B)	1.019(14)
C(12)-H(12C)	0.990(16)	N(2)-S(2)	1.6356(7)
N(2)-H(2)	0.888(13)	N(4)-H(4)	0.943(14)
O(1)-S(2)	1.4397(6)	O(2)-S(2)	1.4273(7)
N(1)-C(1)-Cl(1)	122.73(7)	N(1)-C(1)-S(1)	118.49(6)
Cl(1)-C(1)-S(1)	118.77(5)	N(1)-C(2)-C(7)	124.74(7)
N(1)-C(2)-C(3)	115.24(7)	C(7)-C(2)-C(3)	120.02(7)
C(4)-C(3)-C(2)	121.97(7)	C(4)-C(3)-S(1)	128.35(6)
C(2)-C(3)-S(1)	109.67(6)	C(5)-C(4)-C(3)	116.67(7)
C(5)-C(4)-H(4A)	121.9(7)	C(3)-C(4)-H(4A)	121.4(7)
C(4)-C(5)-C(6)	122.42(7)	C(4)-C(5)-S(2)	117.28(6)
C(6)-C(5)-S(2)	120.26(6)	C(7)-C(6)-C(5)	120.16(8)
C(7)-C(6)-H(6)	121.6(8)	C(5)-C(6)-H(6)	118.3(8)
C(6)-C(7)-C(2)	118.73(8)	C(6)-C(7)-H(7)	119.8(8)
C(2)-C(7)-H(7)	121.4(8)	N(3)-C(8)-C(9)	123.94(7)
N(3)-C(8)-N(2)	118.00(7)	C(9)-C(8)-N(2)	118.05(7)
O(3)-C(9)-C(8)	120.28(7)	O(3)-C(9)-C(10)	120.40(6)
C(8)-C(9)-C(10)	119.21(7)	O(4)-C(10)-N(4)	120.91(7)
O(4)-C(10)-C(9)	125.12(7)	N(4)-C(10)-C(9)	113.97(6)
N(3)-C(11)-N(4)	125.14(8)	N(3)-C(11)-H(11)	119.2(7)

Crystallographic data for compound 52', CCDC-2086010

N(4)-C(11)-H(11)	115.6(7)	O(3)-C(12)-H(12A)	112.5(7)
O(3)-C(12)-H(12B)	108.7(8)	H(12A)-C(12)-H(12B)	109.0(10)
O(3)-C(12)-H(12C)	105.7(9)	H(12A)-C(12)-H(12C)	109.5(11)
H(12B)-C(12)-H(12C)	111.4(11)	C(1)-N(1)-C(2)	108.91(7)
C(8)-N(2)-S(2)	126.25(6)	C(8)-N(2)-H(2)	118.3(8)
S(2)-N(2)-H(2)	115.4(8)	C(11)-N(3)-C(8)	115.01(7)
C(11)-N(4)-C(10)	122.35(7)	C(11)-N(4)-H(4)	120.5(8)
C(10)-N(4)-H(4)	117.0(8)	C(9)-O(3)-C(12)	114.22(7)
C(3)-S(1)-C(1)	87.67(4)	O(2)-S(2)-O(1)	119.89(4)
O(2)-S(2)-N(2)	110.65(4)	O(1)-S(2)-N(2)	103.36(4)
O(2)-S(2)-C(5)	107.86(4)	O(1)-S(2)-C(5)	108.09(4)
N(2)-S(2)-C(5)	106.19(4)		

XI. References

1 Moser, D. *et al.* Selective functionalization of aminoheterocycles by a pyrylium salt. *Angew. Chem. Int. Ed.* **57**, 11035-11039 (2018).

2 Karimov, R. R., Sharma, A.and Hartwig, J. F. Late Stage Azidation of Complex Molecules. *ACS Cent. Sci.* **2**, 715-724 (2016).

3 Loredo-Calderón, E. L. *et al.* Synthesis of novel α-aminophosphonates under microwave irradiation, biological evaluation as antiproliferative agents and apoptosis inducers. *Med. Chem. Res.* **28**, 2067-2078 (2019).

4 Sharif, S. *et al.* (2Z)-Methyl 2-(2-amino-1,3-thia-zol-4-yl)-2-(methoxy-imino)ethano-ate. *Acta Crystallogr. E* **65**, o1455-o1455 (2009).

5 Gelin, M. *et al.* From Substrate to Fragments to Inhibitor Active In Vivo against Staphylococcus aureus. *ACS Infect. Dis.* **6**, 422-435 (2020).

6 Michels, T. D., Rhee, J. U. and Vanderwal, C. D. Synthesis of δ-Tributylstannyl-α,β,γ,δ-Unsaturated Aldehydes from Pyridines. *Org. Lett.* **10**, 4787-4790 (2008).

7 Gurinov, A. A., Lesnichin, S. B., Limbach, H.-H. and Shenderovich, I. G. How short is the strongest hydrogen bond in the proton-bound homodimers of pyridine derivatives? *J. Phys. Chem. A* **118**, 10804-10812 (2014).

8 Alekseev, R. S., Kurkin, A. V. and Yurovskaya, M. A. Use of the Graebe-Ullmann reaction in the synthesis of 8-methyl-γ-carboline and isomeric aromatic aza-γ-carbolines. *Chem.Heterocycl. Compd.* **48**, 1235-1250 (2012).

9 Ishida, Y. *et al.* Sequence selective dual-emission detection of (i, i + 1) bis-phosphorylated peptide using diazastilbene-type Zn(ii)-Dpa chemosensor. *Chem. Commun.*, 2848-2850 (2009).

10 Andersson, H., Almqvist, F. and Olsson, R. Synthesis of 2-Substituted Pyridines via a Regiospecific Alkylation, Alkynylation, and Arylation of Pyridine N-Oxides. *Org. Lett.* **9**, 1335-1337 (2007).

11 Reeves, E. K., Humke, J. N. and Neufeldt, S. R. N-Heterocyclic Carbene Ligand-Controlled Chemodivergent Suzuki–Miyaura Cross Coupling. *J. Org. Chem.* **84**, 11799-11812 (2019).

12 Sakai, H. A., Liu, W., Le, C. C. and MacMillan, D. W. C. Cross-Electrophile Coupling of Unactivated Alkyl Chlorides. *J. Am. Chem. Soc.* **142**, 11691-11697, (2020).

13 Wang, J. *et al.* Efficient Phosphorus-Free Chlorination of Hydroxy Aza-Arenes and Their Application in One-Pot Pharmaceutical Synthesis. *Org. Progress Res. Dev.* **24**, 146-153 (2020).

14 Brooke, D. G. *et al.* Targeting the Warburg Effect in cancer; relationships for 2-arylpyridazinones as inhibitors of the key glycolytic enzyme 6-phosphofructo-2-kinase/2,6-bisphosphatase 3 (PFKFB3). *Biorg. Med. Chem.* **22**, 1029-1039 (2014).

^{1}H NMR

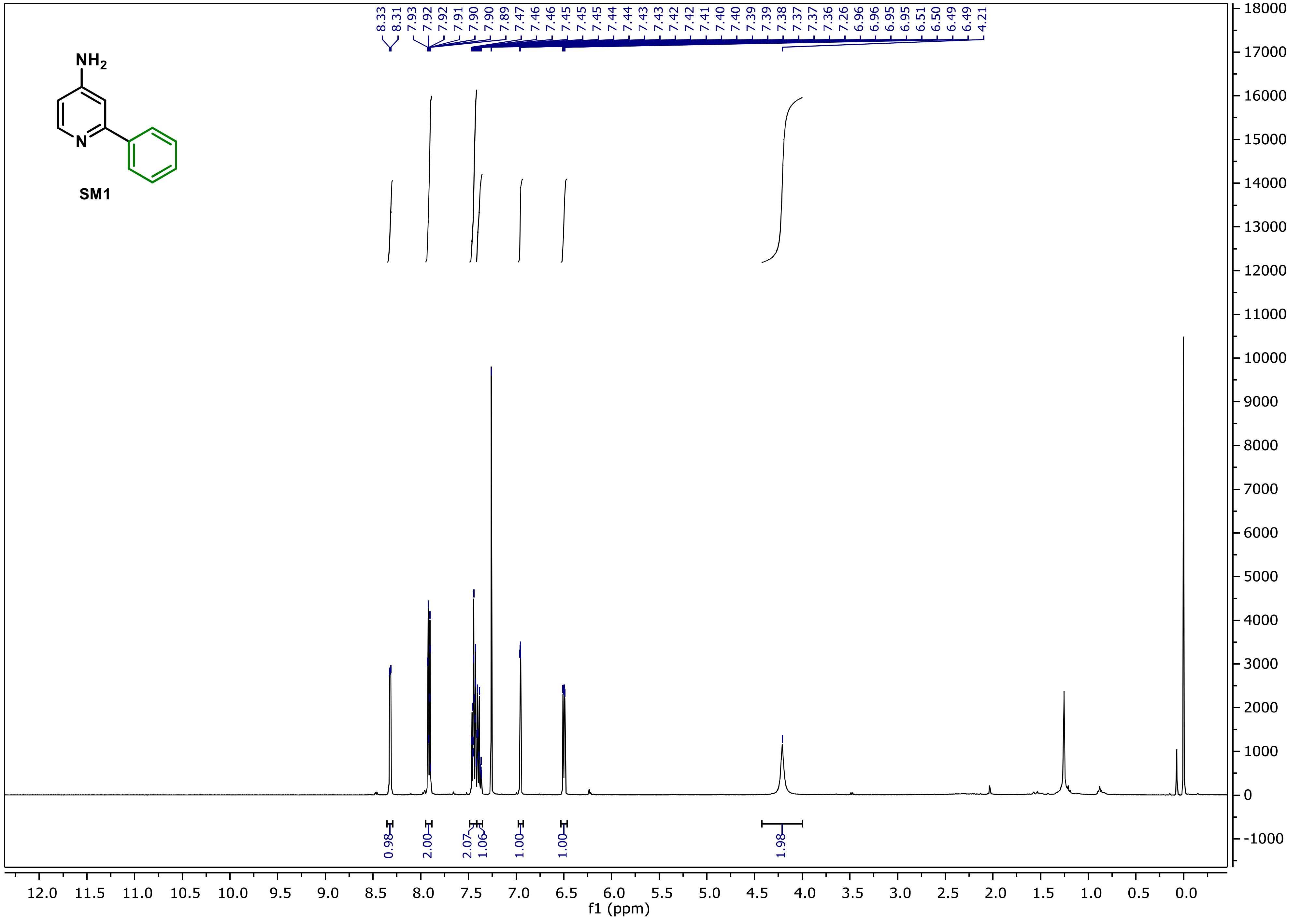

NH2
N
SM1
8.33
8.31
7.93
7.92
7.92
7.91
7.90
7.90
7.89
7.47
7.46
7.46
7.45
7.45
7.45
7.44
7.44
7.43
7.43
7.42
7.42
7.41
7.40
7.40
7.39
7.39
7.38
7.37
7.37
7.36
7.26
6.96
6.96
6.95
6.95
6.51
6.50
6.49
6.49
4.21
0.98
2.00
2.07
1.06
1.00
1.00
1.98
18000
17000
16000
15000
14000
13000
12000
11000
10000
9000
8000
7000
6000
5000
4000
3000
2000
1000
0
-1000
12.0
11.5
11.0
10.5
10.0
9.5
9.0
8.5
8.0
7.5
7.0
6.5
6.0
5.5
5.0
4.5
4.0
3.5
3.0
2.5
2.0
1.5
1.0
0.5
0.0
f1 (ppm)

^1H NMR

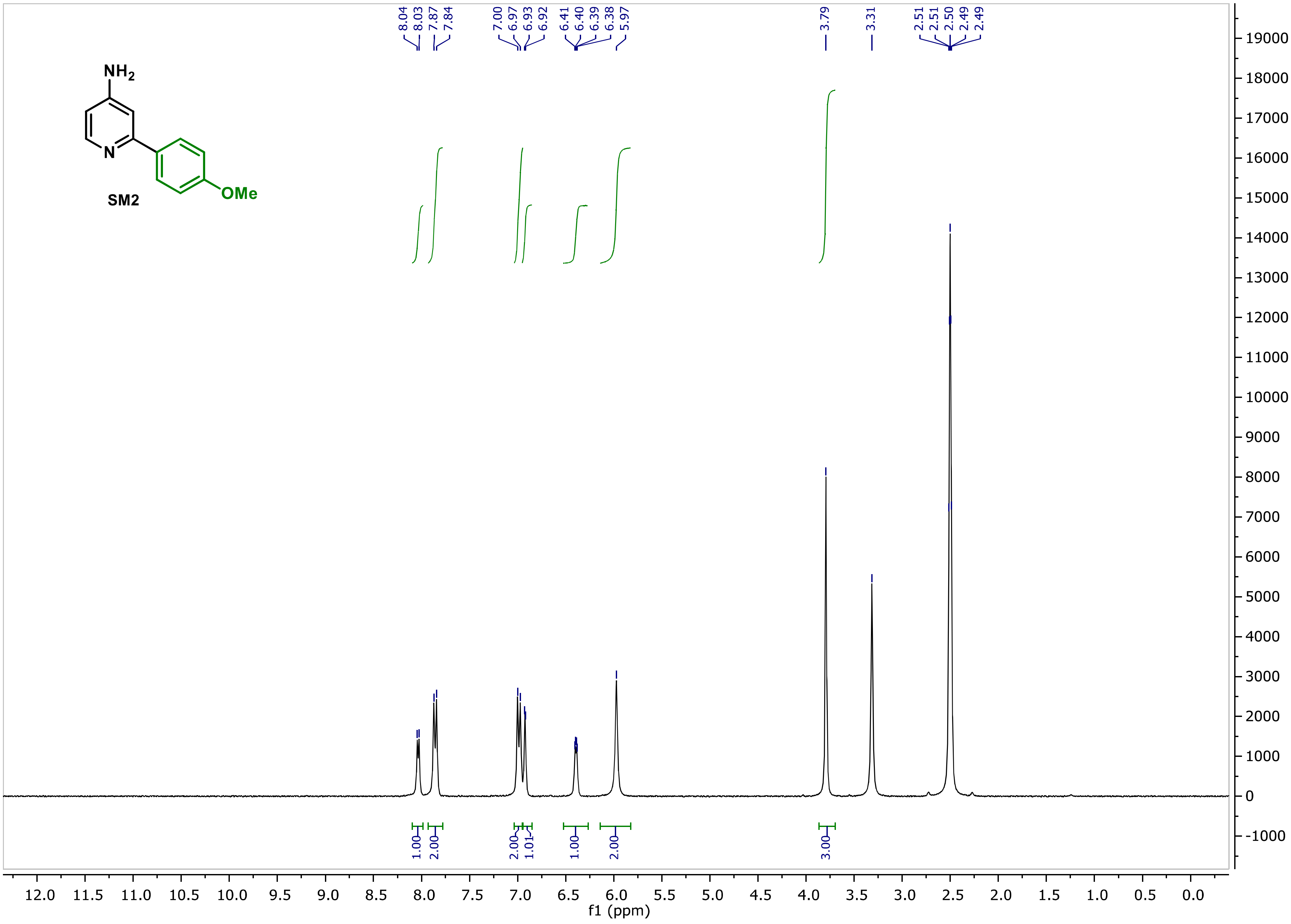

8.04
8.03
7.87
7.84
7.00
6.97
6.93
6.92
6.41
6.40
6.39
6.38
5.97
3.79
3.31
2.51
2.51
2.50
2.49
2.49
NH2
N
OMe
SM2
1.00
2.00
2.00
1.01
1.00
2.00
3.00
f1 (ppm)

^{1}H NMR

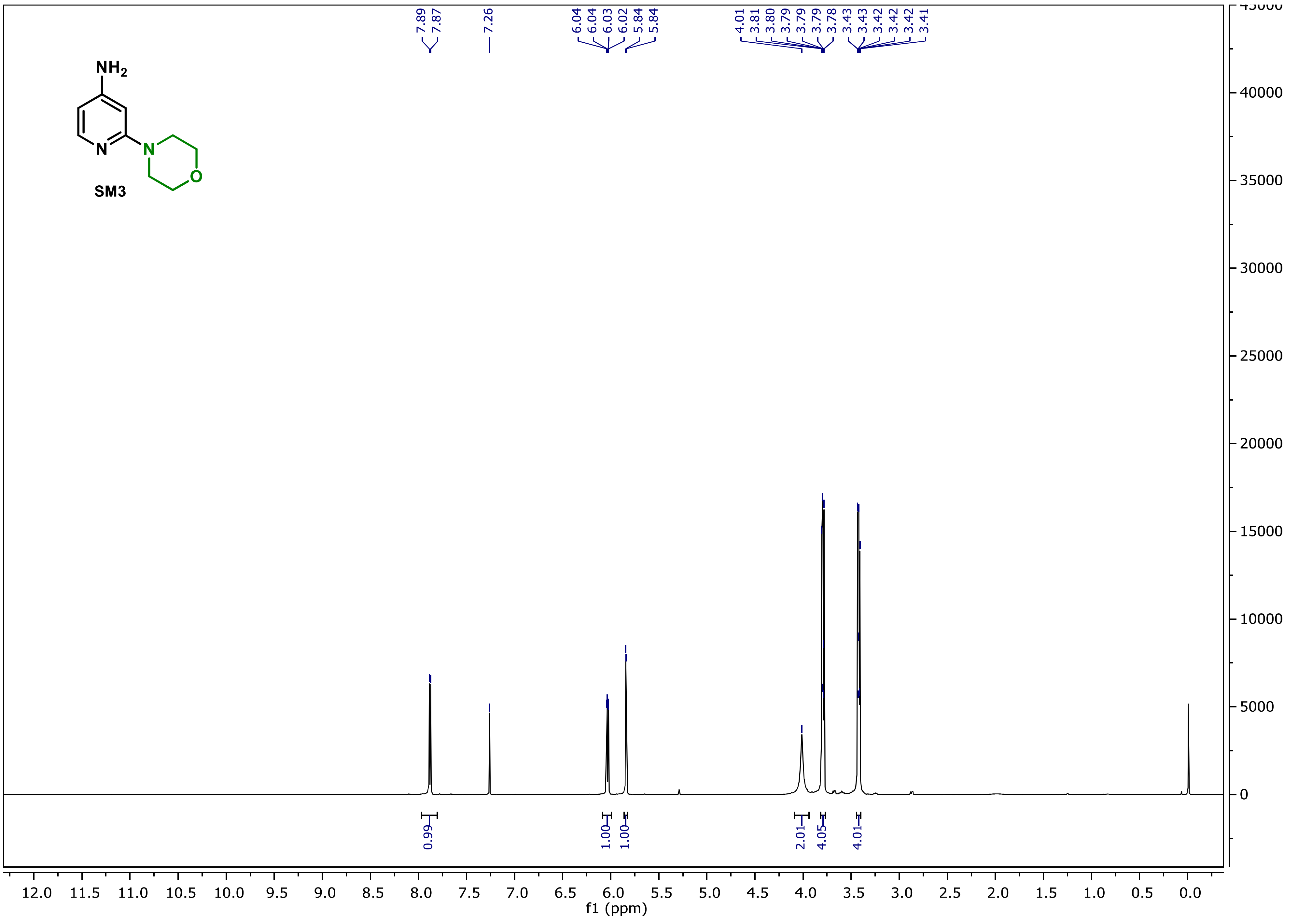

7.89
7.87
7.26
6.04
6.04
6.03
6.02
5.84
5.84
4.01
3.81
3.80
3.79
3.79
3.79
3.78
3.43
3.43
3.42
3.42
3.42
3.41
NH2
N
N
O
SM3
0.99
1.00
1.00
2.01
4.05
4.01
f1 (ppm)

^{1}H NMR

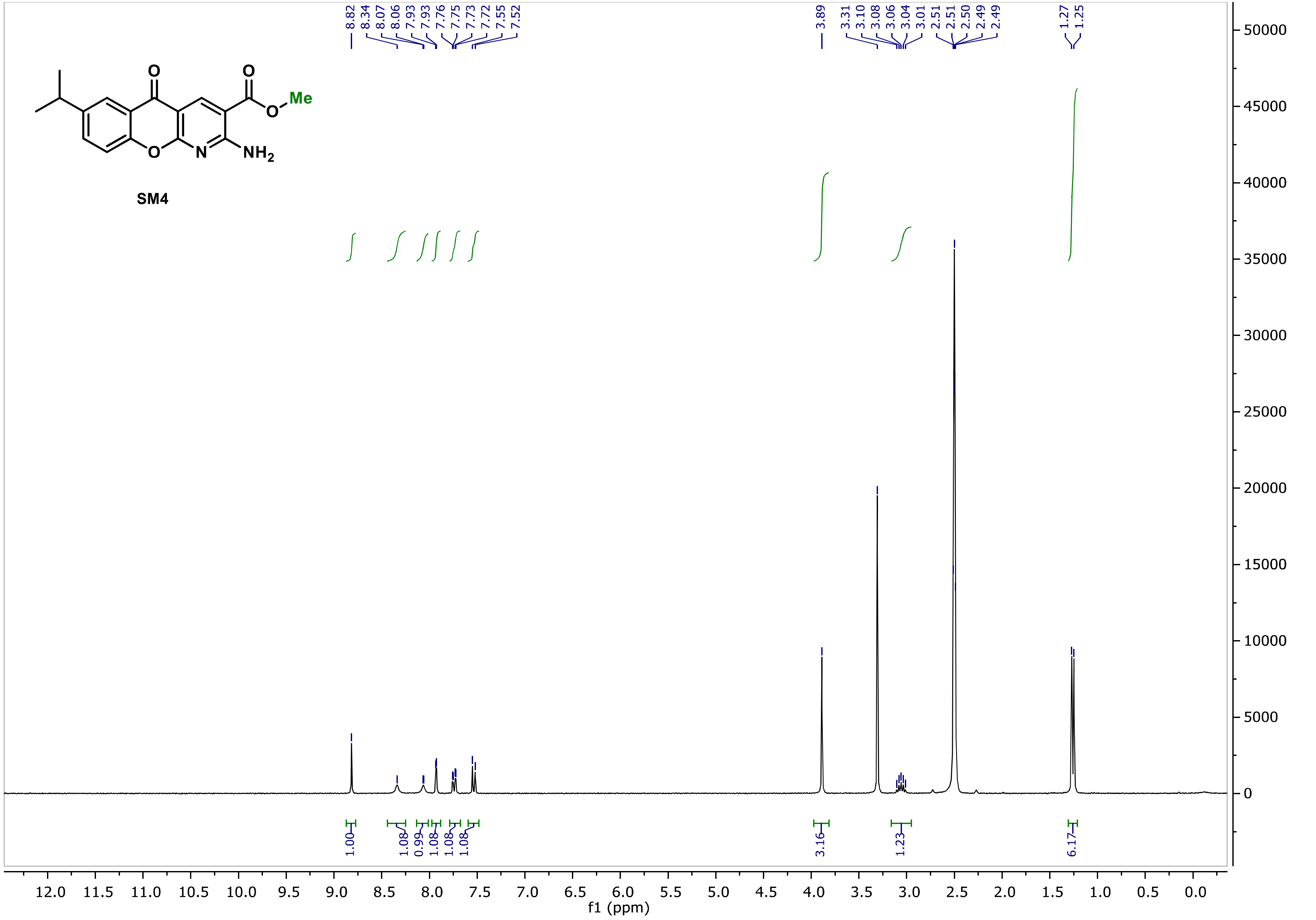
SM4
Me
NH2
8.82
8.34
8.07
8.06
7.93
7.93
7.76
7.75
7.73
7.72
7.55
7.52
3.89
3.31
3.10
3.08
3.06
3.04
3.01
2.51
2.51
2.50
2.49
2.49
1.27
1.25
1.00
1.08
0.99
1.08
1.08
1.08
3.16
1.23
6.17
f1 (ppm)

^1H NMR

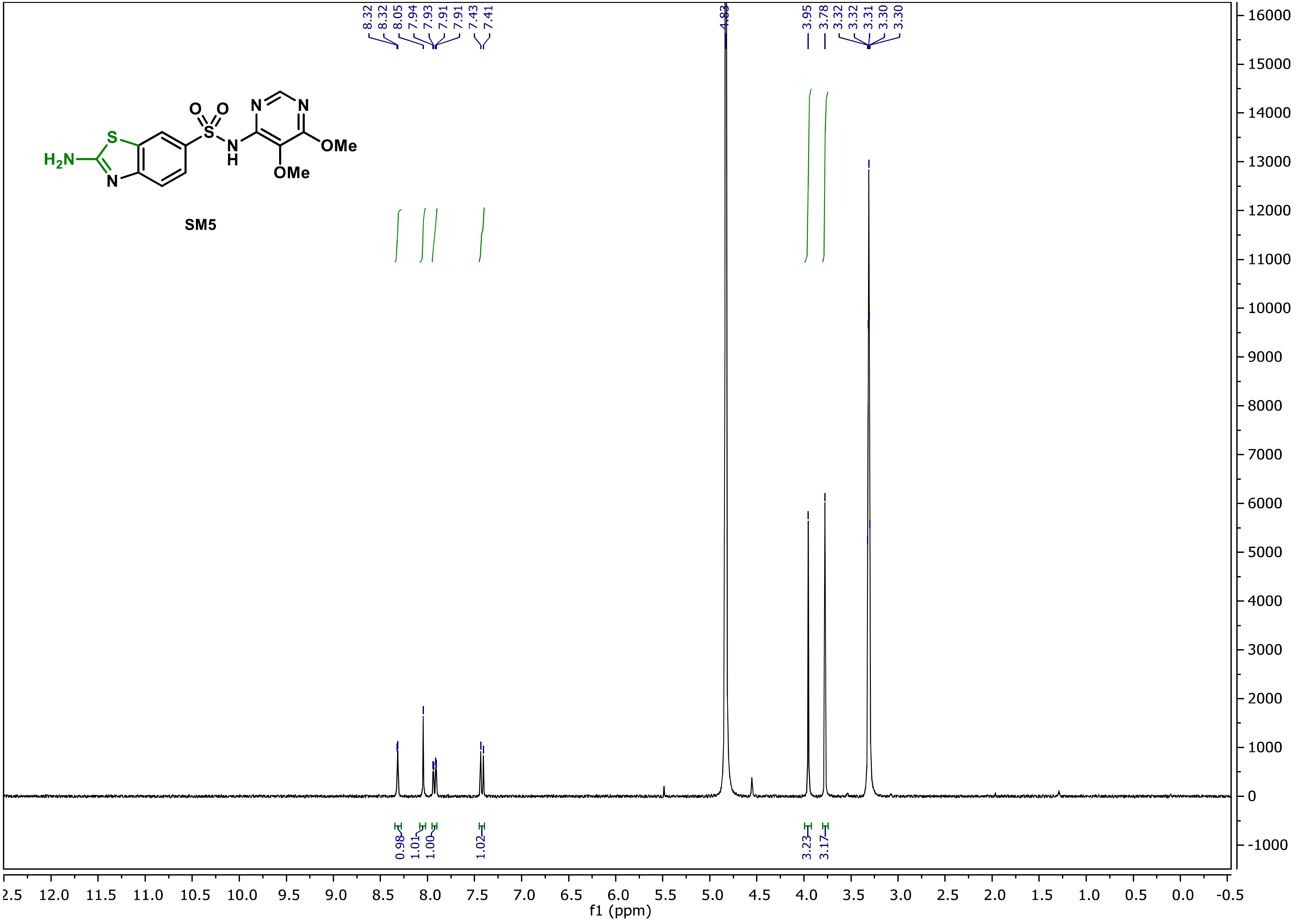
8.32
8.32
8.05
7.94
7.93
7.91
7.91
7.43
7.41
4.83
3.95
3.78
3.32
3.32
3.31
3.30
3.30
0.98
1.01
1.00
1.02
3.23
3.17
H2N
S
N
O
O
N
H
N
N
OMe
OMe
f1 (ppm)

SM5

^{13}C NMR

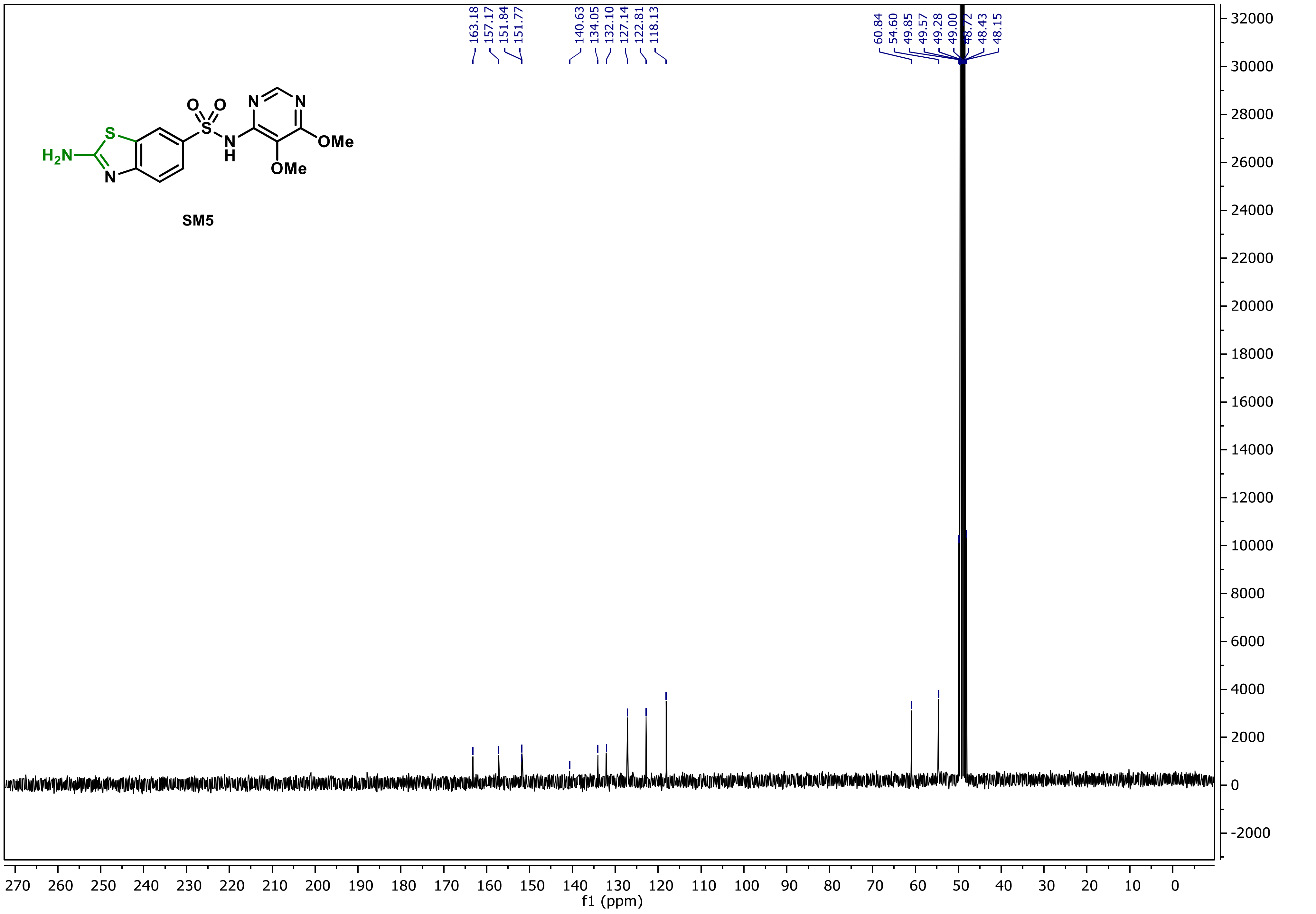
163.18
157.17
151.84
151.77
140.63
134.05
132.10
127.14
122.81
118.13
60.84
54.60
49.85
49.57
49.28
49.00
48.72
48.43
48.15
H2N
S
N
O
O
N
H
N
N
OMe
OMe
SM5
f1 (ppm)

Mass to be matched (m/z): 368.048120 Charge: 1

Mass Tolerance: ±0.005000
Restriction of atom numbers:

C	H	N	O	S
1-100	1-100	1-5	max 5	2-2

Number of calculated Formulas: 4

Formula	Diff.(ppm)	theor. m/z
C13 H14 N5 O4 S2	0.15	368.048174
C15 H16 N2 O5 S2	3.80	368.049518
C18 H14 N3 O2 S2	11.08	368.052196
C22 H12 N2 S2	-12.17	368.043642

Datum	29.03.2021
Analyse:	152529c-00
Sigel:	GHC-GA-654-01
COP: Dr. Clement Ghiazza	
Messung:	HRMS
Methode:	ESIpos
Lösungsmittel:	CH3OH
Spektrometer:	Exactive
Auswerter:	Kampen (2242)

Suggestion:
C13H13N5O4S2 MW 367

characteristical ion
368 = [367 + H]+

^{1}H NMR

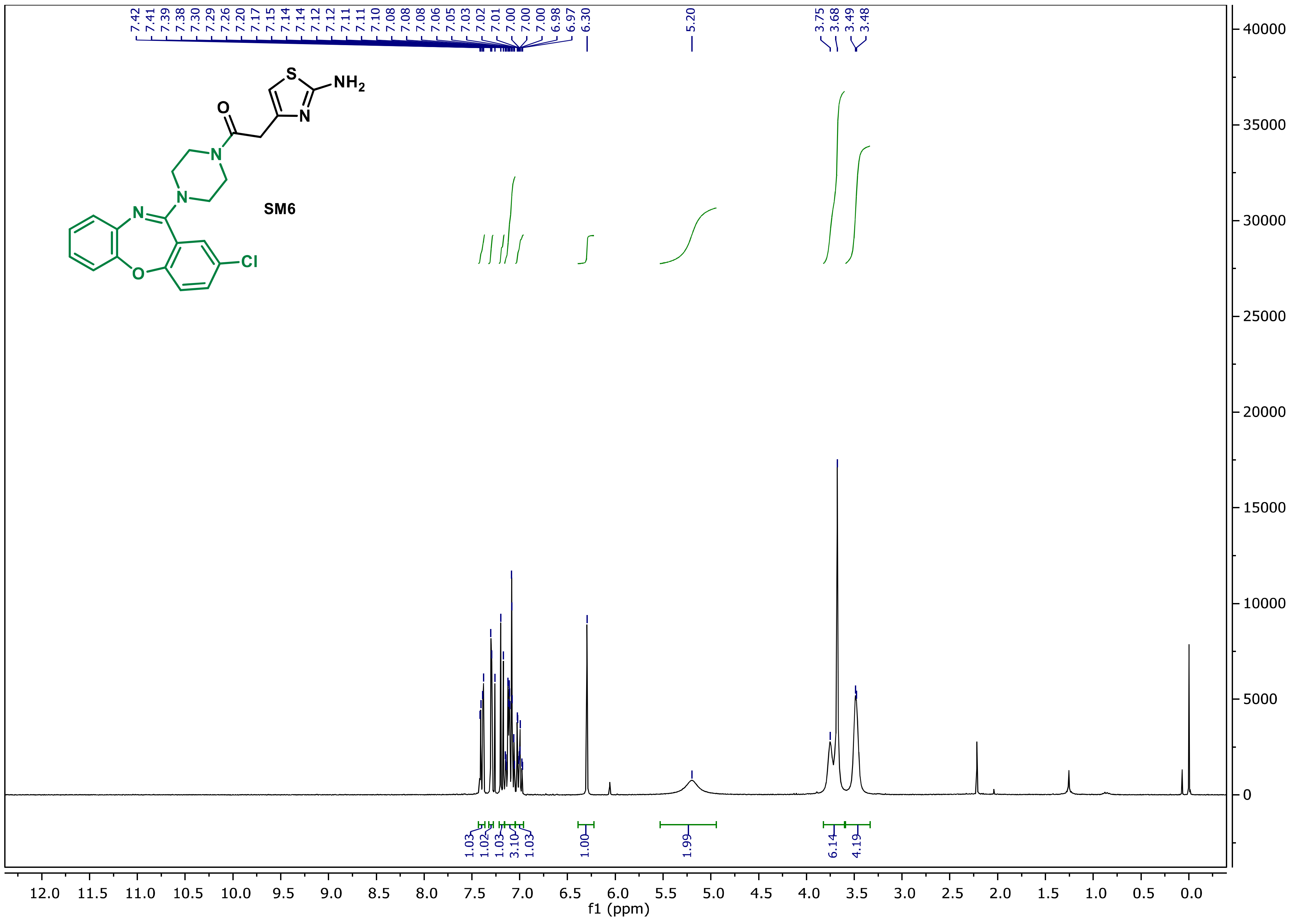
7.42 7.41 7.39 7.38 7.30 7.29 7.26 7.20 7.17 7.15 7.14 7.14 7.12 7.12 7.11 7.11 7.10 7.08 7.08 7.08 7.06 7.05 7.03 7.02 7.01 7.00 7.00 7.00 6.98 6.97 6.30
5.20
3.75 3.68 3.49 3.48
SM6
NH2
S
N
O
Cl
1.03 1.02 1.03 3.10 1.03
1.00
1.99
6.14 4.19
f1 (ppm)
40000 35000 30000 25000 20000 15000 10000 5000 0
12.0 11.5 11.0 10.5 10.0 9.5 9.0 8.5 8.0 7.5 7.0 6.5 6.0 5.5 5.0 4.5 4.0 3.5 3.0 2.5 2.0 1.5 1.0 0.5 0.0

^{13}C NMR

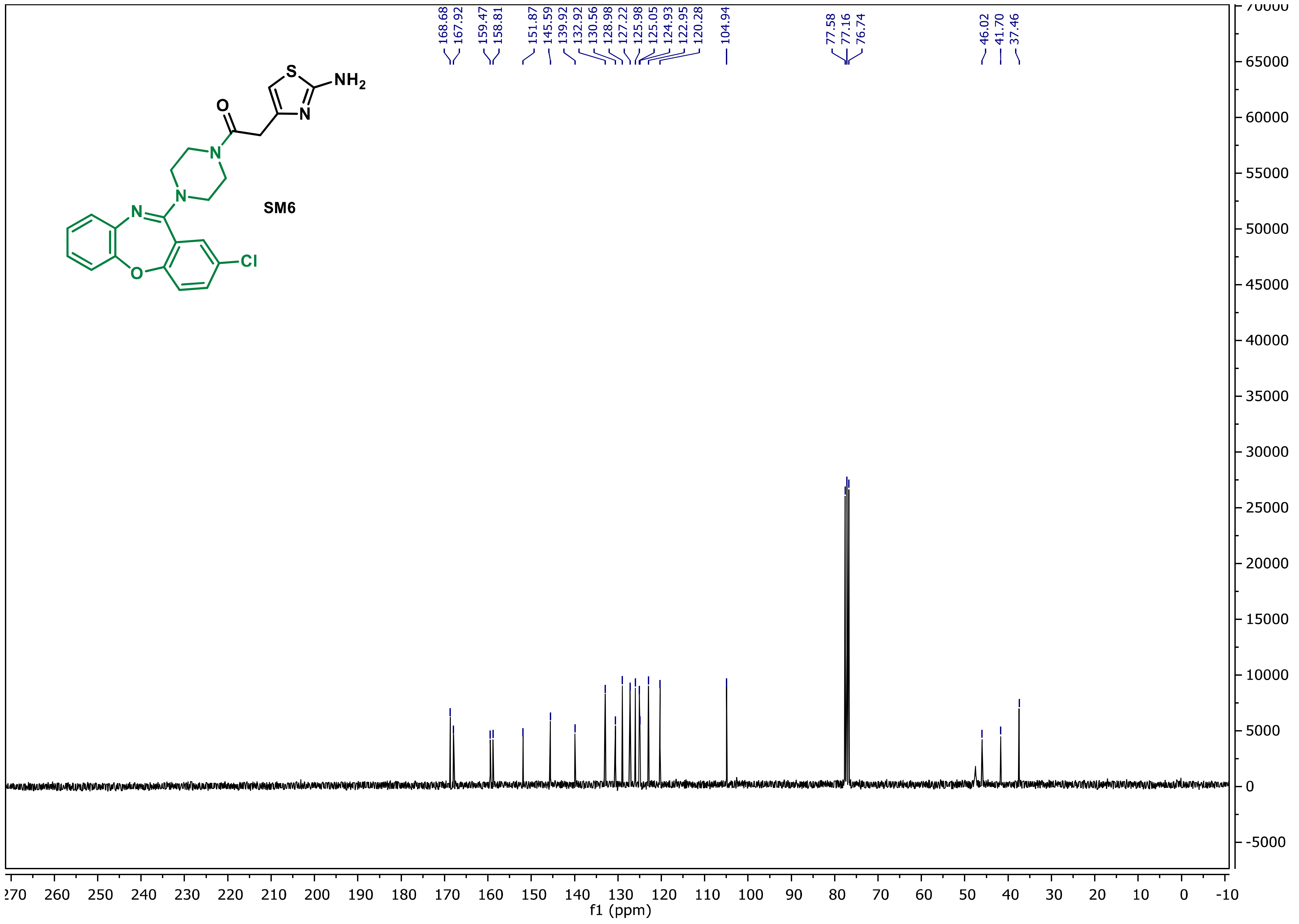

168.68
167.92
159.47
158.81
151.87
145.59
139.92
132.92
130.56
128.98
127.22
125.98
125.05
124.93
122.95
120.28
104.94
77.58
77.16
76.74
46.02
41.70
37.46
SM6
f1 (ppm)

Mass to be matched (m/z): 454.110070 Charge: 1

Mass Tolerance: ±0.005000
Restriction of atom numbers:

C	H	N	O	S	Cl
1-100	1-100	1-5	1-10	1-1	1-1

Number of calculated Formulas: 5

Formula	Diff.(ppm)	theor. m/z
C22 H21 N5 O2 S1 Cl1	-0.38	454.109899
C24 H23 N2 O3 S1 Cl1	2.58	454.111243
C21 H25 N1 O6 S1 Cl1	-3.32	454.108564
C19 H23 N4 O5 S1 Cl1	-6.28	454.107220
C12 H27 N4 O10 S1 Cl1	6.66	454.113096

Datum:	29.10.2020
Analyse:	150158c-00
Sigel:	GHC-GA-406-01<br>COP: Dr. Clement Ghiazza
Method:	HR-MS
Ionis. :	ESIpos
solvent :	CH2Cl2 + CH3OH
Spectrometer:	Exactive
Auswerter:	Marcus, Tel:2243

suggestion:
C22H20N5O2S1Cl1   MW: 453

Characteristic Ions:
454 = [453 + H]

¹H NMR

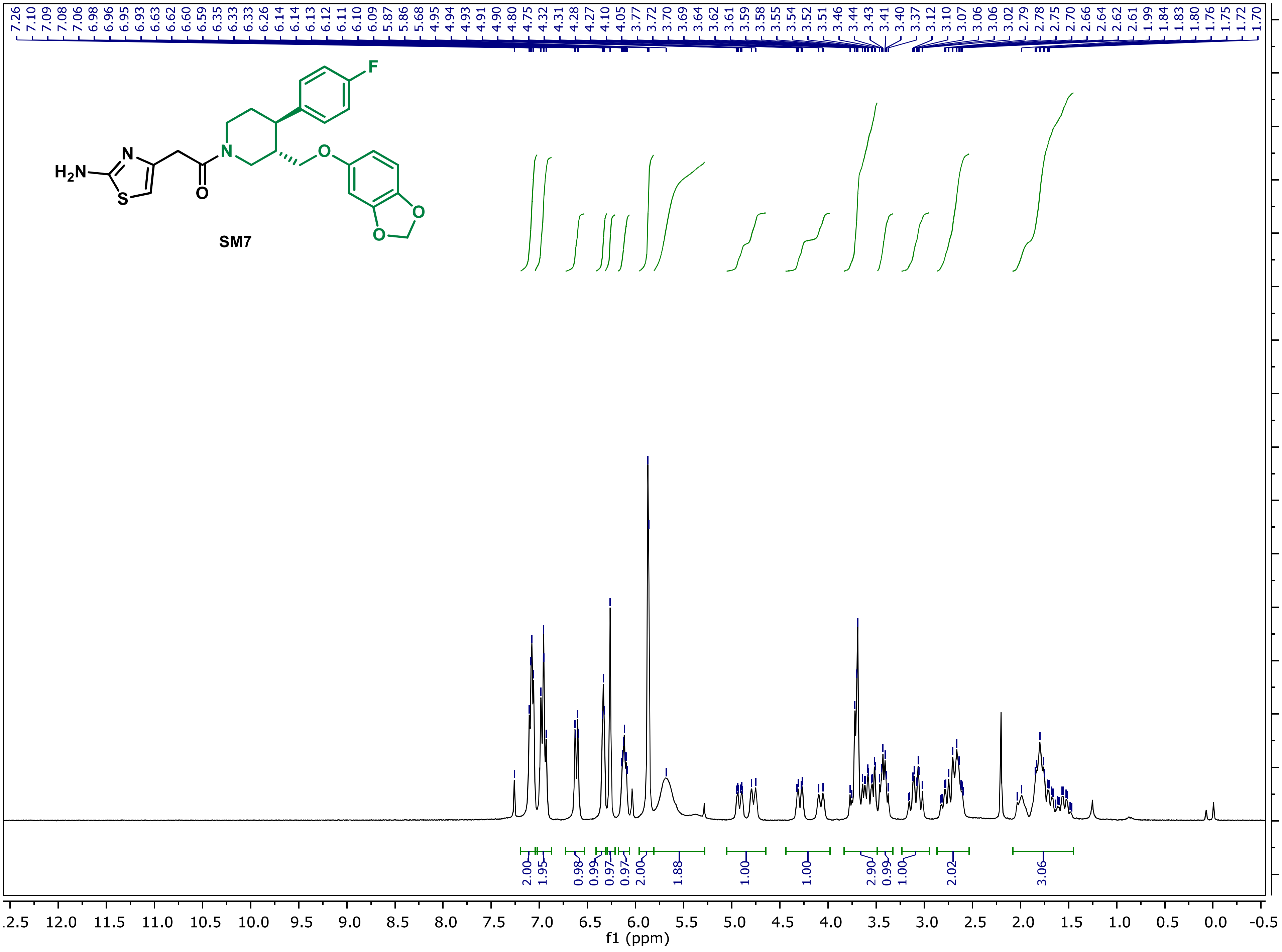
SM7
7.26 7.10 7.09 7.08 7.06 6.98 6.96 6.95 6.93 6.63 6.62 6.60 6.59 6.35 6.33 6.33 6.26 6.14 6.14 6.13 6.12 6.11 6.10 6.09 5.87 5.86 5.68 4.95 4.94 4.93 4.91 4.90 4.80 4.75 4.32 4.31 4.28 4.27 4.10 4.05 3.77 3.72 3.70 3.69 3.64 3.62 3.61 3.59 3.58 3.55 3.54 3.52 3.51 3.46 3.44 3.43 3.41 3.40 3.37 3.12 3.10 3.07 3.06 3.06 3.02 2.79 2.78 2.75 2.70 2.66 2.64 2.62 2.61 1.99 1.84 1.83 1.80 1.76 1.75 1.72 1.70
2.00 1.95 0.98 0.99 0.97 0.97 2.00 1.88 1.00 1.00 2.90 0.99 1.00 2.02 3.06
f1 (ppm)

^{13}C NMR

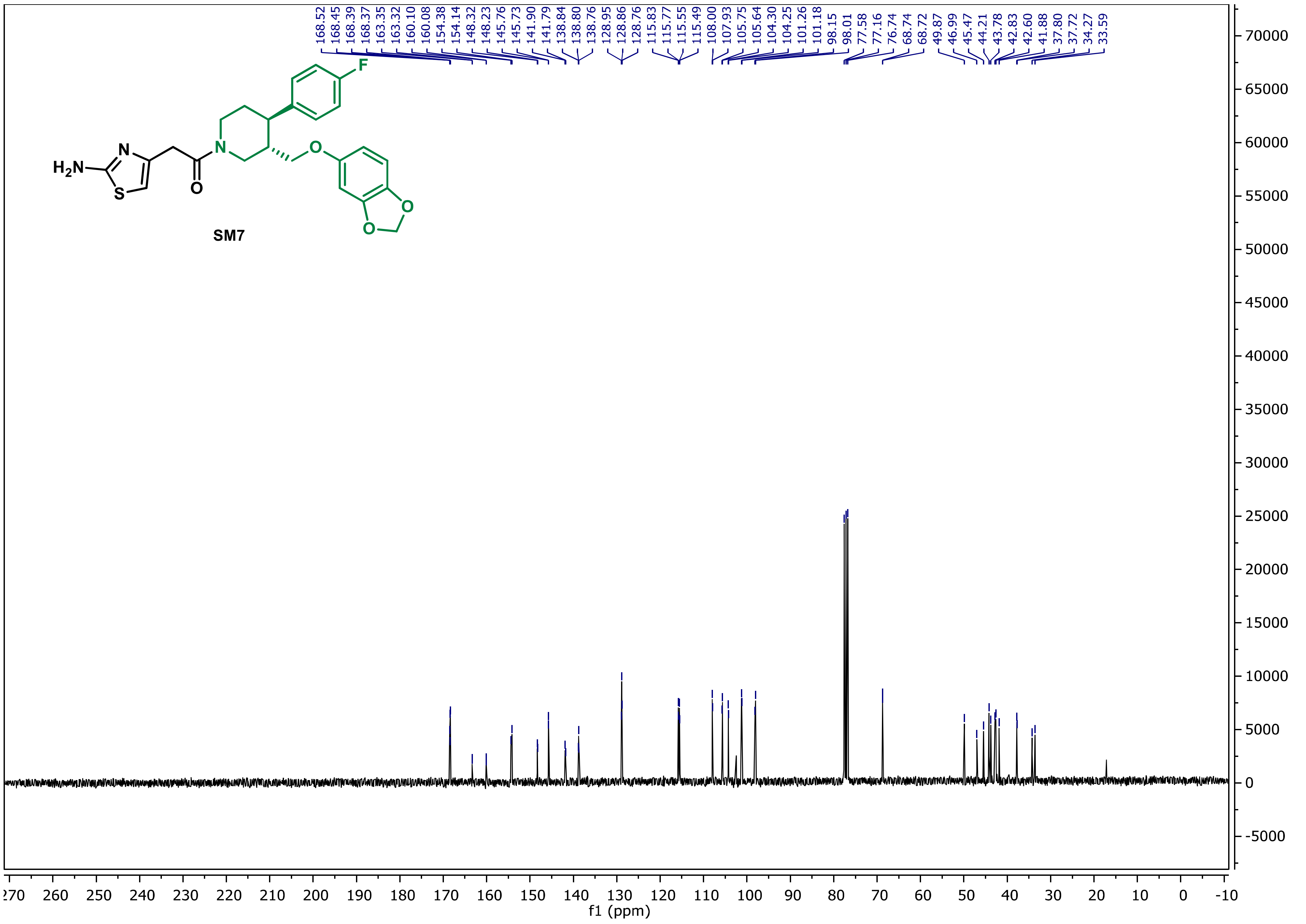

168.52
168.45
168.39
168.37
163.35
163.32
160.10
160.08
154.38
154.14
148.32
148.23
145.76
145.73
141.90
141.79
138.84
138.80
138.76
128.95
128.86
128.76
115.83
115.77
115.55
115.49
108.00
107.93
105.75
105.64
104.30
104.25
101.26
101.18
98.15
98.01
77.58
77.16
76.74
68.74
68.72
49.87
46.99
45.47
44.21
43.78
42.83
42.60
41.88
37.80
37.72
34.27
33.59
H2N
N
S
O
N
F
O
O
O
SM7
f1 (ppm)

^{19}F NMR

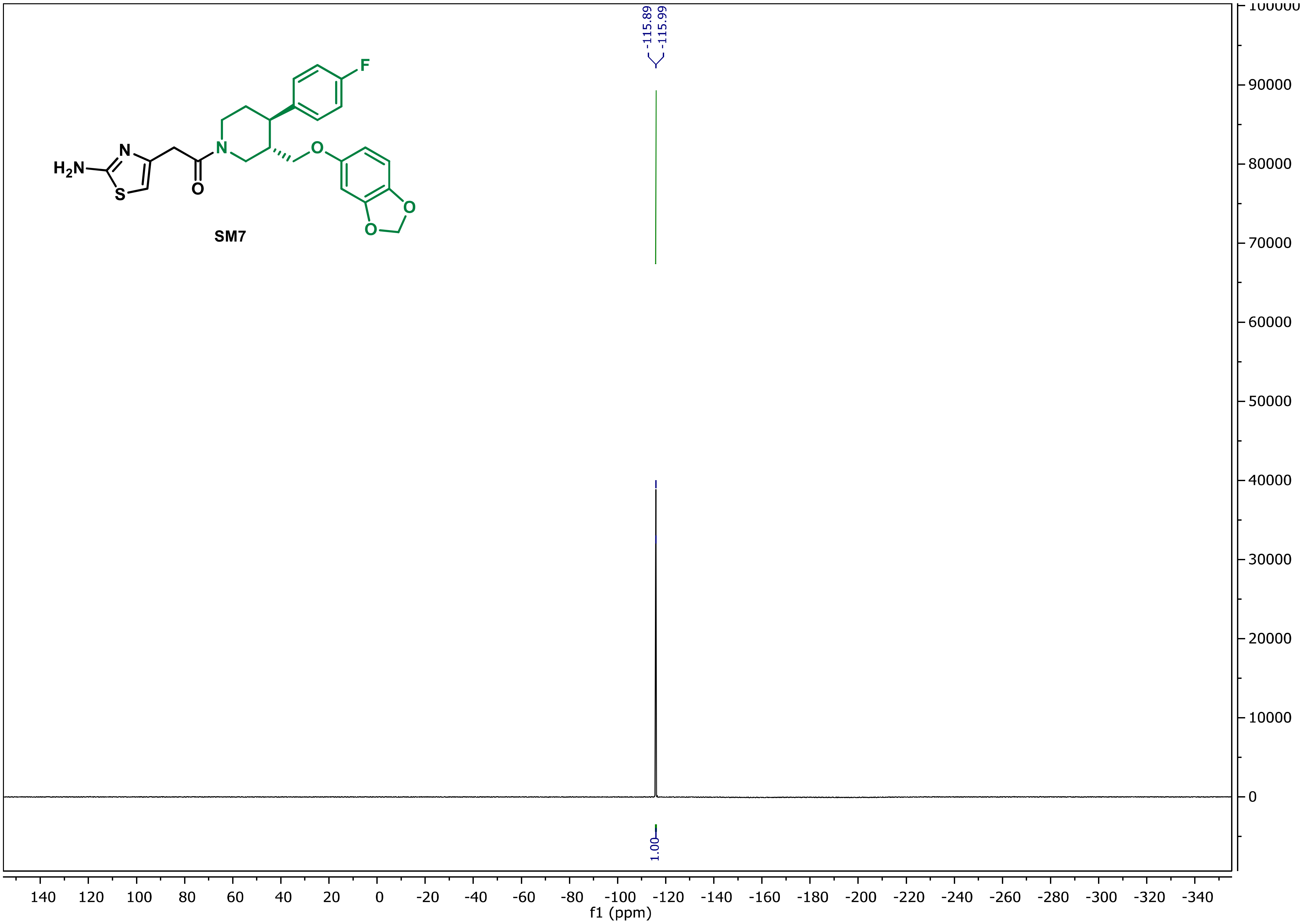
SM7
-115.89
-115.99
1.00
f1 (ppm)
140 120 100 80 60 40 20 0 -20 -40 -60 -80 -100 -120 -140 -160 -180 -200 -220 -240 -260 -280 -300 -320 -340
100000 90000 80000 70000 60000 50000 40000 30000 20000 10000 0

Mass to be matched (m/z): 470.154860 Charge: 1

Mass Tolerance: ±0.005000
Restriction of atom numbers:

C	H	N	O	S	F
1-100	1-100	1-3	1-10	1-1	1-1

Number of calculated Formulas: 3

Formula	Diff.(ppm)	theor. m/z
C24 H25 N3 O4 S1 F1	-0.91	470.154432
C21 H27 N2 O7 S1 F1	-6.61	470.151753
C29 H25 N1 O2 S1 F1	7.65	470.158455

Datum:	25.02.2021
Analyse:	151917b-00
Sigel:	GHC-GA-615-01
	COP: Dr. Clement Ghiazza
Method:	HR-MS
Ionis. :	ESIpos
solvent :	CH2Cl2 + CH3OH
Spectrometer:	Exactive
Auswerter:	Marcus, Tel:2243

suggestion:
C24H24N3O4S1F1 MW: 469

Characteristic Ions:
470 = [469 + H]

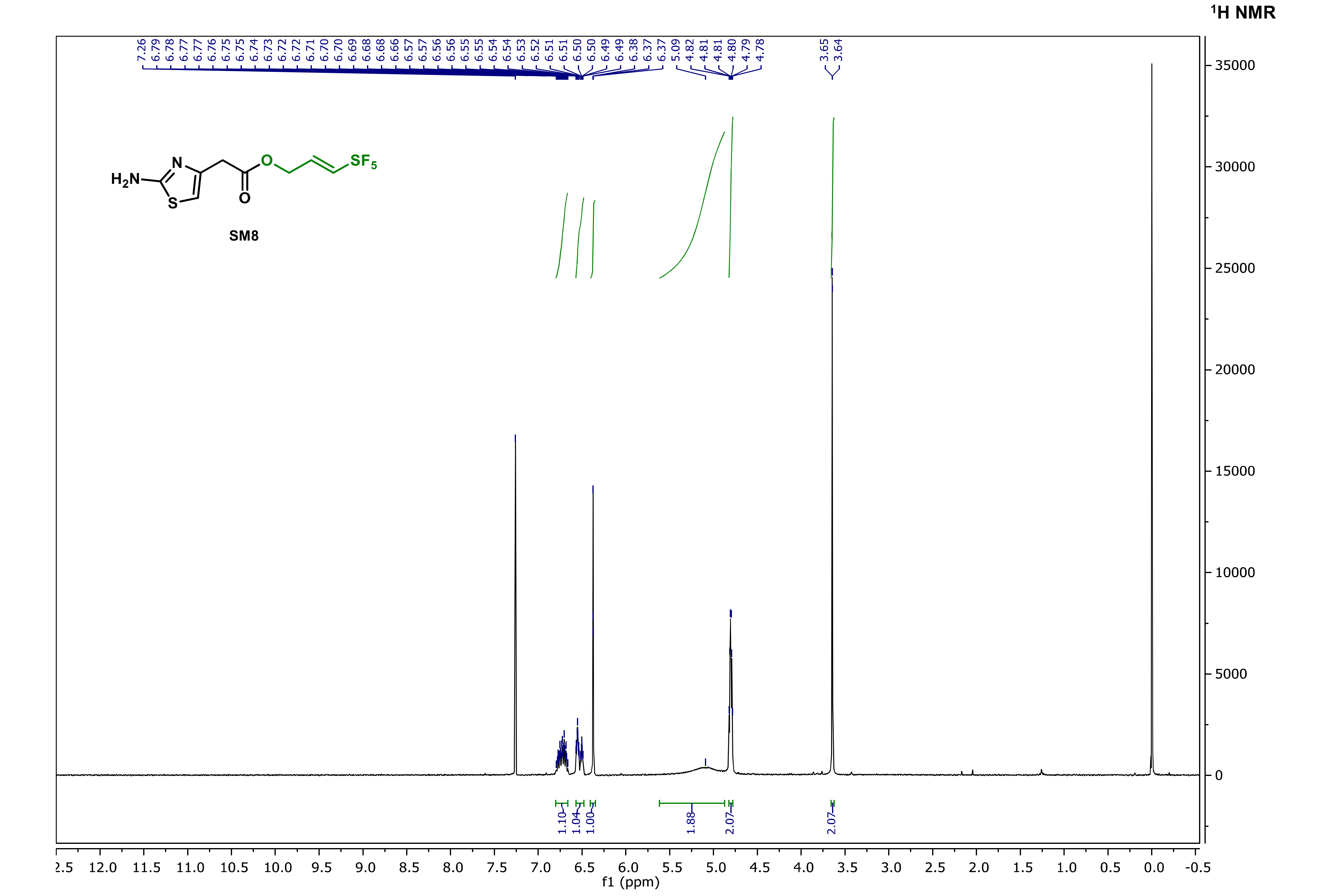

¹H NMR
7.26
6.79
6.78
6.77
6.77
6.76
6.75
6.75
6.74
6.73
6.72
6.72
6.71
6.70
6.70
6.69
6.68
6.68
6.66
6.57
6.57
6.56
6.56
6.55
6.55
6.54
6.54
6.53
6.52
6.51
6.51
6.50
6.50
6.49
6.49
6.38
6.37
6.37
5.09
4.82
4.81
4.81
4.80
4.79
4.78
3.65
3.64
H2N
N
S
O
O
SF5
SM8
1.10
1.04
1.00
1.88
2.07
2.07
35000
30000
25000
20000
15000
10000
5000
0
12.5
12.0
11.5
11.0
10.5
10.0
9.5
9.0
8.5
8.0
7.5
7.0
6.5
6.0
5.5
5.0
4.5
4.0
3.5
3.0
2.5
2.0
1.5
1.0
0.5
0.0
-0.5
f1 (ppm)

^{13}C NMR

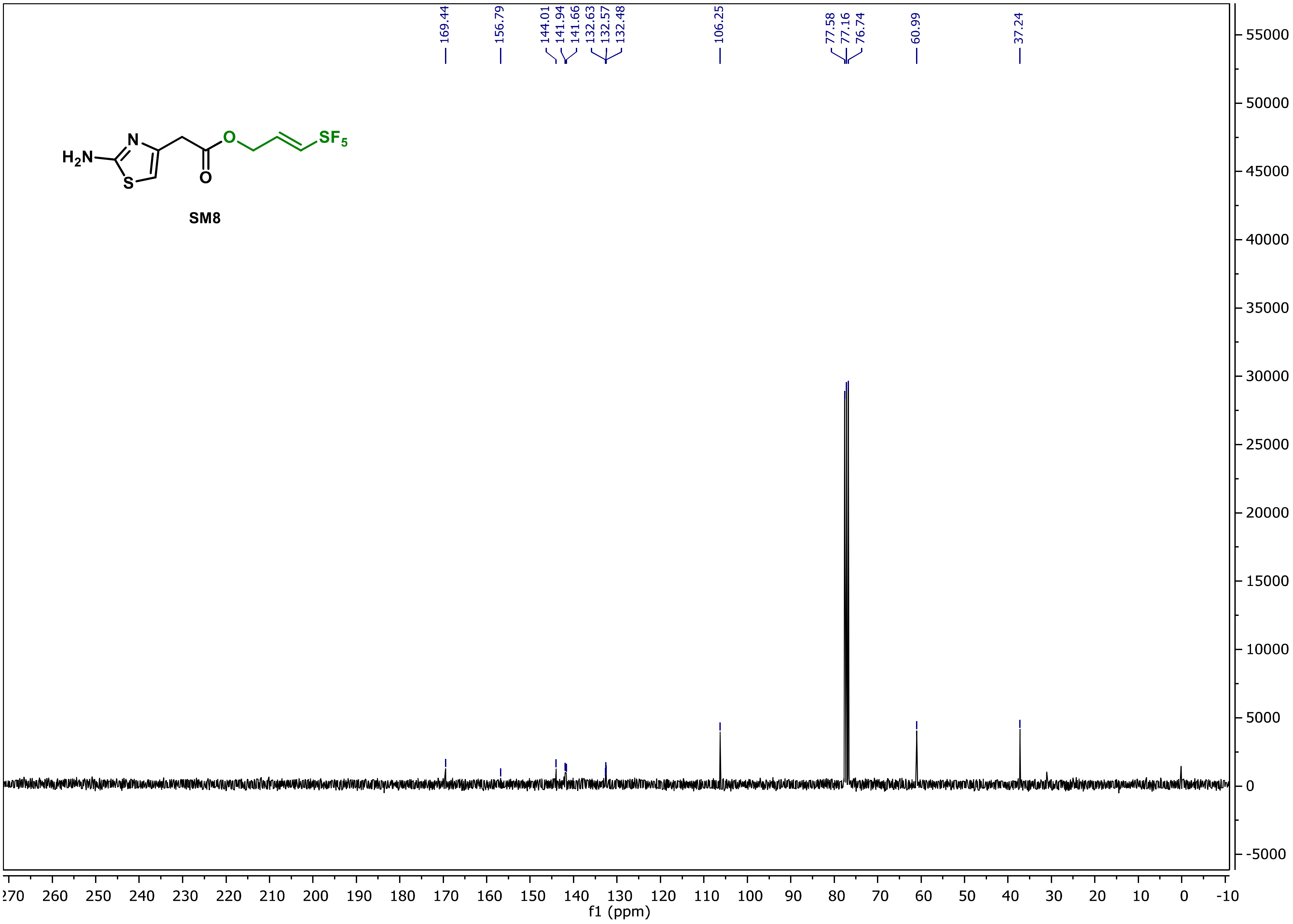

H2N
N
S
O
O
SF5
SM8
169.44
156.79
144.01
141.94
141.66
132.63
132.57
132.48
106.25
77.58
77.16
76.74
60.99
37.24
55000
50000
45000
40000
35000
30000
25000
20000
15000
10000
5000
0
-5000
270 260 250 240 230 220 210 200 190 180 170 160 150 140 130 120 110 100 90 80 70 60 50 40 30 20 10 0 -10
f1 (ppm)

^{19}F NMR

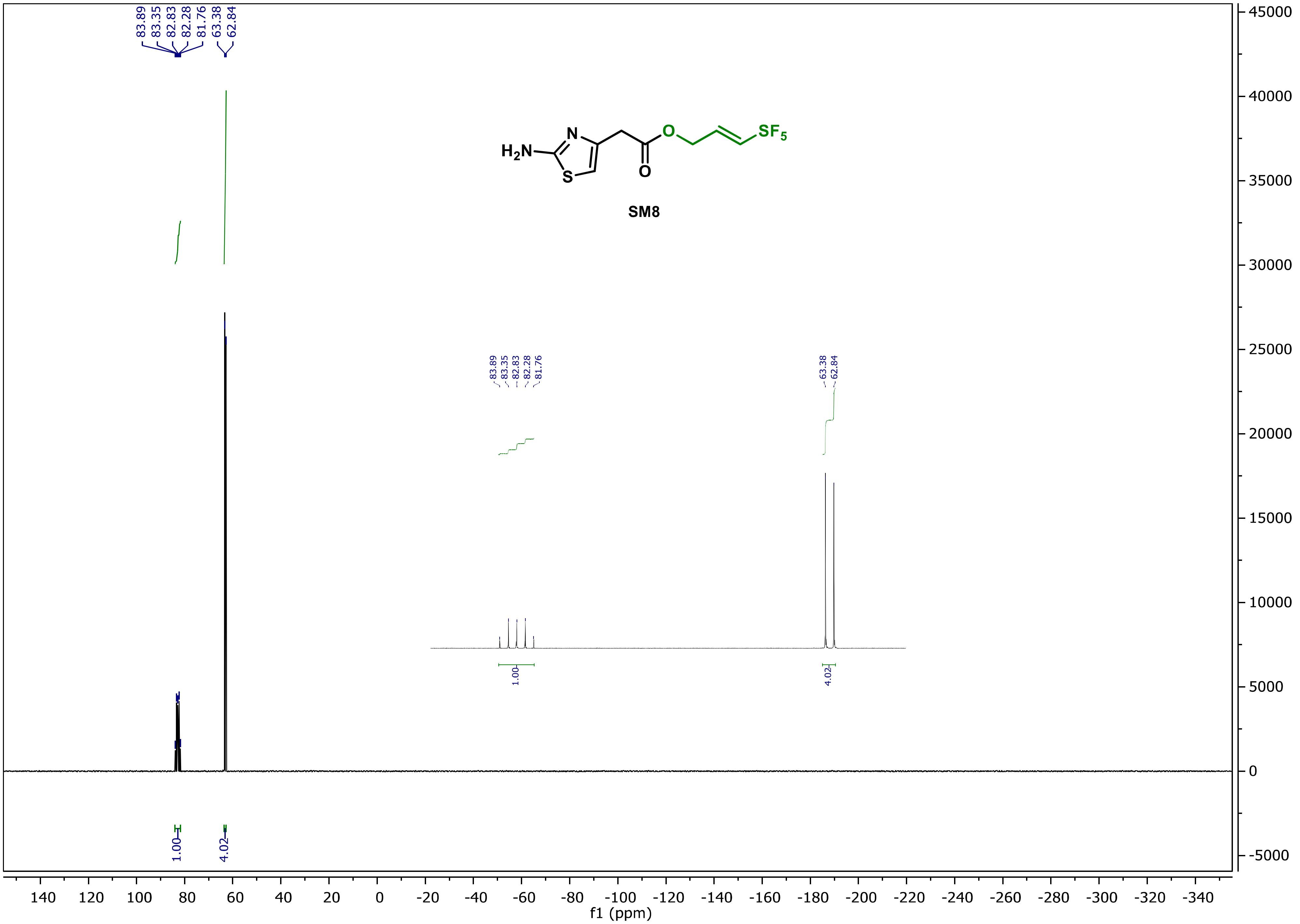

83.89
83.35
82.83
82.28
81.76
63.38
62.84
H2N
N
S
O
O
SF5
SM8
1.00
4.02
f1 (ppm)

Mass to be matched (m/z): 325.009620 Charge: 1

Mass Tolerance: ±0.005000
Restriction of atom numbers:

C	H	N	O	S	F
1-100	1-100	1-5	1-10	2-2	5-5

Number of calculated Formulas: 4

Formula	Diff.(ppm)	theor. m/z
C8 H10 N2 O2 S2 F5	0.68	325.009840
C6 H8 N5 O1 S2 F5	-3.46	325.008496
C5 H12 N1 O5 S2 F5	-7.56	325.007161
C3 H10 N4 O4 S2 F5	-11.70	325.005818

Datum:	20.05.2021
Analyse:	153436c-00
Sigel:	GHC-GA-727-01
	COP: Dr. Clement Ghiazza
Method:	HR-MS
Ionis. :	ESIpos
solvent :	CH2Cl2 + CH3OH
Spectrometer:	Exactive
Auswerter:	Marcus, Tel:2243

suggestion:
C8H9N2O2S2F5   MW: 324

Characteristic Ions:
325 = [324 + H]

^{1}H NMR

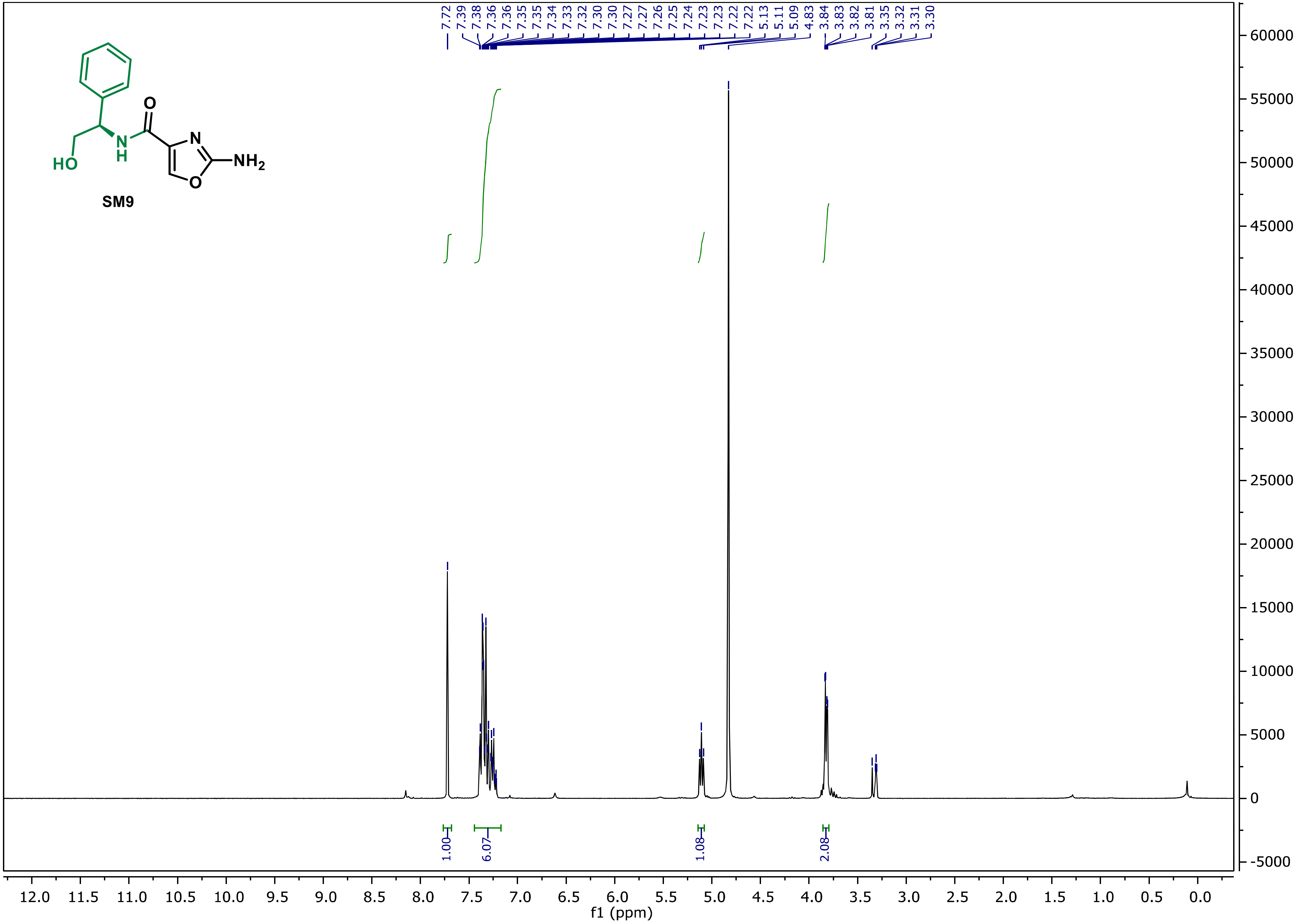

SM9
HO
N
H
O
N
O
NH2
7.72
7.39
7.38
7.36
7.36
7.35
7.35
7.34
7.33
7.32
7.30
7.30
7.27
7.27
7.26
7.25
7.24
7.23
7.23
7.22
7.22
5.13
5.11
5.09
4.83
3.84
3.83
3.82
3.81
3.35
3.32
3.31
3.30
1.00
6.07
1.08
2.08
60000
55000
50000
45000
40000
35000
30000
25000
20000
15000
10000
5000
0
-5000
12.0 11.5 11.0 10.5 10.0 9.5 9.0 8.5 8.0 7.5 7.0 6.5 6.0 5.5 5.0 4.5 4.0 3.5 3.0 2.5 2.0 1.5 1.0 0.5 0.0
f1 (ppm)

^{13}C NMR

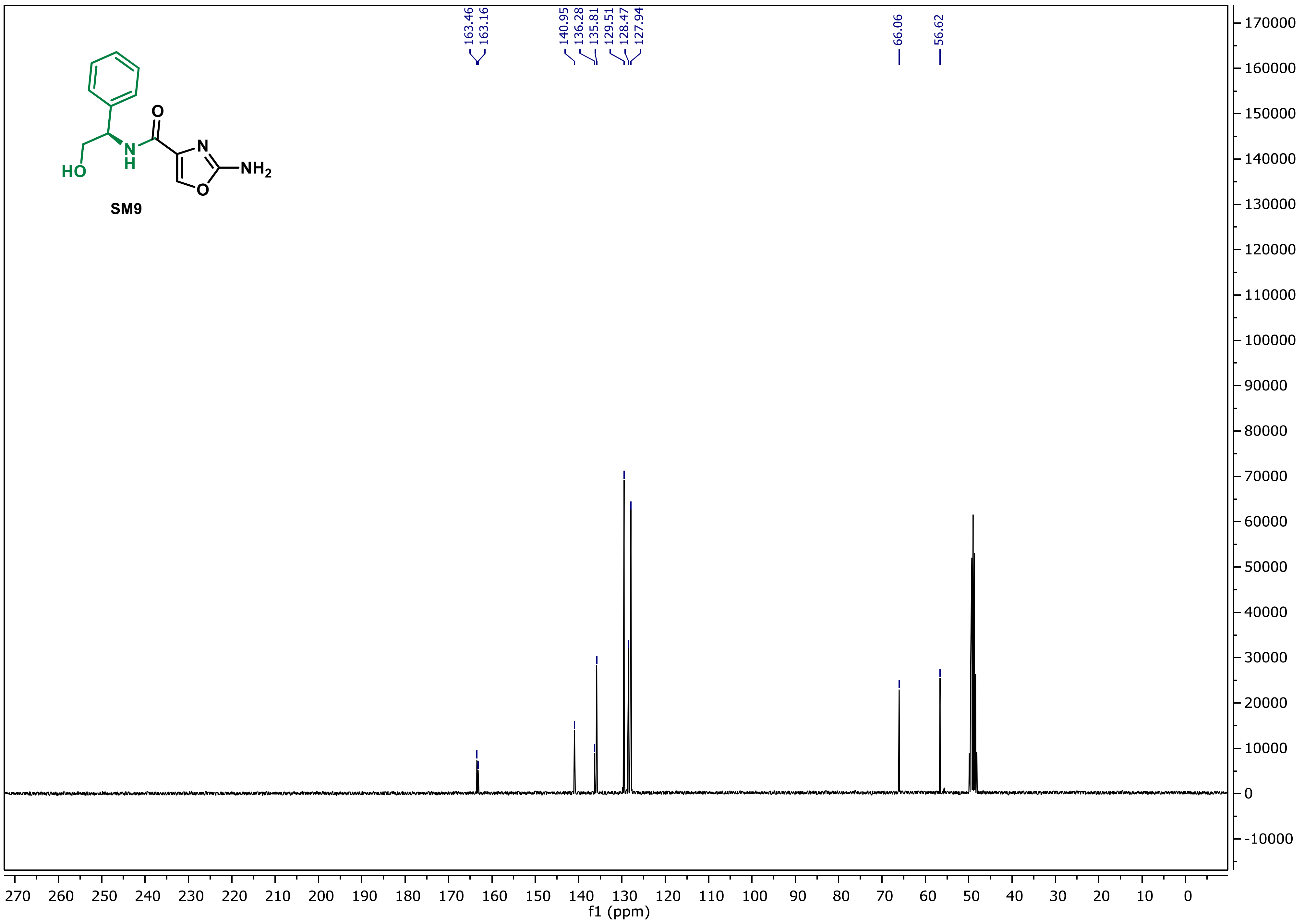

SM9
HO
N
H
O
N
NH2
O
163.46
163.16
140.95
136.28
135.81
129.51
128.47
127.94
66.06
56.62
170000
160000
150000
140000
130000
120000
110000
100000
90000
80000
70000
60000
50000
40000
30000
20000
10000
0
-10000
270 260 250 240 230 220 210 200 190 180 170 160 150 140 130 120 110 100 90 80 70 60 50 40 30 20 10 0
f1 (ppm)

Mass to be matched (m/z): 270.084710 Charge: 1

Mass Tolerance: ±0.005000
Restriction of atom numbers:

C	H	N	O	Na
1-100	1-100	1-3	max 10	1-1

Number of calculated Formulas: 3

Formula	Diff.(ppm)	theor. m/z
C12 H13 N3 O3 Na1	0.74	270.084910
C9 H15 N2 O6 Na1	-9.18	270.082231
C17 H13 N1 O1 Na1	15.64	270.088933

Datum	22.09.2020
Analyse:	149430c-00
Sigel:	GHC-AA-030-01
COP: Dr. Clement Ghiazza	
Messung:	HRMS
Methode:	ESIpos
Lösungsmittel:	CH3OH
Spektrometer:	Exactive
Auswerter:	Kampen (2242)

Suggestion:
C12H13N3O3 MW 247

characteristical ion
270 = [247 + Na]+

1H NMR

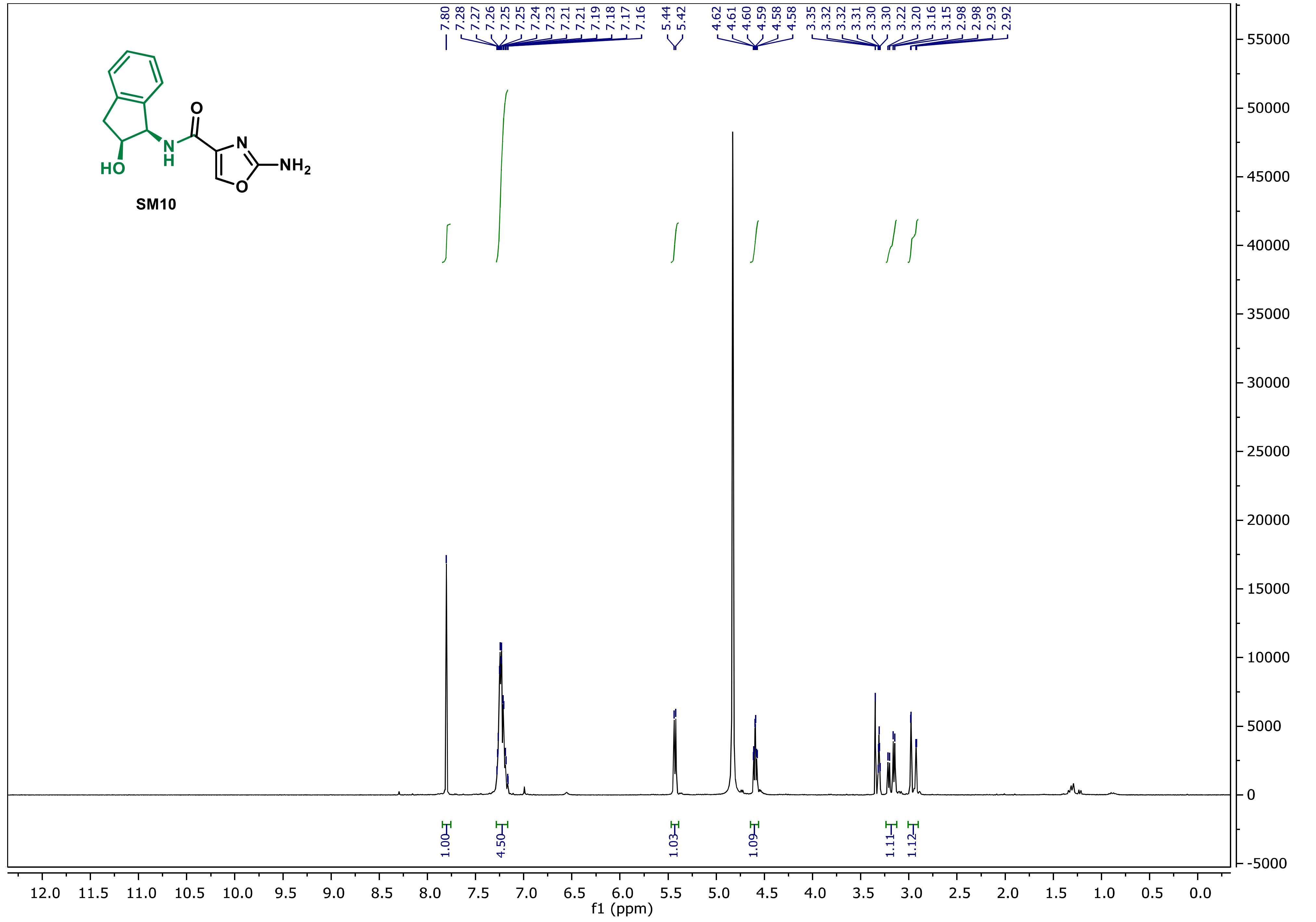

HO
N
H
O
N
NH2
O
SM10
7.80
7.28
7.27
7.26
7.25
7.25
7.24
7.23
7.21
7.21
7.19
7.18
7.17
7.16
5.44
5.42
4.62
4.61
4.60
4.59
4.58
4.58
3.35
3.32
3.32
3.31
3.30
3.30
3.22
3.20
3.16
3.15
2.98
2.98
2.93
2.92
1.00
4.50
1.03
1.09
1.11
1.12
f1 (ppm)

^{13}C NMR

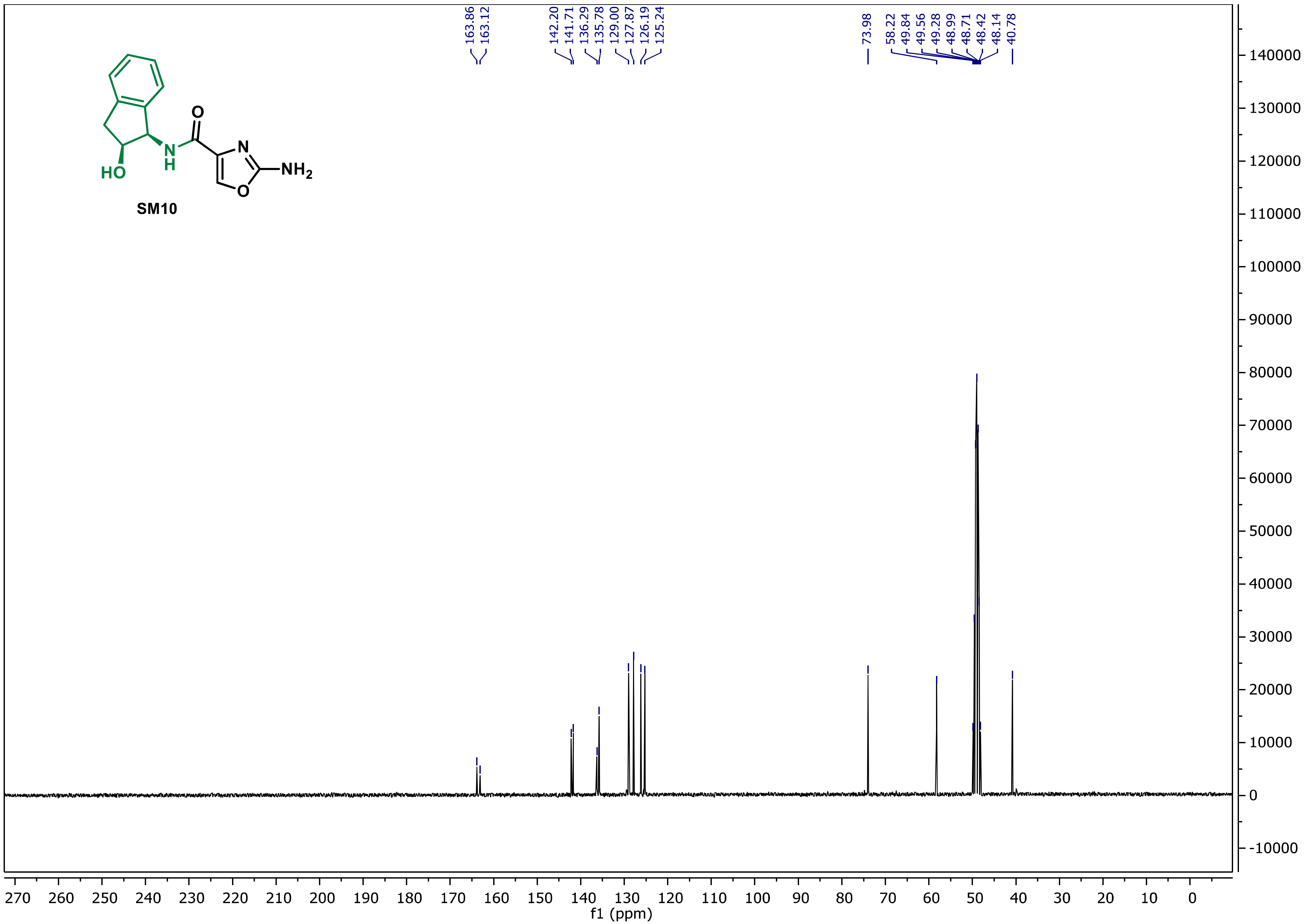

163.86
163.12
142.20
141.71
136.29
135.78
129.00
127.87
126.19
125.24
73.98
58.22
49.84
49.56
49.28
48.99
48.71
48.42
48.14
40.78
140000
130000
120000
110000
100000
90000
80000
70000
60000
50000
40000
30000
20000
10000
0
-10000
270 260 250 240 230 220 210 200 190 180 170 160 150 140 130 120 110 100 90 80 70 60 50 40 30 20 10 0
f1 (ppm)

SM10

Mass to be matched (m/z): 260.103220 Charge: 1

Mass Tolerance: ±0.005000
Restriction of atom numbers:

C	H	N	O
1-100	1-100	1-5	1-10

Number of calculated Formulas: 4

Formula	Diff.(ppm)	theor. m/z
C13 H14 N3 O3	-0.98	260.102966
C10 H16 N2 O6	-11.27	260.100287
C18 H14 N1 O1	14.49	260.106989
C8 H14 N5 O5	-16.44	260.098944

Datum:	1.10.2020
Analyse:	149625c-00
Sigel:	GHC-AA-042-01<br>COP: Dr. Clement Ghiazza
Method:	HR-MS
Ionis. :	ESIpos
solvent :	CH2Cl2 + CH3OH
Spectrometer:	Exactive
Auswerter:	Marcus, Tel:2243

suggestion:
C13H13N3O3 MW: 259

Characteristic Ions:
260 = [259 + H]

^{1}H NMR

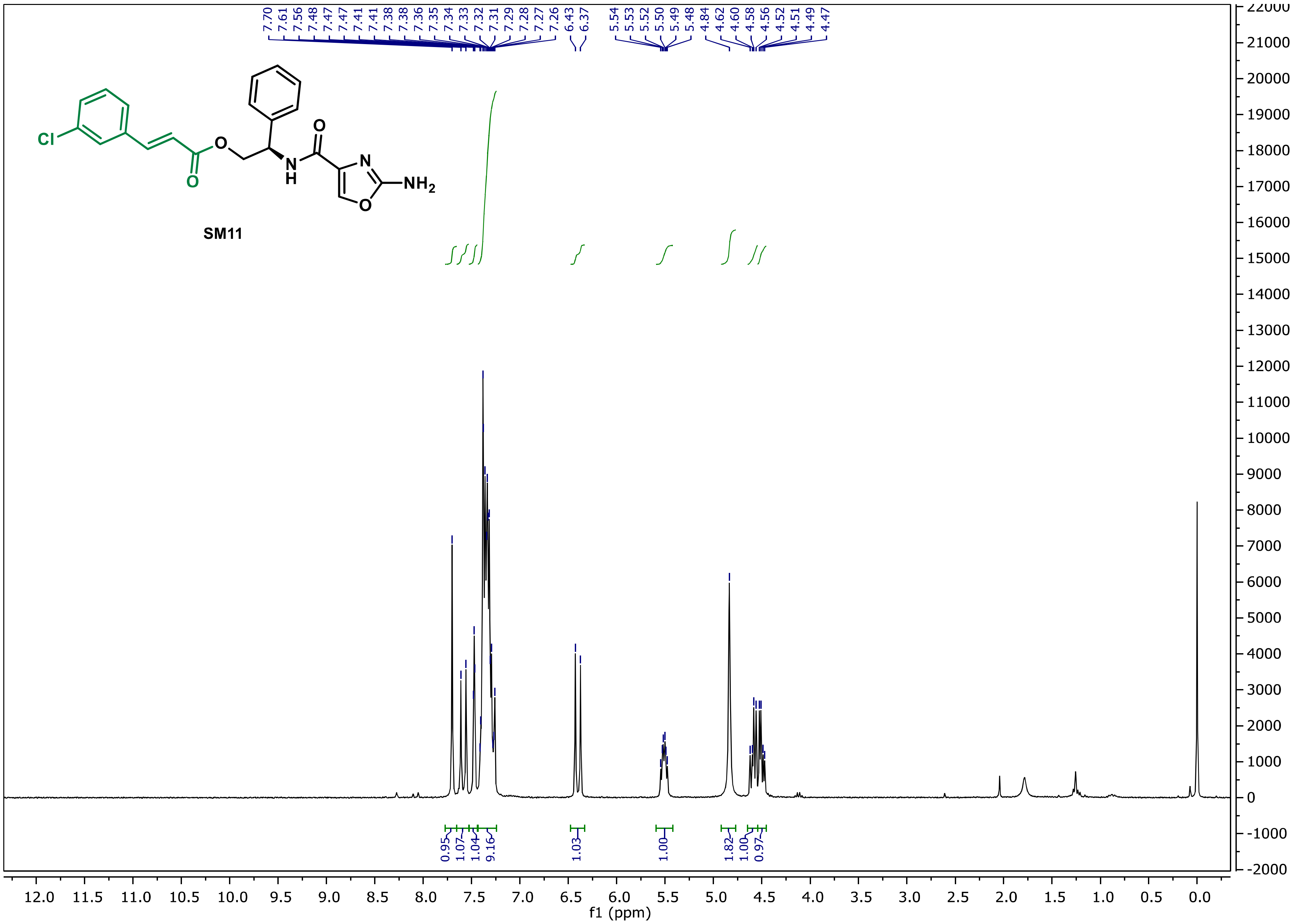

SM11

7.70 7.61 7.56 7.48 7.47 7.47 7.41 7.41 7.38 7.38 7.36 7.35 7.34 7.33 7.32 7.31 7.29 7.28 7.27 7.26 6.43 6.37 5.54 5.53 5.52 5.50 5.49 5.48 4.84 4.62 4.60 4.58 4.56 4.52 4.51 4.49 4.47
0.95 1.07 1.04 9.16 1.03 1.00 1.82 1.00 0.97
22000 21000 20000 19000 18000 17000 16000 15000 14000 13000 12000 11000 10000 9000 8000 7000 6000 5000 4000 3000 2000 1000 0 -1000 -2000
12.0 11.5 11.0 10.5 10.0 9.5 9.0 8.5 8.0 7.5 7.0 6.5 6.0 5.5 5.0 4.5 4.0 3.5 3.0 2.5 2.0 1.5 1.0 0.5 0.0
f1 (ppm)

^{13}C NMR

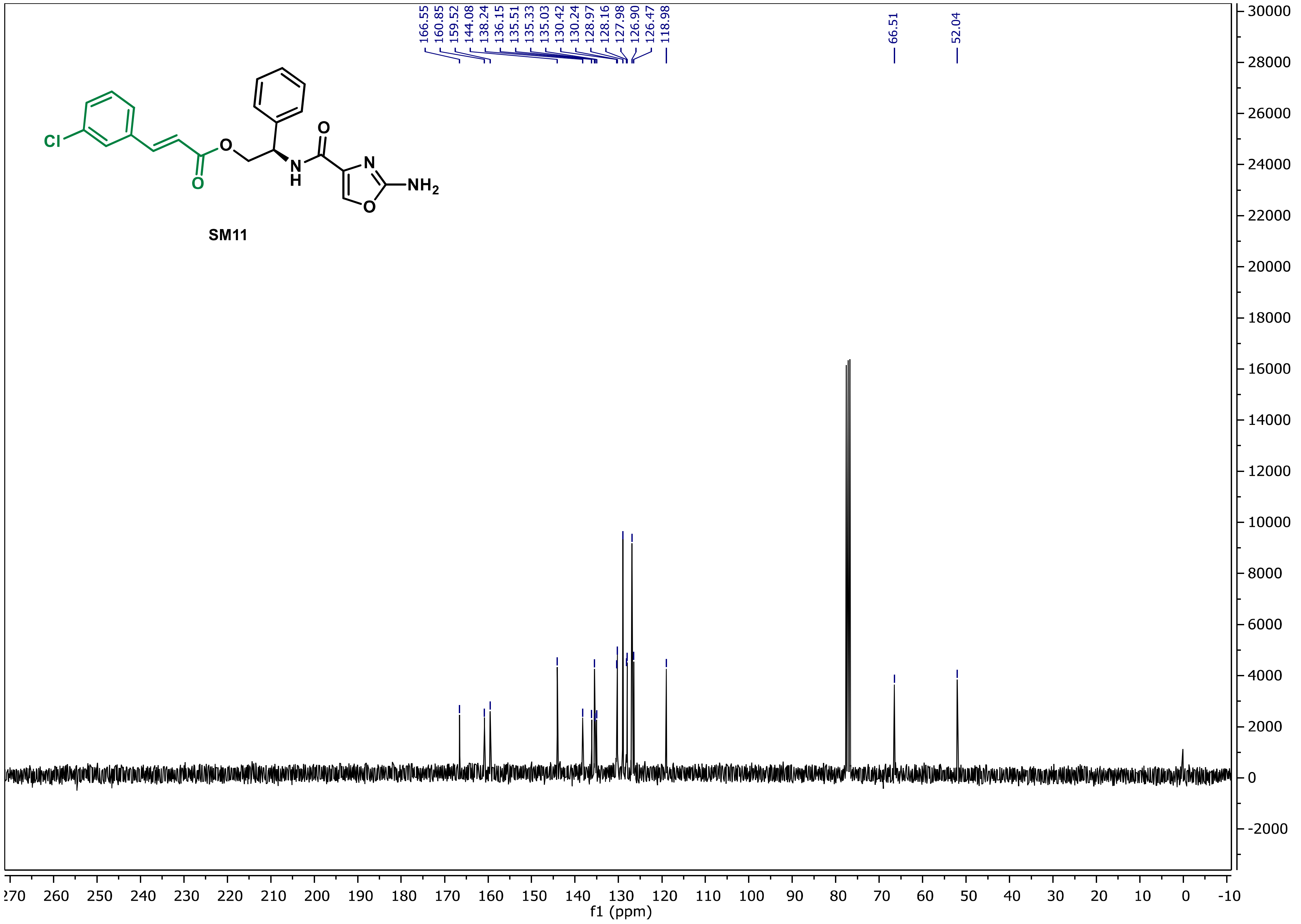

166.55
160.85
159.52
144.08
138.24
136.15
135.51
135.33
135.03
130.42
130.24
128.97
128.16
127.98
126.90
126.47
118.98
66.51
52.04
Cl
O
O
N
H
O
N
O
NH2
SM11
f1 (ppm)

*** Angegebene Mol.-Gewichte u. Massenzahlen basieren auf dem häufigsten Isotop der Elemente ***

MassLib

Mass to be matched (m/z): 434.087680 Charge: 1

Mass Tolerance: ±0.005000
Restriction of atom numbers:

C	H	Cl	N	O	Na
1-100	1-100	1-1	1-3	max 10	1-1

Number of calculated Formulas: 3

Formula	Diff.(ppm)	theor. m/z
C21 H18 Cl1 N3 O4 Na1	0.28	434.087803
C18 H20 Cl1 N2 O7 Na1	-5.89	434.085125
C26 H18 Cl1 N1 O2 Na1	9.55	434.091826

Datum	5.10.2020
Analyse:	149709c-00
Sigel:	GHC-AA-044-01
COP: Dr. Clement Ghiazza	
Messung:	HRMS
Methode:	ESIpos
Lösungsmittel:	CH2Cl2+CH3OH
Spektrometer:	Exactive
Auswerter:	Kampen (2242)

Suggestion:
C21H18Cl1N3O4 MW 411

characteristical ion
434 = [411 + Na]+

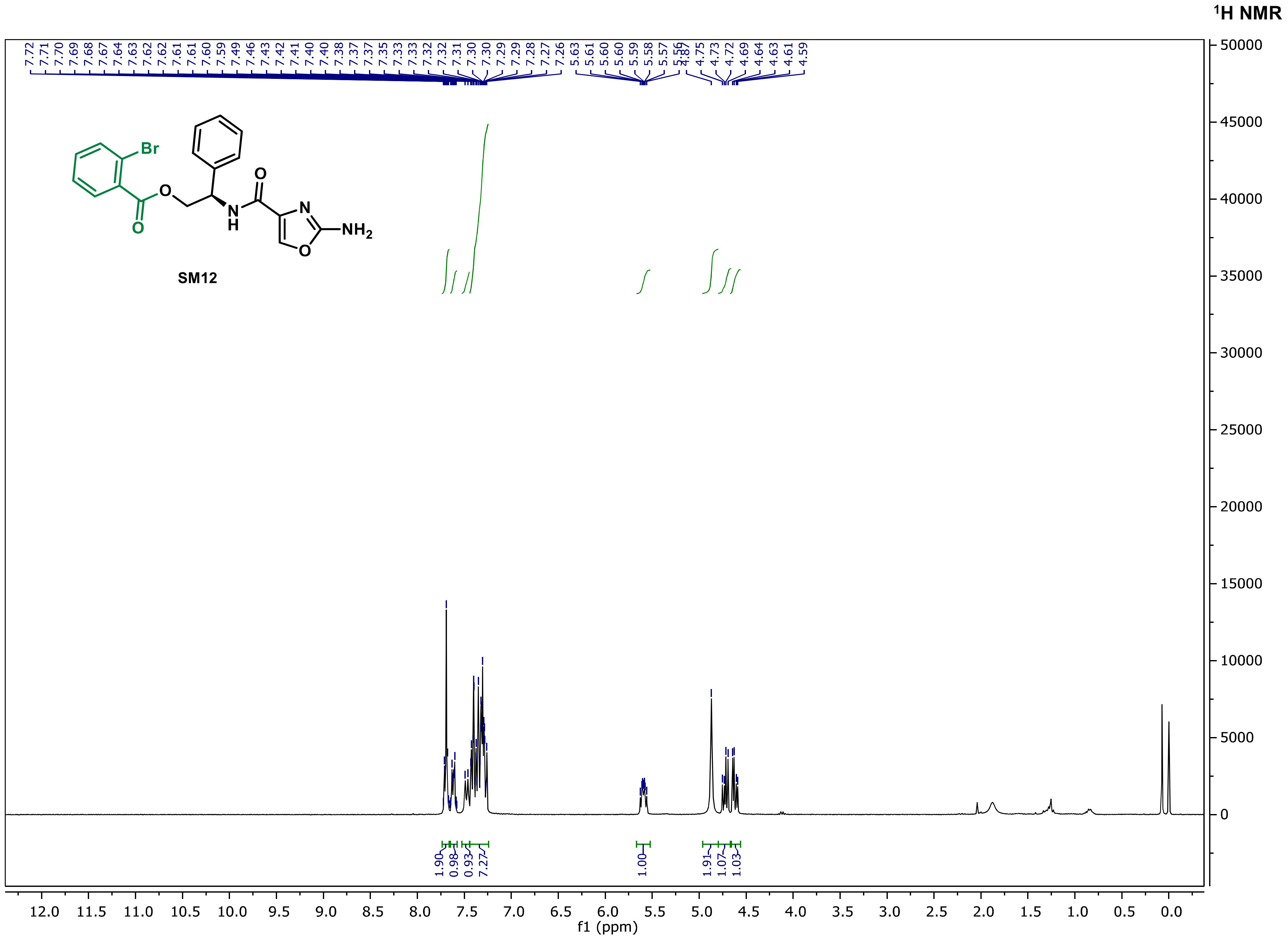
¹H NMR
SM12
Br
O
N
H
N
NH2
7.72 7.71 7.70 7.69 7.68 7.67 7.64 7.63 7.62 7.62 7.61 7.61 7.60 7.59 7.49 7.46 7.43 7.42 7.41 7.40 7.40 7.38 7.37 7.37 7.35 7.33 7.33 7.32 7.32 7.31 7.30 7.30 7.29 7.29 7.28 7.27 7.26
5.63 5.61 5.60 5.60 5.59 5.58 5.57 5.56
4.87 4.75 4.73 4.72 4.69 4.64 4.63 4.61 4.59
1.90 0.98 0.93 7.27 1.00 1.91 1.07 1.03
50000 45000 40000 35000 30000 25000 20000 15000 10000 5000 0
12.0 11.5 11.0 10.5 10.0 9.5 9.0 8.5 8.0 7.5 7.0 6.5 6.0 5.5 5.0 4.5 4.0 3.5 3.0 2.5 2.0 1.5 1.0 0.5 0.0
f1 (ppm)

^{13}C NMR

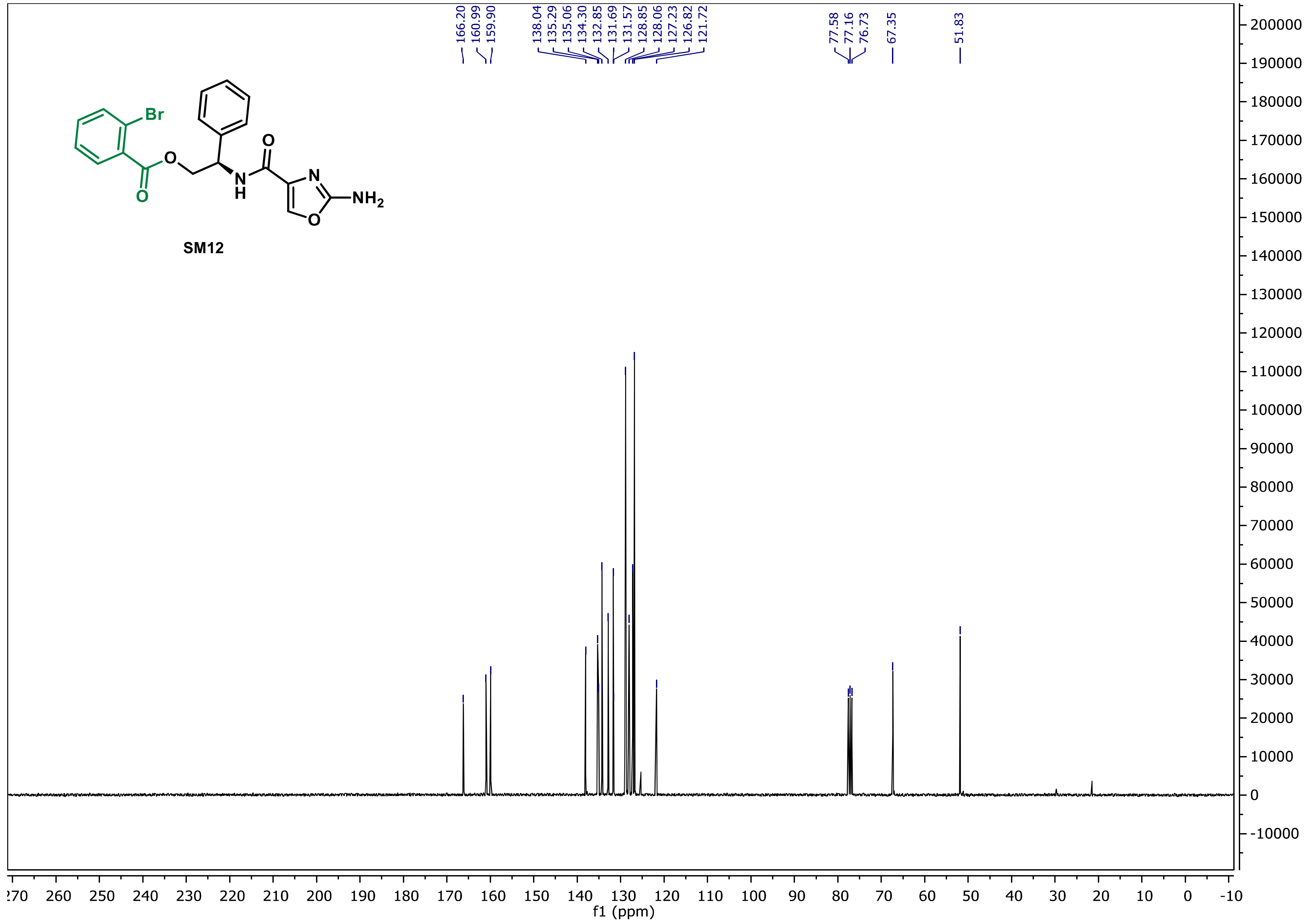

SM12
166.20
160.99
159.90
138.04
135.29
135.06
134.30
132.85
131.69
131.57
128.85
128.06
127.23
126.82
121.72
77.58
77.16
76.73
67.35
51.83
200000
190000
180000
170000
160000
150000
140000
130000
120000
110000
100000
90000
80000
70000
60000
50000
40000
30000
20000
10000
0
-10000
270
260
250
240
230
220
210
200
190
180
170
160
150
140
130
120
110
100
90
80
70
60
50
40
30
20
10
0
-10
f1 (ppm)

Mass to be matched (m/z): 452.021330 Charge: 1

Mass Tolerance: ±0.005000
Restriction of atom numbers:

C	H	N	O	Br	Na
1-110	1-100	1-3	1-4	1-1	1-1

Number of calculated Formulas: 2

Formula	Diff.(ppm)	theor. m/z
C19 H16 N3 O4 Br1 Na1	0.71	452.021650
C24 H16 N1 O2 Br1 Na1	9.61	452.025673

09.10.2020
File: 149797b-00

Analyse: GHC-AA-059-01
COP: Dr. Clement Ghiazza

Messung:	HRMS ESIpos
Lösemittel:	CH3OH
Spektrometer:	Exactive
Auswerter:	Kohler (2243)

Suggestion:
C19H16N3O4Br1 Mw 429

Characteristicial ions:
452 = [429 + Na]+

^1H NMR

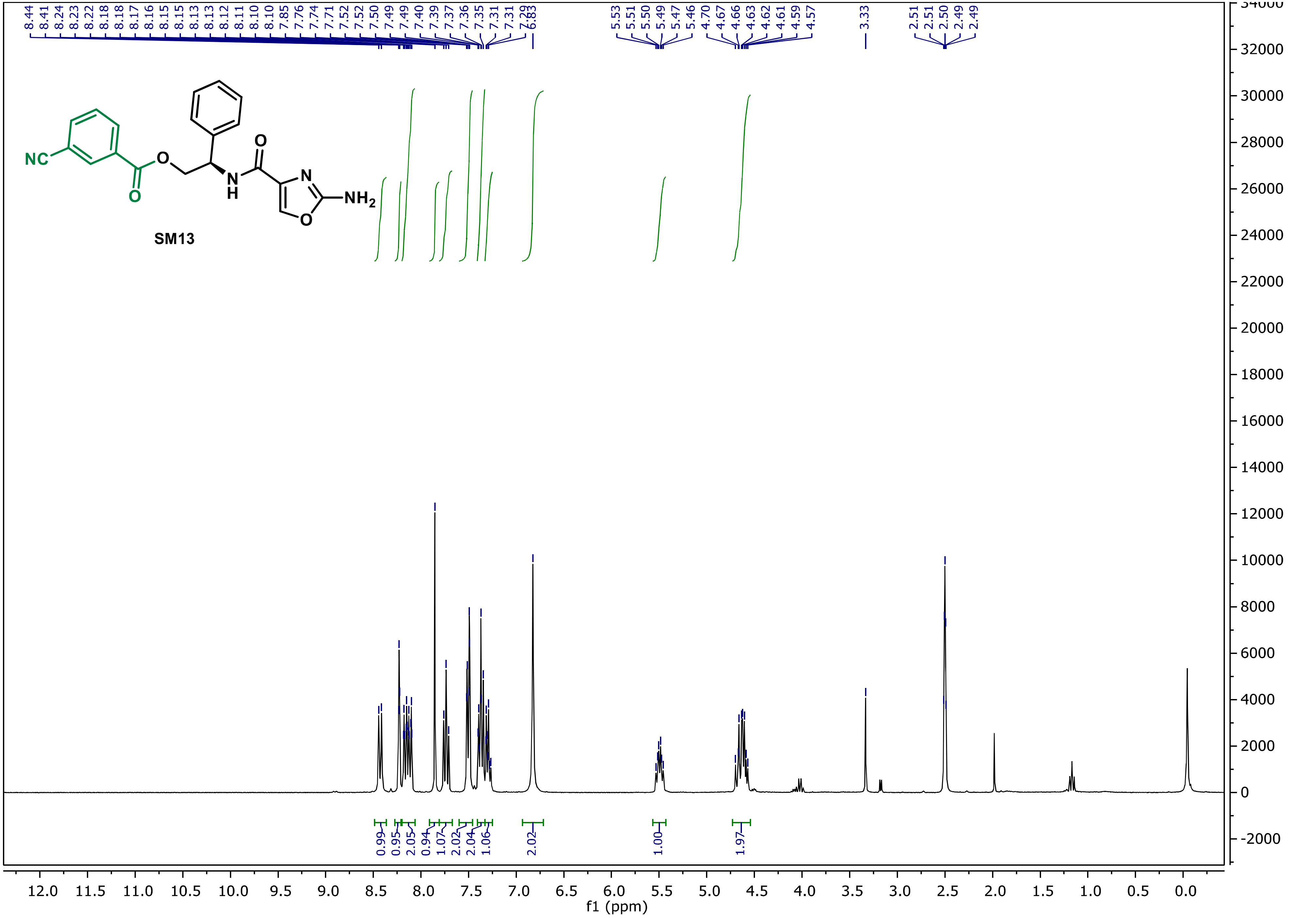

8.44 8.41 8.24 8.23 8.22 8.18 8.18 8.17 8.16 8.15 8.15 8.13 8.13 8.12 8.11 8.10 8.10 7.85 7.76 7.74 7.71 7.52 7.52 7.50 7.49 7.49 7.40 7.39 7.37 7.36 7.35 7.31 7.31 7.29 6.83
5.53 5.51 5.50 5.49 5.47 5.46 4.70 4.67 4.66 4.63 4.62 4.61 4.59 4.57
3.33
2.51 2.51 2.50 2.49 2.49
NC
O
O
N
H
O
N
O
NH2
0.99 0.95 2.05 0.94 1.07 2.02 2.04 1.06 2.02 1.00 1.97
12.0 11.5 11.0 10.5 10.0 9.5 9.0 8.5 8.0 7.5 7.0 6.5 6.0 5.5 5.0 4.5 4.0 3.5 3.0 2.5 2.0 1.5 1.0 0.5 0.0
f1 (ppm)
34000 32000 30000 28000 26000 24000 22000 20000 18000 16000 14000 12000 10000 8000 6000 4000 2000 0 -2000

SM13

^{13}C NMR

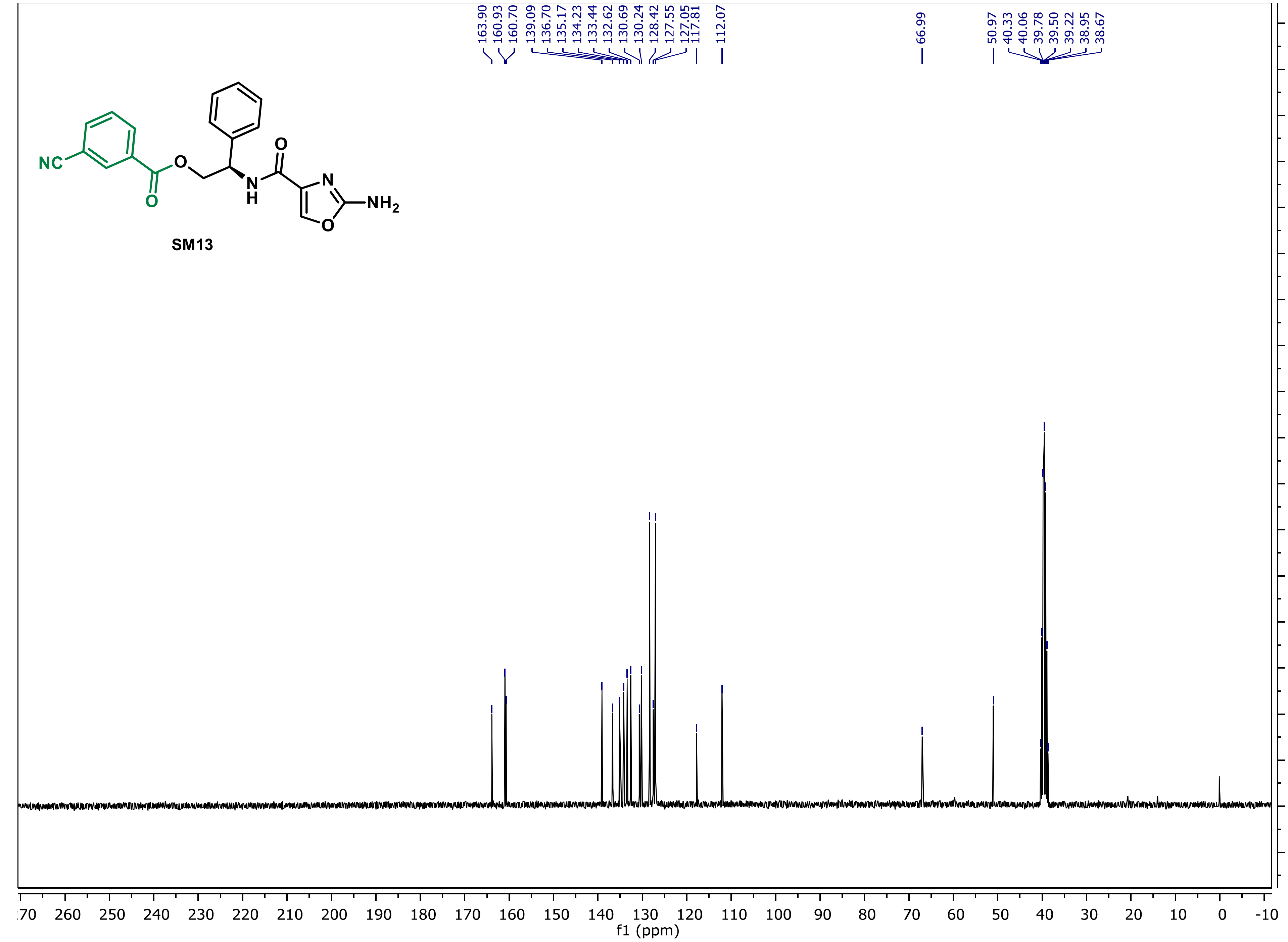

163.90
160.93
160.70
139.09
136.70
135.17
134.23
133.44
132.62
130.69
130.24
128.42
127.55
127.05
117.81
112.07
66.99
50.97
40.33
40.06
39.78
39.50
39.22
38.95
38.67
NC
O
N
H
NH2
SM13
f1 (ppm)

Mass to be matched (m/z): 399.106750 Charge: 1

Mass Tolerance: ±0.005000
Restriction of atom numbers:

C	H	N	O	Na
1-100	1-100	2-4	max 10	1-1

Number of calculated Formulas: 3

Formula	Diff.(ppm)	theor. m/z
C20 H16 N4 O4 Na1	-0.94	399.106374
C17 H18 N3 O7 Na1	-7.65	399.103695
C25 H16 N2 O2 Na1	9.14	399.110396

Datum	6.10.2020
Analyse:	149718b-00
Sigel:	GHC-AA-049-01
COP: Dr. Clement Ghiazza	
Messung:	HRMS
Methode:	ESIpos
Lösungsmittel:	CH3OH
Spektrometer:	Exactive
Auswerter:	Kampen (2242)

Suggestion:
C20H16N4O4 MW 376

characteristical ion
399 = [376 + Na]+

¹H NMR

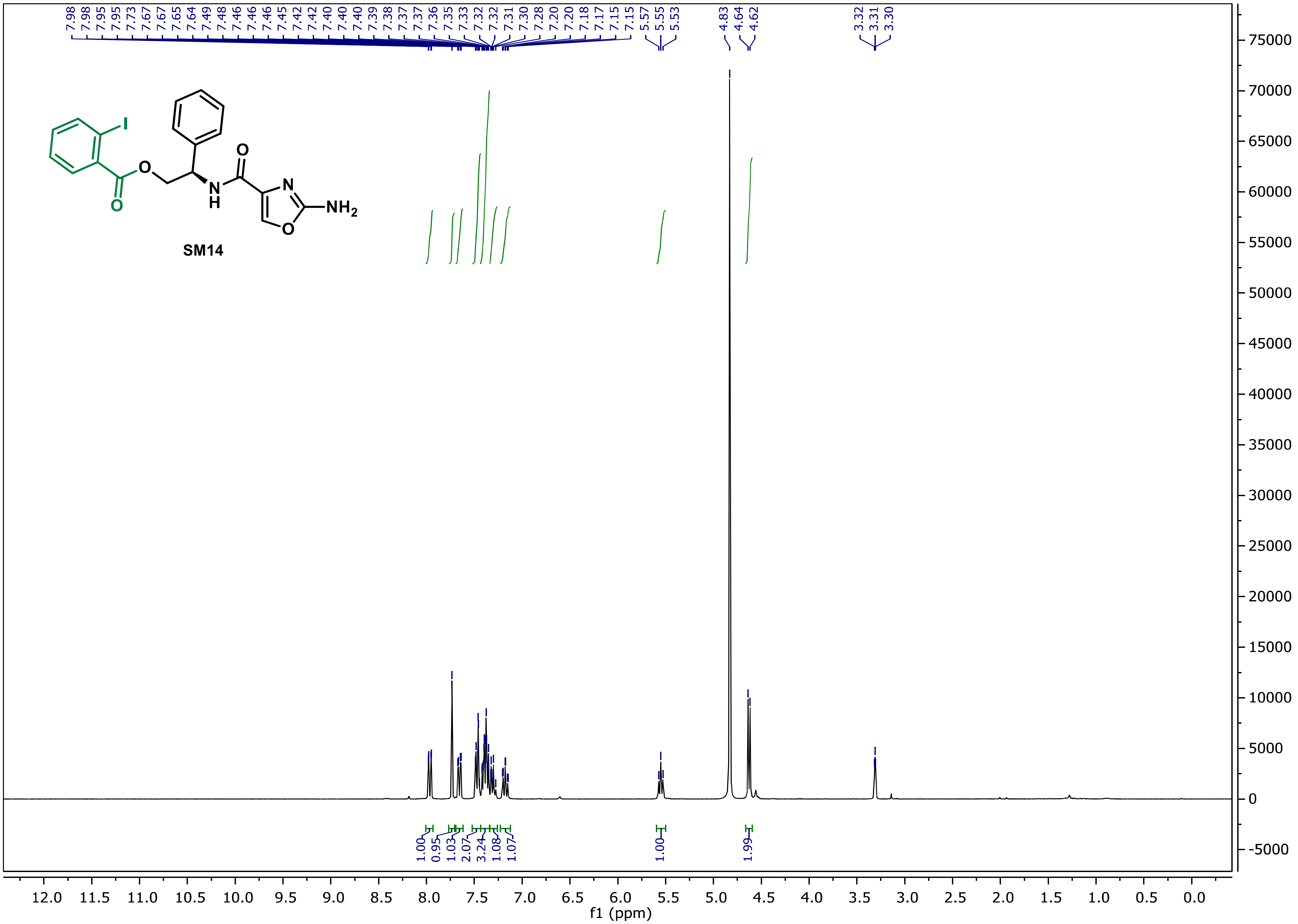
7.98
7.98
7.95
7.95
7.73
7.67
7.67
7.65
7.64
7.49
7.48
7.46
7.46
7.46
7.45
7.42
7.42
7.40
7.40
7.40
7.39
7.38
7.37
7.37
7.36
7.35
7.33
7.32
7.32
7.31
7.30
7.28
7.20
7.20
7.18
7.17
7.15
7.15
5.57
5.55
5.53
4.83
4.64
4.62
3.32
3.31
3.30
I
O
O
N
H
O
N
NH2
O
SM14
1.00
0.95
1.03
2.07
3.24
1.08
1.07
1.00
1.99
f1 (ppm)

^{13}C NMR

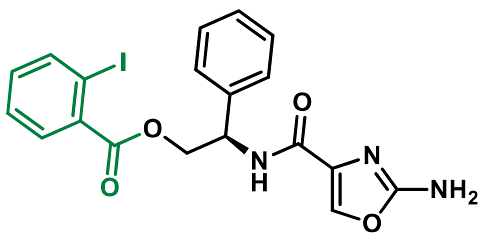

I
O
O
N
H
O
N
NH2
O

SM14

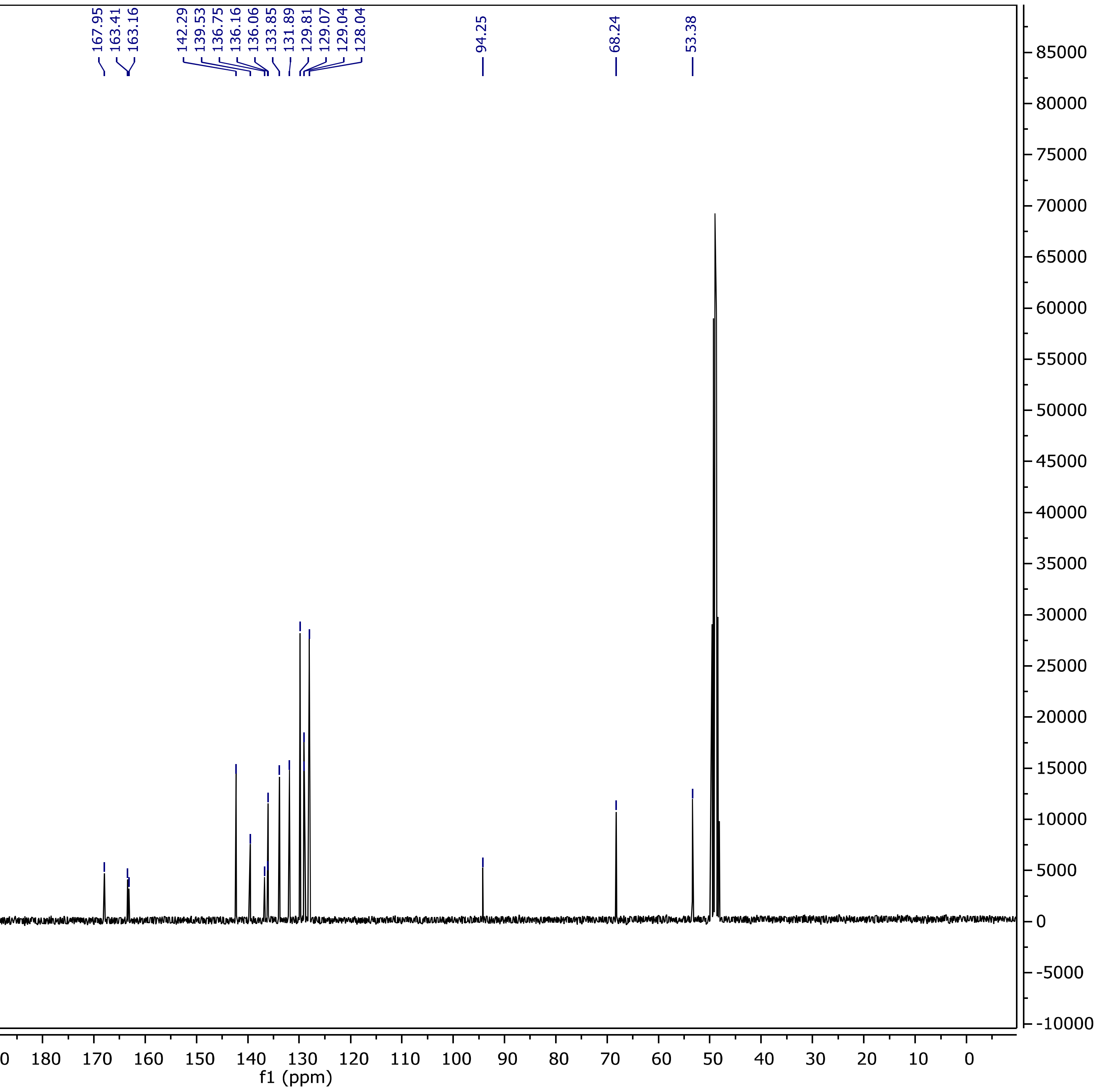

167.95
163.41
163.16
142.29
139.53
136.75
136.16
136.06
133.85
131.89
129.81
129.07
129.04
128.04
94.25
68.24
53.38
85000
80000
75000
70000
65000
60000
55000
50000
45000
40000
35000
30000
25000
20000
15000
10000
5000
0
-5000
-10000
270
260
250
240
230
220
210
200
190
180
170
160
150
140
130
120
110
100
90
80
70
60
50
40
30
20
10
0
f1 (ppm)

Mass to be matched (m/z): 500.007860 Charge: 1

Mass Tolerance: ±0.005000
Restriction of atom numbers:

C	H	I	N	O	Na
1-100	1-100	max 1	max 5	max 5	1-1

Number of calculated Formulas: 9

Formula	Diff.(ppm)	theor. m/z
C19 H16 I1 N3 O4 Na1	-0.18	500.007772
C34 H5 O4 Na1	0.29	500.008004
C32 H3 N3 O3 Na1	-2.40	500.006660
C21 H18 I1 O5 Na1	2.51	500.009116
C35 H1 N4 Na1	2.96	500.009339
C22 H14 I1 N4 O1 Na1	5.18	500.010451
C37 H3 N1 O1 Na1	5.65	500.010683
C24 H16 I1 N1 O2 Na1	7.87	500.011795
C28 H14 I1 Na1	-9.24	500.003241

Datum	5.10.2020
Analyse:	149697b-00
Sigel:	GHC-AA-043-01
COP: Dr. Clement Ghiazza	
Messung:	HRMS
Methode:	ESIpos
Lösungsmittel:	CH3OH
Spektrometer:	Exactive
Auswerter:	Kampen (2242)

Assumed suggestion based on the reaction of previous samples: C19H16I1N3O4 MW 477

characteristical ion
500 = [477 + Na]+

^{1}H NMR

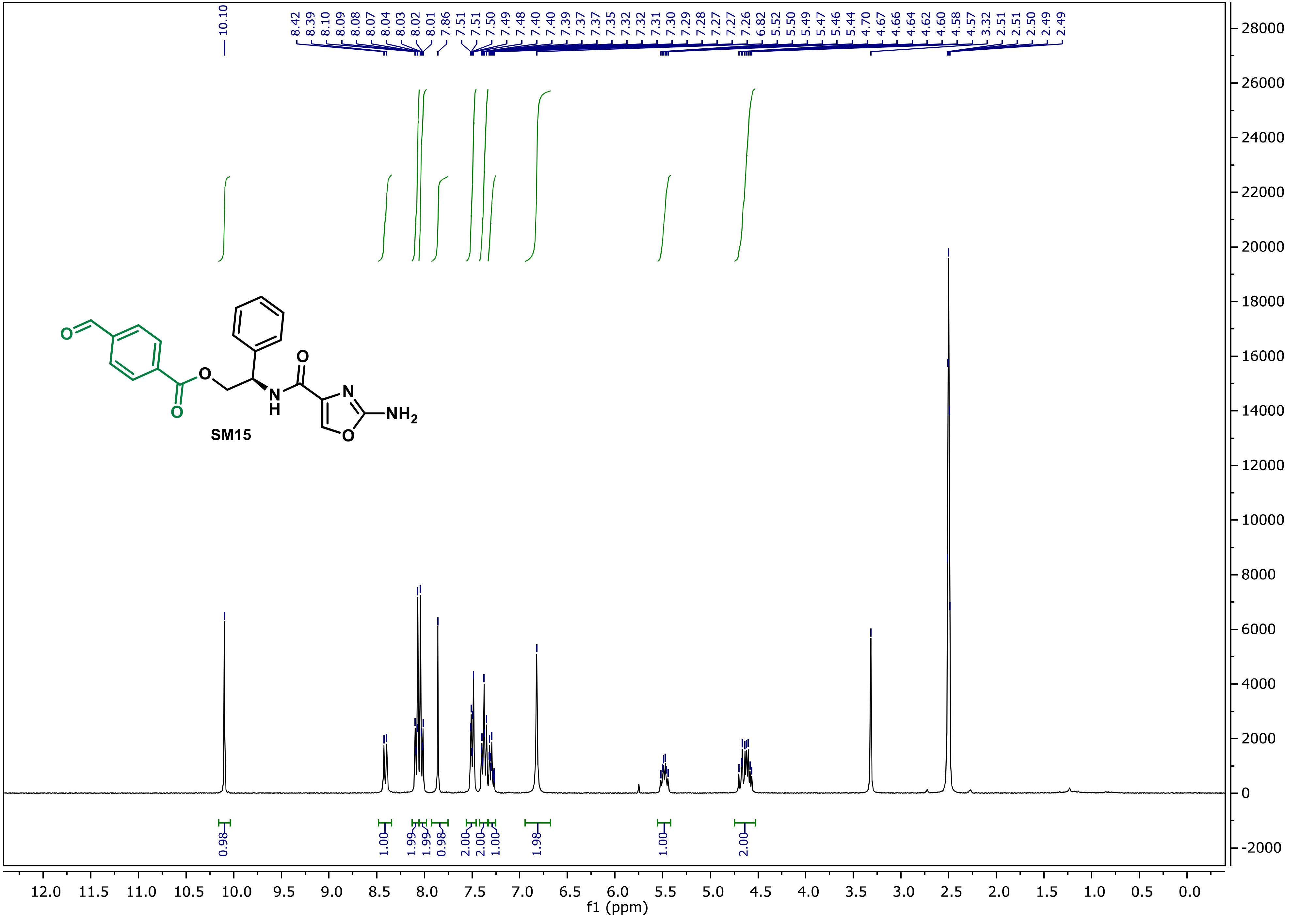

SM15
NH2
10.10
8.42 8.39 8.10 8.09 8.08 8.07 8.04 8.03 8.02 8.01 7.86 7.51 7.51 7.50 7.49 7.48 7.40 7.40 7.39 7.37 7.37 7.35 7.32 7.32 7.31 7.30 7.29 7.28 7.27 7.27 7.26 6.82 5.52 5.50 5.49 5.47 5.46 5.44 4.70 4.67 4.66 4.64 4.62 4.60 4.58 4.57 3.32 2.51 2.51 2.50 2.49 2.49
0.98 1.00 1.99 1.99 0.98 2.00 2.00 1.00 1.98 1.00 2.00
f1 (ppm)

^{13}C NMR

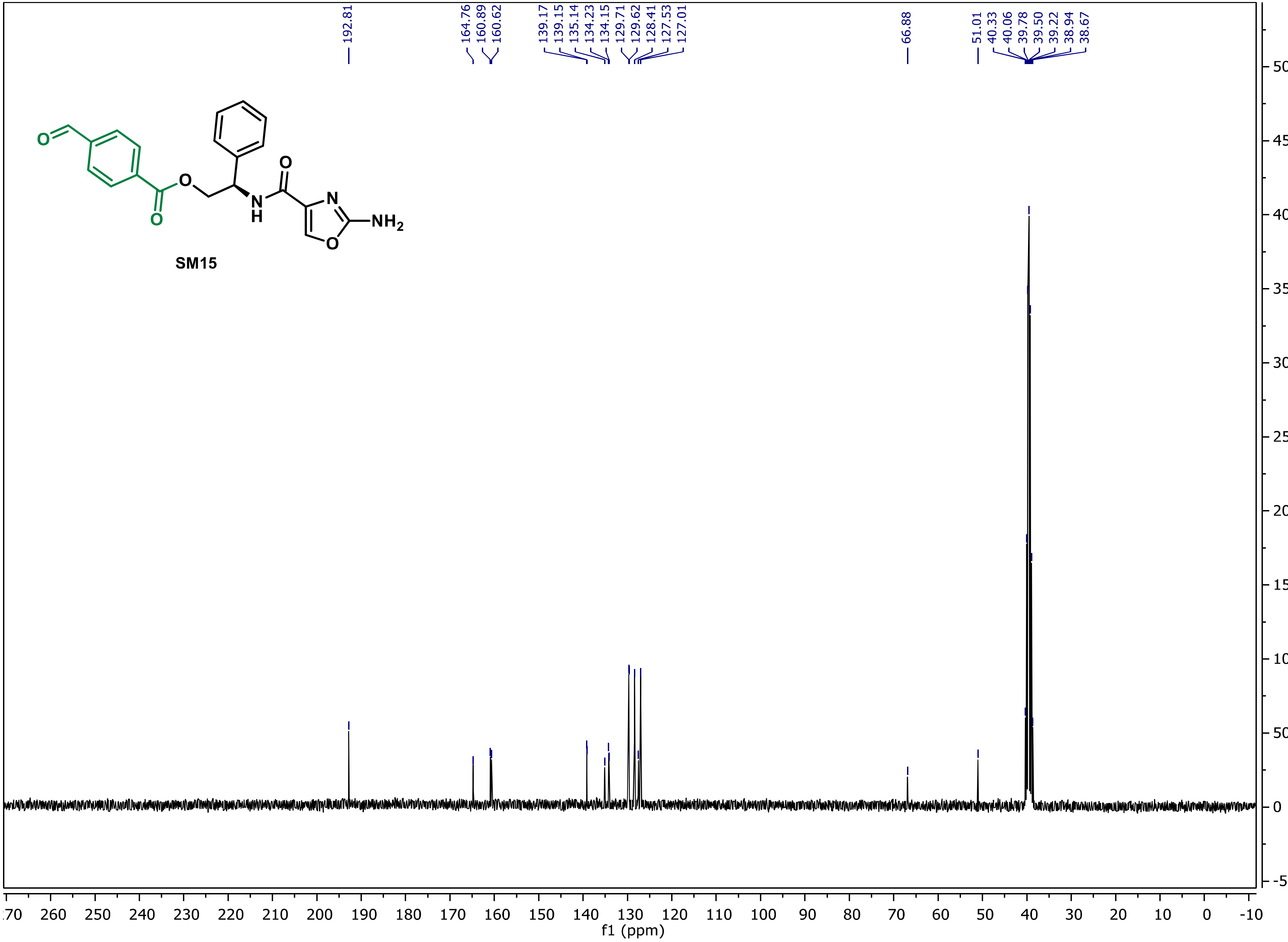
SM15
192.81
164.76
160.89
160.62
139.17
139.15
135.14
134.23
134.15
129.71
129.62
128.41
127.53
127.01
66.88
51.01
40.33
40.06
39.78
39.50
39.22
38.94
38.67
f1 (ppm)

*** Angegebene Mol.-Gewichte u. Massenzahlen basieren auf dem häufigsten Isotop der Elemente ***

MassLib

Mass to be matched (m/z): 402.106620 Charge: 1

Mass Tolerance: ±0.005000
Restriction of atom numbers:

C	H	N	O	Na
1-100	1-100	1-5	1-10	1-1

Number of calculated Formulas: 5

Formula	Diff.(ppm)	theor. m/z
C20 H17 N3 O5 Na1	-1.44	402.106040
C23 H15 N4 O2 Na1	5.22	402.108719
C17 H19 N2 O8 Na1	-8.10	402.103361
C25 H17 N1 O3 Na1	8.56	402.110063
C15 H17 N5 O7 Na1	-11.45	402.102018

Datum:	15.10.2020
Analyse:	149875c-00
Sigel:	GHC-AA-063-01<br>COP: Dr. Clement Ghiazza
Method:	HR-MS
Ionis. :	ESIpos
solvent :	CH3OH
Spectrometer:	Exactive
Auswerter:	Marcus, Tel:2243

suggestion:
C20H17N3O5 MW: 379

402 = [379 + Na]

^{1}H NMR

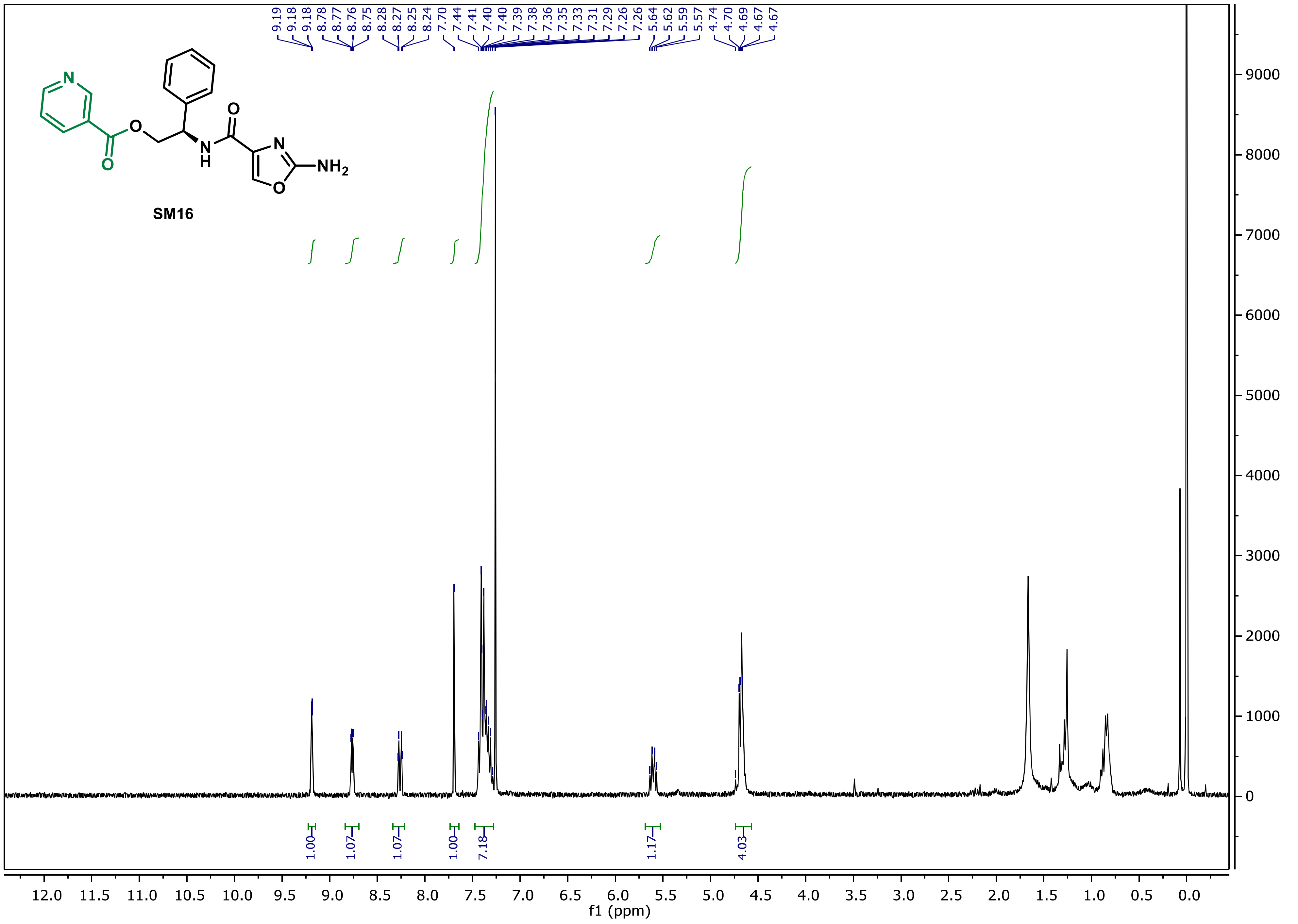

SM16
9.19 9.18 9.18 8.78 8.77 8.76 8.75 8.28 8.27 8.25 8.24 7.70 7.44 7.41 7.40 7.40 7.39 7.38 7.36 7.35 7.33 7.31 7.29 7.26 7.26 5.64 5.62 5.59 5.57 4.74 4.70 4.69 4.67 4.67
1.00 1.07 1.07 1.00 7.18 1.17 4.03
f1 (ppm)

^{13}C NMR

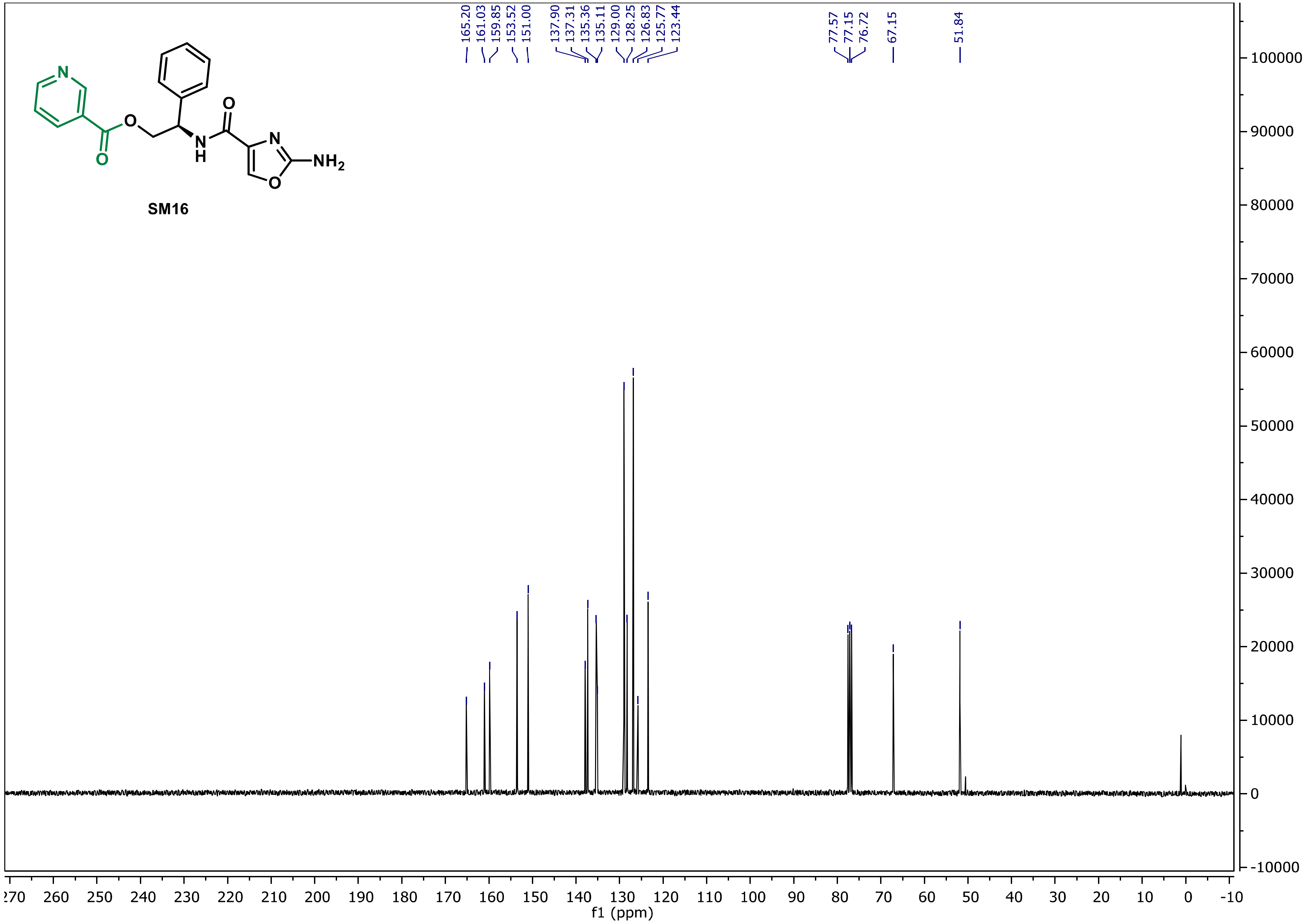
SM16
165.20
161.03
159.85
153.52
151.00
137.90
137.31
135.36
135.11
129.00
128.25
126.83
125.77
123.44
77.57
77.15
76.72
67.15
51.84
f1 (ppm)

Mass to be matched (m/z): 375.106510 Charge: 1

Mass Tolerance: ±0.005000
Restriction of atom numbers:

C	H	N	O	Na
1-100	1-100	1-5	1-10	1-1

Number of calculated Formulas: 5

Formula	Diff.(ppm)	theor. m/z
C18 H16 N4 O4 Na1	-0.36	375.106374
C20 H18 N1 O5 Na1	3.22	375.107718
C21 H14 N5 O1 Na1	6.78	375.109052
C15 H18 N3 O7 Na1	-7.50	375.103695
C23 H16 N2 O2 Na1	10.36	375.110396

Datum: 15.10.2020
Analyse: 149876c-00

Sigel: GHC-AA-064-01
COP: Dr. Clement Ghiazza

Method: HR-MS
Ionis. : ESIpos
solvent : CH3OH
Spectrometer: Exactive

Auswerter: Marcus, Tel:2243

suggestion:
C18H16N4O4   MW: 352

Characteristic Ions:
375 = [352 + Na]

¹H NMR

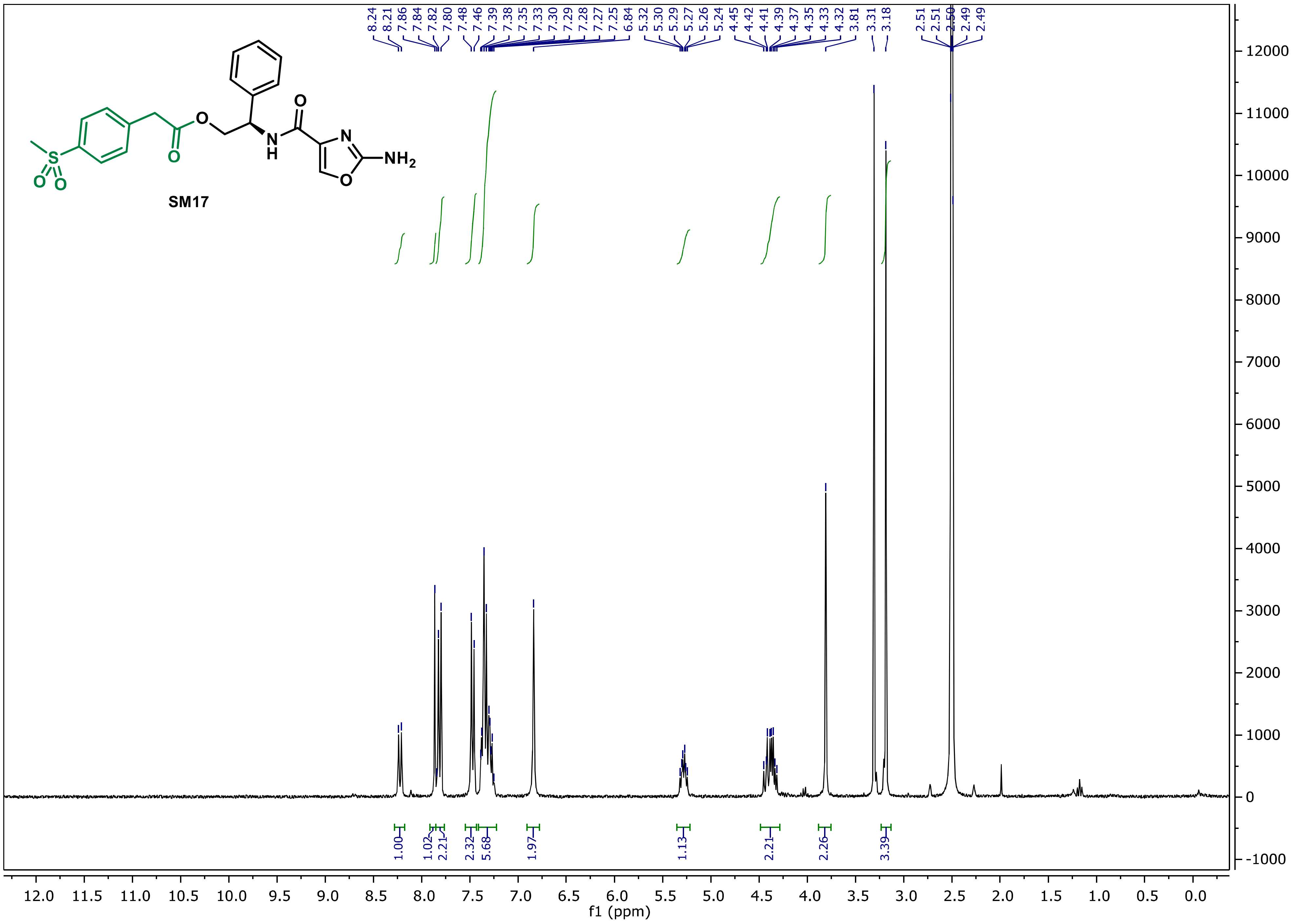
SM17
NH2
8.24
8.21
7.86
7.84
7.82
7.80
7.48
7.46
7.39
7.38
7.35
7.33
7.30
7.29
7.28
7.27
7.25
6.84
5.32
5.30
5.29
5.27
5.26
5.24
4.45
4.42
4.41
4.39
4.37
4.35
4.33
4.32
3.81
3.31
3.18
2.51
2.51
2.50
2.49
2.49
1.00
1.02
2.21
2.32
5.68
1.97
1.13
2.21
2.26
3.39
f1 (ppm)

^{13}C NMR

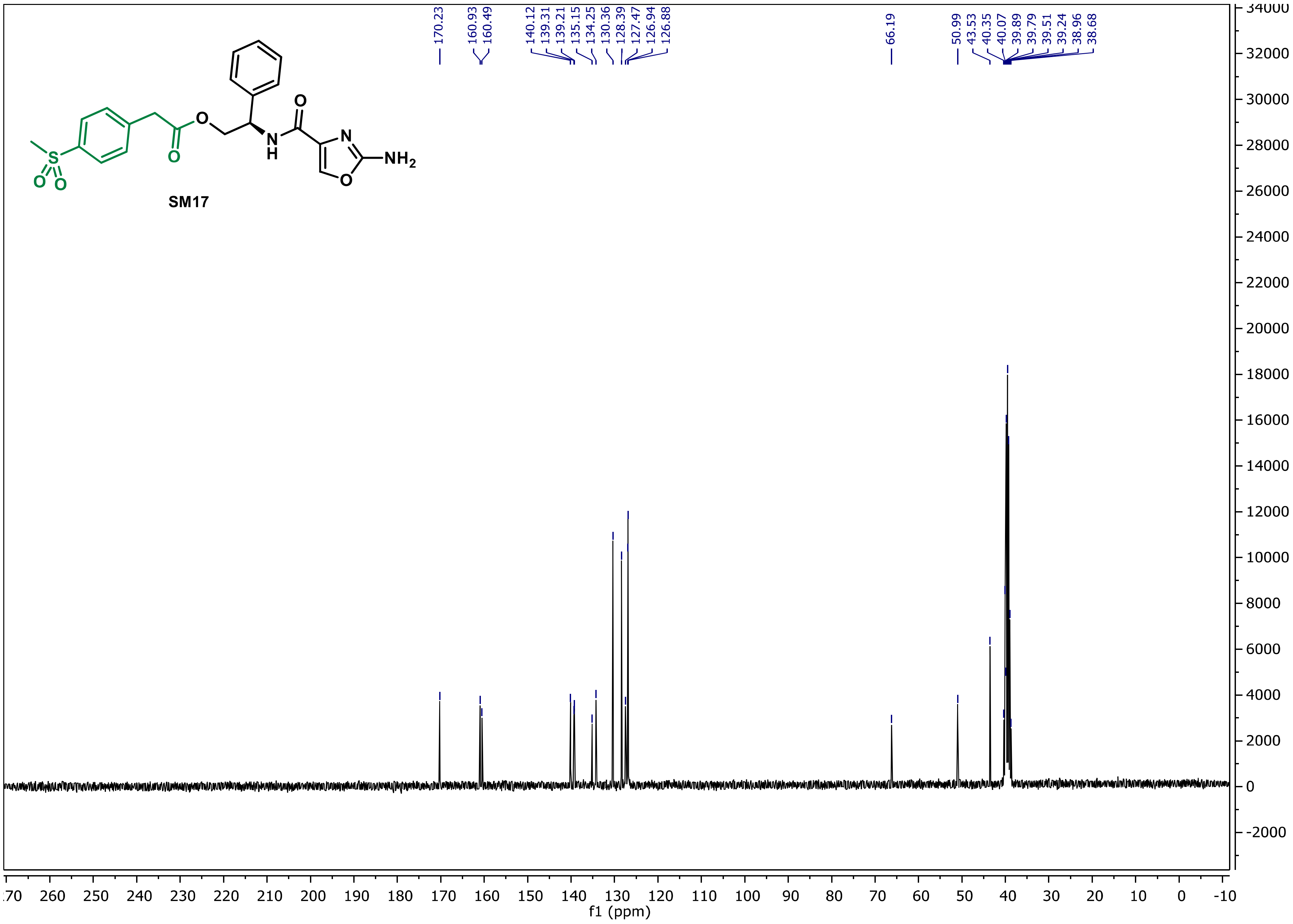
170.23
160.93
160.49
140.12
139.31
139.21
135.15
134.25
130.36
128.39
127.47
126.94
126.88
66.19
50.99
43.53
40.35
40.07
39.89
39.79
39.51
39.24
38.96
38.68
NH2
f1 (ppm)

SM17

Mass to be matched (m/z): 466.103980 Charge: 1

Mass Tolerance: ±0.005000
Restriction of atom numbers:

C	H	N	O	S	Na
1-110	1-100	1-3	1-6	1-1	1-1

Number of calculated Formulas: 2

Formula	Diff.(ppm)	theor. m/z
C21 H21 N3 O6 S1 Na1	0.75	466.104328
C26 H21 N1 O4 S1 Na1	9.38	466.108350

09.10.2020
File: 149779b-00

Analyse: GHC-AA-053-01
COP: Dr. Clement Ghiazza

Messung: HRMS ESIpos
Lösemittel: CH3OH
Spektrometer: Exactive

Auswerter: Kohler (2243)

Suggestion:
C21H21N3O6S1 MW 443

Characteristicial ions:
466 = [443 + Na]+

^{1}H NMR

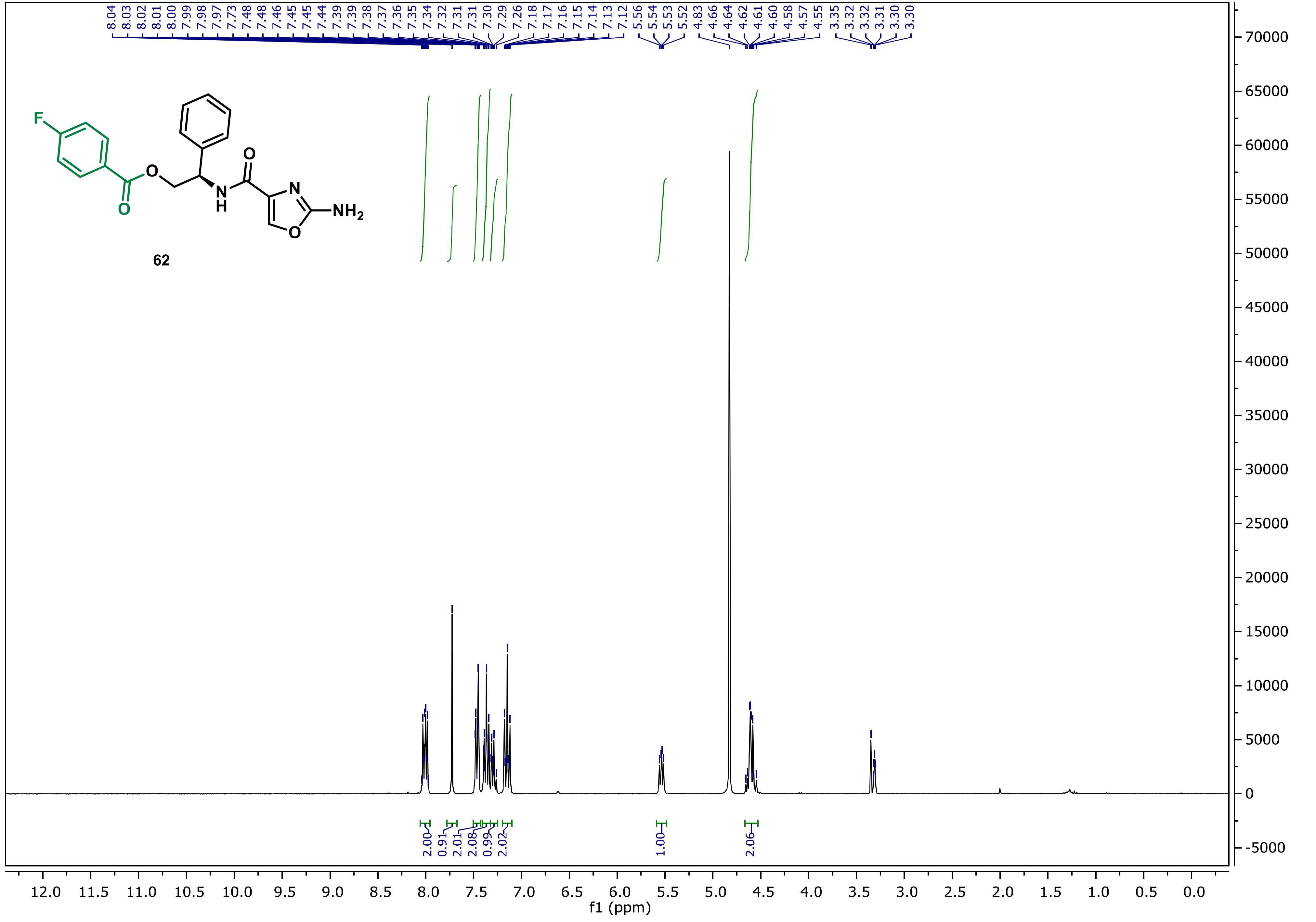
8.04 8.03 8.02 8.01 8.00 7.99 7.98 7.97 7.73 7.48 7.48 7.46 7.45 7.45 7.44 7.39 7.39 7.38 7.37 7.36 7.35 7.34 7.32 7.31 7.31 7.30 7.29 7.26 7.18 7.17 7.16 7.15 7.14 7.13 7.12 5.56 5.54 5.53 5.52 4.83 4.66 4.64 4.62 4.61 4.60 4.58 4.57 4.55 3.35 3.32 3.32 3.31 3.30 3.30
F
O
O
N
H
O
N
O
NH2
2.00
0.91
2.01
2.08
0.99
2.02
1.00
2.06
f1 (ppm)

62

^{13}C NMR

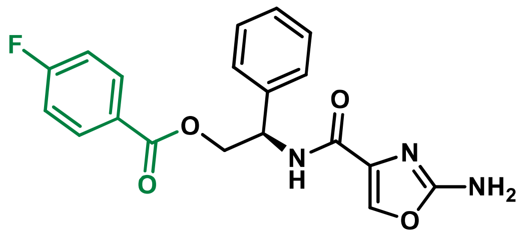

62

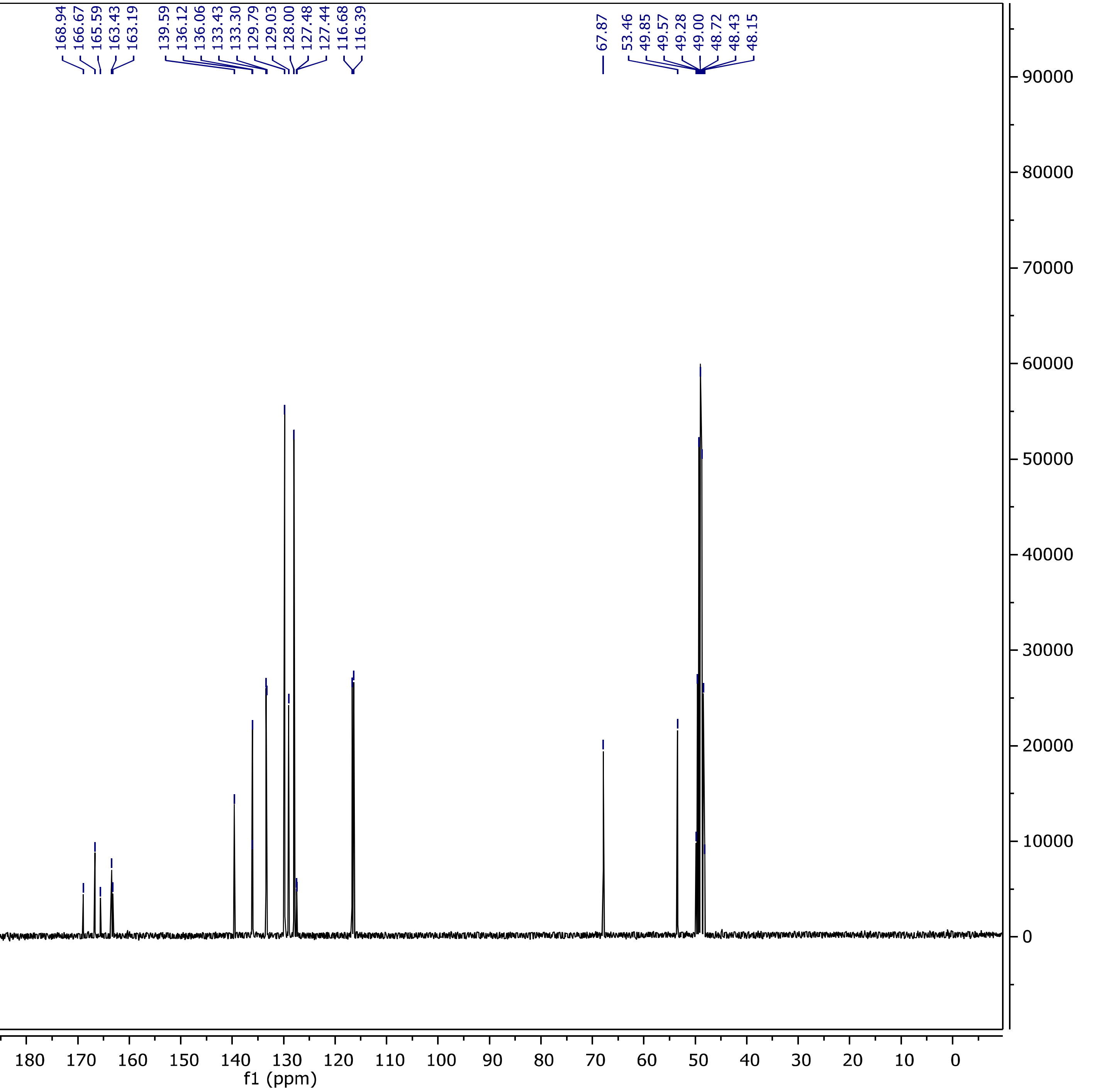
168.94
166.67
165.59
163.43
163.19
139.59
136.12
136.06
133.43
133.30
129.79
129.03
128.00
127.48
127.44
116.68
116.39
67.87
53.46
49.85
49.57
49.28
49.00
48.72
48.43
48.15
f1 (ppm)

^{19}F NMR

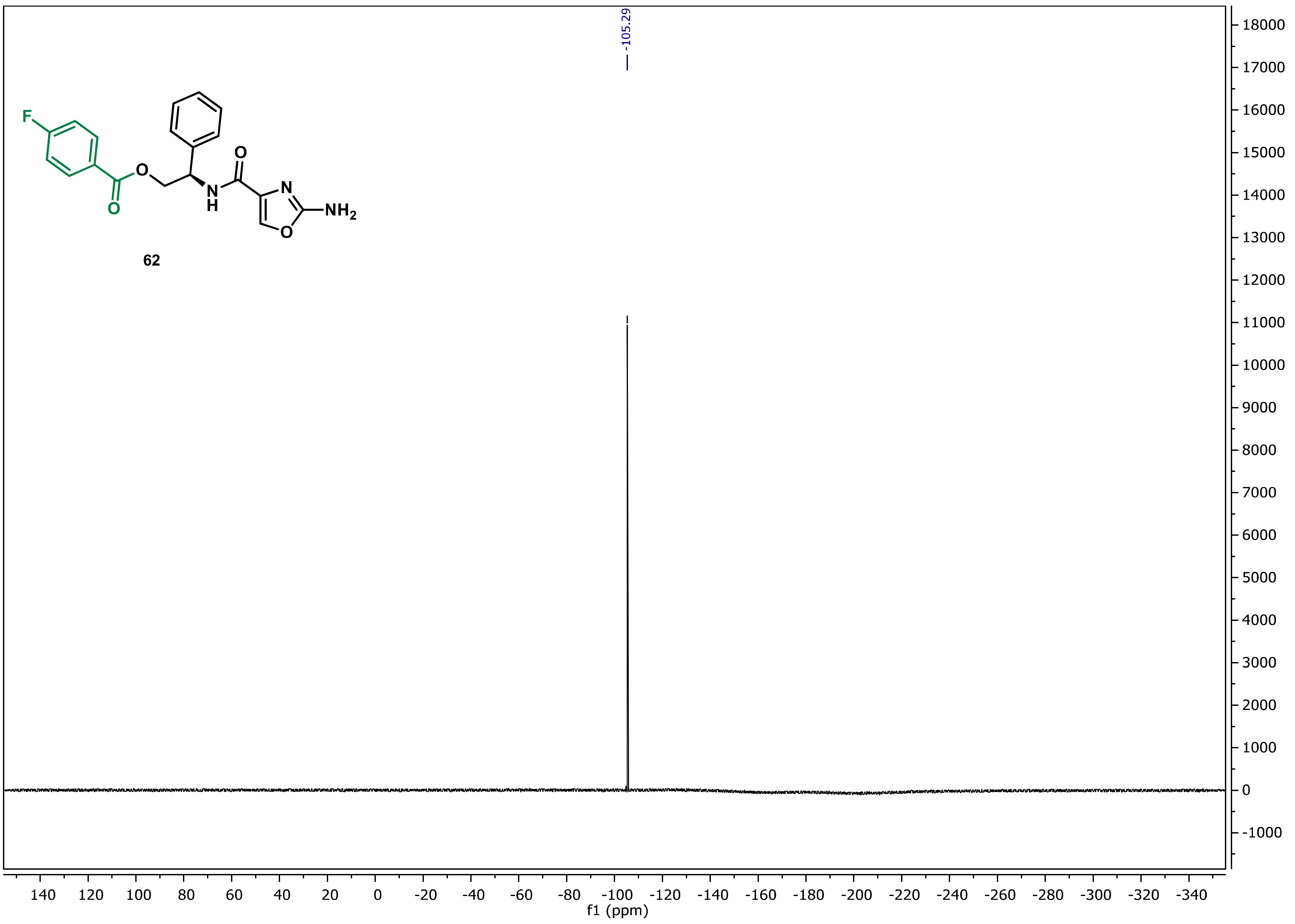

-105.29
f1 (ppm)
140
120
100
80
60
40
20
0
-20
-40
-60
-80
-100
-120
-140
-160
-180
-200
-220
-240
-260
-280
-300
-320
-340
18000
17000
16000
15000
14000
13000
12000
11000
10000
9000
8000
7000
6000
5000
4000
3000
2000
1000
0
-1000
F
O
N
H
N
NH2

62

Mass to be matched (m/z): 392.102070 Charge: 1

Mass Tolerance: ±0.050000
Restriction of atom numbers:

C	H	N	O	Na	F
1-100	1-100	1-3	1-4	1-1	1-1

Number of calculated Formulas: 13

Formula	Diff.(ppm)	theor. m/z
C19 H16 N3 O4 Na1 F1	-0.93	392.101704
C24 H16 N1 O2 Na1 F1	9.33	392.105726
C23 H14 N2 O2 Na1 F1	-22.75	392.093150
C20 H18 N2 O4 Na1 F1	31.14	392.114280
C23 H16 N3 O1 Na1 F1	37.97	392.116959
C22 H12 N3 O2 Na1 F1	-54.82	392.080574
C21 H20 N1 O4 Na1 F1	63.21	392.126856
C24 H18 N2 O1 Na1 F1	70.05	392.129535
C23 H12 N1 O3 Na1 F1	-83.47	392.069341
C20 H20 N3 O3 Na1 F1	91.86	392.138088
C25 H20 N1 O1 Na1 F1	102.12	392.142111
C22 H10 N2 O3 Na1 F1	-115.54	392.056765
C21 H22 N2 O3 Na1 F1	123.93	392.150665

2.10.2020
File: 149636c-00
Analysis: GHC-AA-040-01
COP: Dr. Clement Ghiazza

Messung:	HR-MS
Ionisierung:	ESIpos
Lösungsmittel:	CH2Cl2 + CH3OH
Spektrometer:	Exactive
ELNA:	27611

Auswerter: Haupt (2243)

Suggestion:
C19H16N3O4F1 MW: 369

Characteristic ions:
392 = [369 + Na]+

^{1}H NMR

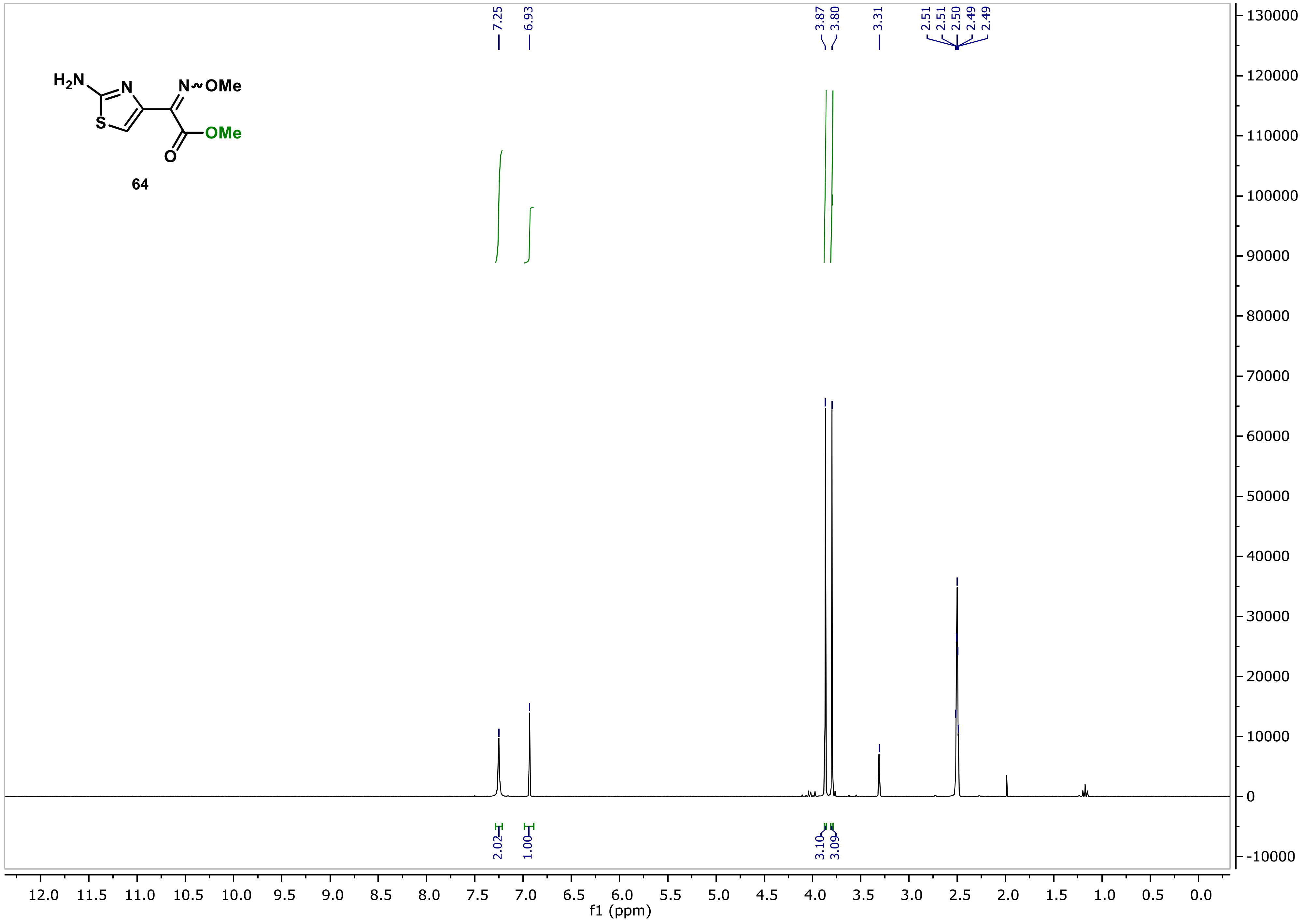

7.25
6.93
3.87
3.80
3.31
2.51
2.51
2.50
2.49
2.49
H2N
N
S
N~OMe
OMe
O
64
2.02
1.00
3.10
3.09
f1 (ppm)

^{1}H NMR

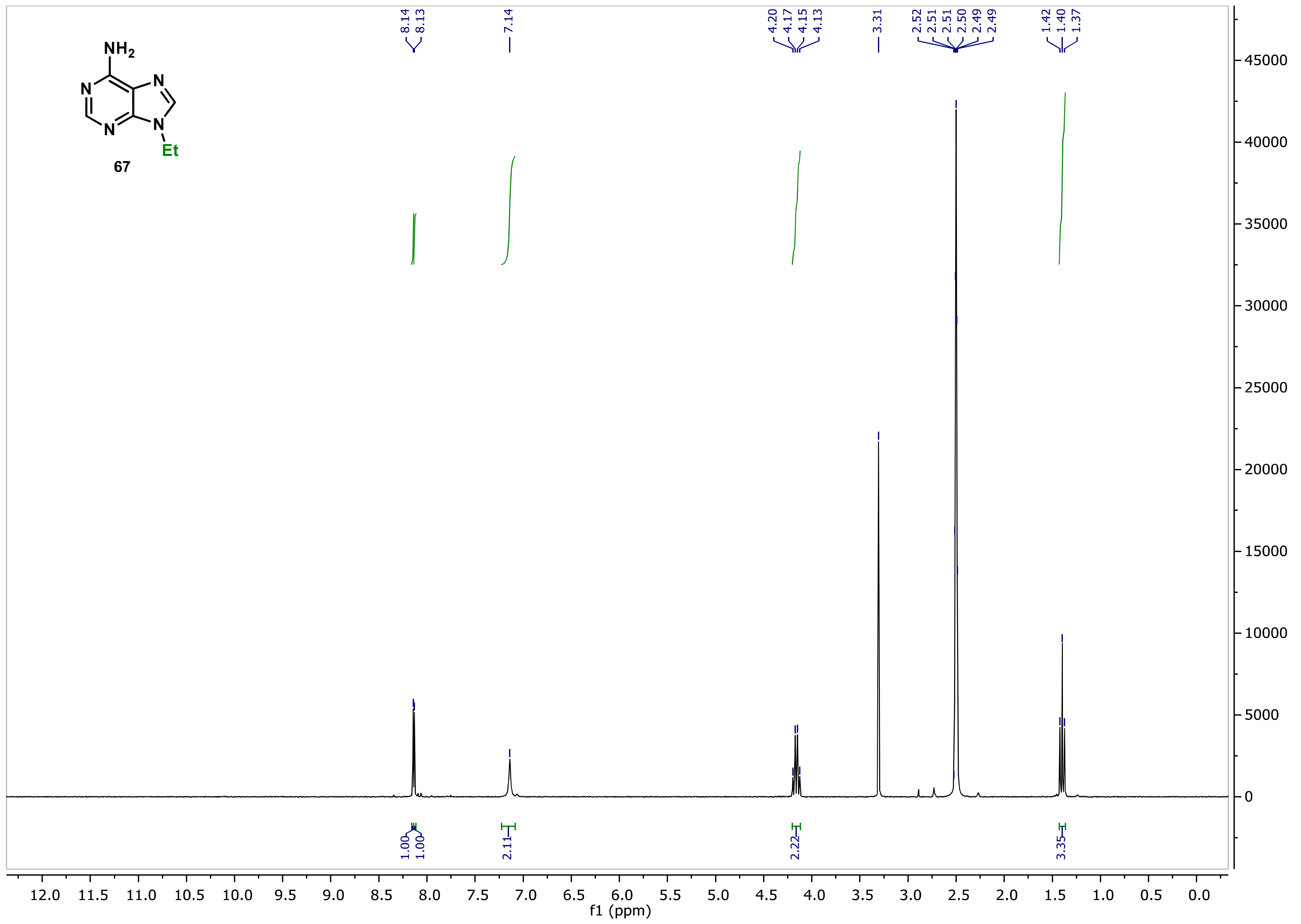

NH2
N
N
N
N
Et
67
8.14
8.13
7.14
4.20
4.17
4.15
4.13
3.31
2.52
2.51
2.51
2.50
2.49
2.49
1.42
1.40
1.37
1.00
1.00
2.11
2.22
3.35
45000
40000
35000
30000
25000
20000
15000
10000
5000
0
12.0
11.5
11.0
10.5
10.0
9.5
9.0
8.5
8.0
7.5
7.0
6.5
6.0
5.5
5.0
4.5
4.0
3.5
3.0
2.5
2.0
1.5
1.0
0.5
0.0
f1 (ppm)

^{1}H NMR

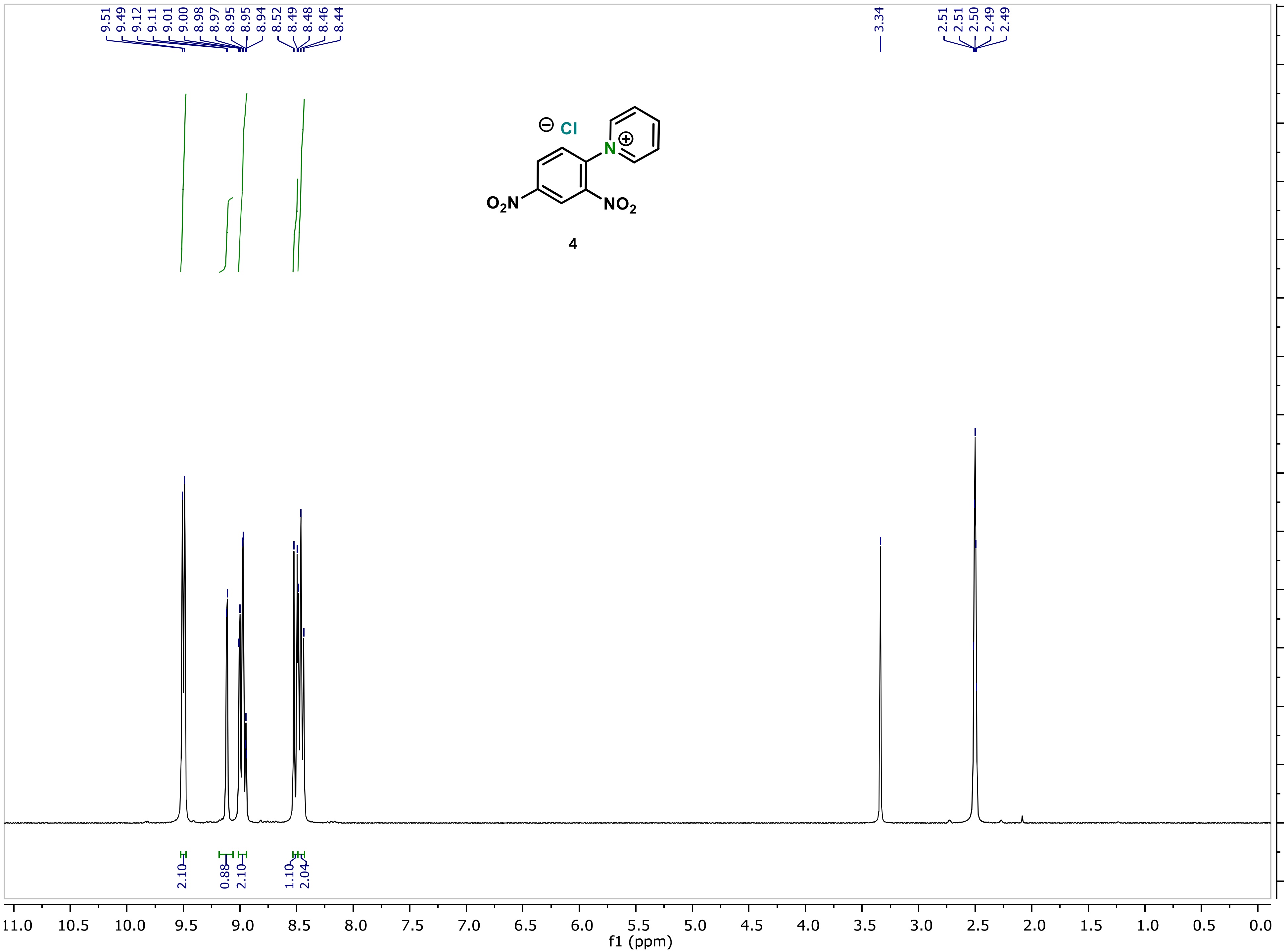
9.51
9.49
9.12
9.11
9.01
9.00
8.98
8.97
8.95
8.95
8.94
8.52
8.49
8.48
8.46
8.44
3.34
2.51
2.51
2.50
2.49
2.49
Cl
N
O2N
NO2
4
2.10
0.88
2.10
1.10
2.04
28000
26000
24000
22000
20000
18000
16000
14000
12000
10000
8000
6000
4000
2000
0
-2000
11.0
10.5
10.0
9.5
9.0
8.5
8.0
7.5
7.0
6.5
6.0
5.5
5.0
4.5
4.0
3.5
3.0
2.5
2.0
1.5
1.0
0.5
0.0
f1 (ppm)

^1H NMR

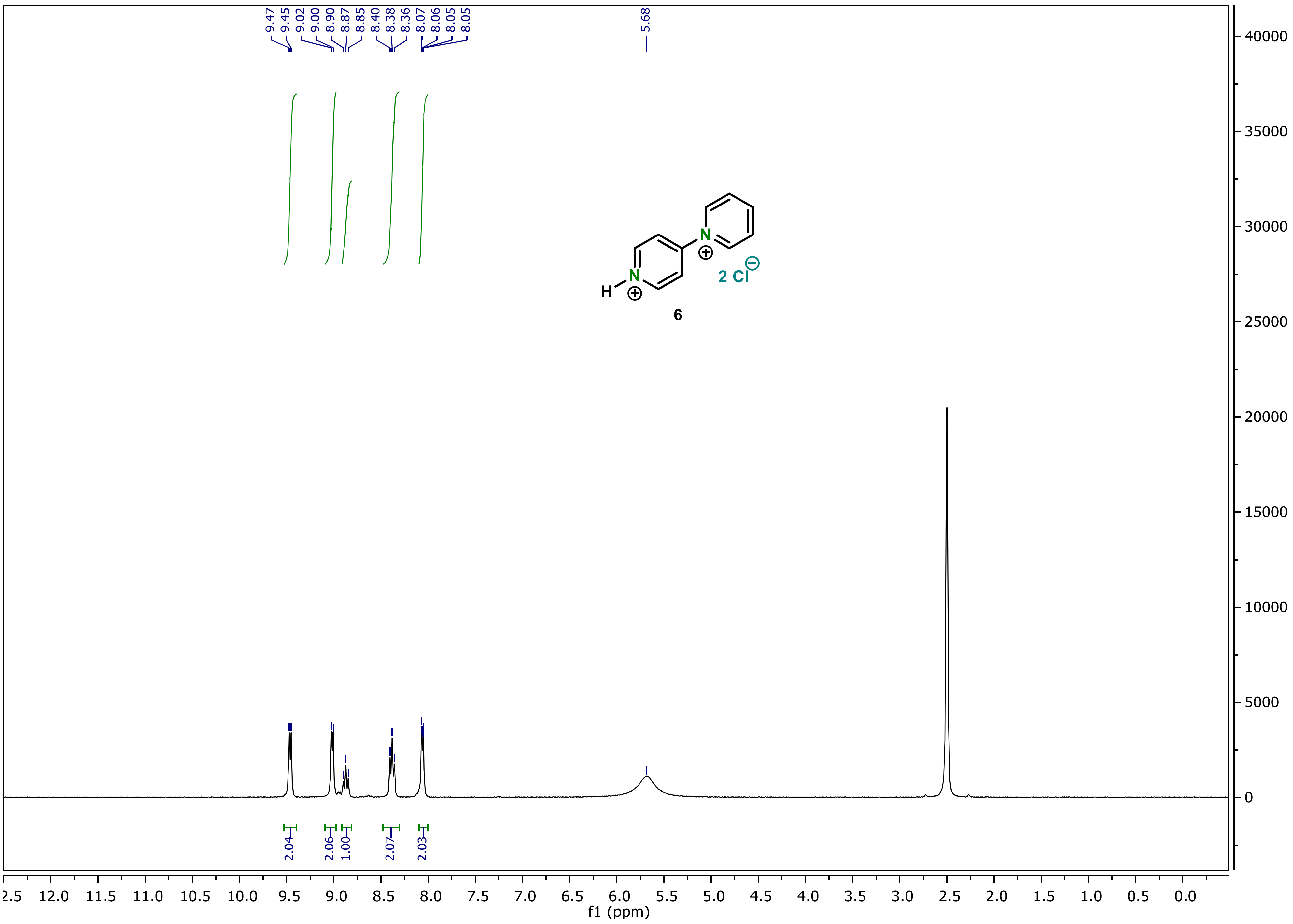

9.47
9.45
9.02
9.00
8.90
8.87
8.85
8.40
8.38
8.36
8.07
8.06
8.05
8.05
5.68
2 Cl
6
2.04
2.06
1.00
2.07
2.03
f1 (ppm)

^{1}H NMR – in situ

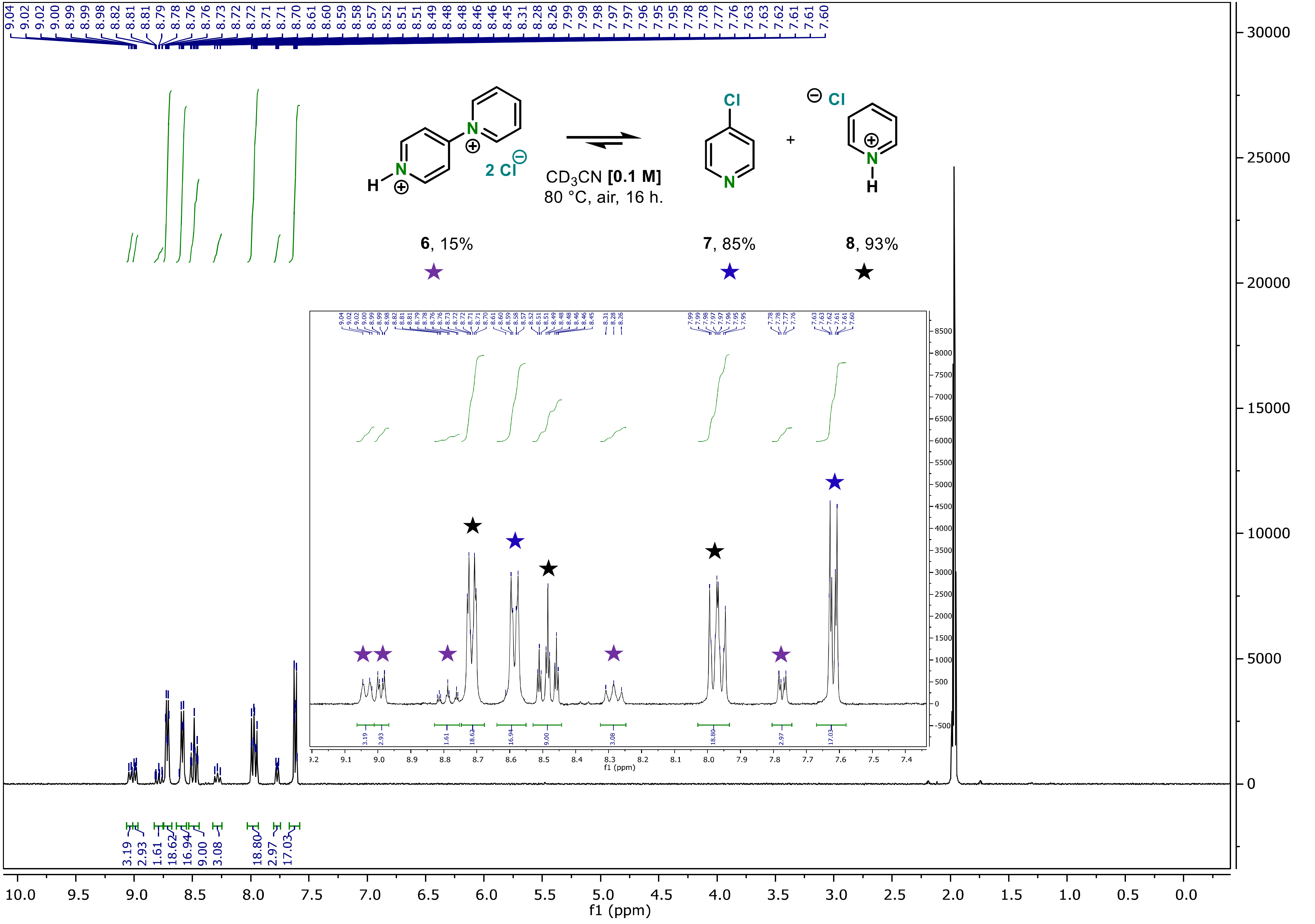

^{1}H NMR

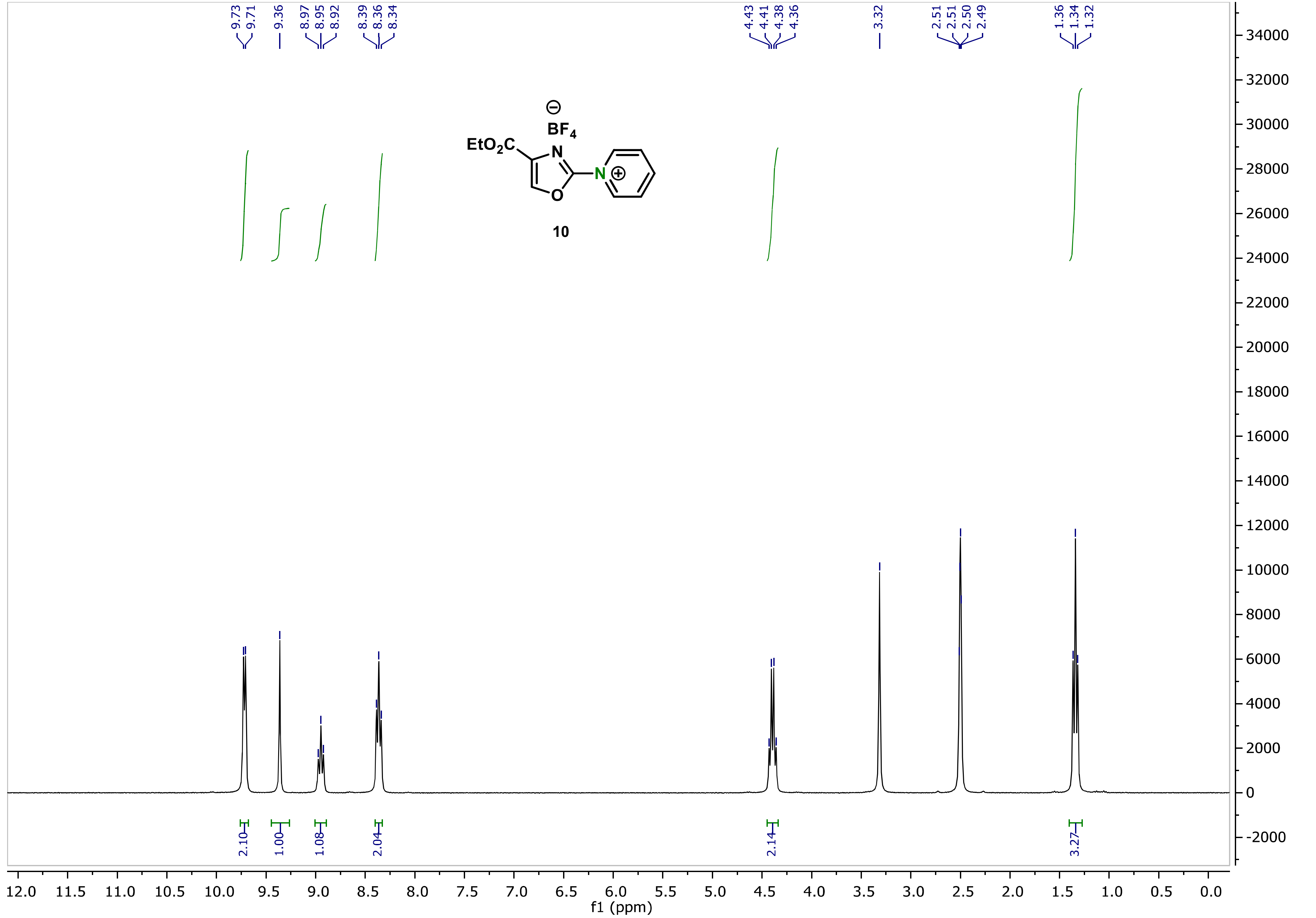

9.73
9.71
9.36
8.97
8.95
8.92
8.39
8.36
8.34
4.43
4.41
4.38
4.36
3.32
2.51
2.51
2.50
2.49
1.36
1.34
1.32
EtO2C
BF4
N
O
N
10
2.10
1.00
1.08
2.04
2.14
3.27
f1 (ppm)

^{13}C NMR

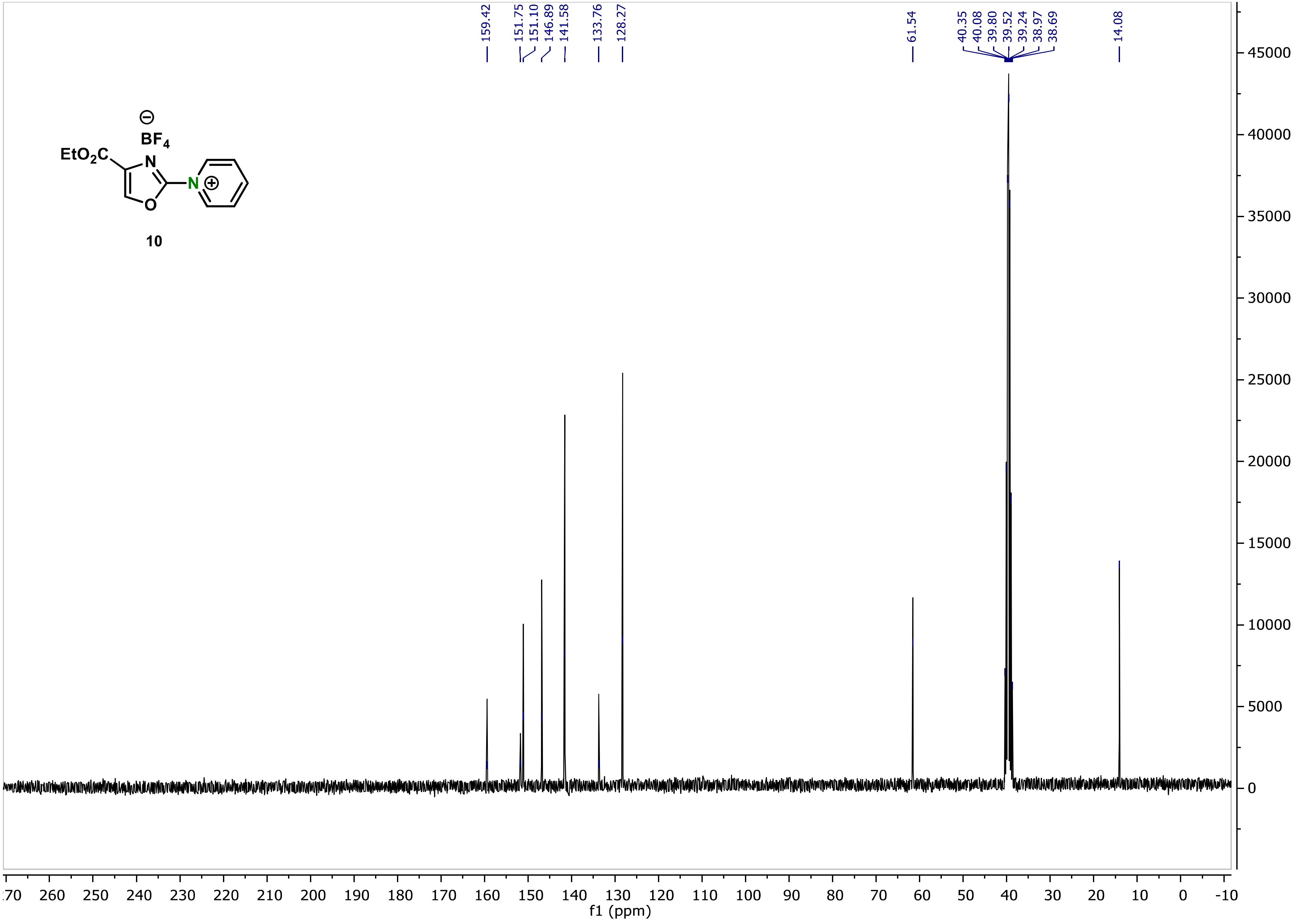

EtO2C
BF4
N
O
N
10
159.42
151.75
151.10
146.89
141.58
133.76
128.27
61.54
40.35
40.08
39.80
39.52
39.24
38.97
38.69
14.08
f1 (ppm)

^{19}F NMR

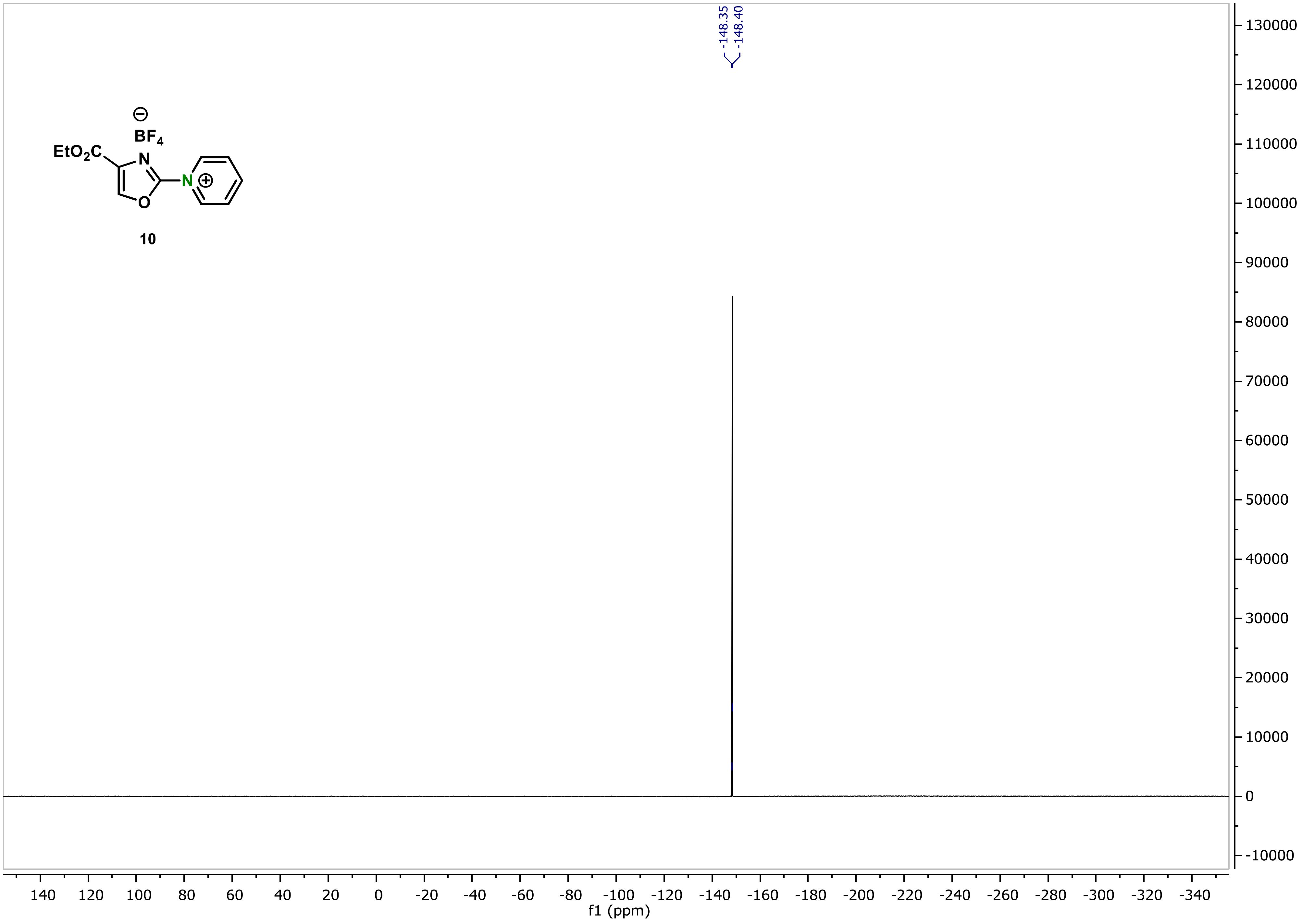

EtO2C
BF4
N
O
N
10
-148.35
-148.40
130000
120000
110000
100000
90000
80000
70000
60000
50000
40000
30000
20000
10000
0
-10000
140 120 100 80 60 40 20 0 -20 -40 -60 -80 -100 -120 -140 -160 -180 -200 -220 -240 -260 -280 -300 -320 -340
f1 (ppm)

Mass to be matched (m/z): 219.076560 Charge: 1

Mass Tolerance: ±0.050000
Restriction of atom numbers:

C	H	N	O
1-100	1-100	1-2	1-3

Number of calculated Formulas: 7

Formula	Diff.(ppm)	theor. m/z
C11 H11 N2 O3	-0.65	219.076417
C15 H9 N1 O1	-39.69	219.067864
C12 H13 N1 O3	56.75	219.088994
C14 H7 N2 O1	-97.10	219.055287
C12 H15 N2 O2	165.43	219.112802
C14 H5 N1 O2	-205.78	219.031479
C13 H17 N1 O2	222.84	219.125379

7.12.2020
File: 150859b-00
Analysis: GHC-GA-431-01
COP: Dr. Clement Ghiazza

Messung:	HR-MS
Ionisierung:	ESIpos
Lösungsmittel:	CH2Cl2
Spektrometer:	Exactive
ELNA:	28796
Auswerter:	Haupt (2243)

Suggestion:
[C11H11N2O3]+[BF4]- MW: 306

Characteristic ions:
219 = [306 - BF4]+

^{1}H NMR – in situ

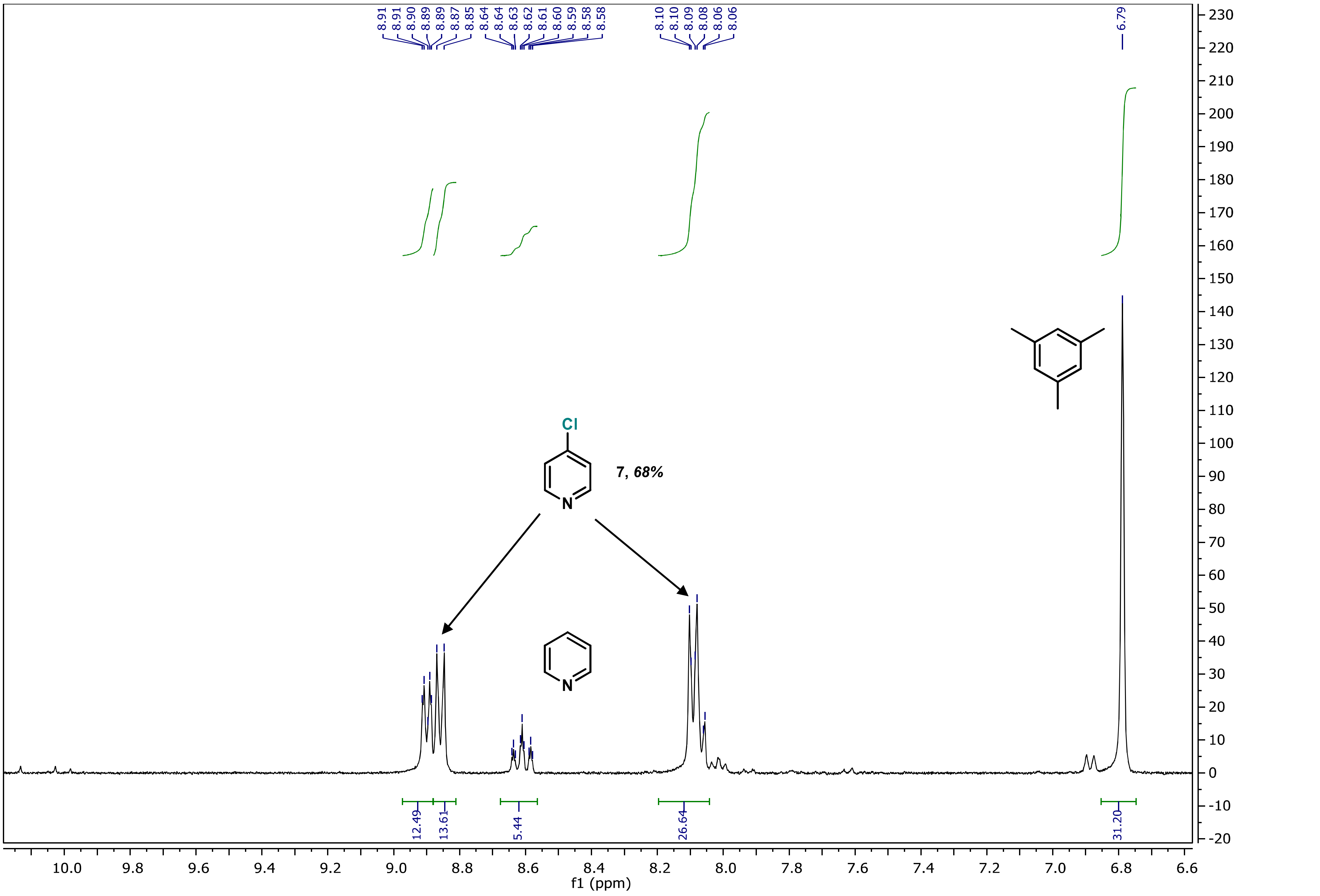

^1H NMR

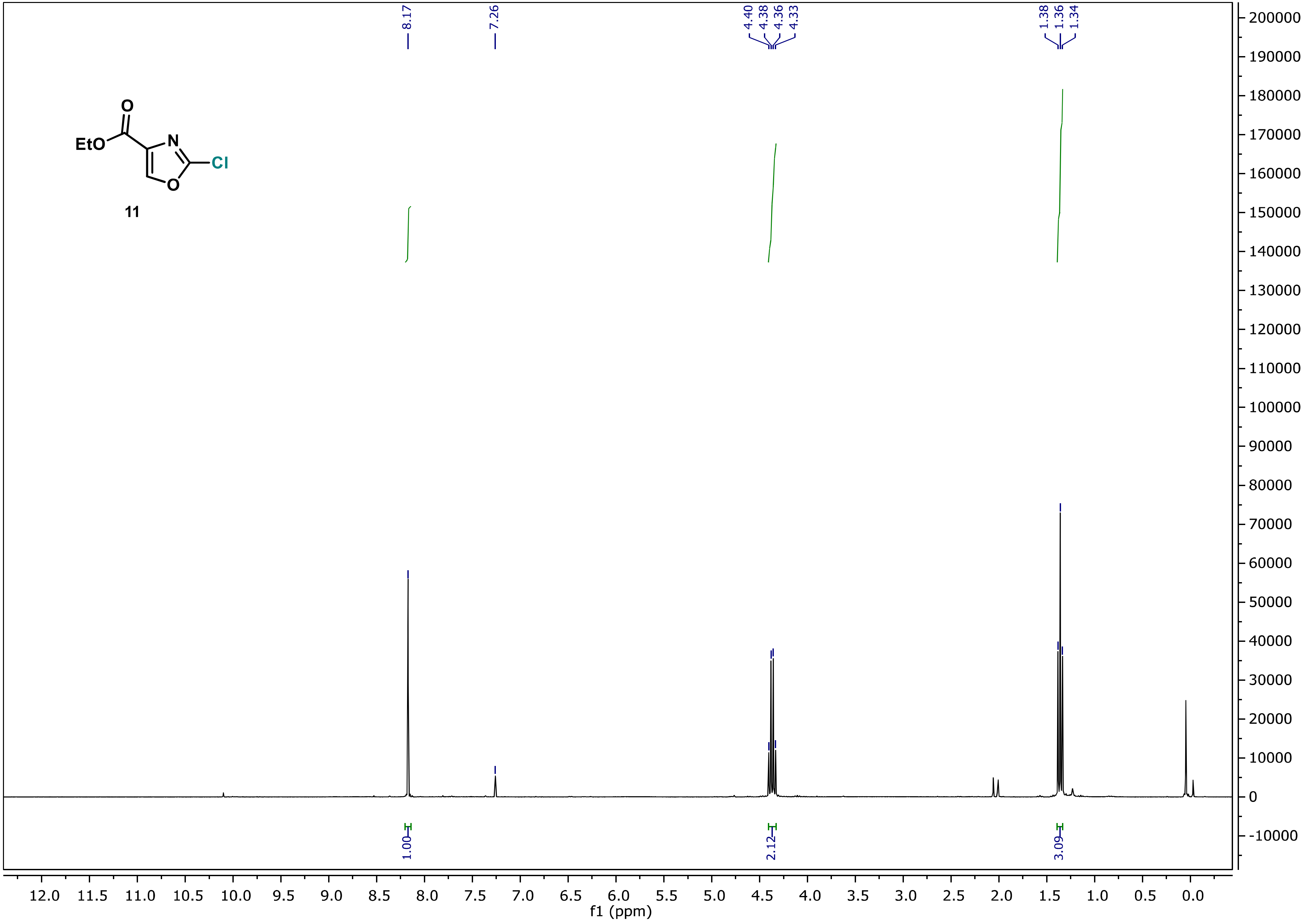

O
EtO
N
Cl
O
11
8.17
7.26
4.40
4.38
4.36
4.33
1.38
1.36
1.34
1.00
2.12
3.09
200000
190000
180000
170000
160000
150000
140000
130000
120000
110000
100000
90000
80000
70000
60000
50000
40000
30000
20000
10000
0
-10000
12.0 11.5 11.0 10.5 10.0 9.5 9.0 8.5 8.0 7.5 7.0 6.5 6.0 5.5 5.0 4.5 4.0 3.5 3.0 2.5 2.0 1.5 1.0 0.5 0.0
f1 (ppm)

^{13}C NMR

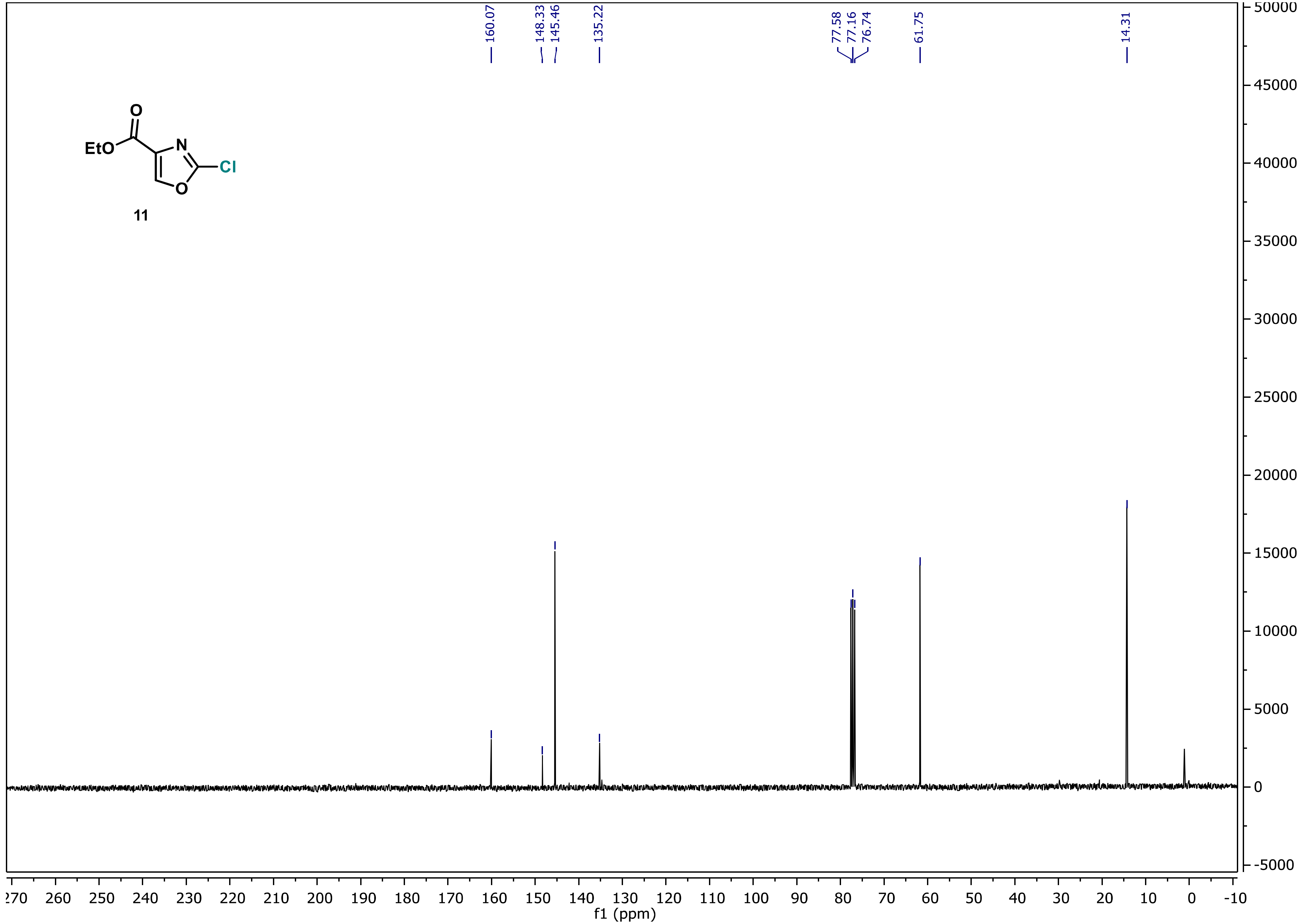

Mass to be matched (m/z): 175.003070 Charge: 1

Mass Tolerance: ±0.050000
Restriction of atom numbers:

C	H	N	O	Cl
1-100	1-100	1-2	1-3	1-2

Number of calculated Formulas: 12

Formula	Diff.(ppm)	theor. m/z
C6 H6 N1 O3 Cl1	0.01	175.003072
C3 H9 N2 O2 Cl2	2.79	175.003558
C5 H4 N2 O3 Cl1	-71.85	174.990495
C4 H11 N1 O2 Cl2	74.66	175.016135
C9 H2 N1 O1 Cl1	-120.73	174.981942
C3 H7 N1 O3 Cl2	-133.26	174.979750
C6 H8 N2 O2 Cl1	136.06	175.026880
C2 H5 N2 O3 Cl2	-205.12	174.967173
C7 H10 N1 O2 Cl1	207.92	175.039456
C4 H13 N2 O1 Cl2	210.70	175.039943
C6 H3 N1 O1 Cl2	-254.00	174.958620
C5 H15 N1 O1 Cl2	282.57	175.052520

Suggestion:
C6H6N1O3Cl1 MW:175

8.07.2020
File: 147978b-00.raw
Analyse: GHC-GA-190-01
COP: Dr. Clement Ghiazza

Messung:	GC-MS
Ionisierung:	GC-EI
Spektrometer:	Q Exactive GC Orbitrap
Säule:	MS 75 ZB-5HT 30+5
Länge:	30+5
Temp.:	35-10-285-5
GC-Nr.:	-
ELNA-Nr.:	25987

Auswerter: Haupt (2243)

^{1}H NMR – in situ

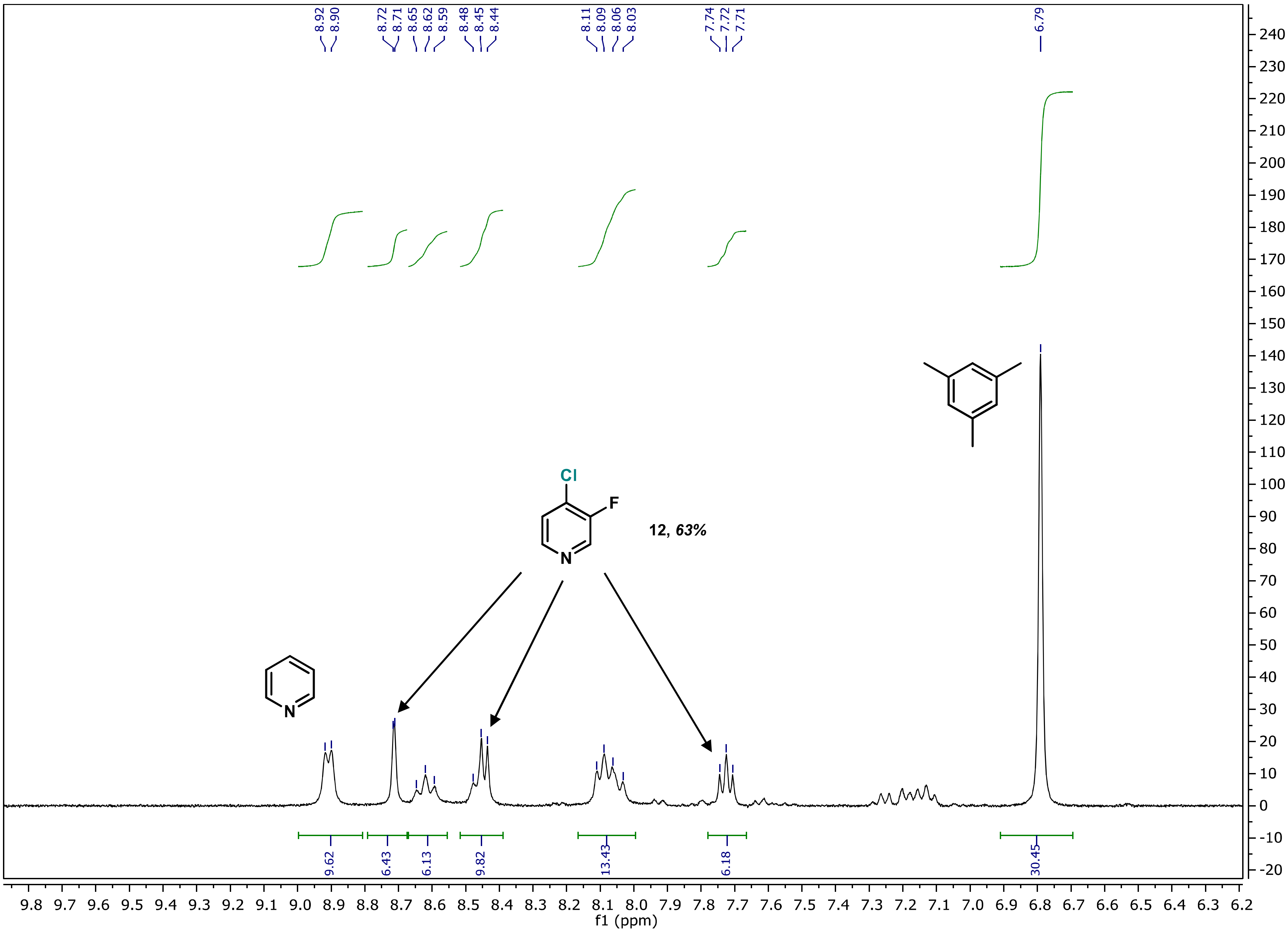

^{1}H NMR – in situ

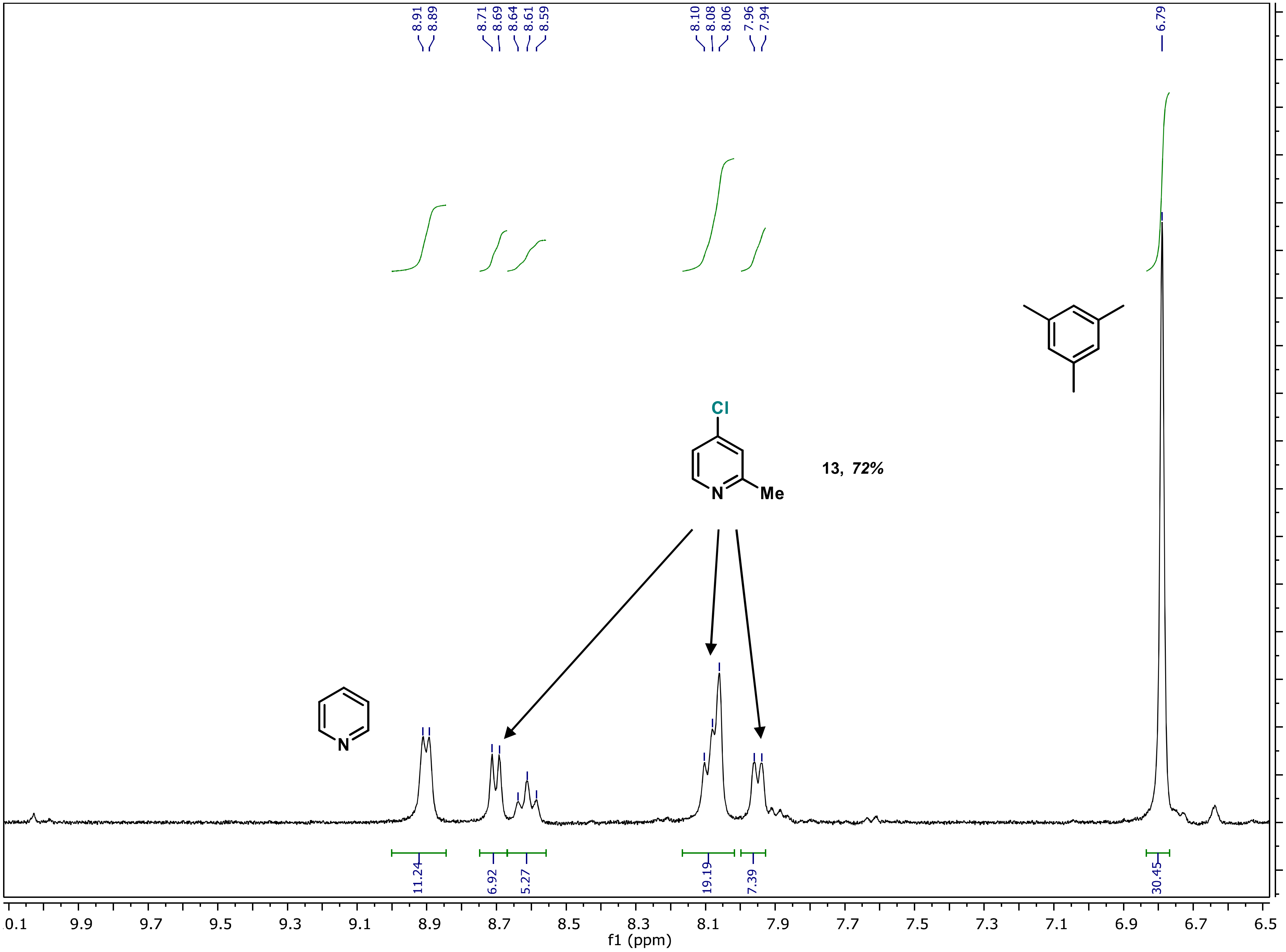

Mass to be matched (m/z): 128.026220 Charge: 1

Mass Tolerance: ±0.005000
Restriction of atom numbers:

C	H	Cl	N
1-100	1-100	1-1	1-3

Number of calculated Formulas: 1

Formula	Diff.(ppm)	theor. m/z
C6 H7 Cl1 N1	-0.53	128.026152

Datum	20.07.2020
Analyse:	148239c-00
Sigel:	GOA-GA-386-01
COP: Dr. Gomez, Alejandro	
Messung:	HRMS
Methode:	ESIpos
Lösungsmittel:	CH3CN
Spektrometer:	Exactive
Auswerter:	Kampen (2242)

Suggestion:
C6H6Cl1N1 MW 127

characteristical ion
128 = [127 + H]+

^{1}H NMR – in situ

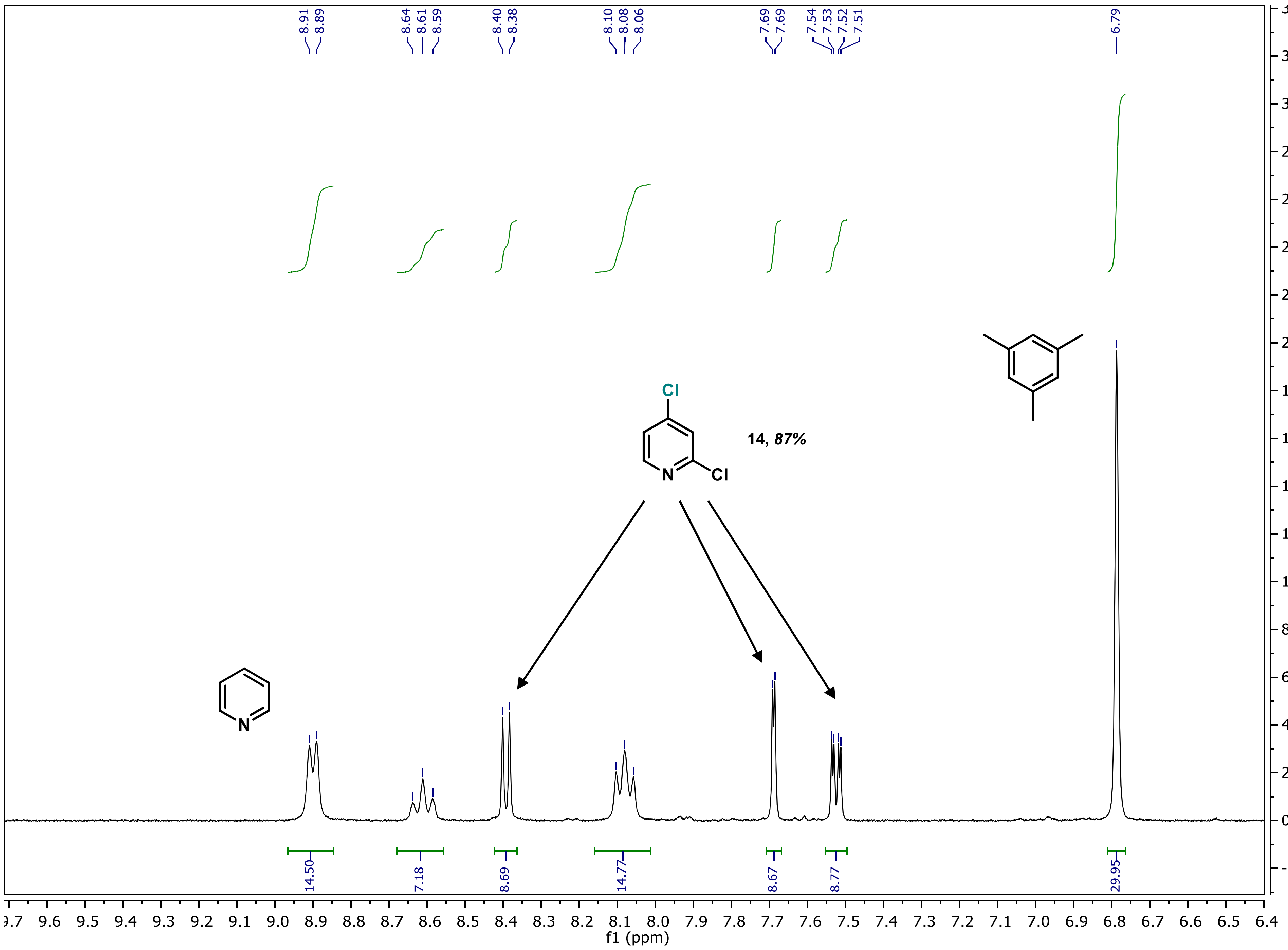

Mass to be matched (m/z): 147.971520 Charge: 1

Mass Tolerance: ±0.005000
Restriction of atom numbers:

C	H	N	Cl
1-110	1-100	1-3	1-2

Number of calculated Formulas: 1

Formula	Diff.(ppm)	theor. m/z
C5 H4 N1 Cl2	0.06	147.971530

15.06.2020
File: 147522b-00

Analyse: GOA-GA-332-01
COP: Dr. Gomez, Alejandro

Messung: HRMS ESIpos
Lösemittel: CH3OH
Spektrometer: Exactive

Auswerter: Kohler (2243)

Suggestion:
C5H3N1Cl2 MW 147

Characteristicial ions:
148 = [147 + H]+

Mass to be matched (m/z): 191.037120 Charge: 1

Mass Tolerance: ±0.005000
Restriction of atom numbers:

C	H	N	Cl
1-110	1-100	1-3	1-2

Number of calculated Formulas: 1

Formula	Diff.(ppm)	theor. m/z
C10 H8 N2 Cl1	-0.36	191.037050

Suggestion:
[C10H8N2Cl1]+ [BF4]- MW 278

Characteristicial ions:
191 = [278 - BF4]+

^{1}H NMR – in situ

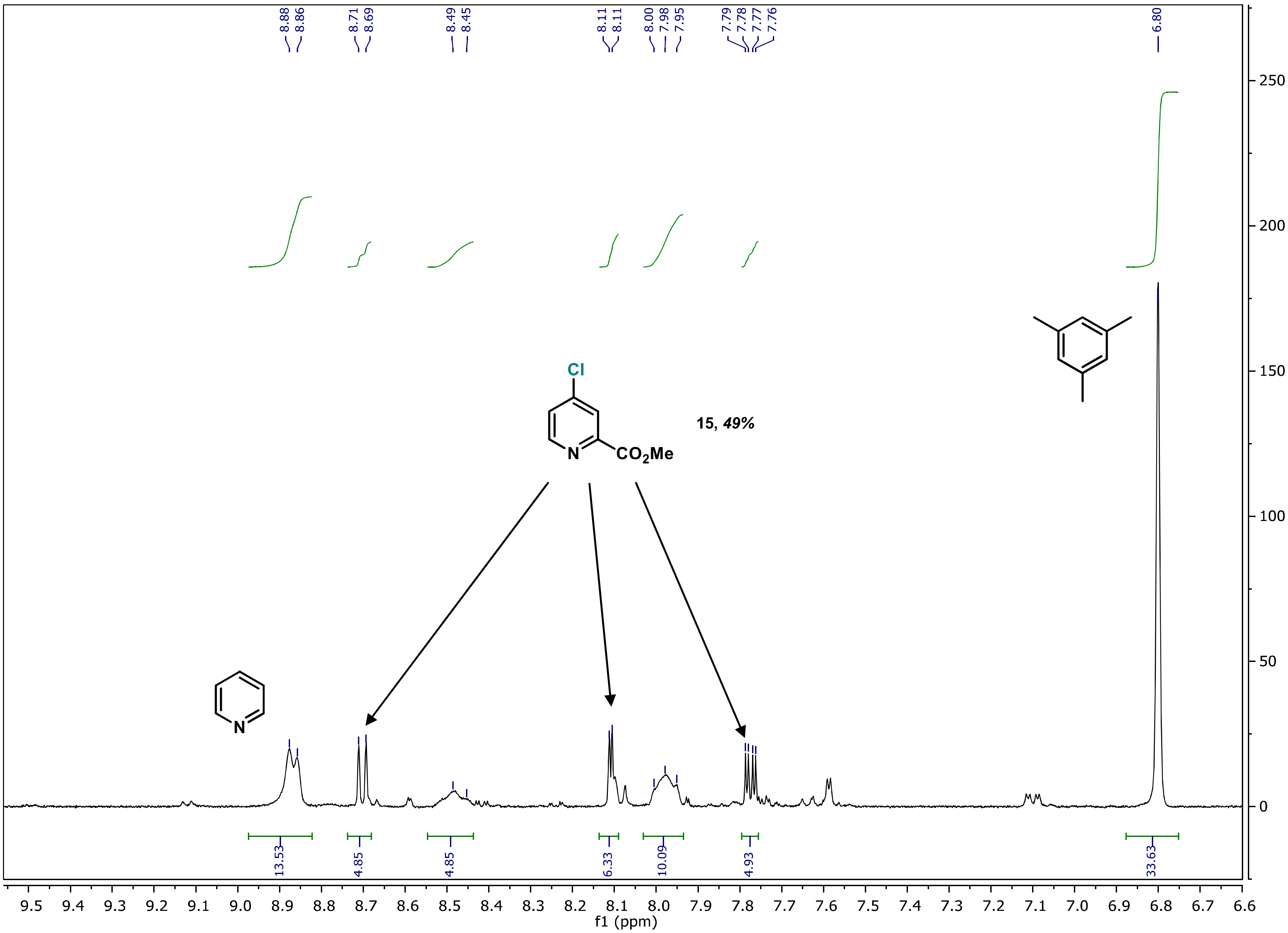

No.	MW.	Comment
1	171	Your proposed structure is possible.<br>Heteroatoms: 1 Cl<br>Ref.-spectrum: U22331:148235a-00 GOA-GA-395-01

20.07.2020
File: 148235a-00.raw
Analyse: GOA-GA-395-01
COP: Dr. Gomez, Alejandro

Messung:	GC-MS
Ionisierung:	GC-EI
Spektrometer:	ISQ Series
Säule:	MS 84 TG-5 SILMS
Länge:	30
Temp.:	35-5-285-5
GC-Nr.:	-
ELNA-Nr.:	26231
Auswerter:	Haupt (2243)

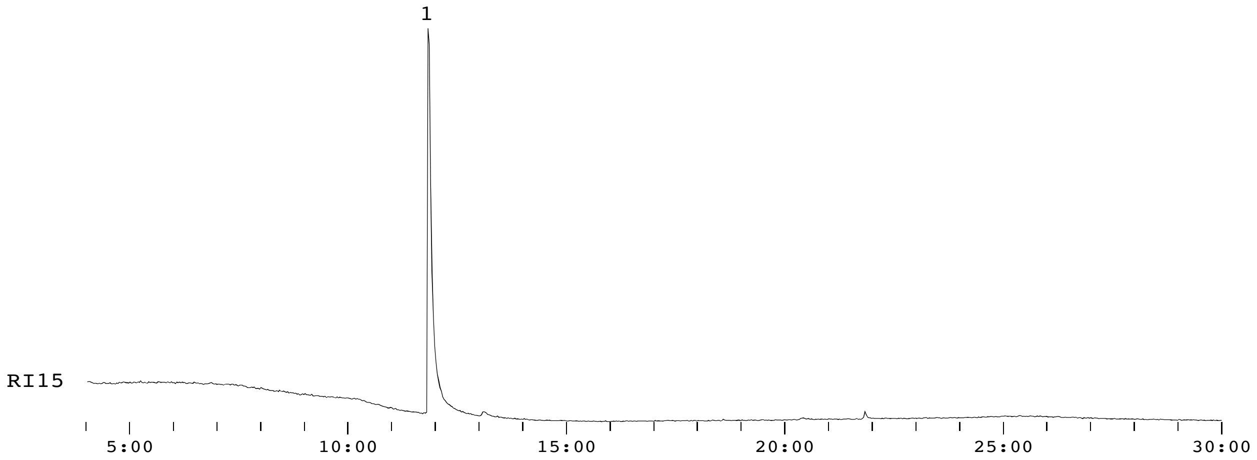

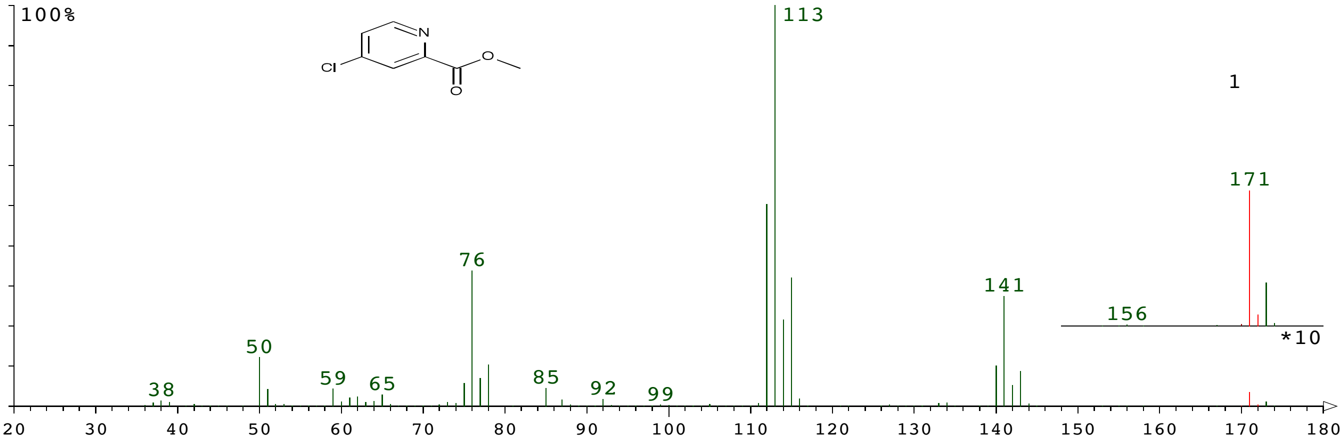

U22331 27% MW:171/171.58 C7H6Cl1N1O2 148235a-00 GOA-GA-395-01

36	0.17	60	1.09	87	1.56	108	0.05	138	0.01
37	0.85	61	2.05	88	0.30	109	0.05	139	0.08
38	1.33	62	2.31	89	0.06	110	0.15	140	10.08
39	0.92	63	1.04	90	0.14	111	0.70	141	27.46
41	0.05	64	1.25	91	0.07	112	50.38	142	5.18
42	0.45	65	2.82	92	1.73	113	100.00	143	8.78
43	0.03	66	0.55	93	0.18	114	21.55	144	0.62
44	0.03	67	0.13	94	0.12	115	32.10	145	0.04
45	0.09	68	0.02	95	0.02	116	1.89	153	0.01
46	0.01	69	0.03	96	0.07	117	0.06	155	0.01
48	0.12	70	0.01	97	0.03	121	0.01	156	0.03
50	12.18	71	0.14	98	0.04	122	0.01	158	0.01
51	4.19	72	0.37	99	0.25	126	0.15	167	0.02
52	0.50	73	0.92	100	0.24	127	0.29	170	0.04
53	0.55	74	0.76	101	0.16	128	0.08	171	3.38
54	0.16	75	5.72	102	0.12	129	0.08	172	0.29
55	0.06	76	33.76	103	0.06	130	0.01	173	1.08
56	0.15	77	6.89	104	0.06	131	0.02	174	0.07
57	0.07	78	10.24	105	0.52	133	0.68		
58	0.05	81	0.02	106	0.01	134	0.82		
59	4.24	85	4.49	107	0.01	135	0.05		

U22331 27% MW:171/171.58 C7H6Cl1N1O2 148235a-00 GOA-GA-395-01

^{1}H NMR

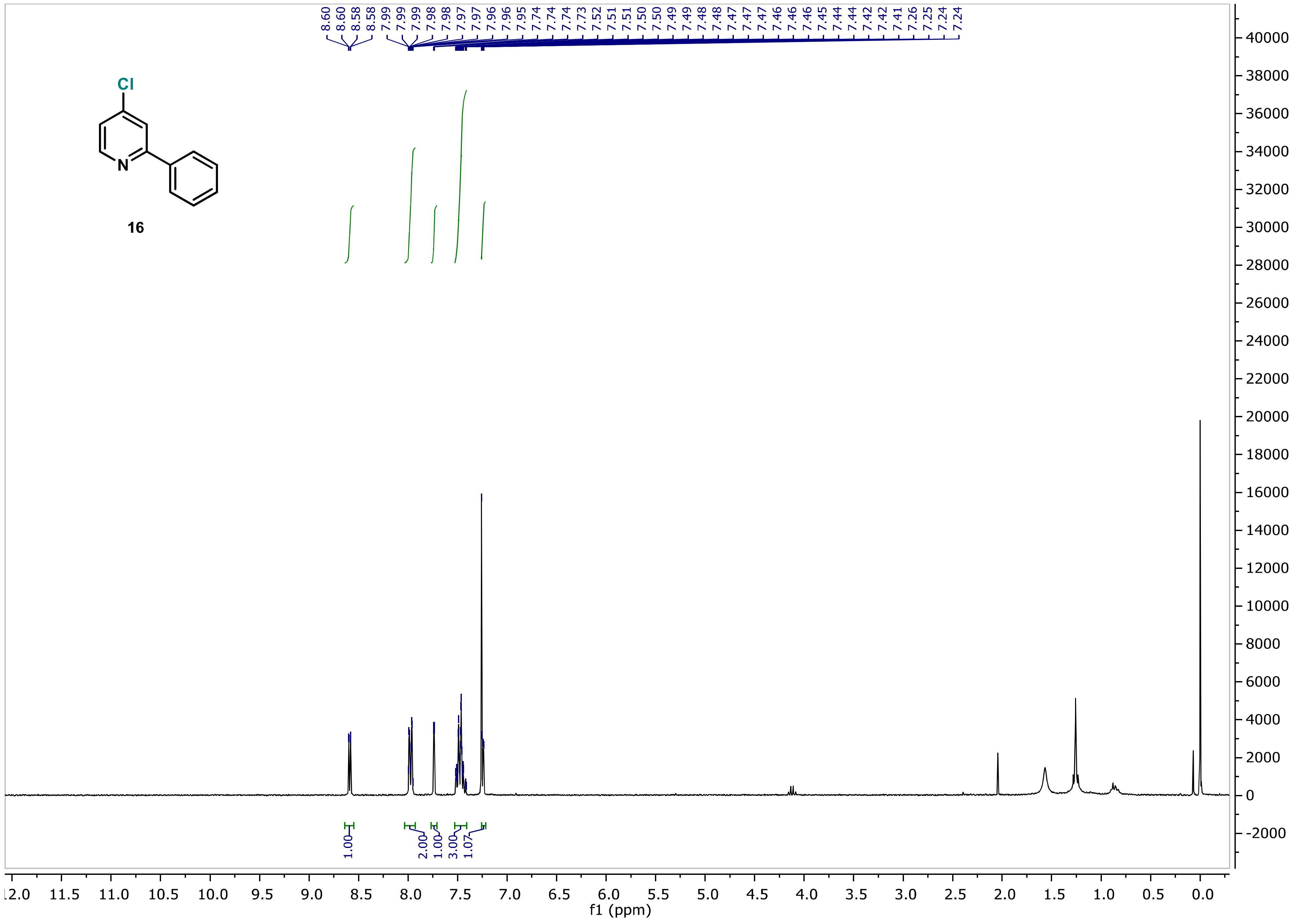
8.60 8.60 8.58 8.58 7.99 7.99 7.99 7.98 7.98 7.97 7.97 7.96 7.96 7.95 7.74 7.74 7.74 7.73 7.52 7.51 7.51 7.50 7.50 7.49 7.49 7.48 7.48 7.47 7.47 7.47 7.46 7.46 7.46 7.45 7.44 7.44 7.42 7.42 7.41 7.26 7.25 7.24 7.24
Cl
N
16
1.00
2.00
1.00
3.00
1.07
f1 (ppm)

¹H NMR

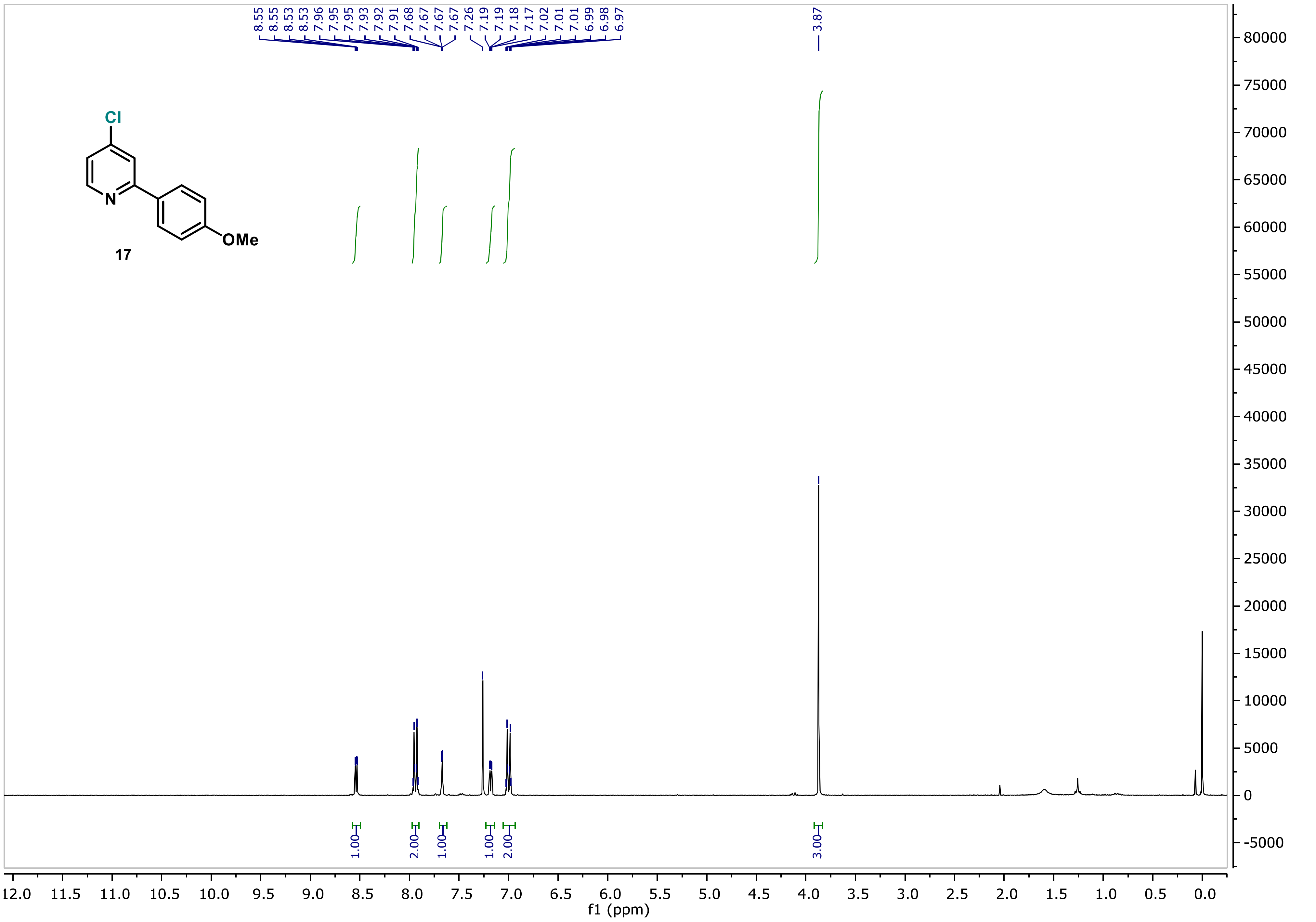
8.55
8.55
8.53
8.53
7.96
7.95
7.95
7.93
7.92
7.91
7.68
7.67
7.67
7.67
7.26
7.19
7.19
7.18
7.17
7.02
7.01
7.01
6.99
6.98
6.97
3.87
1.00
2.00
1.00
1.00
2.00
3.00
f1 (ppm)

17

^{1}H NMR – in situ

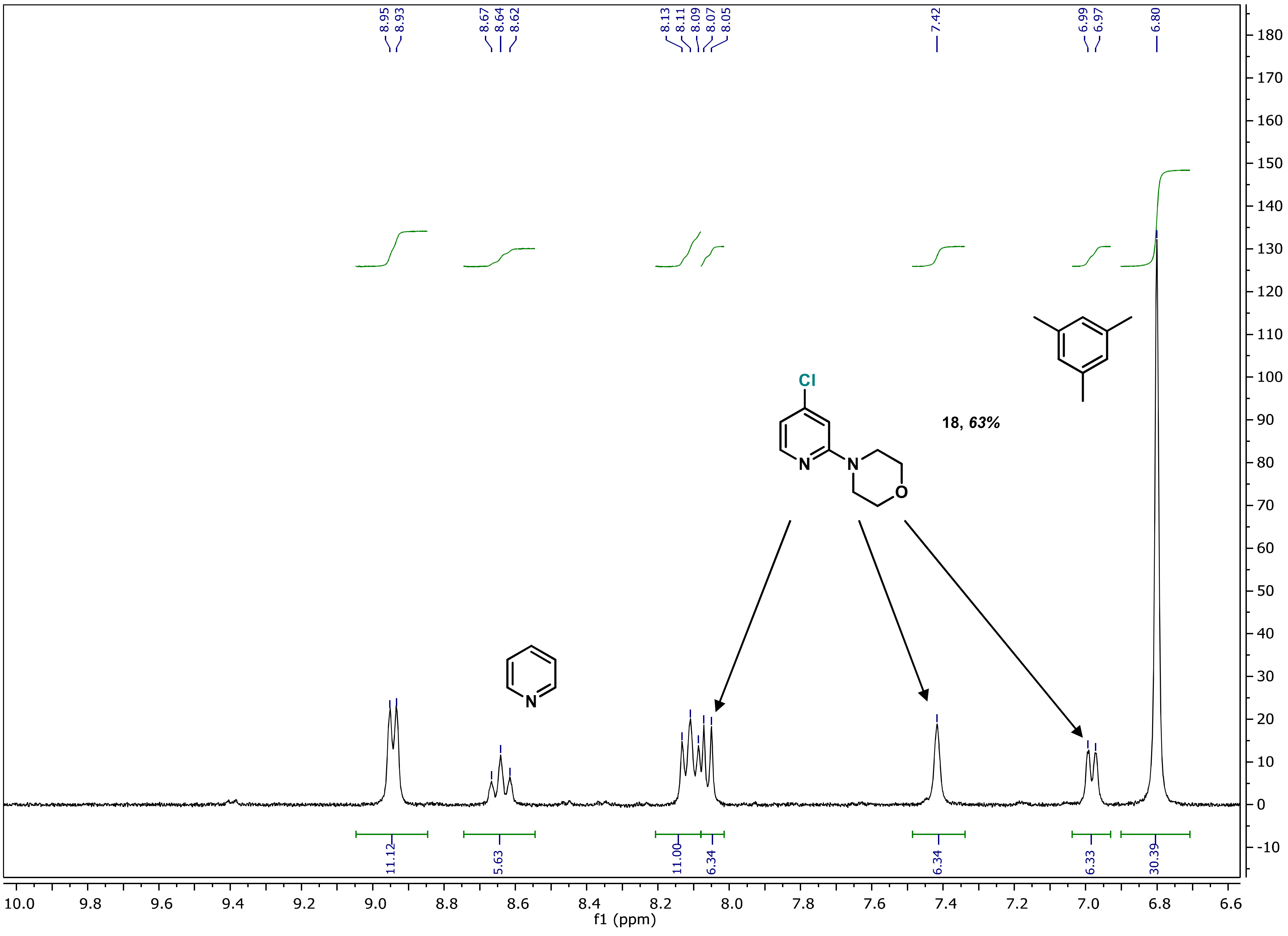

MassLib

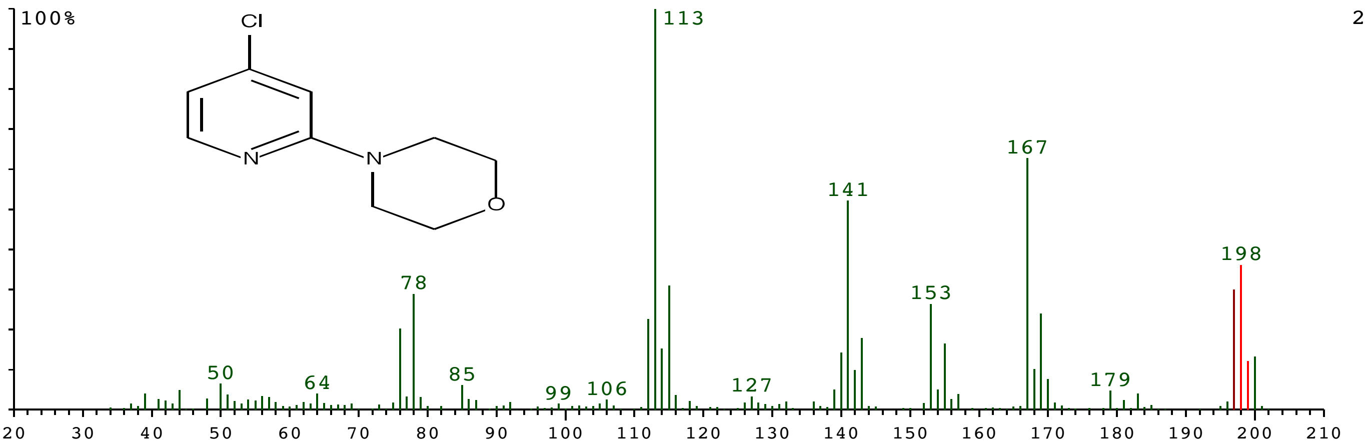

U22277 14% MW:198/198.65 C9H11Cl1N2O1 148066a-00 GHC-GA-167-01

34	0.43	59	0.74	82	0.81	107	0.94	130	0.80	157	3.84	182	0.40
36	0.28	60	0.71	85	6.13	108	0.15	131	1.31	159	0.36	183	3.99
37	1.38	61	1.09	86	2.64	110	0.16	132	2.01	161	0.31	184	0.54
38	0.82	62	1.78	87	2.40	111	0.57	133	0.40	162	0.49	185	1.07
39	3.90	63	1.52	89	0.23	112	22.48	136	1.98	163	0.35	186	0.05
41	2.50	64	3.95	90	0.86	113	100.00	137	0.82	164	0.16	187	0.14
42	2.24	65	1.57	91	1.04	114	15.21	138	0.50	165	0.70	192	0.11
43	1.42	66	1.10	92	1.79	115	30.85	139	5.05	166	0.83	195	0.82
44	4.75	67	1.24	93	0.01	116	3.57	140	14.18	167	62.70	196	1.91
45	0.05	68	1.06	95	0.21	117	0.36	141	52.16	168	10.16	197	29.94
46	0.23	69	1.38	96	0.68	118	2.11	142	9.81	169	23.89	198	36.09
48	2.67	70	0.13	97	0.28	119	0.79	143	17.78	170	7.59	199	12.14
50	6.46	72	0.22	98	0.44	121	0.50	144	0.89	171	1.64	200	13.16
51	3.77	73	1.16	99	1.38	122	0.50	145	0.72	172	1.02	201	0.86
52	2.08	74	0.08	100	0.23	123	0.17	149	0.32	173	0.27	202	0.11
53	1.50	75	1.63	101	0.86	124	0.01	150	0.40	174	0.23		
54	2.46	76	20.18	102	0.95	125	0.33	152	1.61	176	0.34		
55	2.21	77	3.17	103	0.73	126	1.63	153	26.34	178	0.37		
56	3.37	78	28.82	104	0.76	127	3.28	154	5.01	179	4.70		
57	3.11	79	3.07	105	1.45	128	1.73	155	16.39	180	0.37		
58	1.83	80	0.77	106	2.45	129	1.35	156	2.56	181	2.30		

U22277 14% MW:198/198.65 C9H11Cl1N2O1 148066a-00 GHC-GA-167-01

^{1}H NMR – in situ

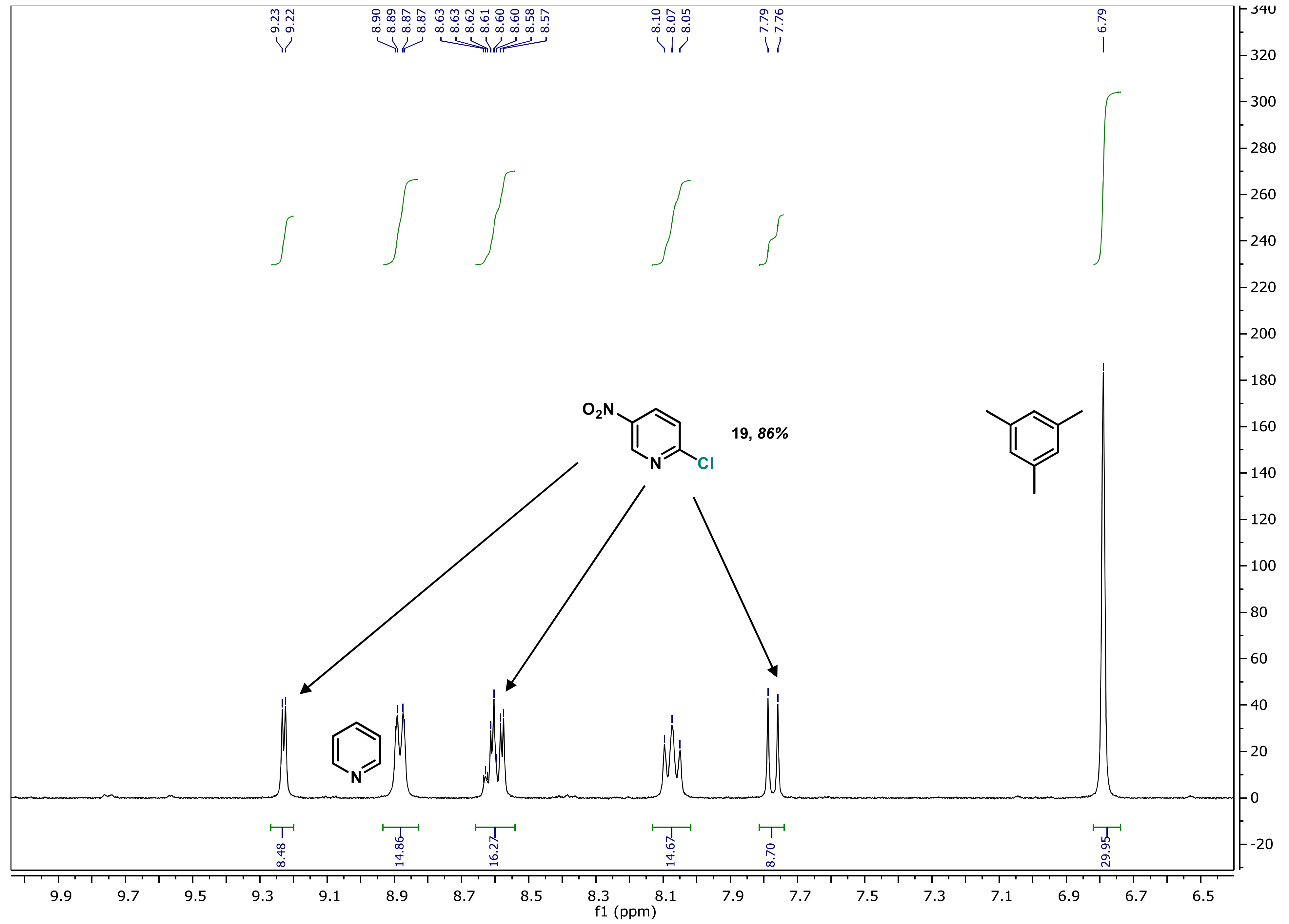

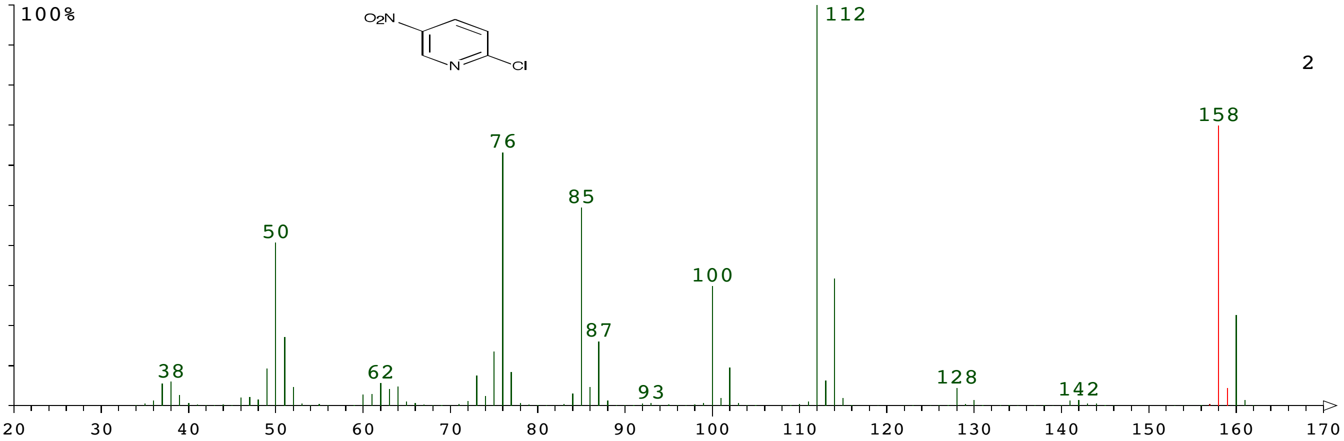

U22337 17% MW:158/158.54 C5H3Cl1N2O2 148237a-00 GOA-GA-389-01

34	0.03	55	0.32	78	0.58	99	0.51	127	0.08	157	0.27		
35	0.49	56	0.03	79	0.21	100	29.79	128	4.32	158	69.85		
36	1.16	59	0.16	80	0.13	101	1.79	129	0.31	159	4.41		
37	5.41	60	2.70	81	0.05	102	9.47	130	1.34	160	22.59		
38	5.89	61	2.81	82	0.01	103	0.63	131	0.08	161	1.37		
39	2.54	62	5.56	83	0.32	104	0.05	133	0.04	162	0.12		
40	0.55	63	4.04	84	3.01	105	0.04	134	0.06				
41	0.20	64	4.69	85	49.41	108	0.01	135	0.01				
42	0.08	65	0.99	86	4.60	109	0.13	137	0.03				
43	0.06	66	0.51	87	15.96	110	0.26	138	0.08				
44	0.21	67	0.21	88	1.23	111	1.00	139	0.01				
45	0.07	68	0.06	89	0.11	112	100.00	140	0.05				
46	1.98	69	0.03	90	0.08	113	6.22	141	1.23				
47	2.05	70	0.01	91	0.05	114	31.62	142	1.35				
48	1.39	71	0.29	92	0.42	115	1.78	143	0.49				
49	9.20	72	1.11	93	0.55	116	0.05	144	0.45				
50	40.60	73	7.39	94	0.11	119	0.01	145	0.03				
51	17.05	74	2.30	95	0.21	122	0.01	146	0.01				
52	4.50	75	13.50	96	0.05	123	0.08	153	0.01				
53	0.42	76	63.17	97	0.09	125	0.01	154	0.01				
54	0.11	77	8.29	98	0.24	126	0.03	156	0.02				

U22337 17% MW:158/158.54 C5H3Cl1N2O2 148237a-00 GOA-GA-389-01

^{1}H NMR – in situ

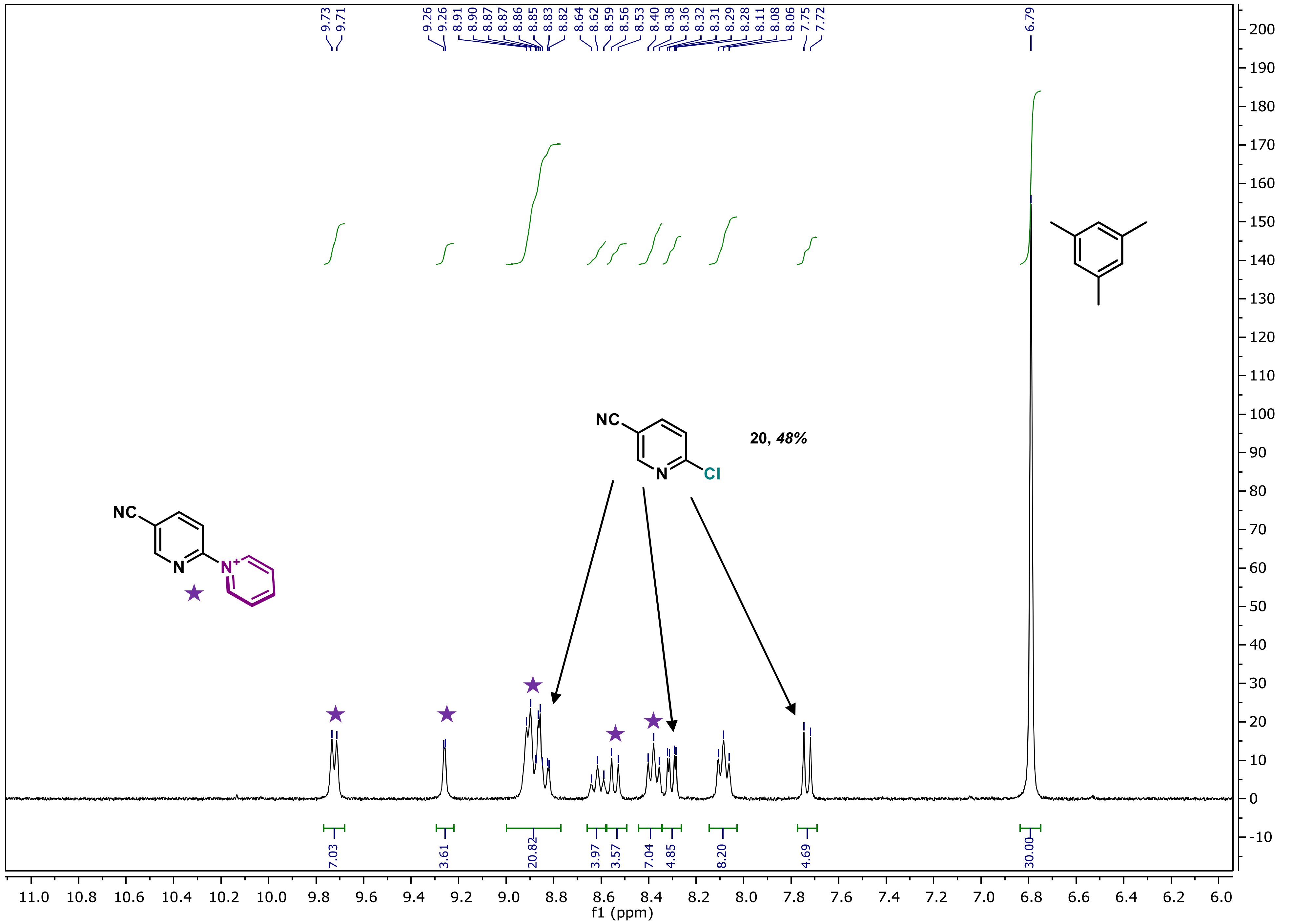

Mass to be matched (m/z): 139.005770 Charge: 1

Mass Tolerance: ±0.005000
Restriction of atom numbers:

C	H	N	Cl
1-110	1-100	1-3	1-2

Number of calculated Formulas: 1

Formula	Diff.(ppm)	theor. m/z
C6 H4 N2 Cl1	-0.14	139.005750

15.06.2020
File: 147526b-00

Analyse: GOA-GA-337-01
COP: Dr. Gomez, Alejandro

Messung:	HRMS ESIpos
Lösemittel:	CH3OH
Spektrometer:	Exactive
Auswerter:	Kohler (2243)

Suggestion:
C6H3N2Cl1   MW 138

Characteristicial ions:
139 = [138 + H]+

Mass to be matched (m/z): 182.071430 Charge: 1

Mass Tolerance: ±0.005000
Restriction of atom numbers:

C	H	N	Cl
1-110	1-100	1-3	1-2

Number of calculated Formulas: 1

Formula	Diff.(ppm)	theor. m/z
C10 H13 N1 Cl1	9.18	182.073102

Suggestion:
[C10H13N1Cl1]+ [BF4]-   MW 269

Characteristicial ions:
182 = [269 - BF4]+

^{1}H NMR – in situ

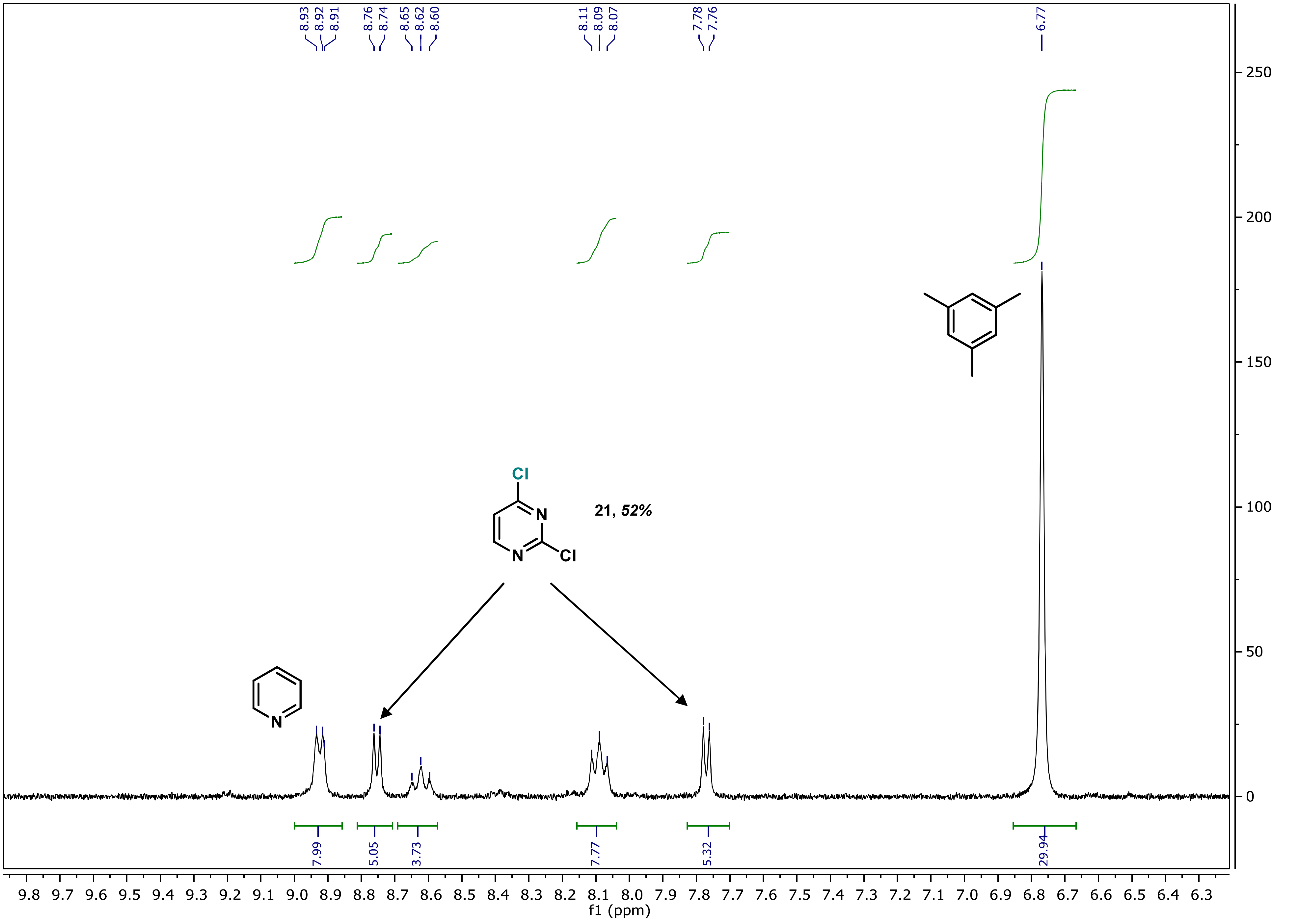

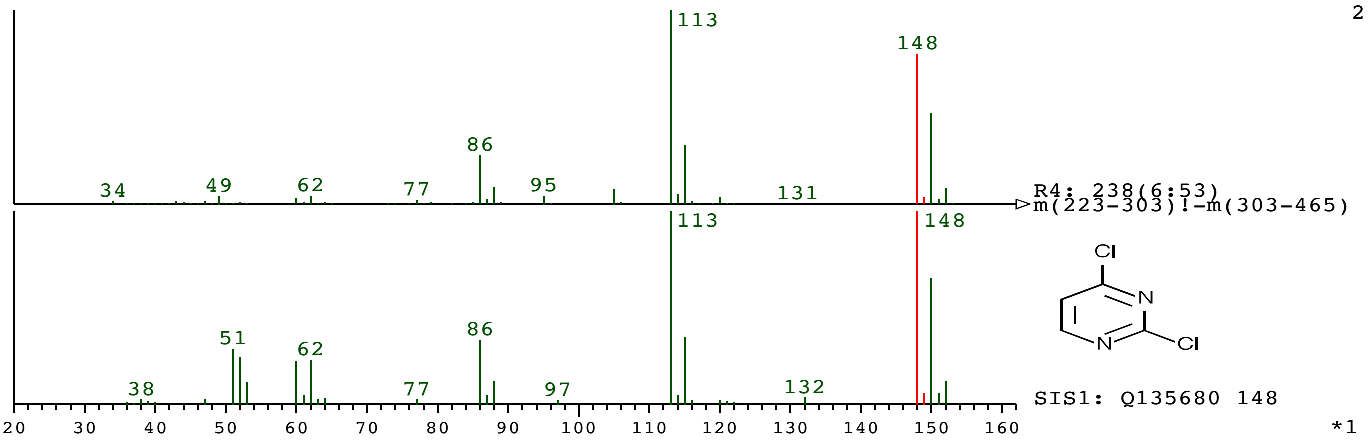

33	0.02	60	2.76	96	0.12	153	0.31
34	1.44	61	0.71	105	7.54		
35	0.03	62	4.09	106	0.87		
36	0.04	63	0.22	111	0.02		
37	0.09	64	0.88	113	100.00		
38	0.20	65	0.03	114	4.70		
39	0.20	73	0.29	115	30.17		
40	0.09	74	0.13	116	1.44		
41	0.11	75	0.02	117	0.12		
42	0.25	76	0.08	119	0.03		
43	1.06	77	1.97	120	3.22		
44	0.85	78	0.32	121	0.11		
45	0.38	79	0.54	122	0.24		
46	0.11	82	0.04	125	0.00		
47	1.14	84	0.18	131	0.01		
48	0.14	85	0.77	147	0.02		
49	3.89	86	25.19	148	77.49		
50	0.36	87	2.53	149	3.59		
51	0.14	88	8.70	150	46.88		
52	1.00	89	0.66	151	2.18		
53	0.17	95	3.99	152	7.86		

Mass to be matched (m/z): 147.959150 Charge: 1

Mass Tolerance: ±0.005000
Restriction of atom numbers:

C	H	N	Cl
1-110	1-100	1-2	2-2

Number of calculated Formulas: 1

Formula	Diff.(ppm)	theor. m/z
C4 H2 N2 Cl2	-1.33	147.958954

18.01.2021
File: 151244a-00.raw
Analyse: GHC-GA-535-01
COP: Dr. Clement Ghiazza

Messung:	GC-MS
Ionisierung:	GC EI
Spektrometer:	QExactiveGC
Säule:	MS 50 RTX1+VS
Länge:	30+7
Temp.:	35-10-285-5
GC-Nr.:	-
MS-Nr.:	29197
Auswerter:	Margold (2242)

^{1}H NMR

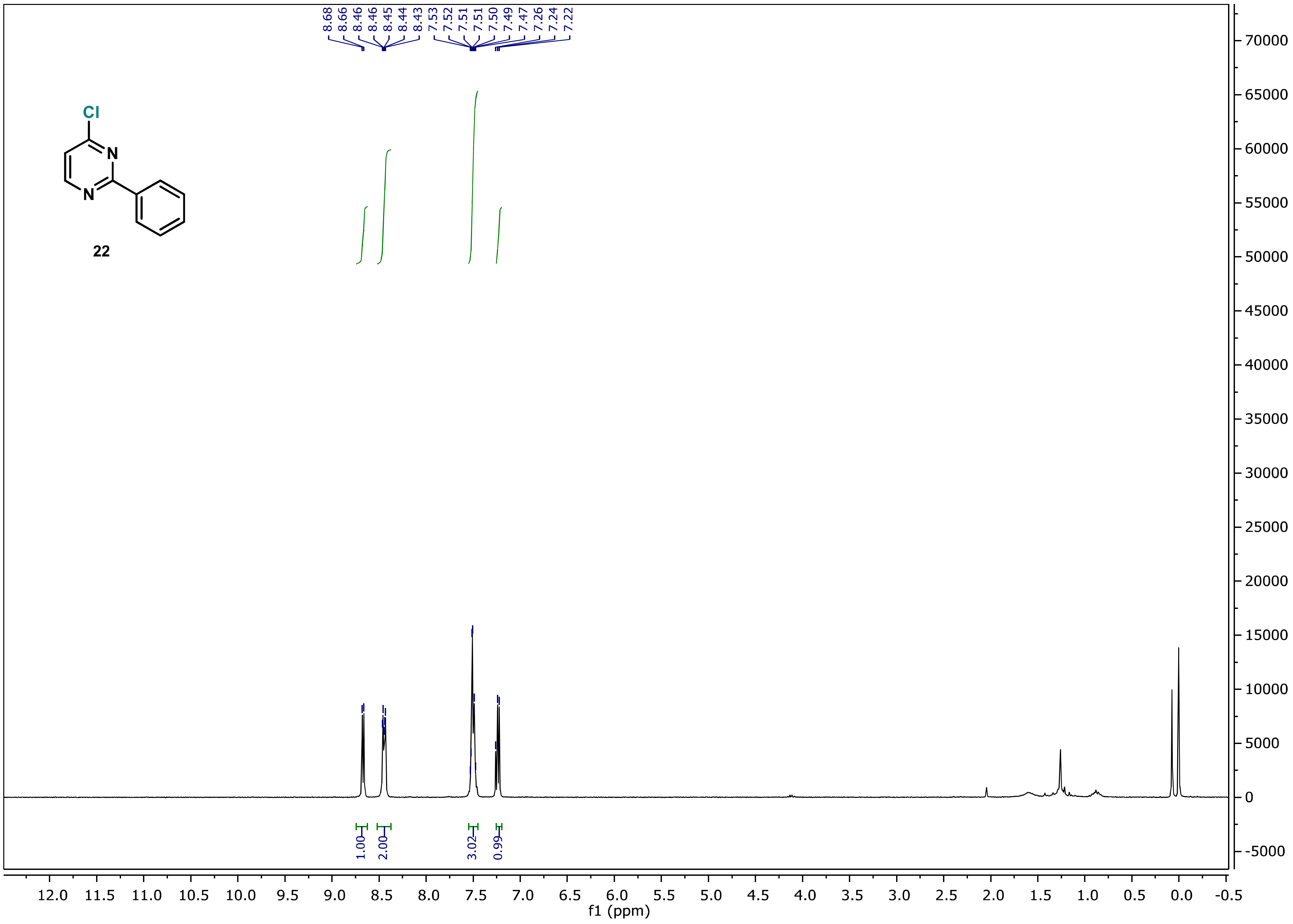
8.68
8.66
8.46
8.46
8.45
8.44
8.43
7.53
7.52
7.51
7.51
7.50
7.49
7.47
7.26
7.24
7.22
Cl
N
N
22
1.00
2.00
3.02
0.99
f1 (ppm)

^{13}C NMR

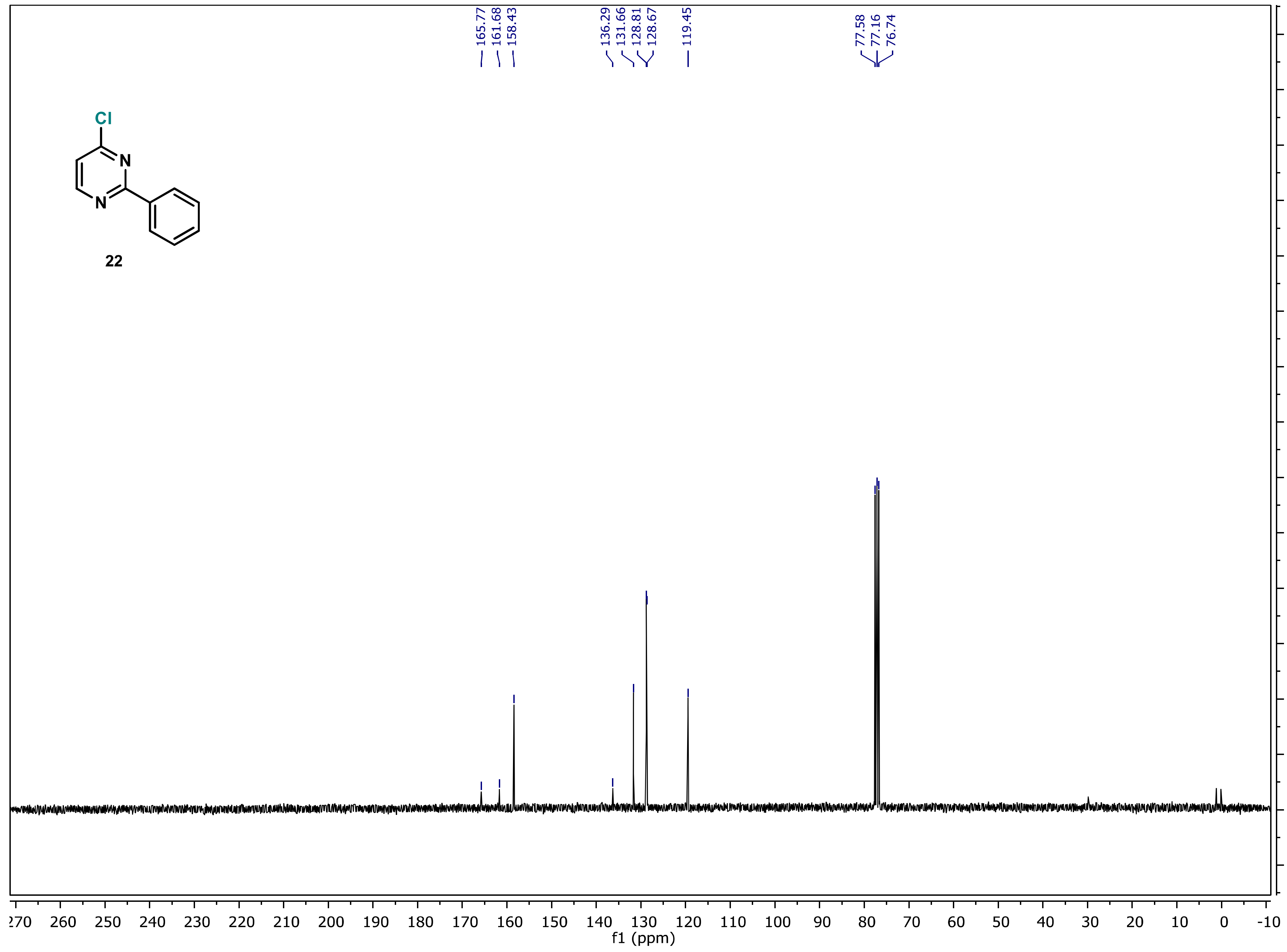

165.77
161.68
158.43
136.29
131.66
128.81
128.67
119.45
77.58
77.16
76.74
Cl
N
N
22
f1 (ppm)

Mass to be matched (m/z): 190.029520 Charge: 1

Mass Tolerance: ±0.050000
Restriction of atom numbers:

C	H	N	Cl
1-100	1-100	1-2	1-2

Number of calculated Formulas: 4

Formula	Diff.(ppm)	theor. m/z
C10 H7 N2 Cl1	-1.55	190.029225
C8 H10 N1 Cl2	-58.10	190.018480
C11 H9 N1 Cl1	64.63	190.041802
C7 H8 N2 Cl2	-124.28	190.005903

2.10.2020
File: 149660b-00.raw
Analyse: GHC-GA-361-01
COP: Dr. Clement Ghiazza

Messung:	GC-MS
Ionisierung:	GC-EI
Spektrometer:	Q Exactive GC Orbitrap
Säule:	MS 50 TX1+VS
Länge:	30+7
Temp.:	35-10-285-5
GC-Nr.:	-
ELNA-Nr.:	27638

Auswerter: Haupt (2243)

Suggestion:
C10H7N2Cl1 MW: 190

^{1}H NMR – in situ

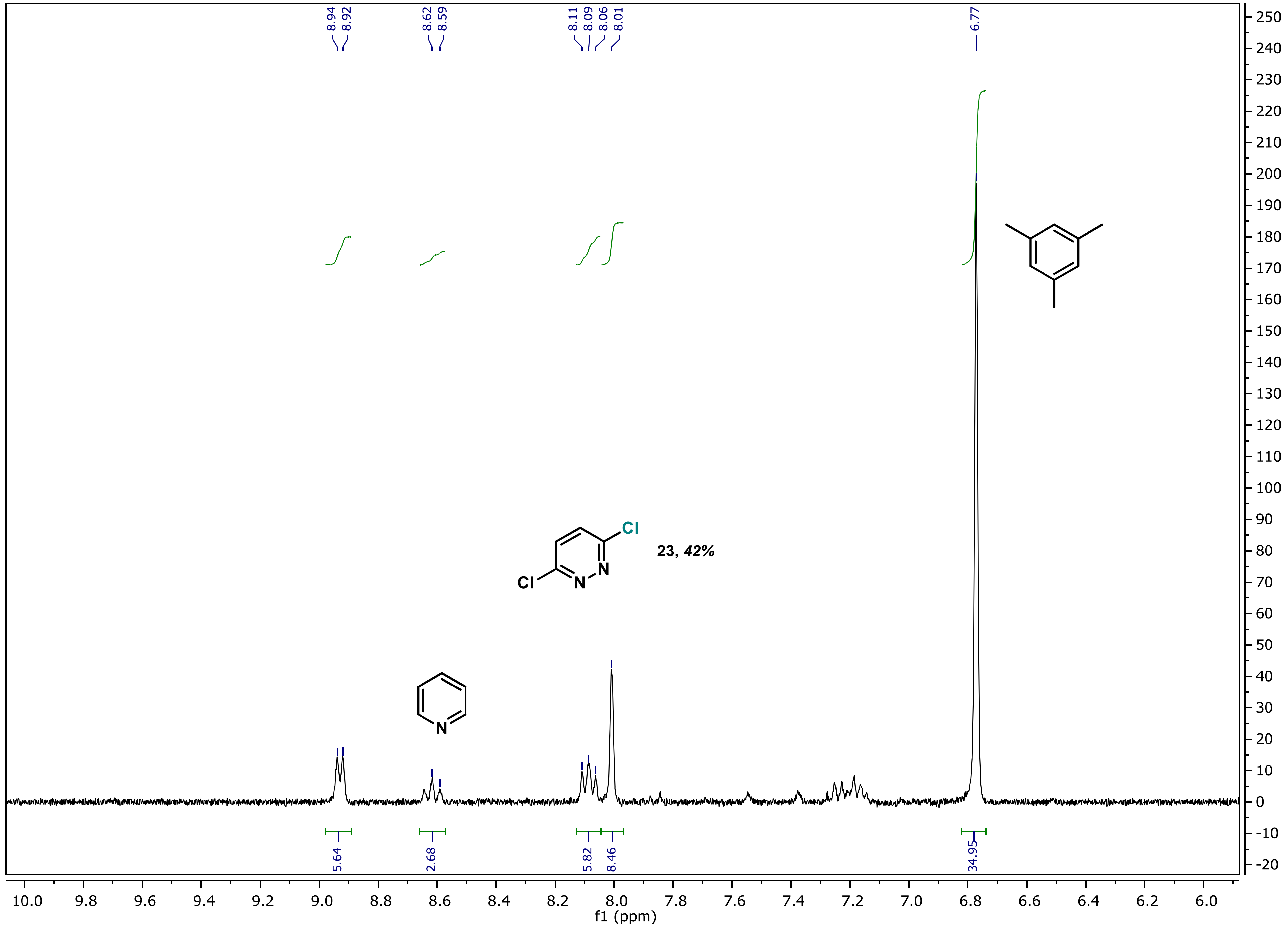

23, *42%*

Mass to be matched (m/z): 148.966910 Charge: 1

Mass Tolerance: ±0.020000
Restriction of atom numbers:

C	H	N	Cl
1-100	1-100	1-5	2-2

Number of calculated Formulas: 3

Formula	Diff.(ppm)	theor. m/z
C4 H3 N2 Cl2	-0.88	148.966778
C5 H5 N1 Cl2	83.54	148.979354
C3 H1 N3 Cl2	-85.31	148.954202

Datum:	3.08.2020
Analyse:	148468c-00
Sigel:	GHC-GA-251-01
	COP: Dr. Clement Ghiazza
Method:	HR-MS
Ionis. :	ESIpos
solvent :	CH2Cl2 + CH3OH
Spectrometer:	Exactive
Auswerter:	Marcus, Tel:2243

suggestion:
C4H2N2Cl2 MW: 148

Characteristic Ions:
149 = [148 + H]

^{1}H NMR

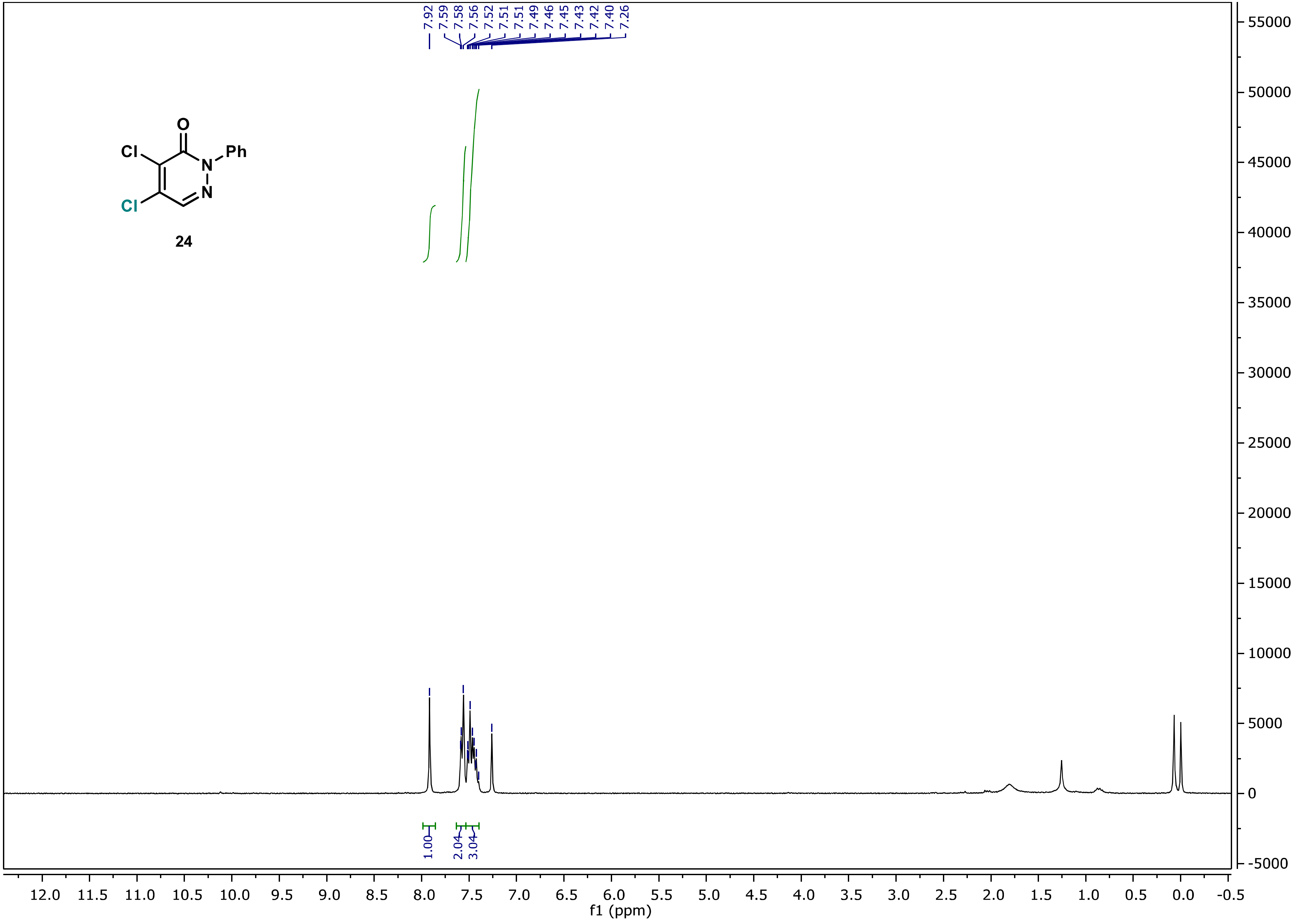
7.92
7.59
7.58
7.56
7.52
7.51
7.51
7.49
7.46
7.45
7.43
7.42
7.40
7.26
1.00
2.04
3.04
55000
50000
45000
40000
35000
30000
25000
20000
15000
10000
5000
0
-5000
12.0
11.5
11.0
10.5
10.0
9.5
9.0
8.5
8.0
7.5
7.0
6.5
6.0
5.5
5.0
4.5
4.0
3.5
3.0
2.5
2.0
1.5
1.0
0.5
0.0
-0.5
f1 (ppm)
O
Cl
Cl
N
N
Ph

24

^{13}C NMR

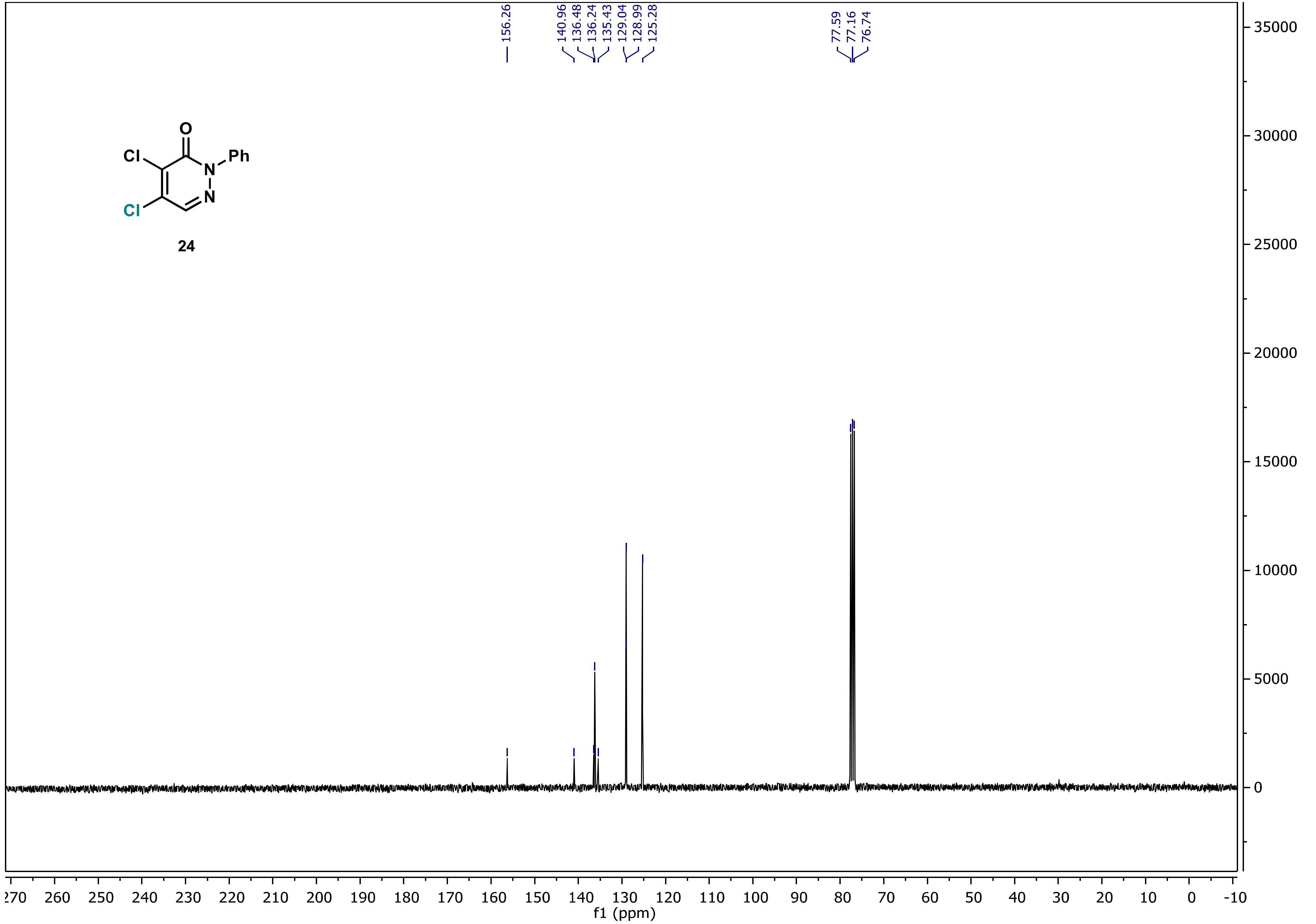

Cl
O
N
Ph
N
Cl
24
156.26
140.96
136.48
136.24
135.43
129.04
128.99
125.28
77.59
77.16
76.74
35000
30000
25000
20000
15000
10000
5000
0
270
260
250
240
230
220
210
200
190
180
170
160
150
140
130
120
110
100
90
80
70
60
50
40
30
20
10
0
-10
f1 (ppm)

Mass to be matched (m/z): 262.975020 Charge: 1

Mass Tolerance: ±0.050000
Restriction of atom numbers:

C	H	N	O	Na	Cl
1-100	1-100	1-2	1-3	1-1	1-2

Number of calculated Formulas: 11

Formula	Diff.(ppm)	theor. m/z
C10 H6 N2 O1 Na1 Cl2	-0.31	262.974938
C13 H3 N1 O2 Na1 Cl1	-2.17	262.974451
C11 H8 N1 O1 Na1 Cl2	47.51	262.987514
C12 H1 N2 O2 Na1 Cl1	-49.99	262.961875
C7 H10 N2 O3 Na1 Cl2	80.04	262.996068
C13 H5 N2 O1 Na1 Cl1	88.37	262.998260
C10 H4 N1 O2 Na1 Cl2	-90.85	262.951129
C8 H12 N1 O3 Na1 Cl2	127.86	263.008644
C14 H7 N1 O1 Na1 Cl1	136.19	263.010836
C9 H2 N2 O2 Na1 Cl2	-138.67	262.938552
C10 H9 N2 O3 Na1 Cl1	168.72	263.019389

18.08.2020
File: 148735f-00
Analysis: GHC-GA-210-01
COP: Dr. Clement Ghiazza

Messung:	HR-MS
Ionisierung:	ESIpos
Lösungsmittel:	CH2Cl2 + CH3OH
Spektrometer:	Exactive
ELNA:	26714
Auswerter:	Haupt (2243)

Suggestion:
C10H6N2O1Cl2 MW: 240

Characteristic ions:
263 = [240 + Na+]

^{1}H NMR – in situ

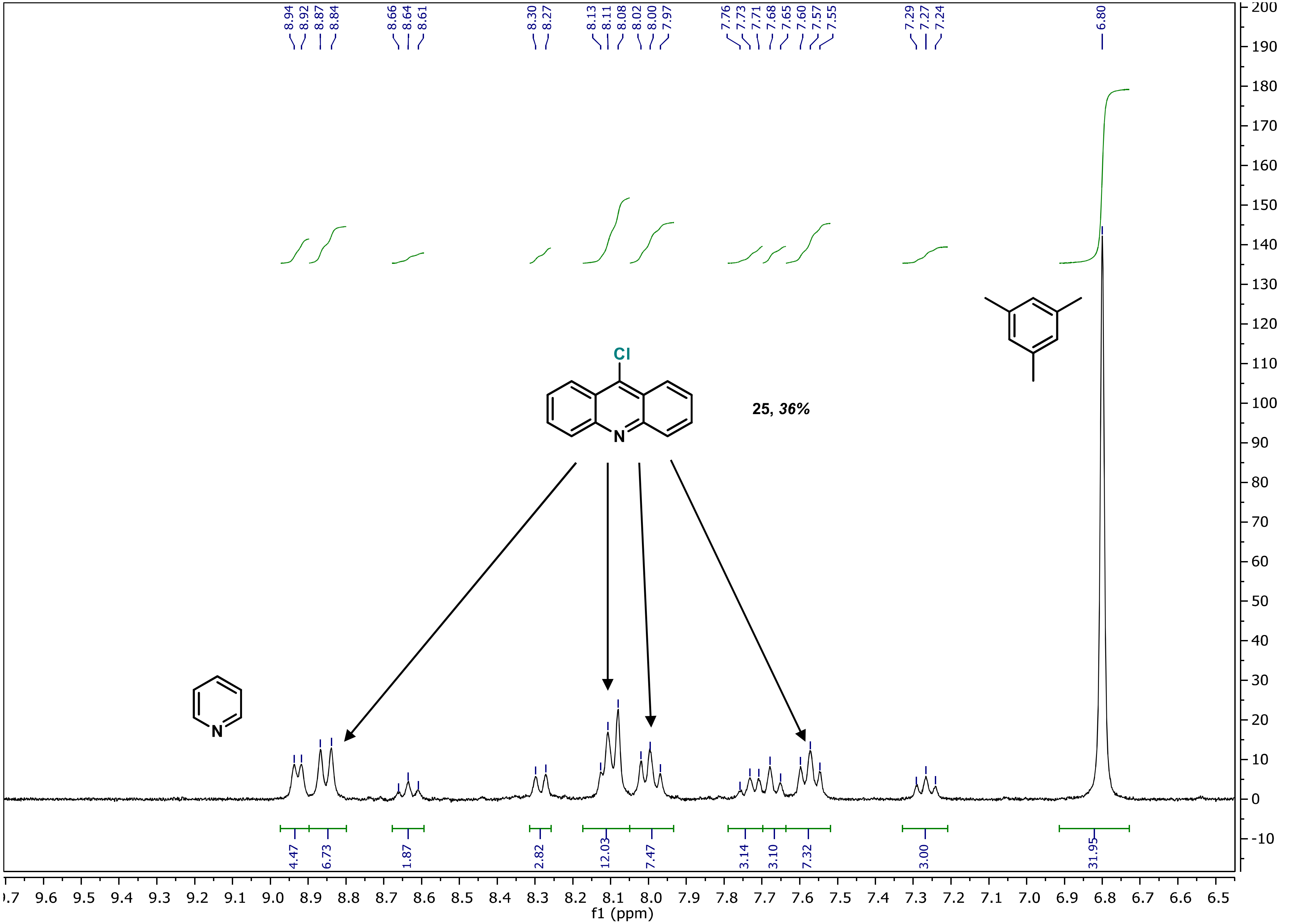

^{1}H NMR

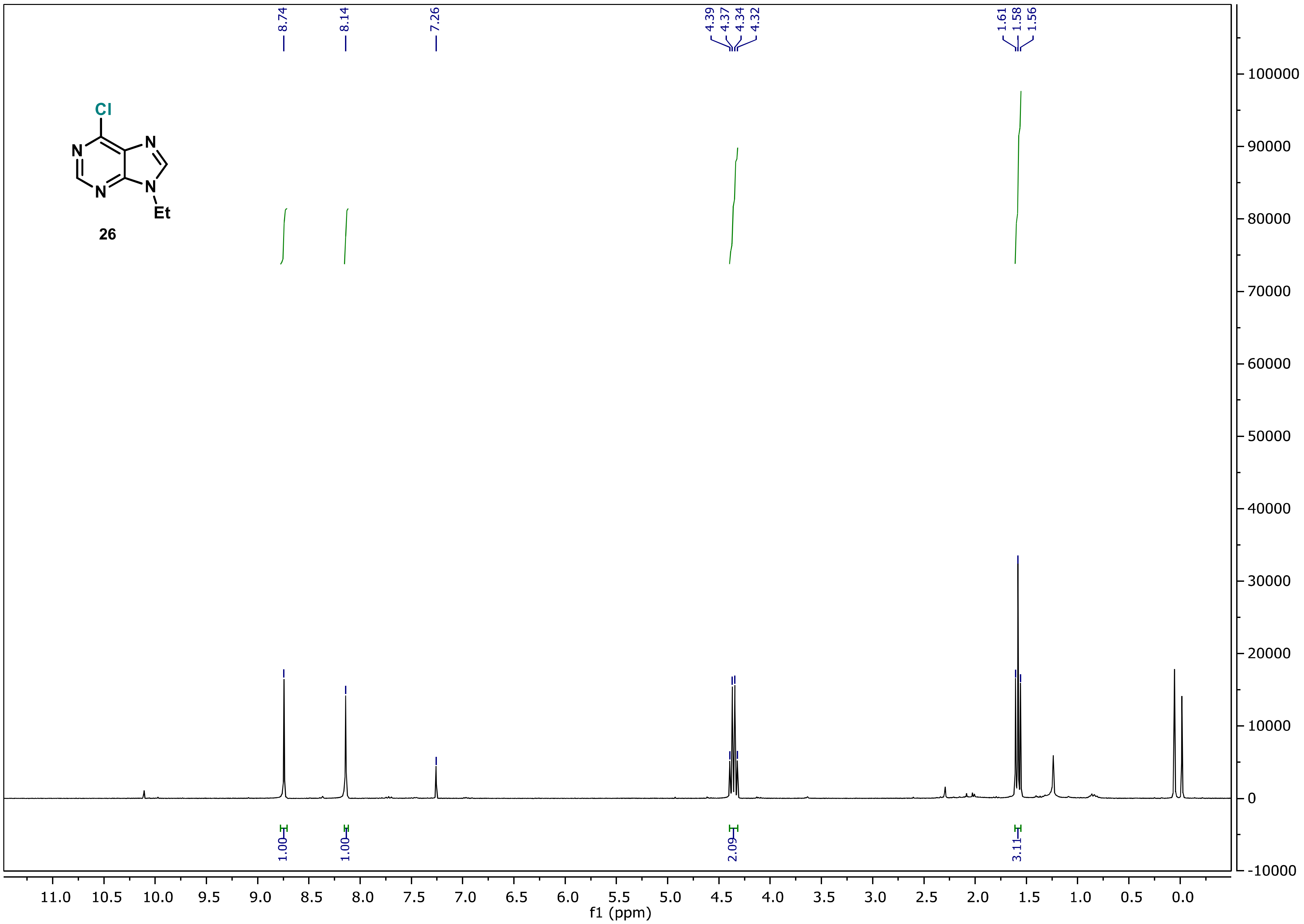

Cl
N
N
N
N
Et
26
8.74
8.14
7.26
4.39
4.37
4.34
4.32
1.61
1.58
1.56
1.00
1.00
2.09
3.11
100000
90000
80000
70000
60000
50000
40000
30000
20000
10000
0
-10000
11.0
10.5
10.0
9.5
9.0
8.5
8.0
7.5
7.0
6.5
6.0
5.5
5.0
4.5
4.0
3.5
3.0
2.5
2.0
1.5
1.0
0.5
0.0
f1 (ppm)

^{13}C NMR

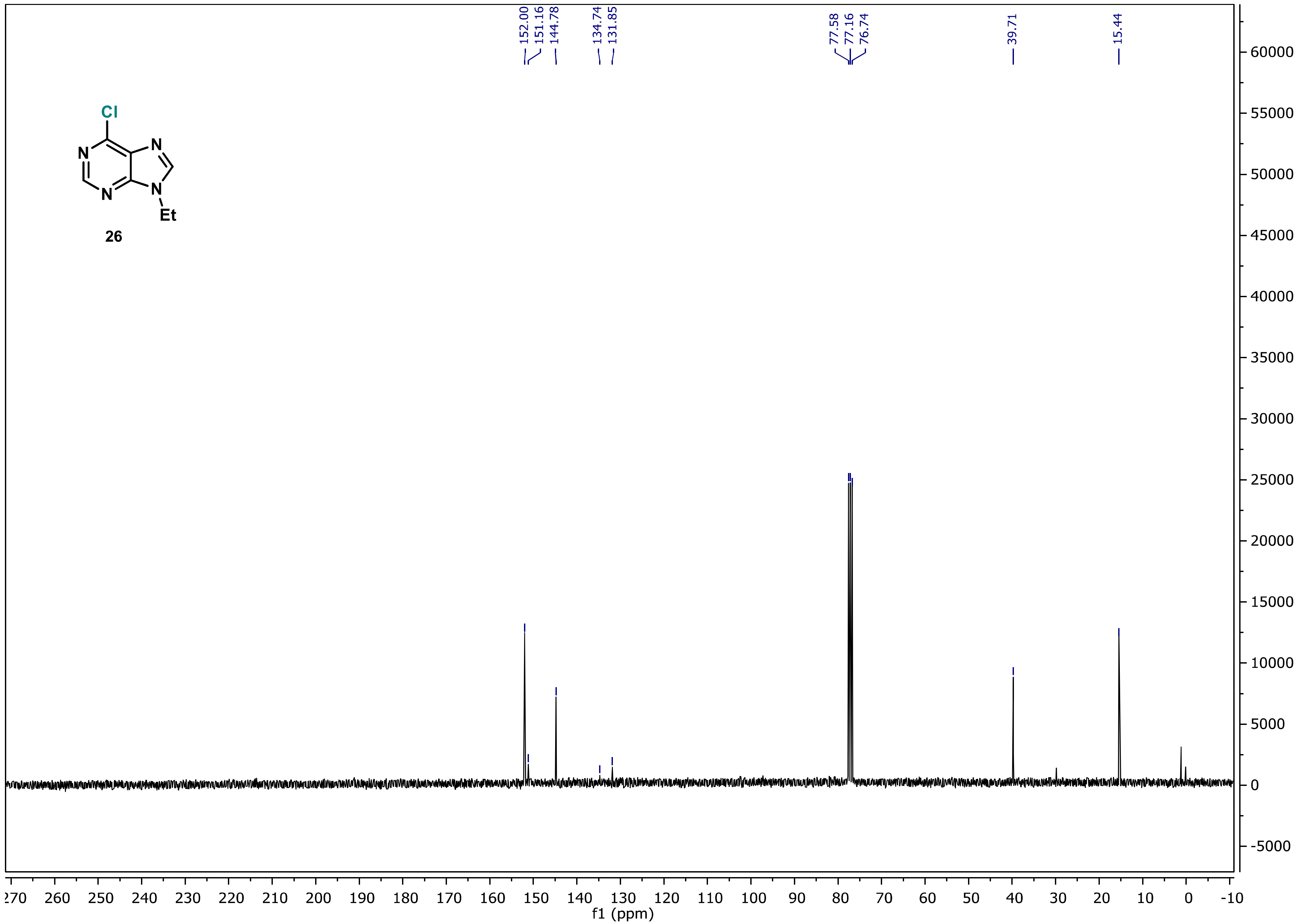

152.00
151.16
144.78
134.74
131.85
77.58
77.16
76.74
39.71
15.44
Cl
N
N
N
N
Et
26
60000
55000
50000
45000
40000
35000
30000
25000
20000
15000
10000
5000
0
-5000
270 260 250 240 230 220 210 200 190 180 170 160 150 140 130 120 110 100 90 80 70 60 50 40 30 20 10 0 -10
f1 (ppm)

Mass to be matched (m/z): 183.043180 Charge: 1

Mass Tolerance: ±0.005000
Restriction of atom numbers:

C	H	Cl	N
1-100	1-100	1-1	2-4

Number of calculated Formulas: 1

Formula	Diff.(ppm)	theor. m/z
C7 H8 Cl1 N4	0.10	183.043198

Datum	24.07.2020
Analyse:	148346c-00
Sigel:	GHS-GA-224-01
LIS: Dr. Gosh, Santanu	
Messung:	HRMS
Methode:	ESIpos
Lösungsmittel:	CH2Cl2+CH3OH
Spektrometer:	Exactive
Auswerter:	Kampen (2242)

Suggestion:
C7H7Cl1N4 MW 182

characteristical ion
183 = [182 + H]+

^{1}H NMR – in situ

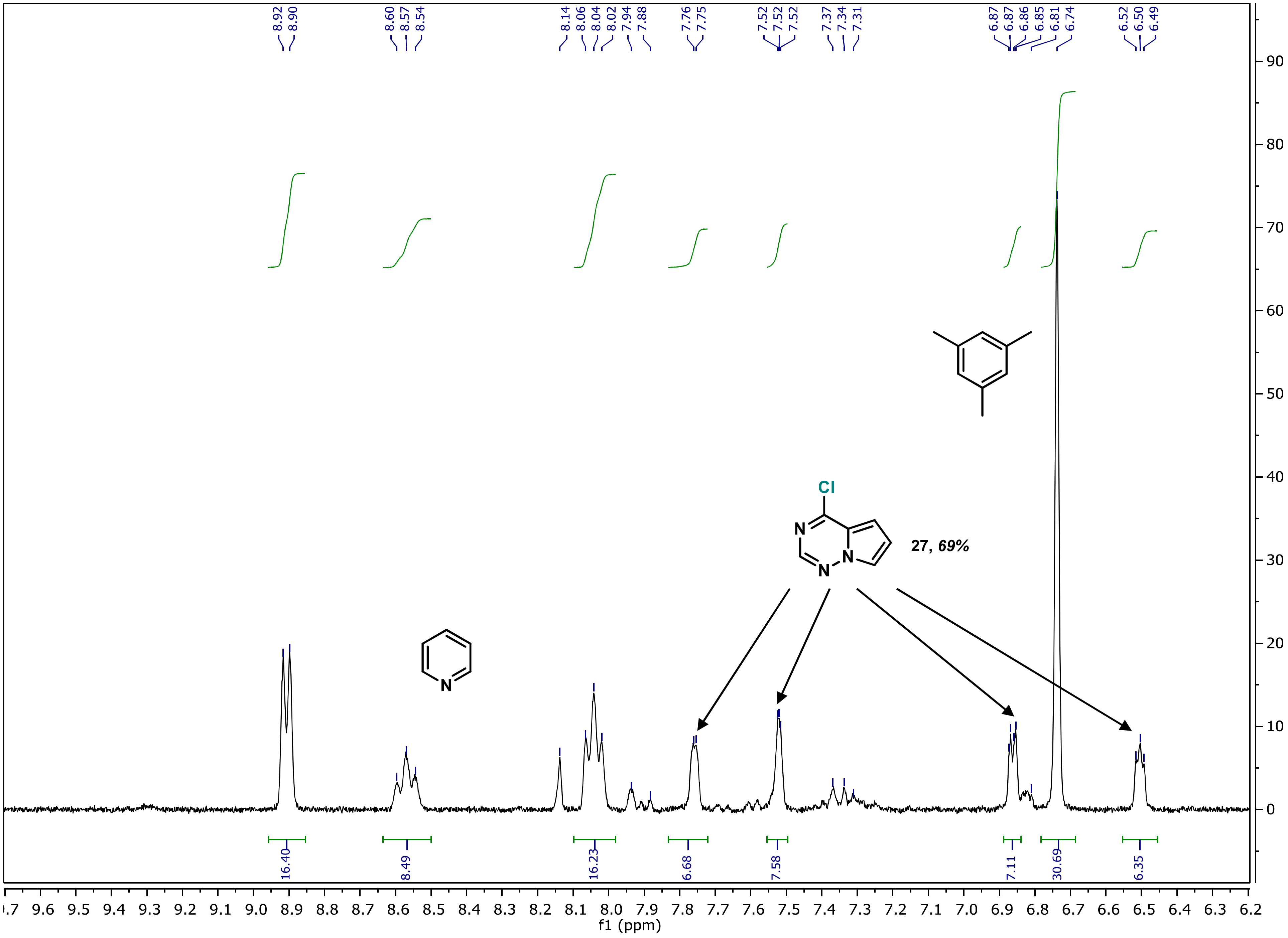

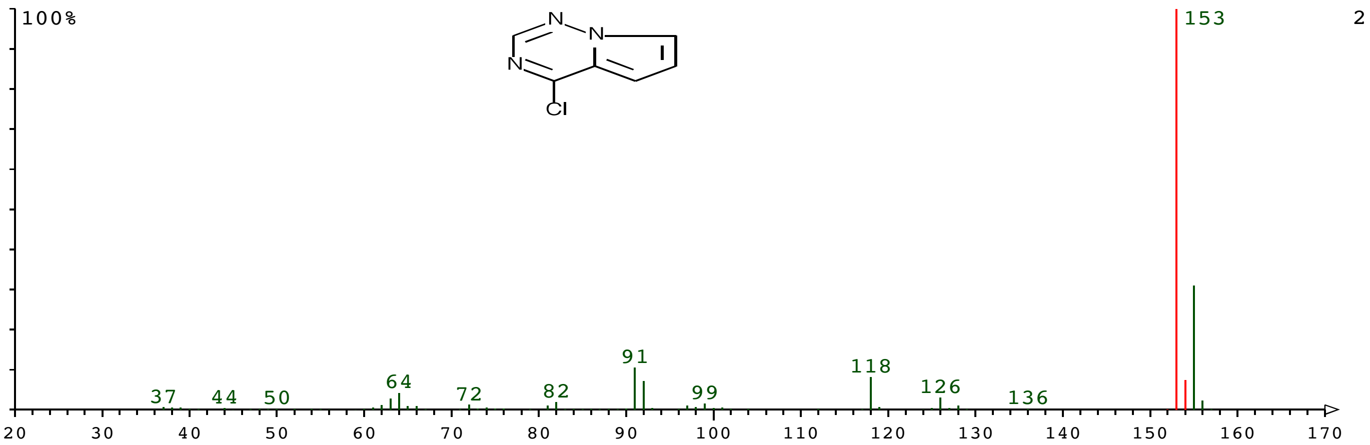

OE1052 51% MW:153/153.57 C6H4Cl1N3

36	0.01	64	4.17	91	10.44	136	0.15		
37	0.51	65	0.87	92	7.12	152	0.06		
38	0.48	66	0.86	93	0.38	153	100.00		
39	0.45	67	0.02	96	0.15	154	7.30		
40	0.10	71	0.04	97	0.93	155	30.85		
41	0.10	72	1.18	98	0.60	156	2.21		
44	0.38	73	0.23	99	1.43	157	0.04		
45	0.01	74	0.49	100	0.37				
47	0.09	75	0.18	101	0.42				
48	0.12	76	0.21	102	0.06				
49	0.15	79	0.12	104	0.04				
50	0.16	81	0.93	112	0.04				
52	0.09	82	1.79	117	0.11				
54	0.06	83	0.05	118	8.13				
55	0.02	84	0.02	119	0.54				
56	0.01	85	0.04	124	0.01				
59	0.01	86	0.10	125	0.40				
60	0.03	87	0.09	126	2.97				
61	0.44	88	0.06	127	0.29				
62	1.06	89	0.05	128	0.91				
63	2.68	90	0.25	129	0.04				

OE1052 51% MW:153/153.57 C6H4Cl1N3

Mass to be matched (m/z): 153.008950 Charge: 1

Mass Tolerance: ±0.005000
Restriction of atom numbers:

C	H	N	Cl
1-110	1-100	1-3	1-1

Number of calculated Formulas: 1

Formula	Diff.(ppm)	theor. m/z
C6 H4 N3 Cl1	-0.82	153.008824

9.10.2020
File: 149786a-00.raw
Analyse: GHC-GA-372-01
COP: Dr. Clement Ghiazza

Messung:	GC-MS
Ionisierung:	GC-EI
Spektrometer:	QExactiveGC
Säule:	MS 50 TX1+VS
Länge:	30+7
Temp.:	35-10-285-5
GC-Nr.:	-

Auswerter: Margold

^{1}H NMR

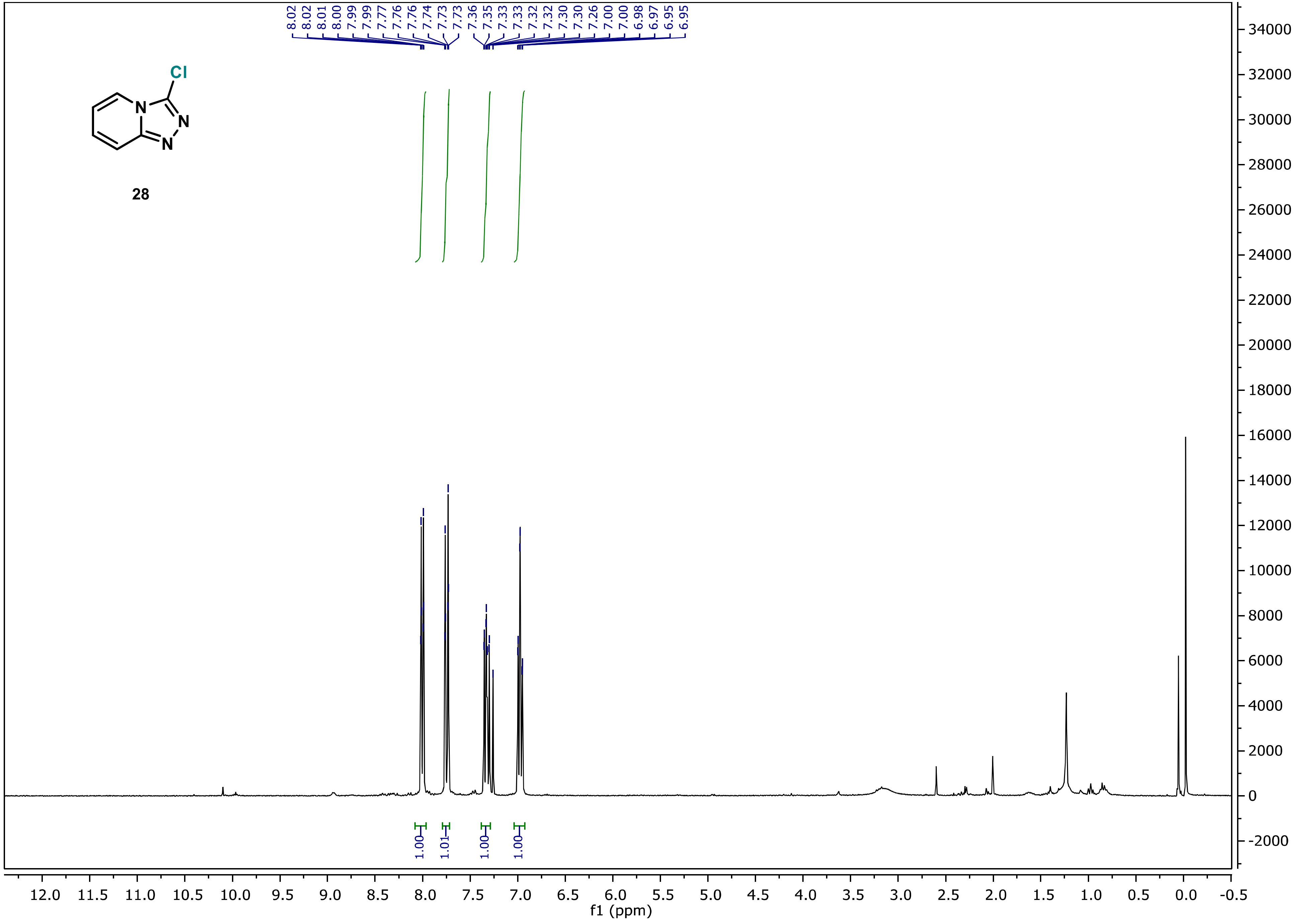
8.02
8.02
8.01
8.00
7.99
7.99
7.77
7.76
7.76
7.74
7.73
7.73
7.36
7.35
7.33
7.33
7.32
7.32
7.30
7.30
7.26
7.00
7.00
6.98
6.97
6.95
6.95
Cl
N
N
N
1.00
1.01
1.00
1.00
f1 (ppm)

28

^{13}C NMR

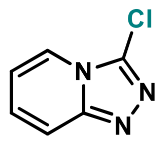

28

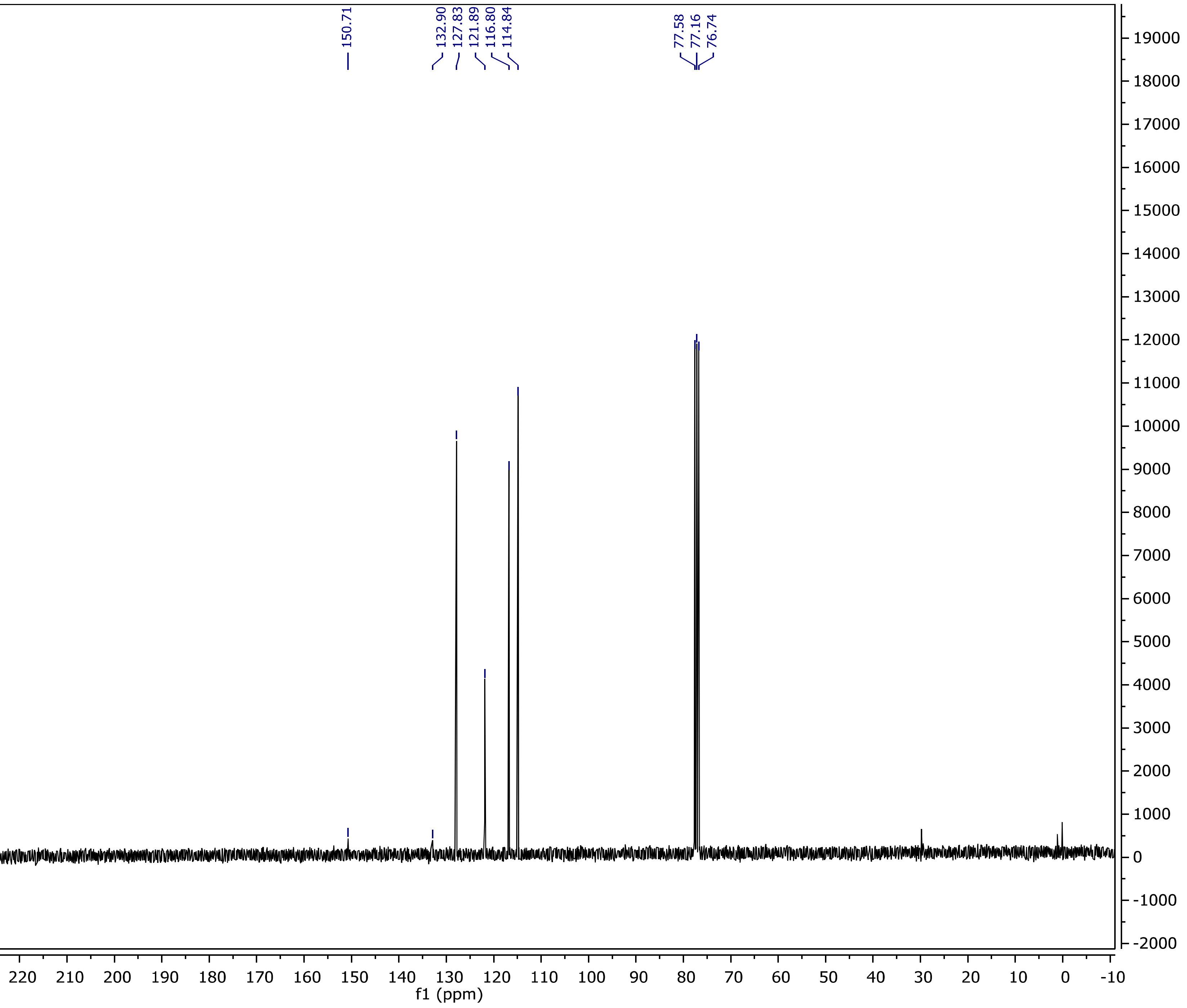
150.71
132.90
127.83
121.89
116.80
114.84
77.58
77.16
76.74
19000
18000
17000
16000
15000
14000
13000
12000
11000
10000
9000
8000
7000
6000
5000
4000
3000
2000
1000
0
-1000
-2000
270 260 250 240 230 220 210 200 190 180 170 160 150 140 130 120 110 100 90 80 70 60 50 40 30 20 10 0 -10
f1 (ppm)

Mass to be matched (m/z): 153.009070 Charge: 1

Mass Tolerance: ±0.005000
Restriction of atom numbers:

C	H	N	Cl
1-110	1-100	1-3	1-2

Number of calculated Formulas: 2

Formula	Diff.(ppm)	theor. m/z
C6 H4 N3 Cl1	-1.61	153.008824
C5 H9 N1 Cl2	10.36	153.010655

7.10.2020
File: 149750a-00.raw
Analyse: GHC-GA-370-01
COP: Dr. Clement Ghiazza

Messung:	GC-MS
Ionisierung:	GC-EI
Spektrometer:	QExactiveGC
Säule:	MS 50 TX1+VS
Länge:	30+7
Temp.:	35-10-285-5
GC-Nr.:	-

Auswerter: Margold

^{1}H NMR

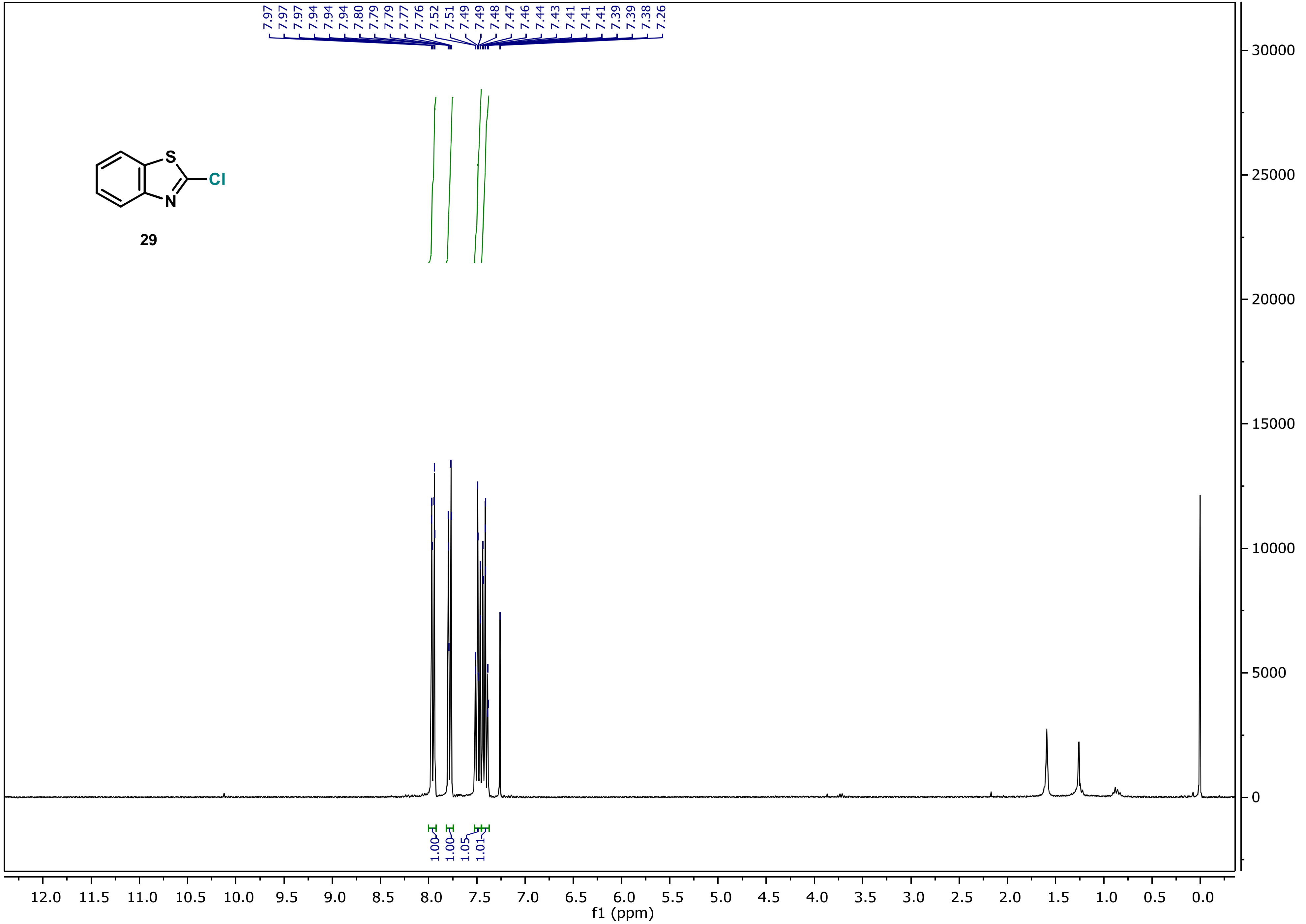

Cl
S
N
29
7.97
7.97
7.97
7.94
7.94
7.94
7.80
7.79
7.79
7.77
7.76
7.52
7.51
7.49
7.49
7.48
7.47
7.46
7.44
7.43
7.41
7.41
7.41
7.39
7.39
7.38
7.26
1.00
1.00
1.05
1.01
12.0
11.5
11.0
10.5
10.0
9.5
9.0
8.5
8.0
7.5
7.0
6.5
6.0
5.5
5.0
4.5
4.0
3.5
3.0
2.5
2.0
1.5
1.0
0.5
0.0
f1 (ppm)
30000
25000
20000
15000
10000
5000
0

^{1}H NMR

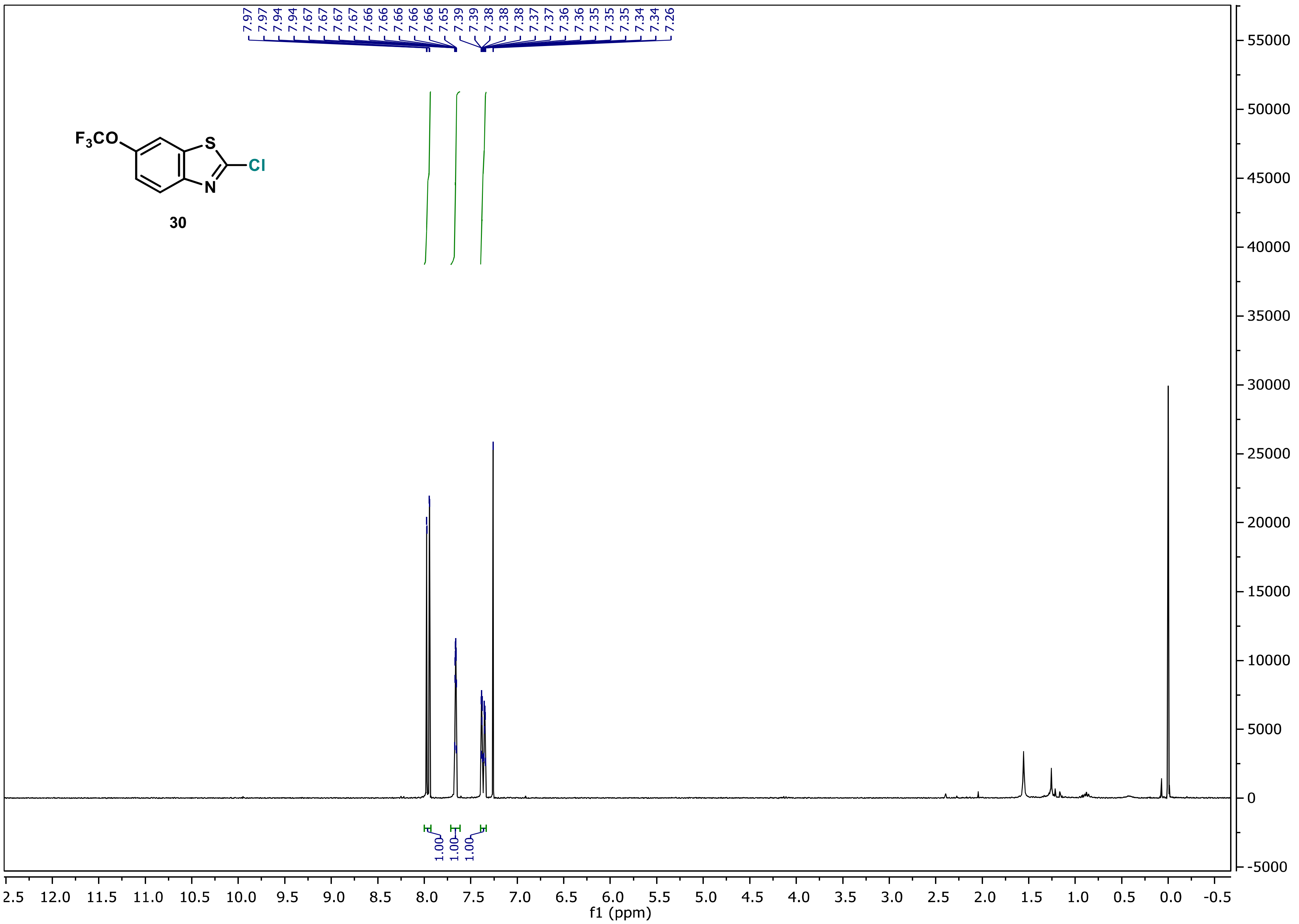

F3CO
S
N
Cl
7.97 7.97 7.94 7.94 7.67 7.67 7.67 7.67 7.66 7.66 7.66 7.66 7.66 7.65 7.39 7.39 7.38 7.38 7.38 7.37 7.37 7.36 7.36 7.35 7.35 7.35 7.34 7.34 7.26
1.00
1.00
1.00
f1 (ppm)

30

^{13}C NMR

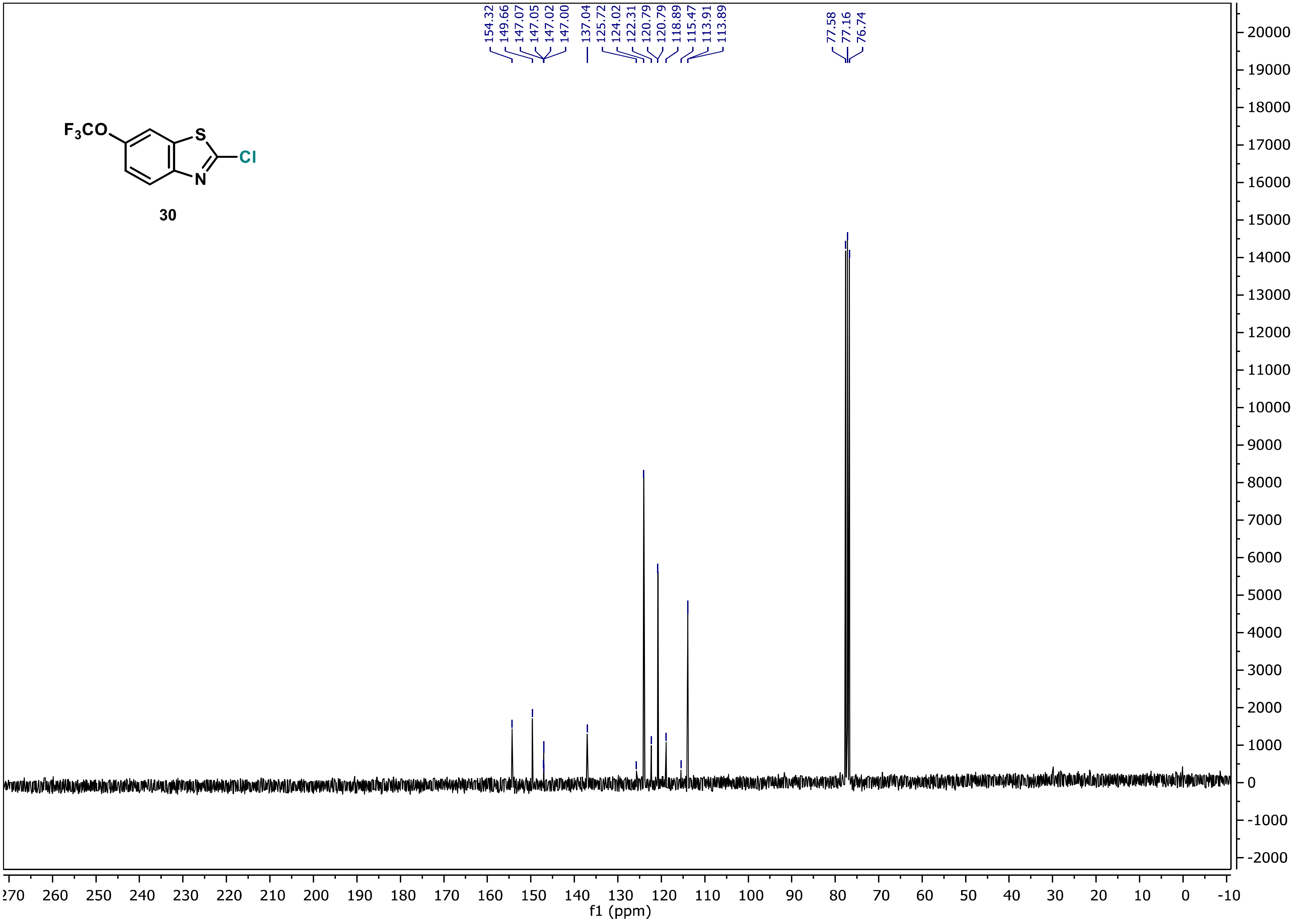
154.32
149.66
147.07
147.05
147.02
147.00
137.04
125.72
124.02
122.31
120.79
120.79
118.89
115.47
113.91
113.89
77.58
77.16
76.74
f1 (ppm)

30

^{19}F NMR

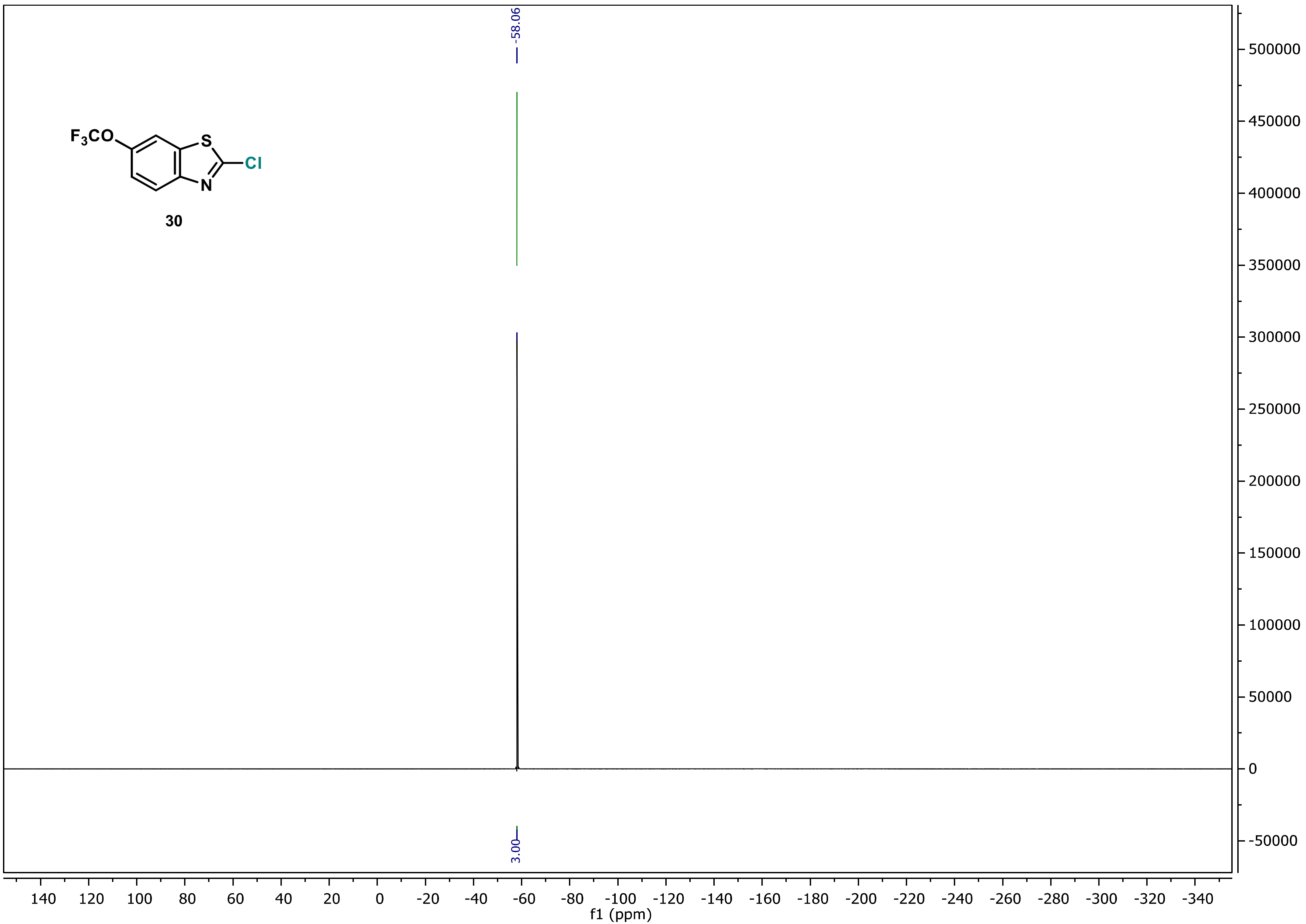

Mass to be matched (m/z): 252.957470 Charge: 1

Mass Tolerance: ±0.005000
Restriction of atom numbers:

C	H	N	O	S	Cl	F
1-100	1-100	1-5	1-10	1-1	1-1	3-3

Number of calculated Formulas: 2

Formula	Diff.(ppm)	theor. m/z
C8 H3 N1 O1 S1 Cl1 F3	-1.66	252.957050
C3 H3 N3 O3 S1 Cl1 F3	-17.56	252.953027

Datum:	6.08.2020
Analyse:	148532c-00
Sigel:	GHC-GA-262-01<br>COP: Dr. Clement Ghiazza
Method:	HR-MS
Ionis. :	GC-EI
Spectrometer:	Q-Exactive
Auswerter:	Marcus, Tel:2243

suggestion:
C8H3N1O1S1Cl1F3   MW: 253

^{1}H NMR

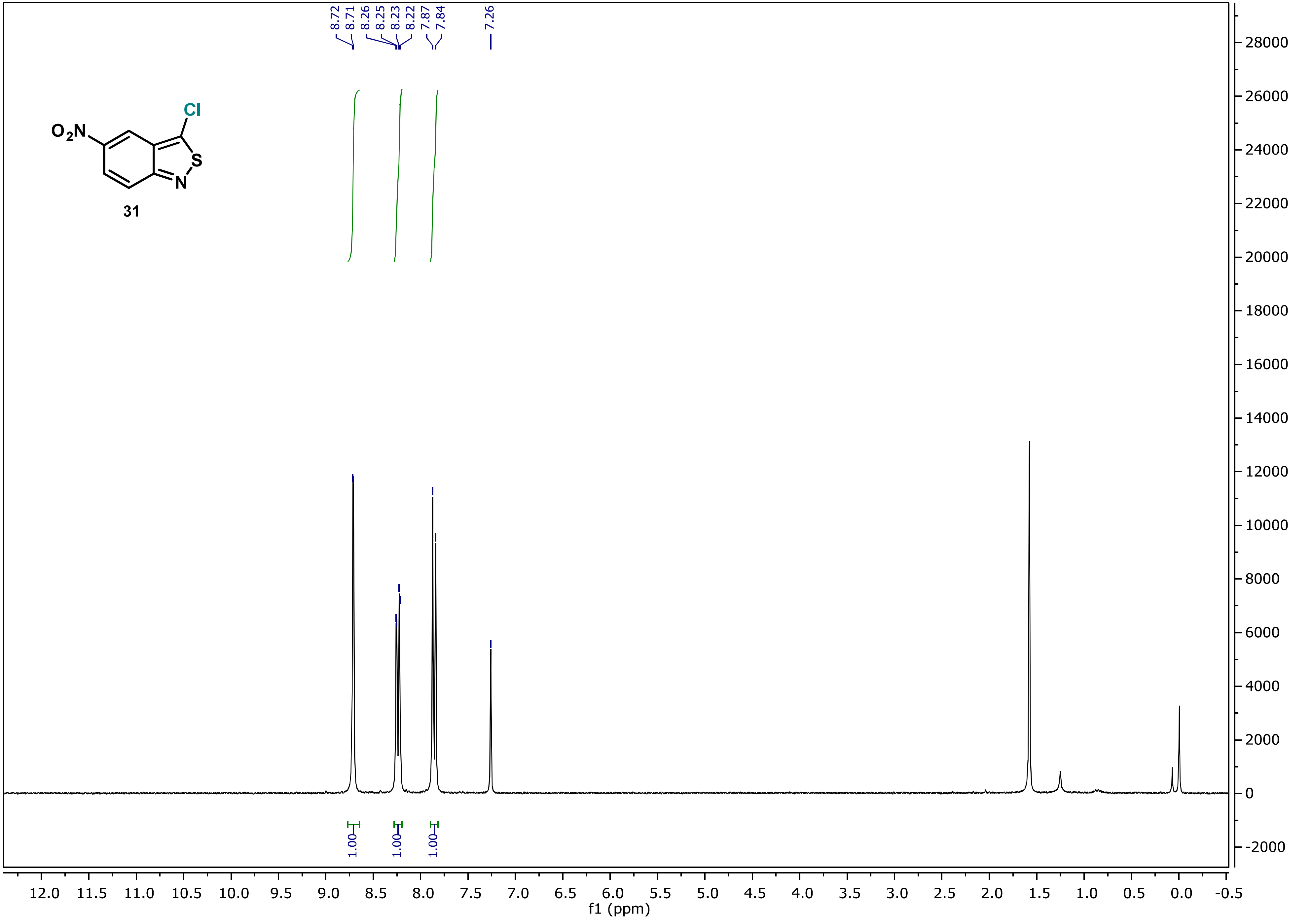

O2N
Cl
S
N
31
8.72
8.71
8.26
8.25
8.23
8.22
7.87
7.84
7.26
1.00
1.00
1.00
f1 (ppm)

^{13}C NMR

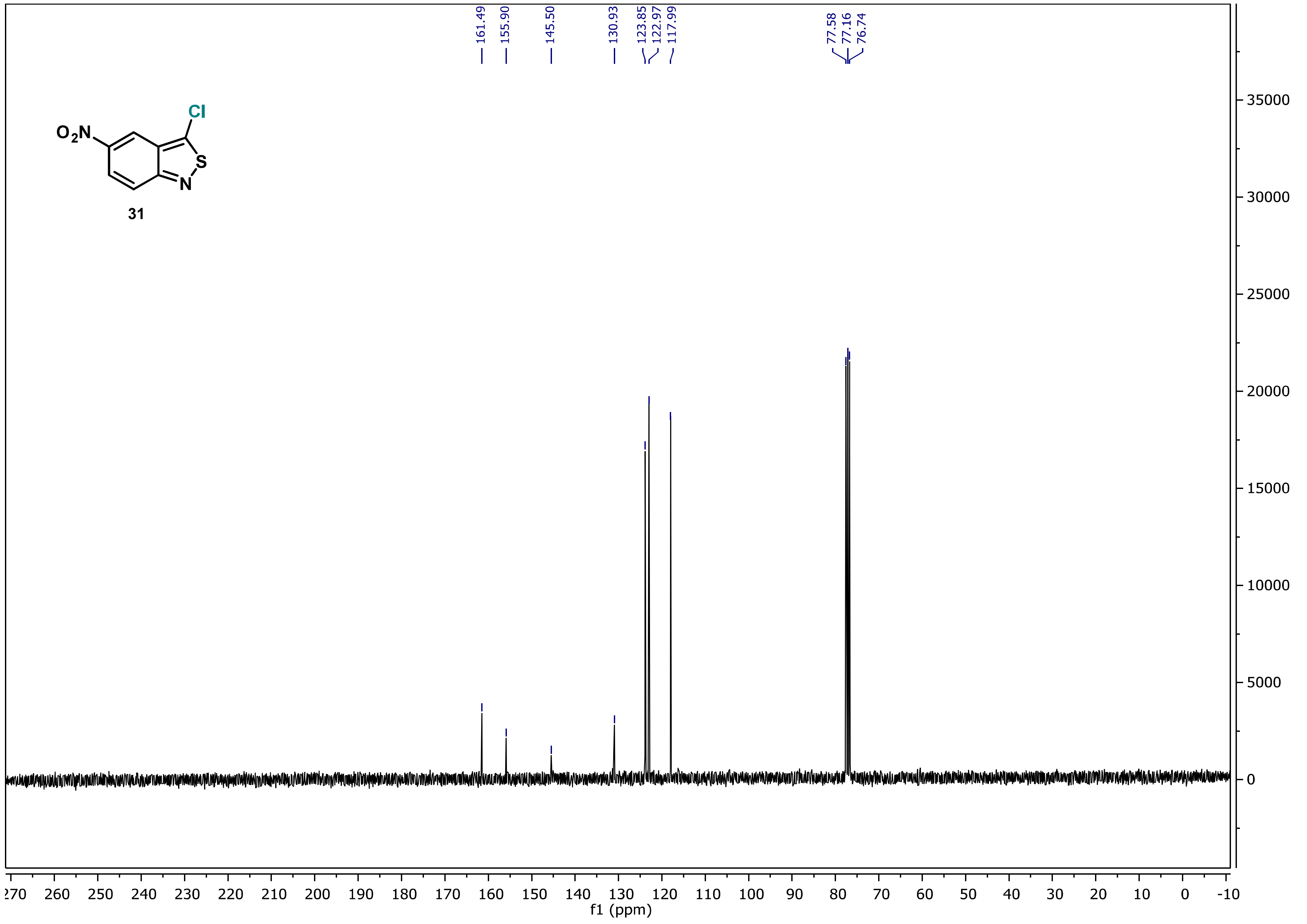
161.49
155.90
145.50
130.93
123.85
122.97
117.99
77.58
77.16
76.74
O2N
Cl
S
N
31
35000
30000
25000
20000
15000
10000
5000
0
270
260
250
240
230
220
210
200
190
180
170
160
150
140
130
120
110
100
90
80
70
60
50
40
30
20
10
0
-10
f1 (ppm)

Mass to be matched (m/z): 214.967750 Charge: 1

Mass Tolerance: ±0.005000
Restriction of atom numbers:

C	H	N	O	S	Cl
1-100	1-100	1-5	1-10	1-1	1-1

Number of calculated Formulas: 4

Formula	Diff.(ppm)	theor. m/z
C7 H4 N2 O2 S1 Cl1	-0.45	214.967653
C5 H2 N5 O1 S1 Cl1	-6.70	214.966309
C4 H6 N1 O5 S1 Cl1	-12.91	214.964974
C2 H4 N4 O4 S1 Cl1	-19.16	214.963631

Datum:	24.07.2020
Analyse:	148347c-00
Sigel:	GHC-GA-192-01<br>COP: Dr. Clement Ghiazza
Method:	HR-MS
Ionis. :	ESIpos
solvent :	CH2Cl2 + CH3OH
Spectrometer:	Exactive
Auswerter:	Marcus, Tel:2243

suggestion:
C7H3N2O2S1Cl1 MW: 214

Characteristic Ions:
215 = [214 + H]

^{1}H NMR

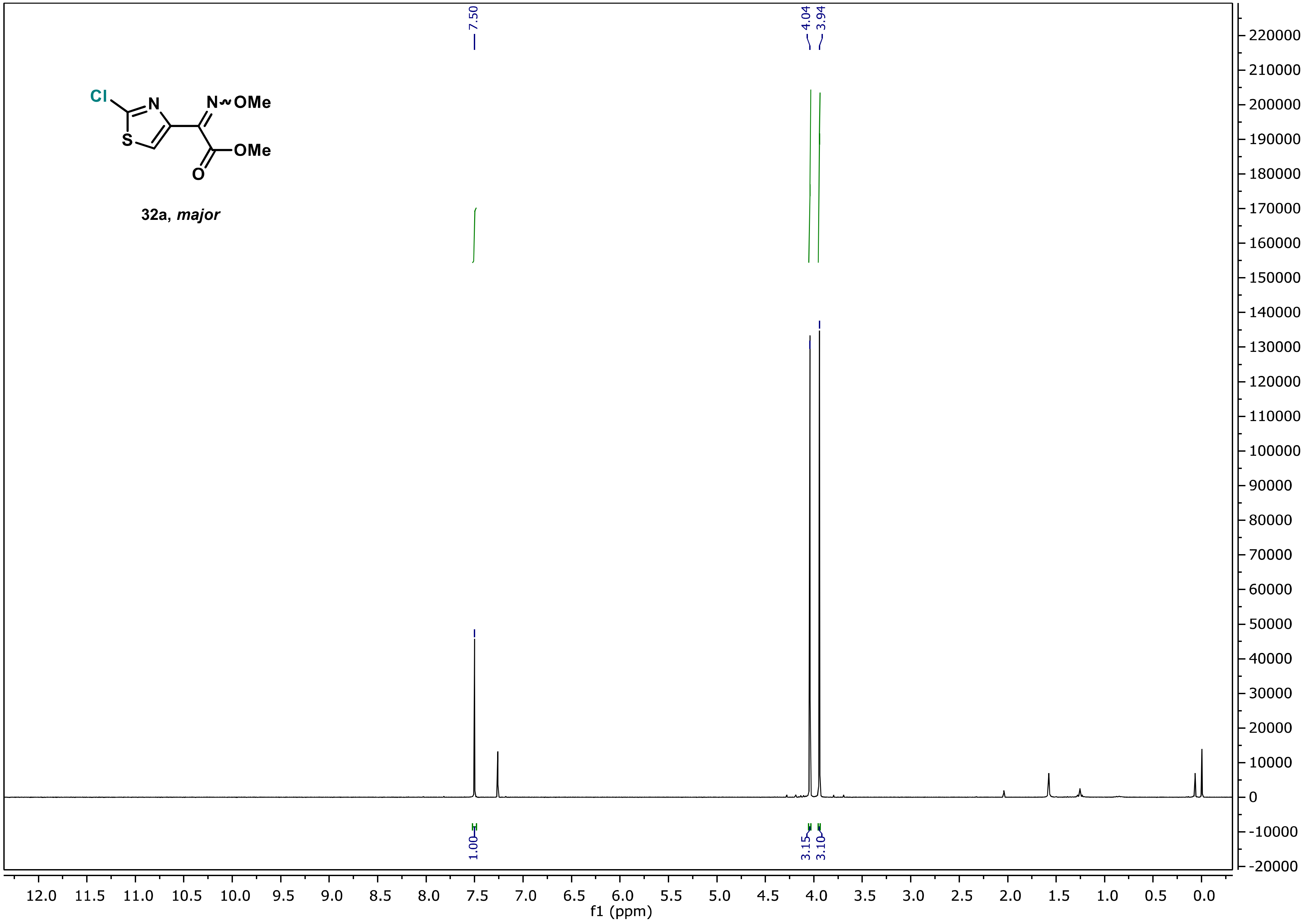

32a, *major*

^{13}C NMR

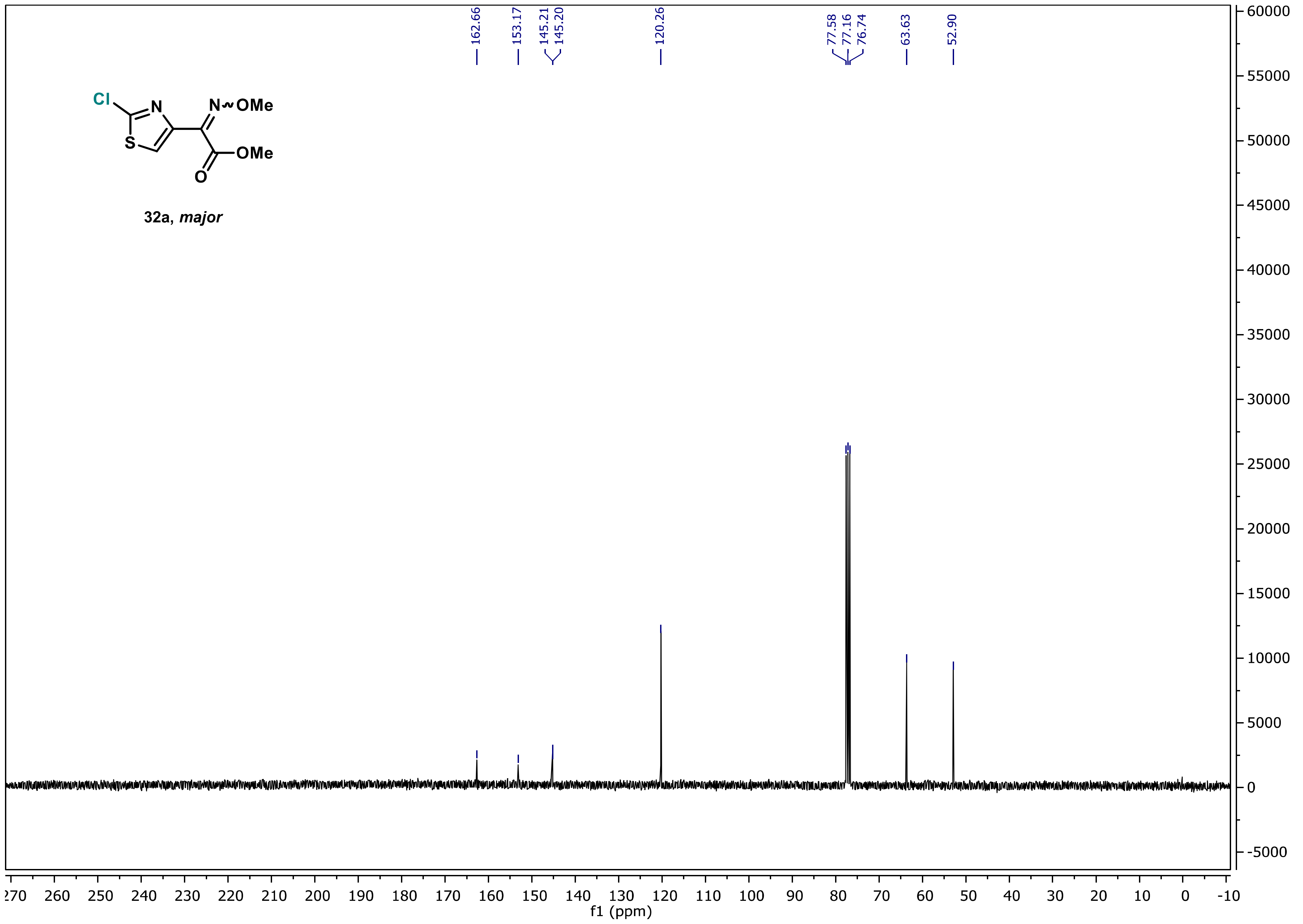

32a, *major*

^{1}H NMR

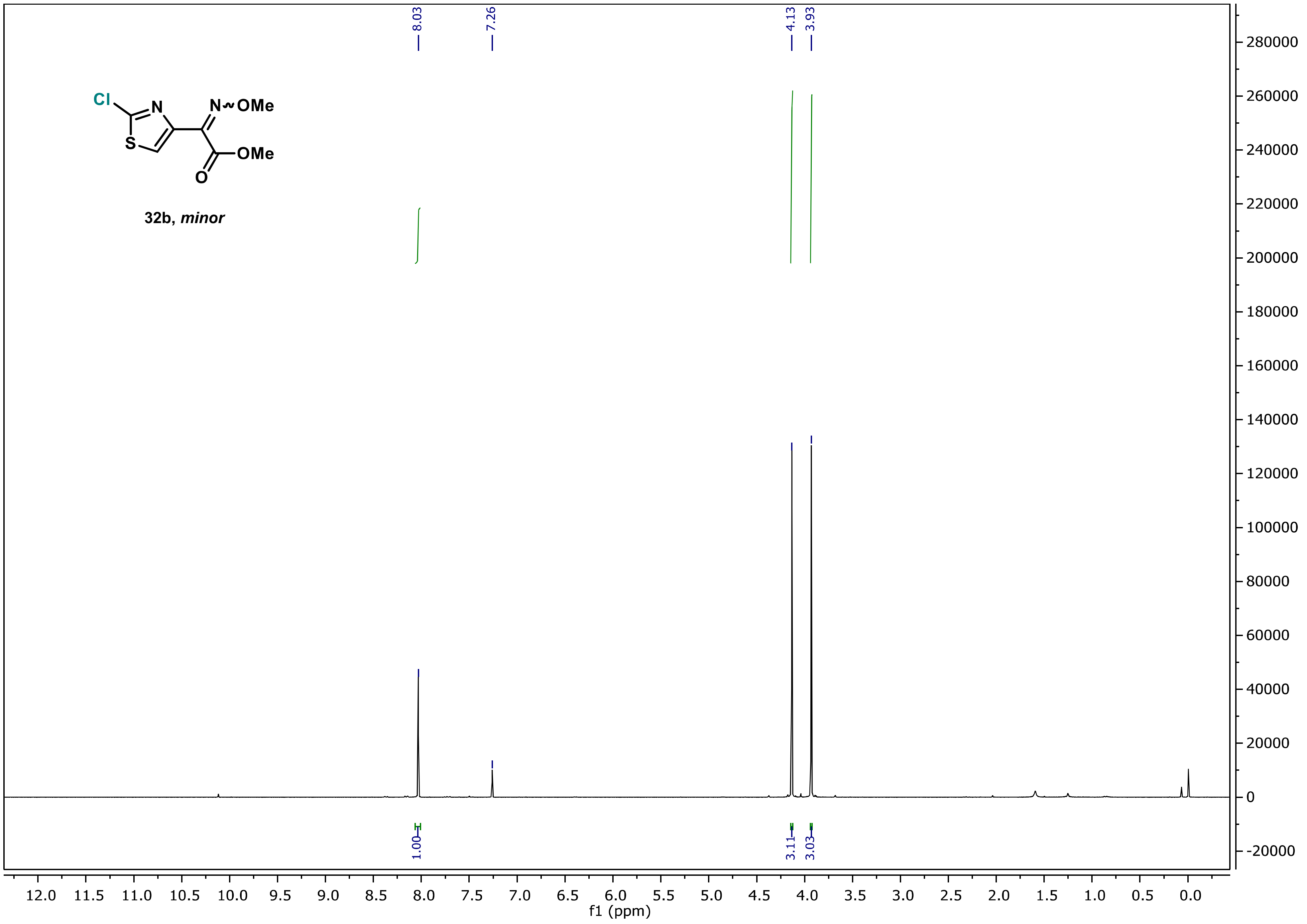

^{13}C NMR

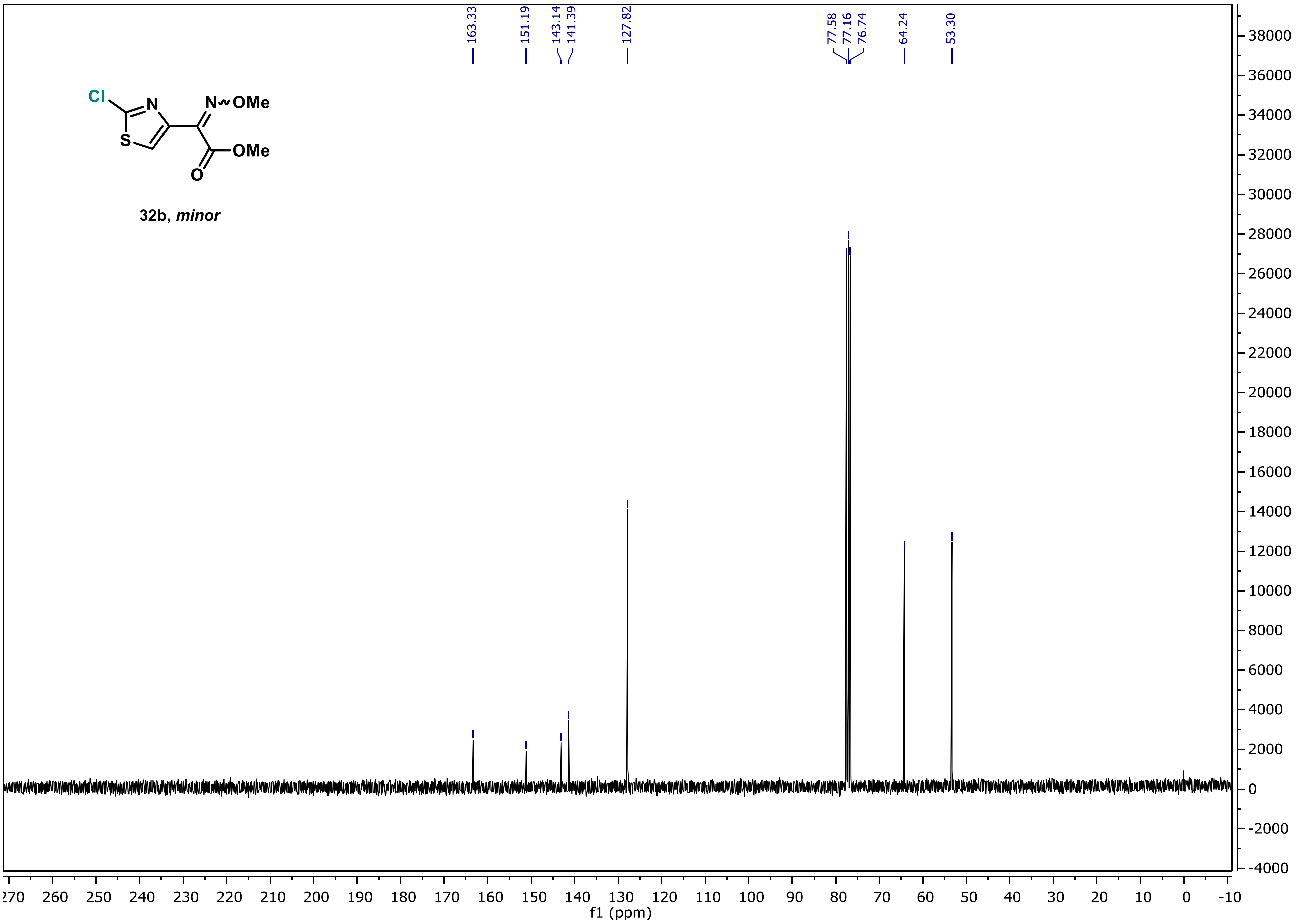

Cl
N
S
N~OMe
OMe
O
32b, *minor*
163.33
151.19
143.14
141.39
127.82
77.58
77.16
76.74
64.24
53.30
f1 (ppm)

*** Angegebene Mol.-Gewichte u. Massenzahlen basieren auf dem häufigsten Isotop der Elemente ***

MassLib

Mass to be matched (m/z): 256.975680 Charge: 1

Mass Tolerance: ±0.020000
Restriction of atom numbers:

C	H	N	O	S	Cl	Na
1-100	1-100	1-5	1-3	1-1	1-1	1-1

Number of calculated Formulas: 6

Formula	Diff.(ppm)	theor. m/z
C7 H7 N2 O3 S1 Cl1 Na1	0.51	256.975812
C5 H5 N5 O2 S1 Cl1 Na1	-4.72	256.974468
C11 H5 N1 O1 S1 Cl1 Na1	-32.77	256.967258
C6 H7 N4 O2 S1 Cl1 Na1	44.22	256.987044
C6 H5 N3 O3 S1 Cl1 Na1	-48.43	256.963236
C8 H9 N1 O3 S1 Cl1 Na1	49.45	256.988388

Datum:	22.07.2020
Analyse:	148260c-00
Sigel:	GHC-GA-209-02
	COP: Dr. Clement Ghiazza
Method:	HR-MS
Ionis. :	ESIpos
solvent :	CH2Cl2 + CH3OH
Spectrometer:	Exactive
Auswerter:	Marcus, Tel:2243

suggestion:
C7H7N2O3S1Cl1   MW: 234

Characteristic Ions:
257 = [234 + Na]

^{1}H NMR

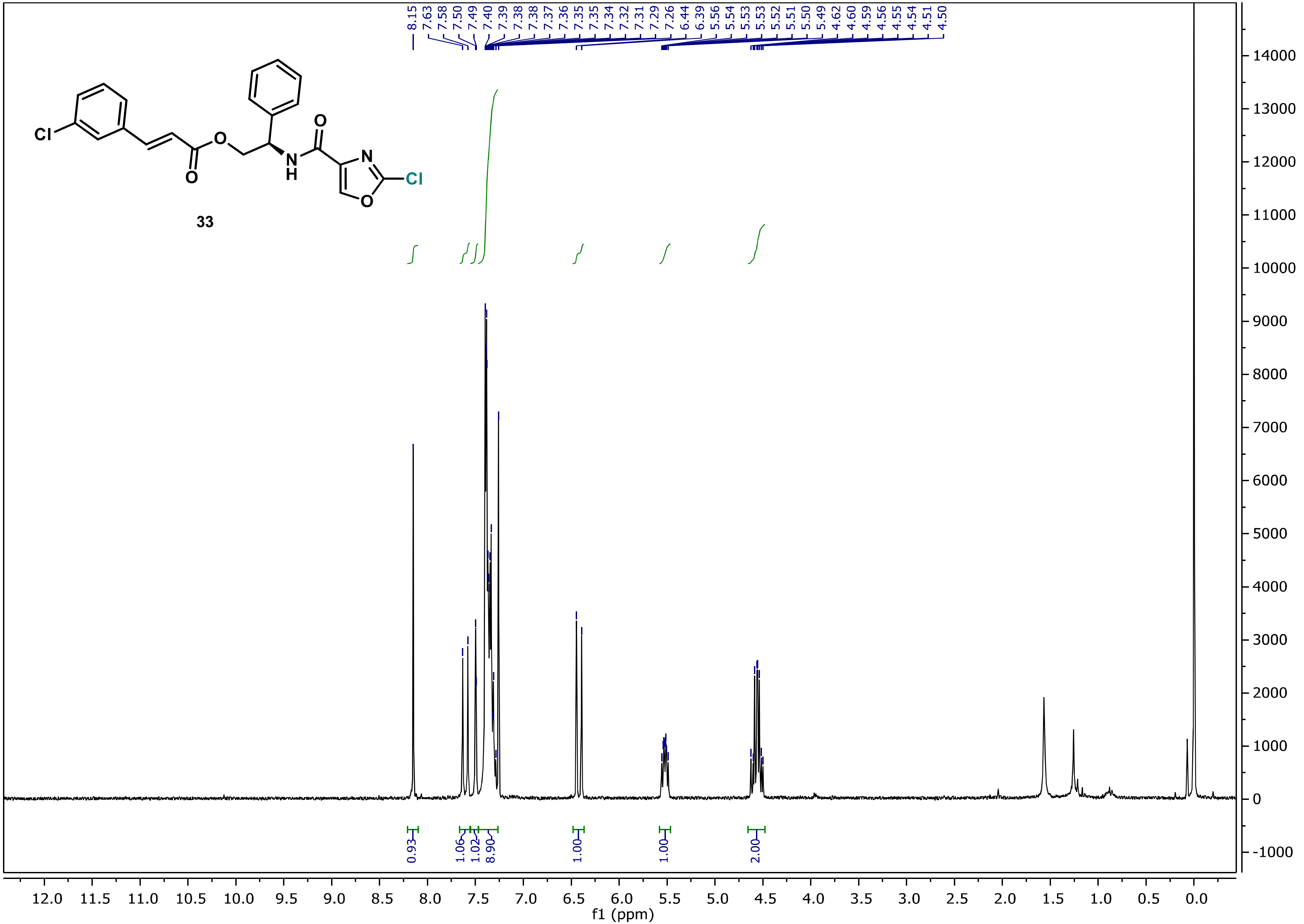
33
8.15
7.63
7.58
7.50
7.49
7.40
7.39
7.38
7.38
7.37
7.36
7.35
7.35
7.34
7.32
7.31
7.29
7.26
6.44
6.39
5.56
5.54
5.53
5.53
5.52
5.51
5.50
5.49
4.62
4.60
4.59
4.56
4.55
4.54
4.51
4.50
0.93
1.06
1.02
8.90
1.00
1.00
2.00
f1 (ppm)

^{13}C NMR

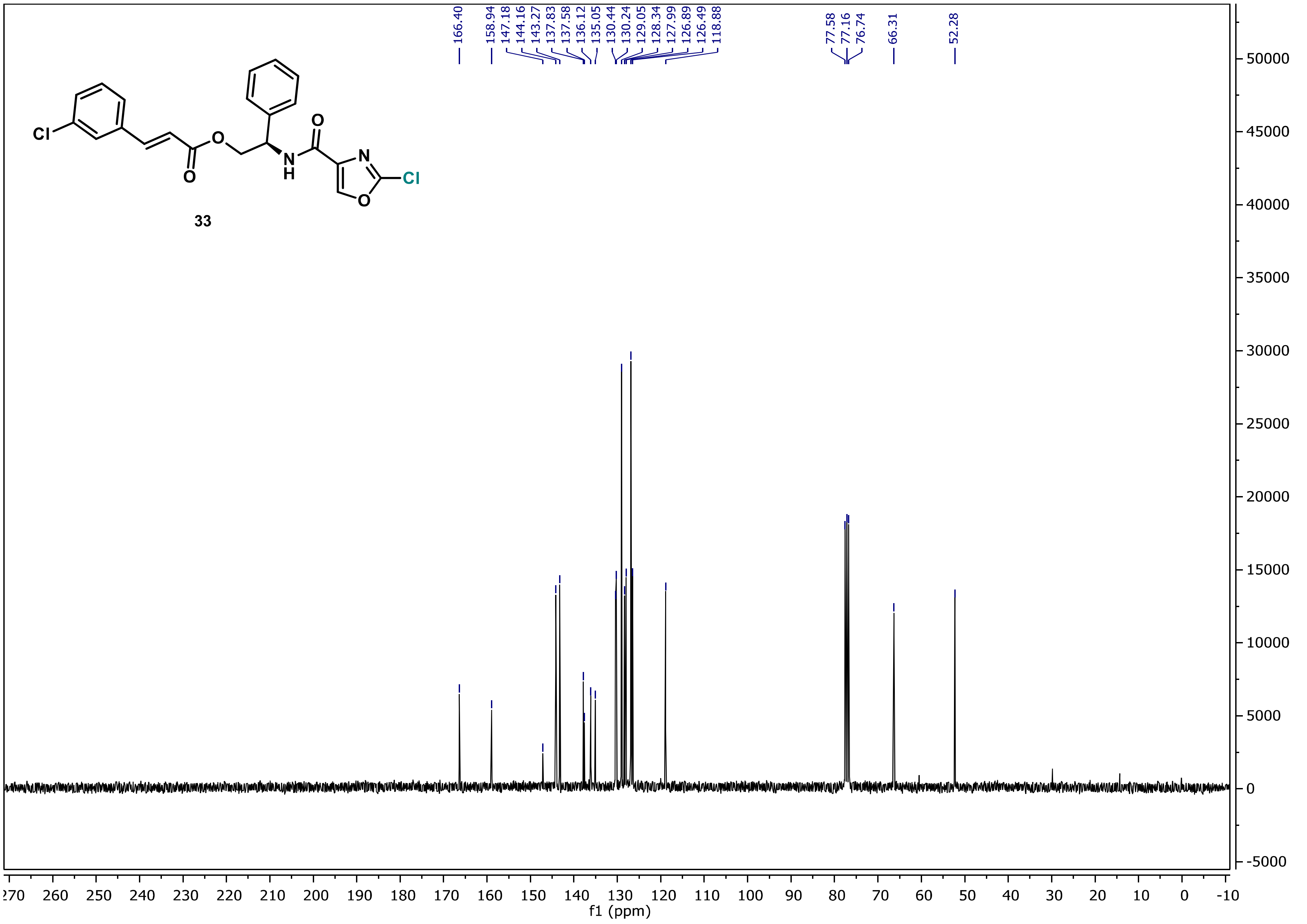

166.40
158.94
147.18
144.16
143.27
137.83
137.58
136.12
135.05
130.44
130.24
129.05
128.34
127.99
126.89
126.49
118.88
77.58
77.16
76.74
66.31
52.28
Cl
O
N
H
Cl
f1 (ppm)

33

*** Angegebene Mol.-Gewichte u. Massenzahlen basieren auf dem häufigsten Isotop der Elemente ***

MassLib

Mass to be matched (m/z): 453.038660 Charge: 1

Mass Tolerance: ±0.005000
Restriction of atom numbers:

C	H	N	O	Cl	Na
1-100	1-100	1-5	1-10	2-2	1-1

Number of calculated Formulas: 6

Formula	Diff.(ppm)	theor. m/z
C21 H16 N2 O4 Cl2 Na1	-1.61	453.037932
C24 H14 N3 O1 Cl2 Na1	4.31	453.040611
C19 H14 N5 O3 Cl2 Na1	-4.57	453.036589
C18 H18 N1 O7 Cl2 Na1	-7.52	453.035253
C12 H18 N5 O8 Cl2 Na1	8.40	453.042464
C16 H16 N4 O6 Cl2 Na1	-10.49	453.033910

Datum:	15.10.2020
Analyse:	149874c-00
Sigel:	GHC-AA-058-01
	COP: Dr. Clement Ghiazza
Method:	HR-MS
Ionis. :	ESIpos
solvent :	CH3OH
Spectrometer:	Exactive
Auswerter:	Marcus, Tel:2243

suggestion:
C21H16N2O4Cl2 MW: 430

Characteristic Ions:
453 = [430 + Na]

^{1}H NMR

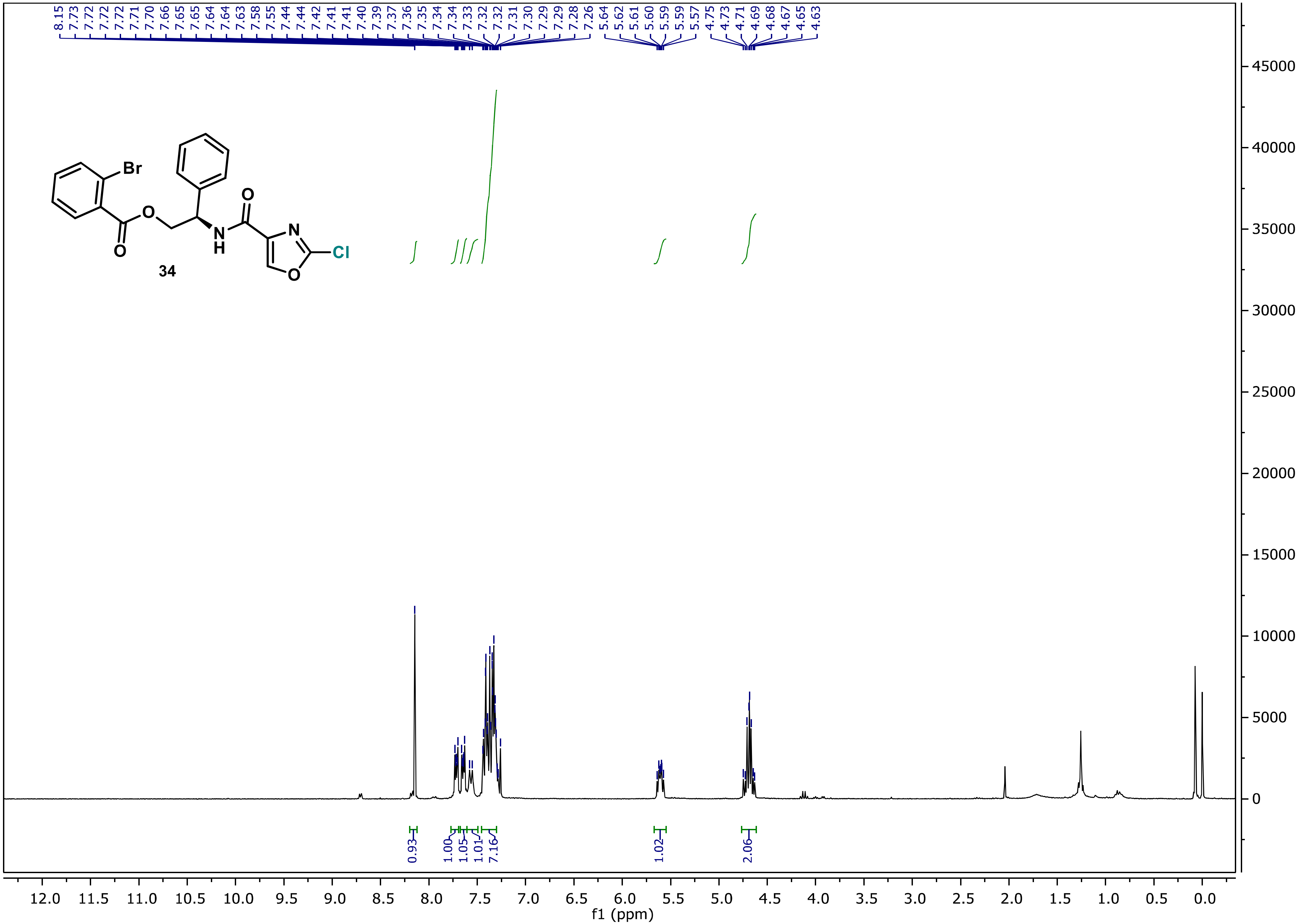
8.15
7.73
7.72
7.72
7.72
7.71
7.70
7.66
7.65
7.65
7.64
7.64
7.63
7.58
7.55
7.44
7.44
7.42
7.41
7.41
7.40
7.39
7.37
7.36
7.35
7.34
7.34
7.33
7.32
7.32
7.31
7.30
7.29
7.29
7.28
7.26
5.64
5.62
5.61
5.60
5.59
5.59
5.57
4.75
4.73
4.71
4.69
4.68
4.67
4.65
4.63
Br
O
O
N
H
O
N
O
Cl
34
0.93
1.00
1.05
1.01
7.16
1.02
2.06
12.0
11.5
11.0
10.5
10.0
9.5
9.0
8.5
8.0
7.5
7.0
6.5
6.0
5.5
5.0
4.5
4.0
3.5
3.0
2.5
2.0
1.5
1.0
0.5
0.0
f1 (ppm)
45000
40000
35000
30000
25000
20000
15000
10000
5000
0

^{13}C NMR

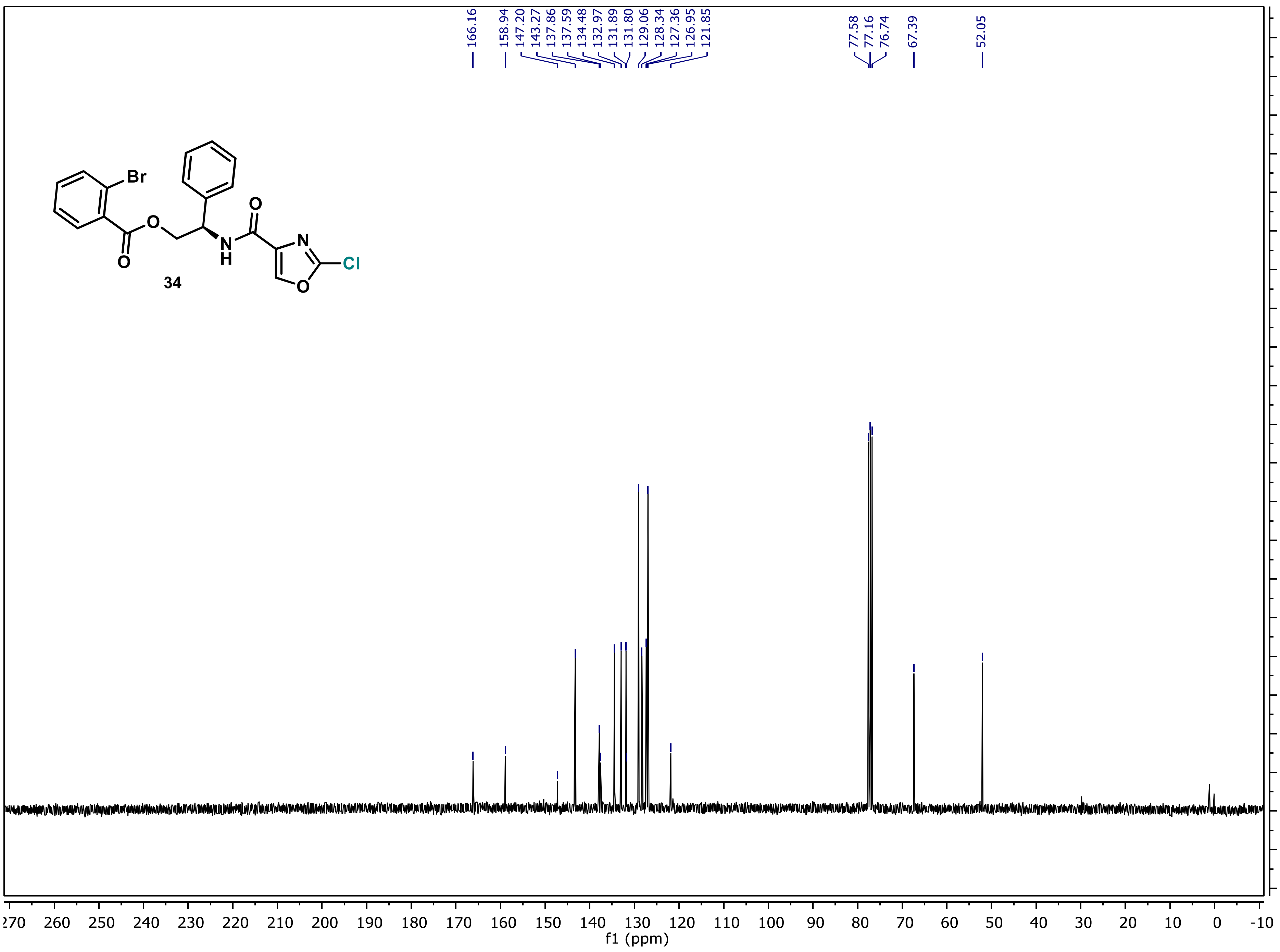
166.16
158.94
147.20
143.27
137.86
137.59
134.48
132.97
131.89
131.80
129.06
128.34
127.36
126.95
121.85
77.58
77.16
76.74
67.39
52.05
Br
O
N
H
N
Cl
O
34
40000
38000
36000
34000
32000
30000
28000
26000
24000
22000
20000
18000
16000
14000
12000
10000
8000
6000
4000
2000
0
-2000
-4000
270 260 250 240 230 220 210 200 190 180 170 160 150 140 130 120 110 100 90 80 70 60 50 40 30 20 10 0 -10
f1 (ppm)

*** Angegebene Mol.-Gewichte u. Massenzahlen basieren auf dem häufigsten Isotop der Elemente ***

MassLib

Mass to be matched (m/z): 470.972260 Charge: 1

Mass Tolerance: ±0.005000

Restriction of atom numbers:

C	H	N	O	Br	Cl	Na
1-100	1-100	1-5	1-10	1-1	1-1	1-1

Number of calculated Formulas: 6

Formula	Diff.(ppm)	theor. m/z
C19 H14 N2 O4 Br1 Cl1 Na1	-1.02	470.971780
C17 H12 N5 O3 Br1 Cl1 Na1	-3.87	470.970436
C22 H12 N3 O1 Br1 Cl1 Na1	4.67	470.974458
C16 H16 N1 O7 Br1 Cl1 Na1	-6.71	470.969101
C10 H16 N5 O8 Br1 Cl1 Na1	8.60	470.976311
C14 H14 N4 O6 Br1 Cl1 Na1	-9.56	470.967757

Datum: 15.10.2020
Analyse: 149897d-00

Sigel: GHC-AA-061-01
COP: Dr. Clement Ghiazza

Method: HR-MS
Ionis. : ESIpos
solvent : CH3OH
Spectrometer: Exactive

Auswerter: Marcus, Tel:2243

suggestion:
C19H14N2O4Br1Cl1 MW: 448

Characteristic Ions:
471 = [448 + Na]

^{1}H NMR

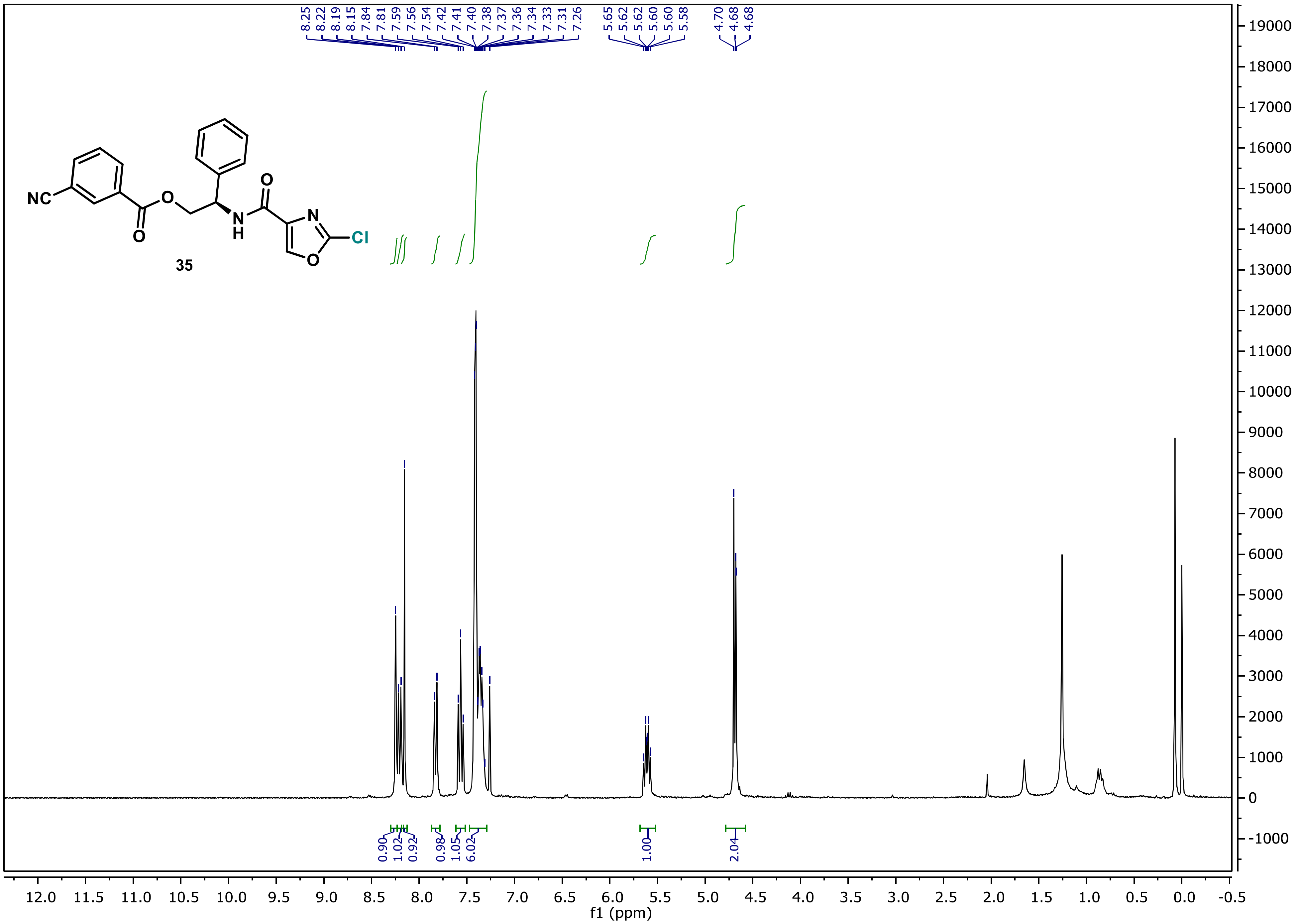

NC
O
O
N
H
O
N
O
Cl
35
8.25 8.22 8.19 8.15 7.84 7.81 7.59 7.56 7.54 7.42 7.41 7.40 7.38 7.37 7.36 7.34 7.33 7.31 7.26
5.65 5.62 5.62 5.60 5.60 5.58
4.70 4.68 4.68
0.90 1.02 0.92 0.98 1.05 6.02 1.00 2.04
f1 (ppm)

^{13}C NMR

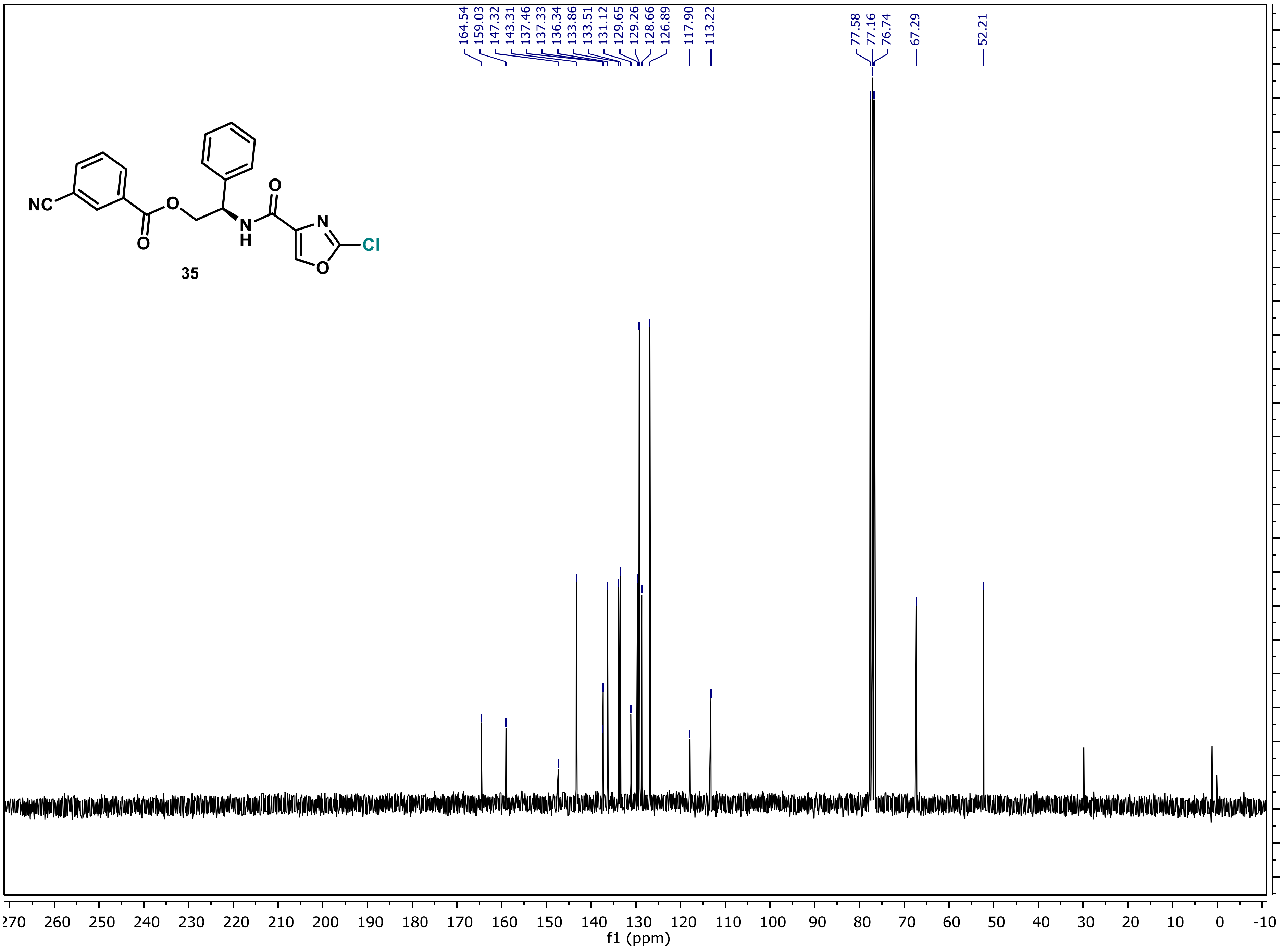

164.54
159.03
147.32
143.31
137.46
137.33
136.34
133.86
133.51
131.12
129.65
129.26
128.66
126.89
117.90
113.22
77.58
77.16
76.74
67.29
52.21
f1 (ppm)
35

Mass to be matched (m/z): 418.056640 Charge: 1

Mass Tolerance: ±0.005000
Restriction of atom numbers:

C	H	N	O	Cl	Na
1-110	1-100	1-3	1-4	1-2	1-1

Number of calculated Formulas: 4

Formula	Diff.(ppm)	theor. m/z
C20 H14 N3 O4 Cl1 Na1	-0.33	418.056503
C19 H19 N1 O4 Cl2 Na1	4.05	418.058334
C25 H14 N1 O2 Cl1 Na1	9.29	418.060526
C22 H17 N2 O1 Cl2 Na1	10.46	418.061013

13.10.2020
File: 149822c-00

Analyse: GHC-AA-056-01
COP: Dr. Clement Ghiazza

Messung: HRMS ESIpos
Lösemittel: CH3OH
Spektrometer: Exactive

Auswerter: Kohler (2243)

Suggestion:
C20h14N3O4Cl1 MW 395

Characteristicial ions:
418 = [395 + Na]+

^{1}H NMR

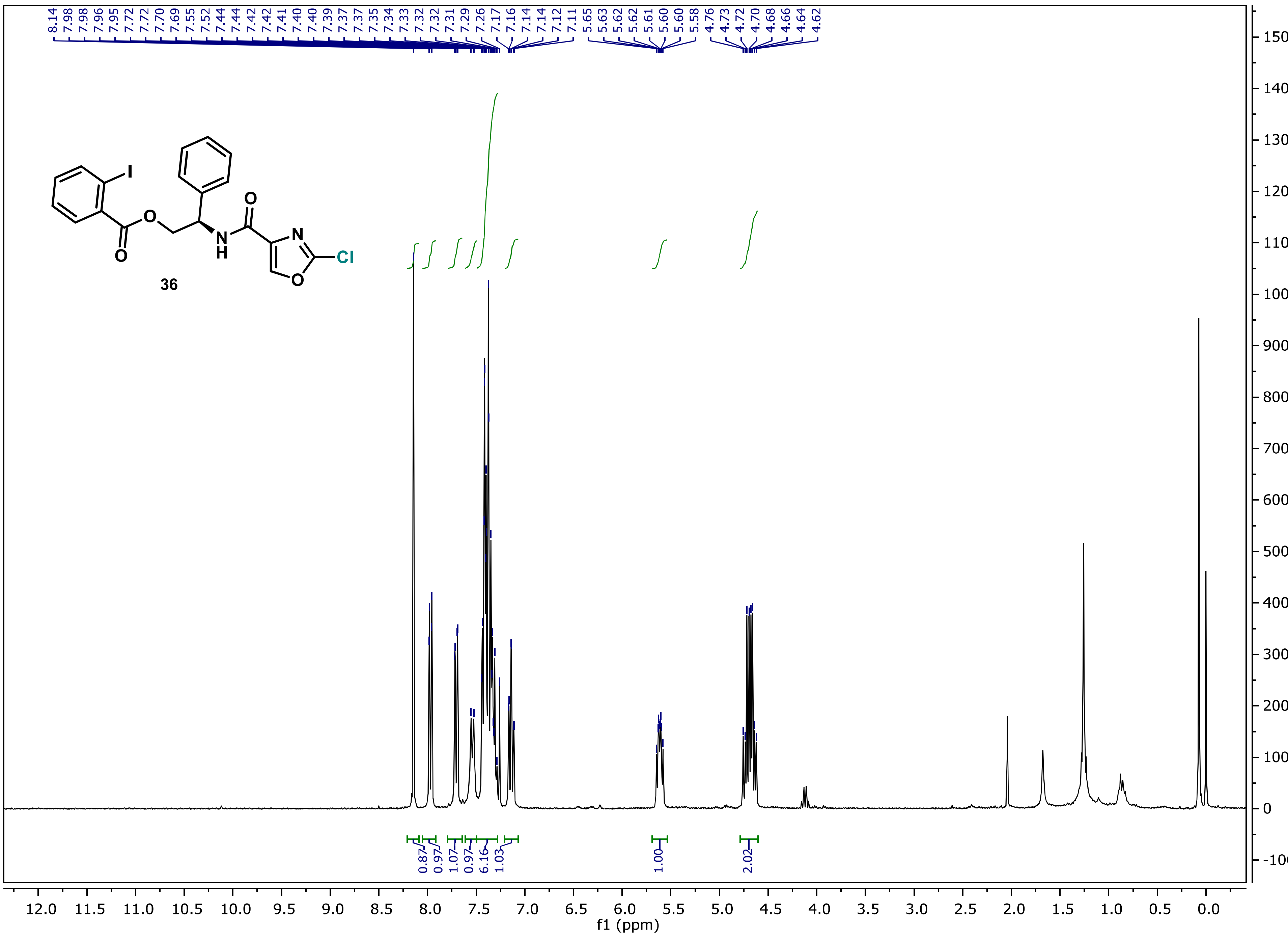

8.14 7.98 7.98 7.96 7.95 7.72 7.72 7.70 7.69 7.55 7.52 7.44 7.44 7.42 7.42 7.41 7.40 7.40 7.39 7.37 7.37 7.35 7.34 7.33 7.32 7.32 7.31 7.29 7.26 7.17 7.16 7.14 7.14 7.12 7.11 5.65 5.63 5.62 5.62 5.61 5.60 5.60 5.58 4.76 4.73 4.72 4.70 4.68 4.66 4.64 4.62
I
O
N
H
Cl
36
0.87
0.97
1.07
0.97
6.16
1.03
1.00
2.02
15000
14000
13000
12000
11000
10000
9000
8000
7000
6000
5000
4000
3000
2000
1000
0
-1000
12.0 11.5 11.0 10.5 10.0 9.5 9.0 8.5 8.0 7.5 7.0 6.5 6.0 5.5 5.0 4.5 4.0 3.5 3.0 2.5 2.0 1.5 1.0 0.5 0.0
f1 (ppm)

^{13}C NMR

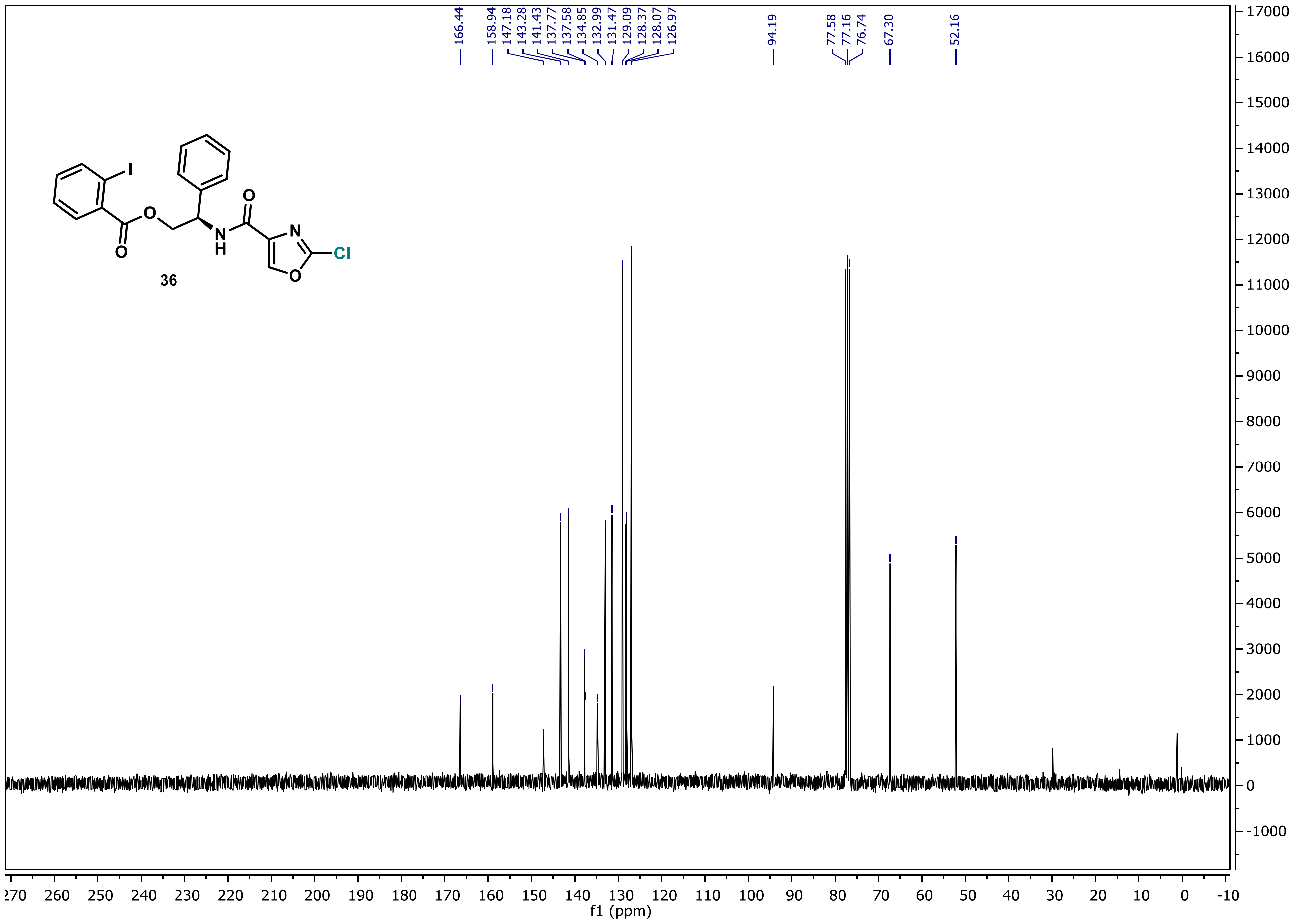
166.44
158.94
147.18
143.28
141.43
137.77
137.58
134.85
132.99
131.47
129.09
128.37
128.07
126.97
94.19
77.58
77.16
76.74
67.30
52.16
36
f1 (ppm)

*** Angegebene Mol.-Gewichte u. Massenzahlen basieren auf dem häufigsten Isotop der Elemente ***

MassLib

Mass to be matched (m/z): 518.957980 Charge: 1

Mass Tolerance: ±0.005000
Restriction of atom numbers:

C	H	N	O	Cl	I	Na
1-100	1-100	1-5	1-10	1-1	1-1	1-1

Number of calculated Formulas: 6

Formula	Diff.(ppm)	theor. m/z
C19 H14 N2 O4 Cl1 I1 Na1	-0.15	518.957902
C17 H12 N5 O3 Cl1 I1 Na1	-2.74	518.956558
C22 H12 N3 O1 Cl1 I1 Na1	5.01	518.960581
C16 H16 N1 O7 Cl1 I1 Na1	-5.31	518.955223
C14 H14 N4 O6 Cl1 I1 Na1	-7.90	518.953879
C10 H16 N5 O8 Cl1 I1 Na1	8.58	518.962433

Datum:	30.10.2020
Analyse:	150172c-00
Sigel:	GHC-AA-051-01
	COP: Dr. Clement Ghiazza
Method:	HR-MS
Ionis. :	ESIpos
solvent :	CH3OH
Spectrometer:	Exactive
Auswerter:	Marcus, Tel:2243

suggestion:
C19H14N2O4I1Cl1   MW: 496

Characteristic Ions:
519 = [496 + Na]

^{1}H NMR

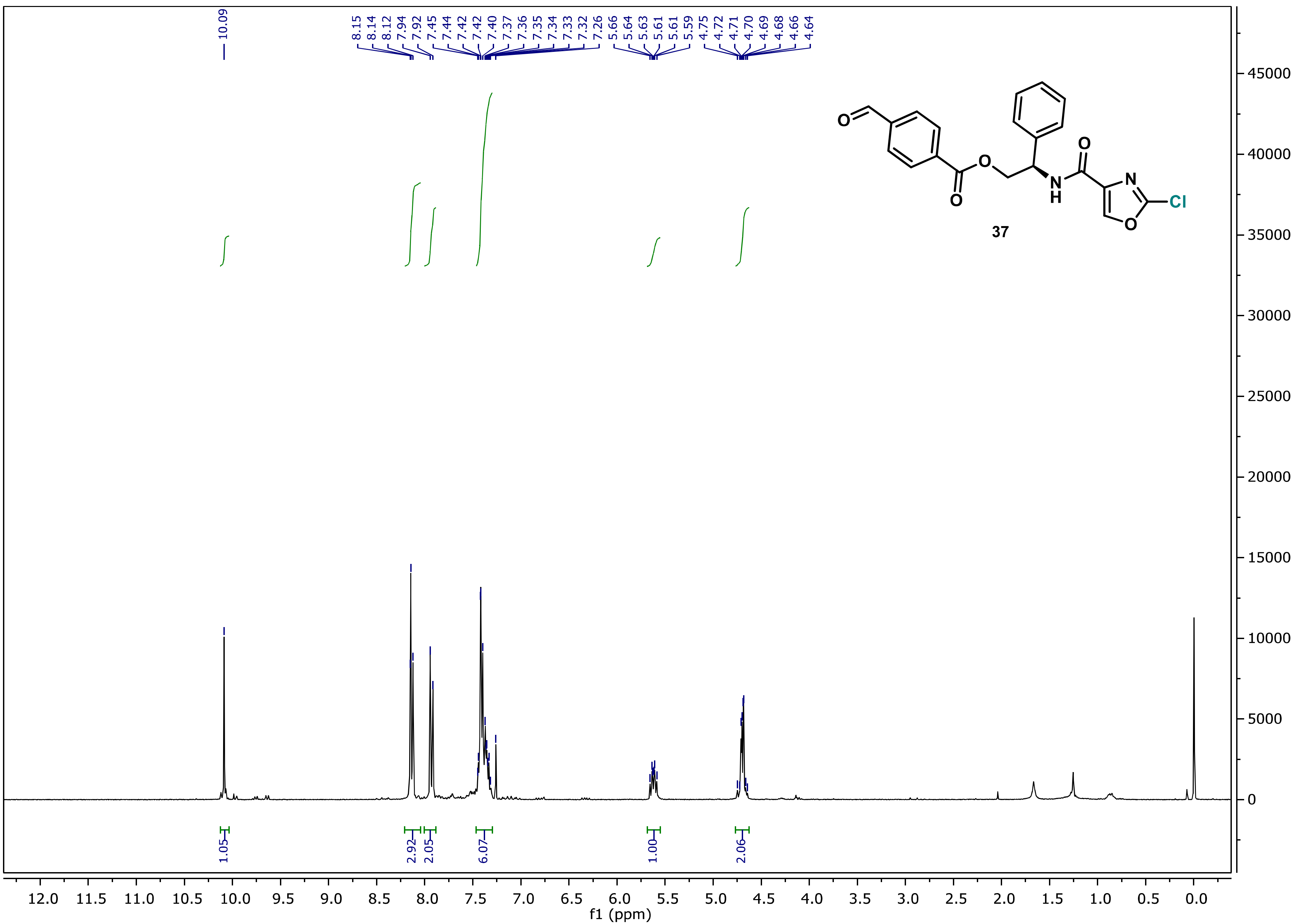
10.09
8.15 8.14 8.12 7.94 7.92 7.45 7.44 7.42 7.42 7.40 7.37 7.36 7.35 7.34 7.33 7.32 7.26
5.66 5.64 5.63 5.61 5.61 5.59
4.75 4.72 4.71 4.70 4.69 4.68 4.66 4.64
1.05 2.92 2.05 6.07 1.00 2.06
O N H O Cl N O O O
37
f1 (ppm)

^{13}C NMR

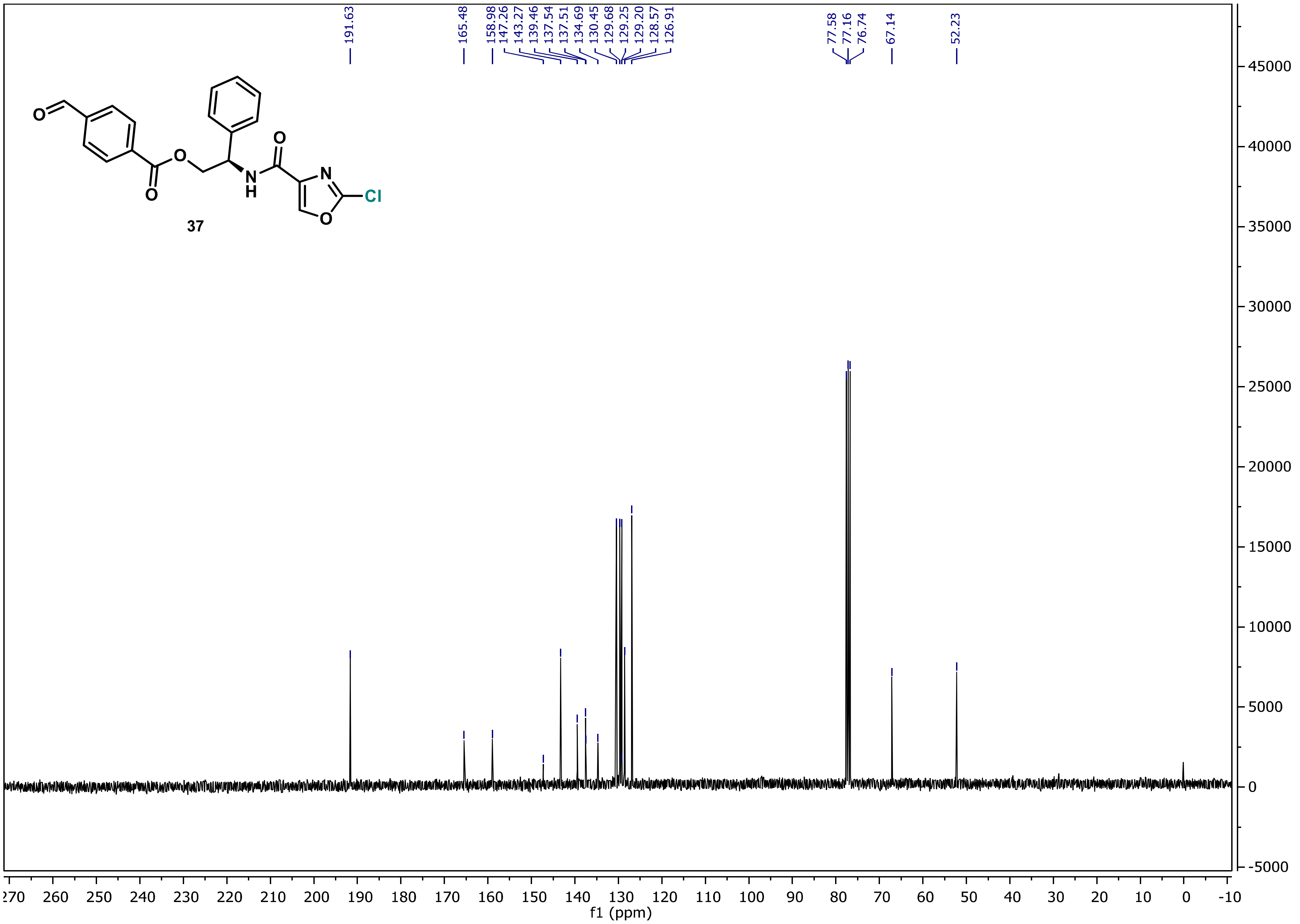

191.63
165.48
158.98
147.26
143.27
139.46
137.54
137.51
134.69
130.45
129.68
129.25
129.20
128.57
126.91
77.58
77.16
76.74
67.14
52.23
O
N
H
N
O
Cl
37
45000
40000
35000
30000
25000
20000
15000
10000
5000
0
-5000
270 260 250 240 230 220 210 200 190 180 170 160 150 140 130 120 110 100 90 80 70 60 50 40 30 20 10 0 -10
f1 (ppm)

Mass to be matched (m/z): 421.056580 Charge: 1

Mass Tolerance: ±0.005000

Restriction of atom numbers:

C	H	N	O	Cl	Na
1-100	1-100	1-5	1-10	1-1	1-1

Number of calculated Formulas: 6

Formula	Diff.(ppm)	theor. m/z
C20 H15 N2 O5 Cl1 Na1	-0.98	421.056169
C18 H13 N5 O4 Cl1 Na1	-4.17	421.054826
C23 H13 N3 O2 Cl1 Na1	5.39	421.058848
C17 H17 N1 O8 Cl1 Na1	-7.34	421.053491
C11 H17 N5 O9 Cl1 Na1	9.79	421.060700
C15 H15 N4 O7 Cl1 Na1	-10.53	421.052147

Datum:	15.10.2020
Analyse:	149877c-00
Sigel:	GHC-AA-065-01
	COP: Dr. Clement Ghiazza
Method:	HR-MS
Ionis. :	ESIpos
solvent :	CH3OH
Spectrometer:	Exactive
Auswerter:	Marcus, Tel:2243

suggestion:
C20H15N2O5Cl1   MW: 398

Characteristic Ions:
421 = [398 + Na]

^{1}H NMR

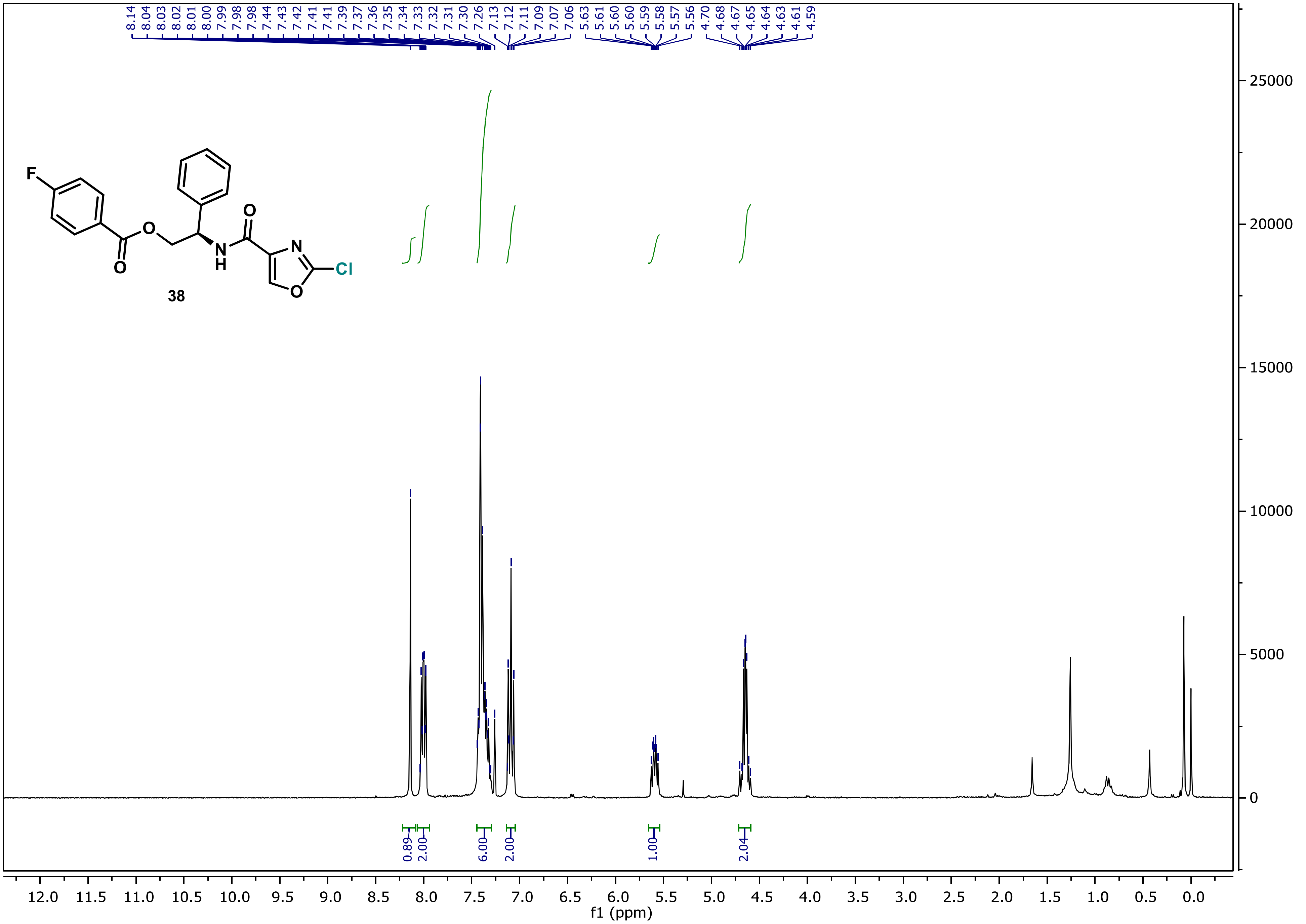

8.14
8.04
8.03
8.02
8.01
8.00
7.99
7.98
7.98
7.44
7.43
7.42
7.41
7.41
7.39
7.37
7.36
7.35
7.34
7.33
7.32
7.31
7.30
7.26
7.13
7.12
7.11
7.09
7.07
7.06
5.63
5.61
5.60
5.60
5.59
5.58
5.57
5.56
4.70
4.68
4.67
4.65
4.64
4.63
4.61
4.59
F
O
O
N
H
O
N
O
Cl
38
0.89
2.00
6.00
2.00
1.00
2.04
25000
20000
15000
10000
5000
0
12.0
11.5
11.0
10.5
10.0
9.5
9.0
8.5
8.0
7.5
7.0
6.5
6.0
5.5
5.0
4.5
4.0
3.5
3.0
2.5
2.0
1.5
1.0
0.5
0.0
f1 (ppm)

^{13}C NMR

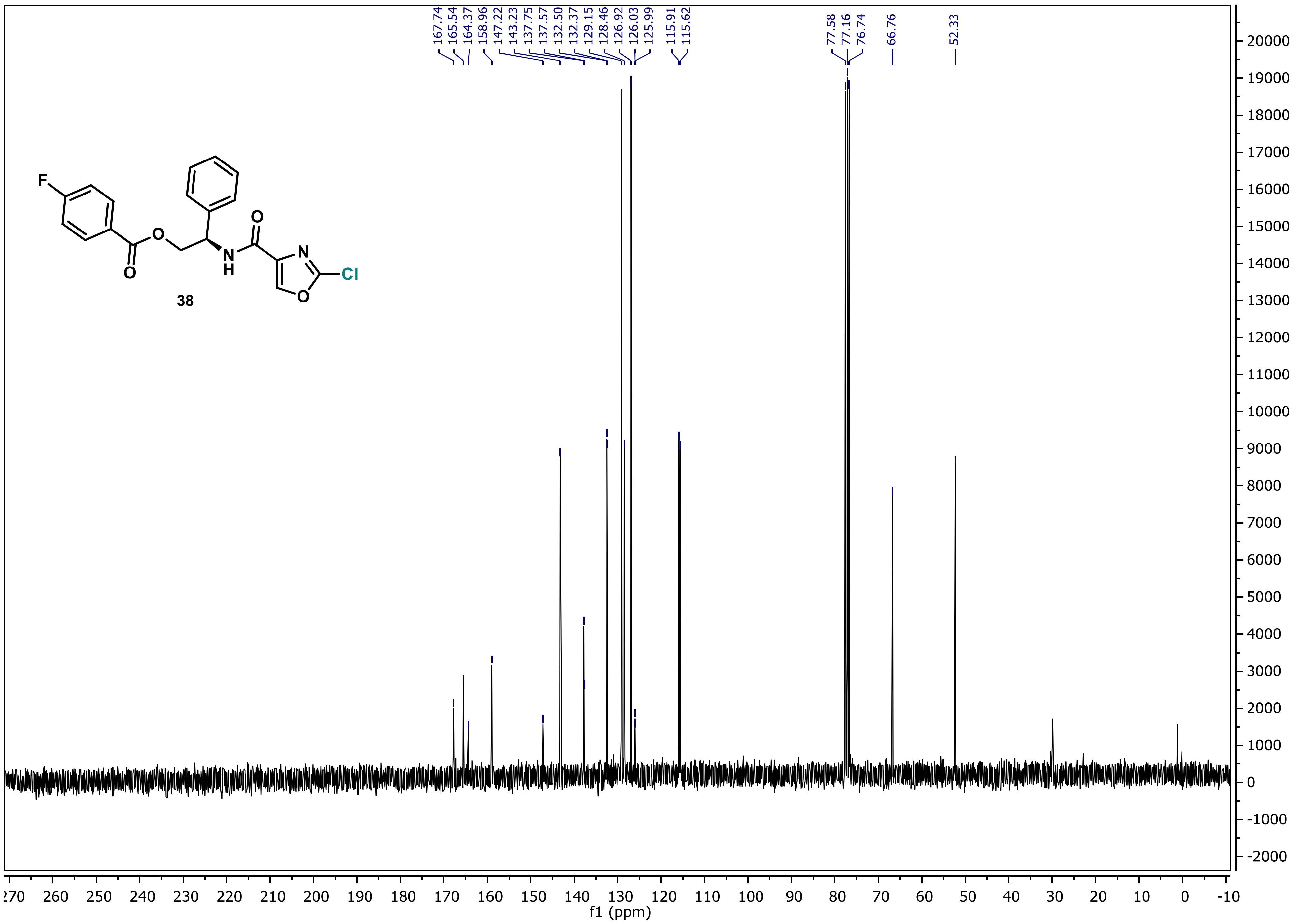

167.74
165.54
164.37
158.96
147.22
143.23
137.75
137.57
132.50
132.37
129.15
128.46
126.92
126.03
125.99
115.91
115.62
77.58
77.16
76.74
66.76
52.33
38
f1 (ppm)

^{19}F NMR

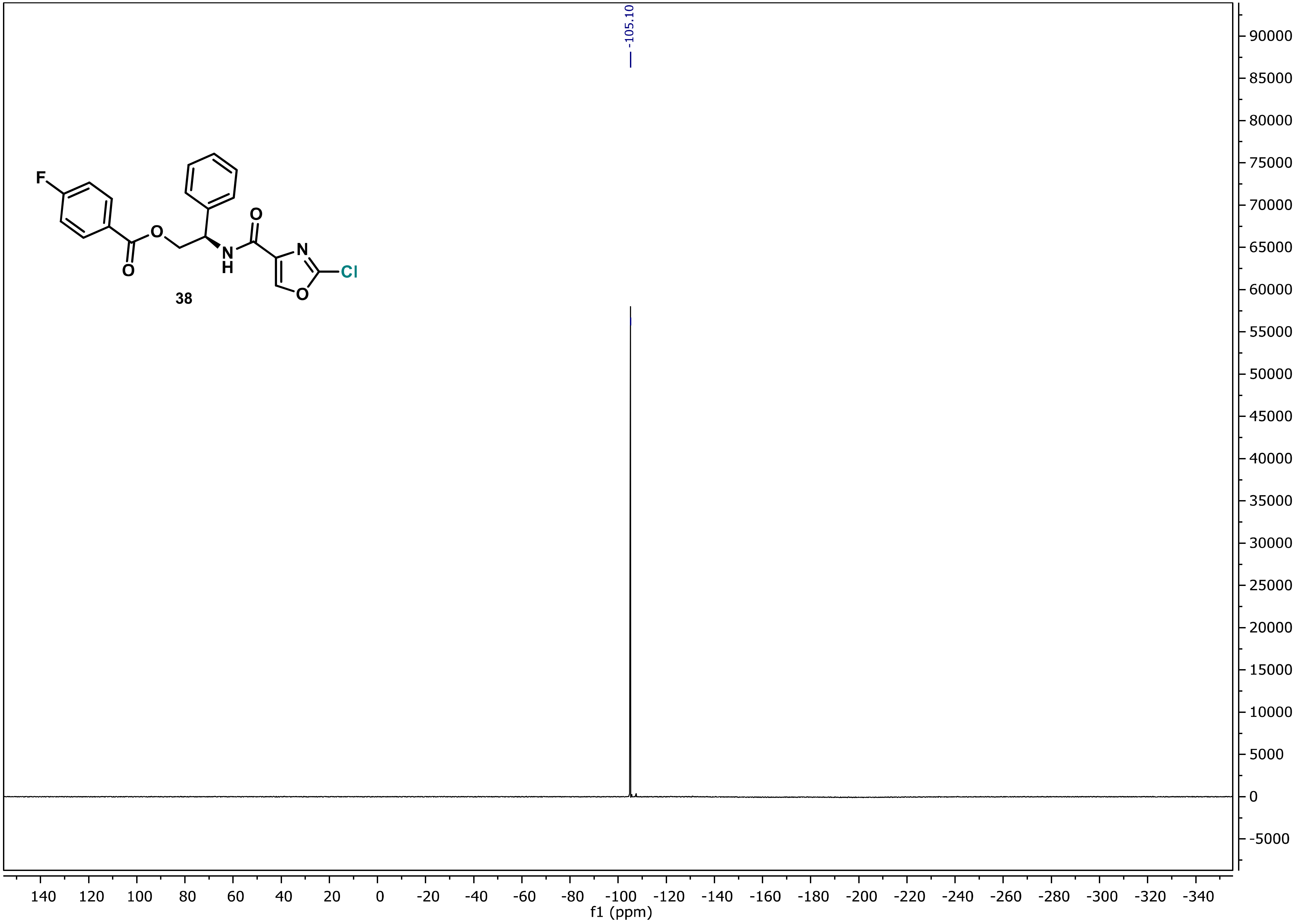

Mass to be matched (m/z): 411.052650 Charge: 1

Mass Tolerance: ±0.005000
Restriction of atom numbers:

C	H	N	O	Cl	F	Na
1-100	1-100	1-5	1-10	1-1	1-1	1-1

Number of calculated Formulas: 6

Formula	Diff.(ppm)	theor. m/z
C19 H14 N2 O4 Cl1 F1 Na1	-1.99	411.051833
C22 H12 N3 O1 Cl1 F1 Na1	4.53	411.054512
C17 H12 N5 O3 Cl1 F1 Na1	-5.26	411.050489
C16 H16 N1 O7 Cl1 F1 Na1	-8.50	411.049154
C10 H16 N5 O8 Cl1 F1 Na1	9.04	411.056364
C14 H14 N4 O6 Cl1 F1 Na1	-11.77	411.047810

Datum:	15.10.2020
Analyse:	149871c-00
Sigel:	GHC-AA-048-01
	COP: Dr. Clement Ghiazza
Method:	HR-MS
Ionis. :	ESIpos
solvent :	CH3OH
Spectrometer:	Exactive
Auswerter:	Marcus, Tel:2243

suggestion:
c19H14N2O4Cl1F1   MW: 388

Characteristic Ions:
411 = [388 + Na]

^1H NMR

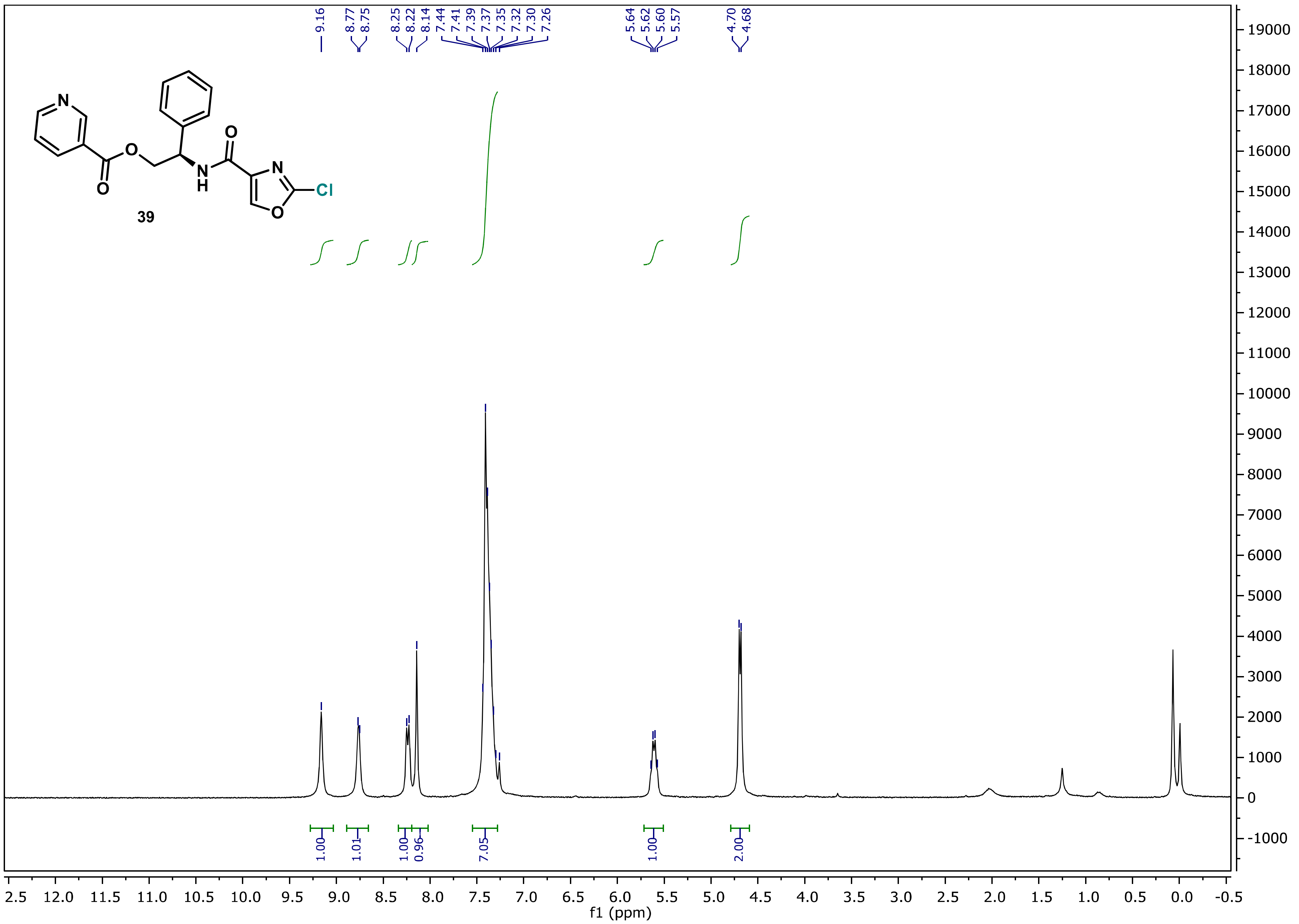

9.16
8.77
8.75
8.25
8.22
8.14
7.44
7.41
7.39
7.37
7.35
7.32
7.30
7.26
5.64
5.62
5.60
5.57
4.70
4.68
39
Cl
1.00
1.01
1.00
0.96
7.05
1.00
2.00
f1 (ppm)

^{13}C NMR

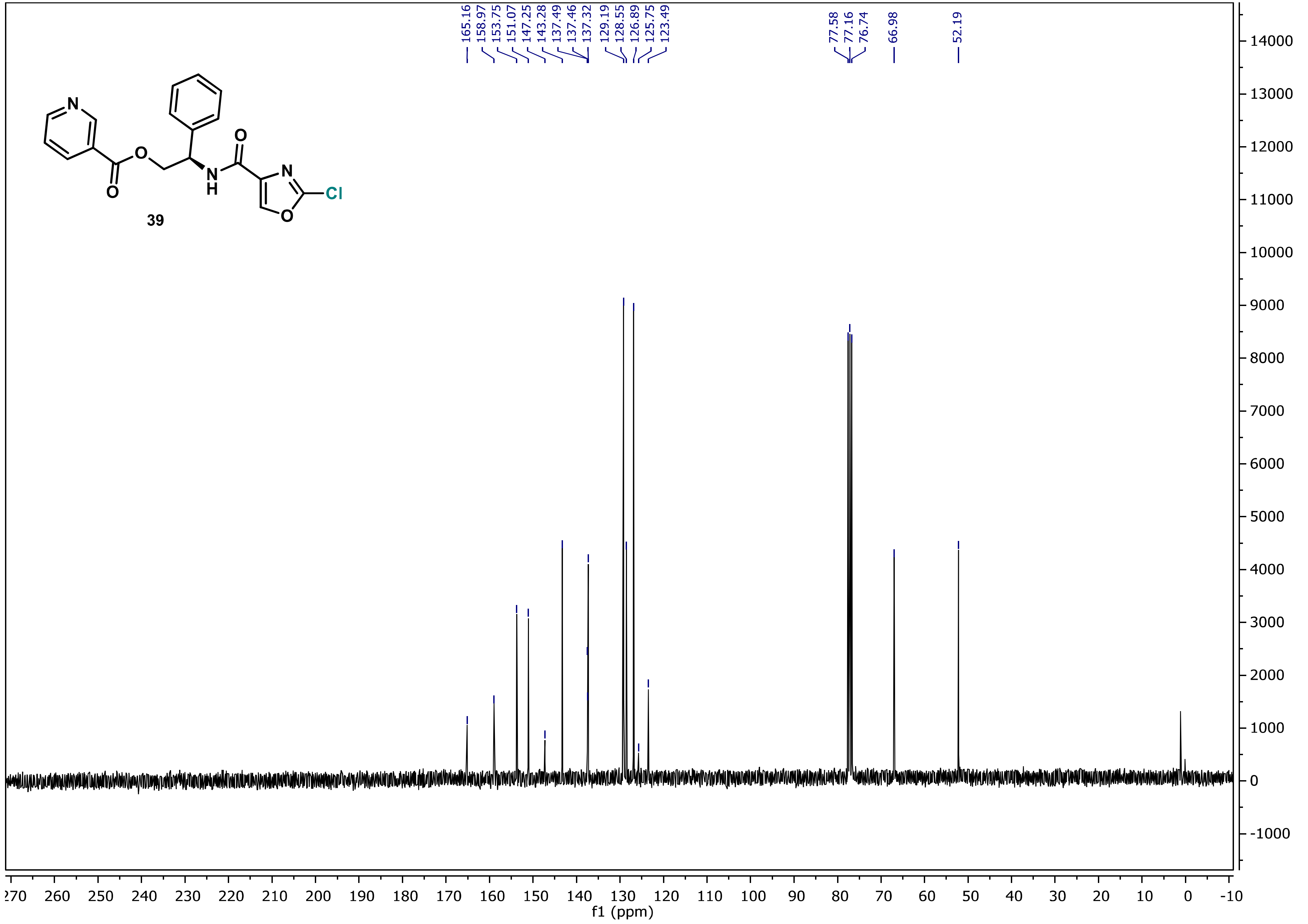

39
165.16
158.97
153.75
151.07
147.25
143.28
137.49
137.46
137.32
129.19
128.55
126.89
125.75
123.49
77.58
77.16
76.74
66.98
52.19
f1 (ppm)

Mass to be matched (m/z): 394.056660 Charge: 1

Mass Tolerance: ±0.005000
Restriction of atom numbers:

C	H	N	O	Cl	Na
1-100	1-100	1-5	1-10	1-1	1-1

Number of calculated Formulas: 5

Formula	Diff.(ppm)	theor. m/z
C18 H14 N3 O4 Cl1 Na1	-0.40	394.056503
C21 H12 N4 O1 Cl1 Na1	6.40	394.059182
C15 H16 N2 O7 Cl1 Na1	-7.20	394.053824
C23 H14 N1 O2 Cl1 Na1	9.81	394.060526
C13 H14 N5 O6 Cl1 Na1	-10.61	394.052481

Datum:	15.10.2020
Analyse:	149898c-00
Sigel:	GHC-AA-066-01<br>COP: Dr. Clement Ghiazza
Method:	HR-MS
Ionis. :	ESIpos
solvent :	CH3OH
Spectrometer:	Exactive
Auswerter:	Marcus, Tel:2243

suggestion:
C18H14N3O4Cl1   MW: 371

Characteristic Ions:
394 = [371 + Na]

^1H NMR

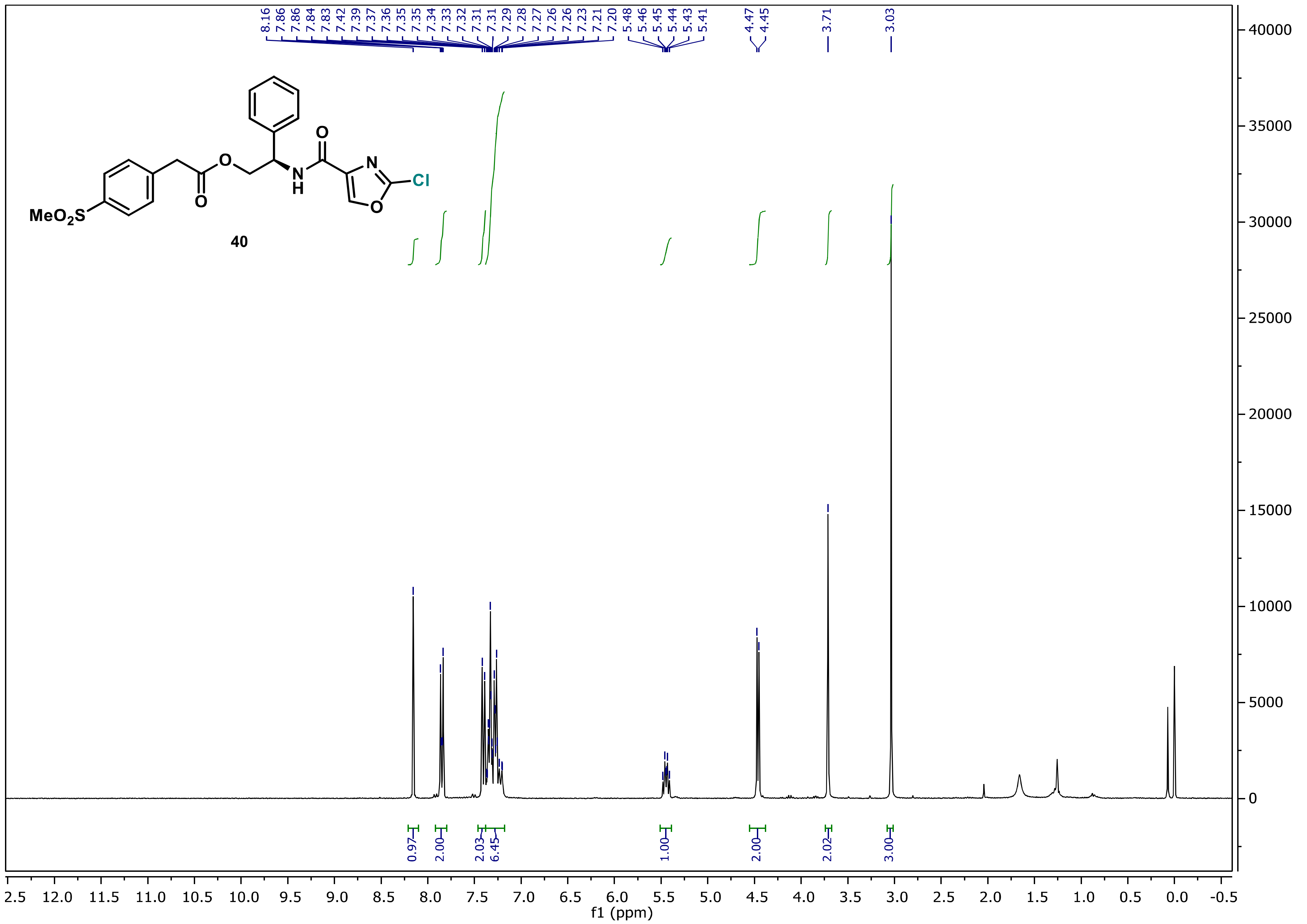

MeO2S
O
O
N
H
O
N
O
Cl
40
8.16
7.86
7.86
7.84
7.83
7.42
7.39
7.37
7.36
7.35
7.35
7.34
7.33
7.32
7.31
7.31
7.29
7.28
7.27
7.26
7.26
7.23
7.21
7.20
5.48
5.46
5.45
5.44
5.43
5.41
4.47
4.45
3.71
3.03
0.97
2.00
2.03
6.45
1.00
2.00
2.02
3.00
40000
35000
30000
25000
20000
15000
10000
5000
0
2.5 12.0 11.5 11.0 10.5 10.0 9.5 9.0 8.5 8.0 7.5 7.0 6.5 6.0 5.5 5.0 4.5 4.0 3.5 3.0 2.5 2.0 1.5 1.0 0.5 0.0 -0.5
f1 (ppm)

^{13}C NMR

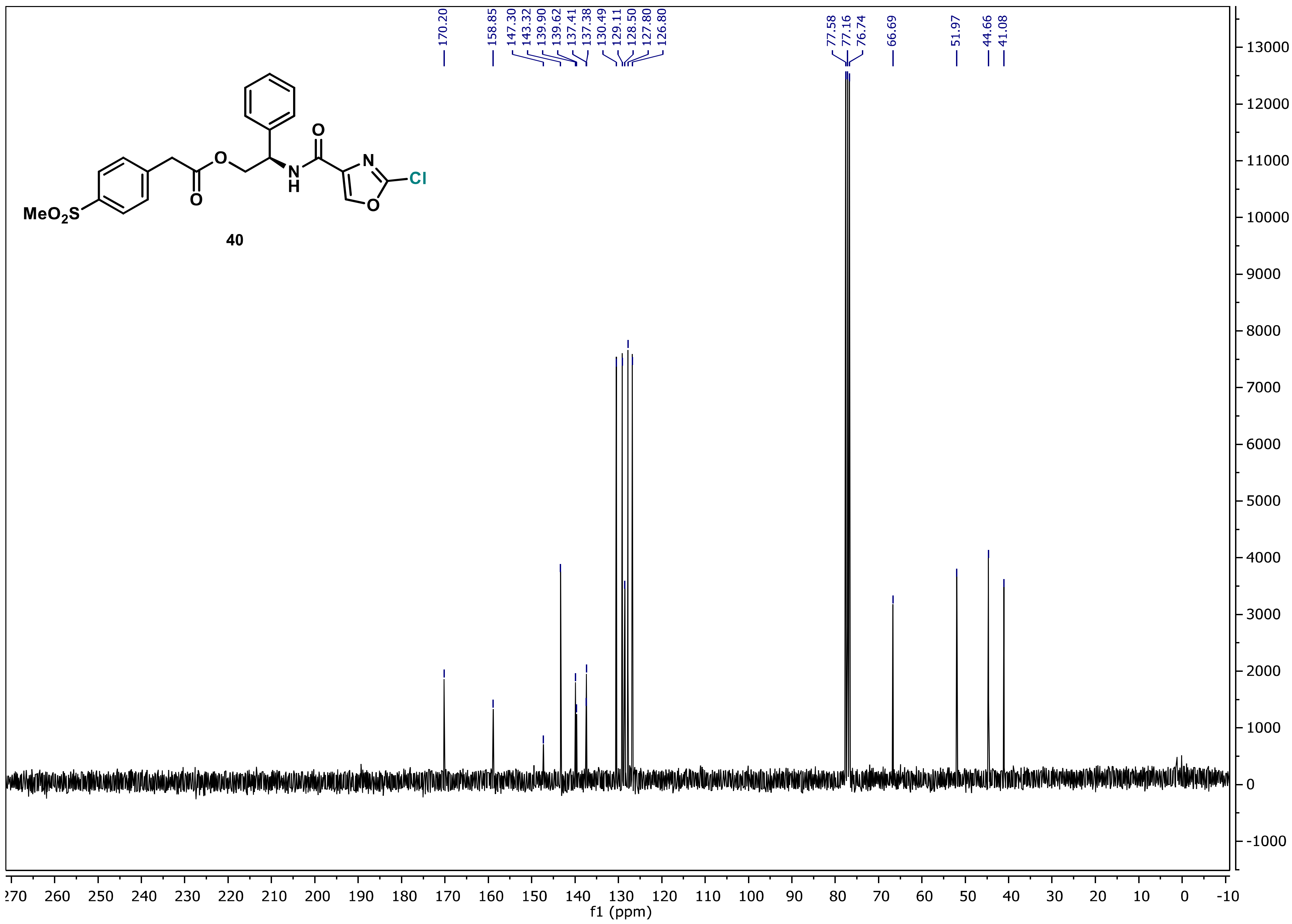

170.20
158.85
147.30
143.32
139.90
139.62
137.41
137.38
130.49
129.11
128.50
127.80
126.80
77.58
77.16
76.74
66.69
51.97
44.66
41.08
MeO2S
O
O
N
H
O
N
O
Cl
13000
12000
11000
10000
9000
8000
7000
6000
5000
4000
3000
2000
1000
0
-1000
270 260 250 240 230 220 210 200 190 180 170 160 150 140 130 120 110 100 90 80 70 60 50 40 30 20 10 0 -10
f1 (ppm)

40

*** Angegebene Mol.-Gewichte u. Massenzahlen basieren auf dem häufigsten Isotop der Elemente ***

MassLib

Mass to be matched (m/z): 485.055210 Charge: 1

Mass Tolerance: ±0.005000
Restriction of atom numbers:

C	H	N	O	S	Cl	Na
1-100	1-100	1-5	1-10	1-1	1-1	1-1

Number of calculated Formulas: 6

Formula	Diff.(ppm)	theor. m/z
C21 H19 N2 O6 S1 Cl1 Na1	-1.55	485.054457
C24 H17 N3 O3 S1 Cl1 Na1	3.97	485.057135
C19 H17 N5 O5 S1 Cl1 Na1	-4.32	485.053113
C18 H21 N1 O9 S1 Cl1 Na1	-7.08	485.051778
C12 H21 N5 O10 S1 Cl1 Na1	7.79	485.058988
C16 H19 N4 O8 S1 Cl1 Na1	-9.85	485.050434

Datum: 15.10.2020
Analyse: 149873c-00

Sigel: GHC-AA-057-01
COP: Dr. Clement Ghiazza

Method: HR-MS
Ionis. : ESIpos
solvent : CH3OH
Spectrometer: Exactive

Auswerter: Marcus, Tel:2243

suggestion:
C21H19N2O6S1Cl1   MW: 462

Characteristic Ions:
485 = [462 + Na]

^{1}H NMR – in situ

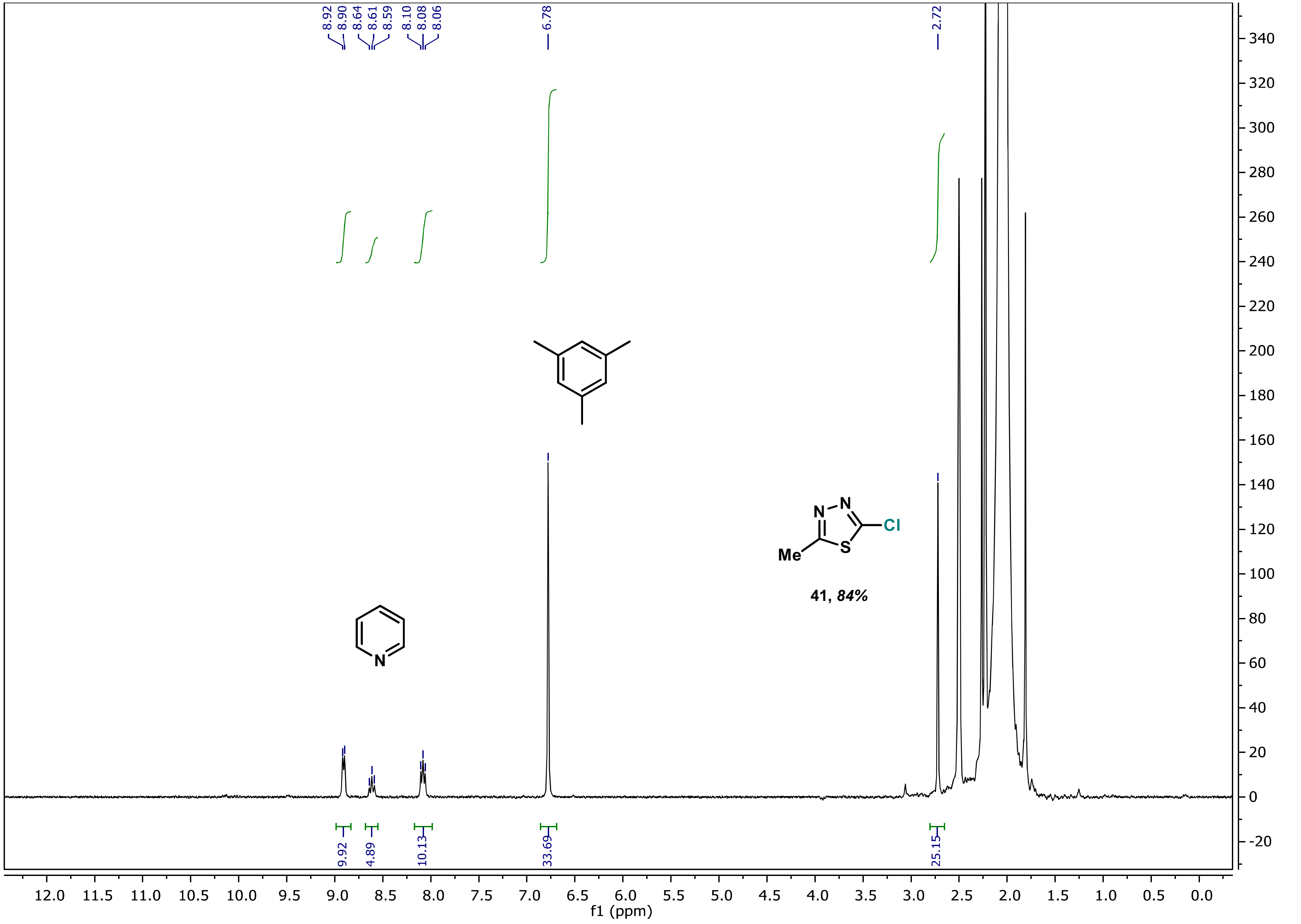

*** Angegebene Mol.-Gewichte u. Massenzahlen basieren auf dem häufigsten Isotop der Elemente ***

MassLib

Mass to be matched (m/z): 133.970270 Charge: 1

Mass Tolerance: ±0.050000
Restriction of atom numbers:

C	H	N	Cl	S
1-100	1-100	1-2	1-2	1-1

Number of calculated Formulas: 3

Formula	Diff.(ppm)	theor. m/z
C3 H3 N2 Cl1 S1	-2.03	133.969998
C1 H6 N1 Cl2 S1	-82.24	133.959252
C4 H5 N1 Cl1 S1	91.84	133.982574

Suggestion:
C3H3N2Cl1S1 MW: 134

8.07.2020
File: 147977c-00.raw
Analyse: GHC-GA-191-01
COP: Dr. Clement Ghiazza

Messung:	GC-MS
Ionisierung:	GC-EI
Spektrometer:	Q Exactive GC Orbitrap
Säule:	MS 75 ZB-5HT 30+5
Länge:	30+5
Temp.:	35-10-285-5
GC-Nr.:	-
ELNA-Nr.:	25986

Auswerter: Haupt (2243)

^{1}H NMR – in situ

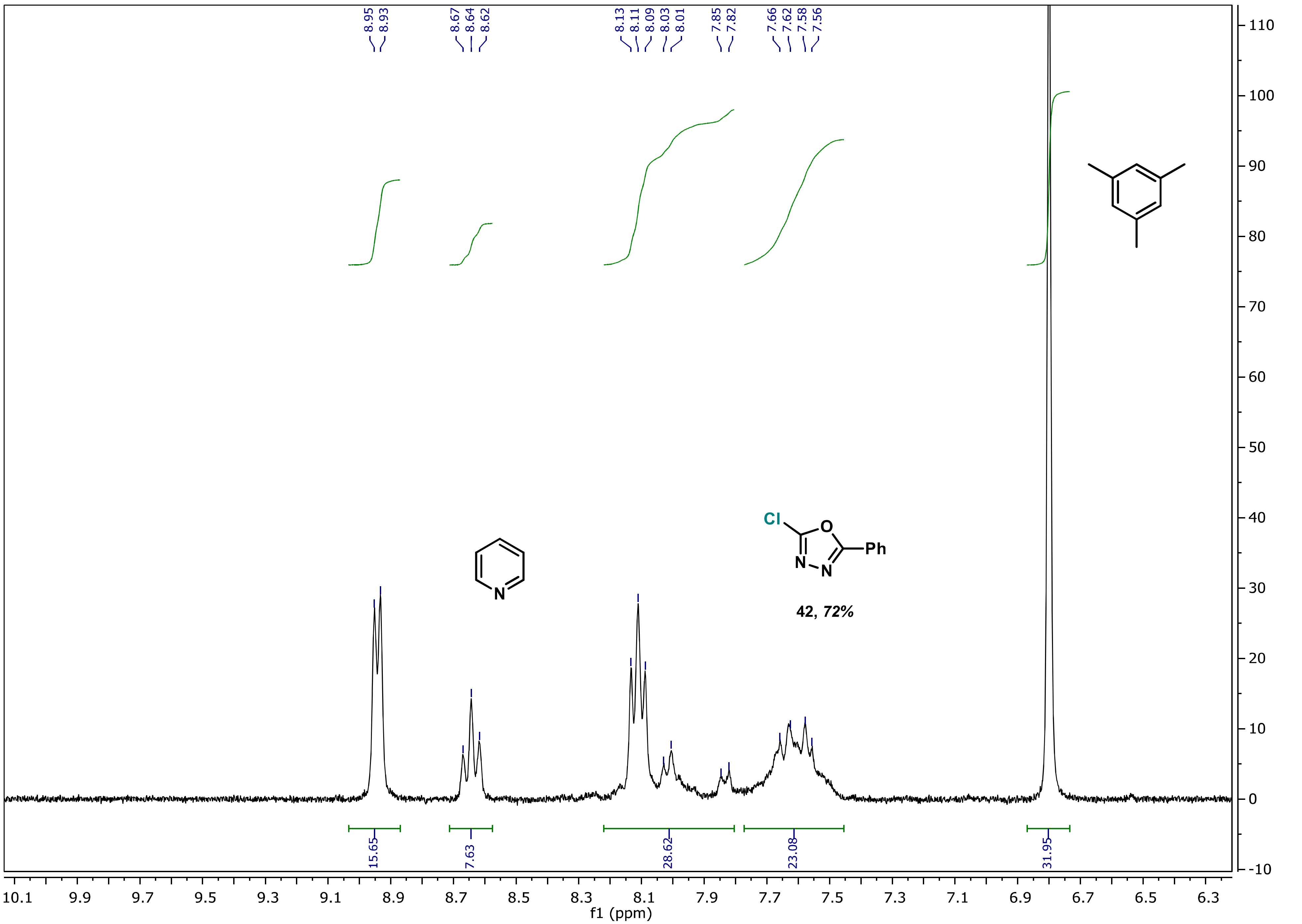

42, *72%*

No.	MW.	Comment
1	180	Your proposed structure is possible<br>Heteroatoms: Cl1<br>Ref.-Spektr. Nr. OU1563:
2	222	Compare NJ232760:1,3,4-Oxadiazole, 2,5-diphenyl-<br>Overlapping with MW:242,<br>Unknown structure, Heteroatoms: Cl1

6.10.2020
File: 149714a-00.raw
Analyse: GHC-GA-366-01
COP: Dr. Clement Ghiazza

Messung:	GC-MS
Ionisierung:	GC-EI
Spektrometer:	QExactiveGC
Säule:	MS 50 TX1+VS
Länge:	30+7
Temp.:	35-10-285-5
GC-Nr.:	-

Auswerter: Margold

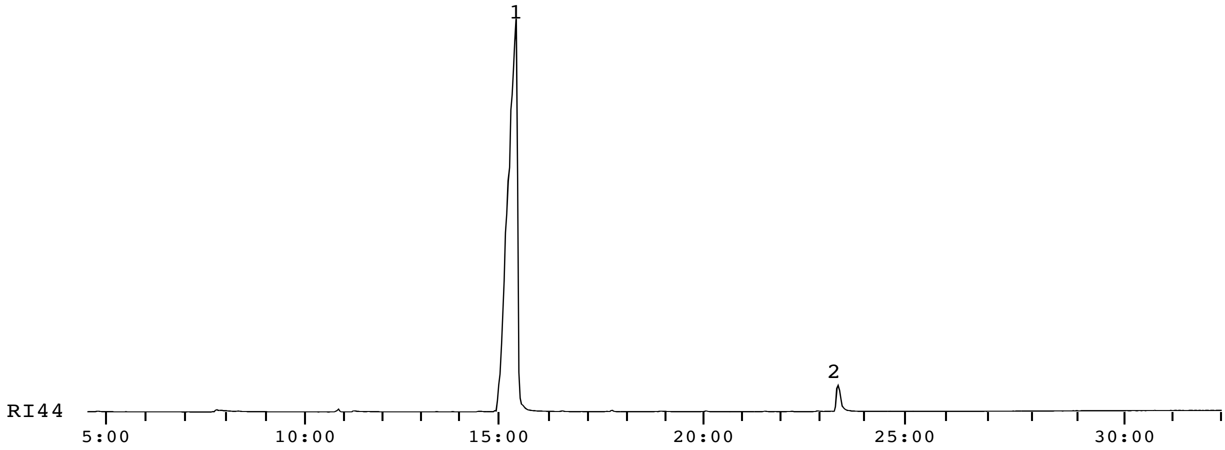

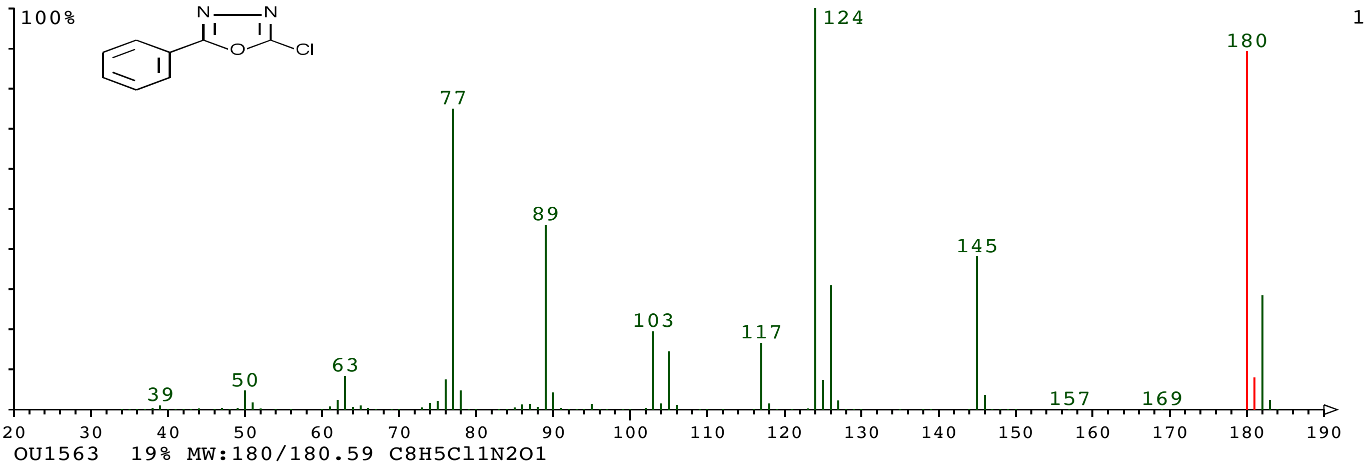

OU1563 19% MW:180/180.59 C8H5Cl1N2O1

34	0.06	61	0.68	87	1.32	116	0.08	149	0.03
35	0.06	62	2.30	88	0.58	117	16.56	150	0.03
36	0.03	63	8.35	89	46.00	118	1.41	157	0.03
37	0.15	64	0.63	90	4.18	119	0.06	169	0.03
38	0.29	65	1.02	91	0.26	121	0.04	180	89.25
39	1.04	66	0.28	92	0.03	123	0.17	181	7.94
40	0.10	67	0.03	93	0.03	124	100.00	182	28.41
41	0.06	69	0.03	94	0.03	125	7.30	183	2.29
42	0.03	70	0.10	95	1.30	126	30.94	184	0.04
43	0.03	73	0.43	96	0.06	127	2.22		
44	0.20	74	1.57	97	0.06	128	0.08		
46	0.06	75	2.01	99	0.12	129	0.03		
47	0.38	76	7.41	100	0.12	130	0.15		
48	0.06	77	74.93	102	0.37	135	0.03		
49	0.34	78	4.74	103	19.45	138	0.06		
50	4.68	79	0.07	104	1.48	139	0.04		
51	1.75	80	0.06	105	14.46	144	0.06		
52	0.18	83	0.03	106	1.07	145	38.12		
54	0.03	84	0.05	109	0.03	146	3.53		
56	0.03	85	0.46	110	0.06	147	0.08		
60	0.07	86	1.13	112	0.03	148	0.03		

OU1563 19% MW:180/180.59 C8H5Cl1N2O1

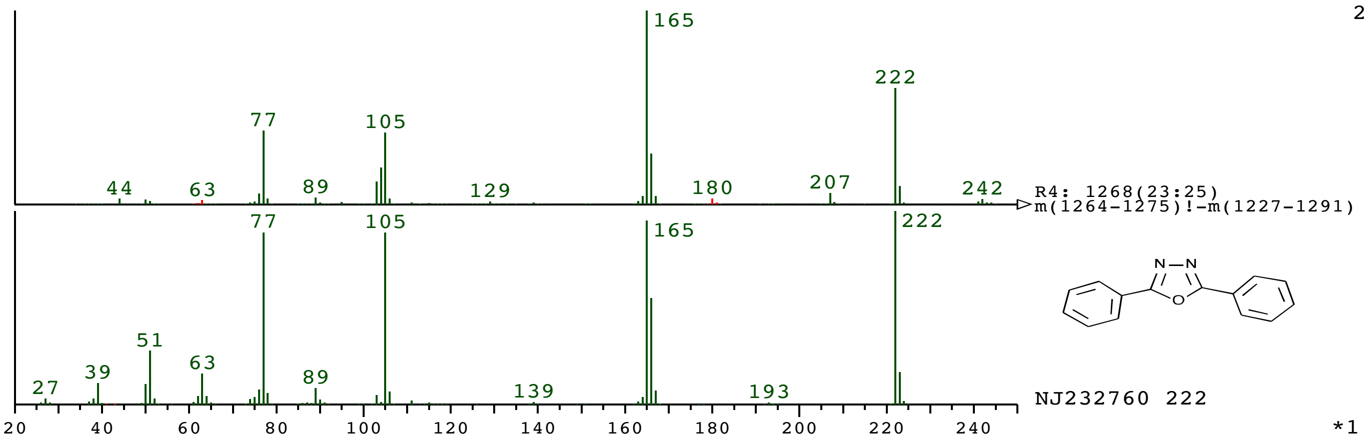

33	0.01	57	0.03	87	0.19	117	0.09	152	0.15	191	0.06	222	59.98
34	0.03	58	0.01	88	0.14	118	0.08	153	0.01	192	0.01	223	9.30
35	0.02	59	0.02	89	3.38	119	0.13	157	0.01	193	0.34	224	0.61
36	0.04	60	0.01	90	0.75	121	0.01	162	0.09	194	0.10	232	0.01
37	0.07	61	0.12	91	0.31	124	0.01	163	1.41	196	0.00	237	0.00
38	0.12	62	0.43	92	0.04	125	0.10	164	4.03	200	0.00	238	0.00
39	0.34	63	1.87	95	0.88	126	0.08	165	100.00	201	0.00	240	0.01
40	0.04	64	0.30	99	0.11	127	0.08	166	25.92	202	0.02	241	1.34
44	2.75	65	0.05	100	0.07	128	0.07	167	4.09	204	0.00	242	2.67
45	0.03	66	0.10	101	0.02	129	1.15	168	0.25	205	0.36	243	0.79
46	0.03	72	0.00	102	0.26	130	0.27	170	0.00	206	0.12	244	0.86
47	0.01	73	0.08	103	11.72	131	0.09	172	0.00	207	5.67	245	0.12
48	0.01	74	0.78	104	18.75	133	0.07	174	0.00	208	0.93	249	0.00
49	0.06	75	1.04	105	36.93	135	0.27	175	0.01	209	0.20	250	0.01
50	2.34	76	5.36	106	2.76	137	0.02	176	0.11	213	0.00		
51	1.38	77	37.99	107	0.16	138	0.06	177	0.13	214	0.01		
52	0.14	78	2.82	111	0.72	139	0.80	178	0.35	215	0.09		
53	0.05	79	0.07	113	0.10	140	0.09	179	0.14	216	0.00		
54	0.02	82	0.00	114	0.02	141	0.01	180	2.82	217	0.01		
55	0.01	85	0.05	115	0.50	143	0.01	181	0.63	218	0.00		
56	0.01	86	0.22	116	0.05	151	0.15	182	0.04	221	0.10		

R4: 1268(23:25) m(1264-1275) 149714a-00! -m(1227-1291) 14361 27% 149714a-00 * GHC-GA-366-01

Mass to be matched (m/z): 180.008840 Charge: 1

Mass Tolerance: ±0.005000
Restriction of atom numbers:

C	H	N	O	Cl
1-110	1-100	1-2	1-5	1-1

Number of calculated Formulas: 2

Formula	Diff.(ppm)	theor. m/z
C8 H5 N2 O1 Cl1	-1.94	180.008490
C5 H7 N1 O4 Cl1	-16.82	180.005812

6.10.2020
File: 149714a-00.raw
Analyse: GHC-GA-366-01
COP: Dr. Clement Ghiazza

Messung:	GC-MS
Ionisierung:	GC-EI
Spektrometer:	QExactiveGC
Säule:	MS 50 TX1+VS
Länge:	30+7
Temp.:	35-10-285-5
GC-Nr.:	-

Auswerter: Margold

^{1}H NMR

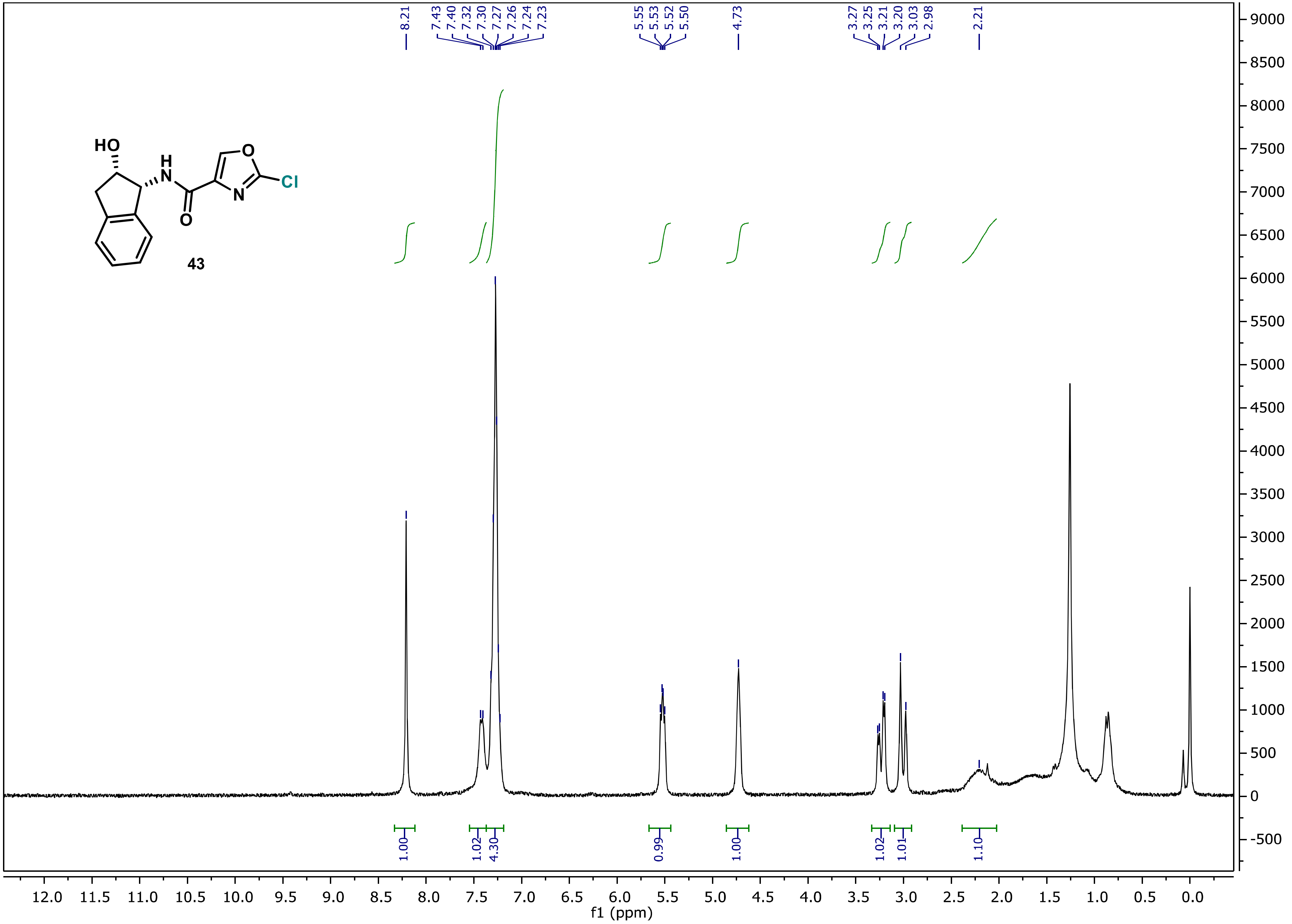

43
8.21
7.43
7.40
7.32
7.30
7.27
7.26
7.24
7.23
5.55
5.53
5.52
5.50
4.73
3.27
3.25
3.21
3.20
3.03
2.98
2.21
1.00
1.02
4.30
0.99
1.00
1.02
1.01
1.10
f1 (ppm)

^{13}C NMR

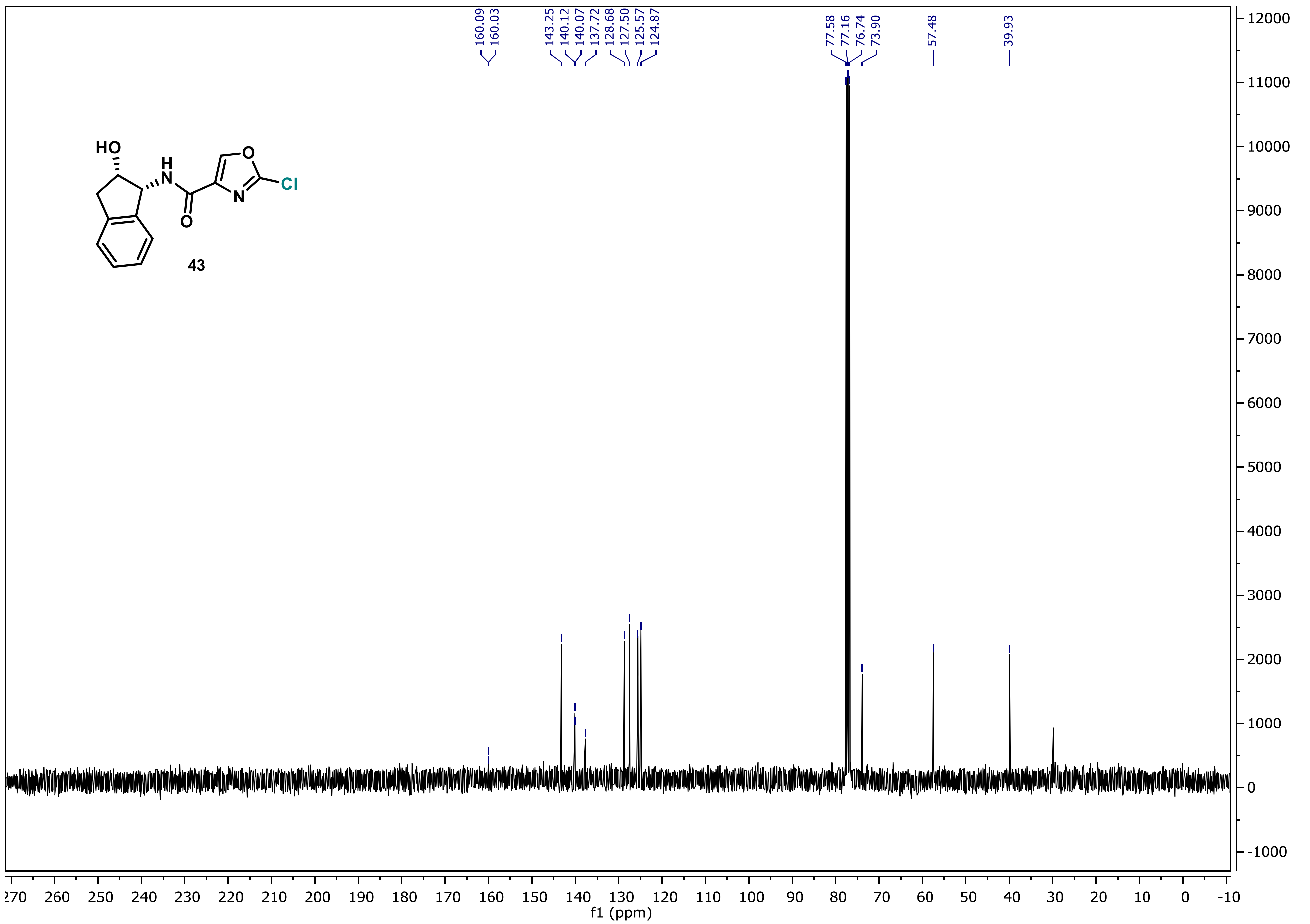
160.09
160.03
143.25
140.12
140.07
137.72
128.68
127.50
125.57
124.87
77.58
77.16
76.74
73.90
57.48
39.93
HO
H
N
O
N
Cl
O
43
f1 (ppm)

Mass to be matched (m/z): 301.034780 Charge: 1

Mass Tolerance: ±0.005000
Restriction of atom numbers:

C	H	Cl	N	O	Na
1-100	1-100	1-1	max 2	max 10	1-1

Number of calculated Formulas: 3

Formula	Diff.(ppm)	theor. m/z
C13 H11 Cl1 N2 O3 Na1	0.86	301.035039
C10 H13 Cl1 N1 O6 Na1	-8.04	301.032361
C18 H11 Cl1 O1 Na1	14.22	301.039062

Datum	19.10.2020
Analyse:	149943c-00
Sigel:	GHC-AA-069-01
COP: Dr. Clement Ghiazza	
Messung:	HRMS
Methode:	ESIpos
Lösungsmittel:	CH3OH
Spektrometer:	Exactive
Auswerter:	Kampen (2242)

Suggestion:
C13H11Cl1N2O3 MW 278

characteristical ion
301 = [278 + Na]+

^{1}H NMR

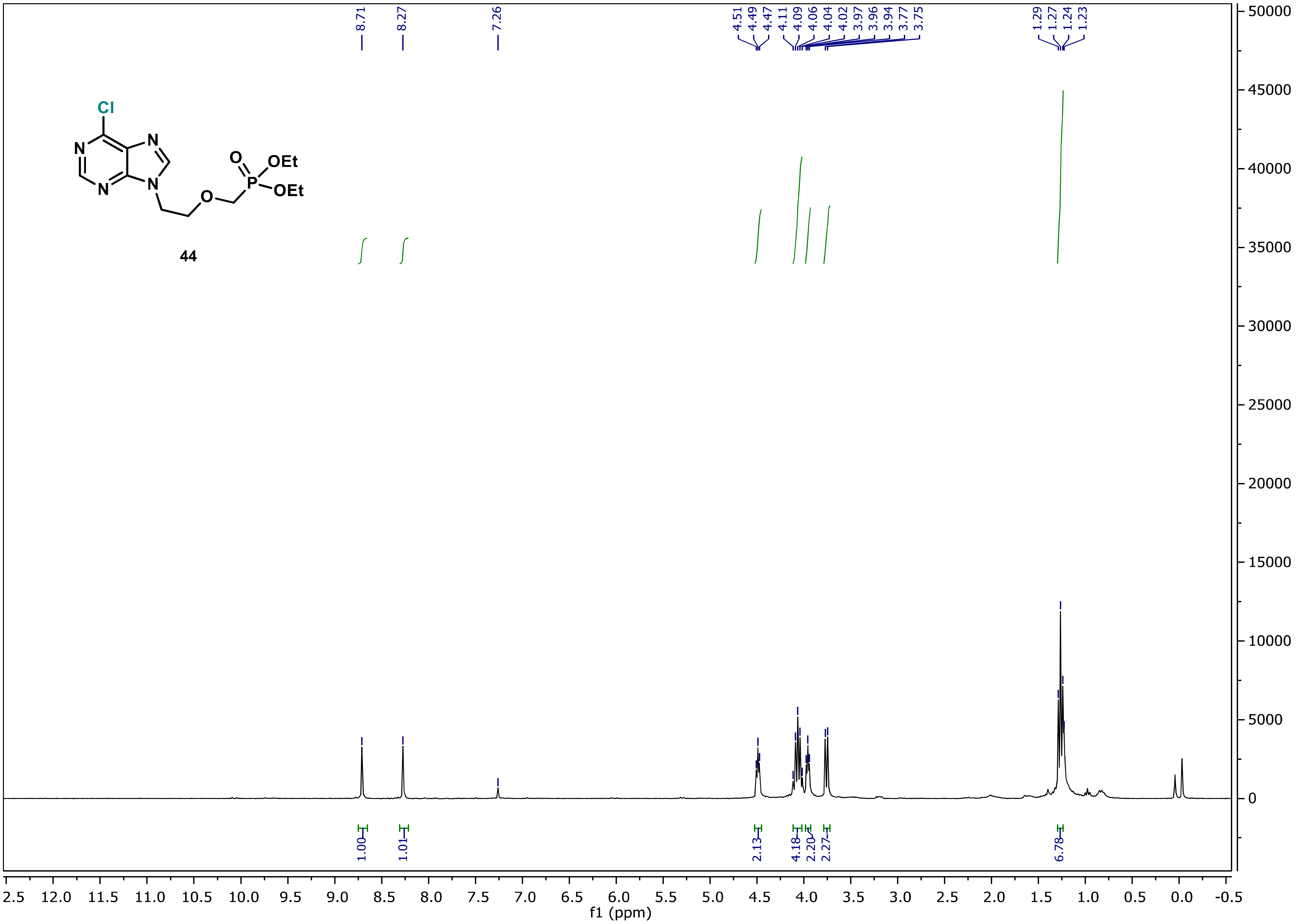
Cl
N
N
N
N
O
O
P
OEt
OEt
44
8.71
8.27
7.26
4.51
4.49
4.47
4.11
4.09
4.06
4.04
4.02
3.97
3.96
3.94
3.77
3.75
1.29
1.27
1.24
1.23
1.00
1.01
2.13
4.18
2.20
2.27
6.78
50000
45000
40000
35000
30000
25000
20000
15000
10000
5000
0
2.5
12.0
11.5
11.0
10.5
10.0
9.5
9.0
8.5
8.0
7.5
7.0
6.5
6.0
5.5
5.0
4.5
4.0
3.5
3.0
2.5
2.0
1.5
1.0
0.5
0.0
-0.5
f1 (ppm)

^{13}C NMR

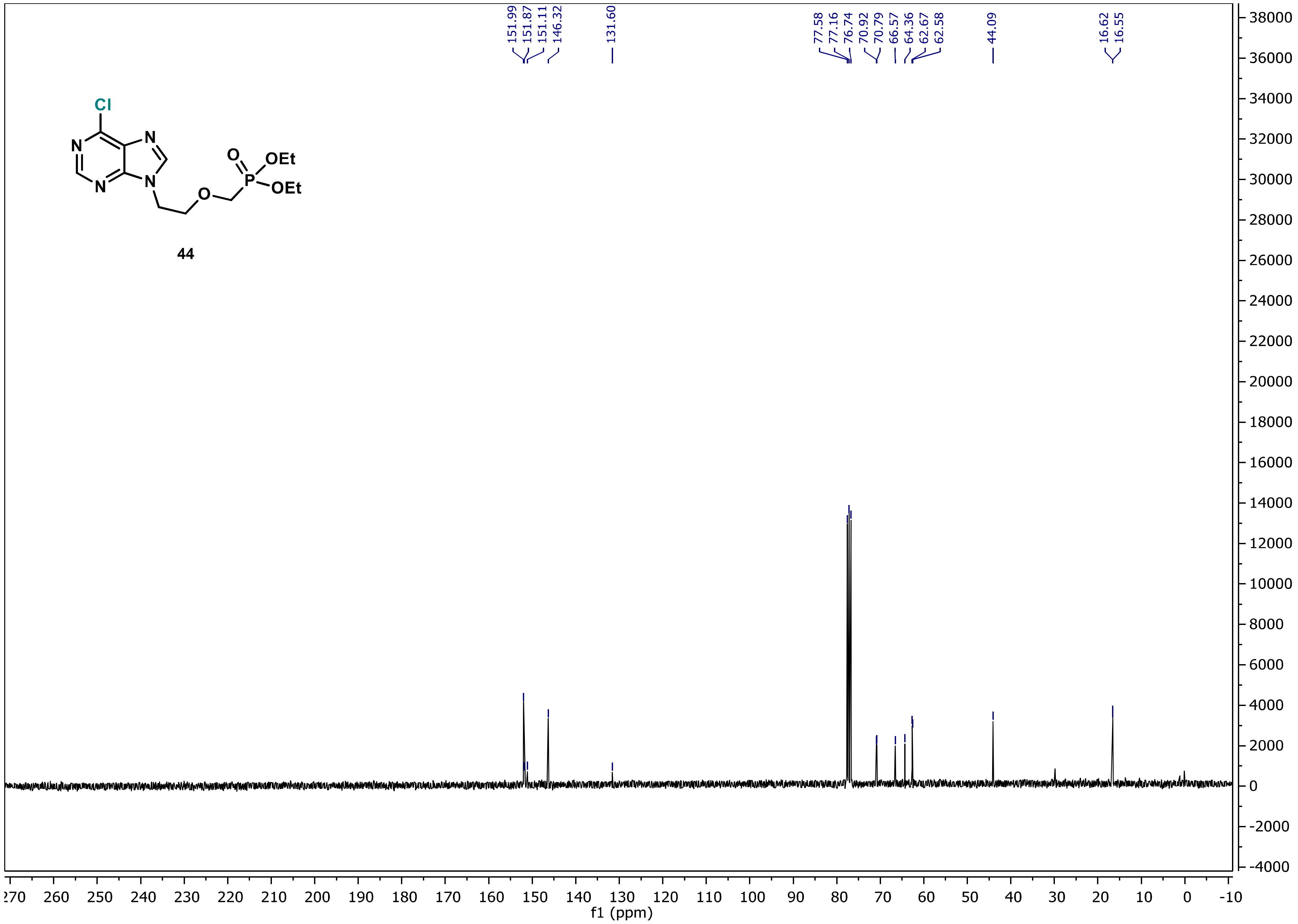

Cl
N
N
N
N
O
O
P
OEt
OEt
151.99
151.87
151.11
146.32
131.60
77.58
77.16
76.74
70.92
70.79
66.57
64.36
62.67
62.58
44.09
16.62
16.55
f1 (ppm)

44

^{31}P NMR

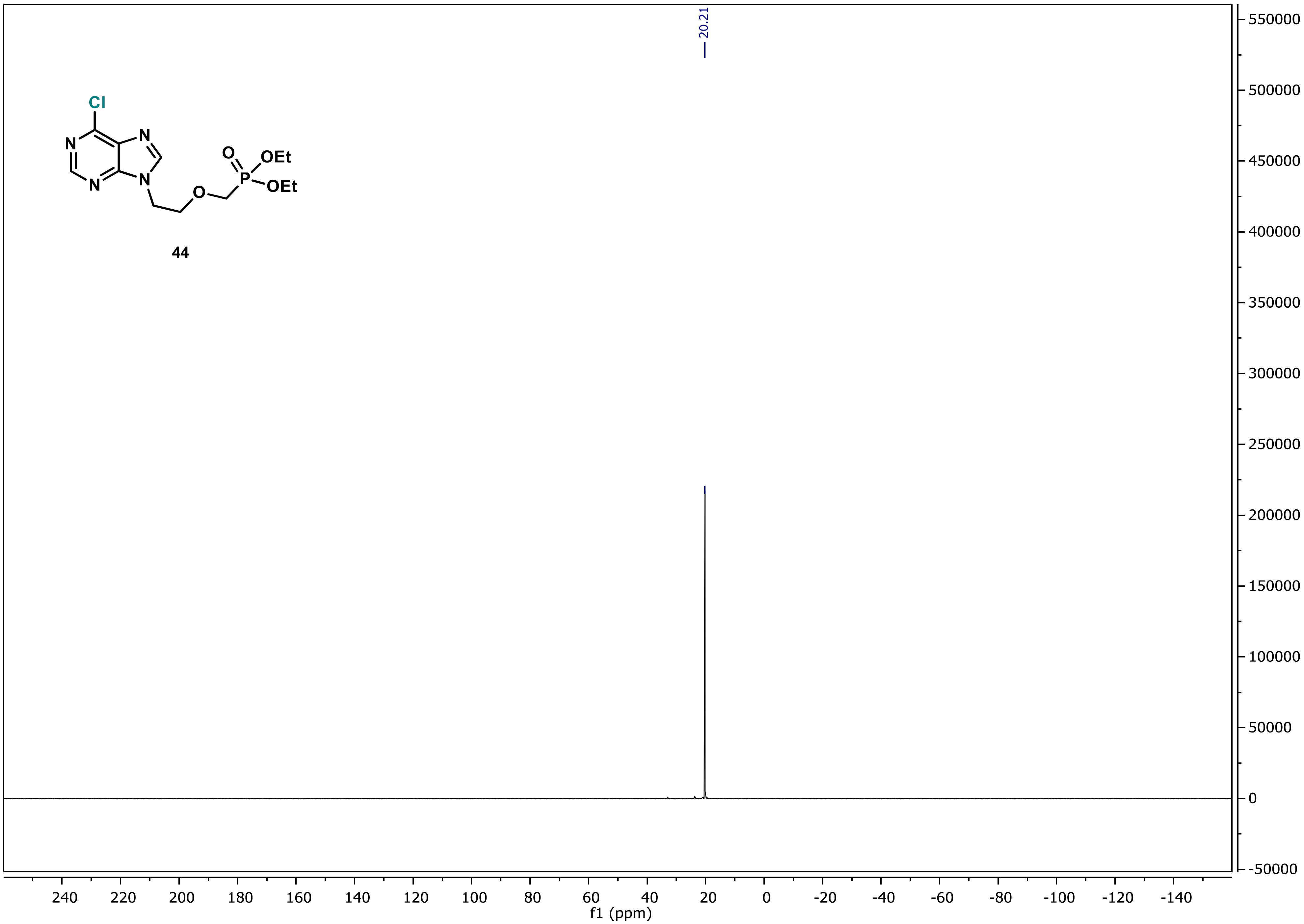

20.21
Cl
N
N
N
N
O
O
P
OEt
OEt
44
550000
500000
450000
400000
350000
300000
250000
200000
150000
100000
50000
0
-50000
240
220
200
180
160
140
120
100
80
60
40
20
0
-20
-40
-60
-80
-100
-120
-140
f1 (ppm)

*** Angegebene Mol.-Gewichte u. Massenzahlen basieren auf dem häufigsten Isotop der Elemente ***

MassLib

Mass to be matched (m/z): 371.064630 Charge: 1

Mass Tolerance: ±0.005000
Restriction of atom numbers:

C	H	N	O	Cl	P	Na
1-100	1-100	1-5	1-10	1-1	1-1	1-1

Number of calculated Formulas: 5

Formula	Diff.(ppm)	theor. m/z
C12 H18 N4 O4 Cl1 P1 Na1	0.03	371.064641
C14 H20 N1 O5 Cl1 P1 Na1	3.65	371.065985
C9 H20 N3 O7 Cl1 P1 Na1	-7.19	371.061963
C15 H16 N5 O1 Cl1 P1 Na1	7.25	371.067320
C17 H18 N2 O2 Cl1 P1 Na1	10.87	371.068664

Datum:	16.10.2020
Analyse:	149929b-00
Sigel:	GHC-GA-380-01
	COP: Dr. Clement Ghiazza
Method:	HR-MS
Ionis. :	ESIpos
solvent :	CH2Cl2 + CH3OH
Spectrometer:	Exactive
Auswerter:	Marcus, Tel:2243

suggestion:
C12H18N4O4Cl1P1 MW: 348

Characteristic Ions:
371 = [348 + Na]

^{1}H NMR

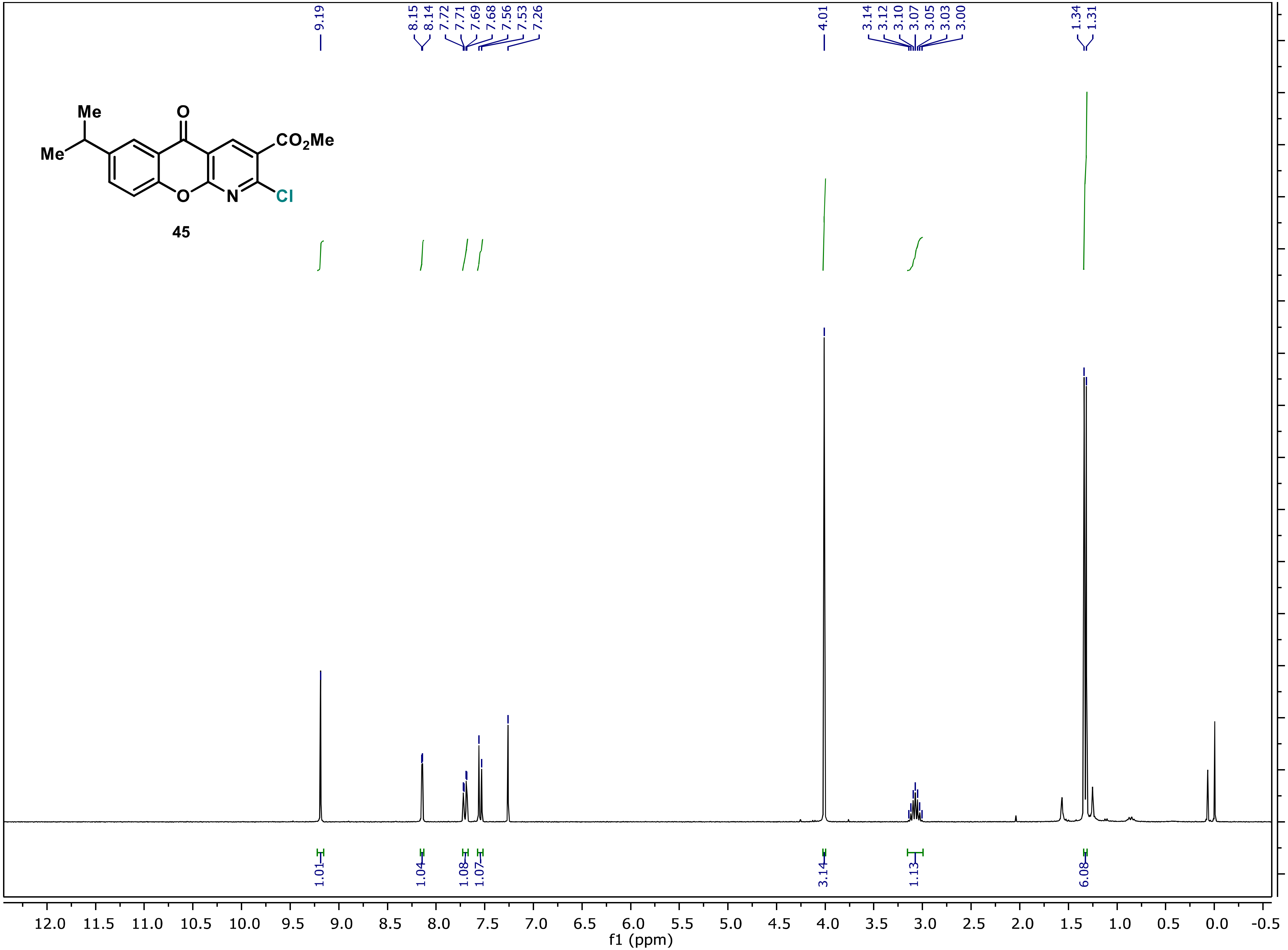

Me
O
CO2Me
Me
O
N
Cl
9.19
8.15
8.14
7.72
7.71
7.69
7.68
7.56
7.53
7.26
4.01
3.14
3.12
3.10
3.07
3.05
3.03
3.00
1.34
1.31
1.01
1.04
1.08
1.07
3.14
1.13
6.08
f1 (ppm)

45

^{13}C NMR

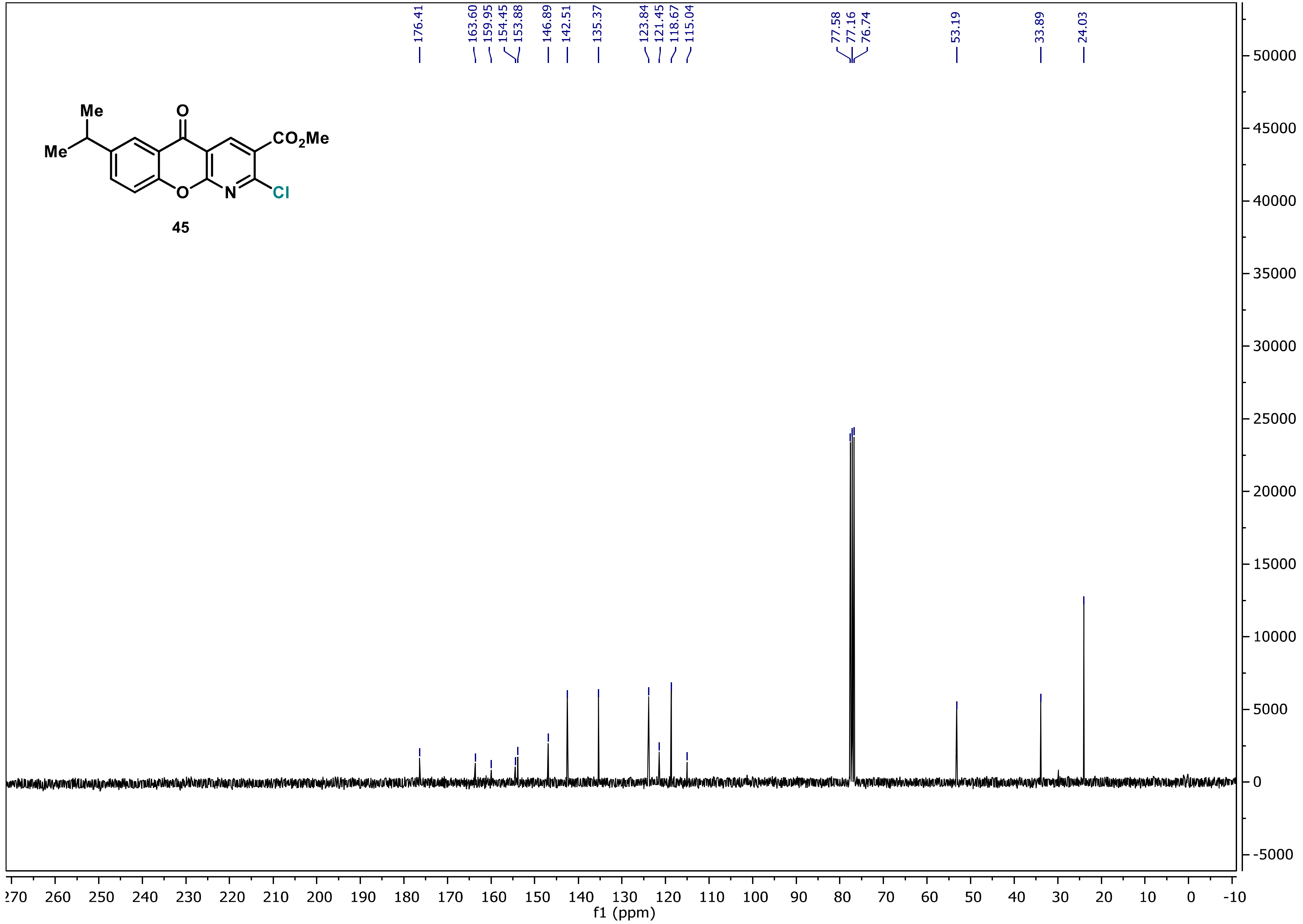
176.41
163.60
159.95
154.45
153.88
146.89
142.51
135.37
123.84
121.45
118.67
115.04
77.58
77.16
76.74
53.19
33.89
24.03
Me
O
CO2Me
Me
O
N
Cl
45
f1 (ppm)

Mass to be matched (m/z): 331.060640 Charge: 1

Mass Tolerance: ±0.005000
Restriction of atom numbers:

C	H	N	O	Cl
1-100	1-100	1-5	1-10	1-1

Number of calculated Formulas: 5

Formula	Diff.(ppm)	theor. m/z
C17 H14 N1 O4 Cl1	-0.16	331.060587
C15 H12 N4 O3 Cl1	-4.22	331.059243
C20 H12 N2 O1 Cl1	7.93	331.063265
C12 H14 N3 O6 Cl1	-12.31	331.056564
C8 H16 N4 O8 Cl1	13.53	331.065118

Datum: 7.08.2020
Analyse: 148567c-00

Sigel: GHC-GA-272-01
COP: Dr. Clement Ghiazza

Method: HR-MS
Ionis. : GC-EI
Spectrometer: Q-Exactive

Auswerter: Marcus, Tel:2243

suggestion:
C17H14N1O4Cl1   MW: 331

^{1}H NMR

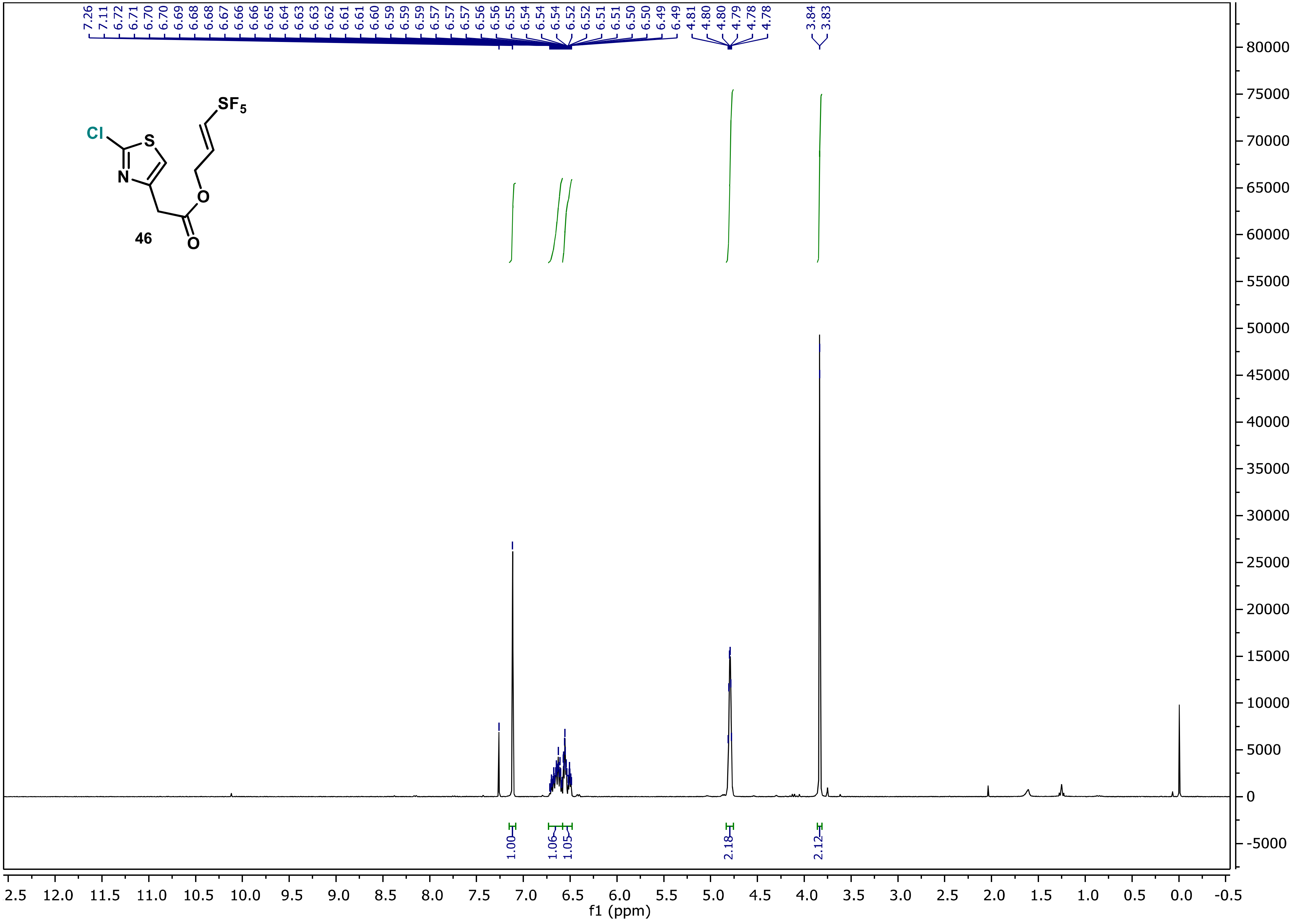

7.26
7.11
6.72
6.71
6.70
6.70
6.69
6.68
6.68
6.67
6.66
6.66
6.65
6.64
6.63
6.63
6.62
6.61
6.61
6.60
6.59
6.59
6.59
6.57
6.57
6.57
6.56
6.56
6.55
6.54
6.54
6.54
6.52
6.52
6.51
6.51
6.50
6.50
6.49
6.49
4.81
4.80
4.80
4.79
4.78
4.78
3.84
3.83
Cl
S
N
O
O
SF5
46
1.00
1.06
1.05
2.18
2.12
f1 (ppm)

^{13}C NMR

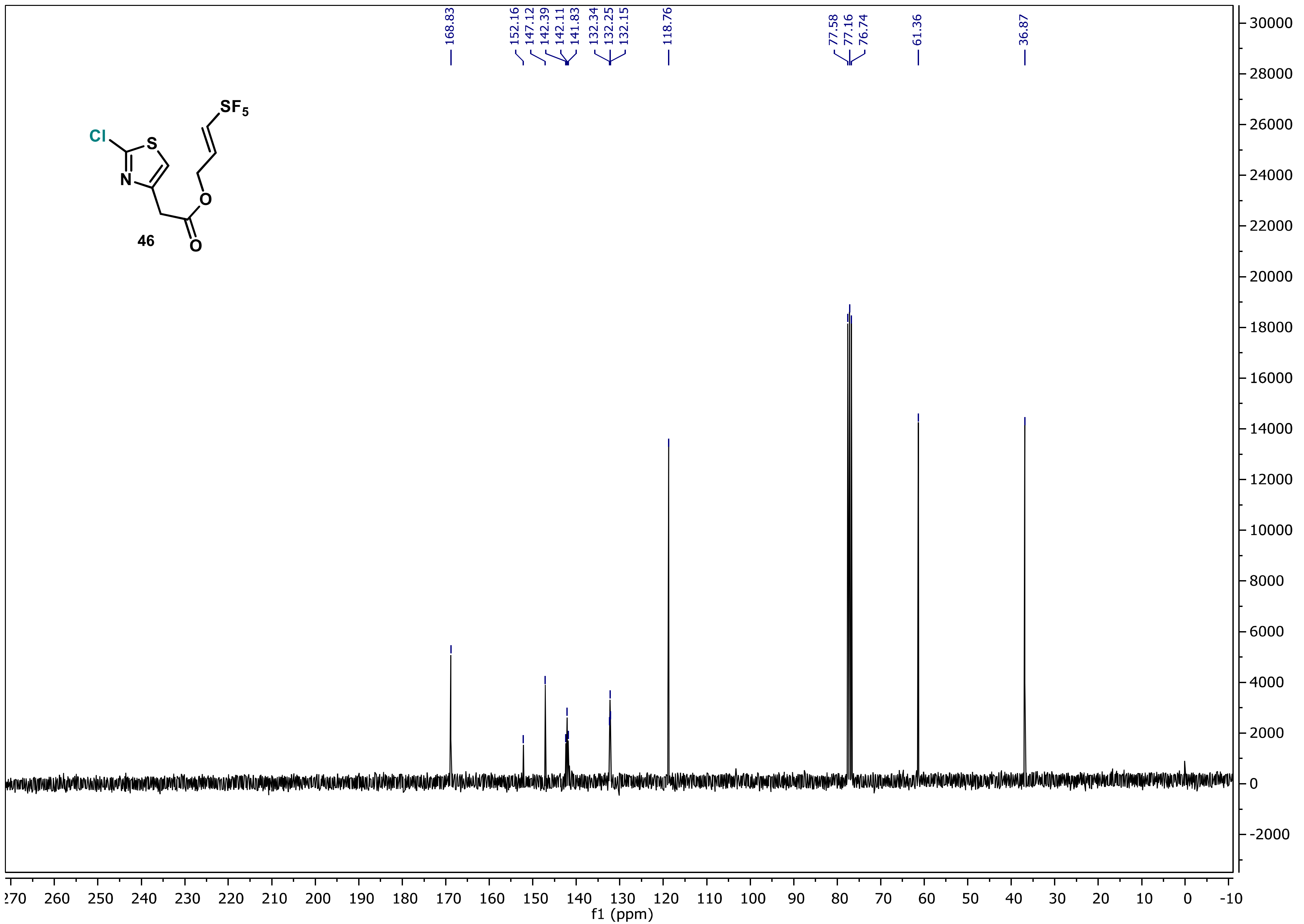
Cl
S
N
O
O
SF5
46
168.83
152.16
147.12
142.39
142.11
141.83
132.34
132.25
132.15
118.76
77.58
77.16
76.74
61.36
36.87
30000
28000
26000
24000
22000
20000
18000
16000
14000
12000
10000
8000
6000
4000
2000
0
-2000
270
260
250
240
230
220
210
200
190
180
170
160
150
140
130
120
110
100
90
80
70
60
50
40
30
20
10
0
-10
f1 (ppm)

^{19}F NMR

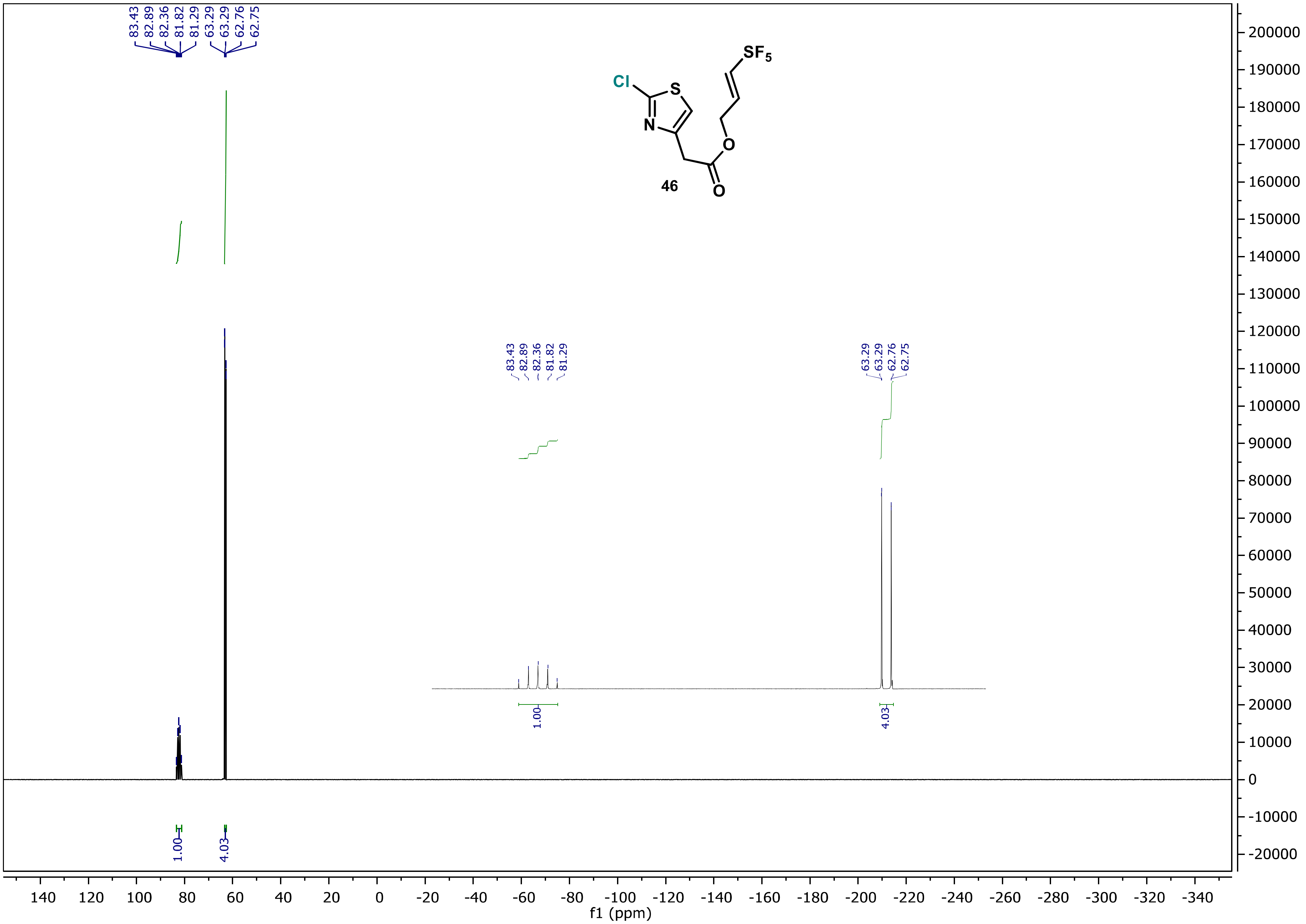
83.43
82.89
82.36
81.82
81.29
63.29
63.29
62.76
62.75
SF5
Cl
S
N
O
O
46
1.00
4.03
f1 (ppm)

*** Angegebene Mol.-Gewichte u. Massenzahlen basieren auf dem häufigsten Isotop der Elemente ***

MassLib

Mass to be matched (m/z): 343.959710 Charge: 1

Mass Tolerance: ±0.005000
Restriction of atom numbers:

C	H	Cl	F	N	O	S
1-100	1-100	1-1	5-5	1-3	max 5	2-2

Number of calculated Formulas: 1

Formula	Diff.(ppm)	theor. m/z
C8 H8 Cl1 F5 N1 O2 S2	0.75	343.959969

Datum	20.05.2021
Analyse:	153440e-00.RAW
Sigel:	GHC-GA-731-01
COP: Dr. Clement Ghiazza	
Messung:	HRMS
Methode:	ESIpos
Lösungsmittel:	CH2Cl2+CH3OH
Spektrometer:	Exactive
Auswerter:	Kampen (2242)

Suggestion:
C8H7Cl1F5N1O2S2 MW 343

characteristical ion
344 = [343 + H]+

¹H NMR

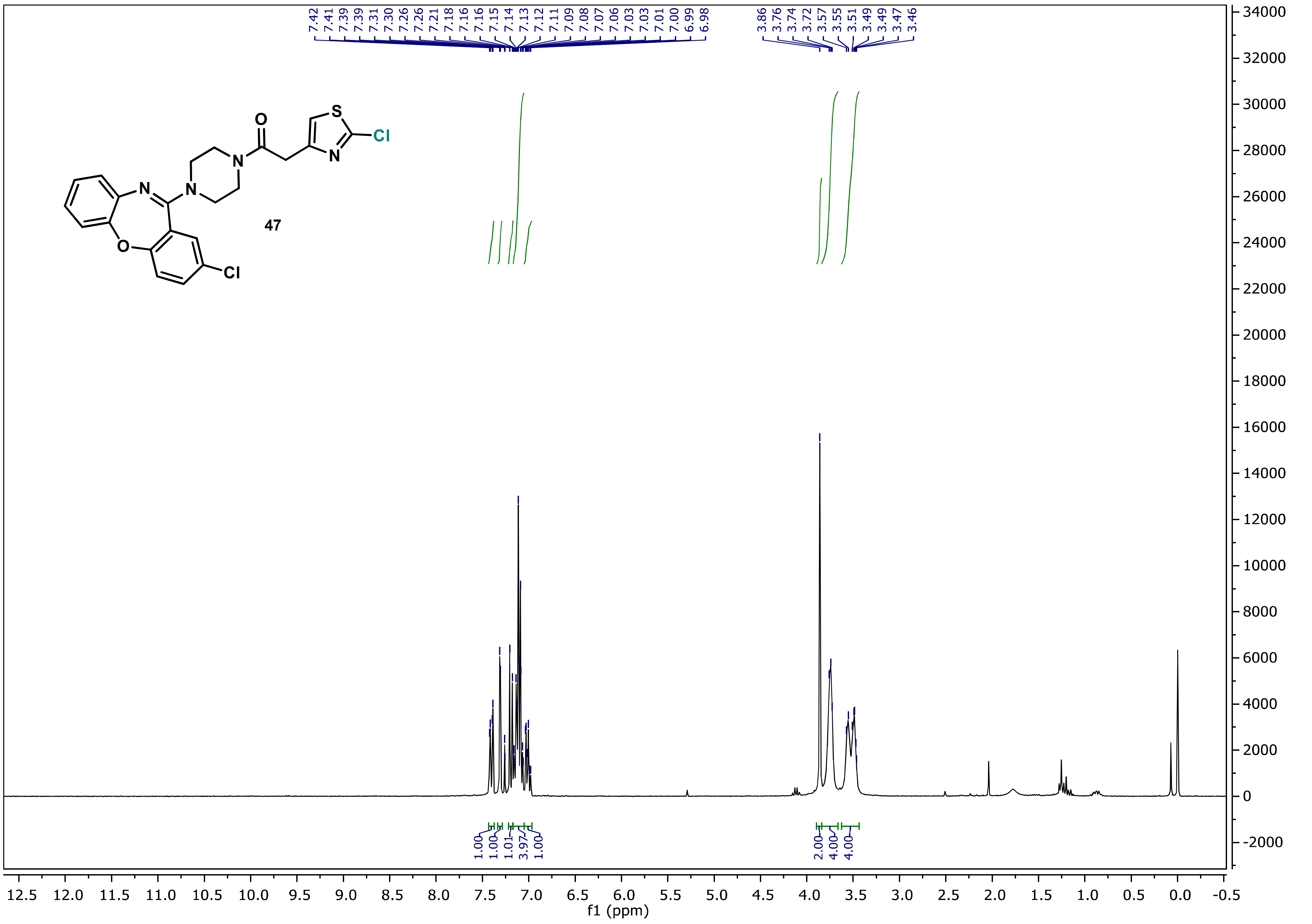

7.42 7.41 7.39 7.39 7.31 7.30 7.26 7.26 7.21 7.18 7.16 7.16 7.15 7.14 7.13 7.12 7.11 7.09 7.08 7.07 7.06 7.03 7.03 7.01 7.00 6.99 6.98
3.86 3.76 3.74 3.72 3.57 3.55 3.51 3.49 3.49 3.47 3.46
O
S
N
N
N
N
O
Cl
Cl
1.00 1.00 1.01 3.97 1.00
2.00 4.00 4.00
f1 (ppm)

47

^{13}C NMR

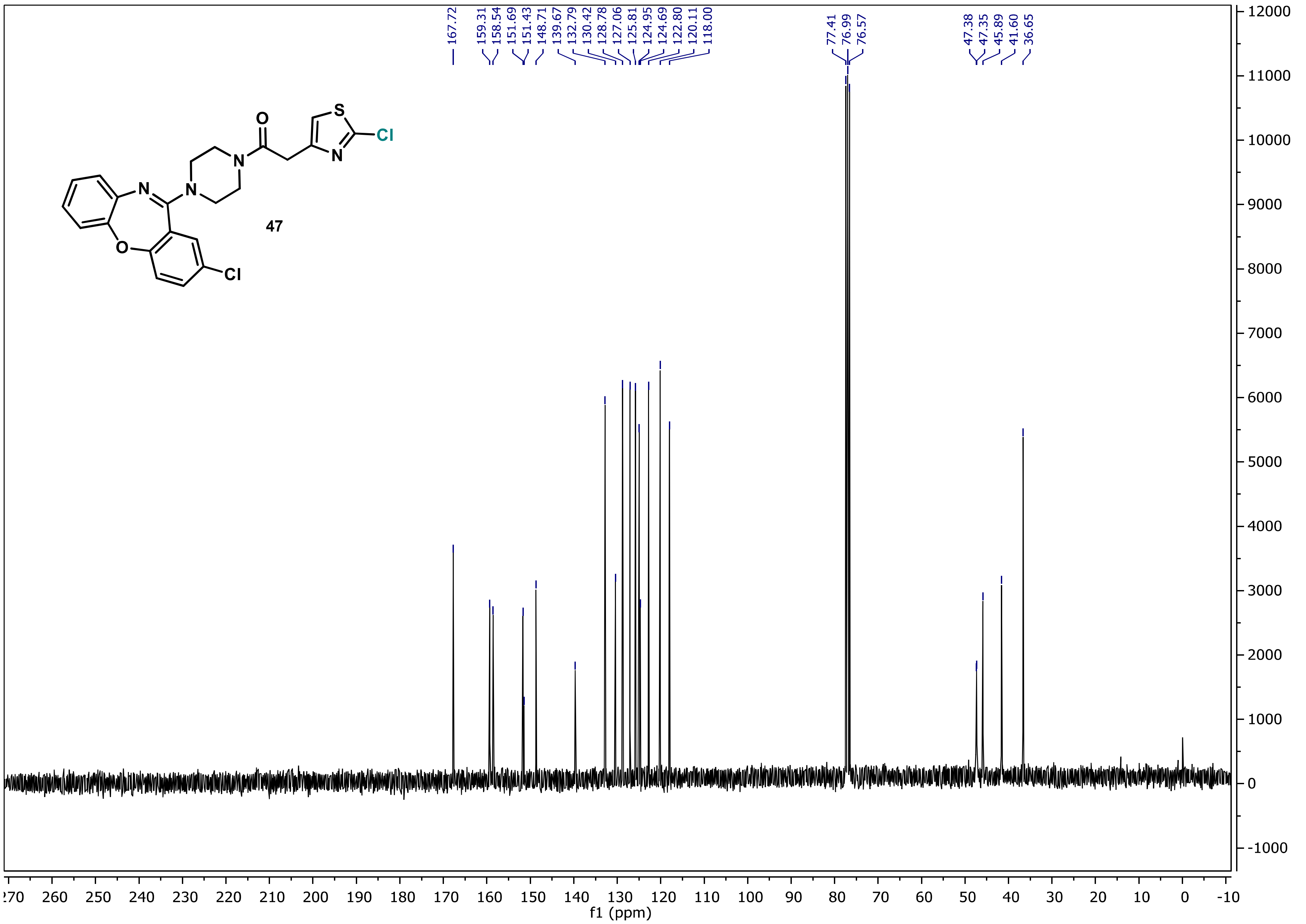

167.72
159.31
158.54
151.69
151.43
148.71
139.67
132.79
130.42
128.78
127.06
125.81
124.95
124.69
122.80
120.11
118.00
77.41
76.99
76.57
47.38
47.35
45.89
41.60
36.65
47
f1 (ppm)

Mass to be matched (m/z): 473.059970 Charge: 1

Mass Tolerance: ±0.005000

Restriction of atom numbers:

C	H	N	O	S	Cl
1-100	1-100	1-5	1-10	1-1	2-2

Number of calculated Formulas: 4

Formula	Diff.(ppm)	theor. m/z
C22 H19 N4 O2 S1 Cl2	0.12	473.060028
C24 H21 N1 O3 S1 Cl2	2.96	473.061372
C19 H21 N3 O5 S1 Cl2	-5.54	473.057350
C12 H25 N3 O10 S1 Cl2	6.88	473.063225

Datum:	30.10.2020
Analyse:	150219c-00
Sigel:	GHC-GA-410-01<br>COP: Dr. Clement Ghiazza
Method:	HR-MS
Ionis. :	ESIpos
solvent :	CH3OH
Spectrometer:	Exactive
Auswerter:	Marcus, Tel:2243

suggestion:
C22H18N4O2S1Cl2 MW: 472

Characteristic Ions:
473 = [472 + H]

^{1}H NMR

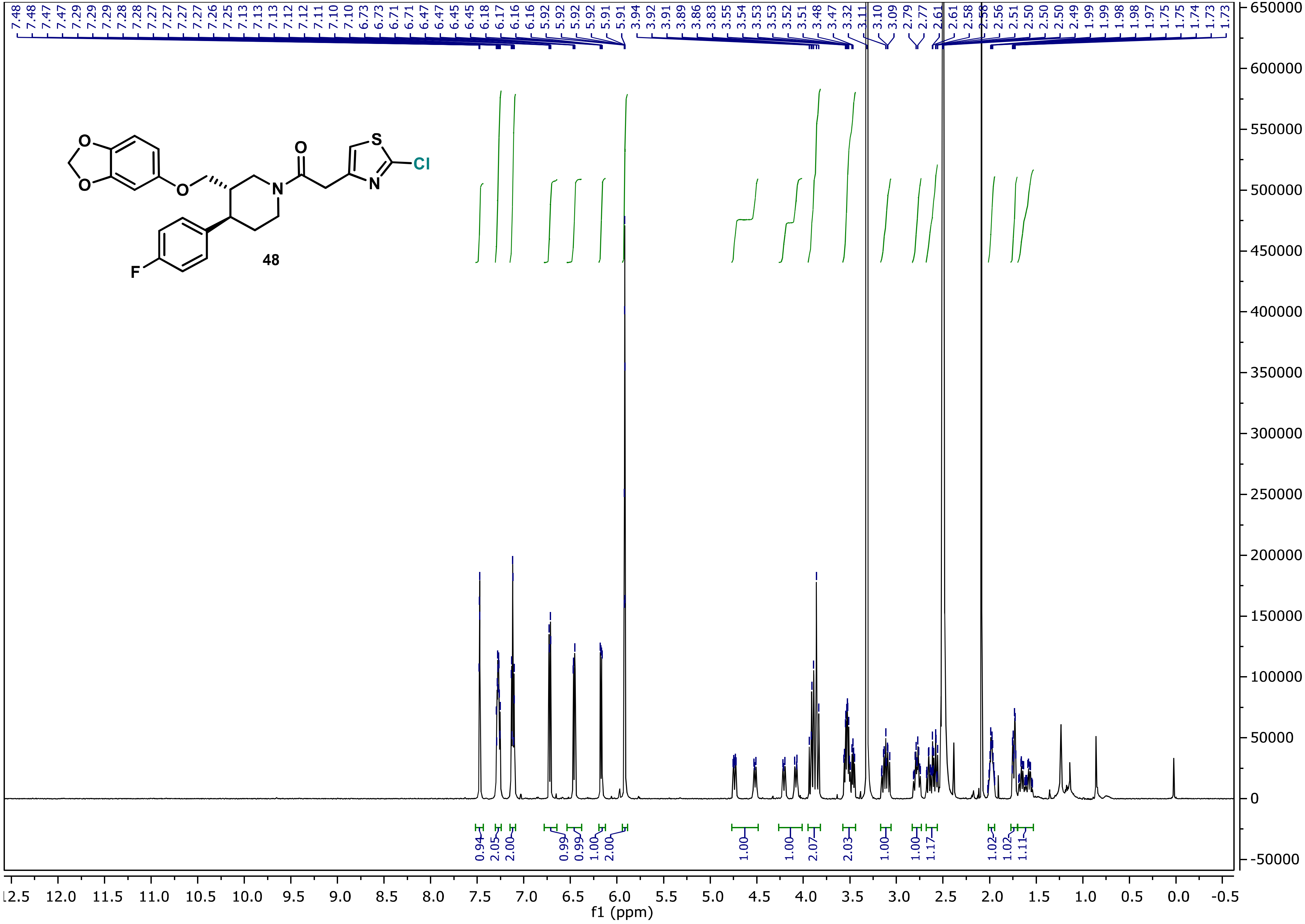
7.48 7.48 7.47 7.47 7.29 7.29 7.28 7.28 7.27 7.27 7.27 7.27 7.26 7.25 7.13 7.13 7.13 7.12 7.12 7.11 7.10 7.10 6.73 6.73 6.71 6.71 6.47 6.47 6.45 6.45 6.18 6.17 6.16 6.16 5.92 5.92 5.92 5.92 5.91 5.91
3.94 3.92 3.91 3.89 3.86 3.83 3.55 3.54 3.53 3.53 3.52 3.51 3.48 3.47 3.32 3.11 3.10 3.09 2.79 2.77 2.61 2.61 2.58 2.58 2.56 2.51 2.50 2.50 2.50 2.49 1.99 1.99 1.98 1.98 1.97 1.75 1.75 1.74 1.73 1.73
O
O
O
N
O
S
N
Cl
F
48
0.94 2.05 2.00 0.99 0.99 1.00 2.00 1.00 1.00 2.07 2.03 1.00 1.00 1.17 1.02 1.02 1.11
f1 (ppm)

^{13}C NMR

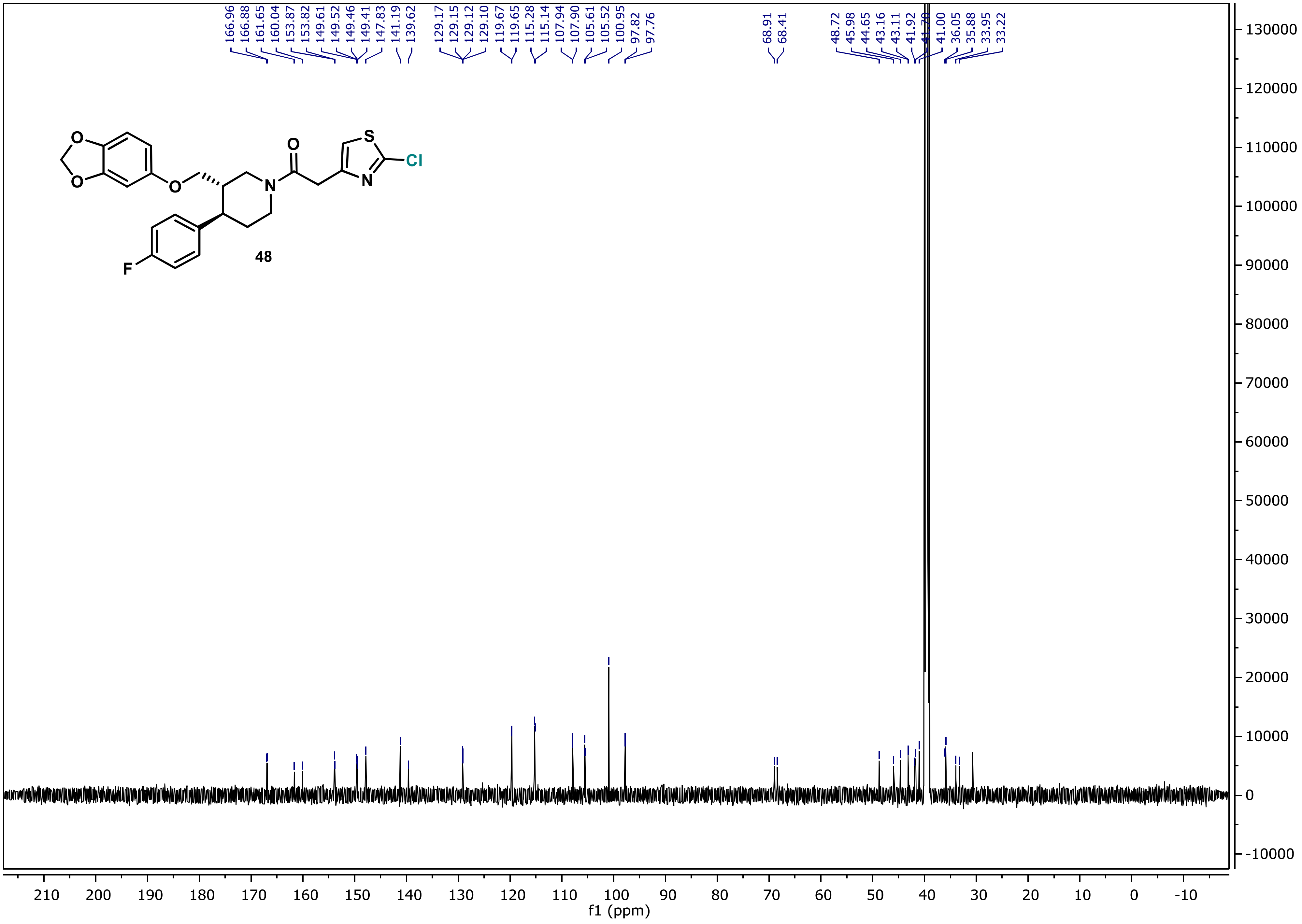

166.96
166.88
161.65
160.04
153.87
153.82
149.61
149.52
149.46
149.41
147.83
141.19
139.62
129.17
129.15
129.12
129.10
119.67
119.65
115.28
115.14
107.94
107.90
105.61
105.52
100.95
97.82
97.76
68.91
68.41
48.72
45.98
44.65
43.16
43.11
41.92
41.78
41.00
36.05
35.88
33.95
33.22
O
O
O
O
N
S
N
Cl
F
130000
120000
110000
100000
90000
80000
70000
60000
50000
40000
30000
20000
10000
0
-10000
210
200
190
180
170
160
150
140
130
120
110
100
90
80
70
60
50
40
30
20
10
0
-10
f1 (ppm)

48

^{19}F NMR

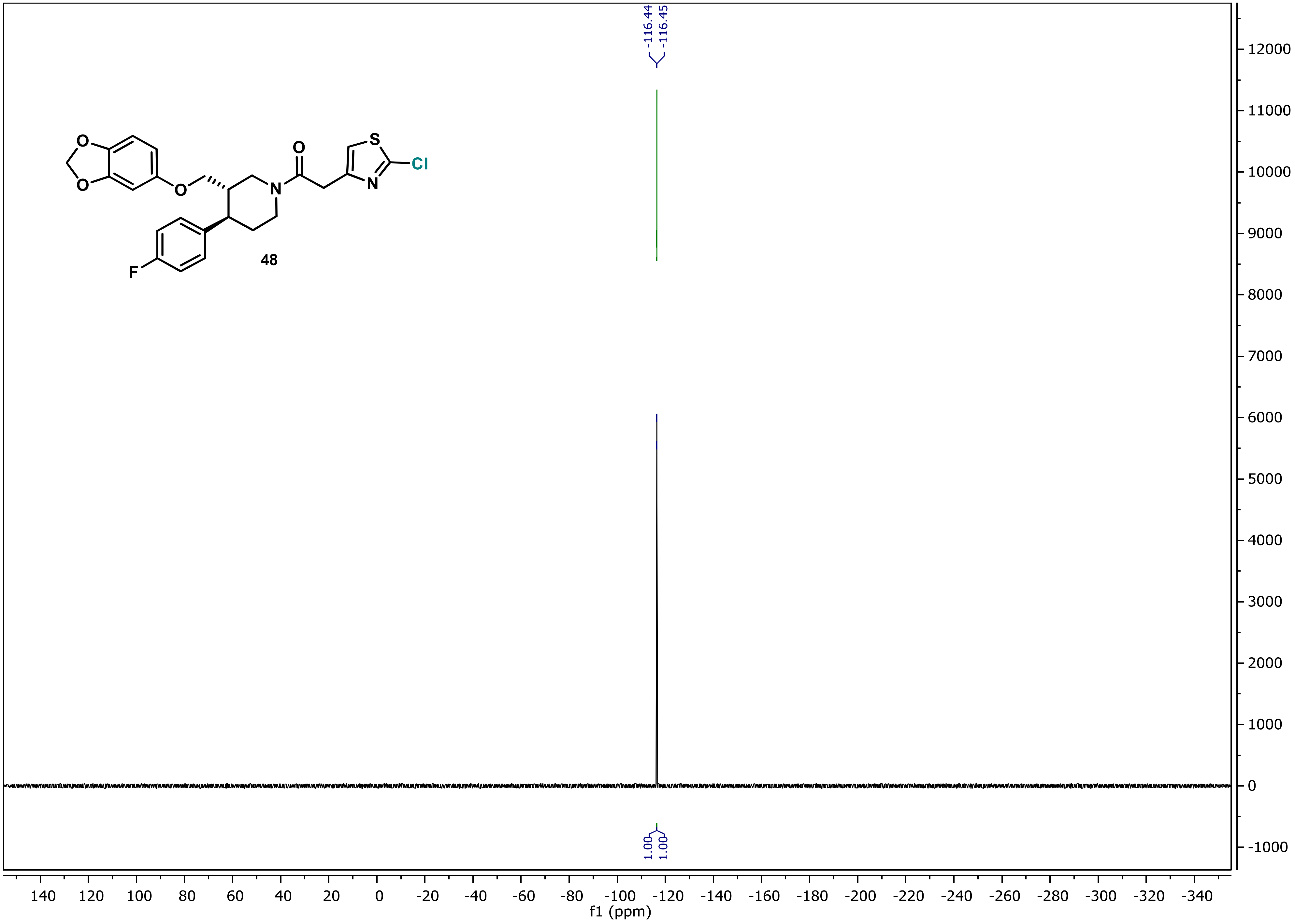

Mass to be matched (m/z): 511.086620 Charge: 1

Mass Tolerance: ±0.005000

Restriction of atom numbers:

C	H	Cl	F	N	O	S	Na
1-100	1-100	1-1	1-1	max 2	max 5	1-1	1-1

Number of calculated Formulas: 2

Formula	Diff.(ppm)	theor. m/z
C24 H22 Cl1 F1 N2 O4 S1 Na1	-0.22	511.086506
C29 H22 Cl1 F1 O2 S1 Na1	7.65	511.090528

Datum	2.03.2021
Analyse:	152006b-00
Sigel:	GHC-GA-618-01
COP: Dr. Clement Ghiazza	
Messung:	HRMS
Methode:	ESIpos
Lösungsmittel:	CH2Cl2+CH3OH
Spektrometer:	Exactive
Auswerter:	Kampen (2242)

Suggestion:
C24H22Cl1F1N2O4S1 MW 488

characteristical ion
511 = [488 + Na]+

^{1}H NMR

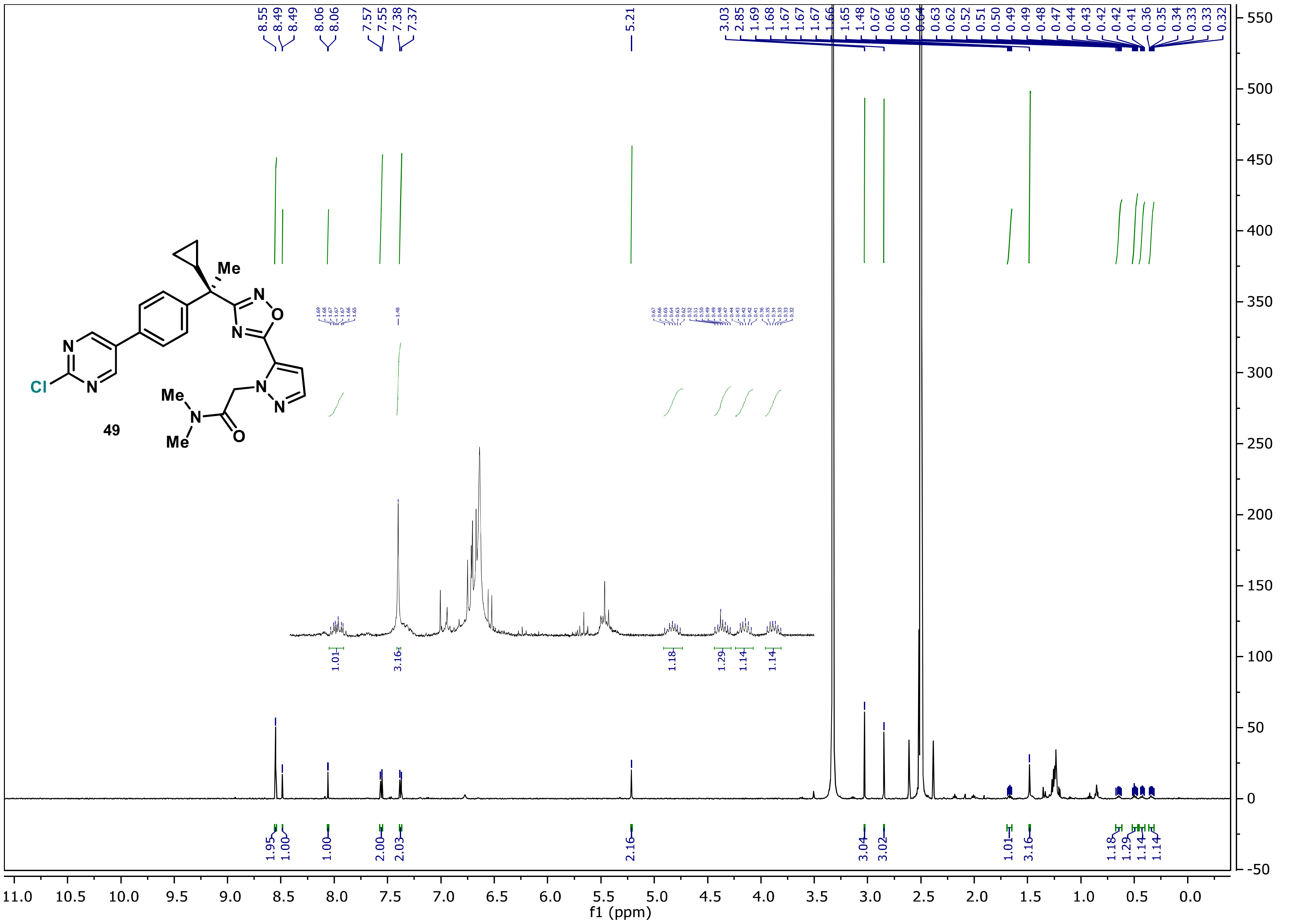

^{13}C NMR

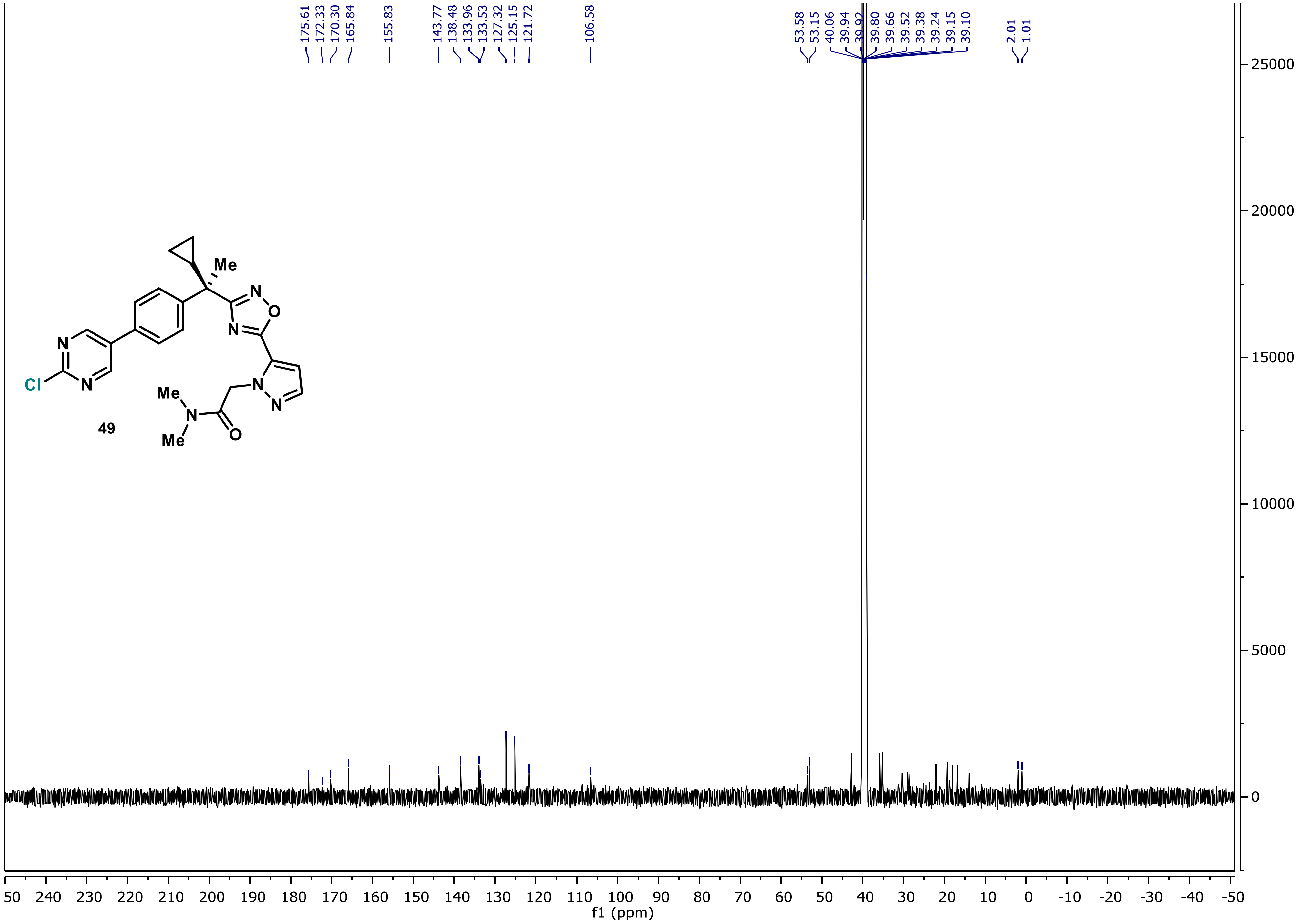
175.61
172.33
170.30
165.84
155.83
143.77
138.48
133.96
133.53
127.32
125.15
121.72
106.58
53.58
53.15
40.06
39.94
39.92
39.80
39.66
39.52
39.38
39.24
39.15
39.10
2.01
1.01
25000
20000
15000
10000
5000
0
f1 (ppm)
Cl
N
Me
O
Me
Me

49

Mass to be matched (m/z): 478.174910 Charge: 1

Mass Tolerance: ±0.050000
Restriction of atom numbers:

C	H	N	O	Cl
1-100	1-100	7-7	1-3	1-2

Number of calculated Formulas: 6

Formula	Diff.(ppm)	theor. m/z
C24 H25 N7 O2 Cl1	0.76	478.175274
C22 H30 N7 O1 Cl2	28.08	478.188337
C21 H26 N7 O2 Cl2	-48.01	478.151952
C19 H34 N7 O3 Cl2	72.27	478.209467
C23 H21 N7 O3 Cl1	-75.33	478.138889
C25 H29 N7 O1 Cl1	76.85	478.211659

12.11.2020
File: 150394c-00
Analysis: GHC-GA-419-01
COP: Dr. Clement Ghiazza

Messung:	HR-MS
Ionisierung:	ESIpos
Lösungsmittel:	CH2Cl2 + CH3OH
Spektrometer:	Exactive
ELNA:	28360

Auswerter: Haupt (2243)

Suggestion:
C24H24Cl1N7O2 MW: 477

Characteristic ions:
478 = [477 + H]+

¹H NMR

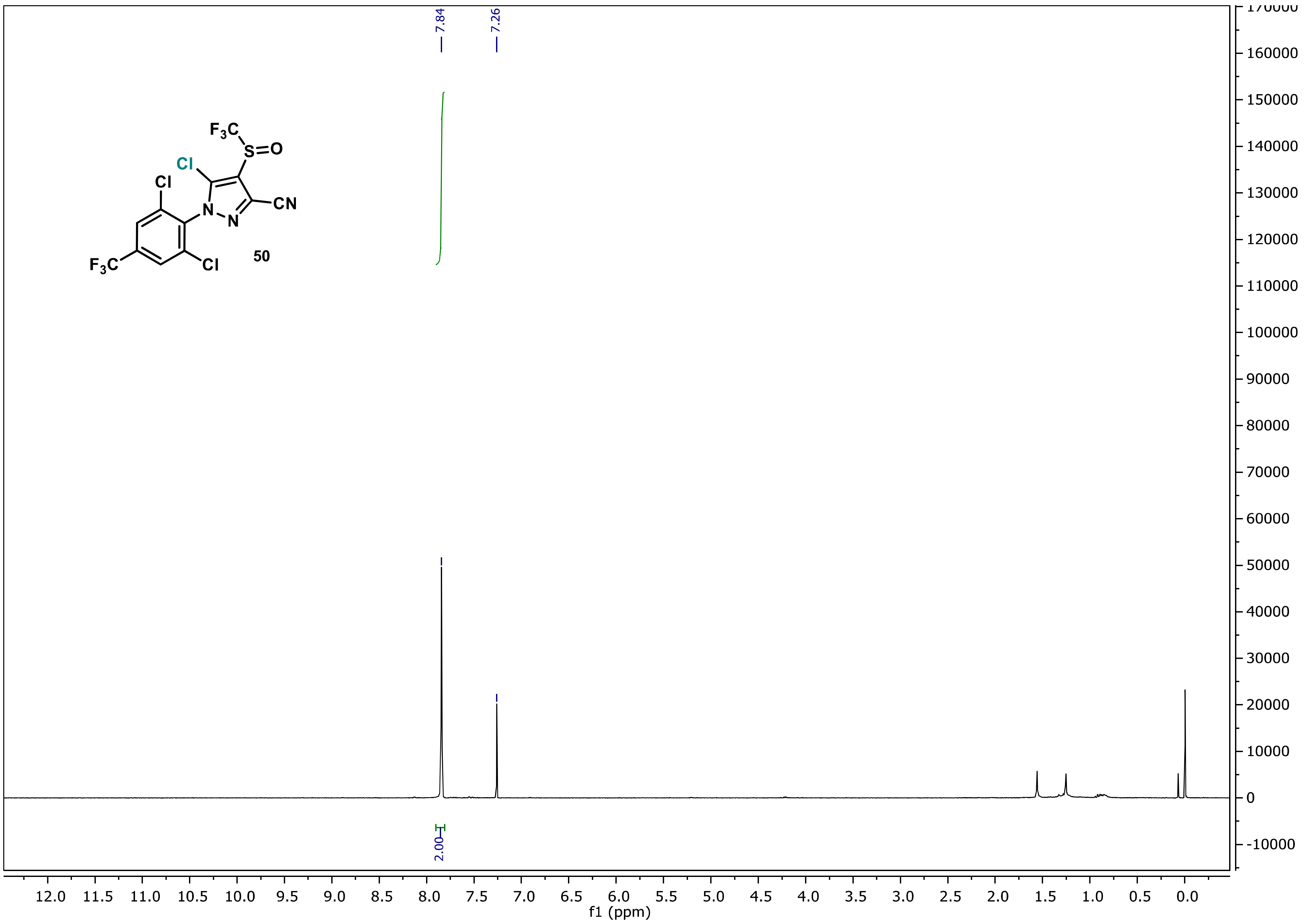

^{13}C NMR

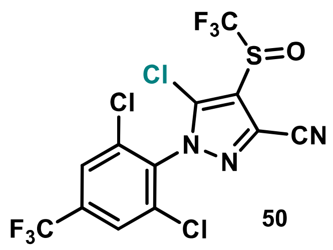

50

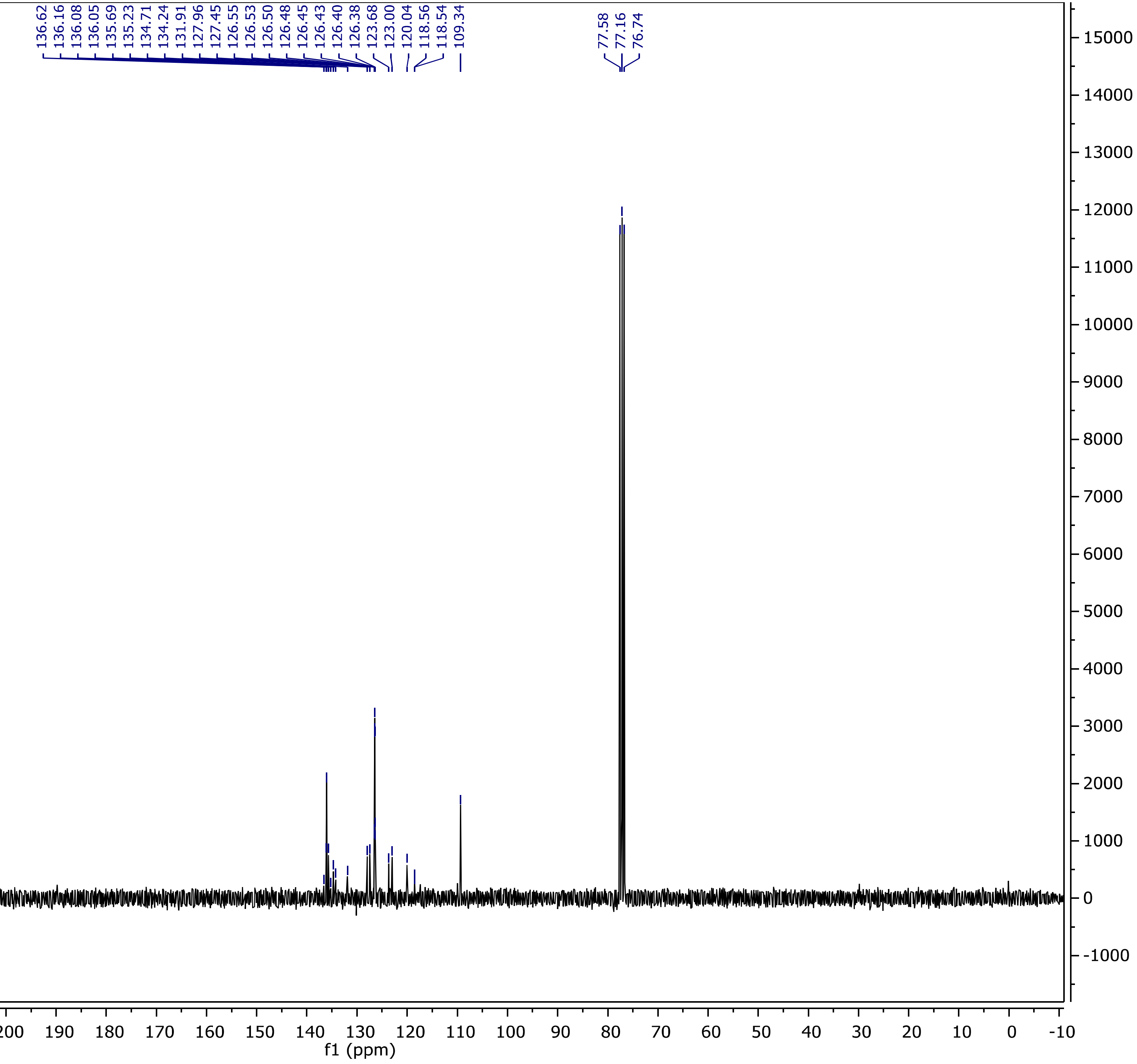

136.62
136.16
136.08
136.05
135.69
135.23
134.71
134.24
131.91
127.96
127.45
126.55
126.53
126.50
126.48
126.45
126.43
126.40
126.38
123.68
123.00
120.04
118.56
118.54
109.34
77.58
77.16
76.74
f1 (ppm)

^{19}F NMR

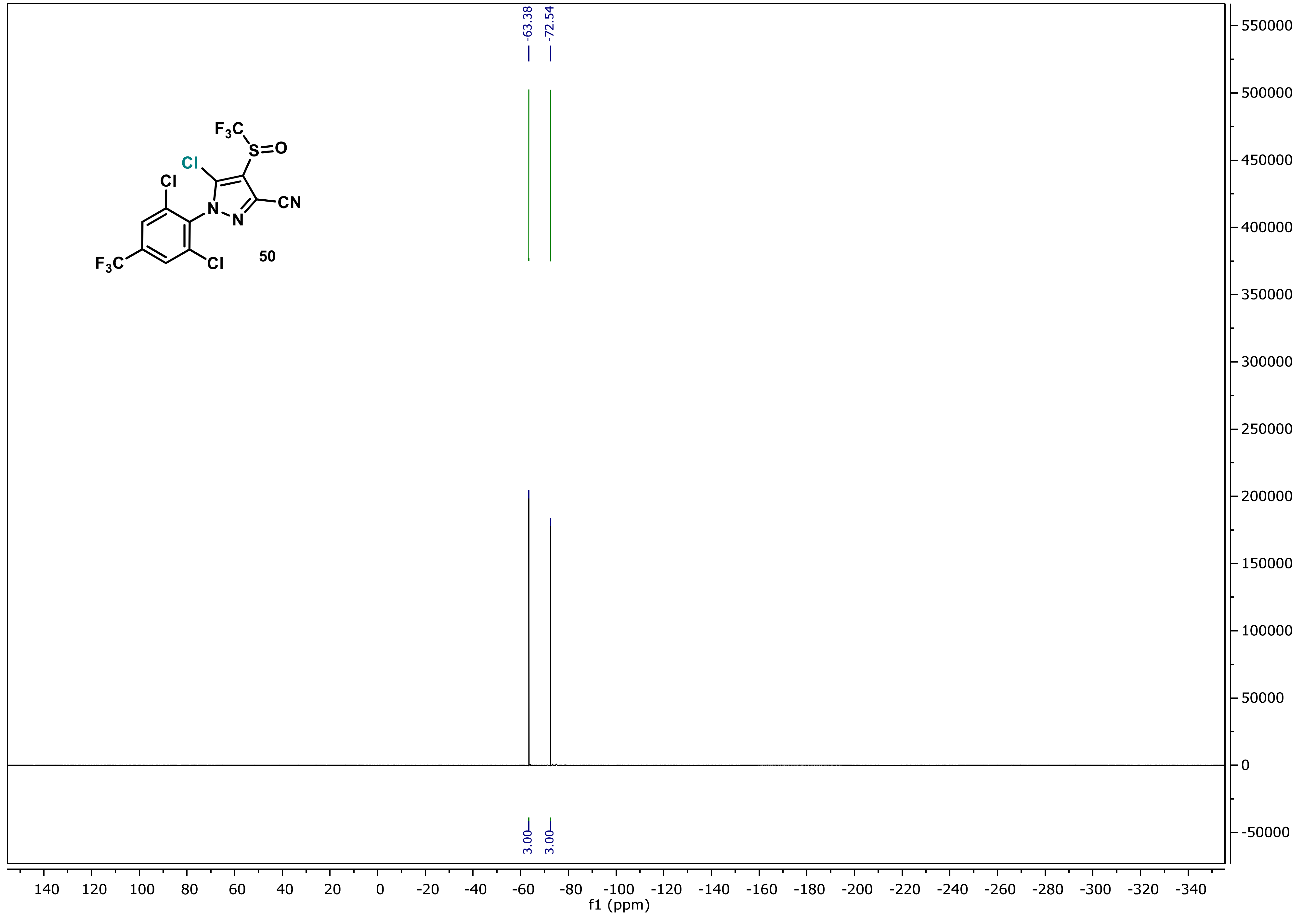

-63.38
-72.54
F3C
S=O
Cl
Cl
N
N
CN
F3C
Cl
50
3.00
3.00
550000
500000
450000
400000
350000
300000
250000
200000
150000
100000
50000
0
-50000
140 120 100 80 60 40 20 0 -20 -40 -60 -80 -100 -120 -140 -160 -180 -200 -220 -240 -260 -280 -300 -320 -340
f1 (ppm)

Mass to be matched (m/z): 477.877890 Charge: 1

Mass Tolerance: ±0.005000
Restriction of atom numbers:

C	H	Cl	F	N	O	S	Na
1-100	1-100	3-3	6-6	1-3	max 5	1-1	1-1

Number of calculated Formulas: 2

Formula	Diff.(ppm)	theor. m/z
C12 H2 Cl3 F6 N3 O1 S1 Na1	0.35	477.878058
C9 H4 Cl3 F6 N2 O4 S1 Na1	-5.25	477.875379

Datum	31.07.2020
Analyse:	148457b-00.raw
Sigel:	GHC-GA-243-01
COP: Dr. Clement Ghiazza	
Messung:	HRMS
Methode:	ESIpos
Lösungsmittel:	CH2Cl2+CH3OH
Spektrometer:	LTQ FT
Auswerter:	Kampen (2242)

Suggestion:
C12H2Cl3F6N3O1S1 MW 455

characteristical ion
478 = [455 + Na]+

^{1}H NMR

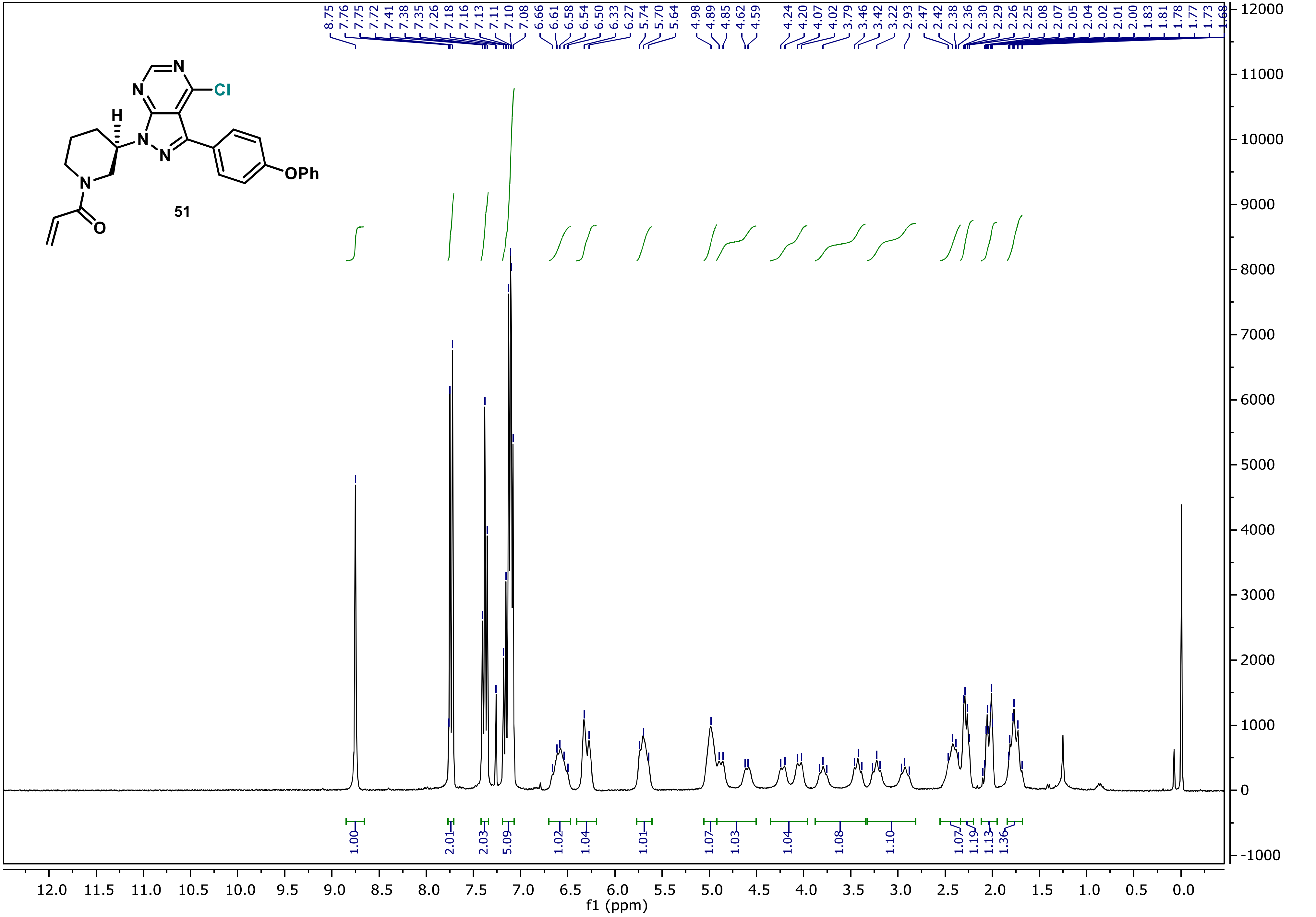

51
8.75 7.76 7.75 7.72 7.41 7.38 7.35 7.26 7.18 7.16 7.13 7.11 7.10 7.08 6.66 6.61 6.58 6.54 6.50 6.33 6.27 5.74 5.70 5.64 4.98 4.89 4.85 4.62 4.59 4.24 4.20 4.07 4.02 3.79 3.46 3.42 3.22 2.93 2.47 2.42 2.38 2.36 2.30 2.29 2.26 2.25 2.08 2.07 2.05 2.04 2.02 2.01 2.00 1.83 1.81 1.78 1.77 1.73 1.68
1.00 2.01 2.03 5.09 1.02 1.04 1.01 1.07 1.03 1.04 1.08 1.10 1.07 1.19 1.13 1.36
f1 (ppm)

^{13}C NMR

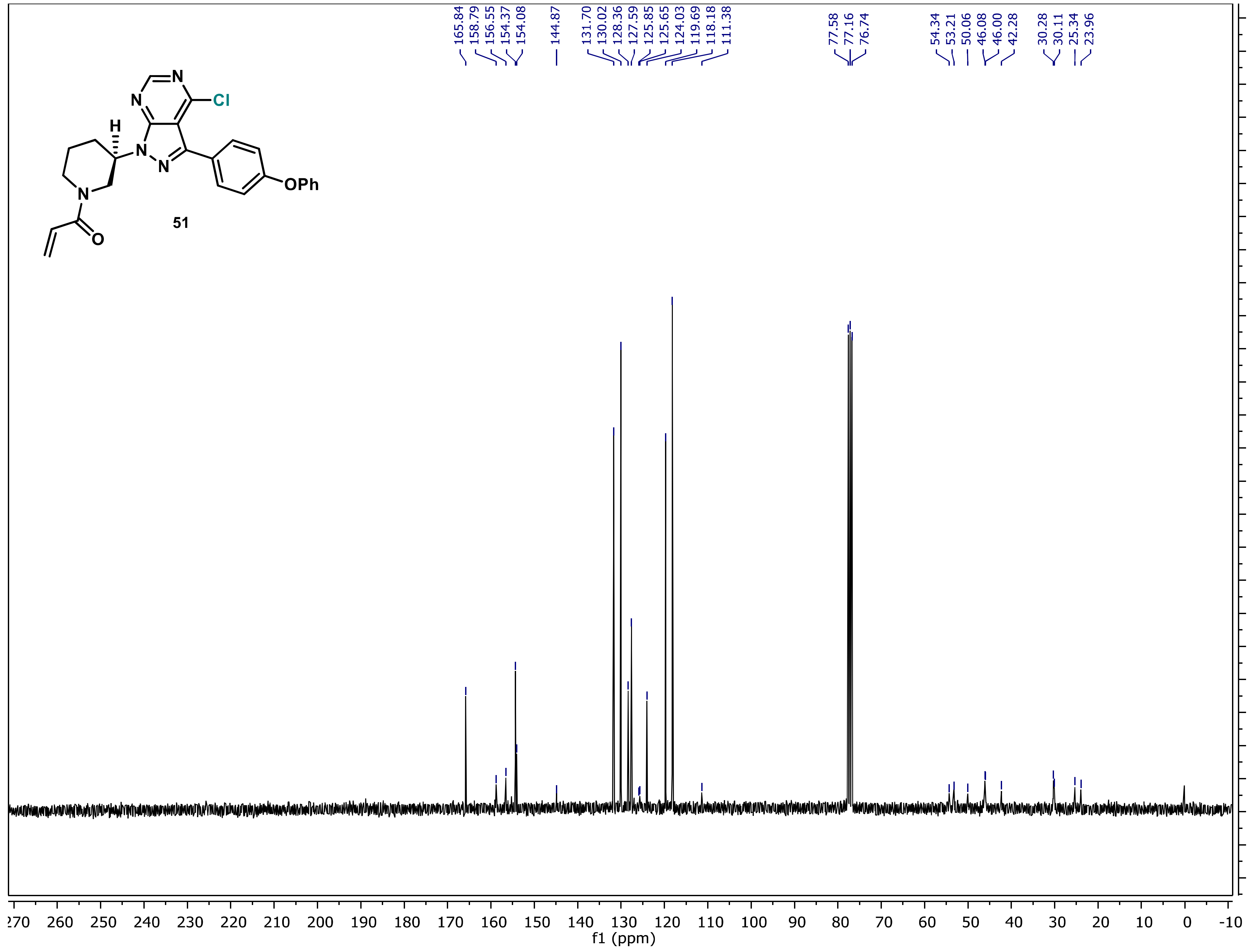

165.84
158.79
156.55
154.37
154.08
144.87
131.70
130.02
128.36
127.59
125.85
125.65
124.03
119.69
118.18
111.38
77.58
77.16
76.74
54.34
53.21
50.06
46.08
46.00
42.28
30.28
30.11
25.34
23.96
N
N
Cl
H
N
N
N
OPh
O
51
f1 (ppm)

Mass to be matched (m/z): 460.153380 Charge: 1

Mass Tolerance: ±0.005000

Restriction of atom numbers:

C	H	Cl	N	O
1-100	1-100	1-1	1-5	max 5

Number of calculated Formulas: 4

Formula	Diff.(ppm)	theor. m/z
C25 H23 Cl1 N5 O2	0.21	460.153476
C27 H25 Cl1 N2 O3	3.13	460.154821
C22 H25 Cl1 N4 O5	-5.61	460.150798
C30 H23 Cl1 N3	8.95	460.157499

Datum	11.08.2020
Analyse:	148633b-00
Sigel:	GHC-GA-268-01
COP: Dr. Clement Ghiazza	
Messung:	HRMS
Methode:	ESIpos
Lösungsmittel:	CH2Cl2+CH3OH
Spektrometer:	Exactive
Auswerter:	Kampen (2242)

Suggestion:
C25H22Cl1N5O2 MW 459

characteristical ion
460 = [459 + H]+

^{1}H NMR

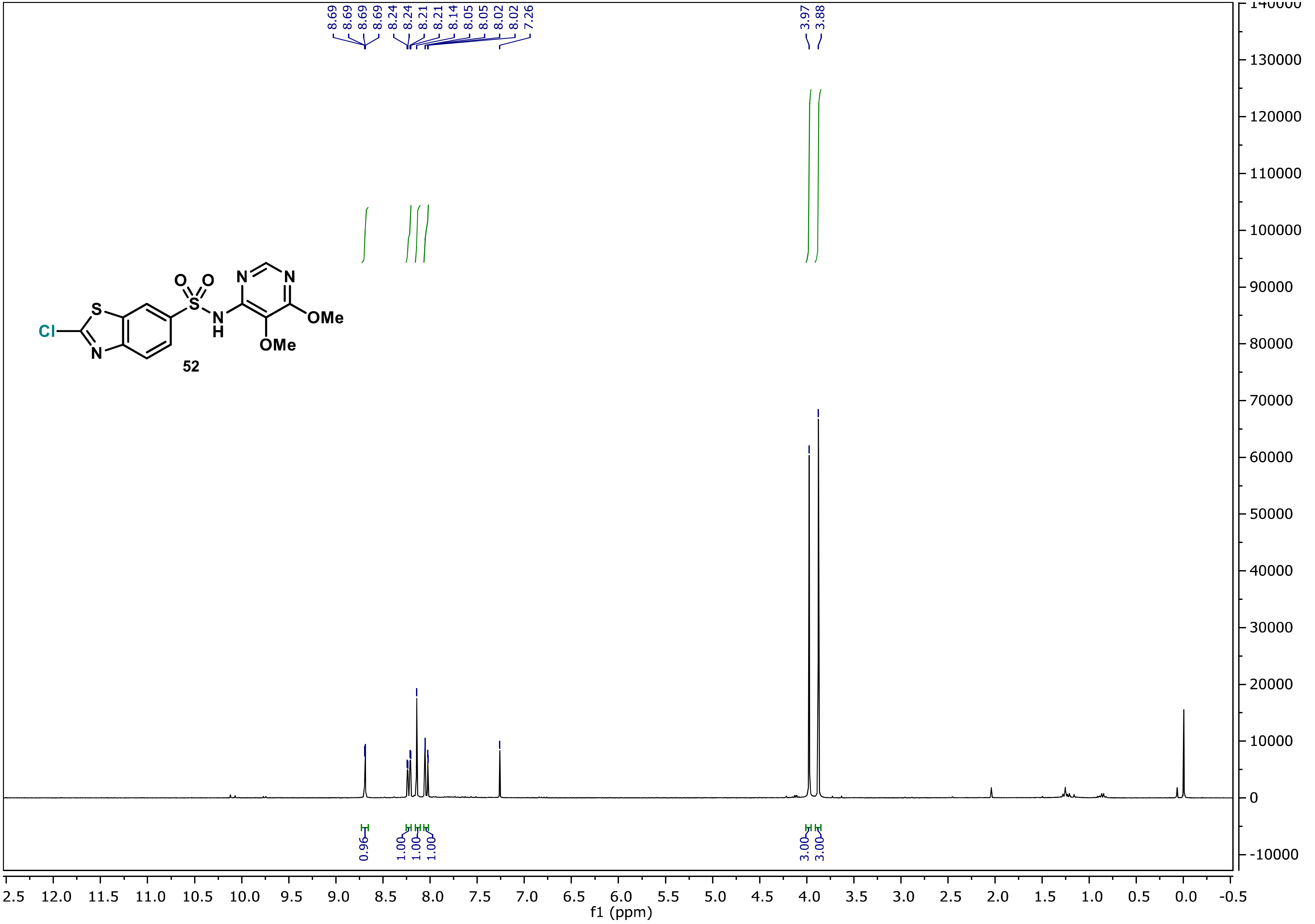

8.69
8.69
8.69
8.69
8.24
8.24
8.21
8.21
8.14
8.05
8.05
8.02
8.02
7.26
3.97
3.88
0.96
1.00
1.00
1.00
3.00
3.00
f1 (ppm)

52

^{13}C NMR

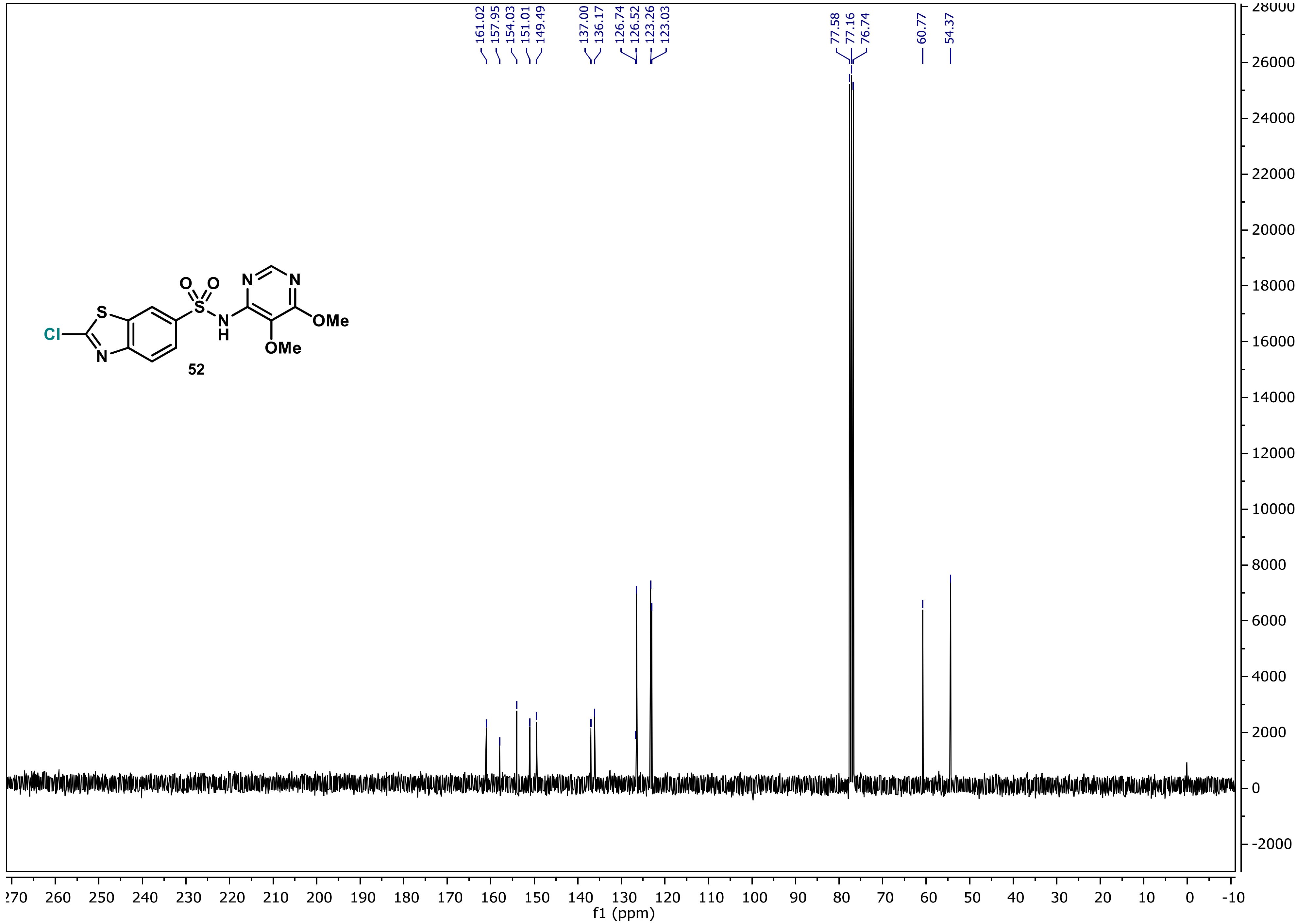

161.02
157.95
154.03
151.01
149.49
137.00
136.17
126.74
126.52
123.26
123.03
77.58
77.16
76.74
60.77
54.37
Cl
S
N
O
O
N
H
N
N
OMe
OMe
52
f1 (ppm)

*** Angegebene Mol.-Gewichte u. Massenzahlen basieren auf dem häufigsten Isotop der Elemente ***

MassLib

Mass to be matched (m/z): 386.998240 Charge: 1

Mass Tolerance: ±0.005000
Restriction of atom numbers:

C	H	N	O	Cl	S
1-100	1-100	1-10	1-10	1-1	2-2

Number of calculated Formulas: 8

Formula	Diff.(ppm)	theor. m/z
C13 H12 N4 O4 Cl1 S2	0.16	386.998303
C11 H10 N7 O3 Cl1 S2	-3.31	386.996959
C15 H14 N1 O5 Cl1 S2	3.63	386.999647
C10 H14 N3 O7 Cl1 S2	-6.76	386.995624
C9 H8 N10 O2 Cl1 S2	-6.78	386.995615
C16 H10 N5 O1 Cl1 S2	7.08	387.000982
C8 H12 N6 O6 Cl1 S2	-10.23	386.994280
C18 H12 N2 O2 Cl1 S2	10.56	387.002326

Datum:	17.05.2021
Analyse:	153345c-00
Sigel:	GHC-GA-718-01
	COP: Dr. Clement Ghiazza
Method:	HR-MS
Ionis. :	ESIpos
solvent :	CH2Cl2 + CH3OH
Spectrometer:	Exactive
Auswerter:	Marcus, Tel:2243

suggestion:
C13H11N4O4Cl1S2 MW: 386

Characteristic Ions:
387 = [386 + H]

^1H NMR

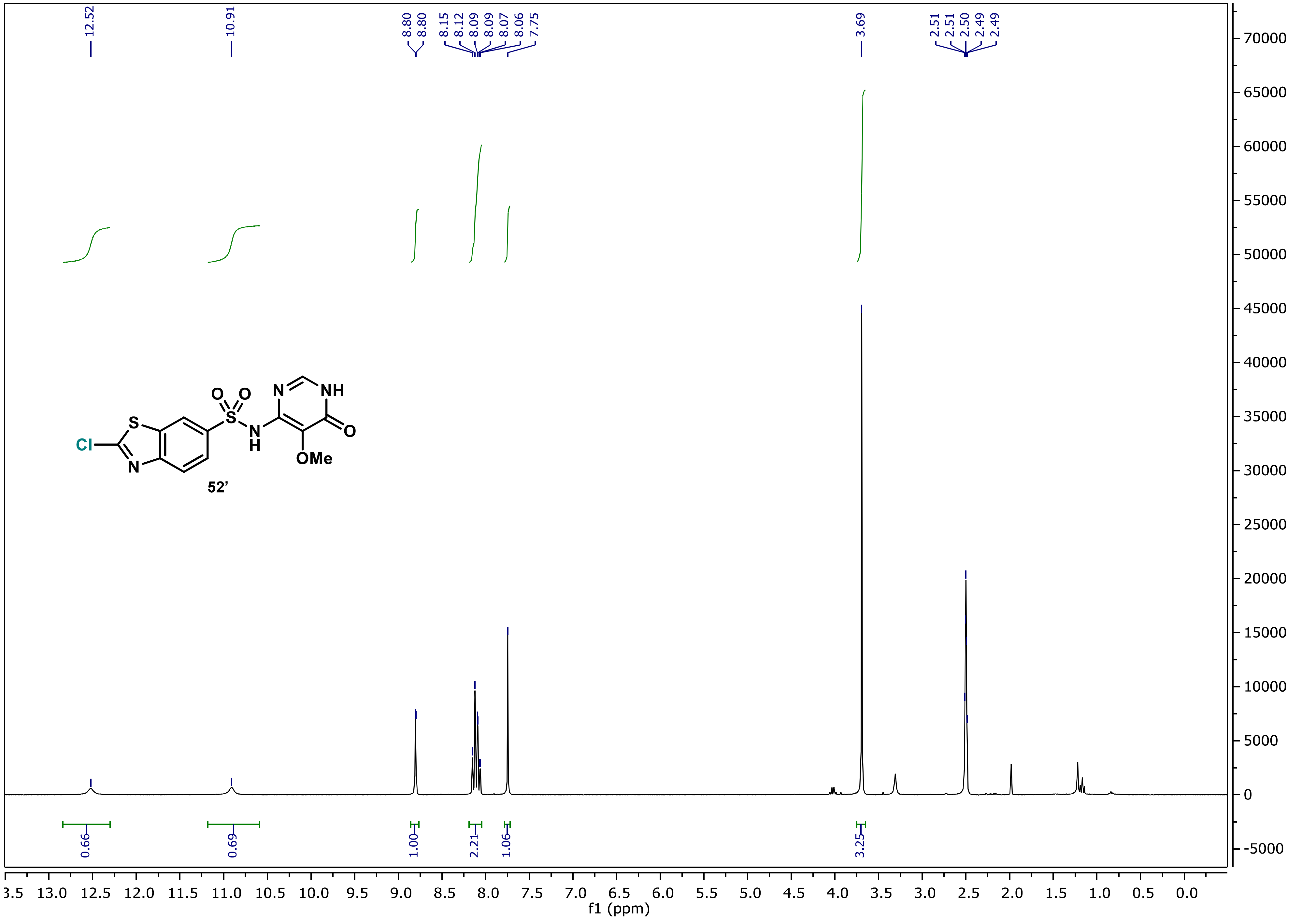
12.52
10.91
8.80
8.80
8.15
8.12
8.09
8.09
8.07
8.06
7.75
3.69
2.51
2.51
2.50
2.49
2.49
0.66
0.69
1.00
2.21
1.06
3.25
f1 (ppm)
Cl
S
N
O
O
S
N
H
N
NH
O
OMe

52’

^{13}C NMR

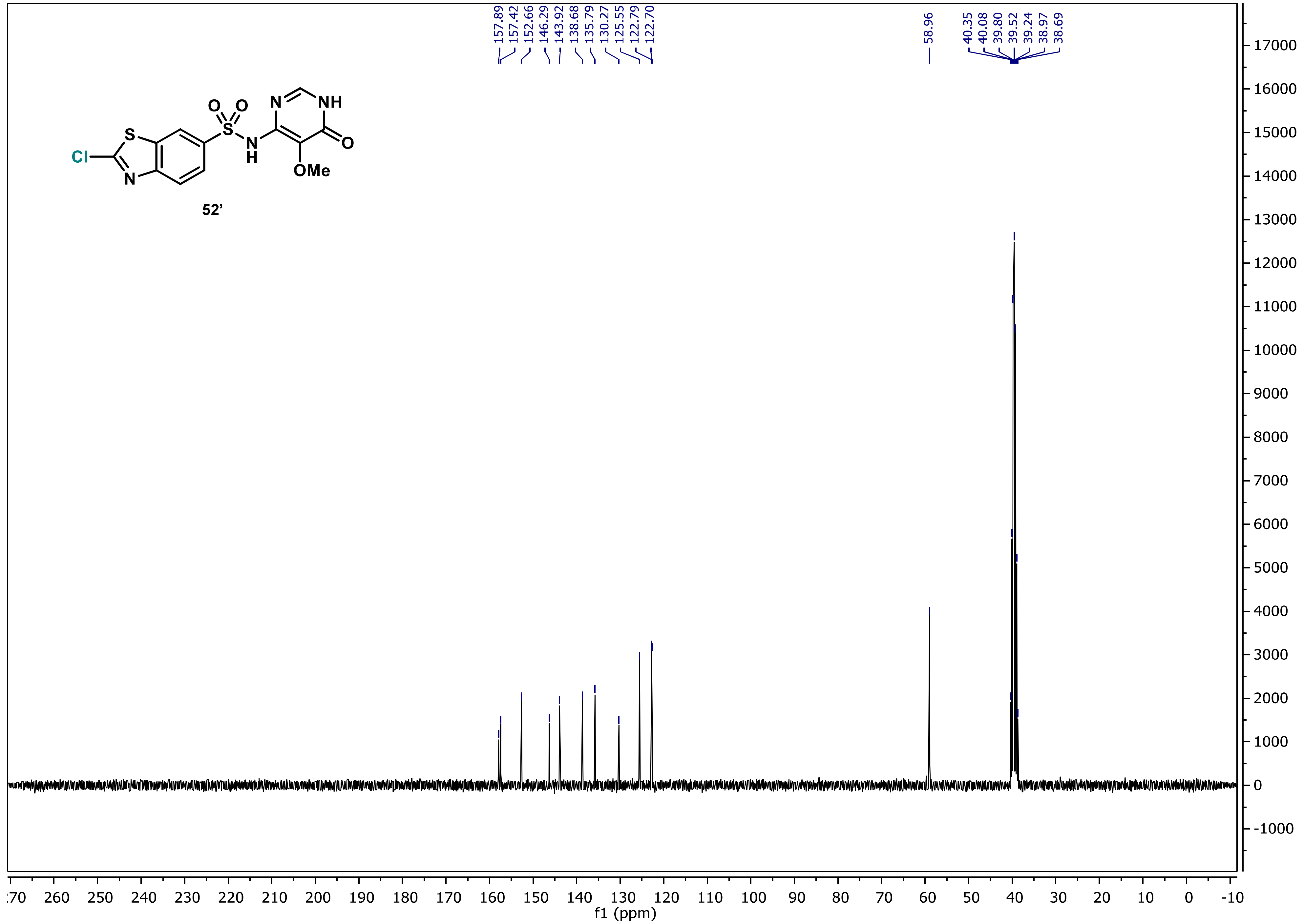
157.89
157.42
152.66
146.29
143.92
138.68
135.79
130.27
125.55
122.79
122.70
58.96
40.35
40.08
39.80
39.52
39.24
38.97
38.69
Cl
S
N
O
O
S
N
H
N
NH
O
OMe
52’
17000
16000
15000
14000
13000
12000
11000
10000
9000
8000
7000
6000
5000
4000
3000
2000
1000
0
-1000
260
250
240
230
220
210
200
190
180
170
160
150
140
130
120
110
100
90
80
70
60
50
40
30
20
10
0
-10
f1 (ppm)

Mass to be matched (m/z): 370.968360 Charge: -1

Mass Tolerance: ±0.005550
Restriction of atom numbers:

C	H	Cl	N	O	S
1-100	1-100	1-1	2-4	max 10	2-2

Number of calculated Formulas: 3

Formula	Diff.(ppm)	theor. m/z
C12 H8 Cl1 N4 O4 S2	-0.69	370.968103
C9 H10 Cl1 N3 O7 S2	-7.91	370.965424
C17 H8 Cl1 N2 O2 S2	10.15	370.972126

Datum	30.03.2021
Analyse:	152574b-00
Sigel:	GHC-GA-660-01
COP: Dr. Clement Ghiazza	
Messung:	HRMS
Methode:	ESIneg
Lösungsmittel:	CH3OH
Spektrometer:	Exactive
Auswerter:	Kampen (2242)

Suggestion:
C12H9Cl1N4O4S2 MW 372

characteristical ion
371 = [372 - H]-

^{1}H NMR

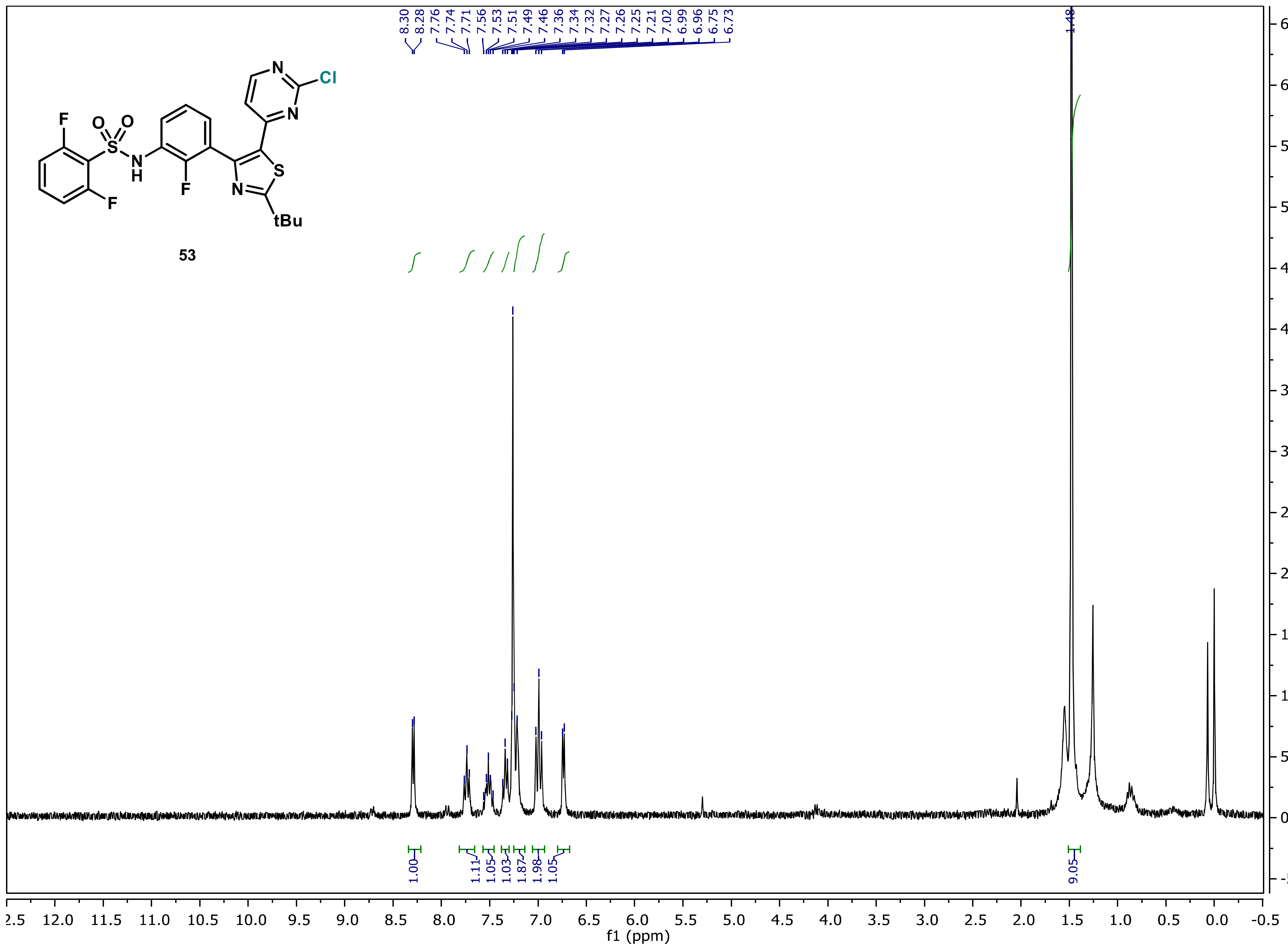
8.30
8.28
7.76
7.74
7.71
7.56
7.53
7.51
7.49
7.46
7.36
7.34
7.32
7.27
7.26
7.25
7.21
7.02
6.99
6.96
6.75
6.73
1.48
1.00
1.11
1.05
1.03
1.87
1.98
1.05
9.05
f1 (ppm)

53

^{13}C NMR

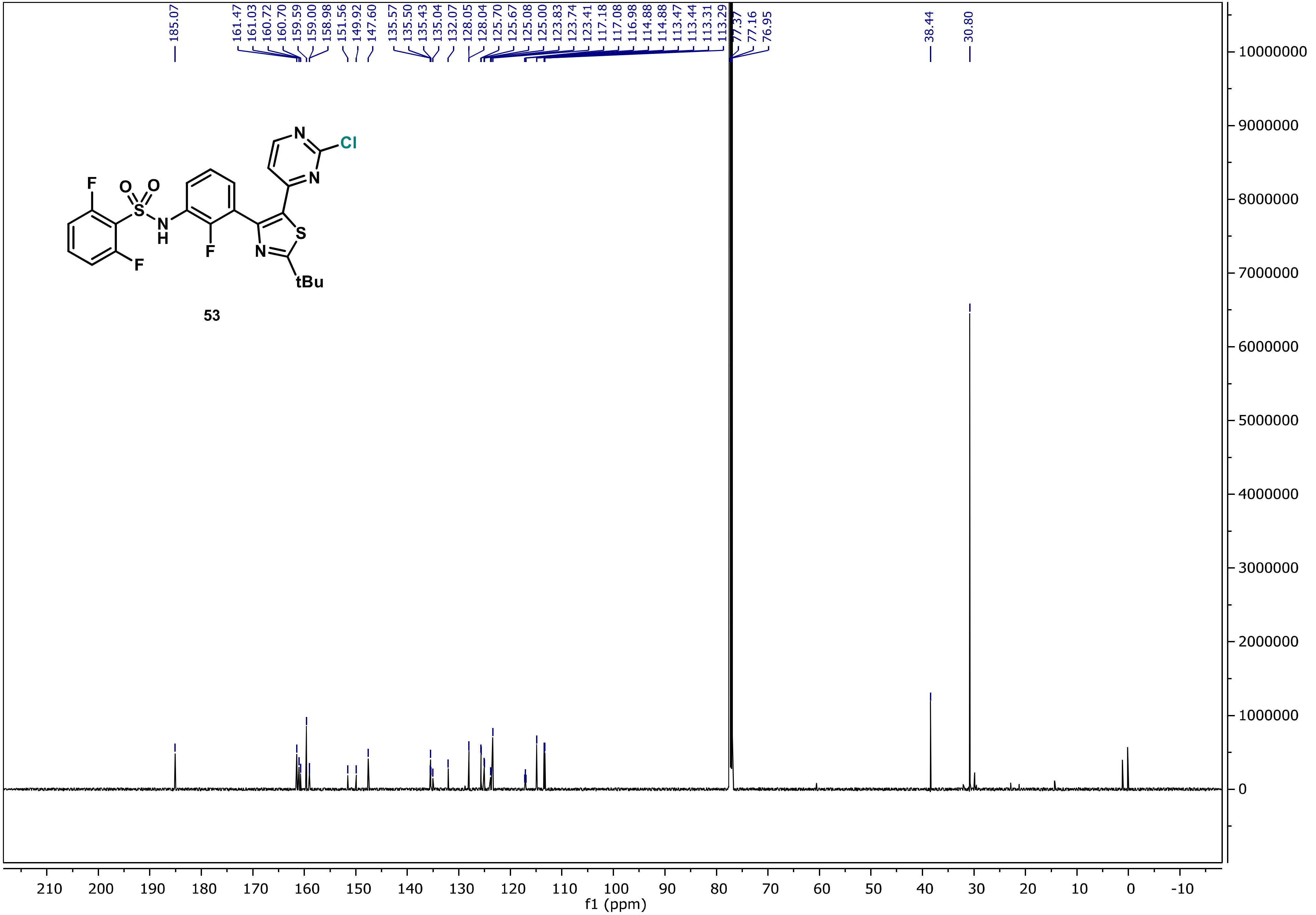

185.07
161.47
161.03
160.72
160.70
159.59
159.00
158.98
151.56
149.92
147.60
135.57
135.50
135.43
135.04
132.07
128.05
128.04
125.70
125.67
125.08
125.00
123.83
123.74
123.41
117.18
117.08
116.98
114.88
114.88
113.47
113.44
113.31
113.29
77.37
77.16
76.95
38.44
30.80
Cl
N
N
S
tBu
F
O
O
S
N
H
F
F
10000000
9000000
8000000
7000000
6000000
5000000
4000000
3000000
2000000
1000000
0
210
200
190
180
170
160
150
140
130
120
110
100
90
80
70
60
50
40
30
20
10
0
-10
f1 (ppm)

53

^{19}F NMR

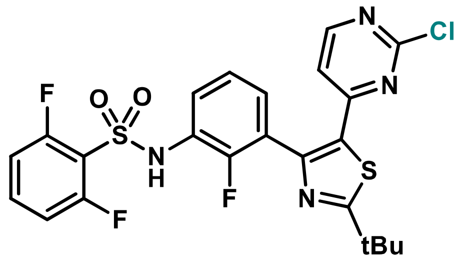

53

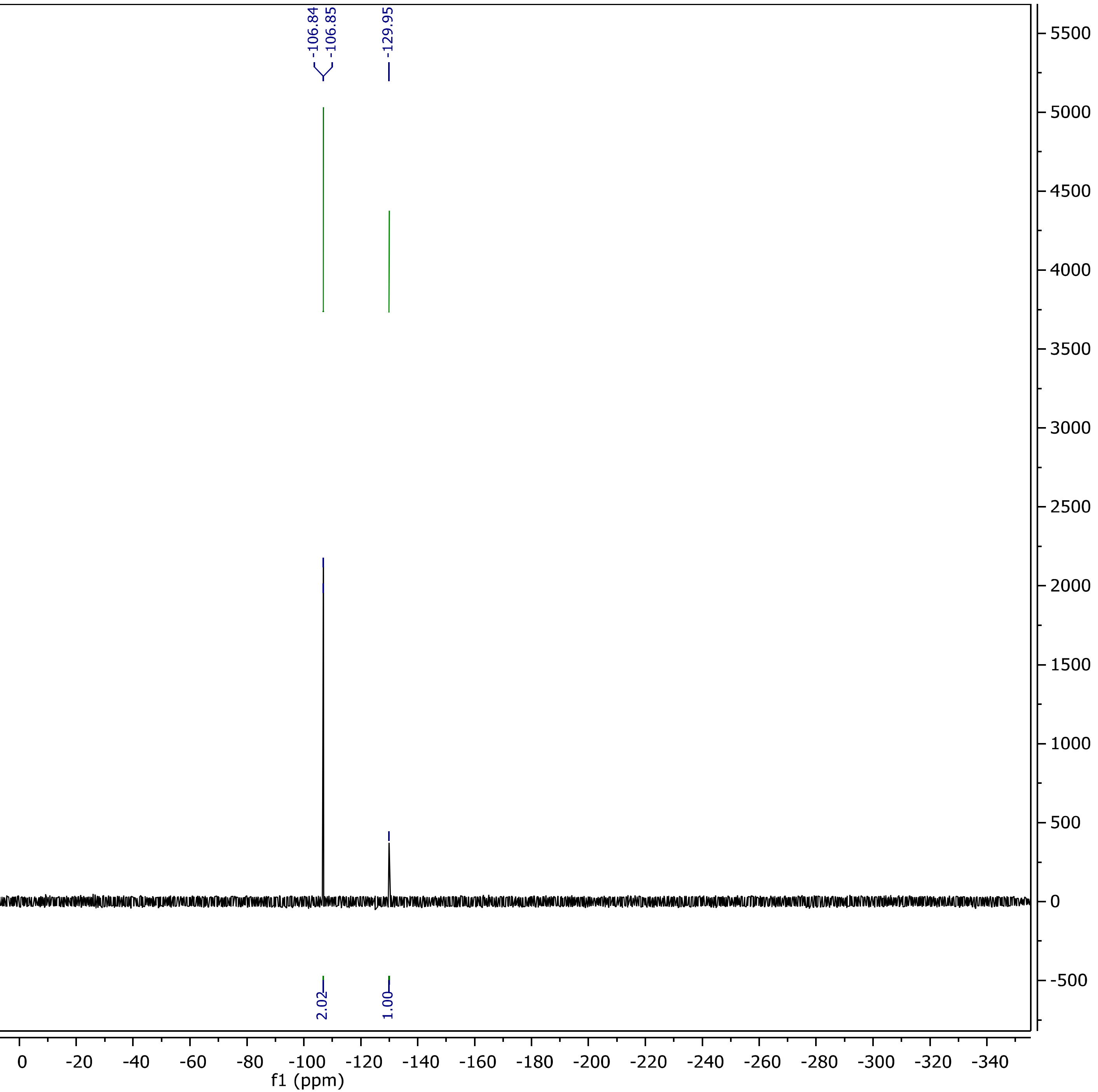
-106.84
-106.85
-129.95
2.02
1.00
5500
5000
4500
4000
3500
3000
2500
2000
1500
1000
500
0
-500
140 120 100 80 60 40 20 0 -20 -40 -60 -80 -100 -120 -140 -160 -180 -200 -220 -240 -260 -280 -300 -320 -340
f1 (ppm)

Mass to be matched (m/z): 539.058050 Charge: 1

Mass Tolerance: ±0.005000
Restriction of atom numbers:

C	H	Cl	F	N	O	S
1-100	1-100	1-1	3-3	2-4	max 5	2-2

Number of calculated Formulas: 3

Formula	Diff.(ppm)	theor. m/z
C23 H19 Cl1 F3 N4 O2 S2	0.76	539.058459
C20 H21 Cl1 F3 N3 O5 S2	-4.21	539.055780
C28 H19 Cl1 F3 N2 S2	8.22	539.062481

Datum	4.03.2021
Analyse:	152063b-00
Sigel:	GHC-GA-629-01
COP: Dr. Clement Ghiazza	
Messung:	HRMS
Methode:	ESIpos
Lösungsmittel:	CH2Cl2+CH3OH
Spektrometer:	Exactive
Auswerter:	Kampen (2242)

Suggestion:
C23H18Cl1F3N4O2S2 MW 538

characteristical ion
539 = [538 + H]+

^{1}H NMR

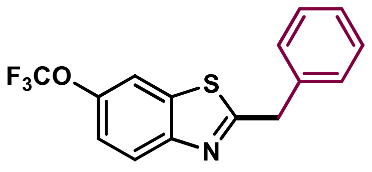

F3CO
S
N

54

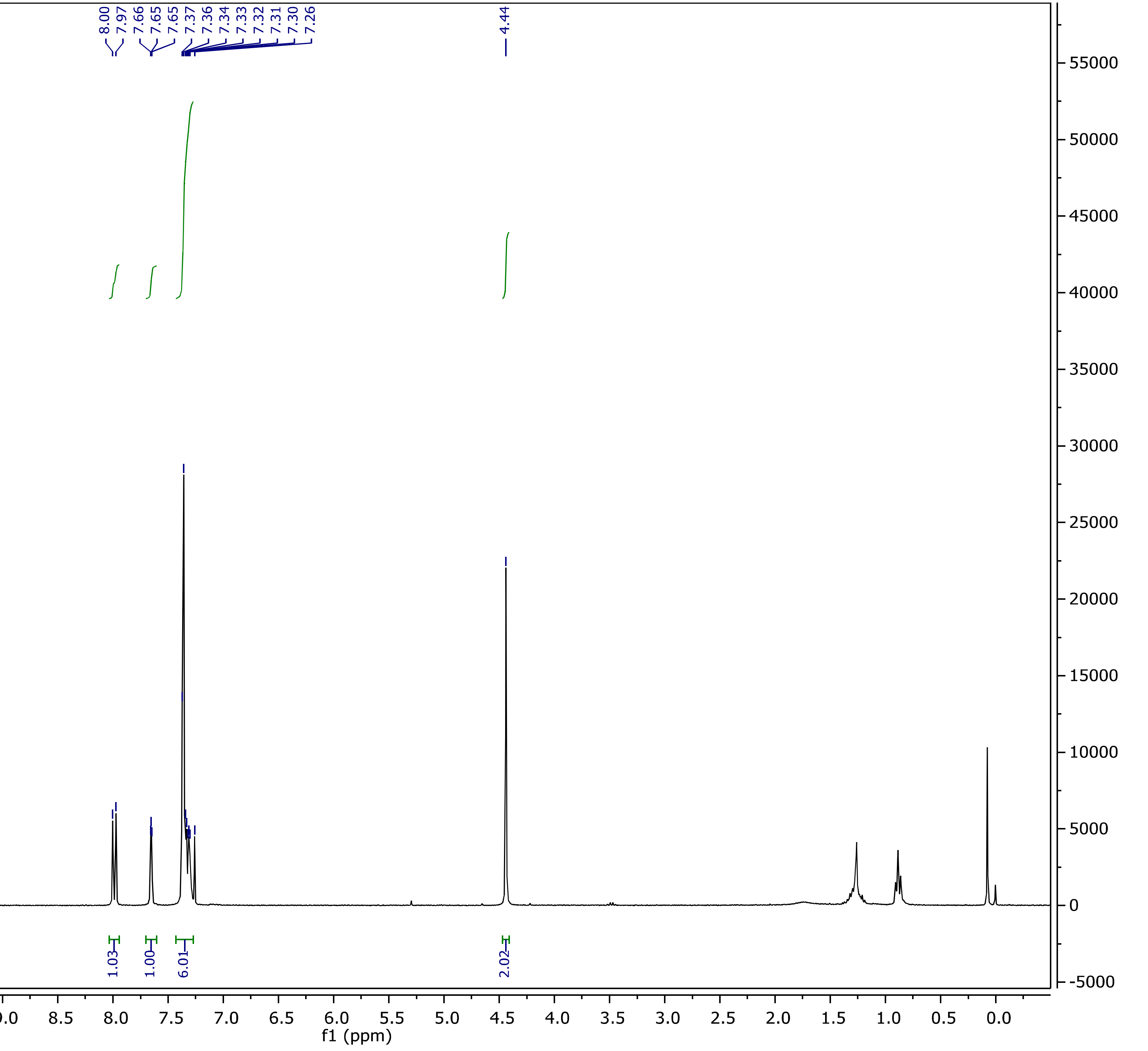

8.00
7.97
7.66
7.65
7.65
7.37
7.36
7.34
7.33
7.32
7.31
7.30
7.26
4.44
1.03
1.00
6.01
2.02
55000
50000
45000
40000
35000
30000
25000
20000
15000
10000
5000
0
-5000
12.0
11.5
11.0
10.5
10.0
9.5
9.0
8.5
8.0
7.5
7.0
6.5
6.0
5.5
5.0
4.5
4.0
3.5
3.0
2.5
2.0
1.5
1.0
0.5
0.0
f1 (ppm)

^{13}C NMR

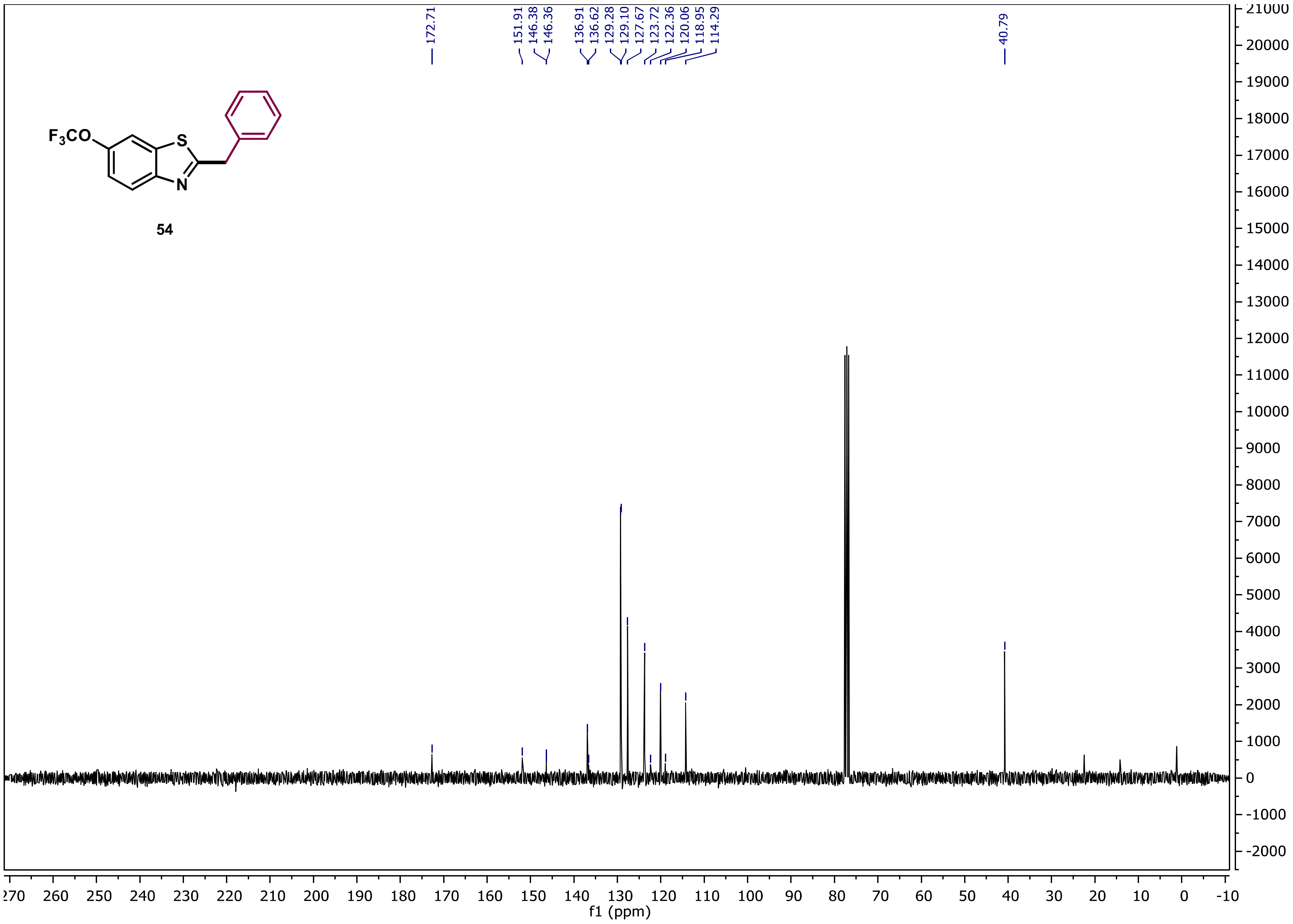

F3CO
S
N
172.71
151.91
146.38
146.36
136.91
136.62
129.28
129.10
127.67
123.72
122.36
120.06
118.95
114.29
40.79
f1 (ppm)

54

^{19}F NMR

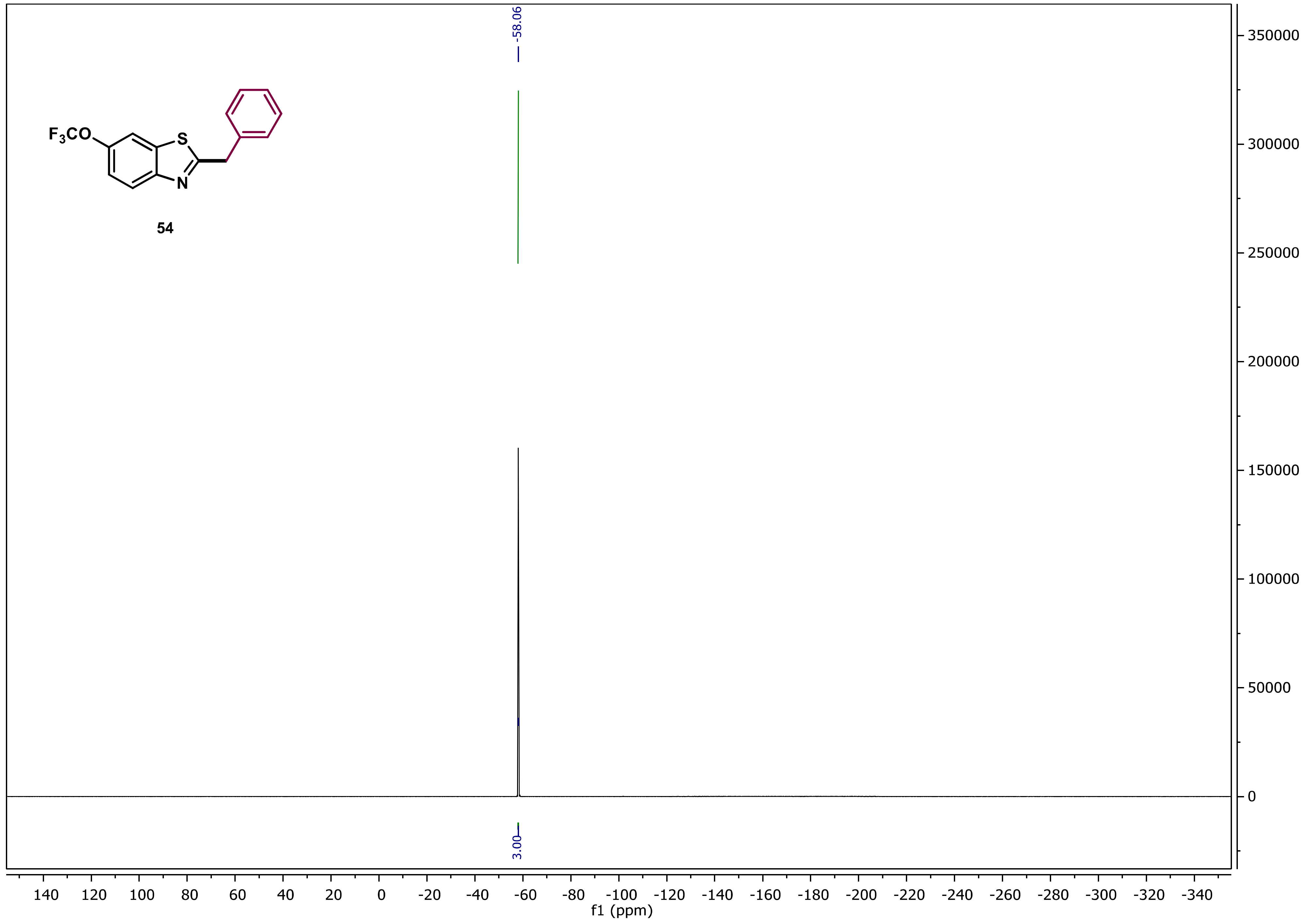

No.	MW.	Comment
1	309	Your proposed structure is possible<br>Ref.-Spektr. Nr. OU1617:

6.11.2020
File: 150300a-00.raw
Analyse: GHC-GA-426-01
COP: Dr. Clement Ghiazza

Messung:	GC-MS
Ionisierung:	GC-EI
Spektrometer:	QExactiveGC
Säule:	MS 50 RTX1+VS
Länge:	30+7
Temp.:	35-10-285-5
GC-Nr.:	-
MS-Nr.:	28258
Auswerter:	Margold (2242)

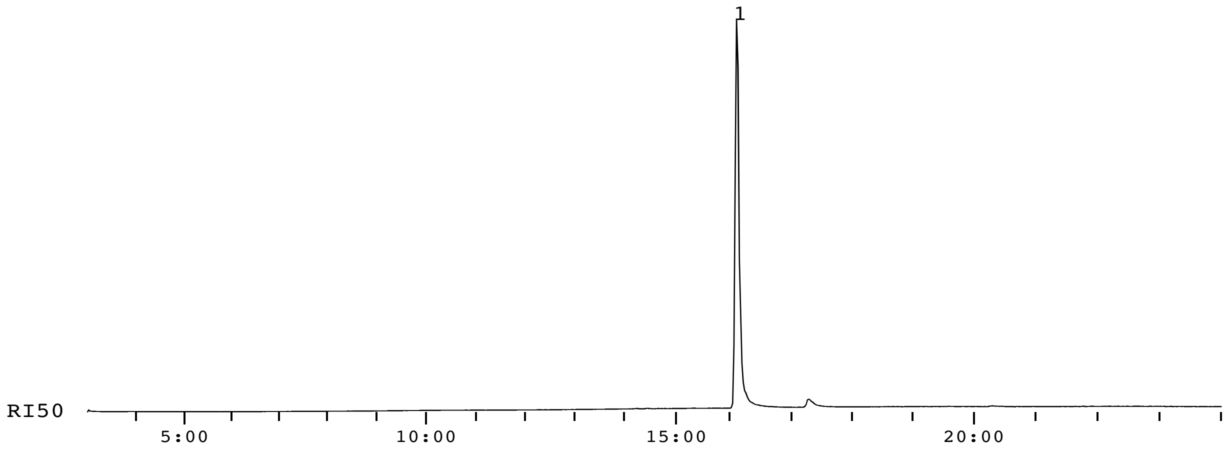

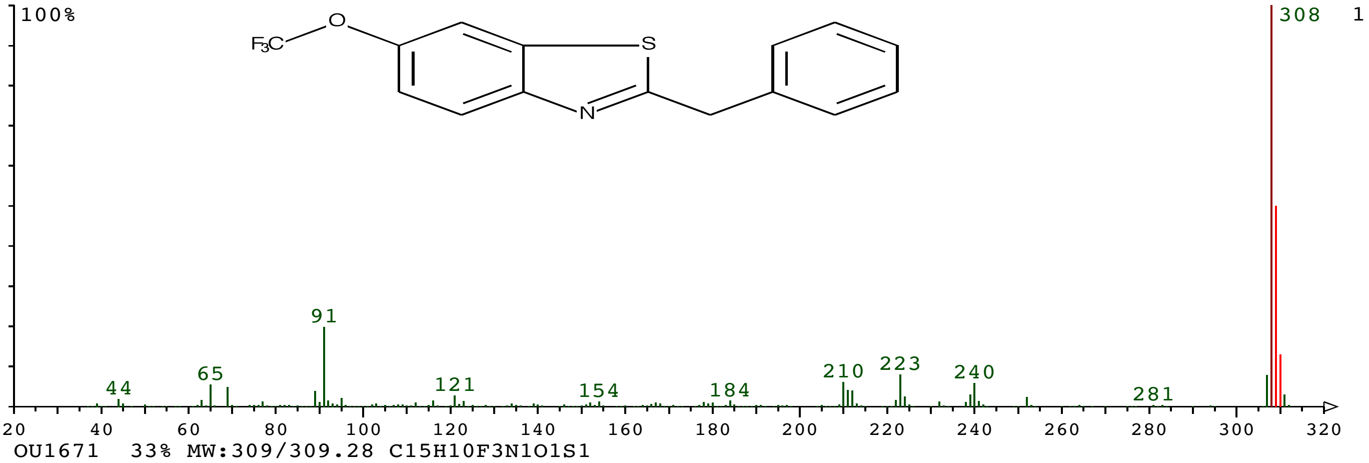

OU1671 33% MW:309/309.28 C15H10F3N1O1S1

39	0.76	80	0.14	106	0.13	134	0.80	169	0.08	206	0.10	241	1.30
41	0.09	81	0.37	107	0.31	135	0.30	171	0.25	207	0.07	242	0.56
44	1.86	82	0.27	108	0.50	136	0.20	173	0.07	208	0.16	249	0.15
45	0.77	83	0.27	109	0.41	139	0.70	177	0.36	209	0.56	252	2.32
50	0.45	84	0.11	110	0.19	140	0.41	178	1.07	210	6.15	253	0.30
51	0.15	85	0.19	111	0.23	141	0.21	179	0.67	211	4.12	254	0.09
57	0.07	87	0.10	112	0.96	146	0.49	180	0.94	212	3.97	264	0.34
62	0.38	88	0.13	113	0.10	148	0.15	181	0.13	213	0.72	275	0.07
63	1.55	89	3.81	114	0.12	150	0.21	183	0.31	214	0.20	276	0.09
64	0.17	90	1.08	115	0.32	151	0.43	184	1.38	221	0.15	277	0.15
65	5.48	91	19.83	116	1.48	152	1.03	185	0.47	222	1.52	281	0.38
66	0.23	92	1.41	117	0.19	153	0.40	186	0.15	223	7.98	282	0.13
69	4.82	93	0.78	120	0.21	154	1.21	187	0.07	224	2.44	283	0.32
70	0.29	94	0.41	121	2.75	155	0.23	188	0.15	225	0.46	294	0.17
71	0.08	95	2.00	122	0.62	160	0.17	190	0.36	226	0.07	306	0.08
74	0.25	96	0.30	123	1.33	162	0.11	191	0.25	230	0.16	307	7.84
75	0.25	97	0.07	125	0.27	164	0.35	192	0.09	232	1.20	308	100.00
76	0.36	102	0.44	126	0.14	165	0.21	195	0.25	233	0.20	309	50.05
77	1.23	103	0.71	127	0.16	166	0.60	196	0.17	238	1.05	310	12.97
78	0.23	104	0.10	128	0.29	167	1.00	197	0.28	239	2.93	311	2.93
79	0.11	105	0.38	133	0.20	168	0.71	205	0.33	240	5.85	312	0.34

OU1671 33% MW:309/309.28 C15H10F3N1O1S1 lim: 0.07%

```
Mass to be matched (m/z): 309.043220  Charge: 1

Mass Tolerance: ±0.005000
Restriction of atom numbers:
C     H     N     O     S     F
1-110 1-100 1-1   1-3   1-1   1-3
Number of calculated Formulas:   1

Formula              Diff.(ppm)    theor. m/z
C15 H10 N1 O1 S1 F3       -0.80    309.042972
```

```
 6.11.2020
File: 150300a-00.raw
Analyse: GHC-GA-426-01
COP: Dr. Clement Ghiazza
```

Messung:	GC-MS
Ionisierung:	GC-EI
Spektrometer:	QExactiveGC
Säule:	MS 50 RTX1+VS
Länge:	30+7
Temp.:	35-10-285-5
GC-Nr.:	-
MS-Nr.:	28258
Auswerter:	Margold (2242)

^{1}H NMR

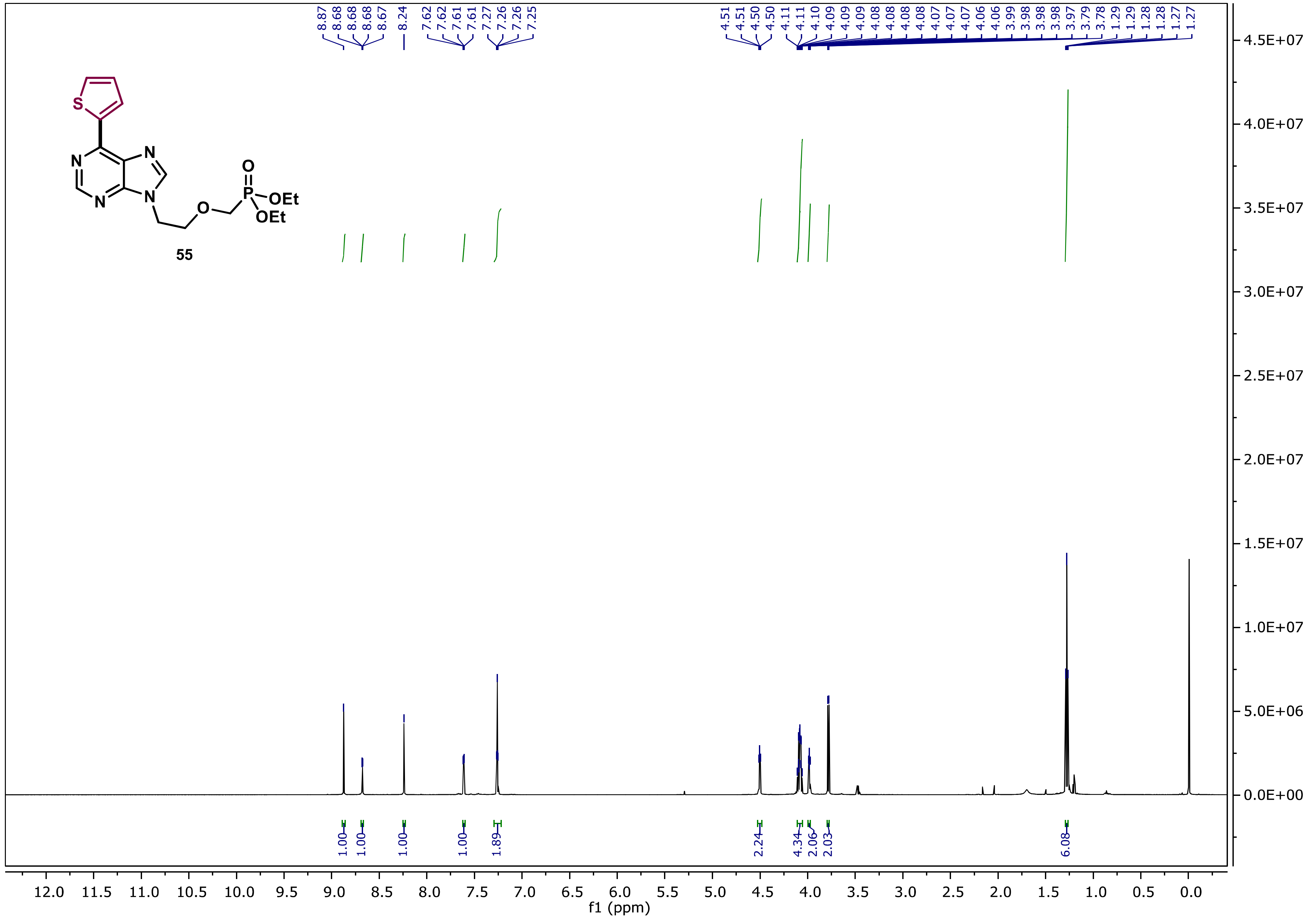

8.87
8.68
8.68
8.68
8.67
8.24
7.62
7.62
7.61
7.61
7.27
7.26
7.26
7.25
4.51
4.51
4.50
4.50
4.11
4.11
4.10
4.09
4.09
4.09
4.08
4.08
4.08
4.08
4.07
4.07
4.07
4.06
4.06
3.99
3.98
3.98
3.98
3.97
3.79
3.78
1.29
1.29
1.28
1.28
1.27
1.27
N
N
N
N
S
O
P
OEt
OEt
O
55
1.00
1.00
1.00
1.00
1.89
2.24
4.34
2.06
2.03
6.08
4.5E+07
4.0E+07
3.5E+07
3.0E+07
2.5E+07
2.0E+07
1.5E+07
1.0E+07
5.0E+06
0.0E+00
12.0
11.5
11.0
10.5
10.0
9.5
9.0
8.5
8.0
7.5
7.0
6.5
6.0
5.5
5.0
4.5
4.0
3.5
3.0
2.5
2.0
1.5
1.0
0.5
0.0
f1 (ppm)

^{13}C NMR

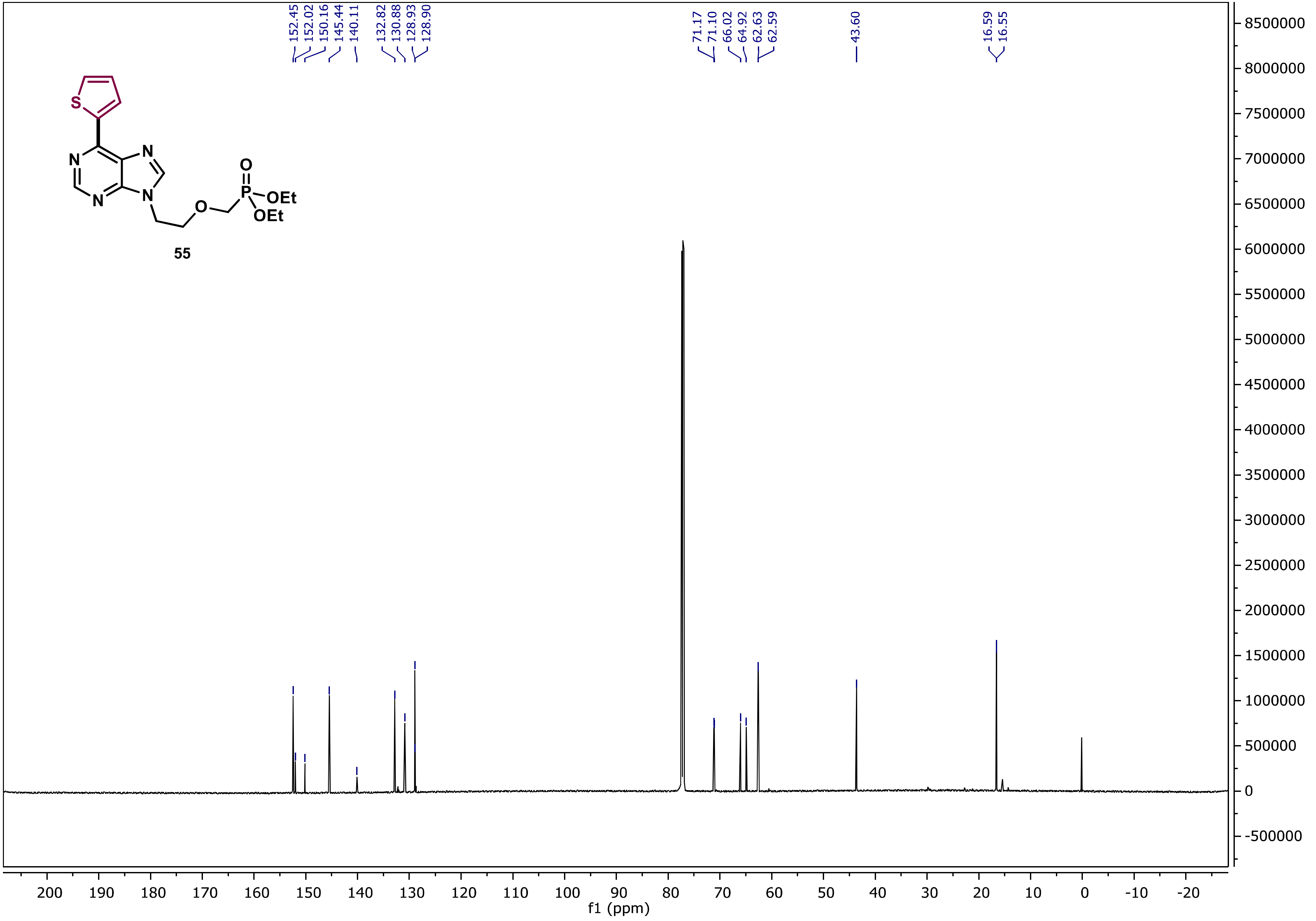

S
N
N
N
N
O
O
P
OEt
OEt
55
152.45
152.02
150.16
145.44
140.11
132.82
130.88
128.93
128.90
71.17
71.10
66.02
64.92
62.63
62.59
43.60
16.59
16.55
8500000
8000000
7500000
7000000
6500000
6000000
5500000
5000000
4500000
4000000
3500000
3000000
2500000
2000000
1500000
1000000
500000
0
-500000
200 190 180 170 160 150 140 130 120 110 100 90 80 70 60 50 40 30 20 10 0 -10 -20
f1 (ppm)

^{31}P NMR

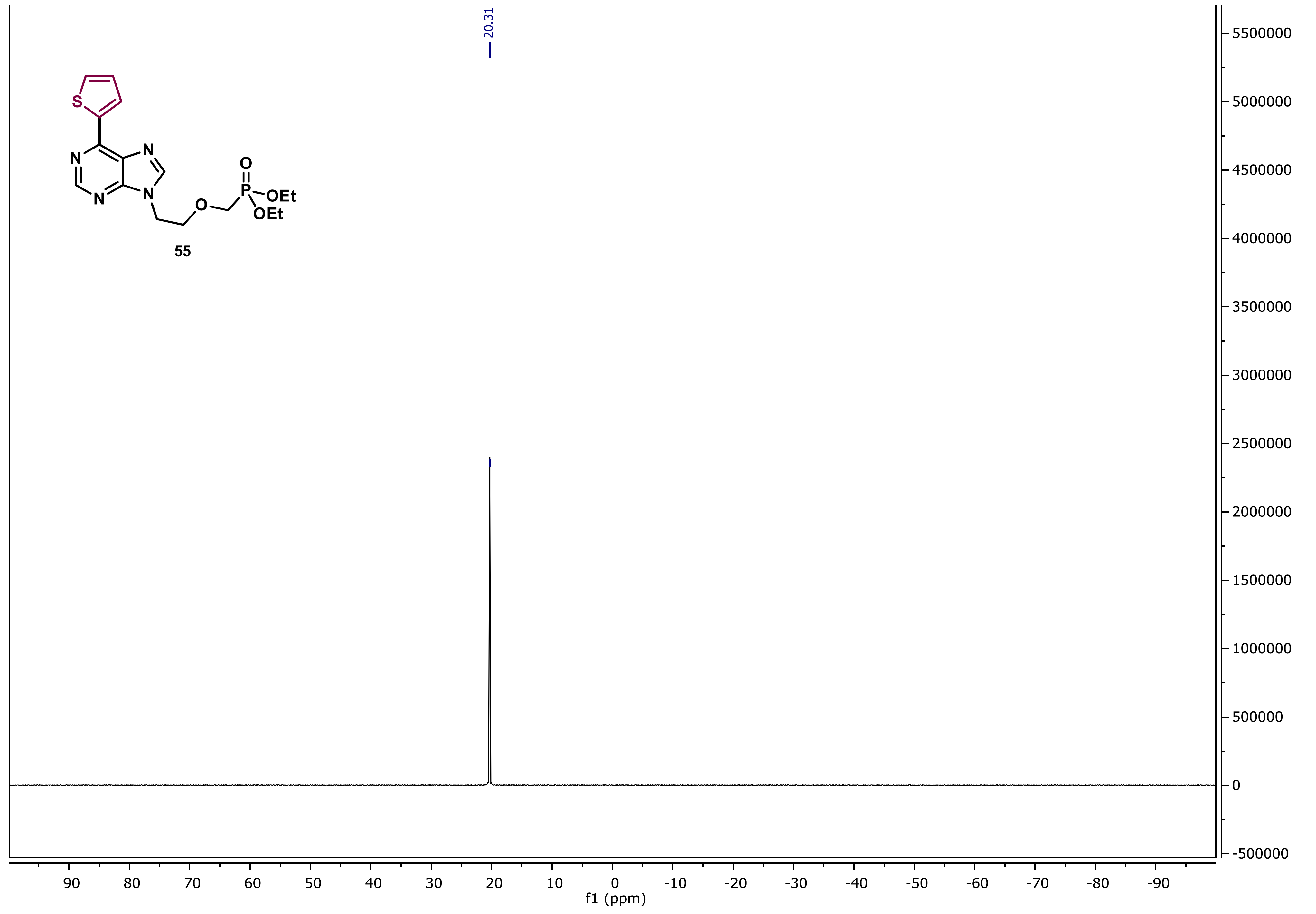

20.31
S
N
N
N
N
O
O
P
OEt
OEt
55
5500000
5000000
4500000
4000000
3500000
3000000
2500000
2000000
1500000
1000000
500000
0
-500000
90
80
70
60
50
40
30
20
10
0
-10
-20
-30
-40
-50
-60
-70
-80
-90
f1 (ppm)

Mass to be matched (m/z): 419.091270 Charge: 1

Mass Tolerance: ±0.005000
Restriction of atom numbers:

C	H	N	O	P	S	Na
1-100	1-100	2-4	max 10	1-1	1-1	1-1

Number of calculated Formulas: 3

Formula	Diff.(ppm)	theor. m/z
C16 H21 N4 O4 P1 S1 Na1	0.16	419.091336
C13 H23 N3 O7 P1 S1 Na1	-6.24	419.088657
C21 H21 N2 O2 P1 S1 Na1	9.76	419.095358

Datum	23.10.2020
Analyse:	150104b-00
Sigel:	GHC-GA-395-01
COP: Dr. Clement Ghiazza	
Messung:	HRMS
Methode:	ESIpos
Lösungsmittel:	CH2Cl2+CH3OH
Spektrometer:	Exactive
Auswerter:	Kampen (2242)

Suggestion:
C16H21N4O4P1S1 MW 396

characteristical ion
419 = [396 + Na]+

^{1}H NMR

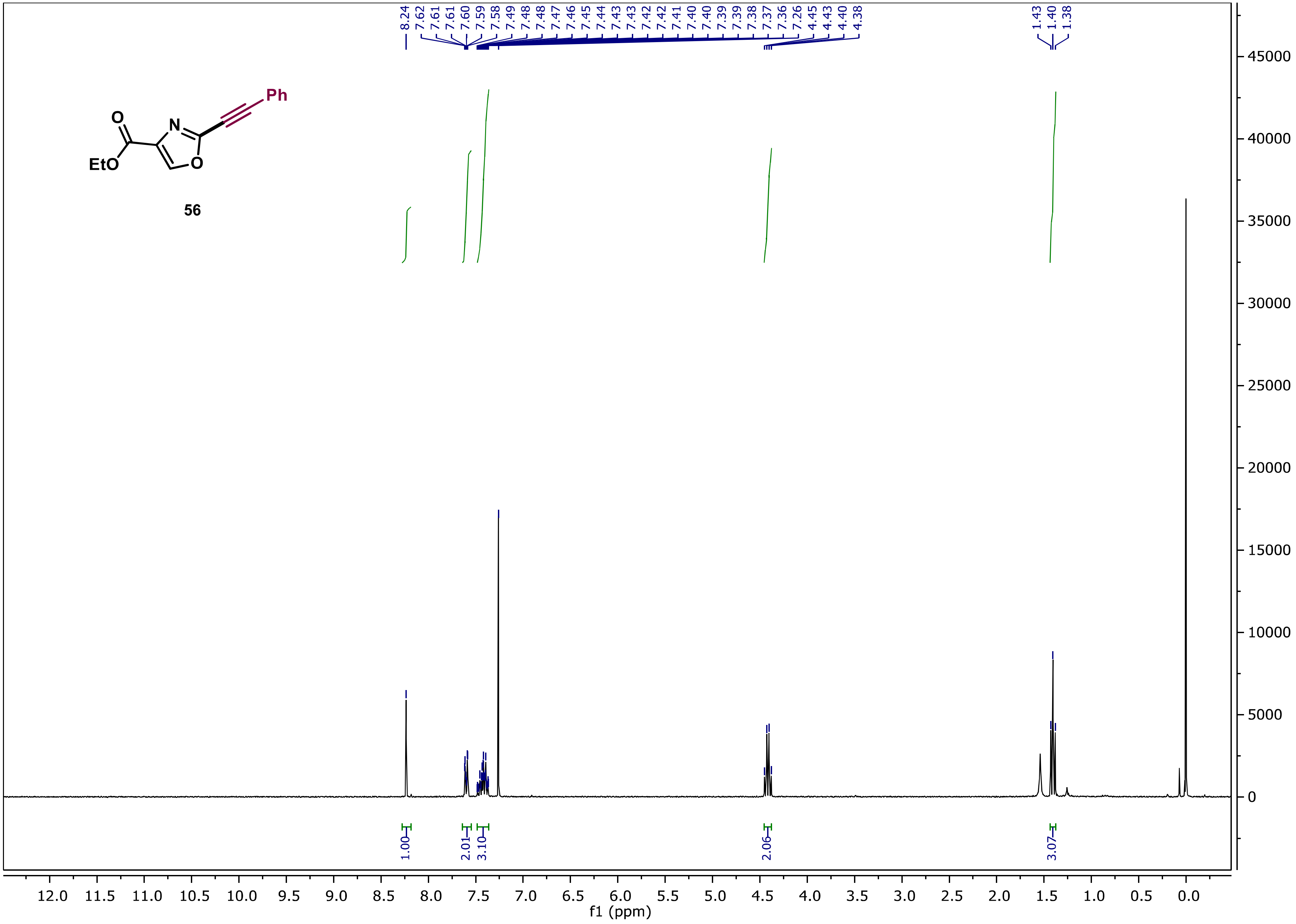
EtO
O
N
O
Ph
56
8.24
7.62
7.61
7.61
7.60
7.59
7.58
7.49
7.48
7.48
7.47
7.46
7.45
7.44
7.43
7.43
7.42
7.42
7.41
7.40
7.40
7.39
7.39
7.38
7.37
7.36
7.26
4.45
4.43
4.40
4.38
1.43
1.40
1.38
1.00
2.01
3.10
2.06
3.07
f1 (ppm)

^{13}C NMR

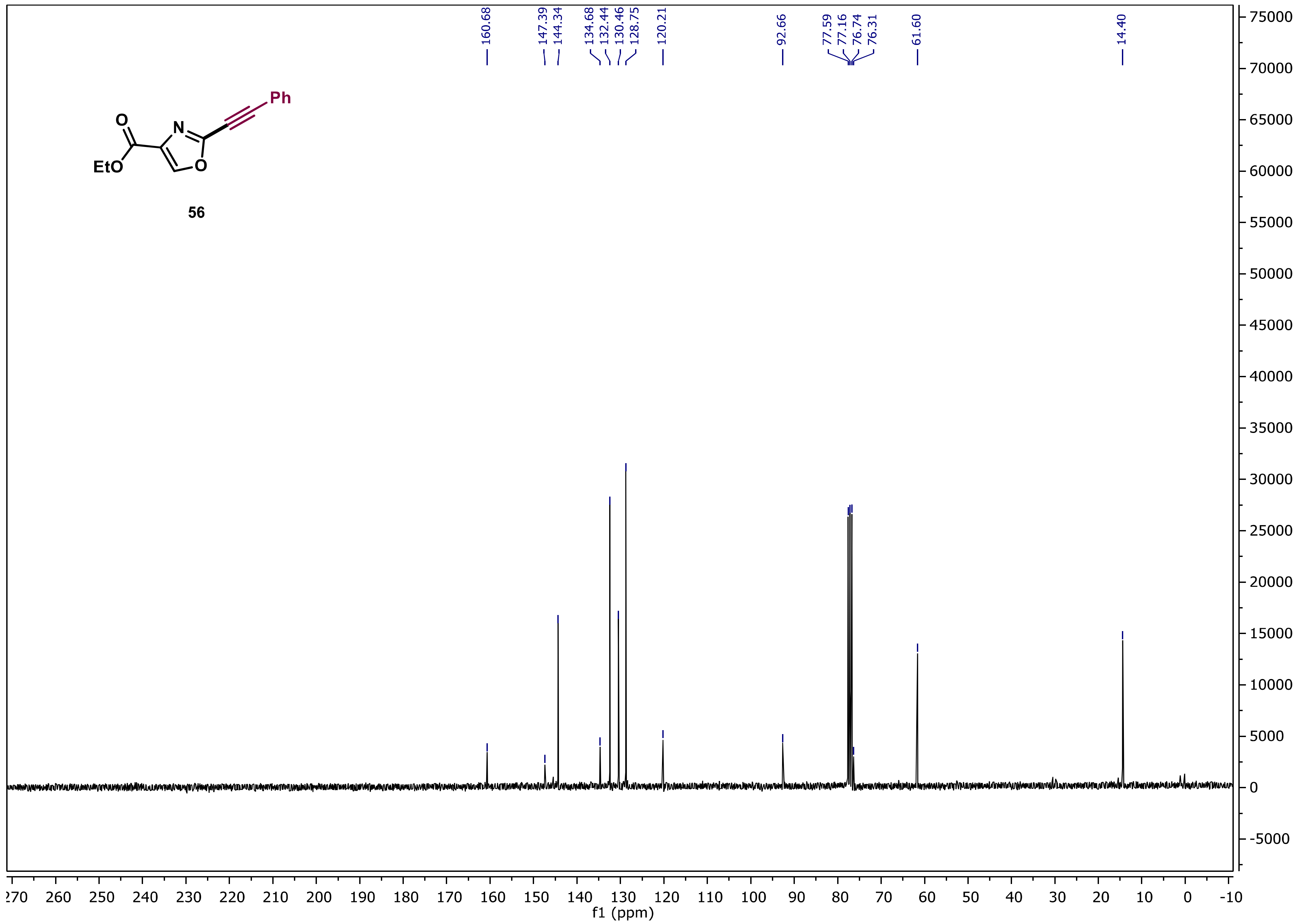

160.68
147.39
144.34
134.68
132.44
130.46
128.75
120.21
92.66
77.59
77.16
76.74
76.31
61.60
14.40
Ph
O
N
EtO
O
56
f1 (ppm)

Mass to be matched (m/z): 241.073490 Charge: 1

Mass Tolerance: ±0.050000
Restriction of atom numbers:

C	H	N	O
1-100	1-100	1-2	1-5

Number of calculated Formulas: 11

Formula	Diff.(ppm)	theor. m/z
C14 H11 N1 O3	-0.61	241.073344
C10 H13 N2 O5	34.88	241.081898
C13 H9 N2 O3	-52.77	241.060768
C11 H15 N1 O5	87.04	241.094474
C17 H7 N1 O1	-88.26	241.052214
C14 H13 N2 O2	98.15	241.097153
C16 H5 N2 O1	-140.42	241.039637
C15 H15 N1 O2	150.32	241.109729
C13 H7 N1 O4	-151.54	241.036959
C11 H17 N2 O4	185.80	241.118282
C12 H5 N2 O4	-203.70	241.024382

25.01.2021
File: 151335b-00
Analyse: GHC-GA-552-01
COP: Dr. Clement Ghiazza

Messung: (HRMS)
Ionisierung: EI
Spektrometer: Q Exactive GC Orbitrap
Säule: MS50 RTX-1+VS
Länge: 30+7
Temp.: 35-10-285-5
GC-Nr.:
ELNA-Nr.: 29288

Auswerter: Haupt (2243)

Suggestion:

C14H11N1O3 MW: 241

¹H NMR

57

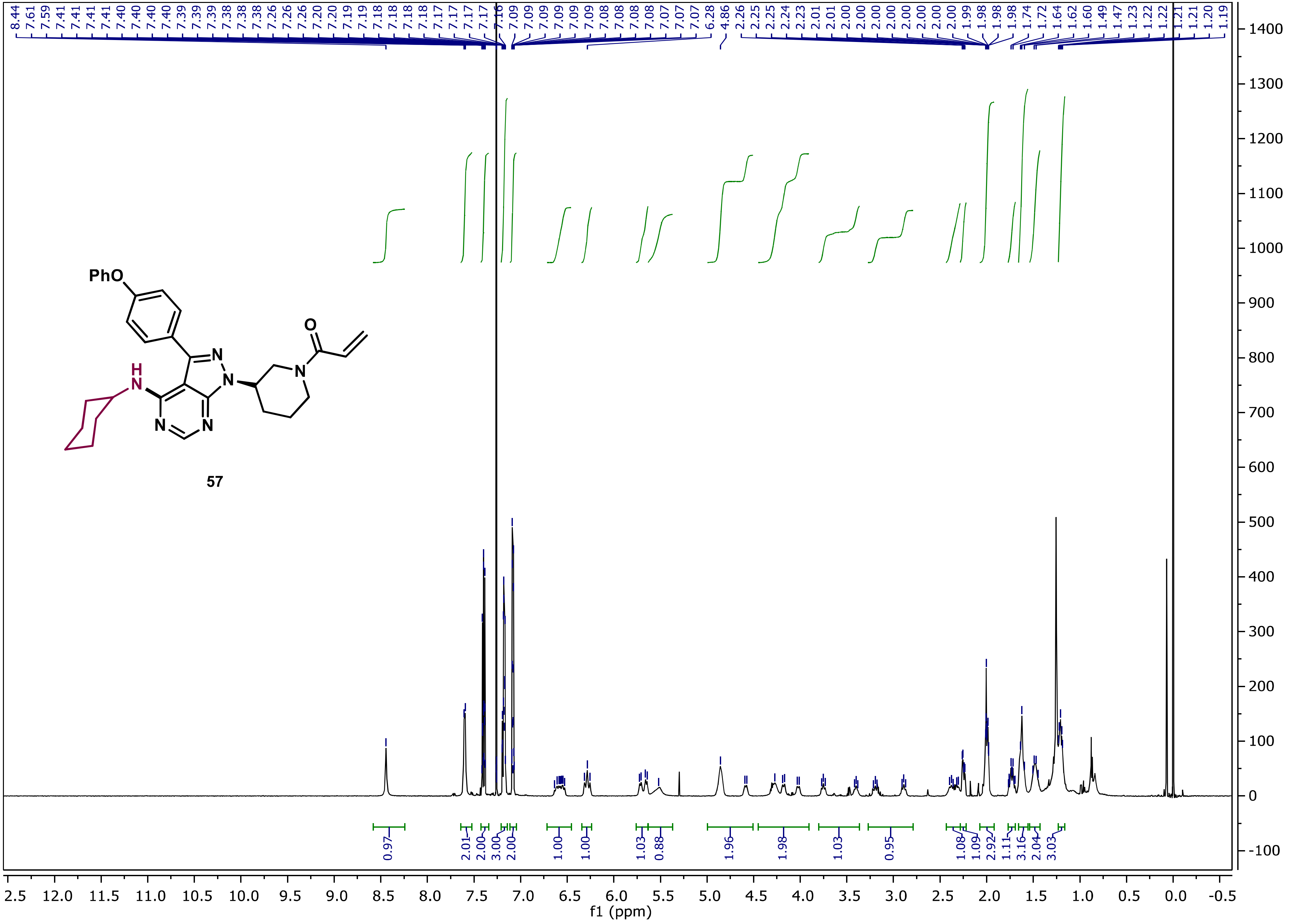
PhO
H
N
N
N
N
N
N
O
8.44
7.61
7.59
7.41
7.41
7.41
7.41
7.40
7.40
7.40
7.40
7.39
7.39
7.38
7.38
7.38
7.26
7.26
7.26
7.20
7.20
7.19
7.19
7.18
7.18
7.18
7.18
7.17
7.17
7.17
7.17
7.16
7.09
7.09
7.09
7.09
7.09
7.09
7.09
7.08
7.08
7.08
7.08
7.07
7.07
7.07
6.28
4.86
2.26
2.25
2.25
2.24
2.23
2.01
2.01
2.00
2.00
2.00
2.00
2.00
2.00
2.00
1.99
1.98
1.98
1.98
1.74
1.72
1.64
1.62
1.60
1.49
1.47
1.23
1.22
1.22
1.21
1.21
1.20
1.19
0.97
2.01
2.00
3.00
2.00
1.00
1.00
1.03
0.88
1.96
1.98
1.03
0.95
1.08
1.09
2.92
1.11
3.16
2.04
3.03
f1 (ppm)

^{13}C NMR

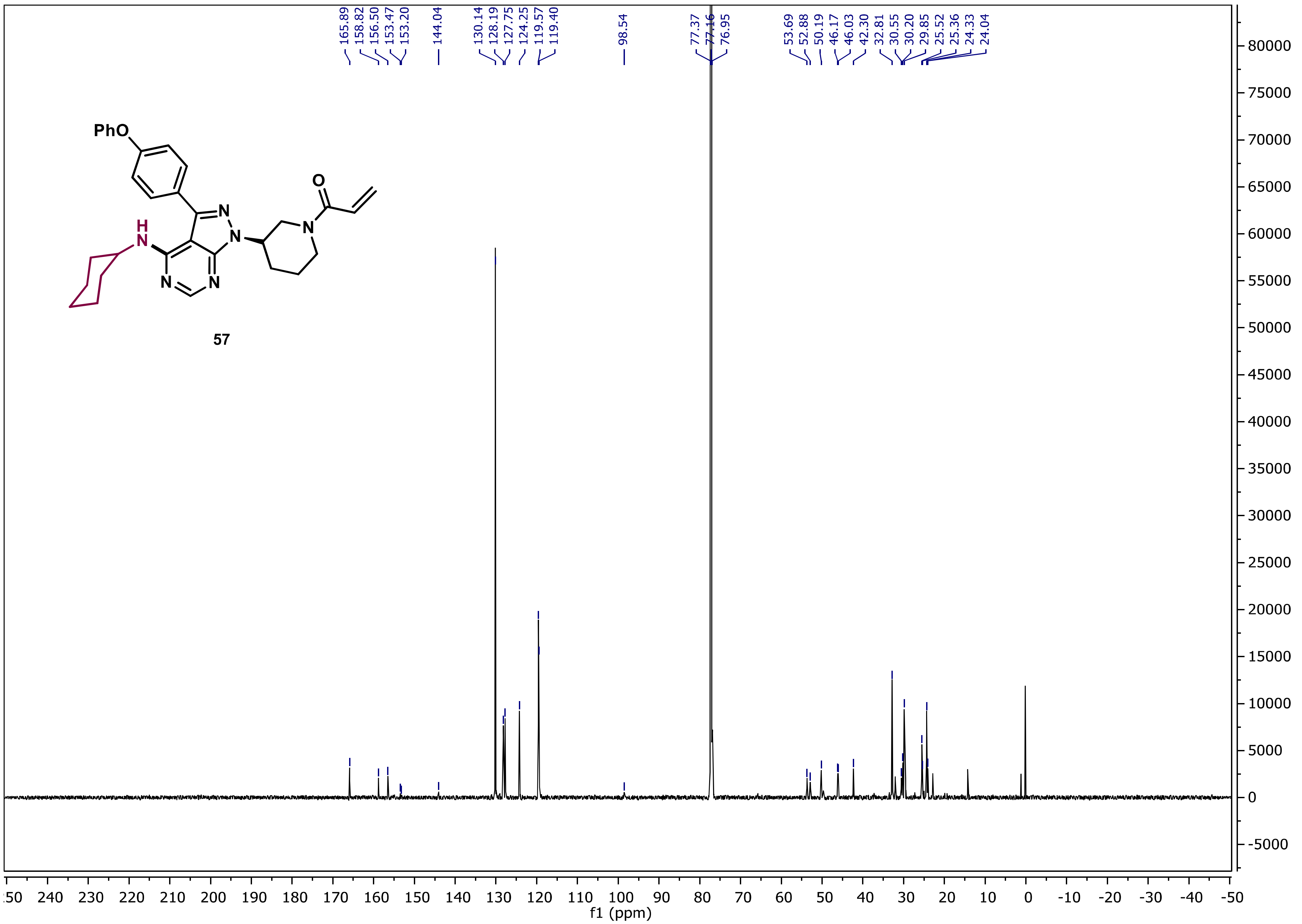
165.89
158.82
156.50
153.47
153.20
144.04
130.14
128.19
127.75
124.25
119.57
119.40
98.54
77.37
77.16
76.95
53.69
52.88
50.19
46.17
46.03
42.30
32.81
30.55
30.20
29.85
25.52
25.36
24.33
24.04
PhO
O
N
H
57
f1 (ppm)

Mass to be matched (m/z): 523.281660 Charge: 1

Mass Tolerance: ±0.005000
Restriction of atom numbers:

C	H	N	O
1-100	1-100	4-6	max 10

Number of calculated Formulas: 4

Formula	Diff.(ppm)	theor. m/z
C31 H35 N6 O2	-0.12	523.281597
C28 H37 N5 O5	-5.24	523.278919
C21 H41 N5 O10	5.99	523.284793
C36 H35 N4	7.57	523.285620

Datum	19.11.2020
Analyse:	150555c-00
Sigel:	GHC-GA-453-01
COP: Dr. Clement Ghiazza	
Messung:	HRMS
Methode:	ESIpos
Lösungsmittel:	CH2Cl2+CH3OH
Spektrometer:	Exactive
Auswerter:	Kampen (2242)

Suggestion:
C31H34N6O2 MW 522

characteristical ion
523 = [522 + H]+

¹H NMR

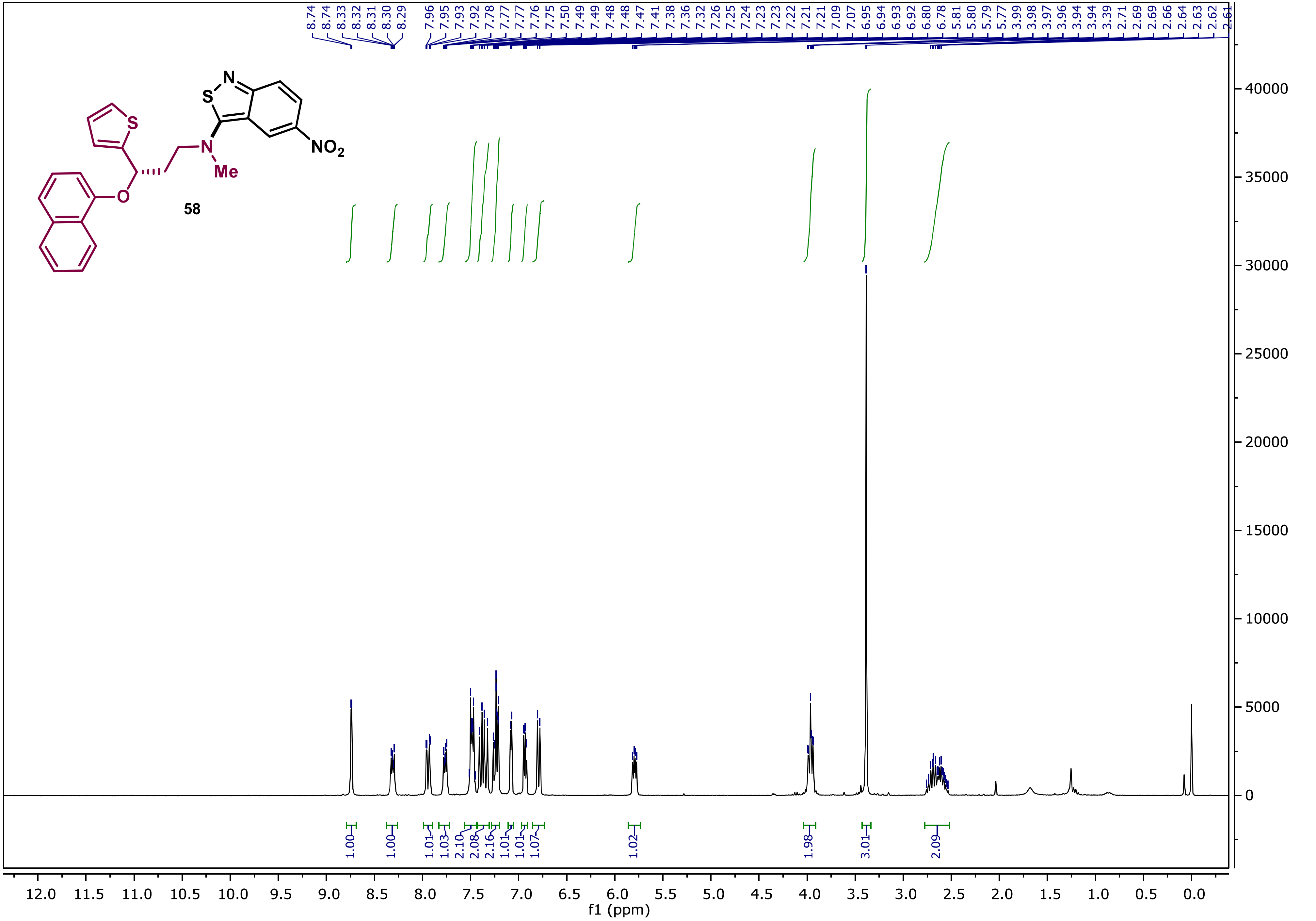

NO2
Me
N
S
O
8.74 8.74 8.33 8.32 8.31 8.30 8.29 7.96 7.95 7.93 7.92 7.78 7.77 7.77 7.76 7.75 7.50 7.49 7.49 7.48 7.48 7.47 7.41 7.38 7.36 7.32 7.26 7.25 7.24 7.23 7.23 7.22 7.21 7.21 7.09 7.07 6.95 6.94 6.93 6.92 6.80 6.78 5.81 5.80 5.79 5.77 3.99 3.98 3.97 3.96 3.94 3.94 3.39 2.71 2.69 2.69 2.66 2.64 2.63 2.62 2.61
1.00 1.00 1.01 1.03 2.10 2.08 2.16 1.01 1.01 1.07 1.02 1.98 3.01 2.09
f1 (ppm)

58

^{13}C NMR

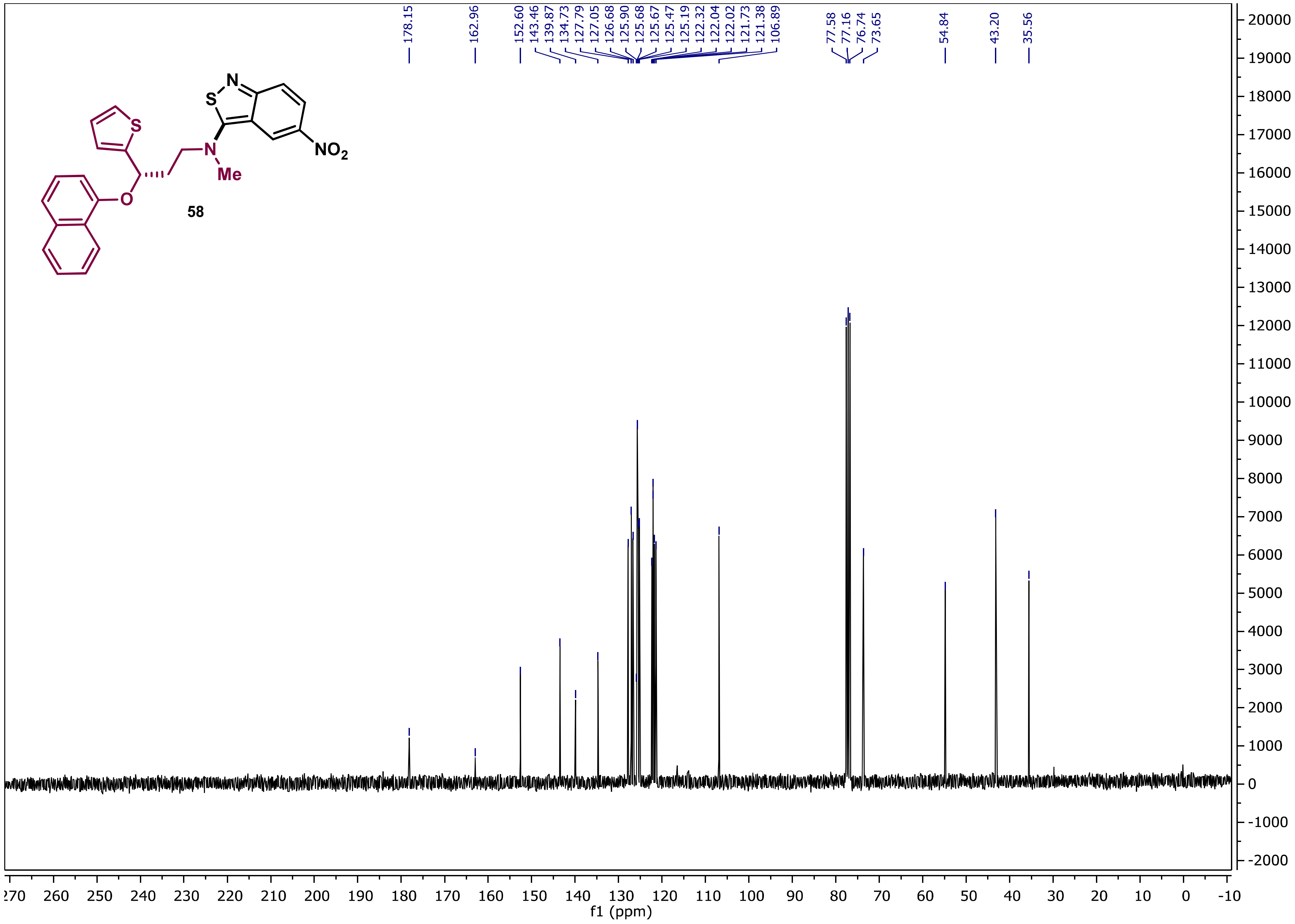

178.15
162.96
152.60
143.46
139.87
134.73
127.79
127.05
126.68
125.90
125.68
125.67
125.47
125.19
122.32
122.04
122.02
121.73
121.38
106.89
77.58
77.16
76.74
73.65
54.84
43.20
35.56
S
N
NO2
O
Me
f1 (ppm)

58

Mass to be matched (m/z): 476.109480 Charge: 1

Mass Tolerance: ±0.005000
Restriction of atom numbers:

C	H	N	O	S
1-100	1-100	1-3	max 5	2-2

Number of calculated Formulas: 2

Formula	Diff.(ppm)	theor. m/z
C25 H22 N3 O3 S2	0.49	476.109711
C30 H22 N1 O1 S2	8.93	476.113734

Datum	3.11.2020
Analyse:	150251b-00
Sigel:	GHC-GA-417-01
COP: Dr. Clement Ghiazza	
Messung:	HRMS
Methode:	ESIpos
Lösungsmittel:	CH2Cl2+CH3OH
Spektrometer:	Exactive
Auswerter:	Kampen (2242)

Suggestion:
C25H22N3O3S2 MW 475

characteristical ion
476 = [475 + H]+

^{1}H NMR

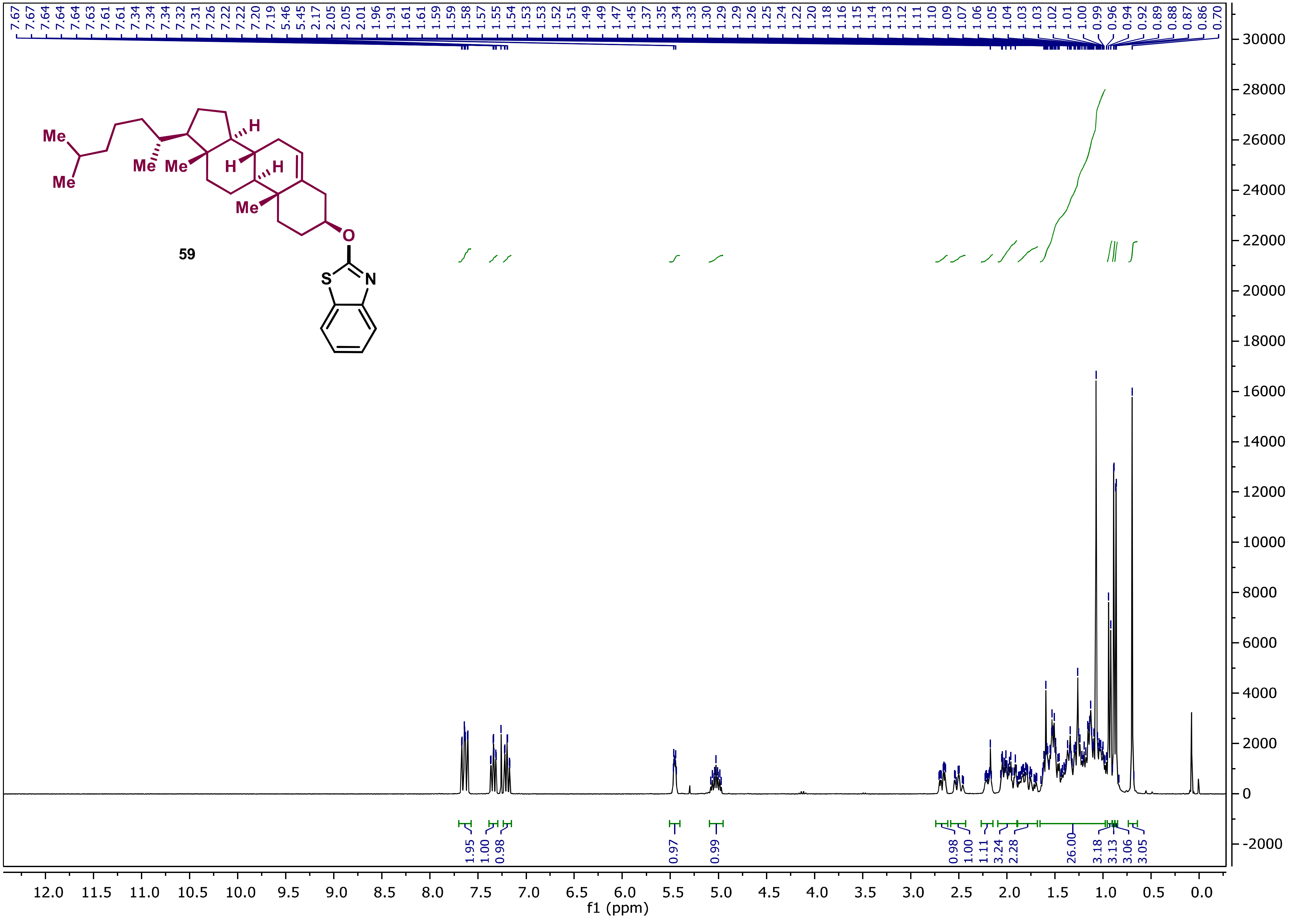
59
Me
Me
Me
Me
Me
H
H
H
O
S
N
7.67 7.67 7.64 7.64 7.64 7.63 7.61 7.61 7.34 7.34 7.34 7.32 7.31 7.26 7.22 7.22 7.20 7.19 5.46 5.45 2.17 2.05 2.05 2.01 1.96 1.91 1.61 1.61 1.59 1.59 1.58 1.57 1.55 1.54 1.53 1.53 1.52 1.51 1.49 1.49 1.47 1.45 1.37 1.35 1.34 1.33 1.30 1.29 1.29 1.26 1.25 1.24 1.22 1.20 1.18 1.16 1.15 1.14 1.13 1.12 1.11 1.10 1.09 1.07 1.06 1.05 1.04 1.03 1.03 1.02 1.01 1.00 0.99 0.96 0.94 0.92 0.89 0.88 0.87 0.86 0.70
1.95 1.00 0.98 0.97 0.99 0.98 1.00 1.11 3.24 2.28 26.00 3.18 3.13 3.06 3.05
f1 (ppm)

^{13}C NMR

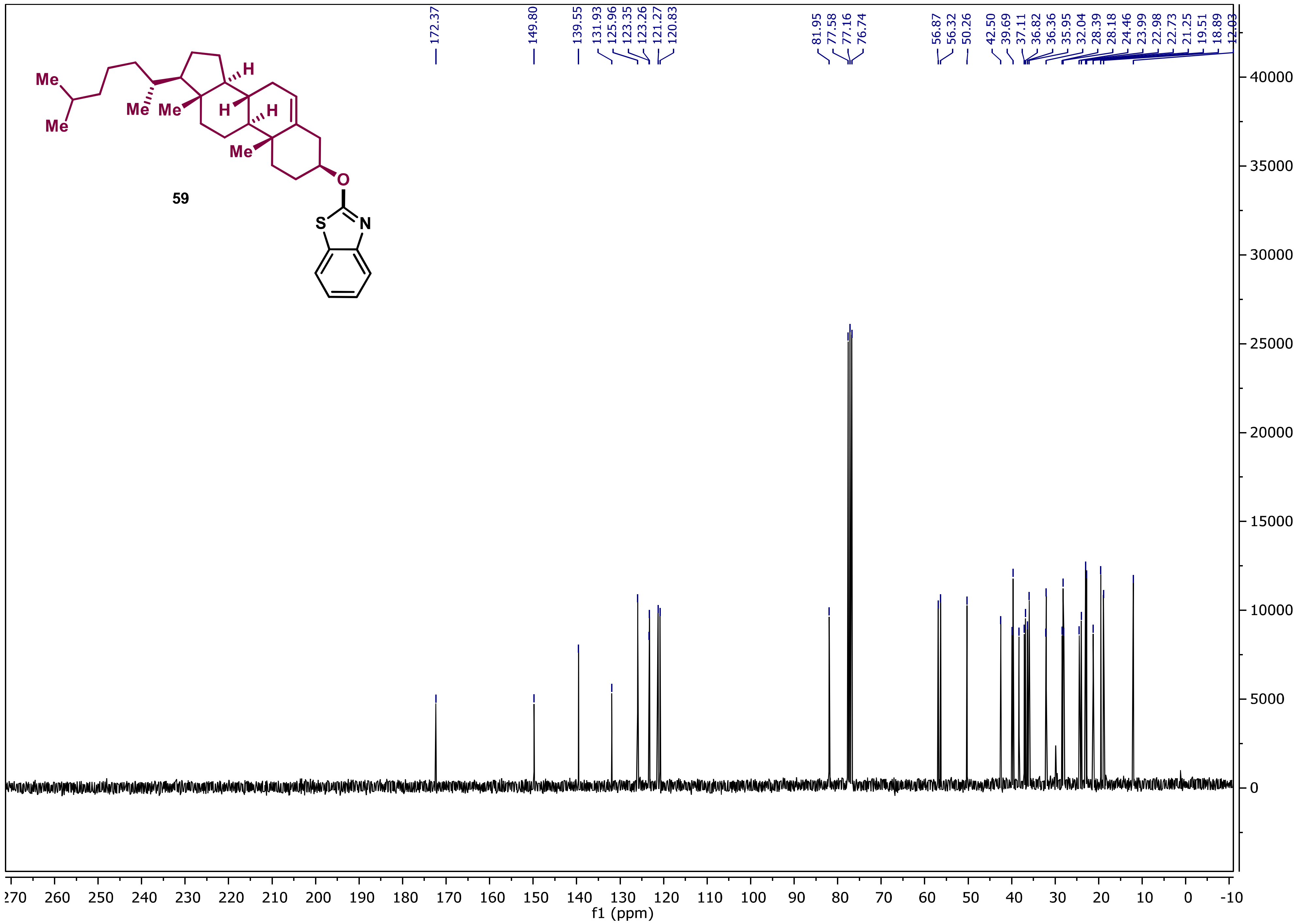
172.37
149.80
139.55
131.93
125.96
123.35
123.26
121.27
120.83
81.95
77.58
77.16
76.74
56.87
56.32
50.26
42.50
39.69
37.11
36.82
36.36
35.95
32.04
28.39
28.18
24.46
23.99
22.98
22.73
21.25
19.51
18.89
12.03
Me
Me
Me
Me
H
H
H
Me
O
S
N
59
f1 (ppm)

Mass to be matched (m/z): 542.342660 Charge: 1

Mass Tolerance: ±0.005000
Restriction of atom numbers:

C	H	N	O	S	Na
1-100	1-100	1-3	max 5	1-1	1-1

Number of calculated Formulas: 2

Formula	Diff.(ppm)	theor. m/z
C34 H49 N1 O1 S1 Na1	0.08	542.342705
C29 H49 N3 O3 S1 Na1	-7.33	542.338683

Datum	19.11.2020
Analyse:	150538c-00.RAW
Sigel:	GHC-GA-454-01
COP: Dr. Clement Ghiazza	
Messung:	HRMS
Methode:	ESIpos
Lösungsmittel:	CH2Cl2+CH3OH
Spektrometer:	Exactive
Auswerter:	Kampen (2242)

Suggestion:
C34H49N1O1S1 MW 519

characteristical ion
542 = [519 + Na]+

^{1}H NMR

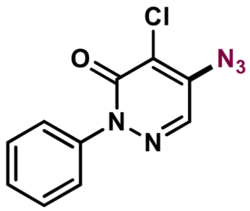

60

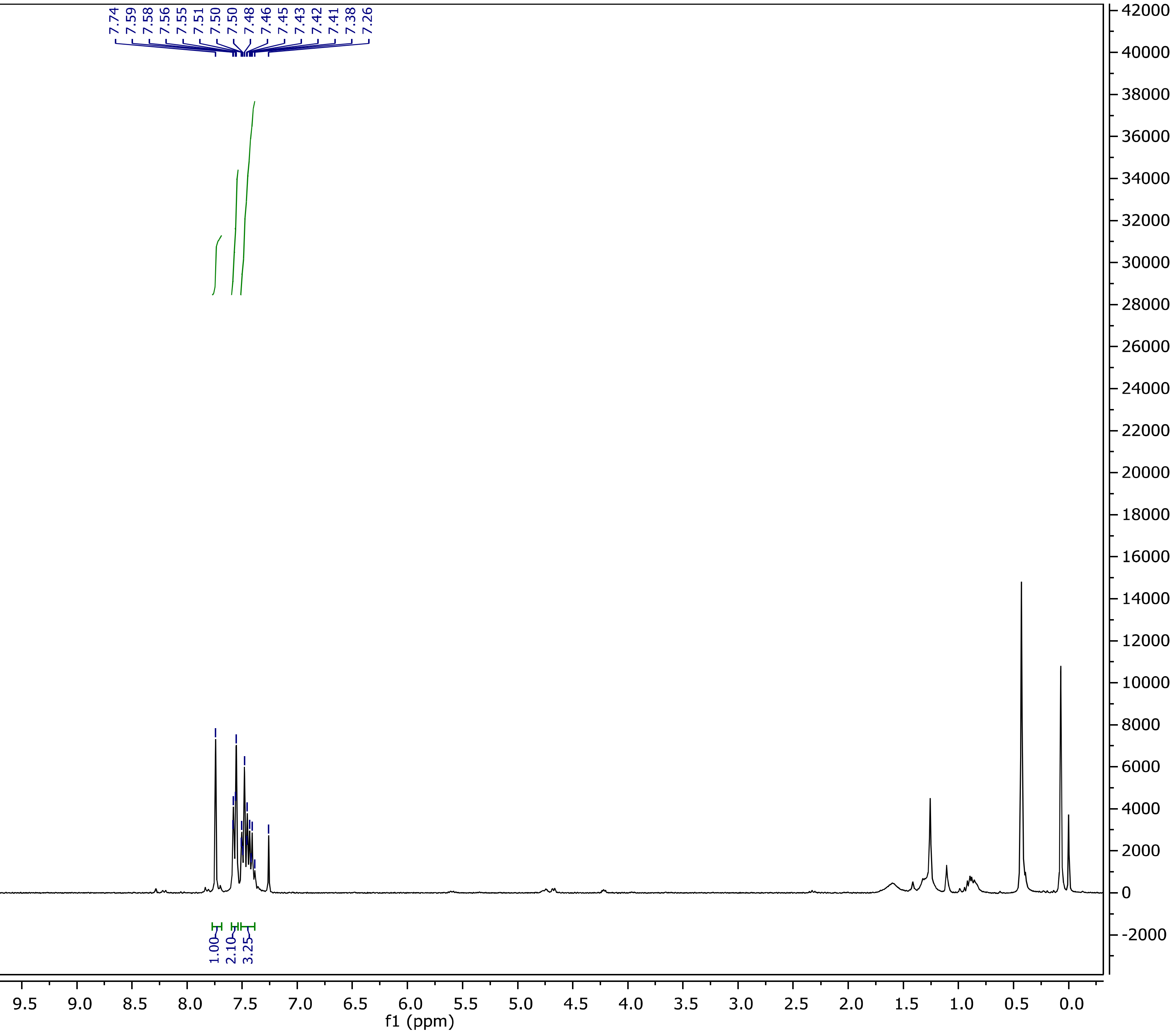
7.74
7.59
7.58
7.56
7.55
7.51
7.50
7.50
7.48
7.46
7.45
7.43
7.42
7.41
7.38
7.26
1.00
2.10
3.25
12.0 11.5 11.0 10.5 10.0 9.5 9.0 8.5 8.0 7.5 7.0 6.5 6.0 5.5 5.0 4.5 4.0 3.5 3.0 2.5 2.0 1.5 1.0 0.5 0.0
f1 (ppm)
42000 40000 38000 36000 34000 32000 30000 28000 26000 24000 22000 20000 18000 16000 14000 12000 10000 8000 6000 4000 2000 0 -2000

Mass to be matched (m/z): 270.015390 Charge: 1

Mass Tolerance: ±0.005000
Restriction of atom numbers:

C	H	Cl	N	O	Na
1-100	1-100	1-1	1-5	max 105	1-1

Number of calculated Formulas: 4

Formula	Diff.(ppm)	theor. m/z
C10 H6 Cl1 N5 O1 Na1	-0.31	270.015306
C12 H8 Cl1 N2 O2 Na1	4.66	270.016649
C9 H10 Cl1 N1 O5 Na1	-5.26	270.013971
C7 H8 Cl1 N4 O4 Na1	-10.23	270.012627

Datum	23.10.2020
Analyse:	150103b-00
Sigel:	GHC-AA-089-01
COP: Dr. Clement Ghiazza	
Messung:	HRMS
Methode:	ESIpos
Lösungsmittel:	CH2Cl2+CH3OH
Spektrometer:	Exactive
Auswerter:	Kampen (2242)

Suggestion:
C10H6Cl1N5O1 MW 247

characteristical ion
270 = [247 + Na]+

^{1}H NMR

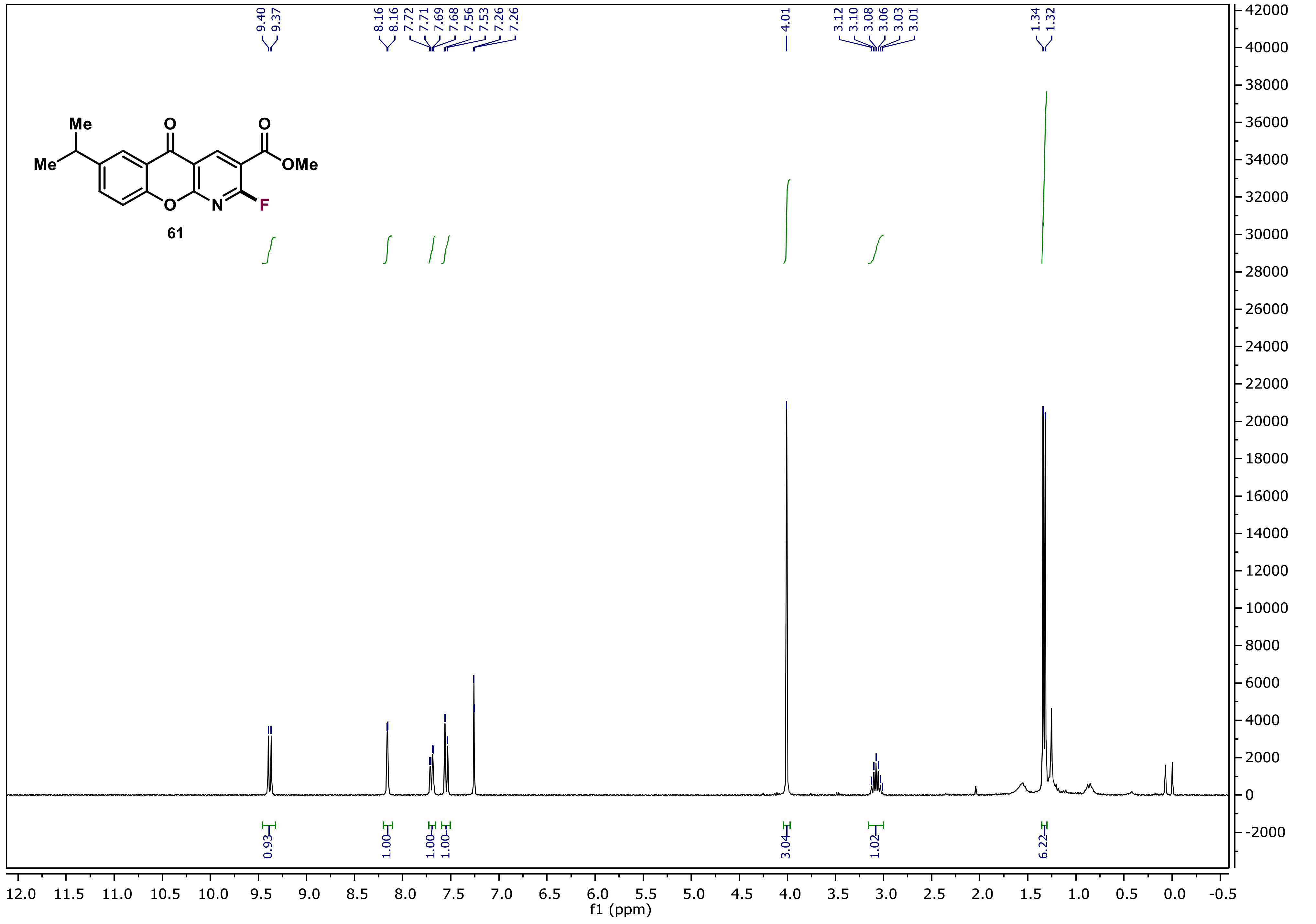
9.40
9.37
8.16
8.16
7.72
7.71
7.69
7.68
7.56
7.53
7.26
7.26
4.01
3.12
3.10
3.08
3.06
3.03
3.01
1.34
1.32
Me
Me
O
O
OMe
O
N
F
61
0.93
1.00
1.00
1.00
3.04
1.02
6.22
f1 (ppm)

^{13}C NMR

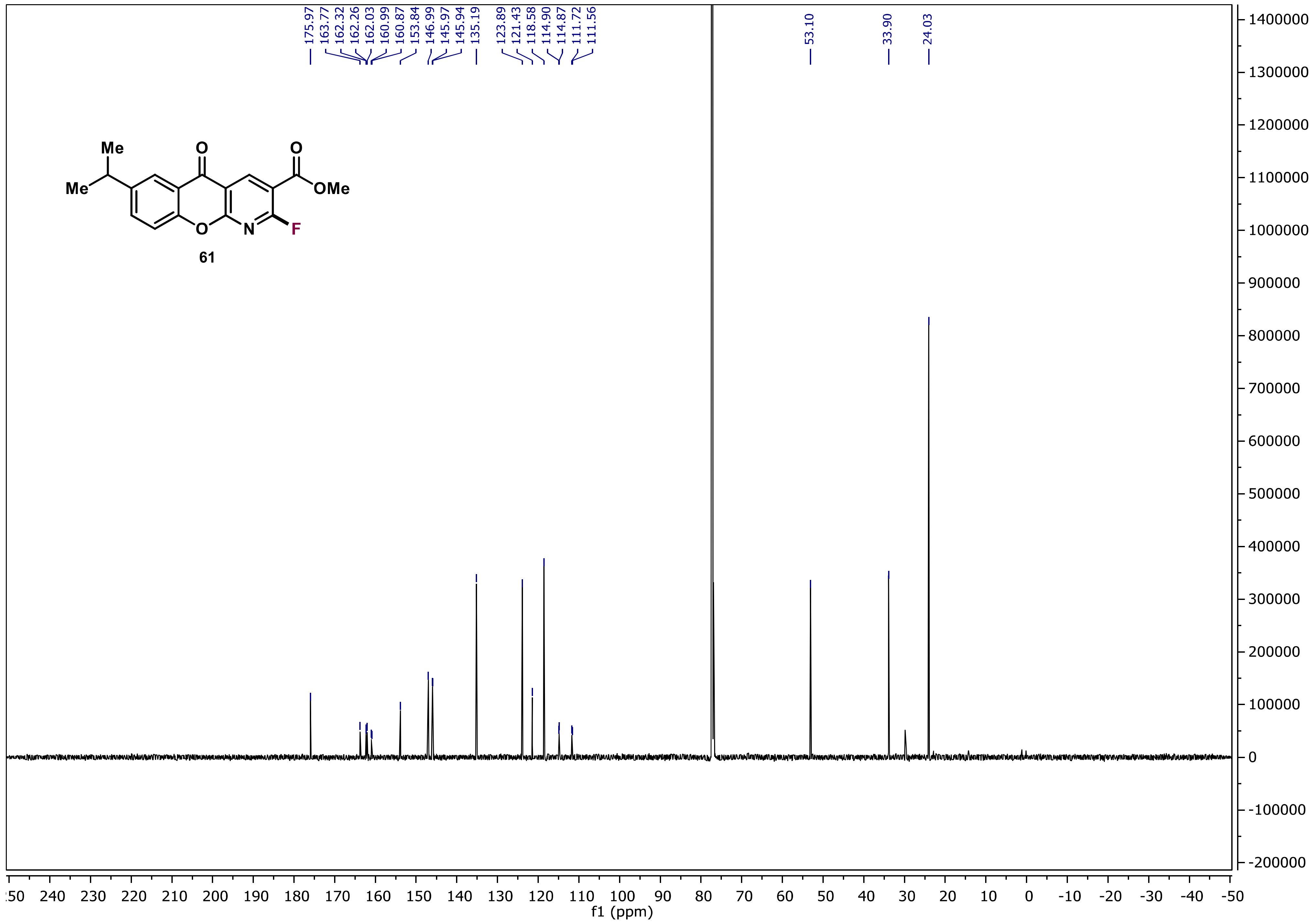
175.97
163.77
162.32
162.26
162.03
160.99
160.87
153.84
146.99
145.97
145.94
135.19
123.89
121.43
118.58
114.90
114.87
111.72
111.56
53.10
33.90
24.03
Me
Me
O
O
OMe
O
N
F
61
1400000
1300000
1200000
1100000
1000000
900000
800000
700000
600000
500000
400000
300000
200000
100000
0
-100000
-200000
250 240 230 220 210 200 190 180 170 160 150 140 130 120 110 100 90 80 70 60 50 40 30 20 10 0 -10 -20 -30 -40 -50
f1 (ppm)

^{19}F NMR

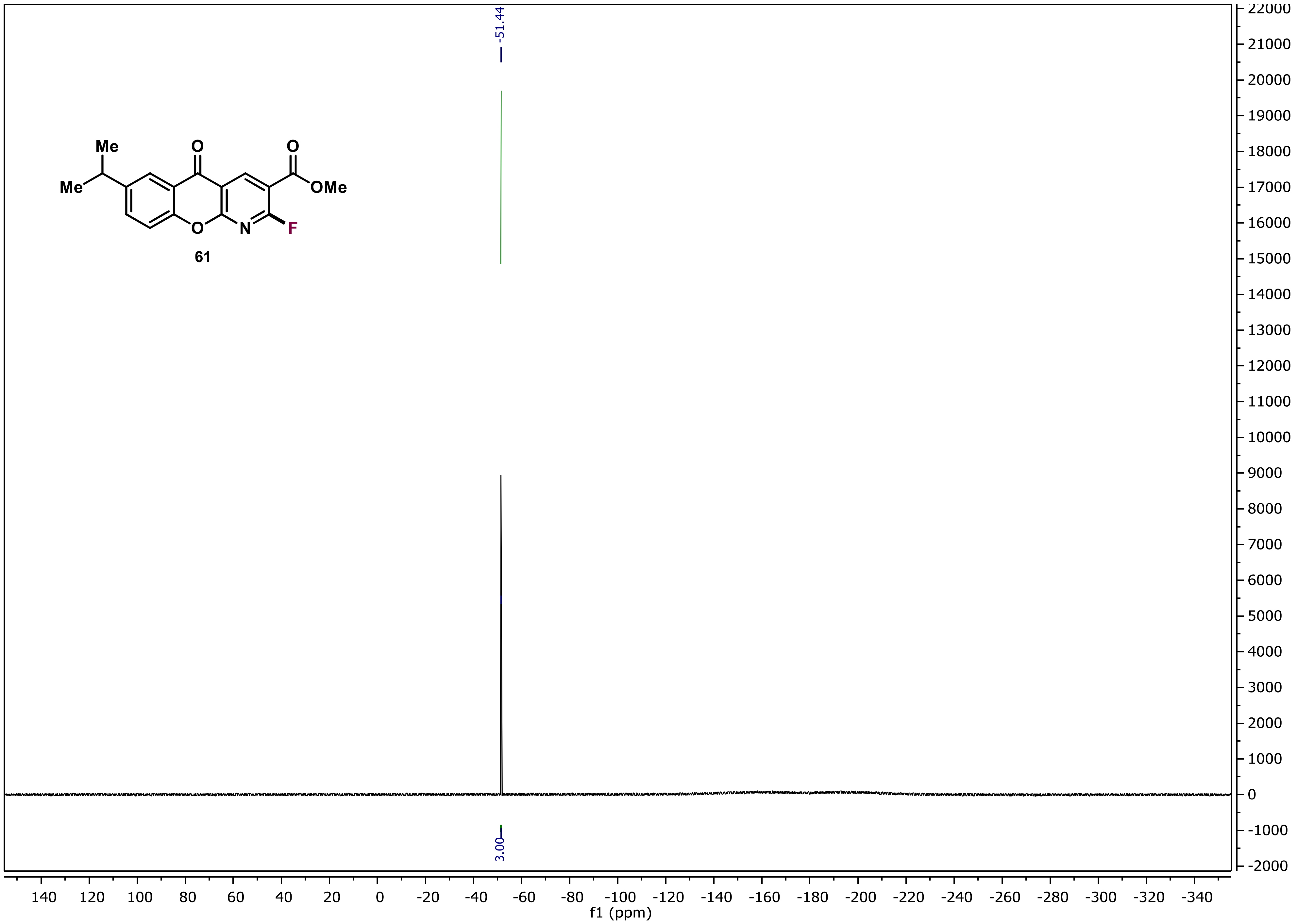

Mass to be matched (m/z): 315.090100 Charge: 1

Mass Tolerance: ±0.050000
Restriction of atom numbers:

C	H	N	O	F
1-100	1-100	1-2	1-4	1-1

Number of calculated Formulas: 8

Formula	Diff.(ppm)	theor. m/z
C17 H14 N1 O4 F1	0.12	315.090137
C20 H12 N2 O1 F1	8.62	315.092816
C16 H12 N2 O4 F1	-39.79	315.077561
C21 H14 N1 O1 F1	48.53	315.105392
C20 H10 N1 O2 F1	-66.94	315.069007
C17 H16 N2 O3 F1	75.68	315.113946
C19 H8 N2 O2 F1	-106.86	315.056431
C18 H18 N1 O3 F1	115.59	315.126522

11.11.2020
File: 150390d-00
Analyse: GHC-GA-435-01
COP: Dr. Clement Ghiazza

Messung: (HRMS)
Ionisierung: EI
Spektrometer: Q Exactive GC Orbitrap
Säule: MS50 RTX-1+VS
Länge: 30+7
Temp.: 35-10-285-5
GC-Nr.:
ELNA-Nr.: 28353

Auswerter: Haupt (2243)

Suggestion:
C17H14N1O4F1 MW: 315

^{1}H NMR

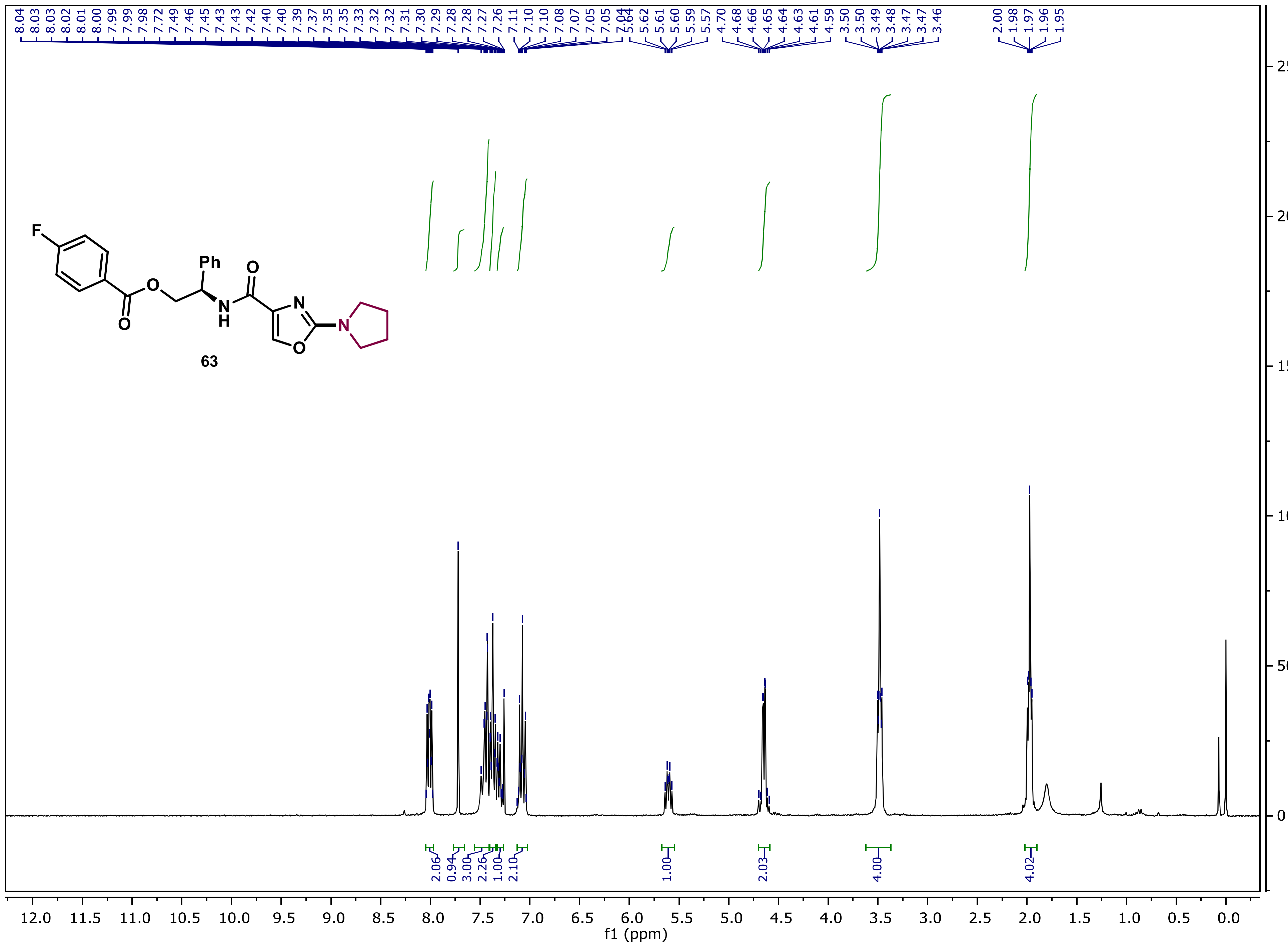
8.04 8.03 8.03 8.02 8.01 8.00 7.99 7.99 7.98 7.72 7.49 7.46 7.45 7.43 7.42 7.40 7.40 7.39 7.37 7.35 7.35 7.33 7.32 7.32 7.31 7.30 7.29 7.28 7.28 7.27 7.26 7.11 7.10 7.10 7.08 7.07 7.05 7.05 7.04 5.64 5.62 5.61 5.60 5.59 5.57 4.70 4.68 4.66 4.65 4.64 4.63 4.61 4.59 3.50 3.50 3.49 3.48 3.47 3.47 3.46 2.00 1.98 1.97 1.96 1.95
F
Ph
O
O
N
H
O
N
O
N
63
2.06
0.94
3.00
2.26
1.00
2.10
1.00
2.03
4.00
4.02
25000
20000
15000
10000
5000
0
12.0 11.5 11.0 10.5 10.0 9.5 9.0 8.5 8.0 7.5 7.0 6.5 6.0 5.5 5.0 4.5 4.0 3.5 3.0 2.5 2.0 1.5 1.0 0.5 0.0
f1 (ppm)

^{13}C NMR

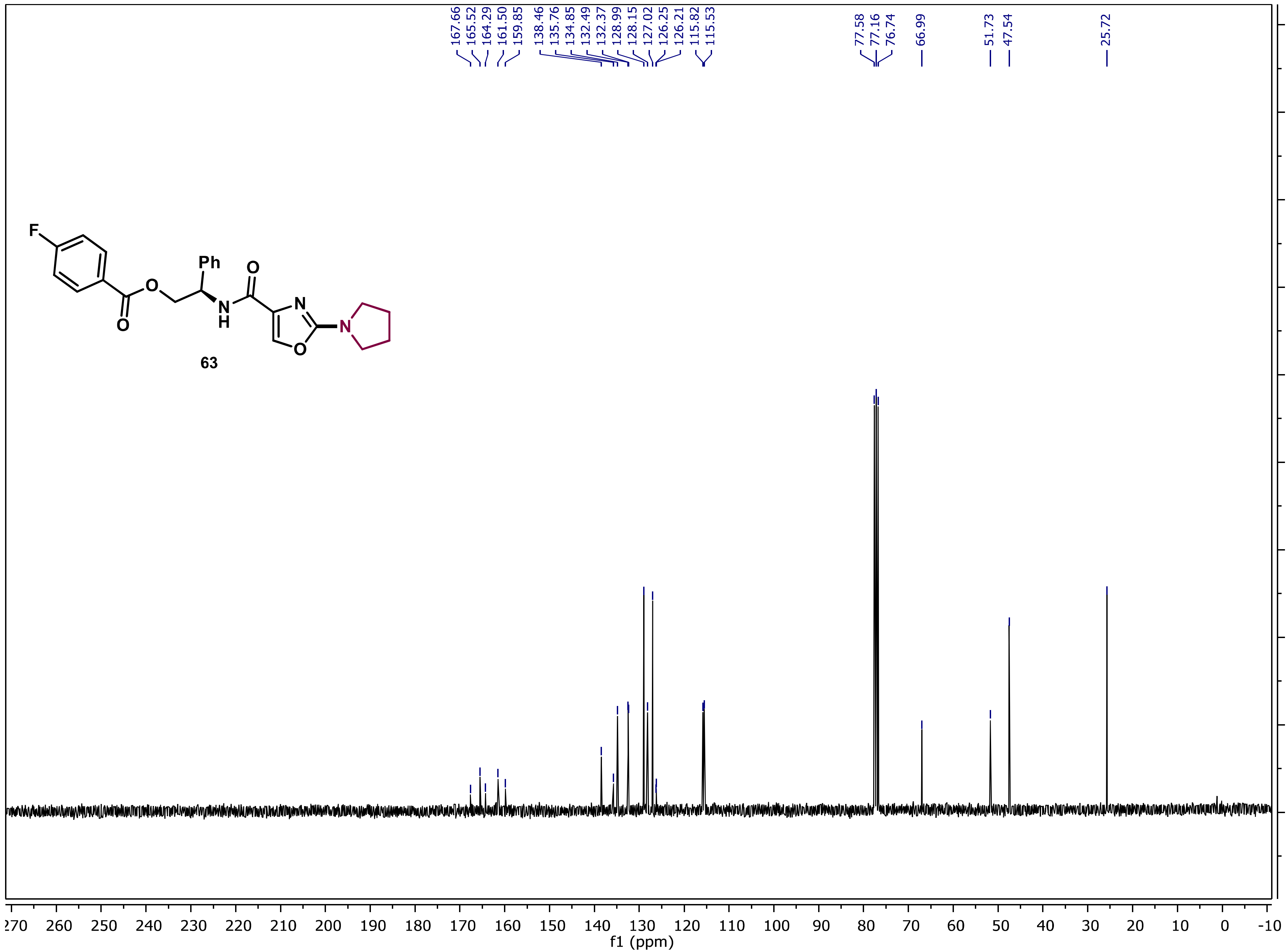

167.66
165.52
164.29
161.50
159.85
138.46
135.76
134.85
132.49
132.37
128.99
128.15
127.02
126.25
126.21
115.82
115.53
77.58
77.16
76.74
66.99
51.73
47.54
25.72
F
Ph
O
N
H
N
O
63
45000
40000
35000
30000
25000
20000
15000
10000
5000
0
270 260 250 240 230 220 210 200 190 180 170 160 150 140 130 120 110 100 90 80 70 60 50 40 30 20 10 0 -10
f1 (ppm)

^{19}F NMR

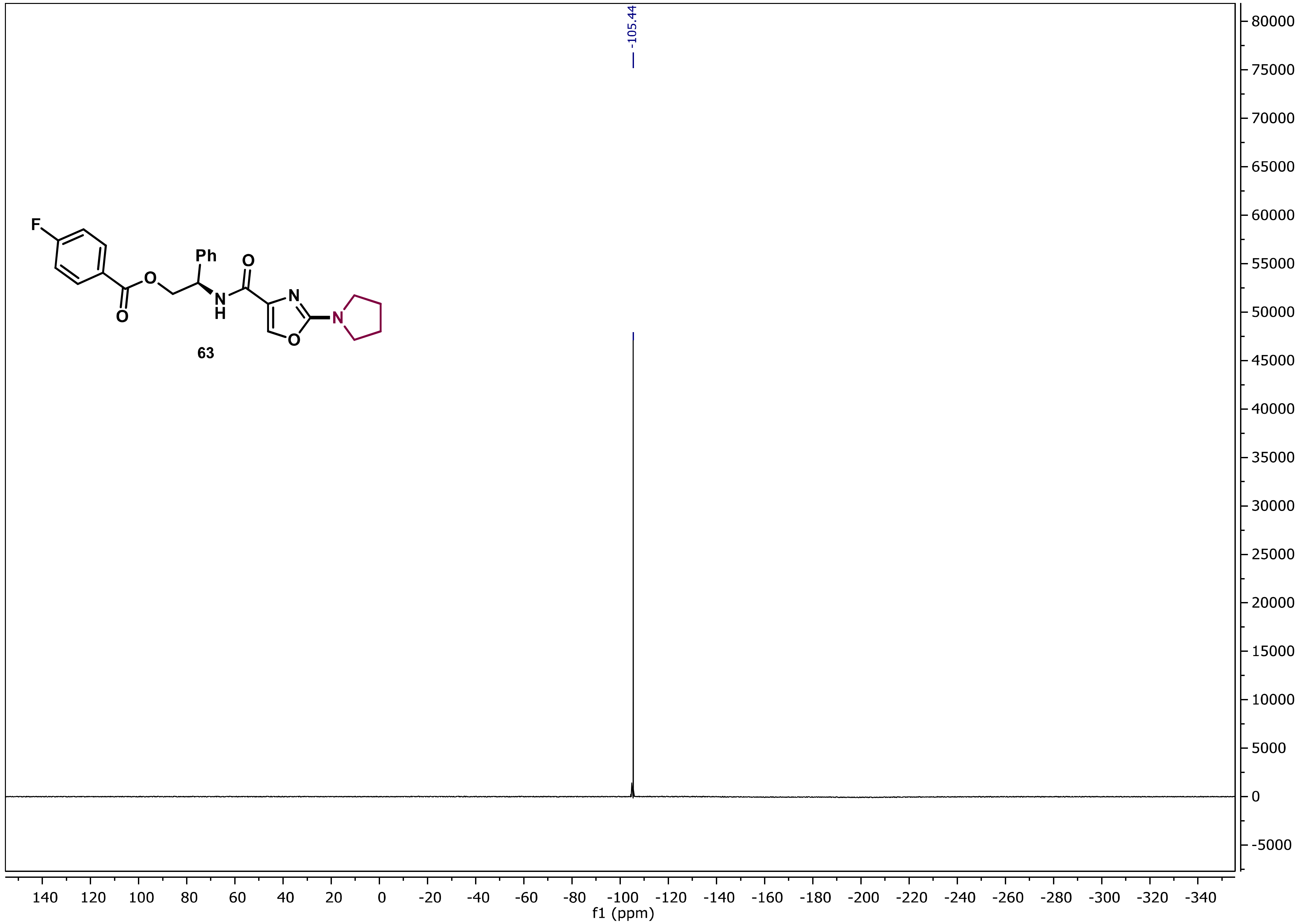

Mass to be matched (m/z): 424.166450 Charge: 1

Mass Tolerance: ±0.005000
Restriction of atom numbers:

C	H	N	O	F
1-110	1-100	1-3	1-4	1-3

Number of calculated Formulas: 3

Formula	Diff.(ppm)	theor. m/z
C23 H23 N3 O4 F1	0.61	424.166709
C24 H21 N3 O1 F3	-7.85	424.163122
C28 H23 N1 O2 F1	10.10	424.170732

22.10.2020
File: 150051b-00

Analyse: GHC-AA-087-01
COP: Dr. Clement Ghiazza

Messung:	HRMS ESIpos
Lösemittel:	CH3OH
Spektrometer:	Exactive
Auswerter:	Kohler (2243)

Suggestion:
C23H22N3O4F1   MW 423

Characteristicial ions:
424 = [423 + H]+

^{1}H NMR

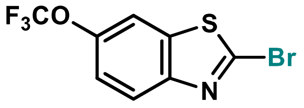

66

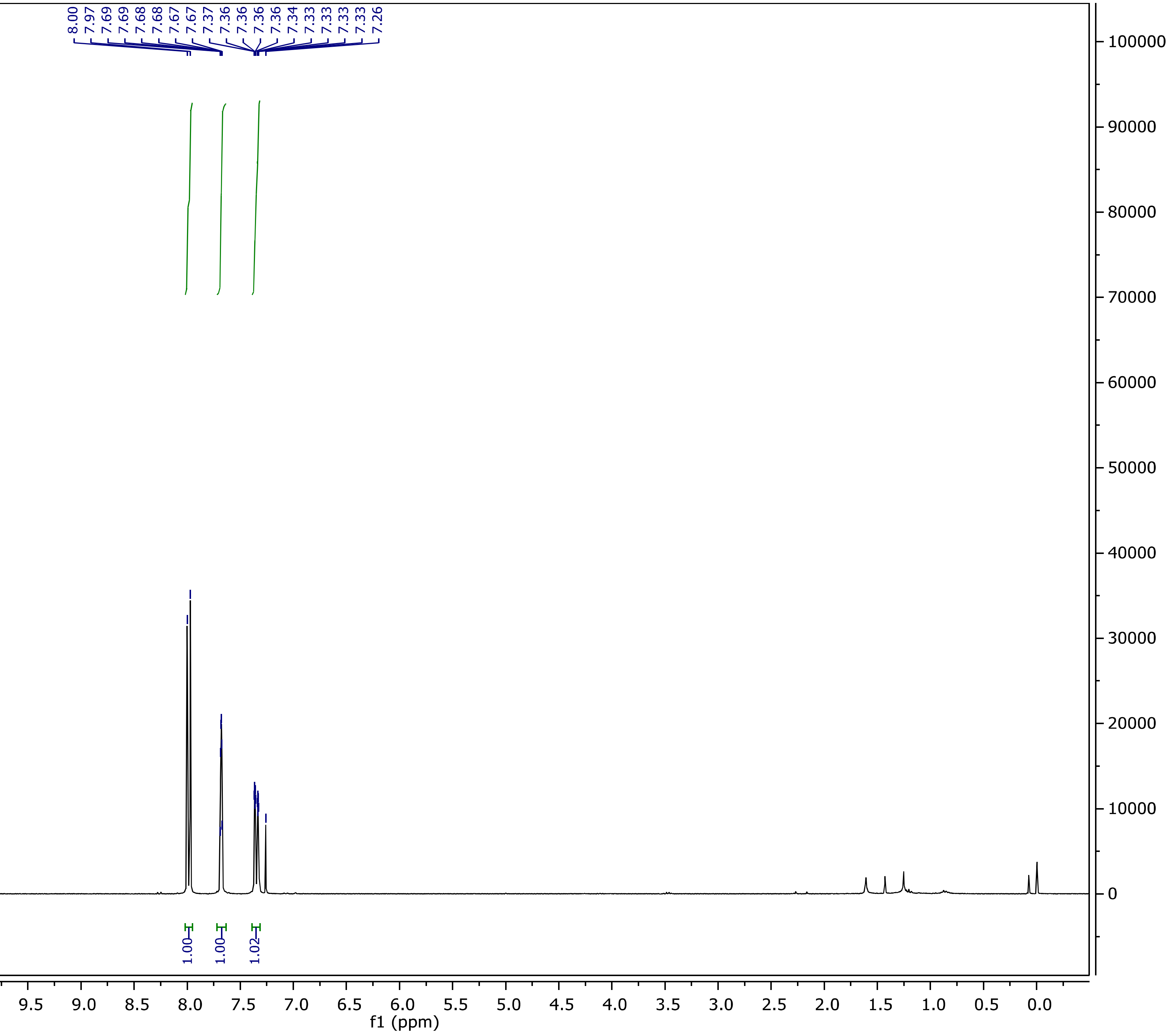

8.00
7.97
7.69
7.69
7.68
7.68
7.67
7.67
7.37
7.36
7.36
7.36
7.36
7.34
7.33
7.33
7.33
7.33
7.26
1.00
1.00
1.02
f1 (ppm)

^{13}C NMR

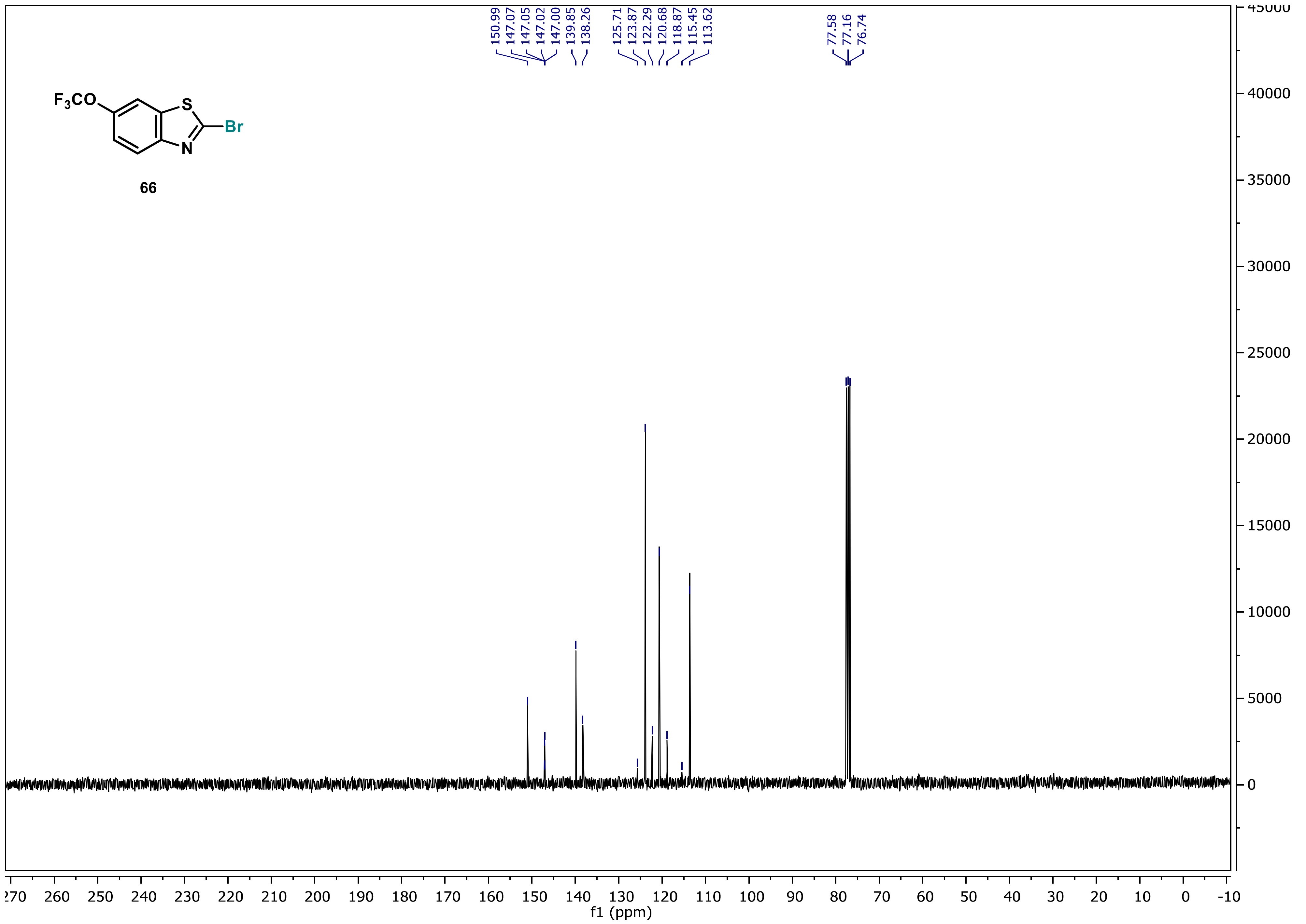

F3CO
S
N
Br
66
150.99
147.07
147.05
147.02
147.00
139.85
138.26
125.71
123.87
122.29
120.68
118.87
115.45
113.62
77.58
77.16
76.74
f1 (ppm)

^{19}F NMR

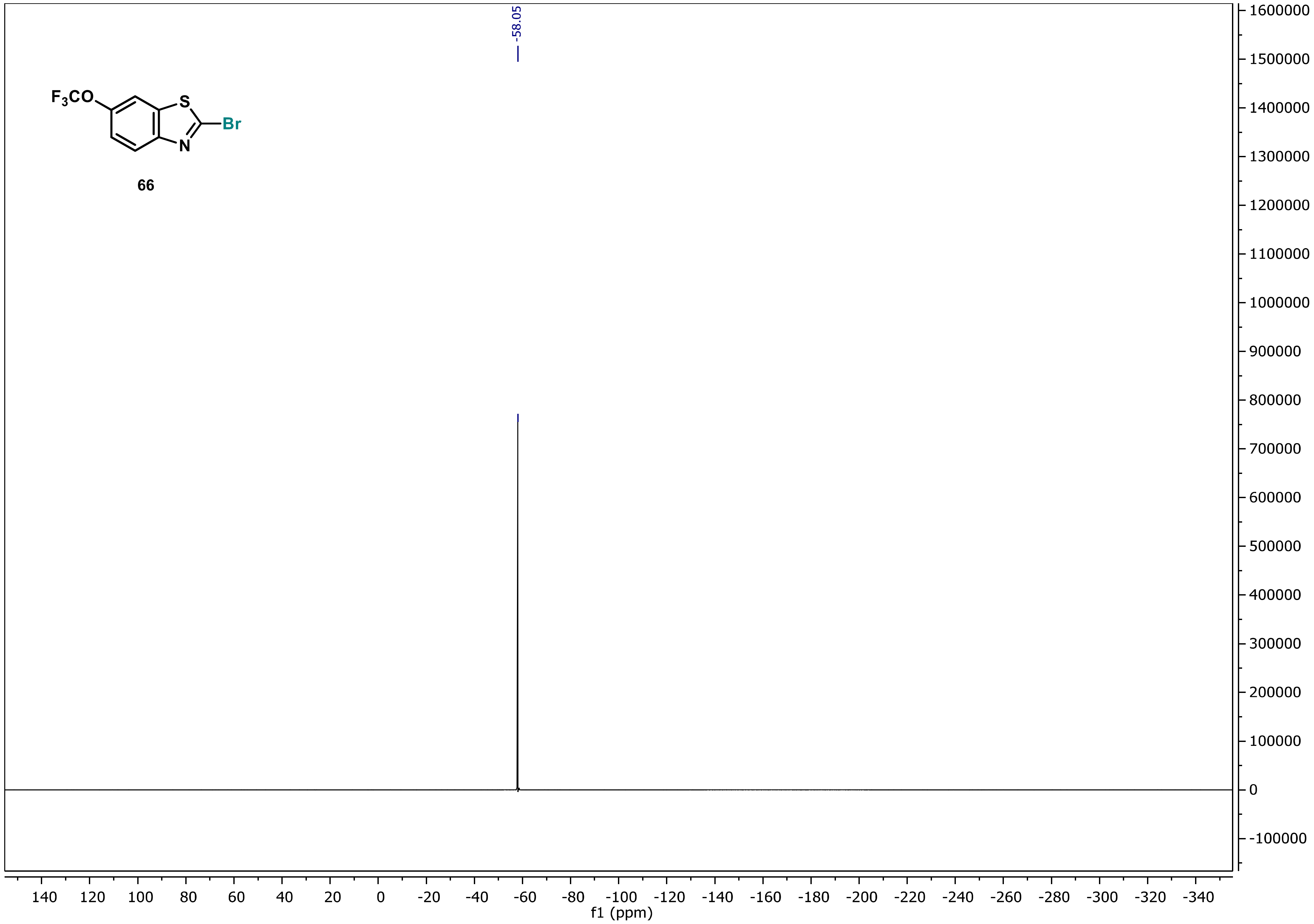
F3CO
S
N
Br
66
-58.05
1600000
1500000
1400000
1300000
1200000
1100000
1000000
900000
800000
700000
600000
500000
400000
300000
200000
100000
0
-100000
140 120 100 80 60 40 20 0 -20 -40 -60 -80 -100 -120 -140 -160 -180 -200 -220 -240 -260 -280 -300 -320 -340
f1 (ppm)

No.	MW.	Comment
1	297	Spectrum correlates with your expected structure<br>Ref.-spectrum: OU2077:

HRMS see below

17.03.2021
File: 152336a-00.raw
Analyse: GHC-GA-645-01
COP: Dr. Clement Ghiazza

Messung:	GC-MS
Ionisierung:	GC-EI
Spektrometer:	Q Exactive GC Orbitrap
Säule:	MS 81 ZB1ms
Länge:	30
Temp.:	35-15-300-3
GC-Nr.:	-
ELNA-Nr.:	30257
Auswerter:	Vetere (2243)

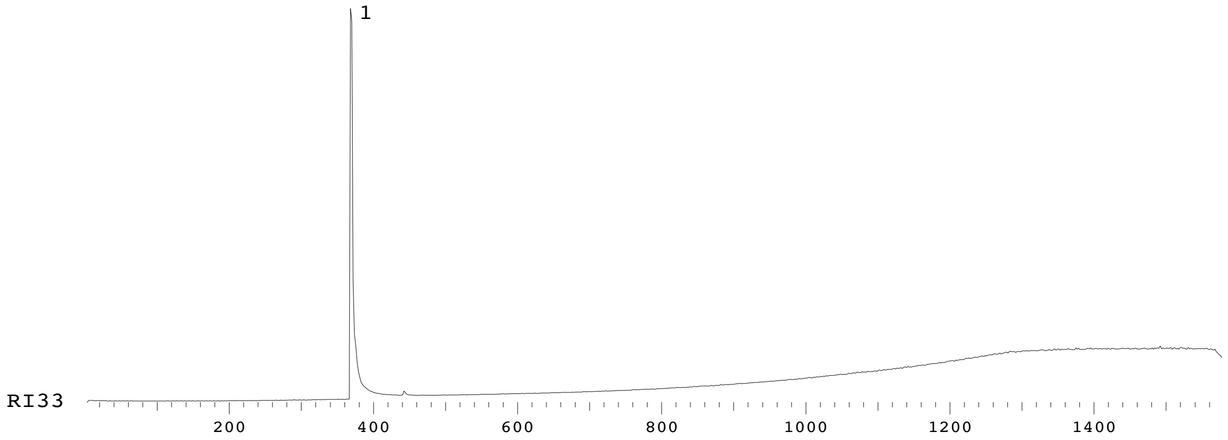

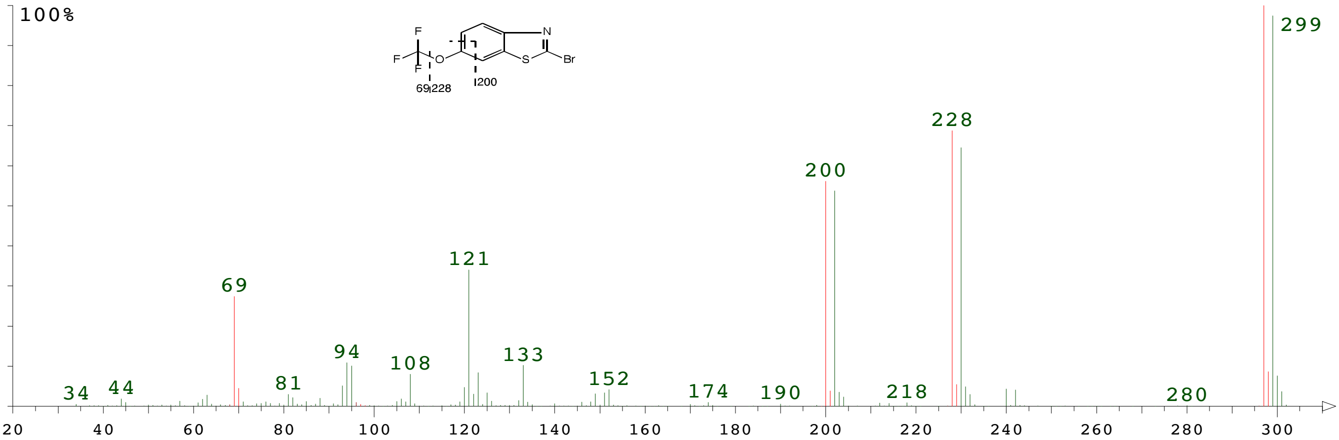

OU2077 14% MW:297/298.05 C8H3Br1F3N1O1S1

34	0.47	63	2.83	84	0.37	108	7.98	133	10.19	175	0.21	231	4.84
37	0.13	64	0.53	85	1.24	109	0.63	134	1.06	190	0.55	232	2.93
38	0.15	65	0.10	86	0.26	111	0.12	135	0.42	192	0.08	233	0.41
39	0.19	66	0.44	87	0.60	113	0.08	140	0.68	198	0.22	240	4.31
41	0.23	67	0.28	88	1.97	115	0.20	141	0.09	200	56.13	241	0.28
42	0.11	68	0.41	89	0.24	117	0.44	146	1.08	201	3.87	242	4.09
43	0.15	69	27.39	91	0.63	118	0.35	147	0.16	202	53.72	243	0.27
44	1.84	70	4.51	92	0.38	119	1.16	148	1.11	203	3.55	244	0.16
45	0.95	71	1.16	93	5.09	120	4.72	149	3.14	204	2.31	278	0.12
49	0.09	72	0.19	94	10.83	121	33.99	150	0.33	205	0.12	280	0.13
50	0.31	73	0.20	95	10.08	122	3.05	151	3.32	207	0.09	297	100.00
51	0.21	74	0.65	96	0.93	123	8.35	152	4.13	211	0.10	298	8.60
53	0.29	75	0.72	97	0.52	124	0.49	153	0.35	212	0.77	299	97.44
54	0.07	76	1.11	98	0.14	125	3.38	154	0.15	213	0.12	300	7.60
55	0.33	77	0.76	99	0.21	126	1.26	156	0.16	214	0.72	301	3.69
56	0.19	78	0.07	100	0.16	127	0.15	158	0.09	218	0.86	302	0.33
57	1.30	79	0.72	103	0.10	128	0.26	163	0.15	219	0.18		
58	0.14	80	0.35	104	0.26	129	0.24	170	0.48	227	0.07		
60	0.07	81	2.98	105	1.22	130	0.26	171	0.17	228	68.77		
61	0.91	82	2.12	106	1.83	131	0.27	173	0.20	229	5.44		
62	1.72	83	0.60	107	1.12	132	1.41	174	0.95	230	64.55		

OU2077 14% MW:297/298.05 C8H3Br1F3N1O1S1 lim: 0.07%

Mass to be matched (m/z): 296.906890 Charge: 1

Mass Tolerance: ±0.001000
Restriction of atom numbers:

C	H	N	O	Br	S	F
			max 3	1-1	max 1	

Number of calculated Formulas: 5

Formula	Diff.(ppm)	theor. m/z
C8 H3 N1 O1 Br1 S1 F3	-1.15	296.906547
C3 H2 N4 O1 Br1 S1 F4	-1.83	296.906346
C6 N7 O1 Br1 S1	-1.98	296.906303
C8 H2 N4 O2 Br1 S1	2.55	296.907648
C5 H4 N1 O2 Br1 S1 F4	2.70	296.907691

Characteristic ions (singly charged):
297 = [M]+

suggested composition of M:
C8H3 BrF3NOS

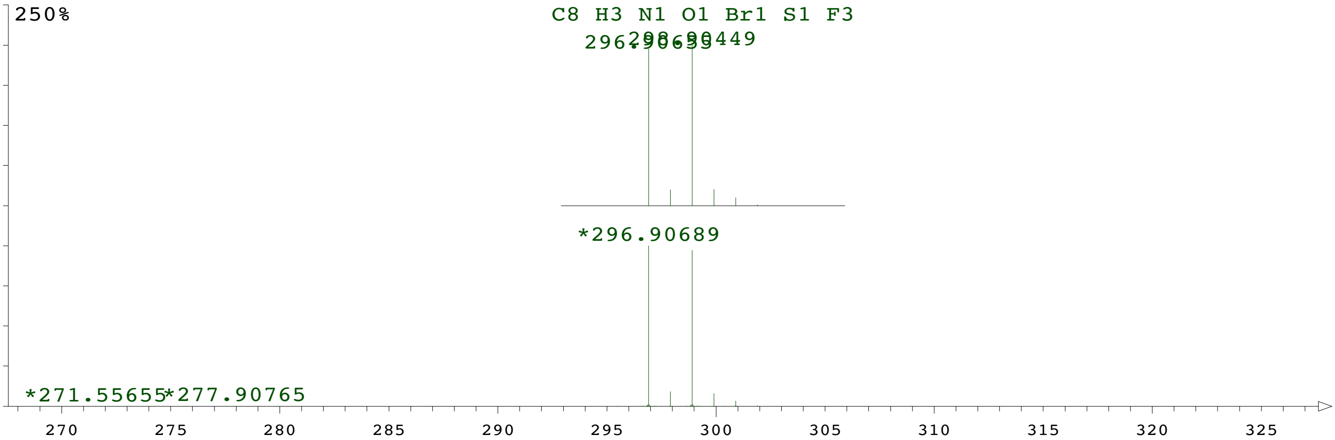

367(8:00) 51806 12% 152336a-00 * GHC-GA-645-01/GC-MS/GC-EI/QExactiveGC/MS 81 ZB1ms/30/35-1

^{1}H NMR

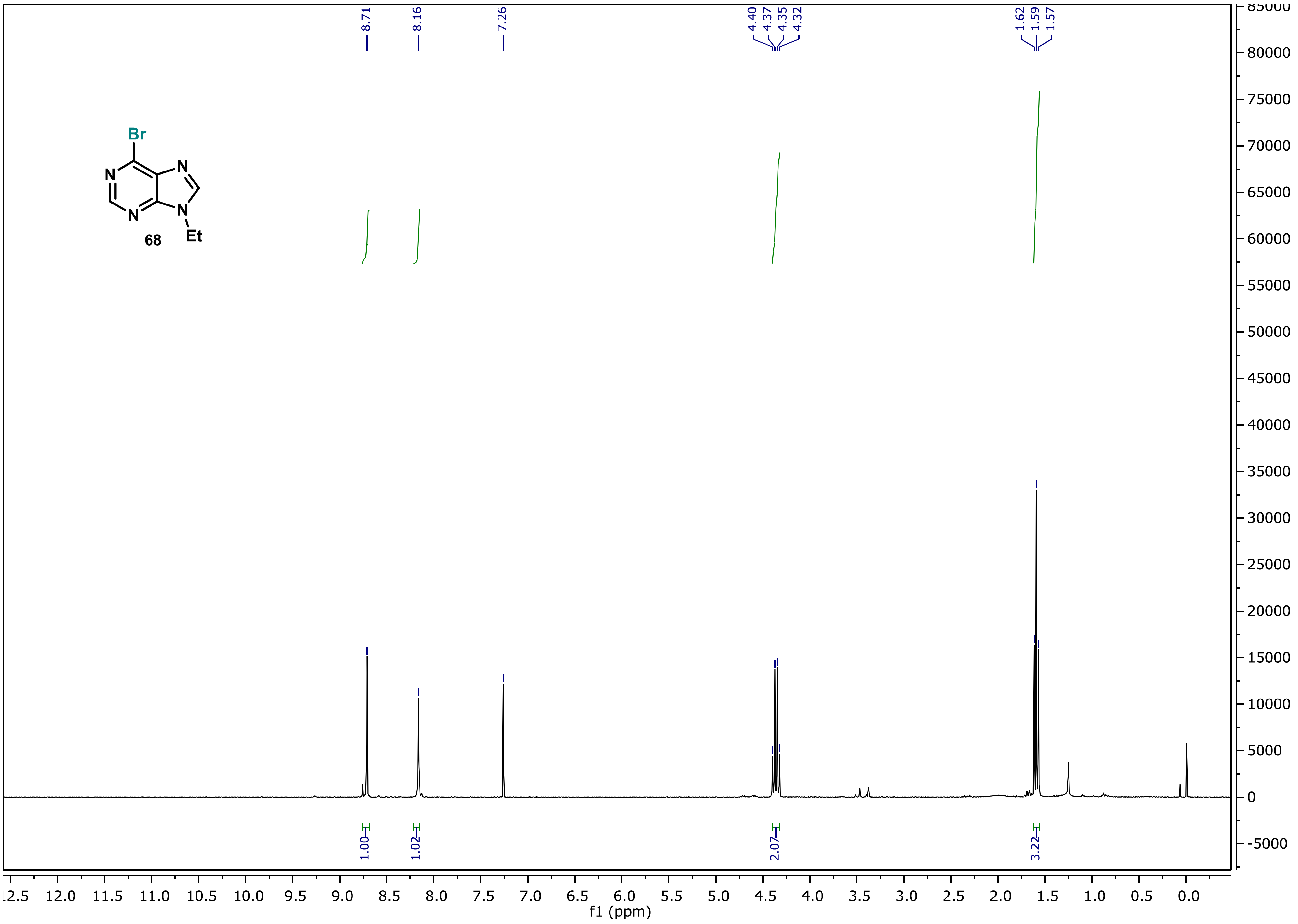

Br
N
N
N
N
Et
68
8.71
8.16
7.26
4.40
4.37
4.35
4.32
1.62
1.59
1.57
1.00
1.02
2.07
3.22
f1 (ppm)

^{13}C NMR

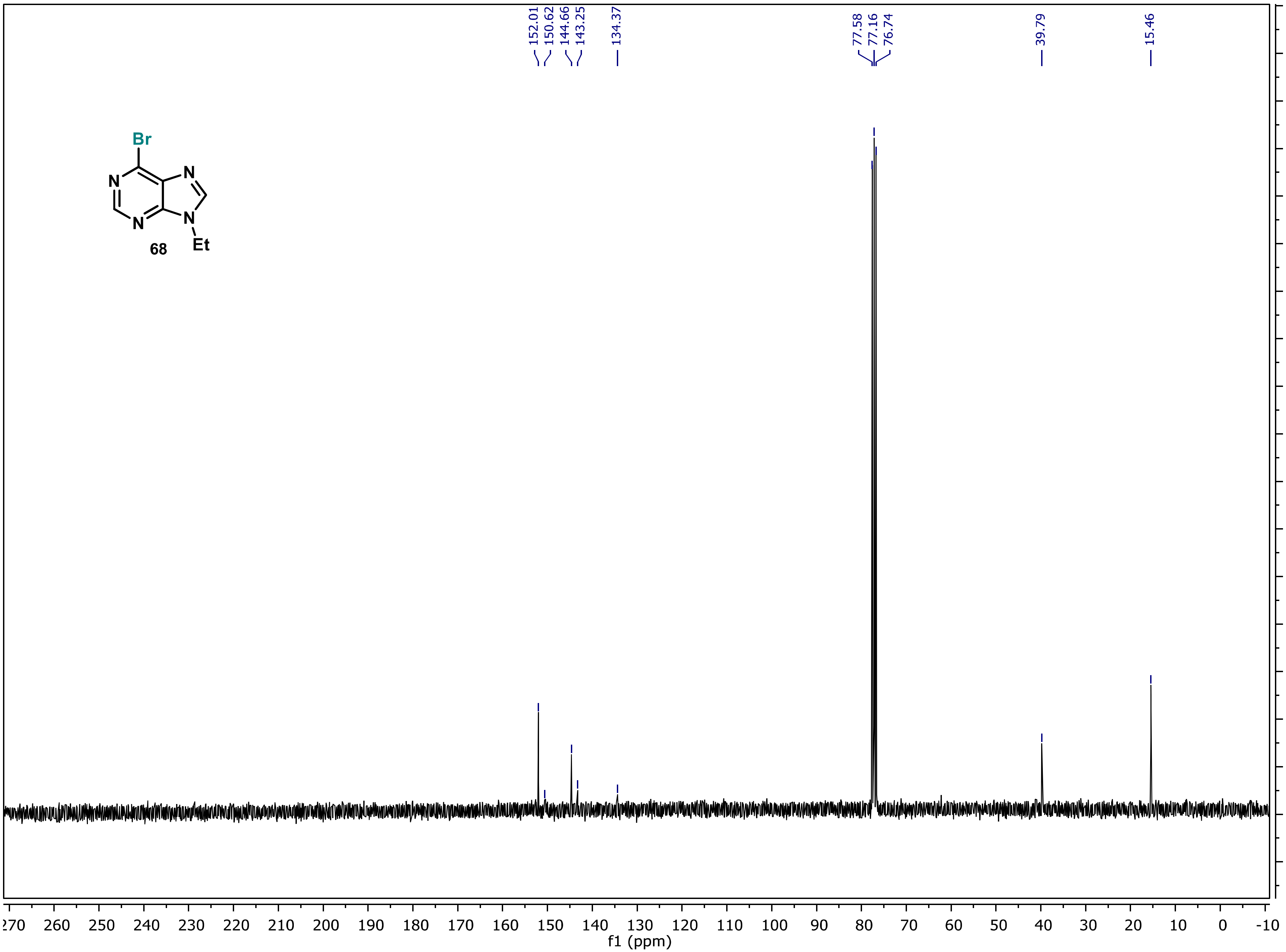
Br
N
N
N
N
Et
68
152.01
150.62
144.66
143.25
134.37
77.58
77.16
76.74
39.79
15.46
f1 (ppm)

Mass to be matched (m/z): 225.984890 Charge: 1

Mass Tolerance: ±0.050000
Restriction of atom numbers:

C	H	N	Br
1-100	1-100	1-4	1-1

Number of calculated Formulas: 4

Formula	Diff.(ppm)	theor. m/z
C7 H7 N4 Br1	-0.09	225.984870
C8 H9 N3 Br1	55.56	225.997446
C9 H11 N2 Br1	111.21	226.010023
C10 H13 N1 Br1	166.86	226.022599

22.03.2021
File: 152395b-00.raw
Analyse: GHC-GA-653-01
COP: Dr. Clement Ghiazza

Messung:	GC-MS
Ionisierung:	GC-EI
Spektrometer:	Q Exactive GC Orbitrap
Säule:	MS 81 ZB1ms
Länge:	30
Temp.:	35-15-300-3
GC-Nr.:	-
ELNA-Nr.:	30330

Auswerter: Haupt (2243)

Suggestion:
C7H7N4Br1 MW: 226

^{1}H NMR – in situ

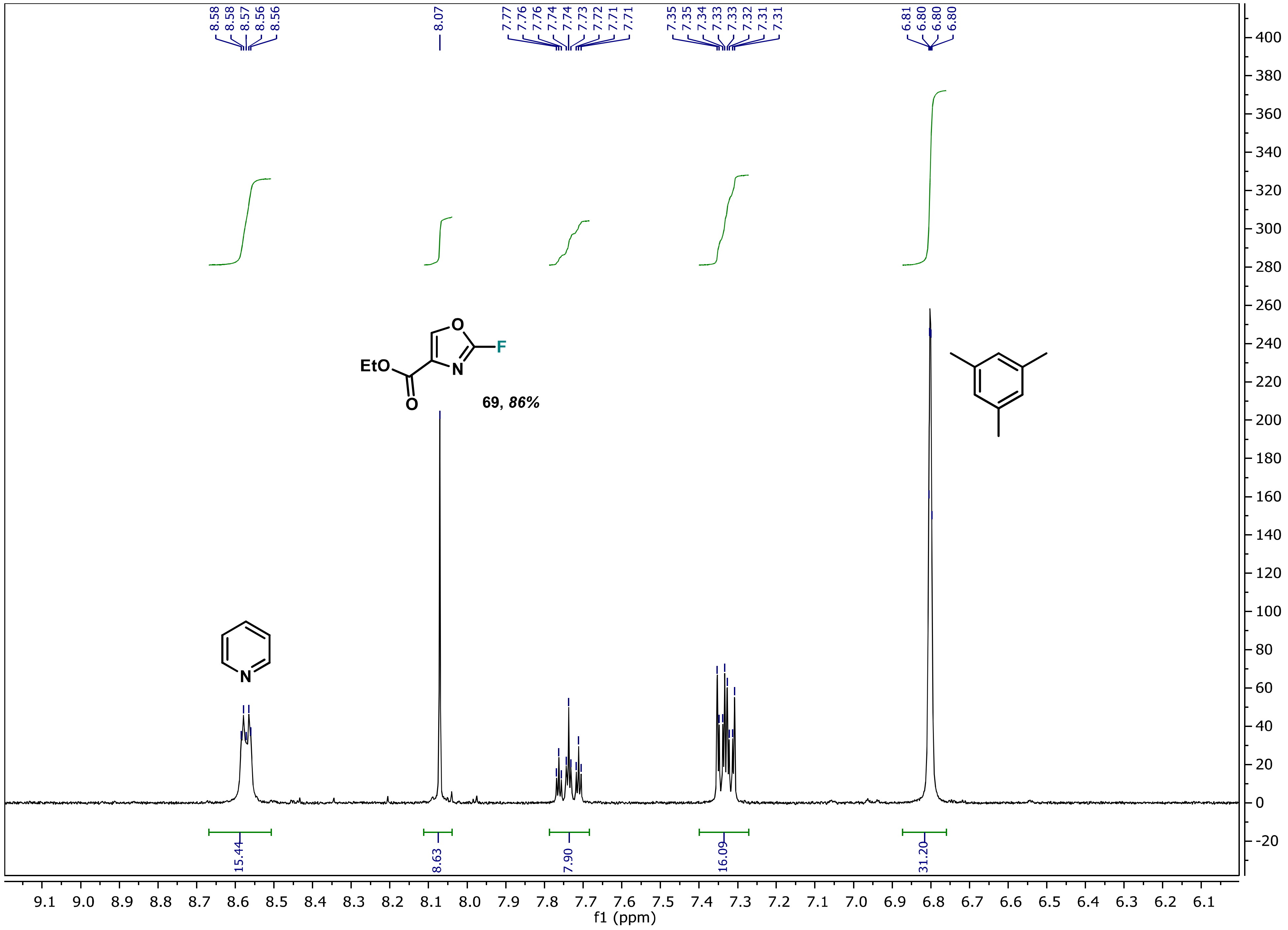

Mass to be matched (m/z): 182.022450 Charge: 1

Mass Tolerance: ±0.005000
Restriction of atom numbers:

C	H	F	N	O	Na
1-150	1-100	1-1	1-3	max 5	1-1

Number of calculated Formulas: 2

Formula	Diff.(ppm)	theor. m/z
C6 H6 F1 N1 O3 Na1	-0.32	182.022391
C9 H4 F1 N2 Na1	14.39	182.025070

Datum	1.03.2021
Analyse:	151985b-00
Sigel:	GHC-GA-619-01
COP: Dr. Clement Ghiazza	
Messung:	HRMS
Methode:	ESIpos
Lösungsmittel:	CH2Cl2+CH3OH
Spektrometer:	Exactive
Auswerter:	Kampen (2242)

Suggestion:
C6H6F1N1O3 MW 159

characteristical ion
182 = [159 + Na]+

^{1}H NMR – in situ

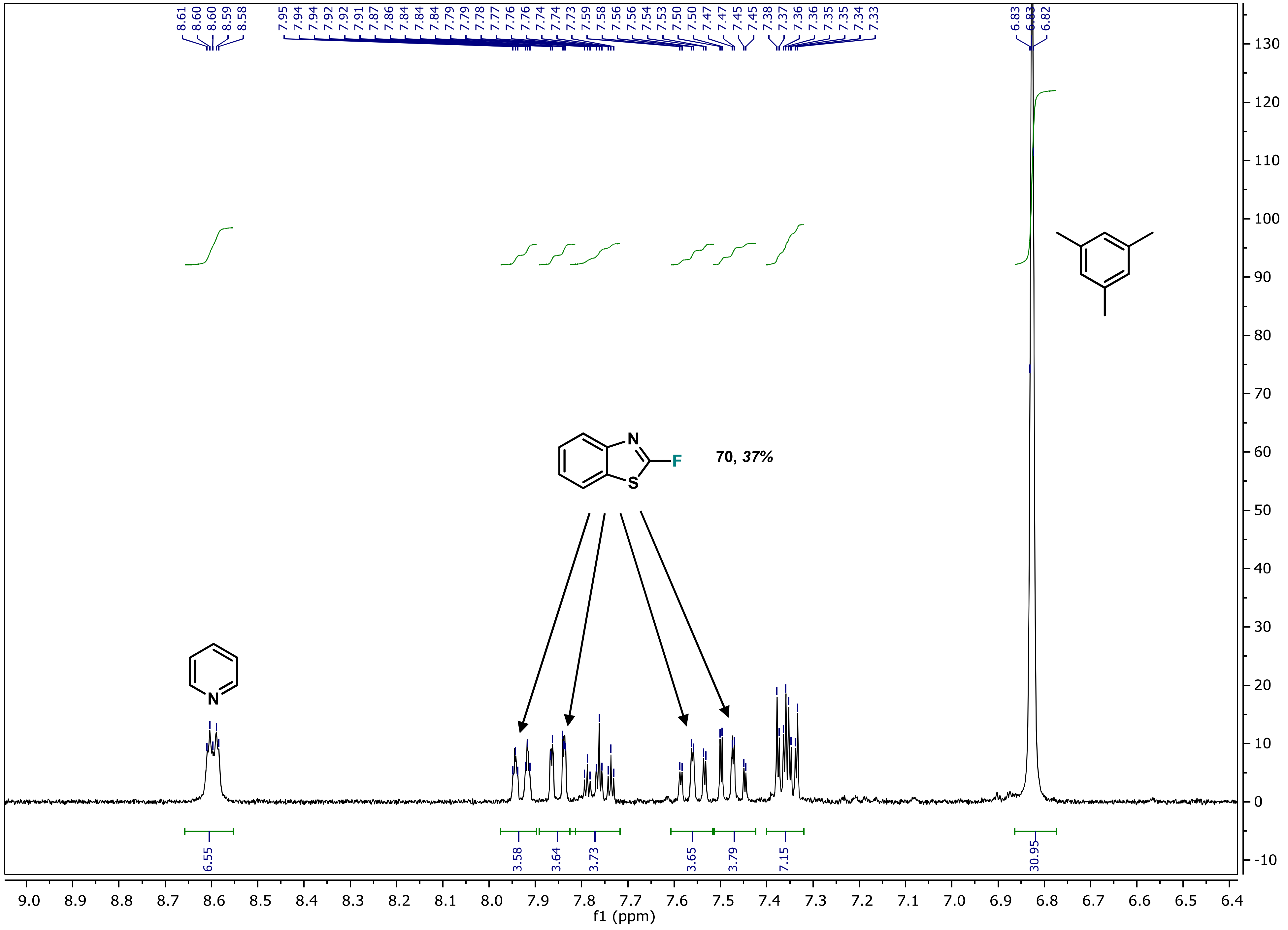

*** Angegebene Mol.-Gewichte u. Massenzahlen basieren auf dem häufigsten Isotop der Elemente ***

MassLib

Mass to be matched (m/z): 153.004340 Charge: 1

Mass Tolerance: ±0.005000
Restriction of atom numbers:

C	H	N	S	F
1-100	1-100	1-10	1-1	1-1

Number of calculated Formulas: 1

Formula	Diff.(ppm)	theor. m/z
C7 H4 N1 S1 F1	-0.26	153.004300

Datum:	9.04.2021
Analyse:	152693b-00
Sigel:	GHC-GA-676-01<br>COP: Dr. Clement Ghiazza
Method:	HR-MS
Ionis. :	GC-EI
Spectrometer:	Q-Exactive
Auswerter:	Marcus, Tel:2243

suggestion:
C7H4N1S1F1   MW: 153

^{1}H NMR – in situ

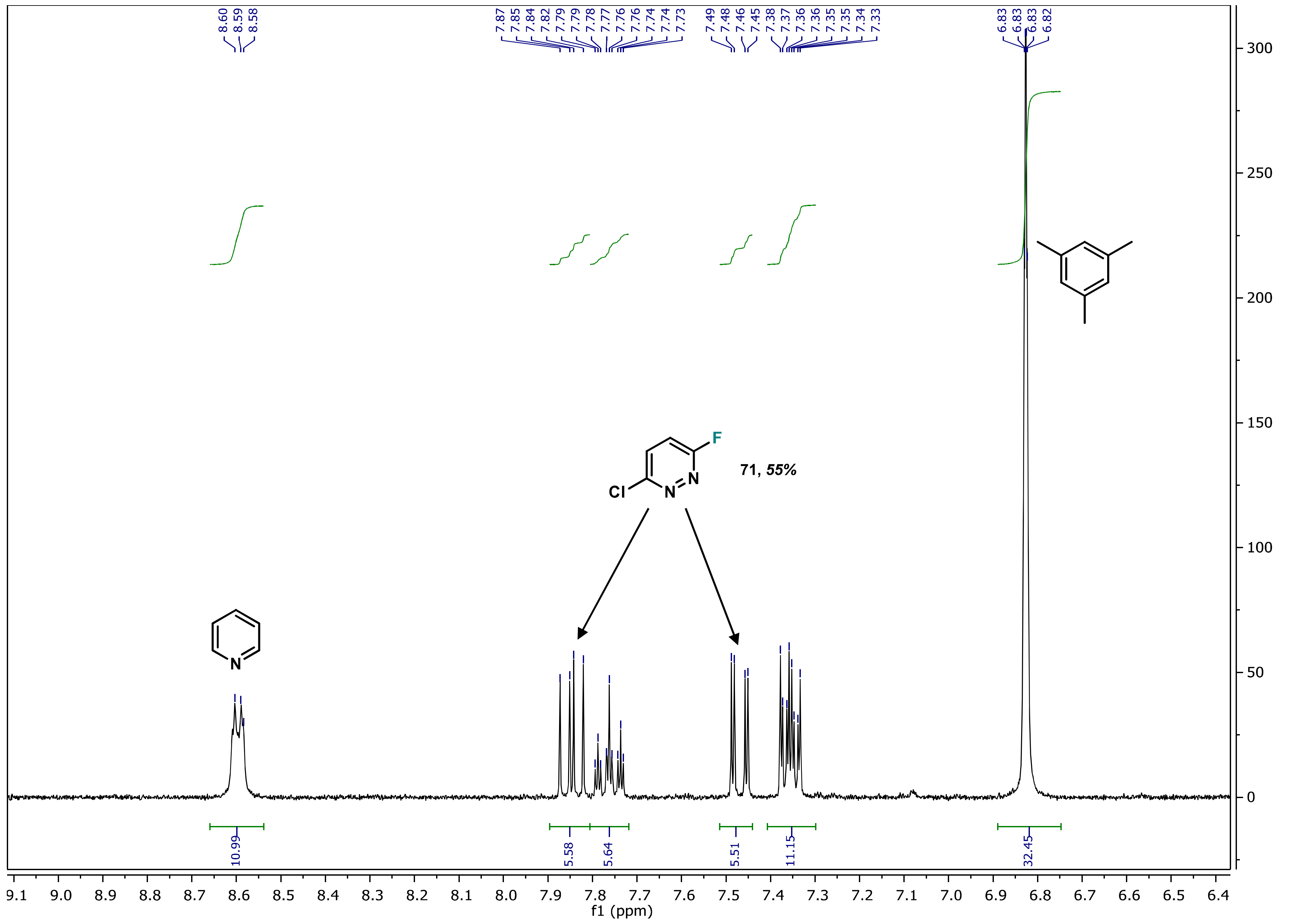

*** Angegebene Mol.-Gewichte u. Massenzahlen basieren auf dem häufigsten Isotop der Elemente ***
MassLib

Mass to be matched (m/z): 131.988720 Charge: 1

Mass Tolerance: ±0.005000
Restriction of atom numbers:

C	H	N	F	Cl
1-100	1-100	1-10	1-1	1-1

Number of calculated Formulas: 1

Formula	Diff.(ppm)	theor. m/z
C4 H2 N2 F1 Cl1	-1.64	131.988504

Datum:	9.04.2021
Analyse:	152691b-00
Sigel:	GHC-GA-679-01<br>COP: Dr. Clement Ghiazza
Method:	HR-MS
Ionis. :	GC-EI
Spectrometer:	Q-Exactive
Auswerter:	Marcus, Tel:2243

suggestion:
C4H2N2F1Cl1   MW: 132

^{1}H NMR – in situ

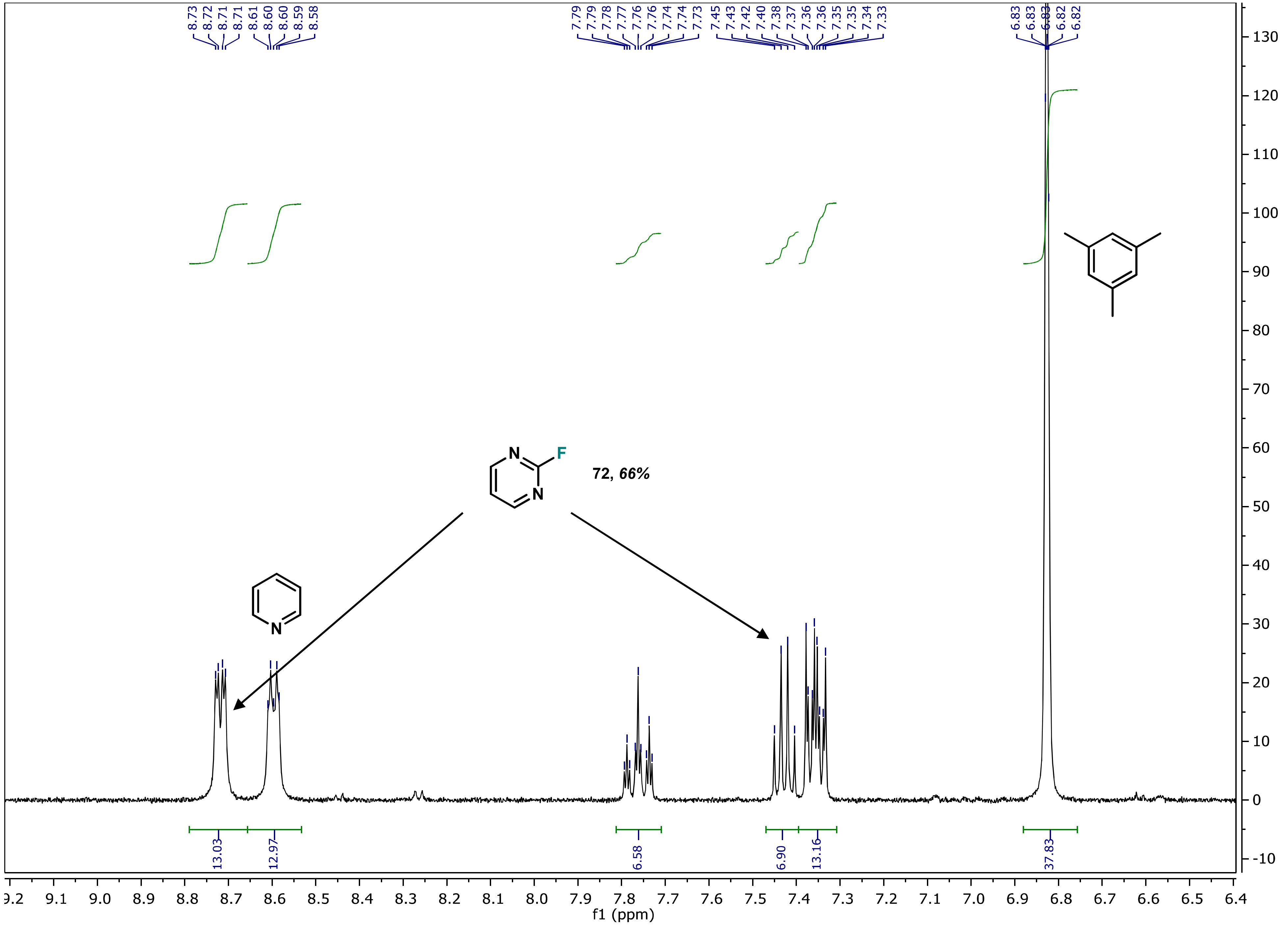

No.	MW.	Comment
1	98	Spectrum correlates with your expected structure<br>Ref.-Spektr. Nr. OE1150:
1	120	Compare OE264, OU303, Q60068

12.04.2021
File: 152709d-00.raw
Analyse: GHC-GA-681-01
COP: Dr. Clement Ghiazza

Messung:	GC-MS
Ionisierung:	GC-EI
Spektrometer:	QExactiveGC
Säule:	MS 81 ZB1ms
Länge:	30
Temp.:	35-15-300-3
GC-Nr.:	-
MS-Nr.:	30642
Auswerter:	Margold (2242)

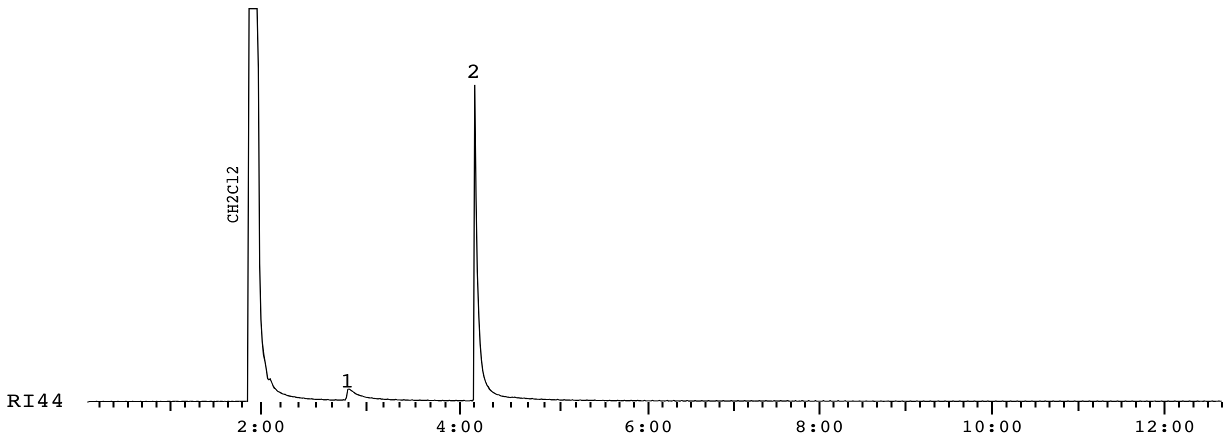

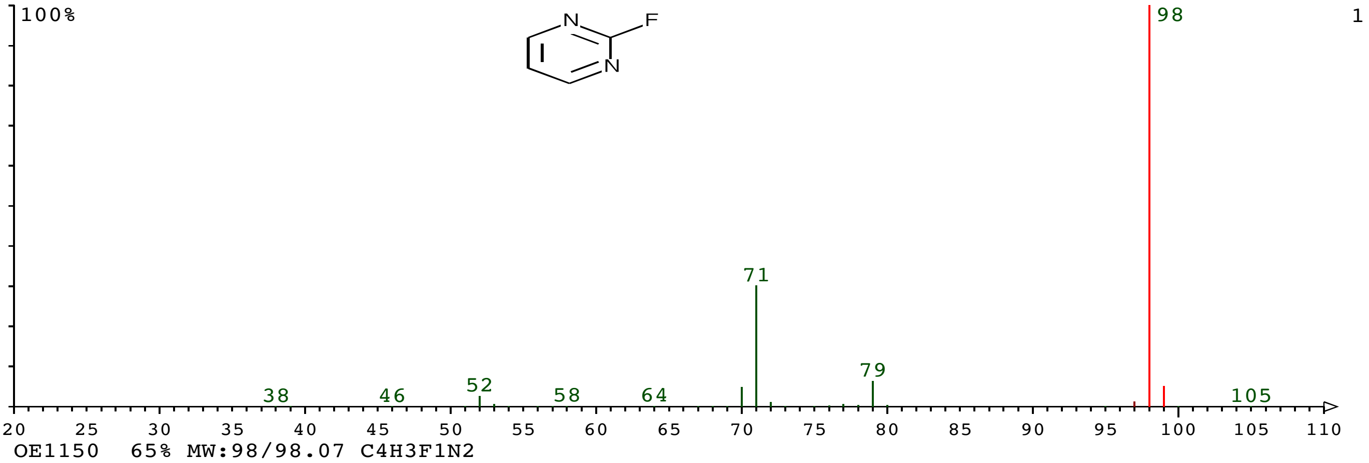

OE1150 65% MW:98/98.07 C4H3F1N2

38	0.03	78	0.39
39	0.02	79	6.34
46	0.01	80	0.29
51	0.15	95	0.05
52	2.59	97	1.15
53	0.61	98	100.00
54	0.03	99	5.10
56	0.02	100	0.08
57	0.04	105	0.01
58	0.05		
64	0.09		
67	0.05		
68	0.09		
69	0.03		
70	4.80		
71	30.14		
72	1.08		
73	0.02		
75	0.06		
76	0.17		
77	0.59		

OE1150 65% MW:98/98.07 C4H3F1N2

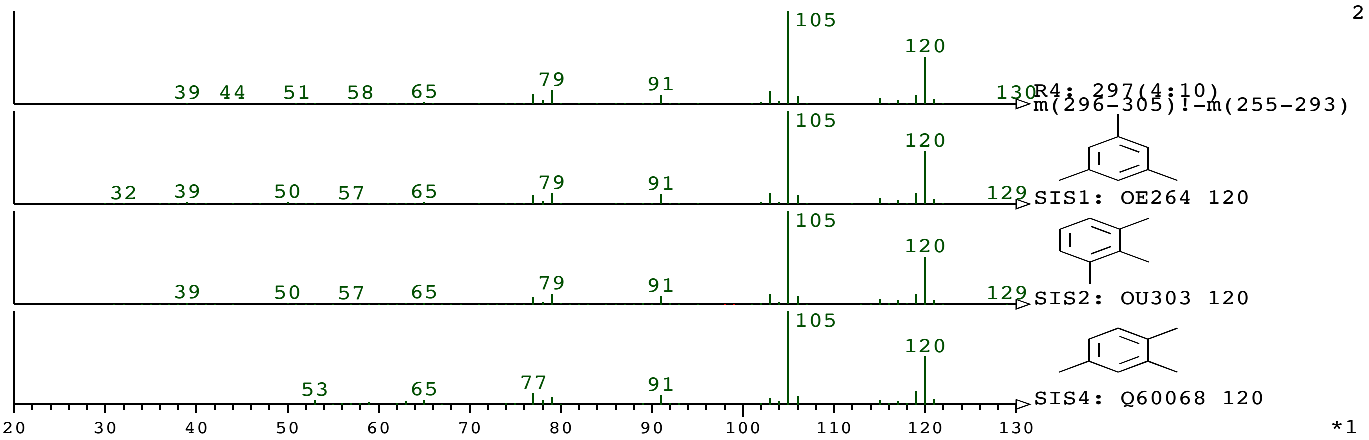

33	0.00	61	0.09	86	0.32	110	0.01	138	0.01
34	0.01	62	0.33	87	0.21	111	0.00	139	0.00
35	0.00	63	0.95	88	0.03	113	0.04	143	0.00
36	0.00	64	0.10	89	0.91	114	0.04	145	0.00
37	0.01	65	1.57	90	0.14	115	6.07	146	0.01
38	0.02	66	0.09	91	9.45	116	0.91	147	0.00
39	0.20	67	0.01	92	0.87	117	3.98	148	0.00
40	0.01	68	0.01	93	0.29	118	0.49	149	0.01
41	0.03	71	0.10	94	0.03	119	9.50	150	0.00
44	0.05	73	0.05	95	0.13	120	50.42	151	0.00
45	0.01	74	0.54	97	0.00	121	4.92	154	0.01
50	0.02	75	0.37	100	0.00	122	0.19	156	0.01
51	0.53	76	0.35	101	0.05	123	0.00	157	0.00
52	0.09	77	10.37	102	1.28	125	0.00	160	0.00
53	0.07	78	3.23	103	13.41	130	0.03	161	0.00
55	0.01	79	14.22	104	2.30	131	0.04	162	0.00
56	0.02	80	0.88	105	100.00	133	0.39	164	0.01
57	0.30	81	0.02	106	8.35	134	0.27		
58	0.37	82	0.07	107	0.29	135	0.04		
59	0.07	84	0.27	108	0.00	136	0.07		
60	0.01	85	0.04	109	0.01	137	0.00		

R4: 297(4:10) m(296-305) 152709d-00! -m(255-293) 67827 40% 152709d-00 * GHC-GA-681-01/GC-MS

Mass to be matched (m/z): 98.027610 Charge: 1

Mass Tolerance: ±0.005000
Restriction of atom numbers:

C	H	N	F
1-110	1-100	1-3	1-3

Number of calculated Formulas: 1

Formula	Diff.(ppm)	theor. m/z
C4 H3 N2 F1	-1.37	98.027476

12.04.2021
File: 152709d-00.raw
Analyse: GHC-GA-681-01
COP: Dr. Clement Ghiazza

Messung:	GC-MS
Ionisierung:	GC-EI
Spektrometer:	QExactiveGC
Säule:	MS 81 ZB1ms
Länge:	30
Temp.:	35-15-300-3
GC-Nr.:	-
MS-Nr.:	30642
Auswerter:	Margold (2242)

^1H NMR – in situ

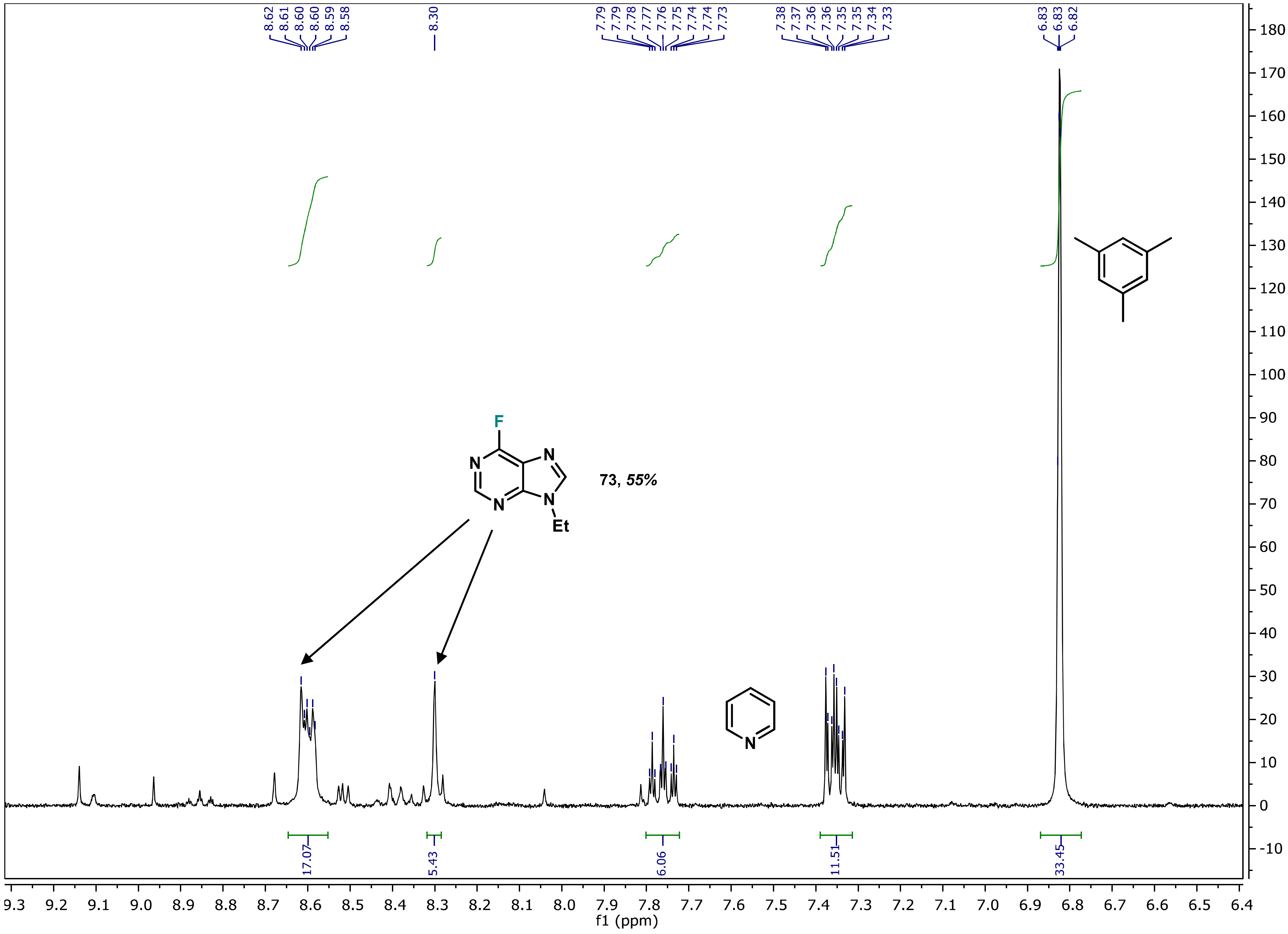

Mass to be matched (m/z): 166.065090 Charge: 1

Mass Tolerance: ±0.005000
Restriction of atom numbers:

C	H	N	F
1-100	1-100	1-10	1-1

Number of calculated Formulas: 1

Formula	Diff.(ppm)	theor. m/z
C7 H7 N4 F1	-1.00	166.064923

Datum:	9.04.2021
Analyse:	152692b-00
Sigel:	GHC-GA-675-01<br>COP: Dr. Clement Ghiazza
Method:	HR-MS
Ionis. :	GC-EI
Spectrometer:	Q-Exactive
Auswerter:	Marcus, Tel:2243

suggestion:
C7H7N4F1   MW: 166

^{1}H NMR

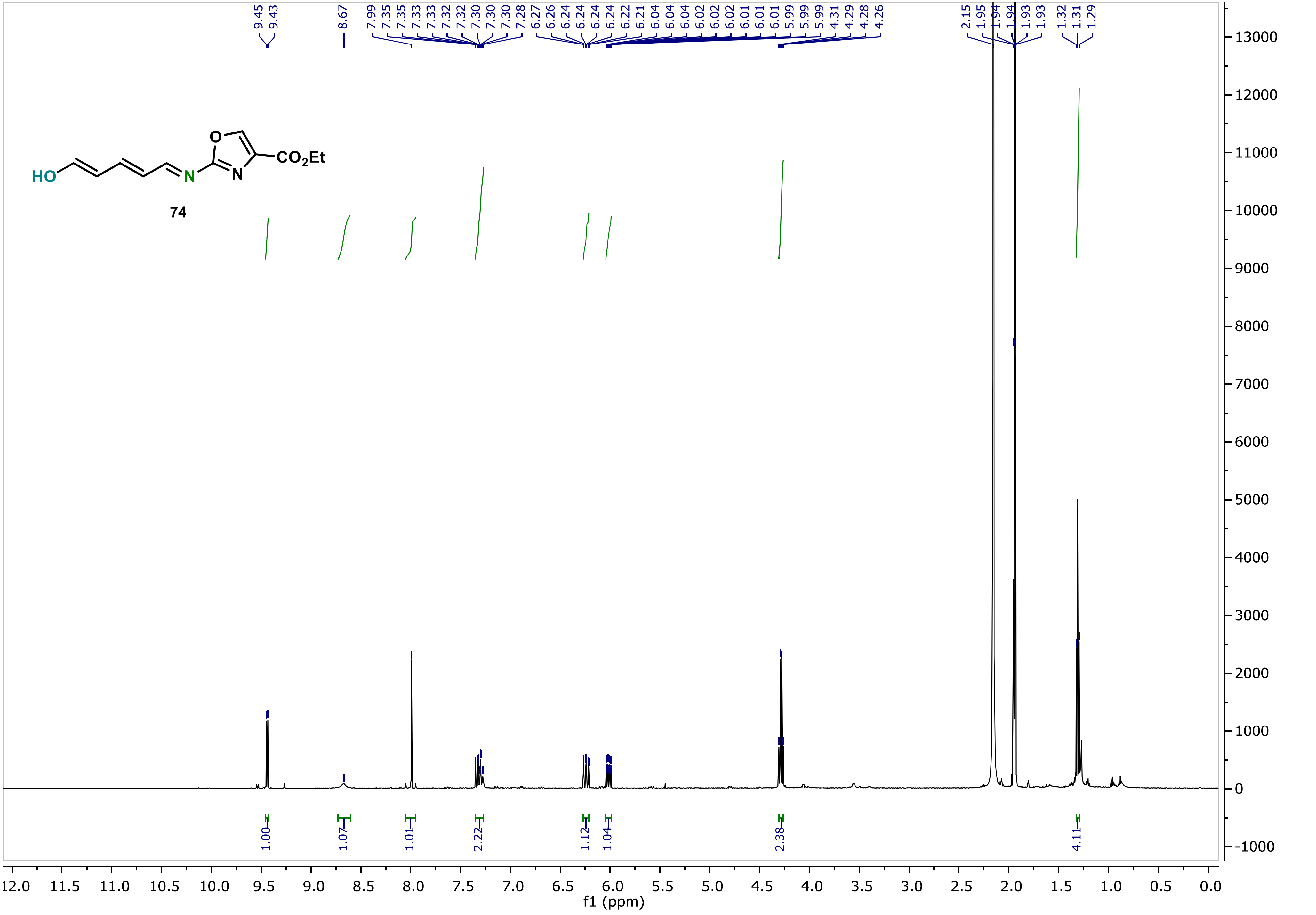

HO
N
O
N
CO2Et
74
9.45
9.43
8.67
7.99
7.35
7.35
7.33
7.33
7.32
7.32
7.30
7.30
7.30
7.28
6.27
6.26
6.24
6.24
6.24
6.24
6.22
6.21
6.04
6.04
6.04
6.02
6.02
6.02
6.01
6.01
6.01
5.99
5.99
5.99
4.31
4.29
4.28
4.26
2.15
1.95
1.94
1.94
1.93
1.93
1.32
1.31
1.29
1.00
1.07
1.01
2.22
1.12
1.04
2.38
4.11
f1 (ppm)

^{13}C NMR

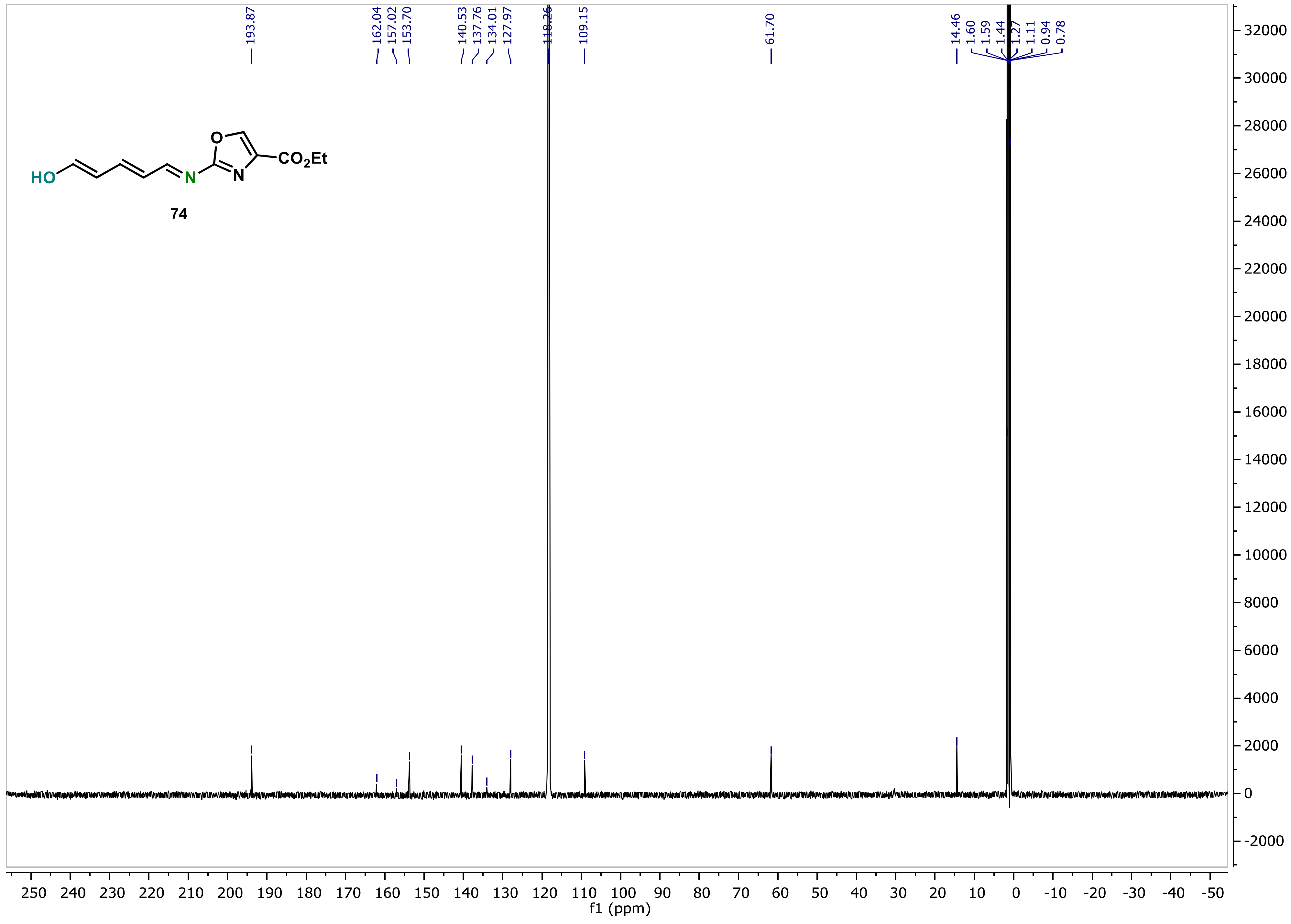

193.87
162.04
157.02
153.70
140.53
137.76
134.01
127.97
118.26
109.15
61.70
14.46
1.60
1.59
1.44
1.27
1.11
0.94
0.78
HO
N
O
N
CO2Et
74
f1 (ppm)

Mass to be matched (m/z): 259.068840 Charge: 1

Mass Tolerance: ±0.005000
Restriction of atom numbers:

C	H	N	O	Na
1-110	1-100	1-3	1-5	1-1

Number of calculated Formulas: 2

Formula	Diff.(ppm)	theor. m/z
C11 H12 N2 O4 Na1	0.33	259.068926
C14 H10 N3 O1 Na1	10.67	259.071605

03.12.2020
File: 150769c-00

Analyse: GHC-GA-464-03
COP: Dr. Clement Ghiazza

Messung:	HRMS ESIpos
Lösemittel:	CH2Cl2+CH3OH
Spektrometer:	Exactive
Auswerter:	Kohler (2243)

Suggestion:
C11H12N2O4 MW 236

Characteristicial ions:
259 = [236 + Na]+
